# Spliced leader RNA *trans*-splicing discovered in copepods

**DOI:** 10.1038/srep17411

**Published:** 2015-12-01

**Authors:** Feifei Yang, Donghui Xu, Yunyun Zhuang, Xiaoyan Yi, Yousong Huang, Hongju Chen, Senjie Lin, David A. Campbell, Nancy R. Sturm, Guangxing Liu, Huan Zhang

**Affiliations:** 1 The Key Laboratory of Marine Environment and Ecology, Ministry of Education, Qingdao 266100, China; 2 College of Environmental Science and Engineering, Ocean University of China, Qingdao 266100, China; 3 Department of Marine Sciences, University of Connecticut, Groton, Connecticut 06340, USA; 4 Department of Microbiology, Immunology & Molecular Genetics, University of California, Los Angeles, California 90095, USA

## Abstract

Copepods are one of the most abundant metazoans in the marine ecosystem, constituting a critical link in aquatic food webs and contributing significantly to the global carbon budget, yet molecular mechanisms of their gene expression are not well understood. Here we report the detection of spliced leader (SL) ***trans-***splicing in calanoid copepods. We have examined nine species of wild-caught copepods from Jiaozhou Bay, China that represent the major families of the calanoids. All these species contained a common 46-nt SL (CopepodSL). We further determined the size of CopepodSL precursor RNA (slRNA; 108-158 nt) through genomic analysis and 3′-RACE technique, which was confirmed by RNA blot analysis. Structure modeling showed that the copepod slRNA folded into typical slRNA secondary structures. Using a CopepodSL-based primer set, we selectively enriched and sequenced copepod full-length cDNAs, which led to the characterization of copepod transcripts and the cataloging of the complete set of 79 eukaryotic cytoplasmic ribosomal proteins (cRPs) for a single copepod species. We uncovered the SL ***trans-***splicing in copepod natural populations, and demonstrated that CopepodSL was a sensitive and specific tool for copepod transcriptomic studies at both the individual and population levels and that it would be useful for metatranscriptomic analysis of copepods.

Copepods are a group of small crustaceans found in various aquatic habitats. They are abundant and diverse, with over 14,000 species recognized to date^1^,^2^. As the dominant secondary producers in the oceans, copepods, especially the species in the order Calanoida, feed on microorganisms and are preyed on by fishes and some marine mammals. Therefore, they are a crucial link in aquatic food webs, transferring material and energy from primary producers to higher trophic levels^3^,^4^. Copepods are also the key players in the global carbon budget. The processes of ingestion, respiration, and egestion of copepods during the vertical migration constitute an important component of the biological pump^5^,^6^,^7^. On the other hand, some copepods are parasites of commercial fishes and thus have considerable economic impacts on aquaculture^8^. Moreover, small size, short life cycle and diverse physiology make copepods sensitive indicators of climate change and anthropogenic activities^9^.

Understanding individual as well as population performance of copepods at the molecular level is the key to gaining insights into the mechanisms regulating their growth and development, physiological functions, and response to the environmental changes^10^. In recent years, an increasing number of transcriptomic studies have been conducted with copepods at different growth stages, fed with different algae, and exposed to different stressors. These studies are revealing genes involved in the sexual differentiation, development, metabolism and molecular markers in copepods^11^,^12^,^13^,^14^,^15^. Despite the increasing data generated by various molecular approaches, transcriptomic studies on copepods still face several challenges. First, with currently available methodologies, it is impossible to separate transcripts of copepods from those of symbiotic or parasitic organisms on or under the chitinous skeleton and appendages of copepods, such as ciliates, fungi, diatoms, dinoflagellates^16^, as well as prey organisms in the gut. Second, field-collected copepod individuals often need to be brought to the laboratory for sorting, species identification and rearing, making transcriptomic data obtained from these individuals not representative of the *in situ* gene expression in the natural assemblages. Third, as most copepods are very small in size (0.5–2 mm)^3^, great effort is required to obtain enough starting material for transcriptomic profiling of wild-caught samples (usually several micrograms of total RNA are required)^7^. These limitations can be addressed if a selective tool to obtain copepod-specific transcripts is available.

mRNA spliced leader (SL) is a potential lineage-specific tag used in generating full-length cDNA libraries for sequencing. SL is a short (usually 15–50 bp) and conserved non-coding RNA fragment that is *trans*-spliced to the splice acceptor site in the 5′-untranslated region of a nuclear encoded mRNA^17^,^18^. SL *trans*-splicing has been found in a number of phylogenetically disjointed eukaryotes such as trypanosomes, flatworms, hydra, ctenophores, rotifers, chordates, dinoflagellates and euglenoids^18^,^19^,^20^,^21^. While the mechanism of SL *trans*-splicing is presumably similar in these different groups of organisms, the SL sequence is highly lineage specific. Thus, SL can be a powerful tool for the transcriptomic work of a specific lineage, especially for metatranscriptomic research, as has been demonstrated in dinoflagellates and euglenoids^18^,^21^,^22^,^23^. Moreover, full-length cDNAs could be easily retrieved by PCR amplification using an SL-based primer paired with a common 3′-end sequence incorporated in a modified oligo-dT used in cDNA synthesis, such as the Racer3′ primer^18^. Full-length cDNA sequences have proven to be informative^24^,^25^,^26^,^27^, as complete coding regions allow for more accurate functional annotations and gene identification in the genome. This is especially true for gene function prediction of non-model species or field-collected samples when a large portion of the transcriptome sequences do not have clear hits to the reported sequences in DNA and protein databases^18^,^27^.

However, proof of the existence of SL *trans*-splicing in a given lineage of organisms is not readily available from the existing transcriptomic data reported in the databases. Because most of these data were not obtained using methods specifically targeting the full-length sequence of the transcripts, the sequence information of the very 5′-end where the SL resides is missing or truncated in almost all cases (see Zhang *et al*.^27^ and the references therein). Douris *et al*.^20^ was the first attempt to find SL in copepods, in which the authors surveyed the copepod expressed sequence tag (EST) data reported in GenBank and found five common sequences at the 5′-end of some of the ESTs, two associated with the parasitic copepods *Caligus rogercresseyi* and *Lepeophtheirus salmonis*, and the other three for a free-living calanoid copepod *Calanus finmarchicus*. They proposed these to be SL-like sequences in copepods. However, without experimental validation, it is unclear whether they represent typical copepod SL, and if so, whether they are ubiquitously *trans*-spliced to all nuclear encoded mRNAs.

In this study, we investigated the SL *trans*-splicing of nine species of copepods wild-caught from Jiaozhou Bay, China, covering seven families of an important copepod order, Calanoida, which are dominant zooplankton in the ocean^3^. We identified one typical copepod SL shared by these nine species, which was strikingly different from the previous computationally-predicted copepod SL. We characterized the structures of several copepod slRNA genes and predicted their RNA secondary structures. Through SL-based cDNA library construction and sequencing, we showed that this copepod SL could be a sensitive and specific tool in calanoid copepod transcriptomic studies.

## Results

### SL, slRNA and coding genes in calanoid copepods

**Figure 1:**
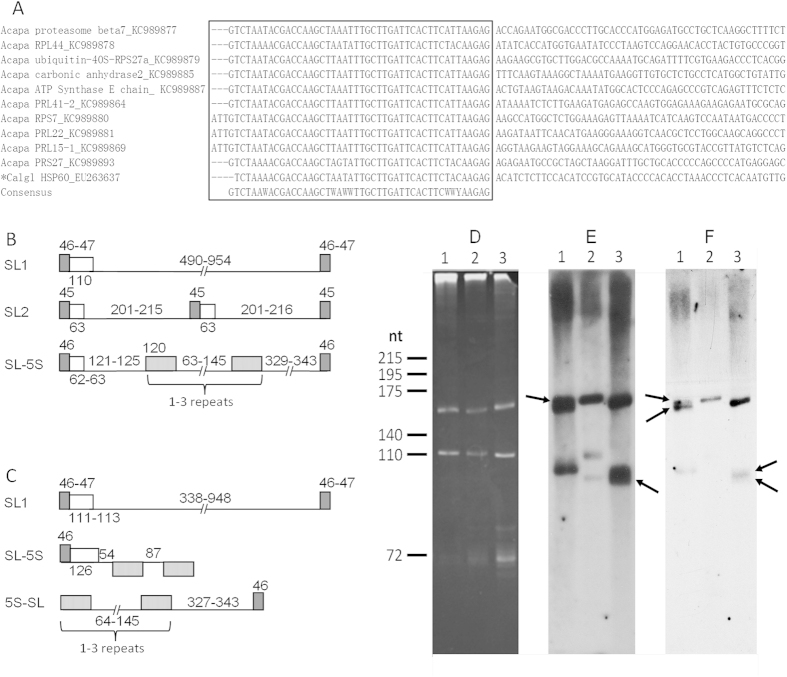
(**A**) Example of CopepodSL identified in various cDNAs recovered from *Acartia pacifica* GeneRacer library (Acapa) and a previously reported *Calanus glacialis* heat shock protein 60 cDNA sequence (*Calgl HSP60). (**B,C**) The genomic structures of the major types of the CopepodSL encoding genes in *A. pacifica* (**B**) and *P. poplesia* (**C**). (**D**,**E**,**F**) Image of total RNAs from *A. pacifica* (lane 1), *Calanus sinicus* (lane 2) and *Pseudodiaptomus poplesia* (lane 3) electrophoresed in an 8% acrylamide and 8M urea gel (**D**), and the Northern Blots hybridized with ^32^P-labeled CopepodSLa/s (**E**,**F**). Arrows indicate the apparent single thick bands under milder washing condition (E; 2 × SSC at 43ºC) were composed of two bands with slightly different sizes as seen under stringent washing (F; 2 × SSC at 50ºC). Total RNA from *Leishmania tarentolae* cells was co-electrophoresed (not shown) to provide known size markers^18^.

Approximately 2 μg of *Acartia pacifica* mRNA was used to construct a library with enriched full-length cDNAs using a GeneRacer^TM^ Kit (Invitrogen, USA). Out of 327 high-quality sequences, 124 sequences contained a complete coding region, totally representing 61 unique genes^7^. Thirty-three of these different transcripts, encoding various proteins such as ribosomal proteins, 14-3-3, carbonic anhydrase 2, and proteasome subunit beta type 7 precursor, had a nearly identical 46-nt sequence at the beginning of the 5′-untranslated region (UTR), indicating that this 46-nt sequence (named CopepodSL hereafter, Table 1) is a spliced leader *trans*-spliced to the 5′-end of mRNAs in *A. pacifica* (Fig. 1A). Three of the full-length cDNAs had an additional three nucleotides, ATT, at the 5′-end of the CopepodSL.

To verify the authenticity of CopepodSL, we isolated and characterized the RNA that donates SL to mRNAs (i.e., precursor RNA of SL, or slRNA) and its template genes. We obtained 54 and 12 cDNA clones of slRNA for *A. pacifica* and *Pseudodiaptomus poplesia* respectively, which can be grouped into 7 or 4 types (GenBank accession # KT758633 to KT758698). Nucleotide substitutions and insertions/deletions (indels) were detected in both exon and intron regions. The exon of slRNA (i.e. CopepodSL) was 46-nt long in most of the clones, although in some cases it was 44-, 45- or 47-nt long. Intron length differed among the types, ranging from 62 to 111 nt, making the range of slRNA length 108–158 nt in *A. pacifica*, and 110–158 nt in *P. poplesia*. Detailed sequence information of the slRNA transcripts obtained in this study was shown in Table S1.

We further isolated and characterized the genes for copepod slRNAs (GenBank accession # KT755459 to KT755647) from *A. pacifica* and *P. poplesia*. The gene organization for various types of slRNAs in *A. pacifica* and *P. poplesia* were either arrayed as tandem gene repeats with various lengths of spacer sequence between each unit, or clustered with a 5S rRNA gene (5S rDNA), a genomic organization found in slRNA of many organisms^17^,^19^,^30^ (Fig. 1B,C). Various nucleotide indels were found among each unit of slRNA genomic clones, mostly in intron and intergenic spacers, similar to the report in dinoflagellate slRNAs^17^,^30^.

To confirm the size of the sequenced slRNA, we performed RNA blot analysis for *A. pacifica*, *P. poplesia* and *Calanus sinicus*. Since the intron of slRNA is highly polymorphic with indels, we chose the conserved region at the 5′-end of CopepodSL to design a reverse-complement 18-nt sequences as the probe for hybridization (Table 1). For all three species, several bands of hybridization were detected in the range of 110 nt to 175 nt (Fig. 1D,E), sizes consistent with that of the slRNA transcripts described above.

The putative secondary structures of the major types of copepod slRNAs predicted by modeling program MFOLD (See supplementary Fig. S1 online) were similar to those commonly predicted in various eukaryotes: a stem-loop structure formed by the exon and the beginning portion of the intron that contains the splice donor dinucleotide “GU”, followed by two stem-loops that flank a single-stranded region, in which the binding site of RNA binding proteins (Sm-protein complex) is located^28^. In some cases (ApaSL4, ApaSL5), one additional stem-loop was found before the single strand region, a structure that also has been reported in the alterative modeling of several slRNAs from other organisms using different programs^29^.

The copepod slRNA also has its unique structure, for which all types but one (PpoSL2) have two additional stem-loops in the exon region besides the stem-loop formed by exon-intron. This could be due to copepod slRNA having a longer exon (44–47 nt, majority 46 nt) than that of the other eukaryote slRNAs (mostly 16– <40 nt), making it easy to form secondary structures. In each slRNA type, the putative Sm-binding site was found at a similar location in the single stranded region of the intron; however, the sequence and the length varied slightly among the types (See supplementary Fig. S1 online). The Sm-binding site variants in copepods usually have the sequence of [RR (U + C) _6-12_RR], with one “A” or “G” in the U-C track in some types; they are generally longer than the conserved eukaryote sequence RR (U + C) _4–6_RR^29^.

### CopepodSL *trans*-splicing is wide-spread in, and specific for, calanoid copepod mRNAs

**Figure 2:**
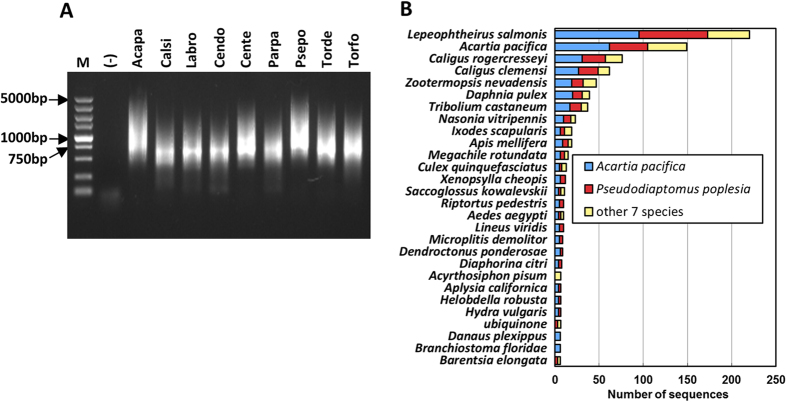
(**A**) The gel image of the PCR products generated with primer set CopepodSL-Racer3. Left, lane M, DL5000 DNA Marker; (−), negative control; Acapa, *Acartia pacifica*, Calsi, *Calanus sinicus*, Labro, *Labidocera rotunda*; Cendo, *Centropages dorsispinatus*; Cente, *Centropages tenuiremis*; Parpa, *Paracalanus parvus*, Psepo, *Pseudodiaptomus poplesia*, Torde, *Tortanus dextrilobatus* and Torfo, *Tortanus forcipatus*. (**B**) Overall annotation result based on BLASTx hits (with E-value cutoff <10^−5^) of the nine copepod cDNAs against the NCBI nr database. No cDNAs from the potential organisms living on copepod exoskeleton were detected.

For a SL to be useful for transcriptomic studies, it has to exist in most, if not all, mRNAs of a species. To verify the ubiquity of CopepodSL in copepod mRNAs, we synthesized cDNAs for nine wild-caught copepod species representing seven families of the order Calanoida. PCR using CopepodSL-Racer3 primer set amplified cDNAs of 0.3–3 kb for all nine species, with 0.6–1 kb being the most abundant regardless of the difference of the original RNA quantity used in cDNA synthesis (Table 1, Fig. 2A). This indicated that the primer set CopepodSL-Racer3 was able to efficiently amplify cDNA libraries synthesized with very small amount of starting material (equivalent to 0.25 to 25 ng of total RNA). These cDNA amplicons were purified and cloned. We sequenced approximately 900 clones each for *A. pacifica* and *P. poplesia*, and 50–70 clones each for the other seven copepods. In total, 1,288 unique sequences were obtained (GenBank accession # KT754169 to KT755456). Rarefaction analysis^31^ indicated that the transcriptomic diversity has not been sampled exhaustively (See supplementary Fig. S2 online). Despite the limited sequencing depth, these cDNA datasets provided complete coding regions (cds) of the genes facilitating further gene characterization and functional annotation. These sequences were screened against the GenBank database, and 95% of them were identified as full-length cDNAs, while 5% had a complete 5′-end but missed the 3′-end likely due to the nonspecific binding of oligo-dT primer to a A-rich non-poly(A) tail region during cDNA synthesis. BLAST analysis of the cDNAs hit various protein genes mostly of copepods, insects and other animals; none of the cDNAs had top hits to genes of the organisms that were potential copepod prey (e.g., phytoplankton), or the symbionts living on or under the exoskeletons of copepods, such as ciliates and fungi^16^ (Fig. 2B, Supplementary Table S2 online). About 89% of *A. pacifica* and 84% of *P. poplesia* cDNAs were assigned putative gene function based on BLASTx search at E-value < 10^−5^. Gene ontology analysis showed diverse functions in each library (See Supplementary Fig. S3 online).

To enlarge the scope of our investigation to see whether CopepodSL exists in the transcripts of the other calanoid copepods as well as the copepods of the other orders, which were not available in the present study, we further queried the existing transcriptomic and genomic data sets (>100 million entries) for copepod sequences reported in GenBank. The available data were from four copepod orders, including Calanoida, Siphonostomatoida, Cyclopoida and Harpacticoida. Partial CopepodSL sequences were detected at the 5′-end of 1220 cDNAs, ESTs or TSA contigs, 1156 of which came from calanoid species, including *C. finmarchicus*, *C. helgolandicus, C. glacialis and Eurytemora affinis* (See Supplementary Table S3a1 online). We further performed gene ontology analysis for the 1121 sequences of *C. finmarchicus*. Similar to what we found in *A. pacifica* and *P. poplesia*, these sequences also had very diverse functions (See Supplementary Fig. S3 online).

Our search also yielded 64 unnamed sequences with partial CopepodSL from the metatranscriptomes of the microbial community in fish intestines, multiple tissue cDNA libraries of dogfish shark and green shore crab, eukaryotic metatranscriptome of sea ice, and whole animal cDNA libraries of amphioxus and ctenophore. Fifty three of these unnamed sequences had the top hit only to copepod transcripts in blast analysis, while the remaining 11 sequences had no hit to any known sequences (See Supplementary Table S3a2 online), suggesting that these sequences were from copepods living on (epizootic), or eaten by those predators.

Douris *et al*.^20^ proposed five potential SL-like sequences in copepods (referred to as CopepodSL-like1a, 1b, 2a, 2b, 2c in this study; Table 1) solely through *in silico* analysis of ESTs. To experimentally test whether these CopepodSL-like also exist in the calanoid copepods used in this study, we paired these sequences with Racer3 in PCR using the cDNAs of the nine calanoid species as templates. No positive result was obtained in any of these species.

We further used these five SL-like sequences to query against the existing transcriptomic and genomic data sets mentioned above. Complete or partial sequences of CopepodSL-like1a and CopepodSL-like1b were found in 228 sequences (CopepodSL-like1a 157 hits and CopepodSL-like1b 71 hits, respectively), 215 of which were ESTs/cDNAs of siphonostomatoid copepods including *L. salmonis*, *Caligus clemensi* and *C. rogercresseyi* (all being fish parasites); 13 of the hits were of other organisms (See Supplementary Table S3b online). On the other hand, the CopepodSL-like2a, 2b, and 2c were not specific to copepod transcripts. For CopepodSL-like2a, 28 of the 45 sequences hit copepod (*C. finmarchicus*) cDNAs; for CopepodSL-like2b, 16 of the 28 hit *C. finmarchicus*; for CopepodSL-like2c, only 3 of 15 hits were of *C. finmarchicus* with two of them overlapping the sequences hit by CopepodSL-like2b (See Supplementary Table S3c online). No significant hits of either CopepodSL or CopepodSL-like sequences were found in the datasets of cyclopoid and harpacticoid copepods. These results suggest that previously reported copepod SL sequences are *trans*-spliced rarely or exclusively in parasitic copepods, if at all.

### Sm-Complex subunits and splicing factors

The Sm proteins assemble around the Sm-binding site to form a heptameric ring and facilitate the assembly of the core snRNP responsible for RNA splicing^32^. Six cDNAs that encode proteins similar to the known Sm-binding proteins (E-value <10^−94^ to 10^−38^) have been detected. Two of them, U2 small nuclear ribonucleoprotein A (KT754249) and U6 snRNA-associated Sm-like protein LSm3 (KT754509), were detected in *A. pacifica*; two, NHP2-like protein 1 (KT754865) and U6 snRNA-associated Sm-like protein LSm1, in *P. poplesia* (KT755114). LSm1 was also detected in *Paracalanus parvus* (KT755333), and LSm3 in *Tortanus forcipatus* (KT755442). Two cDNAs showing high similarity to ATP-dependent RNA helicase DDX39/ spliceosome RNA helicase BAT1 of insects and parasitic copepods (E-value <10^−132^) were identified in *A. pacifica* (KT754357, KT754358). Spliceosome RNA helicase BAT1 is a member of the DEAD box family of RNA-dependent ATPases that mediate ATP hydrolysis during pre-mRNA splicing^33^, an essential splicing factor that is required for the association of U2 small nuclear ribonucleoprotein with pre-mRNA, and plays an important role in mRNA export from the nucleus to the cytoplasm. The presence of the Sm-binding protein subunits as well as spliceosome RNA helicase BAT1 indicates that mRNA *trans*-splicing mechanism in copepods is similar to that in other organisms.

### Unique characteristics of copepod mRNA

From our CopepodSL-containing cDNA data, we found that copepod full-length cDNAs have GC content below 50%, with an average of 45.6% in *A. pacifica* and 47.7% in *P. poplesia.* This is due to the high number of single nucleotide repeats (SNR; >3 repeats) dominated by “A” repeats, followed by “T”, “C” and “G” in abundance. In some cases, the SNR were up to 12 times.

We analyzed the size distributions of the full-length cDNAs obtained from the CopepodSL-based libraries, the cds and the 5′- and 3′- untranslated regions (UTRs). For *A. pacifica* and *P. poplesia*, the average cDNA length for 509 and 437 unique genes was 878±457 bp and 721 ± 287 bp, cds 553 ± 373 bp and 457 ± 255 bp, 5′-UTR 24 ± 36 bp and 17 ± 26 bp, and 3′-UTR 249 ± 209 bp and 192 ± 163 bp, respectively. The detailed codon usage of the cDNAs can be found in Table S4 online. Generally, copepod transcripts have short 5′-UTR and long 3′-UTR.

For *A. pacifica* and *P. poplesia* cDNAs, the sequence flanking the putative start codon AUG was investigated for the presence of the Kozak-like sequence^34^ that plays an important role in the initiation of the translation process in eukaryotes. We found that copepods used a consensus sequence, AAAAUGGCU, in their mRNAs (See supplementary Fig. S4 online).

The consensus polyadenylation signal AAUAAA, the binding site recognized by the RNA cleavage complex, was detected only in about half of the copepod cDNAs. Poly(A) was added 7–20 nucleotides downstream of the binding site, similar to what was found in the mRNA of other eukaryotes^35^, although in some cases poly(A) tail was added several tens to hundreds of nucleotides downstream of the binding site. Many genes (e.g. RPL27a, RPL11) have transcripts with different length of 3′-UTR, and AAUAAA exists in some cDNAs but is absent in others. Some cDNAs (e.g. Cu/Zn SOD, RPS27) have multiple polyadenylation signals.

### Complete set of cytoplasmic ribosomal proteins (cRPs) in copepods

cRPs are critical components of gene translation machinery, but have not been systematically documented for a single copepod species. Many of the full-length cDNAs were retrieved from the sequenced GeneRacer-based library of *A. pacifica* encoded cRPs. Similarly, cRP cDNAs were abundant in the SL-based full-length libraries for all the nine copepods included in this study. We have obtained full-length cDNA sequences for 67 and 70 cRP genes from the *A. pacifica* and *P. poplesia* cDNA libraries, respectively. Compared to typical eukaryotic set, some cRPs were missing. To search for these missing members, we used an SL-based technique to PCR amplify the nine RP full-length cDNAs^18^. As a result, a total of 79 cRP genes were identified from *P. poplesia*, and from the combined cDNA data of the other copepod species included in this study. The sequence details are to be published elsewhere^36^.

## Discussion

Spliced Leader *trans*-splicing has been found in a number of phylogenetically-disjointed eukaryotes^21^,^29^, and the present study adds a significant lineage to the list. CopepodSL is a *bona fide* addition because 1) the conserved sequences occur at the 5′ end of mRNAs of diverse genes in calanoid copepods, 2) the gene encoding CopepodSL was isolated, and RNA blot analysis confirmed the existence of corresponding RNA, and 3) structural simulation showed typical folding of slRNA.

Although five spliced leader-like sequences have previously been identified through *in silico* analysis of the existing copepod EST data^20^, they were very different from our CopepodSL. Furthermore, they do not seem to occur in the calanoid copepods we examined as PCR using these CopepodSL-like sequences as the forward primer failed to amplify any of the cDNA templates. Our further BLAST analyses of these sequences against GenBank databases indicated that two CopepodSL-like sequences, CopepodSL-like1a and CopepodSL-like1b, hit cDNAs only of the parasitic siphonostomatoid copepods (215 out of 228 when the hits of the two sequences were combined; see Supplementary Table S3b online); while the other three sequences, CopepodSL-like 2a, 2b and 2c only hit a small number of cDNAs of calanoid copepod *C. finmarchicus*, and the cDNAs of other organisms (45 hits to *C. finmarchicus* out of 88 total hits; see Supplementary Table S3c online). In contrast, our CopepodSL had 1,220 hits; 1,156 of them were of copepods including *C. finmarchicus*, *C. helgolandicus, C. glacialis and E. affinis*, and 53 of them were of mixed cDNAs with top hit only to copepod transcripts (See Supplementary Tables S3a1 and 3a2 online). Of the copepod hits, 1,121 sequences were of *C. finmarchicus*. These data strongly suggest that our CopepodSL is the universal SL in calanoid copepods, CopepodSL-like1a and 1b are probably the actual types of SL of the parasitic siphonostomatoid copepods, while CopepodSL-like2a, 2b and 2c are likely not the real types of SL in *C. finmarchicus*.

It is interesting to note that neither CopepodSL, nor the CopepodSL-like sequences hit any sequences from the copepods in the other two orders, Cyclopoida and Harpacticoida. Further studies are needed to elucidate whether these two orders of copepods have their unique (order-specific) SL, or they if share SL with calanoid or siphonostomatoid copepods.

Our data indicate that CopepodSL can be used as a specific and sensitive tool for *in situ* calanoid copepod transcriptomic studies at both the individual and population level. The CopepodSL-based amplification method is highly specific for constructing copepod-specific cDNA libraries. All the sequences we obtained using CopepodSL, and those sequences reported in GenBank database with CopepodSL at the 5′-end are clearly of copepod origin and not from the contamination of food organisms in the gut, or the symbionts on the surface. This method is also very sensitive. Full-length cDNA can be amplified using the 1^st^ strand cDNA template synthesized from as little as 10 ng total RNA (equivalent to 1/10 to 1/5 of the RNA obtained from an individual small-sized copepod such as *A. hudsonica*^7^). The 0.3–3 kb smear of the SL-enriched cDNAs for all nine species shown in the gel image (Fig. 2A) together with the functional diversity of these cDNAs and the 1121 CopepodSL-containing *C. finmarchicus* cDNA sequences obtained from GenBank strongly indicate that CopepodSL is ubiquitous at least in the transcriptomes of calanoid copepods.

The application of this approach in the current study, albeit on a small scale, yielded interesting results. The ability to generate a quality cDNA library from as little as one copepod individual for sequencing by either traditional cloning methods or next generation sequencing techniques clearly demonstrates its usefulness in the transcriptomic study. The comparison of gene expression at different development stages or under various environmental conditions will be greatly facilitated. This will be particularly important for the transcriptomic study of copepod samples collected in the field when the sample size of the copepod species of interest is often limited. As the sequencing technique advances and the copepod dataset grows, an in-depth understanding of how an individual copepod genome is expressed *in situ* is achievable in the future.

Seventy to 80% of the unigenes were represented by 1-2 cDNAs, i.e., expressed at a uniform background level. Most of the highly represented genes are those associated with essential cellular processes, especially the cRPs. Generally there are 32 small subunit (40S) and 47 large subunit (60S) cRPs in eukaryotes (excluding the plant-specific RPLP3 and human Y-chromosome specific isoform of RPS4) with few exceptions. Until now, no copepod species has its complete cRPs set documented^37^. We used the previously developed SL-based RACER method^18^ and successfully obtained all 79 cRP genes from the copepod *P. poplesia*. This is the first report for the complete set of cRP genes from a single copepod. Interestingly, although both cRPs and mitochondrial RPs (mRPs) are nuclear-encoded^38^, cRP genes in copepods were highly expressed, while mRP genes were expressed at lower levels; only five [L14 (KT754492), S2 (KT754321), S15 (KT754325), S16 (KT754507, KT754508), S17 (KT754448, KT754449), five [L16 (KT754818), L36 (KT754873), L42 (KT754840, KT755125), L55 (KT755120), S23 (KT755093)], one (L54; KT755291) and one (L17; KT755357) mRP cDNAs were obtained from *A. pacifica*, *P. poplesia*, *L. rotunda*, and *T. dextrilobatus*, respectively, and each had only 1-2 clones. This may be because cytoplasmic protein synthesis is much more active than mitochondrial protein synthesis.

## Methods

### Copepod sampling and nucleic acid isolation

Live copepods were collected at Jiaozhou Bay, Shandong Province, China (36°02′60″N, 120°20′52″E) and sorted to species in May and June, 2012; total RNA of each species was isolated as reported previously^7^. Nine copepod species from seven families in the order Calanoida were obtained: *Acartia pacifica, Calanus sinicus, Centropages dorsispinatus, Centropages tenuiremis, Labidocera rotunda* (previously *L. bipinnata*)*, Paracalanus parvus, Pseudodiaptomus poplesia, Tortanus dextrilobatus* and *Tortanus forcipatus*. For *A. pacifica*, ~8000 individuals were used for RNA isolation.

Genomic DNA was isolated from *A. pacifica* and *P. poplesia* individuals collected as described above. About 100 individuals of each species were pooled into a 1.5 mL microtubes containing 100 μL DNA buffer (0.1 M EDTA pH 8; 1% SDS) and homogenized using a pestle as reported^7^. After homogenization, 300 μL DNA buffer containing 40 μg Proteinase K (TaKaRa, Japan) was added to each sample and incubated at 55 °C for 24 h. DNA was extracted and purified using DNA Clean & Concentrator (Zymo Research, USA) as reported^39^.

### Enriched full-length cDNA library construction for *A. pacifica*

Approximately 2 μg of mRNA was isolated from 250 μg total RNA of *A. pacifica* using PolyATract mRNA Isolation System III Kit (Promega, USA) following the manufacturer’s protocol. An enriched full-length cDNA library was synthesized using GeneRacer^TM^ Kit (Invitrogen, USA) and the cDNAs were cloned and sequenced following Kuo *et al*.^21^. The cDNA sequences were aligned using ClustalW to identify the potential SL sequence at the 5′-end of the cDNAs. A common 46-nt sequence found in the 5′ UTR of 33 cDNAs was used to query databases including Nucleotide collection (nr/nt), Expressed sequence tags (EST), Transcriptome shotgun assembly (TSA) and Whole-genome shotgun contigs (WGS) at the National Center of Biotechnology Information (NCBI) (http://www.ncbi.nlm.nih.gov/) using BLASTn.

### PCR amplification and sequencing of full-length cDNAs for nine copepods using CopepodSL

Ten to 1,000 ng total RNA of the nine copepod species isolated from 1–100 individuals was used as the template for first strand cDNA synthesis using GeneRacer^TM^ Oligo dT (Invitrogen, USA). The obtained cDNA libraries were purified using DNA Clean & Concentrator (Zymo Research, USA) as reported^18^, and eluted in 40 μl of 10 mM Tris·Cl (pH 8). One μl of the cDNA was used as the template with CopepodSL paired with GeneRacer™ 3′ Primer (Racer3) to PCR-amplify the cDNAs for all nine copepod species (Table 1). The following PCR program was used: an initial denaturing step at 95 °C for 2 min; followed by 10 cycles at 95 °C for 15 sec and 68 °C for 4 min; and 20 cycles at 95 °C for 15 sec, 60 °C for 30 sec and 72 °C for 3 min 30 sec. PCR products were cloned and sequenced. For each species, the amplicon was cloned into a pCR^®^2.1-TOPO^®^ vector following the manufacturer’s protocol (TOPO TA Cloning® Kit, Invitrogen, USA), and 50-900 resultant clones were randomly picked up and sequenced as reported^18^.

The five CopepodSL like sequences reported by Douris *et al*.^20^ were tested using the same method described (See Table 1 for the primer sequences). Due to the lower Tm of these primers in comparison to CopepodSL, the following PCR program was used: 1 cycle of 95 °C for 2 min; followed by 10 cycles at 95 °C for 15 sec and 60 °C for 4 min; and 20 cycles at 95 °C for 15 sec, 58 °C for 30 sec and 72 °C for 3 min 30 sec.

### cDNA sequence analysis and functional annotation

Full-length sequences of the cDNA clones were assembled for the nine copepod libraries according to Zhang *et al*.^18^ and the open reading frames (ORFs) were predicted using the getorf application in the EMBOSS package^40^; the first 5′ AUG within the frame was taken as the start codon. The distance between the 1^st^ nucleotide (nt) after CopepodSL and the predicted start codon defined the 5′-UTR, while the 1^st^ nt after the stop codon to the 1^st^ A of poly(A) tails delineated the 3′-UTR, as estimated with customized perl scripts (to be made available upon request). Codon Usage was analysed by CUSP in EMBOSS. The full-length cDNA sequences obtained were annotated using Blast2GO^41^ based on sequence similarity searches (BLASTx) against NCBI non-redundant protein (nr) database (E-value < 10^−5^). Gene ontology was assigned based on associated biological process, molecular function, and cellular components of the hit. Sequences with significant BLASTx hit (E-value <10^−14^) to the known genes were manually checked for their start codon and the lengths of 5′- and 3′-UTRs to confirm the accuracy of the ORF prediction.

### RNA blots for *A. pacifica, C. sinicus* and *P. poplesia* slRNA

Total RNA (1–2 μg) from *A. pacifica*, *C. sinicus* and *P. poplesia* was electrophoresed in an 8% acrylamide and 8 M urea gel and transferred to a nylon membrane as previously reported^18^,^42^. A copepod slRNA oligonucleotide (CopepodSLa/s, Table 1) was labeled with ^32^P and hybridized to the membrane as reported^42^. The membrane was washed with 2 × SSC at 43 °C and 50 °C, respectively and exposed for 24 h.

### Isolation of slRNA genes and determination of their 3′-end for *A. pacifica* and *P. poplesia*

It is known that slRNA gene is often arranged as tandem repeats or clustered with small RNAs such as 5S rDNA^30^. Based on the SL sequence detected in copepods and the conserved regions of 5S rDNA in insects and other organisms, appropriate primers (Table 1) were designed and PCR performed essentially following the reported methods^18^,^30^. The 3′-end of copepod slRNA transcripts were determined following Zhang *et al*.^18^. In brief, poly(A) mRNA was subtracted from the total RNA, the poly(A) tail was then artificially added to the remaining non-mRNA using Poly(A) Polymerase (Takara, Japan) and the resultant RNA was used to synthesize cDNA using modified oligo-dT primer (Invitrogen, USA). The 3′-end of the slRNA was then PCR amplified using CopepodSL5a and CopepodSL 5b pairing with Racer3.

### Modeling of RNA secondary structure

Secondary structures of slRNAs for *A. pacifica* and *P. poplesia* were predicted using MFOLD: Prediction of RNA secondary structure modeling program (http://bioweb.pasteur.fr/seqanal/interfaces/mfold-simple.html). Folding was performed at 25 °C, the water temperature of the sampling site, without any constraints.

### *In silico* SL search of copepod datasets

We queried the 6 CopepodSL sequences (CopepodSL, CopepodSL-like1a, 1b, 2a, 2b, 2c) against existing transcriptomic and genomic data sets of copepod in NCBI using BLASTn. The databases included Expressed sequence tags (ESTs), Transcriptome shotgun assembly (TSA) and Whole-genome shotgun contigs (WGS). Copepod sequences available in the databases were from the orders of Calanoida, Siphonostomatoida, Cyclopoida and Harpacticoida.

### PCR amplification and sequencing of copepod ribosomal protein cDNAs absent in the cDNA libraries

To obtain a complete set of eukaryotic cRPs sequences from copepods, we designed gene specific primers for the cRPs that were absent in the full-length cDNA library based on the conserved region of cRPs in other copepods and insects. These primers were paired with CopepodSL or Racer3 to obtain the 5′- or 3′-end of the genes. A touch-up PCR program (95 °C 20 sec, 95 °C 20 sec for 1 cycle; 52 °C 30 sec and 72 °C 1 min for 5 cycles; 95 °C 20 sec, 56 °C 30 sec and 72 °C 1 min for 30 cycles) was used in amplification; PCR products were purified and sequenced as reported^18^. The details have been presented elsewhere^36^.

## Additional Information

**How to cite this article**: Yang, F. *et al*. Spliced leader RNA *trans*-splicing discovered in copepods. *Sci. Rep.* **5**, 17411; doi: 10.1038/srep17411 (2015).

## Supplementary Material

Supplementary Information

## Figures and Tables

**Table 1:** Oligonucleotides used in the study.

Primer Name	Sequence (5′–3′)	Application	Reference
CopepodSL	GTCTAAWACGACCAAGCTWAWWTTGCTTGATTCAC TTCWWYAAGAG	PCR of full-length cDNA	This study
Racer3	TGTCAACGATACGCTACGTAACG	PCR of full-length cDNA	GeneRacer kit
CopepodSL-like1a	CCAAGTAAATAATACGTGTCTCTGACAAAAATCAAG	PCR of full-length cDNA	Douris *et al*.^20^
CopepodSL-like1b	CCAAGTAAATAATACGTGTCTCTGACTAATAATCAAG	PCR of full-length cDNA	Douris *et al*.^20^
CopepodSL-like2a	CCAAGCTACACTGCTTGAGTATAACACTTTAAAAG	PCR of full-length cDNA	Douris *et al*.^20^
CopepodSL-like2b	CCAAGCTATACTGCTTGTCTAAACACTTTAA AAG	PCR of full-length cDNA	Douris *et al*.^20^
CopepodSL-like2c	ATGCTATACTGCTTGTTTAACACTTTAAAAG	PCR of full-length cDNA	Douris *et al*.^20^
CopepodSL5b	agagGTCTAAWACGACCAAGCT*	PCR of slRNA gene	This study
CopepodSL5c	CCAAGCTWAWWTTGCTTGATTCACTTC	PCR of slRNA gene	This study
CopepodSL-RcA	CTCTTAATGAAGTGAATCAAGCA	PCR of slRNA gene	This study
CopepodSL-RcB	CTCTTGTAGAAGTGAATCAAGCA	PCR of slRNA gene	This study
5ScomF1	ACCCRGTCTCGTCMGATCMNSGAAGTYA	PCR of 5S rDNA-slRNA gene cluster	This study
5ScomF2	TACTTGGATGGGTGACCGCC	PCR of 5S rDNA-slRNA gene cluster	This study
5ScomR	GGCGGTCACCCATCCAAGTA	PCR of 5S rDNA-slRNA gene cluster	This study
CopepodSLa/s	AGCTTGGTCGTWTTAGAC	Northern blot analysis	This study
