# Supplementary material for: Spliced leader RNA trans-splicing discovered in copepods
Source: Sci Rep. 2015 Dec 1;5:17411. doi: 10.1038/srep17411 (PMC4664967; doi:10.1038/srep17411)
Supplement: Supplementary Information [file srep17411-s1.pdf]

Spliced leader RNA *trans*-splicing discovered in copepods

Feifei Yang, Donghui Xu, Yunyun Zhuang, Xiaoyan Yi, Yousong Huang, Hongju Chen, Senjie Lin, David A. Campbell, Nancy R. Sturm, Guangxing Liu, & Huan Zhang

Supplementary Information

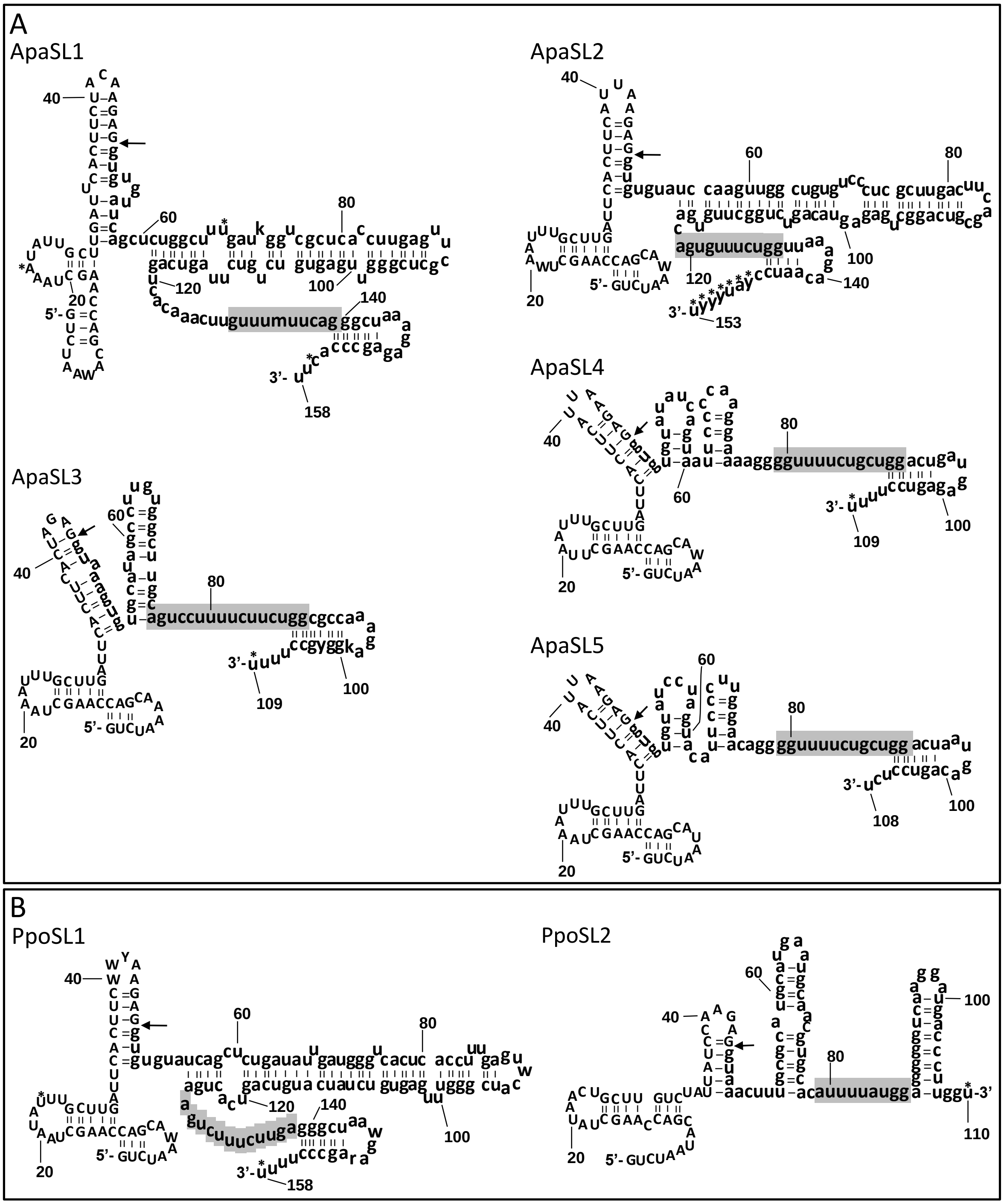

Fig. S1. Secondary structure of the major type Copepod SL RNA. Predicted structures of SL RNA for (A) *Acartia pacifica* (ApaSL), and (B) *Pseudodiaptomus poplesia* (PpoSL) based on the most abundant cDNAs obtained. Exons are shown in uppercase, and introns are shown in lowercase; arrows indicate the exon–intron boundaries. Potential Sm binding sites are shaded.

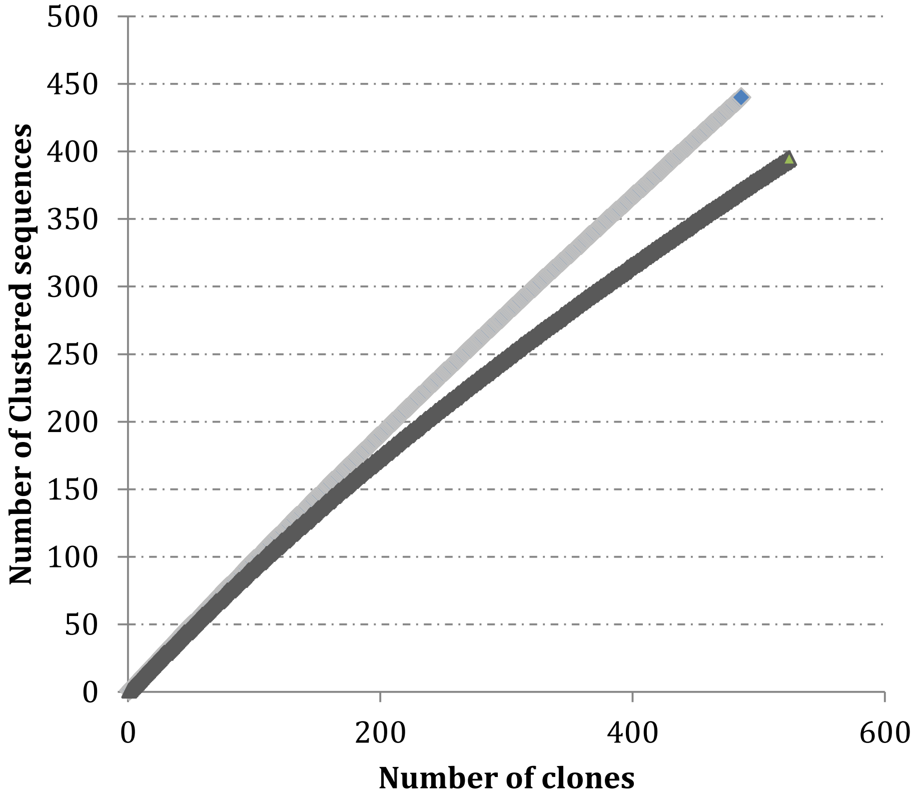

Fig. S2. Rarefaction curves of the cDNA sequences of *Acartia pacifica* (486 cDNAs) and *Pseudodiaptomus poplesia* (524 cDNAs).

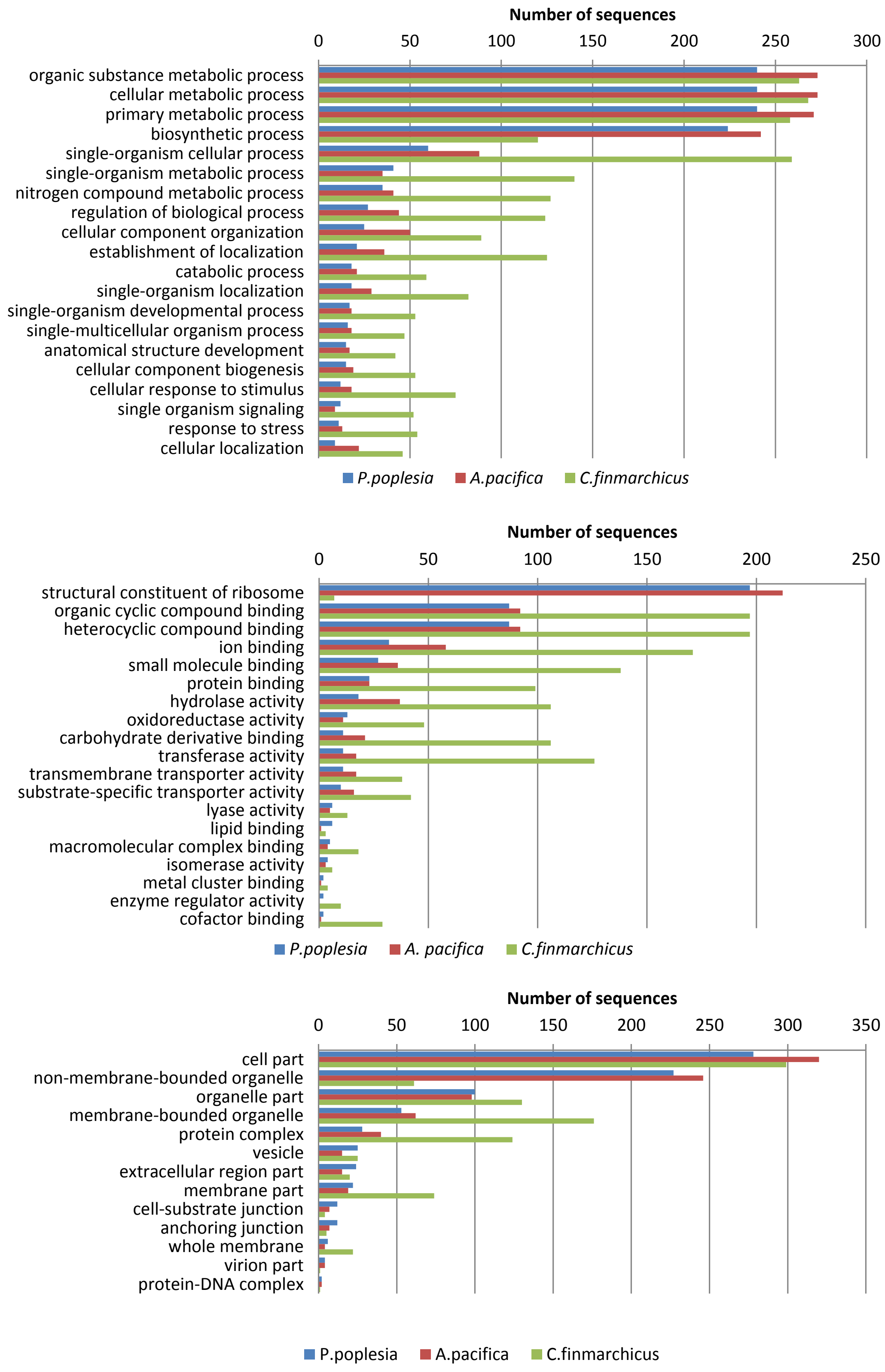

Fig. S3. GO category assignments for *Acartia pacifica*, *Pseudodiaptomus poplesia* and *Calanus finmarchicus* cDNAs. A) biological process; B) molecular function; C) cellular components.

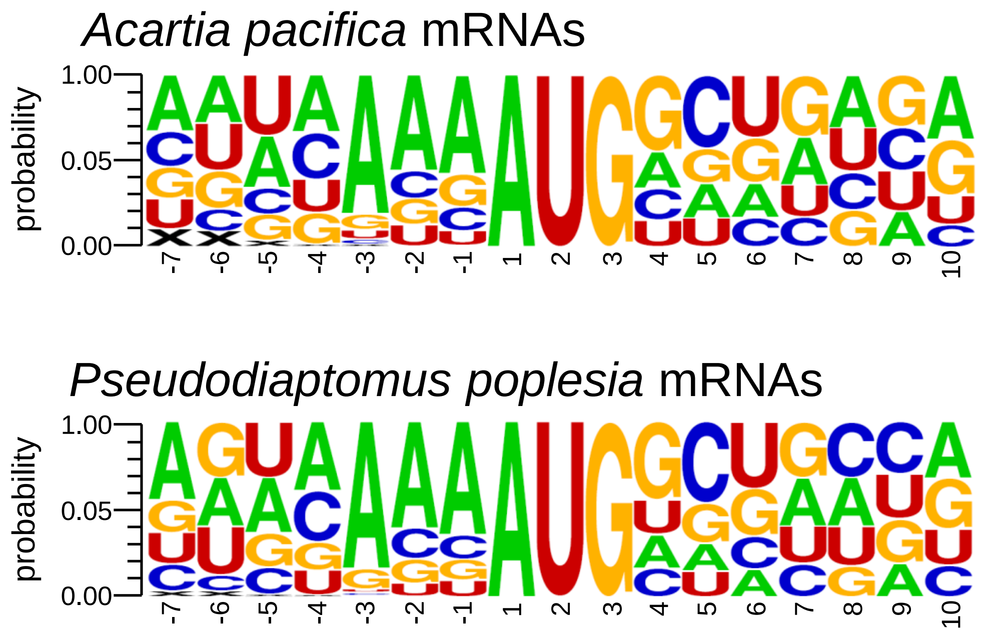

Fig. S4. Probability of nucleotides flanking the start codon AUG in copepod mRNAs.

Table S1. SL RNA transcripts of *Acartia pacifica* (ApaSLRNA) and *Pseudodiaptomus poplesia* (PpoSLRNA)
>ApaSLRNA1-1
GUCUAAUACGACCAAGCUAAUAUUGCUUGAUUCACUUCUACAAGAGguguguaucagcucuggcuuugauuggu
cgcucaccuugaguucgcucggguugagugucugucuuagucagucacaaaccuguuucuucagggcuaaagag
agcccauu
>ApaSLRNA1-2
GUCUAAAACGACCAAGCUAAUAUUGCUUGAUUCACUUCUACAAGAGguguguaucagcucuggcuuugauuggu
cgcucaccuugaguucgcucggguugagugucugucuuagucagucacaaaccuguuucuucagggcuaaagag
agcccauu
>ApaSLRNA1-3
GUCUAAUACGACCAAGCUAAUAUUGCUUGAUUCACUUCUACAAGAGguguguaucagcucuggcuuugauuggu
cgcucaccuugaguucgcucggguugagugucugucuuagucagucacaaaccuguuuauucagggcuaaagag
agcccacu
>ApaSLRNA1-4
GUCUAAUACGACCAAGCUAAUAUUGCUUGAUUCACUUCUACAAGAGguguguaucagcucuggcuuugauuggu
cgcucaucuugaguucgcucggguugagugucugucuuagucagucacaaaccuguuucuucagggcuaaagag
agccc
>ApaSLRNA1-5
GUCUAAUACGACCAAGCUAAUAUUGCUUGAUUCACUUCUAAAAGAGguguguaucagcaacagcuguggcaggu
cgcucaccuugaguuagcucggguugagugucugccuuaguuguaugcaauccuauuucuacagggcugaugag
agccc
>ApaSLRNA1-6
GUCUAAUACGACCAAGCUAAUAUUGCUUGAUUCACUUCUACAAGAGguguguaucagcucuggcuugaugggguc
gcucaccgugaguucgcucggguugagugucugucuuagucagucacaaacuuguuuauucagggcuaaagaga
gcccacu
>ApaSLRNA1-7
GUCUAAUACGACCAAGCUAAUAUUGCUUGAAUCACUUCUACAAGAGguguguaucagcucuggcuugaugggguc
gcucaccuugaguucgcucggguugagugucugucuuagucagucacaaacuuguuuauucagggcuaaagaga
gcccacuu
>ApaSLRNA1-8
GUCUAAUACGACCAAGCUAAUAUUGCUUGAUUCACUUCUACAAGAGguguguaucagcucuggcuugaugggguc
gcucaccuugaguucgcucggguugagugucugucuuggucagucacaaacuuguuuauucagggcuaaagaga
gcccacu
>ApaSLRNA1-9
GUCUAAUACGACCAAGCUAAAUAUUGCUUGAUUCACUUCUACAAGAGguguguaucagcucuagcuuuggugag
ucgcucaccuugaguucgcucggguugagugucugccucagucagaugcgaacuuguuuuuacagggcugaugu
aagccc
>ApaSLRNA1-10
GUCUAAUACGACCAAGCUAAAUAUUGCUUGAUUCACUUCUACAAGAGguguguaucagcucuagcuuuggugggg
ucgcucaccuugaguucgcucggguugagugucugccucagucagaugcaaacuuguuuuuacagggcugauga
aagccc
>ApaSLRNA1-11
GUCUAAUACGACCAAGCUAAUAUUGCUUGAUUCACUUCUACAAGAGguguguaucagcucaggcuuugauuggu
cgcucaccuugaguuugcucggguugagugucugucuuagucagucacuuuuu
>ApaSLRNA6-1
GUCUAAUACGACCAAGCUAAUAUUGCUUGAUUCACUUCUACAAGAGguguguaucagcucuggcuuugauuggu
cgcucgcccaggagaaaucccaggcugagugucugucuuagucagucacaaacuuguuuauucagggcuaaaga
gagcccaucu
>ApaSLRNA6-2
GUCUAAUACGACCAAGCUAAUAUUGCUUGAUUCACUUCUACAAGAGguguguaucagcucuggcuuugauuggu
cgcucgcccaggagaaaucccaggcugagugucugucuuagucagucacaaacuuguuuauucagggcuaaaga
gagcccaucu
>ApaSLRNA6-3

GUCUAAUACGACCAAGCUAAUAUUGCUUGAUUCACUUCUACAAGAGguguguaucagcucuggcuuugauuggu
cgcucgcccaggagaaaucccaggcugagugucugucuuagucagucacaaacuuguuuauucagggcuaaaga
gagccc
>ApaSLRNA3-1
GUCUAAUACGACCAAGCUAAAUUUGCUUGAUUCACUUCACUAGAGguaaagugugcauagccuuguggcuugca
guccuuuucuucuggcgccaaagagggugccuuu
>ApaSLRNA3-2
GUCUAAAACGACCAAGCUAAAUUUGCUUGAUUCACUUCACUAGAGguaaagugugcauagccuuguggcuugca
guccuuuucuucuggcgccaaagauggcgccuuuu
>ApaSLRNA3-3
GUCUAAUACGACCAAGCUAAUACUUGCUUGAUUCACUUCACUAGAGguaaaguguuugaagccuuuuggcuucc
aguccuguucuucuggcaccaaagagggugccuuu
>PpoSLRNA2-1
GUCUAAUACGACCAAGCUAUAACUGCUUGUCUAUUAUCCAAGAGguaaacuuugcgcaugcaugaaugcaacgu
gcacauuuuauggaggggucaaggaugacccuuggu
>PpoSLRNA2-2
GUCUAAUACGACCAAGCUAUAACUGCUUGUCUAUUAUCCAAGAGguaaacuuugcgcaugcaugaaugcaacgu
gcacauuuuauggaggggucaaggaugacccuuggu
>PpoSLRNA2-3
GUCUAAAACGACCAAGCUAUAACUGCUUGUCUAUUAUCCAAGAGguaaacuuugcgcaugcaugaaugcaacgu
gcacauuuuauggaggggucaaggaugacccuugg
>PpoSLRNA2-4
GUCUAAAACGACCAAGCUAUAACUGCUUGUCUAUUAUCCAAGAGguaaacuuugcguaugcaugaaugcaacgu
gcacauuuuauggaggggucaaggaugacccuuggu
>PpoSLRNA2-5
GUCUAAUACGACCAAGCUAUAACUGCUUGUCUAUUAUCCAAGAGguaaacuuugcgcaugcaugaaugcaacgu
gcacauuuuauggaggggucaaggaugacccuuggu
>PpoSLRNA3-1
GUCUAAUACGACCAAGCUAUAACUGCUUGUCUAUUAUCCAAGAGguaaacuuugcgcaugaauccaacgugcac
auuuuauggaggggucaaggaugacccuugu
>PpoSLRNA3-2
GUCUAAUACGACCAAGCUAUAACUGCUUGUCUAUUAUCCAAGAGguaaacuuugcgcaugaauccaacgugcac
auuuuauggaggggucaaggaugacccuugu
>PpoSLRNA4-1
GUCUAAUACGACCAAGCUAAUAUUUGCUUGUUUCUUACACUUGAGguaugauccgaucuuaaagccguuggauc
uuuucuugaaggguuaaugaugaccuuau
>ApaSLRNA4-1
GUCUAAUACGACCAAGCUUAAUUUGCUUGAUUCACUUCAUUAAGAGguguguauaucaguaauuccccaaggaa
aaggguuuucugcuggacugaugagaguccuuu
>ApaSLRNA4-2
GUCUAAUACGACCAAGCUUAAUUUGCUUGAUUCACUUCAUUAAGAGguguguauaucaguaauuccccaaggaa
aaggguuuucugcuggacugaugagaguccuuuu
>ApaSLRNA4-3
GUCUAAAACGACCAAGCUUAAUUUGCUUGAUUCACUUCAUUAAGAGguguguauaucaguaauuccccaaggaa
aaggguuuucugcuggacugaugagaguccuuuu
>ApaSLRNA4-4
GUCUAAUACGACCAAGCUUAAUUUGCUUGAUUCACUUCAUUAAGAGguguguauaucaguaauuccccaaggaa
aaggguuuucugcuggacugaugagaguccuuu
>ApaSLRNA5-1
GUCUAAUACGACCAAGCUAAGUUUGCUUGAUUCACUUCAUUAAGAGguguguauccuaguacauucccuuggaa
caggguuuucugcuggacuaaugacaguccucu
>PpoSLRNA1-1

GUCUAAAACGACCAAGCUAAUAUUGCUUGAUUCACUUCUACAAGAGguguguaucagcucugauauugaugggu
cacucaccuuugaguucaucucggguuugagugucuaucaugucagucacugaagucuuucuugagggcuaaag
agagcccuuuu
>PpoSLRNA1-2
GUCUAAAACGACCAAGCUAAUAUUGCUUGAUUCACUUCUACAAGAGguguguaucagcucugauauugaugggu
cacucaccuuugaguucaucucggguuugagugucuaucaugucagucacugaagucuuucuugagggcuaaag
agagcccuuuu
>PpoSLRNA1-3
GUCUAAUACGACCAAGCUAAUAUUGCUUGAUUCACUUCUACAAGAGguguguaucagcucugauauugaugggu
cacucaccuuugaguucaucucggguuugagugucuaucaugucagucacugaagucuuucuugagggcuaaag
agagcccuuu
>PpoSLRNA1-4
GUCUAAUACGACCAAGCUAAUAUUGCUUGAUUCACUUCUACAAGAGguguguaucagcucugauauugaugggu
cacucaccuuugaguucaucucggguuugagugucuaucaugucagucacugaagucuuucuugagggcuaaag
agagcccuuu
>ApaSLRNA2-1
GUCUAAUACGACCAAGCUAAAUUUGCUUGAUACACUUCAUUAAGAGguguguauccaaguuggcugugucccuc
gcuugacuucagcgucaggcugagaguacagucuagcuugugacaguguuucugcuggauuaaagacaauccua
uc
>ApaSLRNA2-2
GUCUAAUACGACCAAGCUAAAUUUGCUUGAUUCACUUCAUUAAGAGguguguauccaaguuggcugugucccuc
gcuugacuucagcgucaggcugagaguacagucuagcuugugacaguguuucugcuggauuaaagacaauccua
uc
>ApaSLRNA2-3
GUCUAAAACGACCAAGCUAAAUUUGCUUGAUUCACUUCAUUAAGAGguguguauccaaguugucugugucccuc
gcuugacuucagcgucaggcugagaguacagucuagcuugugacaguguuucugcuggauuaaagacaauccua
ucccu
>ApaSLRNA2-4
GUCUAAUACGACCAAGCUAAAUUUGCUUGAUUCACUUCAUUAAGAGguguguauccaaguuagcugugucccuc
gcuugacuucagcgucaggcugagaguacagucuagcuugugacagugucucugcuggauuaaagacaauccua
ucuuu
>ApaSLRNA2-5
GUCUAAUACGACCAAGCUAAAUUUGCUUGAUUCACUUCAUUAAGAGguguguauccaaguuagcugugccccuc
gcuugacuucagcgucaggcugagaguacagucuagcuugugacaguguuucugcuggauuaaagacaauccua
ucc
>ApaSLRNA2-6
GUCUAAUACGACCAAGCUAAAUUUGCUUGAUUCACUUCAUUAAGAGguguguauccaaguuggcugugucccuc
gcuugacuucagcgucaggcugagaguacagucuagcuugugacaguguuucugcuggauuaaagacaauccua
uuu
>ApaSLRNA2-7
GUCUAAUACGACCAAGCUAAAUUUGCUUGAUUCACUUCAUUAAGAGguguguauccaaguuagcugugucccuc
gcuugacuucagcgucaggcugagaguacagucuagcuugugacaguguuucugcuggauuaaagacaauccua
uc
>ApaSLRNA2-8
GUCUAAUACGACCAAGCUAAAUUUGCUUGAUUCACUUCAUUAAGAGguguguauccaaguuagcugugucccuc
gcuugacuucagcgucaggcugagaguacagucuagcuugugacaguguuucugcuggauuaaagacaauccua
uc
>ApaSLRNA2-9
GUCUAAUACGACCAAGCUAAAUUUGCUUGAUUCACUUCAUUAAGAGguguguauccaaguuagcugugucccuc
gcuugacuucagcgucaggcugagaauacagucuagcuugugacaguguuucugcuggauuaaagacaauccua
u
>ApaSLRNA2-10
GUCUAAAACGACCAAGCUAAAUUUGCUUGAUUCACUUCAUUAAGAGguguguauccaaguuagcugugucccuc
gcuugacuucagcgucaggcugagaguacagucuagcuugugacaguguuucugcuggauuaaagacaauccu

>ApaSLRNA2-11
GUCUAAAACGACCAAGCUAAAUUUGCUUGAUUCACUUCAUUAAGAGguguguauccaaguuagcugugucccuc
gcuugacuucagcgucaggcugagaguacagucuagcuugugacaguguuucugcuggauuaaagacaauccu
>ApaSLRNA2-12
GUCUAAUACGACCAAGCUAAAUUUGCUUGAUUCACUUCAUUAAGAGguguguauccaaguuagcugugucccuc
gcuugacuucagcgucaggcugagaguacagucuagcuugugacaguguuucugcuggauuaaagacaauccu
>ApaSLRNA2-13
GUCUAAUACGACCAAGCUAAAUUUGCUUGAUUCACUUCAUCAAGAGguguguauccaaguuagcugugucccuc
gcuugacuucagcgucaggcugagaguacagucuagcuugugacaguguuucugcuggauuaaagacaauccu
>ApaSLRNA2-14
GUCUAAUACGACCAAGCUAAAUUUGCUUGAUUCACUUCAUUAAGAGguguguauccaaguuagcugugucccuc
gcuugacuucagcgucaggcugagaguacagucuagcuugugacaguguuucugcuggauuaaagacaauccu
>ApaSLRNA2-15
GUCUAAUACGACCAAGCUAAAUUUGCUUGAUUCACUUCAUUAAGAGguguguauccaaguuagcugugucccuc
gcuugacuucagcgucaggcugagaguacagucuagcuugcgacaguguuucugcuggauuaaagacaauccu
>ApaSLRNA2-16
GUCUAAUACGACCAAGCUAAAUUUGCUUGAUUCACUUCAUUAAGAGguguguauccaaguuagcugugucccuc
gcuugacuucagcgucaggcugagaguacagucuagcuugugacaguguuucugcuggauuaaagacaauccu
>ApaSLRNA2-17
GUCUAAUACGACCAAGCUAAUAUUGCUUGAUUCACUUCAUUAAGAGguguguauccaaguuagcugugucccuc
gcuugacuucagcgucaggcugagaguacagucuagcuugugacaguguuucugcuggauuaaagacaauccu
>ApaSLRNA2-18
GUCUAAUACGACCAAGCUAAAUUUGCUUGAUUCACUUCAUUAAGAGguguguauccaaguuagcugugucccuc
gcuugacuucagcgucaggcugagaguacagucuagcuugugacaguguuucugcuggauuaaagacaauccu
>ApaSLRNA2-19
GUCUAAUACGACCAAGCUAAAUUUGCUUGAUUCACUUCAUUAAGAGguguguauccaaguuagcugugucccuc
gcuugacuucagcgucaggcugagaguacagucuagcuugugacaguguuucugcuggauuaaagacaauccc
>ApaSLRNA2-20
GUCUAAUACGACCAAGCUAAAUUUGCUUGAUUCACUUCAUUAAGAGguguguauccaaguuagcugugucccuc
gcuugacuucagcgucaggcugagaguacagucuagcuugugacaguguuucugcuggauuaaagacaauccc
>ApaSLRNA2-21
GUCUAAUACGACCAAGCUAAAUUUGCUUGAUUCACUUCAUUAAGAGguguguauccaaguuagcugugucccuc
gcuugacuucagcgucaggcugagaguacagucuagcuugugacaguguuucugcuggauuaaagacaauccc
>ApaSLRNA2-22
GUCUAAUACGACCAAGCUAAAUUUGCUUGAUUCACUUCAUUAAGAGguguguauccaaguuagcugugucccuc
gcuugacuucagcgucaggcugagaguacaguccagcucgugacaguguuucugcuggauuaaagacaauccc
>ApaSLRNA2-23
GUCUAAUACGACCAAGCUAAAUUUGCUUGAUUCACUUCAUUAAGAGguguguauccaaguuagcugugucccuc
gcuugacuucagcgucaggcugagaguacagucuagcuugugacaguguuucugcuggauuaaagacaaucc
>ApaSLRNA2-24
GUCUAAUACGACCAAGCUAAAUUUGCUUGAUUCACUUCAUUAAGAGguguguauccaaguuggcugugucccuc
gcuugacuucagcgucaggcugagaguacagucuagcuugugacaguguuucugcuggauuaaagacaauccu
>ApaSLRNA2-25
GUCUAAUACGACCAAGCUAAAUCUGCUUGAUUCACUUCAUUAAGAGguguguauccaaguuggcugugucccuc
gcuugacuucagcgucaggcugagaguacagucuagcuugugacaguguuucugcuggauuaaagacaauccu
>ApaSLRNA2-26
GUCUAAUACGACCAAGCUAAAUUUGCUAGAUUCACUUCAUUAAGAGguguguauccaaguuggcugugucccuc
gcuugacuucagcgucaggcugagaguacagucuggcuugugacaguguuucugcuggauuaaagacaauccu
>ApaSLRNA2-27
GUCUAAAACGACCAAGCUAAAUUUGCUUGAUUCACUUCAUUAAGAGguguguauccaaguuggcugugucccuc
gcuugacuucagcgucaggcugagaguacagucuagcuugugacaguguuucugcuggauuaaagacaaucc
>ApaSLRNA2-28
GUCUAAAACGACCAAGCUAAAUUCGCUUGAUUCACUUCAUUAAGAGguguguauccaaguuagcugugucccuc
gcuugacuucagcgucaggcugagaguacagucuagcuugugacaguguuucugcuggauuaaagacaaucc

```
>ApaSLRNA2-29
GUCUAAUACGACCAAGCUAAAUUUGCUUGAUUCACUUCAUUAAGAGguguguauccaaguuggcugugucccuc
gcuugacuucagcgucaggcugagaguacagucuagcuugugacaguguuucugcuggauuaaagacaaucc
>ApaSLRNA2-30
GUCUAAUACGACCAAGCUAAAUUUGCUUGAUUCACUUCAUUAAGAGguguguauccaaguuggcugugucccuc
gcuugacuucagcgucaggcugagaguacagucuagcuugugacaguguuucugcuggauuaaagacaaucc
>ApaSLRNA2-31
GUCUAAUACGACCAAGCUAAAUUUGCUUGAUUCACUUCAUUAAGAGguguguauccaaguuggcugugucccuc
gcuugacuucagcgucaggcugagaguacagucuagcuugugacaguguuucugcuggauuaaagacaauccu
>ApaSLRNA7-1
GUCUAAUACGACCAAGCUAAUAUUGCUUGAUUCACUUCUACAAGAGguguguaucagcucuggcuuugauuggu
cgcucaccuugaguucgcucggguugagugucugucuuagucagucacaaaccuguuucuucagggcuaaagag
agcccauu
```

Table S2. Top hits of the 1288 cDNAs obtained from 9 copepods using CopepodSL, including *Acartia pacifica*, *Calanus sinicus*, *Centropages dorsispinatus*, *Centropages tenuiremis*, *Labidocera rotunda* (previously *L. bipinnata*), *Paracalanus parvus*, *Pseudodiaptomus poplesia*, *Tortanus dextrilobatus* and *Tortanus forcipatus.*

**Sequence ID**	**Species**	**Definition**	**Hit species**	**Hit Accession No.**	**Evalue**
Apa_cDNA1	*Acartia pacifica*	lethal 35Di	*Microplitis demolitor*	XP_008547855	1.91E-22
Apa_cDNA10	*A. pacifica*	U2 small nuclear ribonucleoprotein A	*Caligus clemensi*	ACO15752	1.78E-95
Apa_cDNA100-1	*A. pacifica*	beta-Ig-H3/fasciclin	*Deinococcus apachensis*	WP_019585125	1.98E-31
Apa_cDNA100-2	*A. pacifica*	beta-Ig-H3/fasciclin	*Deinococcus apachensis*	WP_019585125	1.98E-31
Apa_cDNA101	*A. pacifica*	60S ribosomal protein L8	*Acartia pacifica*	AGN29659	8.20E-170
Apa_cDNA102	*A. pacifica*	tryptophan-rich protein	*Salmo salar*	ACI69574	1.82E-22
Apa_cDNA103	*A. pacifica*	NADH dehydrogenase	*Drosophila virilis*	XP_002046735	6.57E-28
Apa_cDNA104-1	*A. pacifica*	28S ribosomal protein S17, mitochondrial	*Lepeophtheirus salmonis*	ACO12803	5.97E-46
Apa_cDNA104-2	*A. pacifica*	28S ribosomal protein S17, mitochondrial	*Lepeophtheirus salmonis*	ACO12803	1.83E-45
Apa_cDNA105	*A. pacifica*	NADH dehydrogenase	*Anopheles darlingi*	ETN62135	8.65E-32
Apa_cDNA106	*A. pacifica*	methyltransferase-like protein 4-like	*Tribolium castaneum*	XP_008195262	9.07E-46
Apa_cDNA107	*A. pacifica*	ADP-ribosylation factor 1	*Lepeophtheirus salmonis*	ACO12345	1.38E-121
Apa_cDNA108	*A. pacifica*	dihydrofolate reductase	*Acromyrmex echinatior*	EGI57779	1.01E-57
Apa_cDNA109	*A. pacifica*	peptide deformylase, mitochondrial	*Lepeophtheirus salmonis*	ADD38403	3.65E-52
Apa_cDNA110-1	*A. pacifica*	60S ribosomal protein L35a	*Lepeophtheirus salmonis*	ACO12970	1.93E-51
Apa_cDNA110-2	*A. pacifica*	60S ribosomal protein L35a	*Lepeophtheirus salmonis*	ACO12970	1.34E-52
Apa_cDNA110-3	*A. pacifica*	60S ribosomal protein L35a	*Lepeophtheirus salmonis*	ACO12970	3.27E-52
Apa_cDNA111	*A. pacifica*	Activated RNA polymerase II transcriptional coactivator p15	*Caligus clemensi*	ACO15626	5.53E-31
Apa_cDNA11-1	*A. pacifica*	60S acidic ribosomal protein P1	*Ixodes scapularis*	XP_002435967	3.32E-19
Apa_cDNA11-2	*A. pacifica*	60S acidic ribosomal protein P1	*Ixodes scapularis*	XP_002435967	6.66E-18
Apa_cDNA112-1	*A. pacifica*	60S ribosomal protein L27	*Caligus rogercresseyi*	ACO11360	2.75E-67
Apa_cDNA112-2	*A. pacifica*	60S ribosomal protein L27	*Caligus rogercresseyi*	ACO11360	2.85E-67
Apa_cDNA112-3	*A. pacifica*	60S ribosomal protein L27	*Caligus rogercresseyi*	ACO11360	2.75E-67
Apa_cDNA112-4	*A. pacifica*	60S ribosomal protein L27	*Caligus rogercresseyi*	ACO11360	4.11E-67
Apa_cDNA112-5	*A. pacifica*	60S ribosomal protein L27	*Caligus rogercresseyi*	ACO11360	3.18E-67
Apa_cDNA112-6	*A. pacifica*	60S ribosomal protein L27	*Caligus rogercresseyi*	ACO11360	1.27E-66
Apa_cDNA113	*A. pacifica*	40S ribosomal protein S4	*Lepeophtheirus salmonis*	ACO12708	1.79E-140
Apa_cDNA11-3	*A. pacifica*	unknown protein			
Apa_cDNA114	*A. pacifica*	endoplasmic reticulum protein	*Oncorhynchus mykiss*	CDR02401	1.71E-18
Apa_cDNA11-4	*A. pacifica*	60S acidic ribosomal protein P1	*Ixodes scapularis*	XP_002435967	6.66E-18
Apa_cDNA115	*A. pacifica*	40S ribosomal protein S10	*Acartia pacifica*	AGN29661	3.55E-91
Apa_cDNA11-5	*A. pacifica*	60S acidic ribosomal protein P1	*Ixodes scapularis*	XP_002435967	5.05E-19
Apa_cDNA11-6	*A. pacifica*	60S acidic ribosomal protein P1	*Ixodes scapularis*	XP_002435967	3.21E-19
Apa_cDNA116-1	*A. pacifica*	ubiquitin/ribosomal protein L40 fusion protein	*Artemia franciscana*	ABS19964	1.04E-83
Apa_cDNA116-2	*A. pacifica*	ubiquitin/ribosomal protein L40 fusion protein	*Artemia franciscana*	ABS19964	5.07E-84

Apa_cDNA116-3	*A. pacifica*	ubiquitin/ribosomal protein L40 fusion protein	*Artemia franciscana*	ABS19964	5.07E-84
Apa_cDNA117	*A. pacifica*	lysM and putative peptidoglycan-binding domain-containing protein 3	*Zootermopsis nevadensis*	KDR09639	2.26E-10
Apa_cDNA118	*A. pacifica*	mitochondrial porin	*Drosophila willistoni*	XP_002064648	6.18E-99
Apa_cDNA119-1	*A. pacifica*	mitotic-spindle organizing protein 1	*Chrysemys picta bellii*	XP_005306889	3.48E-20
Apa_cDNA119-2	*A. pacifica*	mitotic-spindle organizing protein 1	*Chrysemys picta bellii*	XP_005306889	3.48E-20
Apa_cDNA12	*A. pacifica*	40S ribosomal protein S2	*Acartia pacifica*	AGN29613	1.62E-153
Apa_cDNA120	*A. pacifica*	mitochondrial tRNA-specific 2-thiouridylase 1	*Xenopus (Silurana) tropicalis*	XP_002932238	7.15E-126
Apa_cDNA121	*A. pacifica*	LYR motif-containing protein 1	*Caligus rogercresseyi*	ACO10582	8.49E-30
Apa_cDNA122	*A. pacifica*	nucleoredoxin-like protein 2	*Hydra vulgaris*	XP_002165519	2.35E-55
Apa_cDNA123-1	*A. pacifica*	Cellular retinoic acid-binding protein 2	*Caligus rogercresseyi*	ACO11291	2.10E-41
Apa_cDNA123-2	*A. pacifica*	Cellular retinoic acid-binding protein 2	*Caligus rogercresseyi*	ACO11291	1.01E-42
Apa_cDNA123-3	*A. pacifica*	Cellular retinoic acid-binding protein 2	*Caligus rogercresseyi*	ACO11291	6.17E-42
Apa_cDNA123-4	*A. pacifica*	Cellular retinoic acid-binding protein 2	*Caligus rogercresseyi*	ACO11291	1.48E-42
Apa_cDNA124-1	*A. pacifica*	40S ribosomal protein S28	*Megachile rotundata*	XP_003700807	3.65E-22
Apa_cDNA124-2	*A. pacifica*	40S ribosomal protein S28	*Megachile rotundata*	XP_003700807	1.60E-23
Apa_cDNA124-3	*A. pacifica*	40S ribosomal protein S28	*Culex quinquefasciatus*	XP_001865978	3.31E-22
Apa_cDNA125-1	*A. pacifica*	40S ribosomal protein S7	*Acartia pacifica*	AGN29653	2.56E-134
Apa_cDNA125-2	*A. pacifica*	40S ribosomal protein S7	*Acartia pacifica*	AGN29653	3.94E-134
Apa_cDNA126-1	*A. pacifica*	60S ribosomal protein L14	*Spodoptera frugiperda*	AAK92157	9.97E-45
Apa_cDNA126-2	*A. pacifica*	60S ribosomal protein L14	*Spodoptera frugiperda*	AAK92157	5.59E-45
Apa_cDNA126-3	*A. pacifica*	60S ribosomal protein L14	*Spodoptera frugiperda*	AAK92157	1.10E-44
Apa_cDNA126-4	*A. pacifica*	60S ribosomal protein L14	*Spodoptera frugiperda*	AAK92157	7.89E-45
Apa_cDNA126-5	*A. pacifica*	60S ribosomal protein L14	*Spodoptera frugiperda*	AAK92157	7.89E-45
Apa_cDNA127	*A. pacifica*	39S ribosomal protein L14, mitochondrial	*Anopheles sinensis*	KFB37519	9.53E-35
Apa_cDNA128	*A. pacifica*	short-chain dehydrogenase/reductase	*Megachile rotundata*	XP_003707776	1.97E-51
Apa_cDNA129	*A. pacifica*	translocon-associated protein subunit gamma	*Bombyx mori*	NP_001040330	1.22E-74
Apa_cDNA13	*A. pacifica*	40S ribosomal protein S4	*Lepeophtheirus salmonis*	ACO12708	4.08E-140
Apa_cDNA130	*A. pacifica*	phenylalanyl-tRNA synthetase beta chain	*Drosophila mojavensis*	XP_001998827	0
Apa_cDNA131	*A. pacifica*	proton-coupled folate transporter-like	*Daphnia pulex*	EFX69296	8.56E-44
Apa_cDNA132-1	*A. pacifica*	Phosphate carrier protein, mitochondrial precursor	*Lepeophtheirus salmonis*	ACO12540	0
Apa_cDNA132-2	*A. pacifica*	Phosphate carrier protein, mitochondrial precursor	*Lepeophtheirus salmonis*	ACO12540	0
Apa_cDNA133	*A. pacifica*	histone	*Drosophila erecta*	XP_001981633	3.04E-75
Apa_cDNA134	*A. pacifica*	histone H3	*Amborella trichopoda*	XP_006851804	4.67E-39
Apa_cDNA135-1	*A. pacifica*	60S ribosomal protein L21	*Hydra vulgaris*	XP_002166786	1.43E-65
Apa_cDNA135-2	*A. pacifica*	60S ribosomal protein L21	*Hydra vulgaris*	XP_002166786	1.94E-65
Apa_cDNA135-3	*A. pacifica*	60S ribosomal protein L21	*Hydra vulgaris*	XP_002166786	1.43E-65
Apa_cDNA135-4	*A. pacifica*	60S ribosomal protein L21	*Hydra vulgaris*	XP_002166786	1.04E-64
Apa_cDNA136-1	*A. pacifica*	40S ribosomal protein S16	*Ixodes scapularis*	AAY66897	1.54E-80
Apa_cDNA136-2	*A. pacifica*	40S ribosomal protein S16	*Ixodes scapularis*	AAY66897	3.44E-80
Apa_cDNA137-1	*A. pacifica*	28S ribosomal protein S16, mitochondrial	*Lepeophtheirus salmonis*	ADD38828	4.50E-37
Apa_cDNA137-2	*A. pacifica*	28S ribosomal protein S16, mitochondrial	*Lepeophtheirus salmonis*	ADD38828	4.22E-37

Apa_cDNA138	*A. pacifica*	U6 snRNA-associated Sm-like protein LSm3-like	*Oryzias latipes*	XP_004068948	4.82E-39
Apa_cDNA139	*A. pacifica*	pten-like isoform A	*Drosophila ananassae*	XP_001955396	4.28E-77
Apa_cDNA140-1	*A. pacifica*	Translation initiation factor eIF-2B subunit alpha	*Zootermopsis nevadensis*	KDR10861	4.31E-86
Apa_cDNA140-2	*A. pacifica*	Translation initiation factor eIF-2B subunit alpha	*Zootermopsis nevadensis*	KDR10861	1.66E-85
Apa_cDNA141	*A. pacifica*	PREDICTED: selenoprotein M-like	*Bombus terrestris*	XP_003394206	3.21E-18
Apa_cDNA14-1	*A. pacifica*	60S ribosomal protein L12	*Piriformospora indica DSM 11827*	CCA69560	6.54E-79
Apa_cDNA142	*A. pacifica*	60S ribosomal protein L11	*Coptotermes formosanus*	AGM32143	4.83E-110
Apa_cDNA14-2	*A. pacifica*	60S ribosomal protein L12	*Piriformospora indica DSM 11827*	CCA69560	6.54E-79
Apa_cDNA143-1	*A. pacifica*	40S ribosomal protein S19	*Lineus viridis*	ABZ04268	9.15E-65
Apa_cDNA143-2	*A. pacifica*	40S ribosomal protein S19	*Lineus viridis*	ABZ04268	9.15E-65
Apa_cDNA143-3	*A. pacifica*	40S ribosomal protein S19	*Lineus viridis*	ABZ04268	6.52E-66
Apa_cDNA143-4	*A. pacifica*	40S ribosomal protein S19	*Lineus viridis*	ABZ04268	8.10E-66
Apa_cDNA144-1	*A. pacifica*	glutathione S-transferase delta-epsilon 2	*Tigriopus japonicus*	ACE81245	4.94E-75
Apa_cDNA144-2	*A. pacifica*	glutathione S-transferase delta-epsilon 2	*Tigriopus japonicus*	ACE81245	4.94E-75
Apa_cDNA145	*A. pacifica*	chromobox protein homolog 3 isoform x2	*Zootermopsis nevadensis*	KDR10033	5.75E-71
Apa_cDNA146-1	*A. pacifica*	DNA-directed RNA polymerase II subunit RPB9	*Drosophila melanogaster*	ACK77674	1.23E-70
Apa_cDNA146-2	*A. pacifica*	DNA-directed RNA polymerase II subunit RPB9	*Zootermopsis nevadensis*	KDR20481	2.85E-71
Apa_cDNA146-3	*A. pacifica*	DNA-directed RNA polymerase II subunit RPB9	*Drosophila melanogaster*	ACK77674	1.32E-70
Apa_cDNA147	*A. pacifica*	dynein light chain roadblock-type 2-like	*Caligus clemensi*	ACO15208	7.94E-47
Apa_cDNA148	*A. pacifica*	26S protease regulatory subunit S10B	*Culex quinquefasciatus*	XP_001845060	0
Apa_cDNA149	*A. pacifica*	copper/zinc superoxide dismutase	*Caligus clemensi*	ACO15446	1.15E-69
Apa_cDNA15	*A. pacifica*	60S ribosomal protein L12	*Culex quinquefasciatus*	XP_001865531	4.04E-95
Apa_cDNA150-1	*A. pacifica*	40S ribosomal protein S24	*Lepeophtheirus salmonis*	ACO12534	2.34E-34
Apa_cDNA150-2	*A. pacifica*	40S ribosomal protein S24	*Lepeophtheirus salmonis*	ACO12534	2.29E-34
Apa_cDNA150-3	*A. pacifica*	40S ribosomal protein S24	*Lepeophtheirus salmonis*	ACO12534	3.40E-34
Apa_cDNA150-4	*A. pacifica*	40S ribosomal protein S24	*Lepeophtheirus salmonis*	ACO12534	2.39E-34
Apa_cDNA150-5	*A. pacifica*	40S ribosomal protein S24	*Lepeophtheirus salmonis*	ACO12534	2.39E-34
Apa_cDNA150-6	*A. pacifica*	40S ribosomal protein S24	*Lepeophtheirus salmonis*	ACO12534	1.07E-33
Apa_cDNA150-7	*A. pacifica*	40S ribosomal protein S24	*Lepeophtheirus salmonis*	ACO12534	2.39E-34
Apa_cDNA150-8	*A. pacifica*	40S ribosomal protein S24	*Lepeophtheirus salmonis*	ACO12534	2.76E-33
Apa_cDNA151	*A. pacifica*	juvenile hormone-inducible	*Hydra vulgaris*	XP_002155193	1.04E-38
Apa_cDNA152-1	*A. pacifica*	ATP synthase E chain	*Acartia pacifica*	AGN29660	8.41E-41
Apa_cDNA152-2	*A. pacifica*	ATP synthase E chain	*Acartia pacifica*	AGN29660	8.35E-40
Apa_cDNA152-3	*A. pacifica*	ATP synthase E chain	*Acartia pacifica*	AGN29660	9.69E-41
Apa_cDNA152-4	*A. pacifica*	ATP synthase E chain	*Acartia pacifica*	AGN29660	3.11E-41
Apa_cDNA153-1	*A. pacifica*	probable rRNA-processing protein ebp2 homolog	*Caligus rogercresseyi*	ACO10230	1.53E-87

Apa_cDNA153-2	*A. pacifica*	probable rRNA-processing protein ebp2 homolog	*Caligus rogercresseyi*	ACO10230	2.56E-87
Apa_cDNA154	*A. pacifica*	anaphase-promoting complex subunit 15-like	*Branchiostoma floridae*	XP_002609161	3.56E-08
Apa_cDNA155-1	*A. pacifica*	60S ribosomal protein L32	*Nasonia vitripennis*	XP_001601522	1.76E-73
Apa_cDNA155-2	*A. pacifica*	60S ribosomal protein L32	*Nasonia vitripennis*	XP_001601522	1.76E-73
Apa_cDNA155-3	*A. pacifica*	60S ribosomal protein L32	*Nasonia vitripennis*	XP_001601522	1.05E-73
Apa_cDNA155-4	*A. pacifica*	60S ribosomal protein L32	*Nasonia vitripennis*	XP_001601522	1.09E-73
Apa_cDNA155-5	*A. pacifica*	60S ribosomal protein L32	*Nasonia vitripennis*	XP_001601522	1.09E-73
Apa_cDNA155-6	*A. pacifica*	60S ribosomal protein L32	*Nasonia vitripennis*	XP_001601522	1.05E-73
Apa_cDNA156	*A. pacifica*	proteasome subunit beta type-3-like	*Tribolium castaneum*	XP_008194648	1.43E-100
Apa_cDNA157-1	*A. pacifica*	60S ribosomal protein L9	*Acartia pacifica*	AGN29667	2.55E-133
Apa_cDNA157-2	*A. pacifica*	60S ribosomal protein L9	*Acartia pacifica*	AGN29667	3.49E-133
Apa_cDNA158	*A. pacifica*	chitin deacetylase-like isoform h	*Apis mellifera*	XP_006566088	2.72E-132
Apa_cDNA159-1	*A. pacifica*	60S ribosomal protein L23	*Acartia pacifica*	AGN29657	3.33E-92
Apa_cDNA159-2	*A. pacifica*	60S ribosomal protein L23	*Acartia pacifica*	AGN29657	7.80E-93
Apa_cDNA159-3	*A. pacifica*	60S ribosomal protein L23	*Acartia pacifica*	AGN29657	6.97E-93
Apa_cDNA160	*A. pacifica*	N-alpha-acetyltransferase 20	*Dendroctonus ponderosae*	AEE61811	5.00E-91
Apa_cDNA161	*A. pacifica*	HemK methyltransferase family member	*Cerapachys biroi*	EZA57918	1.46E-60
Apa_cDNA16-1	*A. pacifica*	40S ribosomal protein S27	*Acartia pacifica*	AGN29666	3.89E-54
Apa_cDNA162	*A. pacifica*	p53 and DNA damage-regulated protein 1	*Paracyclopina nana*	AIA61435	1.63E-25
Apa_cDNA16-2	*A. pacifica*	40S ribosomal protein S27	*Acartia pacifica*	AGN29666	1.72E-51
Apa_cDNA163	*A. pacifica*	glycogenin-1	*Caligus rogercresseyi*	ACO11092	9.43E-123
Apa_cDNA16-3	*A. pacifica*	40S ribosomal protein S27	*Acartia pacifica*	AGN29666	1.40E-51
Apa_cDNA16-4	*A. pacifica*	40S ribosomal protein S27	*Acartia pacifica*	AGN29666	8.27E-54
Apa_cDNA164-1	*A. pacifica*	nuclear autoantigenic sperm protein	*Penaeus monodon*	ACM66845	8.57E-32
Apa_cDNA164-2	*A. pacifica*	nuclear autoantigenic sperm protein	*Penaeus monodon*	ACM66845	9.05E-32
Apa_cDNA165	*A. pacifica*	Poly(A) RNA polymerase gld-2-like protein A	*Microplitis demolitor*	XP_008555871	2.30E-74
Apa_cDNA16-5	*A. pacifica*	40S ribosomal protein S27	*Acartia pacifica*	AGN29666	6.32E-53
Apa_cDNA166	*A. pacifica*	PREDICTED: protein LLP homolog isoform X1	*Microtus ochrogaster*	XP_005357977	1.57E-07
Apa_cDNA16-6	*A. pacifica*	40S ribosomal protein S27	*Acartia pacifica*	AGN29666	8.27E-54
Apa_cDNA167	*A. pacifica*	DNA-directed RNA polymerases I, II, and III subunit RPABC4-like	*Ixodes scapularis*	XP_002415907	1.96E-24
Apa_cDNA168	*A. pacifica*	proteasome subunit alpha type-3	*Branchiostoma floridae*	XP_002610181	9.06E-111
Apa_cDNA169-1	*A. pacifica*	40S ribosomal protein S25	*Acartia pacifica*	AGN29668	8.46E-49
Apa_cDNA169-2	*A. pacifica*	40S ribosomal protein S25	*Acartia pacifica*	AGN29668	8.46E-49
Apa_cDNA169-3	*A. pacifica*	40S ribosomal protein S25	*Acartia pacifica*	AGN29668	8.27E-48
Apa_cDNA169-4	*A. pacifica*	40S ribosomal protein S25	*Acartia pacifica*	AGN29668	8.46E-49
Apa_cDNA169-5	*A. pacifica*	40S ribosomal protein S25	*Acartia pacifica*	AGN29668	6.90E-48
Apa_cDNA169-6	*A. pacifica*	40S ribosomal protein S25	*Acartia pacifica*	AGN29668	6.45E-48
Apa_cDNA169-7	*A. pacifica*	40S ribosomal protein S25	*Acartia pacifica*	AGN29668	2.83E-50
Apa_cDNA169-8	*A. pacifica*	40S ribosomal protein S25	*Acartia pacifica*	AGN29668	2.73E-50
Apa_cDNA169-9	*A. pacifica*	40S ribosomal protein S25	*Acartia pacifica*	AGN29668	1.37E-48
Apa_cDNA170	*A. pacifica*	60S ribosomal protein L30	*Acartia pacifica*	AGN29635	7.69E-62
Apa_cDNA17-1	*A. pacifica*	40S ribosomal protein S27a	*Acartia pacifica*	AGN29644	6.22E-78
Apa_cDNA171-1	*A. pacifica*	60S ribosomal protein L30	*Acartia pacifica*	AGN29635	5.00E-75
Apa_cDNA171-2	*A. pacifica*	60S ribosomal protein L30	*Acartia pacifica*	AGN29635	3.70E-75
Apa_cDNA171-3	*A. pacifica*	60S ribosomal protein L30	*Acartia pacifica*	AGN29635	3.99E-75

Apa_cDNA17-2	*A. pacifica*	40S ribosomal protein S27a	*Acartia pacifica*	AGN29644	4.48E-74
Apa_cDNA172-1	*A. pacifica*	40S ribosomal protein S12	*Harpegnathos saltator*	EFN80764	1.17E-62
Apa_cDNA172-2	*A. pacifica*	40S ribosomal protein S12	*Apis florea*	XP_003689761	3.95E-62
Apa_cDNA172-3	*A. pacifica*	40S ribosomal protein S12	*Harpegnathos saltator*	EFN80764	6.89E-62
Apa_cDNA172-4	*A. pacifica*	40S ribosomal protein S12	*Apis florea*	XP_003689761	1.54E-62
Apa_cDNA172-5	*A. pacifica*	40S ribosomal protein S12	*Harpegnathos saltator*	EFN80764	6.05E-62
Apa_cDNA173	*A. pacifica*	thioredoxin domain-containing protein 17-like	*Daphnia pulex*	EFX66964	3.81E-35
Apa_cDNA174-1	*A. pacifica*	thymosin isoform 1	*Hodotermopsis sjostedti*	BAJ11759	5.22E-39
Apa_cDNA174-2	*A. pacifica*	thymosin isoform 1	*Hodotermopsis sjostedti*	BAJ11759	5.22E-39
Apa_cDNA175-1	*A. pacifica*	60S ribosomal protein L10	*Lepeophtheirus salmonis*	ABU41039	6.87E-136
Apa_cDNA175-2	*A. pacifica*	60S ribosomal protein L10	*Lepeophtheirus salmonis*	ABU41039	5.21E-135
Apa_cDNA176-1	*A. pacifica*	60S ribosomal protein L24	*Biphyllus lunatus*	CAJ17315	1.12E-50
Apa_cDNA176-2	*A. pacifica*	60S ribosomal protein L24	*Biphyllus lunatus*	CAJ17315	3.16E-50
Apa_cDNA177	*A. pacifica*	eukaryotic translation initiation factor 5A	*Lepeophtheirus salmonis*	ACO12518	1.17E-77
Apa_cDNA178-1	*A. pacifica*	matrix metallopeptidase 2	*Echinops telfairi*	XP_004704722	5.98E-06
Apa_cDNA178-2	*A. pacifica*	matrix metallopeptidase 2	*Echinops telfairi*	XP_004704722	1.10E-06
Apa_cDNA178-3	*A. pacifica*	matrix metallopeptidase 2	*Echinops telfairi*	XP_004704722	1.10E-06
Apa_cDNA179	*A. pacifica*	cytochrome c	*Litopenaeus vannamei*	AGX27197	2.10E-47
Apa_cDNA180	*A. pacifica*	40S ribosomal protein S14	*Caligus clemensi*	ACO14952	9.10E-87
Apa_cDNA181	*A. pacifica*	actin-related protein 2/3 complex subunit 3	*Caligus clemensi*	ACO15095	2.20E-88
Apa_cDNA18-1	*A. pacifica*	40S ribosomal protein S15	*Georissus sp. APV-2005*	CAJ17200	4.18E-71
Apa_cDNA18-2	*A. pacifica*	40S ribosomal protein S15	*Georissus sp. APV-2005*	CAJ17200	5.14E-71
Apa_cDNA182-1	*A. pacifica*	40S ribosomal protein S3a	*Lepeophtheirus salmonis*	ACO12723	1.80E-139
Apa_cDNA182-2	*A. pacifica*	40S ribosomal protein S3a	*Lepeophtheirus salmonis*	ACO12723	5.48E-139
Apa_cDNA18-3	*A. pacifica*	40S ribosomal protein S15	*Nematostella vectensis*	XP_001637830	2.16E-71
Apa_cDNA183-1	*A. pacifica*	protein extra bases	*Zootermopsis nevadensis*	KDR20399	4.36E-154
Apa_cDNA183-2	*A. pacifica*	protein extra bases	*Zootermopsis nevadensis*	KDR20399	4.46E-142
Apa_cDNA184	*A. pacifica*	small nuclear ribonucleoprotein g	*Pediculus humanus corporis*	XP_002429935	7.71E-31
Apa_cDNA185	*A. pacifica*	AGAP008195-PA	*Anopheles gambiae str. PEST*	XP_317273	4.29E-77
Apa_cDNA186	*A. pacifica*	bifunctional aminoacyl-tRNA synthetase	*Acyrthosiphon pisum*	XP_008183274	4.25E-154
Apa_cDNA187	*A. pacifica*	prefoldin subunit 3	*Lepeophtheirus salmonis*	ADD38576	8.19E-72
Apa_cDNA188-1	*A. pacifica*	NADH dehydrogenase	*Danaus plexippus*	EHJ65227	4.98E-14
Apa_cDNA188-2	*A. pacifica*	NADH dehydrogenase	*Danaus plexippus*	EHJ65227	4.98E-14
Apa_cDNA188-3	*A. pacifica*	NADH dehydrogenase	*Danaus plexippus*	EHJ65227	4.98E-14
Apa_cDNA188-4	*A. pacifica*	NADH dehydrogenase	*Danaus plexippus*	EHJ65227	9.26E-14
Apa_cDNA189	*A. pacifica*	dynactin subunit 4	*Taeniopygia guttata*	NP_001232452	1.81E-55
Apa_cDNA19	*A. pacifica*	copper/zinc superoxide dismutase	*Caligus clemensi*	ACO15446	1.15E-69
Apa_cDNA190	*A. pacifica*	DNA polymerase epsilon subunit 3	*Lepeophtheirus salmonis*	ADD38397	5.49E-36
Apa_cDNA191	*A. pacifica*	phenylalanine-tRNA mitochondrial-like isoform X1	*Solenopsis invicta*	EFZ14320	1.71E-156
Apa_cDNA192	*A. pacifica*	aldehyde mitochondrial isoform X1	*Nematostella vectensis*	XP_001639716	0
Apa_cDNA193	*A. pacifica*	peptidyl-prolyl cis-trans isomerase	*Anopheles darlingi*	ETN67998	1.27E-89

Apa_cDNA194	*A. pacifica*	Predicted:tetraspanin-31	*Tribolium castaneum*	XP_968578	1.15E-43
Apa_cDNA195-1	*A. pacifica*	60S ribosomal protein L34	*Caligus clemensi*	ACO14672	2.52E-49
Apa_cDNA195-2	*A. pacifica*	60S ribosomal protein L34	*Caligus clemensi*	ACO14672	9.17E-50
Apa_cDNA196	*A. pacifica*	prefoldin subunit 1	*Daphnia pulex*	EFX83244	6.80E-40
Apa_cDNA197	*A. pacifica*	elongation factor 1-alpha	*Nasonia vitripennis*	NP_001166227	0
Apa_cDNA198	*A. pacifica*	chorion peroxidase-like	*Daphnia pulex*	EFX81608	1.46E-113
Apa_cDNA199	*A. pacifica*	proteasome assembly chaperone 4-like	*Saccoglossus kowalevskii*	XP_002741269	5.99E-13
Apa_cDNA2	*A. pacifica*	inorganic pyrophosphatase	*Lepeophtheirus salmonis*	ADD38827	2.88E-126
Apa_cDNA200	*A. pacifica*	isobutyryl-CoA dehydrogenase, mitochondrial-like	*Oryzias latipes*	XP_004075393	0
Apa_cDNA20-1	*A. pacifica*	40S ribosomal protein S11	*Acartia pacifica*	AGN29665	2.26E-97
Apa_cDNA201-1	*A. pacifica*	ubiquitin-conjugating enzyme E2-24 kDa	*Daphnia pulex*	EFX85571	3.02E-96
Apa_cDNA201-2	*A. pacifica*	40S ribosomal protein S8	*Lepeophtheirus salmonis*	ACO12033	3.00E-110
Apa_cDNA202	*A. pacifica*	chitin deacetylase 1	*Apis mellifera*	XP_391915	0
Apa_cDNA20-2	*A. pacifica*	40S ribosomal protein S11	*Acartia pacifica*	AGN29665	1.62E-98
Apa_cDNA20-3	*A. pacifica*	40S ribosomal protein S11	*Acartia pacifica*	AGN29665	1.72E-98
Apa_cDNA203-1	*A. pacifica*	60S ribosomal protein L6	*Zootermopsis nevadensis*	KDR23771	8.90E-73
Apa_cDNA203-2	*A. pacifica*	60S ribosomal protein L6	*Zootermopsis nevadensis*	KDR23771	4.66E-73
Apa_cDNA20-4	*A. pacifica*	40S ribosomal protein S11	*Acartia pacifica*	AGN29665	4.45E-97
Apa_cDNA204-1	*A. pacifica*	60S ribosomal protein L17	*Lepeophtheirus salmonis*	ACO13139	6.40E-98
Apa_cDNA204-2	*A. pacifica*	60S ribosomal protein L17	*Lepeophtheirus salmonis*	ACO13139	9.63E-98
Apa_cDNA20-5	*A. pacifica*	40S ribosomal protein S11	*Acartia pacifica*	AGN29665	5.11E-98
Apa_cDNA205-1	*A. pacifica*	probable nucleolar GTP-binding protein 1-like	*Daphnia pulex*	EFX82588	0
Apa_cDNA205-2	*A. pacifica*	probable nucleolar GTP-binding protein 1-like	*Daphnia pulex*	EFX82588	0
Apa_cDNA206	*A. pacifica*	fumarate hydratase, mitochondrial	*Daphnia pulex*	EFX85065	0
Apa_cDNA207	*A. pacifica*	60S ribosomal protein L18a	*Caligus clemensi*	ACO14776	1.77E-97
Apa_cDNA208	*A. pacifica*	cytochrome c oxidase polypeptide	*Tribolium castaneum*	EFA07589	2.67E-08
Apa_cDNA209	*A. pacifica*	isoform A	*Bactrocera oleae*	ADQ64580	1.47E-11
Apa_cDNA21	*A. pacifica*	NAD kinase, mitochondrial-like	*Daphnia pulex*	EFX80504	2.99E-137
Apa_cDNA211	*A. pacifica*	60S ribosomal protein L4	*Lepeophtheirus salmonis*	ACO12528	0
Apa_cDNA212	*A. pacifica*	nucleoporin Nup43	*Lepeophtheirus salmonis*	ADD38156	1.10E-91
Apa_cDNA213	*A. pacifica*	60S ribosomal protein L26	*Tribolium castaneum*	XP_972048	2.05E-81
Apa_cDNA214	*A. pacifica*	growth arrest and DNA-damage-inducible proteins-interacting protein 1	*Lepeophtheirus salmonis*	ACO12905	1.13E-19
Apa_cDNA215	*A. pacifica*	elongation factor 1-alpha	*Nasonia vitripennis*	NP_001166227	0
Apa_cDNA216	*A. pacifica*	heat shock protein beta-1	*Acartia pacifica*	AGN29598	7.33E-148
Apa_cDNA217-1	*A. pacifica*	60S acidic ribosomal protein P2	*Lepeophtheirus salmonis*	ACO12871	5.67E-07
Apa_cDNA217-2	*A. pacifica*	60S acidic ribosomal protein P2	*Xenopus (Silurana) tropicalis*	NP_001016645	1.53E-07
Apa_cDNA218-1	*A. pacifica*	ras-related protein Rab-1A	*Haliotis discus discus*	ABO26625	4.43E-127
Apa_cDNA218-2	*A. pacifica*	ras-related protein Rab-1A	*Haliotis discus discus*	ABO26625	1.04E-126
Apa_cDNA219	*A. pacifica*	60S ribosomal protein L5	*Danaus plexippus*	EHJ70750	1.41E-144
Apa_cDNA220	*A. pacifica*	profilin	*Lepeophtheirus salmonis*	ACO12777	1.83E-49

Apa_cDNA221	*A. pacifica*	gamma-secretase subunit PEN-2	*Drosophila grimshawi*	XP_001986974	1.18E-29
Apa_cDNA22-1	*A. pacifica*	tubulin alpha-1 chain	*Bemisia tabaci*	AGJ76540	0
Apa_cDNA222	*A. pacifica*	endonuclease III-like protein 1	*Acyrthosiphon pisum*	XP_001949525	1.54E-104
Apa_cDNA22-2	*A. pacifica*	tubulin alpha-1 chain	*Bemisia tabaci*	AGJ76540	0
Apa_cDNA223	*A. pacifica*	WD repeat-containing protein 55	*Caligus rogercresseyi*	ACO10563	3.41E-108
Apa_cDNA22-3	*A. pacifica*	tubulin alpha-1 chain	*Bemisia tabaci*	AGJ76540	0
Apa_cDNA224	*A. pacifica*	NAD-dependent deacetylase sirtuin-4	*Culex quinquefasciatus*	XP_001843271	1.38E-103
Apa_cDNA22-4	*A. pacifica*	tubulin alpha-1 chain	*Canis lupus familiaris*	XP_005640747	0
Apa_cDNA225	*A. pacifica*	speckle-type POZ protein	*Mus musculus*	EDL38714	2.23E-34
Apa_cDNA226	*A. pacifica*	GTP-binding nuclear protein Ran	*Solenopsis invicta*	EFZ10904	2.56E-132
Apa_cDNA227-1	*A. pacifica*	transmembrane emp24 domain-containing protein 2 isoform X2	*Branchiostoma floridae*	XP_002612119	1.58E-100
Apa_cDNA227-2	*A. pacifica*	transmembrane emp24 domain-containing protein 2 isoform X2	*Branchiostoma floridae*	XP_002612119	1.19E-97
Apa_cDNA228	*A. pacifica*	unknown protein			
Apa_cDNA229	*A. pacifica*	vacuolar ATP synthase 16 kDa proteolipid subunit	*Camponotus floridanus*	EFN66078	1.28E-56
Apa_cDNA230	*A. pacifica*	PREDICTED: stress-associated endoplasmic reticulum protein 2-like isoform X1	*Ceratitis capitata*	XP_004519873	3.05E-15
Apa_cDNA231	*A. pacifica*	protein disulfide isomerase	*Anopheles sinensis*	KFB50164	0
Apa_cDNA23-1	*A. pacifica*	40S ribosomal protein S18	*Caligus rogercresseyi*	ACO11045	6.03E-79
Apa_cDNA232	*A. pacifica*	chaperone protein dnaJ	*Aedes aegypti*	XP_001661333	1.41E-98
Apa_cDNA23-2	*A. pacifica*	40S ribosomal protein S18	*Caligus rogercresseyi*	ACO11045	4.09E-79
Apa_cDNA233	*A. pacifica*	ubiquitin thioesterase OTU1	*Capitella teleta*	ELT95674	5.26E-65
Apa_cDNA23-3	*A. pacifica*	40S ribosomal protein S18	*Caligus rogercresseyi*	ACO11045	3.74E-79
Apa_cDNA234	*A. pacifica*	peptidyl-prolyl cis-trans isomerase NIMA-interacting 1	*Caligus rogercresseyi*	ACO11316	1.33E-67
Apa_cDNA23-4	*A. pacifica*	40S ribosomal protein S18	*Caligus rogercresseyi*	ACO11045	9.00E-90
Apa_cDNA235	*A. pacifica*	ankyrin	*Gloeophyllum trabeum ATCC 11539*	XP_007865608	8.53E-27
Apa_cDNA23-5	*A. pacifica*	40S ribosomal protein S18	*Caligus rogercresseyi*	ACO11045	5.29E-79
Apa_cDNA236	*A. pacifica*	unknown protein			
Apa_cDNA237	*A. pacifica*	protein OPI10 homolog	*Capitella teleta*	ELU16398	1.96E-62
Apa_cDNA238	*A. pacifica*	isoform A	*Daphnia pulex*	EFX76719	8.88E-18
Apa_cDNA239	*A. pacifica*	protein OPI10 homolog	*Capitella teleta*	ELU16398	1.96E-62
Apa_cDNA240	*A. pacifica*	PREDICTED: uncharacterized protein LOC101670138	*Mustela putorius furo*	XP_004755978	3.07E-38
Apa_cDNA241-1	*A. pacifica*	hypothetical protein YQE_10512, partial	*Dendroctonus ponderosae*	ENN72863	7.84E-06
Apa_cDNA241-2	*A. pacifica*	hypothetical protein	*Dendroctonus ponderosae*	ENN70324	6.41E-06
Apa_cDNA243	*A. pacifica*	up-regulated during skeletal muscle growth protein 5 isoform X1	*Drosophila virilis*	XP_002059507	0.00038528
Apa_cDNA244	*A. pacifica*	hypothetical protein	*Hydra vulgaris*	XP_002161777	4.00E-38
Apa_cDNA245	*A. pacifica*	protein FAM32A-like	*Drosophila ananassae*	XP_001965229	1.61E-11
Apa_cDNA246	*A. pacifica*	UPF0668 protein C10orf76 homolog	*Zootermopsis nevadensis*	KDR10796	0
Apa_cDNA247	*A. pacifica*	DnaJ-like protein subfamily C member 24	*Zootermopsis nevadensis*	KDR17004	5.91E-21
Apa_cDNA248	*A. pacifica*	CG18508	*Daphnia pulex*	EFX74301	1.01E-10

Apa_cDNA249	*A. pacifica*	AGAP011412-PA	*Anopheles gambiae str. PEST*	XP_317897	3.70E-20
Apa_cDNA250	*A. pacifica*	proteasome assembly chaperone 1	*Apis dorsata*	XP_006614009	4.56E-12
Apa_cDNA251	*A. pacifica*	PREDICTED: hypothetical protein LOC100748697	*Bombus impatiens*	XP_003489803	4.39E-05
Apa_cDNA25-1	*A. pacifica*	60S ribosomal protein L15	*Acartia pacifica*	AGN29643	2.35E-120
Apa_cDNA252	*A. pacifica*	unknown protein			
Apa_cDNA25-2	*A. pacifica*	60S ribosomal protein L15	*Acartia pacifica*	AGN29643	2.44E-120
Apa_cDNA253	*A. pacifica*	unknown protein			
Apa_cDNA25-3	*A. pacifica*	60S ribosomal protein L15	*Acartia pacifica*	AGN29643	6.31E-125
Apa_cDNA254	*A. pacifica*	unknown protein			
Apa_cDNA255	*A. pacifica*	unknown protein			
Apa_cDNA256-1	*A. pacifica*	unknown protein			
Apa_cDNA256-2	*A. pacifica*	unknown protein			
Apa_cDNA256-3	*A. pacifica*	unknown protein			
Apa_cDNA257	*A. pacifica*	unknown protein			
Apa_cDNA258	*A. pacifica*	unknown protein			
Apa_cDNA258-1	*A. pacifica*	unknown protein			
Apa_cDNA258-2	*A. pacifica*	unknown protein			
Apa_cDNA258-3	*A. pacifica*	unknown protein			
Apa_cDNA260-1	*A. pacifica*	unknown protein			
Apa_cDNA260-2	*A. pacifica*	unknown protein			
Apa_cDNA260-3	*A. pacifica*	unknown protein			
Apa_cDNA26-1	*A. pacifica*	40S ribosomal protein S13	*Latimeria chalumnae*	XP_005990576	4.47E-93
Apa_cDNA261-1	*A. pacifica*	PREDICTED: zinc finger protein 25	*Myotis davidii*	XP_006774592	1.23E-23
Apa_cDNA261-2	*A. pacifica*	zinc finger protein 37a	*Saimiri boliviensis boliviensis*	XP_003930301	1.38E-23
Apa_cDNA262	*A. pacifica*	unknown protein			
Apa_cDNA26-2	*A. pacifica*	40S ribosomal protein S13	*Latimeria chalumnae*	XP_005990576	2.02E-90
Apa_cDNA263	*A. pacifica*	ADP,ATP carrier protein 3	*Caligus rogercresseyi*	ACO10375	2.11E-157
Apa_cDNA26-3	*A. pacifica*	40S ribosomal protein S13	*Latimeria chalumnae*	XP_005990576	4.30E-93
Apa_cDNA26-4	*A. pacifica*	40S ribosomal protein S13	*Latimeria chalumnae*	XP_005990576	2.15E-93
Apa_cDNA264-1	*A. pacifica*	unknown protein			
Apa_cDNA264-2	*A. pacifica*	unknown protein			
Apa_cDNA265	*A. pacifica*	T-complex protein 1 subunit eta, partial	*Calanus helgolandicus*	AHC70360	1.12E-12
Apa_cDNA26-5	*A. pacifica*	40S ribosomal protein S13	*Coptotermes formosanus*	AGM32421	3.72E-90
Apa_cDNA266	*A. pacifica*	apoptosis regulator Bcl-2-like	*Lottia gigantea*	ESO88195	4.94E-21
Apa_cDNA26-6	*A. pacifica*	40S ribosomal protein S13	*Cricetulus griseus*	NP_001231462	2.65E-92
Apa_cDNA267	*A. pacifica*	unknown protein			
Apa_cDNA268	*A. pacifica*	unknown protein			
Apa_cDNA269	*A. pacifica*	unknown protein			
Apa_cDNA27	*A. pacifica*	ATP synthase subunit O, mitochondrial precursor	*Caligus clemensi*	ACO15580	5.25E-84
Apa_cDNA270	*A. pacifica*	unknown protein			
Apa_cDNA271	*A. pacifica*	unknown protein			
Apa_cDNA272	*A. pacifica*	40S ribosomal protein S5	*Lysiphlebus testaceipes*	AAX62424	4.55E-114
Apa_cDNA273	*A. pacifica*	unknown protein			
Apa_cDNA274	*A. pacifica*	unknown protein			
Apa_cDNA275	*A. pacifica*	39S ribosomal protein L34, mitochondrial, putative	*Bombyx mori*	XP_004929612	0.00066469
Apa_cDNA276	*A. pacifica*	unknown protein			
Apa_cDNA277	*A. pacifica*	unknown protein			
Apa_cDNA278	*A. pacifica*	dynactin subunit 6	*Branchiostoma floridae*	XP_002613156	2.83E-17

Apa_cDNA279	*A. pacifica*	unknown protein			
Apa_cDNA28	*A. pacifica*	ATP synthase subunit f, mitochondrial	*Lepeophtheirus salmonis*	ACO12343	6.98E-42
Apa_cDNA280	*A. pacifica*	unknown protein			
Apa_cDNA281	*A. pacifica*	unknown protein			
Apa_cDNA282	*A. pacifica*	unknown protein			
Apa_cDNA283	*A. pacifica*	unknown protein			
Apa_cDNA284	*A. pacifica*	unknown protein			
Apa_cDNA285	*A. pacifica*	unknown protein			
Apa_cDNA286	*A. pacifica*	unknown protein			
Apa_cDNA287-1	*A. pacifica*	unknown protein			
Apa_cDNA287-2	*A. pacifica*	unknown protein			
Apa_cDNA288	*A. pacifica*	Apa_cDNA288	*LOW QUALITY PROTEIN: putative pre-mRNA-splicing factor ATP-dependent RNA helicase DHX16-like*	Oryzias latipes	XP_004078 407
Apa_cDNA289	*A. pacifica*	Apa_cDNA289	*WD repeat-containing protein 36*	Tribolium castaneum	XP_966791
Apa_cDNA29	*A. pacifica*	ATP synthase subunit epsilon, mitochondrial	*Lepeophtheirus salmonis*	ADD38640	6.52E-17
Apa_cDNA290	*A. pacifica*	Apa_cDNA290	*60S ribosomal protein L13*	Lepeophtheirus salmonis	ADD38054
Apa_cDNA291	*A. pacifica*	Apa_cDNA291	*chromobox protein homolog 1-like*	Megachile rotundata	XP_003704 915
Apa_cDNA292	*A. pacifica*	Apa_cDNA292	*general transcription factor IIF subunit 1-like*	Apis mellifera	XP_006564 343
Apa_cDNA293	*A. pacifica*	Apa_cDNA293	*DNA repair protein XRCC1-like*	Aplysia californica	XP_005111 437
Apa_cDNA294	*A. pacifica*	Apa_cDNA294	*PREDICTED: DNA repair protein RAD52 homolog*	Helobdella robusta	ESO06134
Apa_cDNA295	*A. pacifica*	Apa_cDNA295	*unknown protein*		
Apa_cDNA296	*A. pacifica*	Apa_cDNA296	*ef-hand calcium-binding domain-containing protein 5 isoform X1*	Trypanosoma congolense IL3000	CCD15878
Apa_cDNA297	*A. pacifica*	Apa_cDNA297	*unknown protein*		
Apa_cDNA298	*A. pacifica*	Apa_cDNA298	*unknown protein*		
Apa_cDNA299	*A. pacifica*	Apa_cDNA299	*unknown protein*		
Apa_cDNA3	*A. pacifica*	cytochrome c oxidase subunit 6B1-like	*Aplysia californica*	XP_005100893	1.86E-31
Apa_cDNA30	*A. pacifica*	ATP synthase subunit epsilon, mitochondrial	*Lepeophtheirus salmonis*	ADD38640	6.38E-17
Apa_cDNA300	*A. pacifica*	Apa_cDNA300	*unknown protein*		
Apa_cDNA301	*A. pacifica*	Apa_cDNA301	*zinc finger protein Ran-binding domain-containing protein, putative*	Pediculus humanus corporis	XP_002425 740
Apa_cDNA302	*A. pacifica*	Apa_cDNA302	*unknown protein*		
Apa_cDNA303	*A. pacifica*	Apa_cDNA303	*unknown protein*		
Apa_cDNA31	*A. pacifica*	ATP synthase subunit gamma, mitochondrial precursor	*Lepeophtheirus salmonis*	ACO12344	1.51E-142
Apa_cDNA32-1	*A. pacifica*	40S ribosomal protein S26	*Lepeophtheirus salmonis*	ACO12442	1.69E-35
Apa_cDNA32-2	*A. pacifica*	40S ribosomal protein S26	*Lepeophtheirus salmonis*	ACO12442	1.31E-35
Apa_cDNA32-3	*A. pacifica*	40S ribosomal protein S26	*Lepeophtheirus salmonis*	ACO12442	1.20E-35
Apa_cDNA33-1	*A. pacifica*	60S ribosomal protein L38	*Tribolium castaneum*	XP_970928	4.12E-34

Apa_cDNA33-2	*A. pacifica*	60S ribosomal protein L38	*Tribolium castaneum*	XP_970928	1.63E-34
Apa_cDNA33-3	*A. pacifica*	60S ribosomal protein L38	*Tribolium castaneum*	XP_970928	7.49E-35
Apa_cDNA33-4	*A. pacifica*	60S ribosomal protein L38	*Tribolium castaneum*	XP_970928	7.24E-35
Apa_cDNA34	*A. pacifica*	alkylated DNA repair protein alkB-like protein	*Zootermopsis nevadensis*	KDR18060	5.22E-83
Apa_cDNA35-1	*A. pacifica*	guanine nucleotide-binding protein subunit beta-2-like 1	*Caligus clemensi*	ACO15464	0
Apa_cDNA35-2	*A. pacifica*	guanine nucleotide-binding protein subunit beta-2-like 1	*Caligus clemensi*	ACO15464	0
Apa_cDNA36	*A. pacifica*	proteasome subunit alpha type-5	*Daphnia pulex*	EFX69073	7.97E-133
Apa_cDNA37	*A. pacifica*	calcineurin subunit B isoform 1	*Lepeophtheirus salmonis*	ACO12203	1.90E-65
Apa_cDNA38-1	*A. pacifica*	60S ribosomal protein L18	*Barentsia elongata*	ABW90395	1.50E-90
Apa_cDNA38-2	*A. pacifica*	60S ribosomal protein L18	*Barentsia elongata*	ABW90395	1.11E-87
Apa_cDNA38-3	*A. pacifica*	60S ribosomal protein L18	*Barentsia elongata*	ABW90395	1.77E-89
Apa_cDNA38-4	*A. pacifica*	60S ribosomal protein L18	*Barentsia elongata*	ABW90395	2.29E-90
Apa_cDNA39-1	*A. pacifica*	60S ribosomal protein L28	*Acartia pacifica*	AGN29640	3.21E-91
Apa_cDNA39-2	*A. pacifica*	60S ribosomal protein L28	*Acartia pacifica*	AGN29640	8.19E-92
Apa_cDNA39-3	*A. pacifica*	60S ribosomal protein L28	*Acartia pacifica*	AGN29640	1.03E-91
Apa_cDNA40	*A. pacifica*	28S ribosomal protein S2, mitochondrial	*Lepeophtheirus salmonis*	ADD24519	2.71E-72
Apa_cDNA41	*A. pacifica*	adenosine 3'-phospho 5'-phosphosulfate transporter	*Zootermopsis nevadensis*	KDR09040	8.30E-156
Apa_cDNA4-1	*A. pacifica*	40S ribosomal protein S30	*Acartia pacifica*	AGN29662	6.46E-41
Apa_cDNA42	*A. pacifica*	dual specificity protein phosphatase 16-like	*Hydra vulgaris*	XP_004206413	1.16E-25
Apa_cDNA4-2	*A. pacifica*	40S ribosomal protein S30	*Acartia pacifica*	AGN29662	2.76E-60
Apa_cDNA43	*A. pacifica*	S1 RNA-binding domain-containing protein 1	*Ixodes scapularis*	XP_002411275	7.19E-13
Apa_cDNA4-3	*A. pacifica*	40S ribosomal protein S30	*Acartia pacifica*	AGN29662	1.52E-58
Apa_cDNA44	*A. pacifica*	28S ribosomal protein S15, mitochondrial	*Lepeophtheirus salmonis*	ACO11925	4.18E-45
Apa_cDNA45-1	*A. pacifica*	60S ribosomal protein L35	*Dendroctonus ponderosae*	AEE61452	2.67E-45
Apa_cDNA45-2	*A. pacifica*	60S ribosomal protein L35	*Dendroctonus ponderosae*	AEE61452	9.33E-47
Apa_cDNA45-3	*A. pacifica*	60S ribosomal protein L35	*Dendroctonus ponderosae*	AEE61452	9.33E-47
Apa_cDNA45-4	*A. pacifica*	60S ribosomal protein L35	*Dendroctonus ponderosae*	AEE61452	9.33E-47
Apa_cDNA45-5	*A. pacifica*	60S ribosomal protein L35	*Dendroctonus ponderosae*	AEE61452	9.33E-47
Apa_cDNA45-6	*A. pacifica*	60S ribosomal protein L35	*Dendroctonus ponderosae*	AEE61452	9.16E-47
Apa_cDNA46-1	*A. pacifica*	60S ribosomal protein L23a	*Daphnia pulex*	EFX84946	3.56E-67
Apa_cDNA46-2	*A. pacifica*	60S ribosomal protein L23a	*Daphnia pulex*	EFX84946	5.03E-67
Apa_cDNA46-3	*A. pacifica*	60S ribosomal protein L23a	*Daphnia pulex*	EFX84946	4.09E-67
Apa_cDNA47	*A. pacifica*	peptidyl-prolyl cis-trans isomerase FKBP10-like	*Meleagris gallopavo*	XP_003213158	1.98E-34
Apa_cDNA48	*A. pacifica*	26S proteasome non-ATPase regulatory subunit 14	*Maylandia zebra*	XP_004549823	2.73E-83
Apa_cDNA49-1	*A. pacifica*	cytochrome c oxidase subunit, mitochondrial-like	*Riptortus pedestris*	BAN21304	5.31E-19
Apa_cDNA49-2	*A. pacifica*	cytochrome c oxidase subunit, mitochondrial-like	*Riptortus pedestris*	BAN21304	8.68E-19
Apa_cDNA50	*A. pacifica*	probable aspartyl aminopeptidase-like	*Guillardia theta CCMP2712*	XP_005838427	1.48E-170
Apa_cDNA5-1	*A. pacifica*	40S ribosomal protein S21	*Acartia pacifica*	AGN29649	7.31E-47

Apa_cDNA51-1	*A. pacifica*	H/ACA ribonucleoprotein complex subunit 2-like	*Drosophila virilis*	XP_002048003	7.59E-50
Apa_cDNA51-2	*A. pacifica*	H/ACA ribonucleoprotein complex subunit 2-like	*Drosophila virilis*	XP_002048003	7.59E-50
Apa_cDNA5-2	*A. pacifica*	40S ribosomal protein S21	*Acartia pacifica*	AGN29649	6.92E-47
Apa_cDNA52-1	*A. pacifica*	40S ribosomal protein S15a	*Acartia pacifica*	AGN29647	3.60E-89
Apa_cDNA52-10	*A. pacifica*	40S ribosomal protein S15a	*Acartia pacifica*	AGN29647	3.74E-89
Apa_cDNA52-11	*A. pacifica*	40S ribosomal protein S15a	*Acartia pacifica*	AGN29647	3.74E-89
Apa_cDNA52-12	*A. pacifica*	40S ribosomal protein S15a	*Acartia pacifica*	AGN29647	7.72E-88
Apa_cDNA52-13	*A. pacifica*	40S ribosomal protein S15a	*Acartia pacifica*	AGN29647	5.10E-89
Apa_cDNA52-14	*A. pacifica*	40S ribosomal protein S15a	*Acartia pacifica*	AGN29647	3.74E-89
Apa_cDNA52-15	*A. pacifica*	40S ribosomal protein S15a	*Acartia pacifica*	AGN29647	3.74E-89
Apa_cDNA52-2	*A. pacifica*	40S ribosomal protein S15a	*Acartia pacifica*	AGN29647	3.74E-89
Apa_cDNA52-3	*A. pacifica*	40S ribosomal protein S15a	*Acartia pacifica*	AGN29647	3.74E-89
Apa_cDNA52-4	*A. pacifica*	40S ribosomal protein S15a	*Acartia pacifica*	AGN29647	3.60E-89
Apa_cDNA52-5	*A. pacifica*	40S ribosomal protein S15a	*Acartia pacifica*	AGN29647	6.60E-88
Apa_cDNA52-6	*A. pacifica*	40S ribosomal protein S15a	*Acartia pacifica*	AGN29647	3.74E-89
Apa_cDNA52-7	*A. pacifica*	40S ribosomal protein S15a	*Acartia pacifica*	AGN29647	1.64E-88
Apa_cDNA52-8	*A. pacifica*	40S ribosomal protein S15a	*Acartia pacifica*	AGN29647	3.46E-89
Apa_cDNA52-9	*A. pacifica*	40S ribosomal protein S15a	*Acartia pacifica*	AGN29647	3.60E-89
Apa_cDNA53	*A. pacifica*	ATP-dependent RNA helicase DDX39	*Caligus rogercresseyi*	ACO10850	9.90E-140
Apa_cDNA5-3	*A. pacifica*	40S ribosomal protein S21	*Acartia pacifica*	AGN29649	4.84E-47
Apa_cDNA54	*A. pacifica*	ATP-dependent RNA helicase DDX39	*Caligus rogercresseyi*	ACO10850	0
Apa_cDNA55	*A. pacifica*	peptidyl-tRNA hydrolase 2, mitochondrial-like	*Chinchilla lanigera*	XP_005394038	1.14E-39
Apa_cDNA56-1	*A. pacifica*	vacuolar protein sorting-associated protein 29-like	*Tribolium castaneum*	XP_969121	1.15E-100
Apa_cDNA56-2	*A. pacifica*	vacuolar protein sorting-associated protein 29-like	*Tribolium castaneum*	XP_969121	1.47E-102
Apa_cDNA57-1	*A. pacifica*	nucleoside diphosphate kinase A 1	*Lepeophtheirus salmonis*	ACO12519	4.26E-89
Apa_cDNA57-2	*A. pacifica*	nucleoside diphosphate kinase A 1	*Lepeophtheirus salmonis*	ACO12519	5.51E-88
Apa_cDNA57-3	*A. pacifica*	nucleoside diphosphate kinase A 1	*Lepeophtheirus salmonis*	ACO12519	1.28E-86
Apa_cDNA57-4	*A. pacifica*	nucleoside diphosphate kinase A 1	*Lepeophtheirus salmonis*	ACO12519	1.84E-88
Apa_cDNA57-5	*A. pacifica*	nucleoside diphosphate kinase A 1	*Lepeophtheirus salmonis*	ACO12519	2.22E-88
Apa_cDNA57-6	*A. pacifica*	nucleoside diphosphate kinase A 1	*Lepeophtheirus salmonis*	ACO12519	3.15E-87
Apa_cDNA58	*A. pacifica*	metalloendopeptidase OMA1, mitochondrial	*Ixodes scapularis*	XP_002413036	1.23E-86
Apa_cDNA59	*A. pacifica*	stathmin-4-like isoform 2	*Megachile rotundata*	XP_003707815	8.72E-13
Apa_cDNA60	*A. pacifica*	ribonucleoside-diphosphate reductase subunit M2	*Anopheles sinensis*	KFB51535	0
Apa_cDNA61	*A. pacifica*	mitochondrial prohibitin complex protein 2	*Lepeophtheirus salmonis*	ACO12491	4.06E-135
Apa_cDNA6-1	*A. pacifica*	60S ribosomal protein L41	*Drosophila melanogaster*	NM_001014551	5.00E-22
Apa_cDNA62	*A. pacifica*	endonuclease 8-like 3	*Chrysemys picta bellii*	XP_005304226	2.69E-74
Apa_cDNA6-2	*A. pacifica*	60S ribosomal protein L41	*Bombyx mori*	NM_001042449	8.00E-15
Apa_cDNA6-3	*A. pacifica*	60S ribosomal protein L41	*Acartia pacifica*	KC989864	0
Apa_cDNA63-1	*A. pacifica*	60S ribosomal protein L38		Q56FC8	2.13E-33
Apa_cDNA63-2	*A. pacifica*	60S ribosomal protein L38		Q56FC8	2.13E-33
Apa_cDNA6-4	*A. pacifica*	60S ribosomal protein L41	*Macaca mulatta*	XP_002804534.1	2.00E+00

Apa_cDNA64-1	*A. pacifica*	60S ribosomal protein L38	*Apis mellifera*	XP_001120263	6.05E-24
Apa_cDNA64-2	*A. pacifica*	60S ribosomal protein L38	*Apis mellifera*	XP_001120263	3.36E-37
Apa_cDNA64-3	*A. pacifica*	60S ribosomal protein L38	*Apis mellifera*	XP_001120263	3.37E-37
Apa_cDNA64-4	*A. pacifica*	60S ribosomal protein L38	*Apis mellifera*	XP_001120263	1.44E-36
Apa_cDNA64-5	*A. pacifica*	60S ribosomal protein L38	*Apis mellifera*	XP_001120263	1.44E-36
Apa_cDNA64-6	*A. pacifica*	60S ribosomal protein L38	*Apis mellifera*	XP_001120263	1.29E-36
Apa_cDNA65	*A. pacifica*	40S ribosomal protein S23	*Lepeophtheirus salmonis*	ACO12024	4.48E-87
Apa_cDNA6-5	*A. pacifica*	60S ribosomal protein L41	*Acartia pacifica*	KC989864	0
Apa_cDNA66	*A. pacifica*	apoptosis-stimulating of p53 protein 1	*Danaus plexippus*	EHJ73745	1.30E-33
Apa_cDNA6-6	*A. pacifica*	60S ribosomal protein L41	*Acartia pacifica*	KC989864	0
Apa_cDNA67	*A. pacifica*	GTP-binding nuclear protein Ran	*Penaeus monodon*	AEO23963	2.49E-136
Apa_cDNA6-7	*A. pacifica*	60S ribosomal protein L41	*Bombyx mori*	NM_001042449	4.00E-24
Apa_cDNA68	*A. pacifica*	mitochondrial GTPase 1	*Danaus plexippus*	EHJ70245	4.76E-90
Apa_cDNA6-8	*A. pacifica*	60S ribosomal protein L41	*Nasonia vitripennis*	NM_001135907	1.00E-18
Apa_cDNA69	*A. pacifica*	aminoacyl tRNA synthase complex-interacting multifunctional protein 1-like	*Lepisosteus oculatus*	XP_006629966	5.62E-76
Apa_cDNA6-9	*A. pacifica*	60S ribosomal protein L41	*Bombyx mori*	NM_001042449	3.00E-14
Apa_cDNA70	*A. pacifica*	peroxiredoxin 1	*Ceratitis capitata*	XP_004536426	4.34E-19
Apa_cDNA71	*A. pacifica*	PREDICTED: LOW QUALITY PROTEIN: RING-box protein 2	*Tribolium castaneum*	XP_008201626	2.35E-50
Apa_cDNA7-1	*A. pacifica*	60S ribosomal protein L7a	*Lepeophtheirus salmonis*	ACO12925	1.50E-118
Apa_cDNA72	*A. pacifica*	cytochrome c oxidase polypeptide	*Tribolium castaneum*	EFA07589	2.30E-08
Apa_cDNA7-2	*A. pacifica*	60S ribosomal protein L7a	*Lepeophtheirus salmonis*	ACO12925	1.87E-117
Apa_cDNA73	*A. pacifica*	mushroom-body expressed, isoform G	*Drosophila melanogaster*	NP_730700	3.16E-40
Apa_cDNA7-3	*A. pacifica*	60S ribosomal protein L7a	*Lepeophtheirus salmonis*	ACO12925	5.68E-118
Apa_cDNA74	*A. pacifica*	hypothetical protein	*Lottia gigantea*	ESO83139	1.29E-31
Apa_cDNA75	*A. pacifica*	vacuolar protein sorting-associated protein 16-like protein	*Zootermopsis nevadensis*	KDR24089	0
Apa_cDNA76-1	*A. pacifica*	60S ribosomal protein L27a	*Lepeophtheirus salmonis*	ACO12227	1.13E-70
Apa_cDNA76-2	*A. pacifica*	60S ribosomal protein L27a	*Lepeophtheirus salmonis*	ACO12227	1.15E-68
Apa_cDNA77-1	*A. pacifica*	translocon-associated protein subunit delta	*Zootermopsis nevadensis*	KDR24467	3.04E-42
Apa_cDNA77-2	*A. pacifica*	translocon-associated protein subunit delta	*Zootermopsis nevadensis*	KDR24467	3.47E-42
Apa_cDNA77-3	*A. pacifica*	translocon-associated protein subunit delta	*Zootermopsis nevadensis*	KDR24467	8.80E-42
Apa_cDNA78-1	*A. pacifica*	60S ribosomal protein L39	*Lineus viridis*	ABZ04248	1.05E-17
Apa_cDNA78-2	*A. pacifica*	60S ribosomal protein L39	*Lineus viridis*	ABZ04248	1.05E-17
Apa_cDNA78-3	*A. pacifica*	60S ribosomal protein L39	*Lineus viridis*	ABZ04248	1.37E-17
Apa_Cdna79-1	*A. pacifica*	60S ribosomal protein L39	*Myotis brandtii*	XP_005859492	4.94E-20
Apa_Cdna79-2	*A. pacifica*	60S ribosomal protein L39	*Caligus rogercresseyi*	ACO10217	8.09E-12
Apa_Cdna79-3	*A. pacifica*	60S ribosomal protein L39	*Myotis brandtii*	XP_005859492	3.60E-12
Apa_cDNA80-1	*A. pacifica*	60S ribosomal protein L31	*Sipunculus nudus*	ABW90363	1.59E-53
Apa_cDNA80-2	*A. pacifica*	60S ribosomal protein L31	*Nematostella vectensis*	XP_001632549	1.07E-53
Apa_cDNA80-3	*A. pacifica*	60S ribosomal protein L31	*Chinchilla lanigera*	XP_005400816	6.10E-54
Apa_cDNA80-4	*A. pacifica*	60S ribosomal protein L31	*Chinchilla lanigera*	XP_005400816	9.40E-54
Apa_cDNA80-5	*A. pacifica*	60S ribosomal protein L31	*Chinchilla lanigera*	XP_005400816	3.98E-54

Apa_cDNA80-6	*A. pacifica*	60S ribosomal protein L31	*Chinchilla lanigera*	XP_005400816	3.98E-54
Apa_cDNA81	*A. pacifica*	MIT domain-containing protein 1	*Capitella teleta*	ELT94043	2.49E-82
Apa_cDNA8-1	*A. pacifica*	60S ribosomal protein L7	*Lepeophtheirus salmonis*	ACO12269	2.12E-131
Apa_cDNA8-2	*A. pacifica*	60S ribosomal protein L7	*Lepeophtheirus salmonis*	ACO12269	1.86E-131
Apa_cDNA82-1	*A. pacifica*	60S ribosomal protein L44	*Acartia pacifica*	AGN29651	3.36E-67
Apa_cDNA82-2	*A. pacifica*	60S ribosomal protein L44	*Acartia pacifica*	AGN29651	4.66E-67
Apa_cDNA82-3	*A. pacifica*	60S ribosomal protein L44	*Acartia pacifica*	AGN29651	3.36E-67
Apa_cDNA83	*A. pacifica*	translocon-associated protein subunit gamma	*Bombyx mori*	NP_001040330	1.22E-74
Apa_cDNA8-3	*A. pacifica*	60S ribosomal protein L7	*Lepeophtheirus salmonis*	ACO12269	4.76E-130
Apa_cDNA84-1	*A. pacifica*	proteasome subunit beta type-5	*Daphnia pulex*	EFX80663	3.80E-129
Apa_cDNA84-2	*A. pacifica*	proteasome subunit beta type-5	*Daphnia pulex*	EFX80663	8.08E-131
Apa_cDNA85-1	*A. pacifica*	60S ribosomal protein L22	*Acartia pacifica*	AGN29654	1.30E-46
Apa_cDNA85-2	*A. pacifica*	60S ribosomal protein L22	*Acartia pacifica*	AGN29654	3.40E-47
Apa_cDNA86-1	*A. pacifica*	40S ribosomal protein S5	*Lysiphlebus testaceipes*	AAX62467	2.45E-114
Apa_cDNA86-2	*A. pacifica*	40S ribosomal protein S5	*Lysiphlebus testaceipes*	AAX62424	1.61E-114
Apa_cDNA87	*A. pacifica*	40S ribosomal protein S5	*Acyrthosiphon pisum*	XP_008184848	4.53E-105
Apa_cDNA88	*A. pacifica*	beta-carbonic anhydrase	*Daphnia pulex*	EFX79480	5.99E-97
Apa_cDNA89	*A. pacifica*	serine arginine-rich splicing factor 2		2LEA_A	8.65E-26
Apa_cDNA90	*A. pacifica*	leucine-rich repeat-containing protein 57	*Nasonia vitripennis*	XP_001601190	1.91E-67
Apa_cDNA91	*A. pacifica*	pten-like isoform B	*Drosophila ananassae*	XP_001955396	5.07E-81
Apa_cDNA9-1	*A. pacifica*	40S ribosomal protein S29	*Acartia pacifica*	AGN29639	1.26E-32
Apa_cDNA92	*A. pacifica*	LYR motif-containing protein 5	*Saccoglossus kowalevskii*	XP_002738948	1.83E-30
Apa_cDNA9-2	*A. pacifica*	40S ribosomal protein S29	*Acartia pacifica*	AGN29639	2.39E-32
Apa_cDNA93	*A. pacifica*	barrier-to-autointegration factor	*Caligus rogercresseyi*	ACO11308	9.81E-45
Apa_cDNA9-3	*A. pacifica*	40S ribosomal protein S29	*Acartia pacifica*	AGN29639	1.80E-32
Apa_cDNA94	*A. pacifica*	beta-carbonic anhydrase	*Daphnia pulex*	EFX79480	5.99E-97
Apa_cDNA9-4	*A. pacifica*	unknown protein			
Apa_cDNA95	*A. pacifica*	40S ribosomal protein S3	*Zootermopsis nevadensis*	KDR16813	1.14E-139
Apa_cDNA9-5	*A. pacifica*	40S ribosomal protein S29	*Acartia pacifica*	AGN29639	1.17E-32
Apa_cDNA9-6	*A. pacifica*	40S ribosomal protein S29	*Acartia pacifica*	AGN29639	1.12E-32
Apa_cDNA96-1	*A. pacifica*	60S ribosomal protein L37	*Acartia pacifica*	AGN29663	1.05E-45
Apa_cDNA96-2	*A. pacifica*	60S ribosomal protein L37	*Acartia pacifica*	AGN29663	1.31E-47
Apa_cDNA96-3	*A. pacifica*	60S ribosomal protein L37	*Acartia pacifica*	AGN29663	1.05E-45
Apa_cDNA96-4	*A. pacifica*	60S ribosomal protein L37	*Acartia pacifica*	AGN29663	1.03E-45
Apa_cDNA96-5	*A. pacifica*	60S ribosomal protein L37	*Acartia pacifica*	AGN29663	1.05E-45
Apa_cDNA96-6	*A. pacifica*	60S ribosomal protein L37	*Acartia pacifica*	AGN29663	1.92E-45
Apa_cDNA9-7	*A. pacifica*	40S ribosomal protein S29	*Acartia pacifica*	AGN29639	5.98E-32
Apa_cDNA97-1	*A. pacifica*	60S ribosomal protein L37a	*Lepeophtheirus salmonis*	ADD38662	1.23E-49
Apa_cDNA97-2	*A. pacifica*	60S ribosomal protein L37a	*Lepeophtheirus salmonis*	ADD38662	1.26E-49
Apa_cDNA97-3	*A. pacifica*	60S ribosomal protein L37a	*Lepeophtheirus salmonis*	ADD38662	1.53E-49
Apa_cDNA97-4	*A. pacifica*	60S ribosomal protein L37a	*Lepeophtheirus salmonis*	ADD38662	1.58E-49
Apa_cDNA9-8	*A. pacifica*	40S ribosomal protein S29	*Acartia pacifica*	AGN29639	1.12E-32
Apa_cDNA98-1	*A. pacifica*	60S ribosomal protein L36	*Caligus rogercresseyi*	ACO11656	5.29E-39
Apa_cDNA98-2	*A. pacifica*	60S ribosomal protein L36	*Caligus rogercresseyi*	ACO11656	1.57E-38
Apa_cDNA98-3	*A. pacifica*	60S ribosomal protein L36	*Caligus rogercresseyi*	ACO11656	1.86E-38

Apa_cDNA98-4	*A. pacifica*	60S ribosomal protein L36	*Caligus rogercresseyi*	ACO11656	5.32E-39
CalsicDNA1(Csi1)	*Calanus sinicus*	myosin light chain 2	*Blattella germanica*	ABD47458	8.16E-42
CalsicDNA10(Csi10)	*C. sinicus*	60S acidic ribosomal protein P1	*Ixodes scapularis*	XP_002435967	1.07E-19
CalsicDNA13(Csi13)	*C. sinicus*	60S ribosomal protein L29	*Koerneria sp. RS1982*	ABR87315	1.25E-18
CalsicDNA15(Csi15)	*C. sinicus*	60S ribosomal protein L41	*Macaca mulatta*	XP_002804534.1	3.30E+00
CalsicDNA17(Csi17)	*C. sinicus*	60S ribosomal protein L19	*Diachasmimorpha longicaudata*	AHN65139	5.29E-99
CalsicDNA18(Csi18)	*C. sinicus*	60S ribosomal protein L12	*Scarabaeus laticollis*	CAJ17266	1.77E-91
CalsicDNA19(Csi19)	*C. sinicus*	ubiquitin-60S ribosomal protein L40	*Sarcophilus harrisii*	XP_003760822	4.56E-82
CalsicDNA22(Csi22)	*C. sinicus*	40S ribosomal protein S29	*Acartia pacifica*	AGN29639	8.73E-30
CalsicDNA23(Csi23)	*C. sinicus*	60S ribosomal protein L23	*Acartia pacifica*	AGN29657	7.71E-79
CalsicDNA24(Csi24)	*C. sinicus*	60S ribosomal protein L41	*Macaca mulatta*	XP_002804534.1	3.40E+00
CalsicDNA25(Csi25)	*C. sinicus*	60S ribosomal protein L37a	*Lepeophtheirus salmonis*	ADD38662	4.01E-39
CalsicDNA29(Csi29)	*C. sinicus*	myosin light chain 2	*Blattella germanica*	ABD47458	2.00E-41
CalsicDNA3(Csi3)	*C. sinicus*	myosin light chain 2	*Blattella germanica*	ABD47458	1.65E-41
CalsicDNA30(Csi30)	*C. sinicus*	40S ribosomal protein S13	*Latimeria chalumnae*	XP_005990576	2.54E-93
CalsicDNA34(Csi34)	*C. sinicus*	profilin	*Pseudodiaptomus annandalei*	AGT28477	5.41E-48
CalsicDNA36(Csi36)	*C. sinicus*	40S ribosomal protein S10	*Tribolium castaneum*	XP_967221	3.29E-72
CalsicDNA37(Csi37)	*C. sinicus*	60S ribosomal protein L34	*Caligus clemensi*	ACO14672	7.59E-55
CalsicDNA38(Csi38)	*C. sinicus*	40S ribosomal protein S17	*Caligus rogercresseyi*	ACO10326	2.26E-69
CalsicDNA39(Csi39)	*C. sinicus*	isoform A	*Bactrocera oleae*	ADQ64580	2.54E-19
CalsicDNA41(Csi41)	*C. sinicus*	60S acidic ribosomal protein P1	*Ixodes scapularis*	XP_002435967	1.44E-19
CalsicDNA43(Csi43)	*C. sinicus*	60S ribosomal protein L37	*Acartia pacifica*	AGN29663	1.02E-36
CalsicDNA44(Csi44)	*C. sinicus*	cytochrome c oxidase subunit 5B, mitochondrial precursor	*Caligus rogercresseyi*	ACO10361	2.59E-42
CalsicDNA45(Csi45)	*C. sinicus*	unknown protein			
CalsicDNA46(Csi46)	*C. sinicus*	60S ribosomal protein L29	*Koerneria sp. RS1982*	ABR87315	1.25E-18
CalsicDNA47(Csi47)	*C. sinicus*	60S acidic ribosomal protein P2	*Aureobasidium melanogenum CBS 110374*	KEQ61663	6.03E-14
CalsicDNA48(Csi48)	*C. sinicus*	ADP-ribosylation factor-like protein 2-like	*Saccoglossus kowalevskii*	XP_002738749	1.16E-93
CalsicDNA49(Csi49)	*C. sinicus*	mitochondrial import inner membrane translocase subunit TIM10	*Caligus clemensi*	ACO14987	4.91E-42

CalsicDNA50(Csi50)	*C. sinicus*	60S ribosomal protein L10	*Caligus clemensi*	ACO14837	5.03E-115
CalsicDNA51(Csi51)	*C. sinicus*	60S ribosomal protein L19	*Diachasmimorpha longicaudata*	AHN65139	5.29E-99
CalsicDNA52(Csi52)	*C. sinicus*	unknown protein			
CalsicDNA53(Csi53)	*C. sinicus*	60S ribosomal protein L37	*Acartia pacifica*	AGN29663	1.77E-38
CalsicDNA54(Csi54)	*C. sinicus*	unknown protein			
CalsicDNA55(Csi55)	*C. sinicus*	60S ribosomal protein L37	*Acartia pacifica*	AGN29663	1.77E-38
CalsicDNA57(Csi57)	*C. sinicus*	up-regulated during skeletal muscle growth protein 5	*Zootermopsis nevadensis*	KDR11975	4.03E-06
CalsicDNA59(Csi59)	*C. sinicus*	NADH dehydrogenase	*Aedes aegypti*	XP_001658796	1.81E-17
CalsicDNA60(Csi60)	*C. sinicus*	unknown protein			
CalsicDNA61(Csi61)	*C. sinicus*	unknown protein			
CalsicDNA63(Csi63)	*C. sinicus*	unknown protein			
CalsicDNA64(Csi64)	*C. sinicus*	cytochrome c	*Ictidomys tridecemlineatus*	XP_005324707	1.99E-51
CendocDNA10	*Centropages dorsispinatus*	60S ribosomal protein L27a	*Lepeophtheirus salmonis*	ACO12227	3.77E-68
CendocDNA12	*C. dorsispinatus*	40S ribosomal protein S16	*Novocrania anomala*	ACD65104	7.15E-79
CendocDNA13	*C. dorsispinatus*	general transcription factor 3C polypeptide 1	*Nematostella vectensis*	XP_001634768	5.59E-11
CendocDNA14	*C. dorsispinatus*	40S ribosomal protein S15	*Barentsia elongata*	ABW90420	3.26E-73
CendocDNA17	*C. dorsispinatus*	40S ribosomal protein S15	*Barentsia elongata*	ABW90420	3.26E-73
CendocDNA19	*C. dorsispinatus*	unknown protein			
CendocDNA2	*C. dorsispinatus*	40S ribosomal protein S13	*Branchiostoma belcheri*	AAN52387	3.81E-94
CendocDNA20	*C. dorsispinatus*	60S ribosomal protein L35a	*Lepeophtheirus salmonis*	ACO12970	4.00E-57
CendocDNA22	*C. dorsispinatus*	mitotic-spindle organizing protein 1	*Saccoglossus kowalevskii*	XP_002733331	6.07E-22
CendocDNA23	*C. dorsispinatus*	Transcription initiation factor IIA subunit 2	*Bombyx mori*	NP_001040463	1.78E-13
CendocDNA24	*C. dorsispinatus*	60S acidic ribosomal protein P1	*Ixodes scapularis*	XP_002435967	8.09E-20
CendocDNA25	*C. dorsispinatus*	unknown protein			
CendocDNA26	*C. dorsispinatus*	40S ribosomal protein S28	*Culex quinquefasciatus*	XP_001865978	2.00E-23
CendocDNA28	*C. dorsispinatus*	unknown protein			
CendocDNA29	*C. dorsispinatus*	40S ribosomal protein S23	*Lepeophtheirus salmonis*	ACO12024	4.54E-91
CendocDNA3	*C. dorsispinatus*	CendocDNA3	*60S ribosomal protein L8*	Acartia pacifica	AGN29659

CendocDNA31	*C. dorsispinatus*	60S ribosomal protein L32	*Camponotus floridanus*	EFN68969	1.99E-74
CendocDNA32	*C. dorsispinatus*	Death-associated protein 1	*Lepeophtheirus salmonis*	ADD24224	1.92E-15
CendocDNA33	*C. dorsispinatus*	60S ribosomal protein L10	*Caligus clemensi*	ACO14837	3.71E-131
CendocDNA35	*C. dorsispinatus*	GTP-binding protein Rheb-like isoform X1	*Acyrthosiphon pisum*	XP_008179467	2.45E-75
CendocDNA36	*C. dorsispinatus*	40S ribosomal protein S23	*Lepeophtheirus salmonis*	ACO12024	4.54E-91
CendocDNA39	*C. dorsispinatus*	60S ribosomal protein L44	*Acartia pacifica*	AGN29651	5.97E-62
CendocDNA4	*C. dorsispinatus*	unknown protein			
CendocDNA40	*C. dorsispinatus*	60S ribosomal protein L18a	*Caligus clemensi*	ACO14776	5.69E-89
CendocDNA41	*C. dorsispinatus*	60S ribosomal protein L12	*Culex quinquefasciatus*	XP_001865531	2.90E-85
CendocDNA45	*C. dorsispinatus*	macrophage mannose receptor 1-like isoform X2	*Erinaceus europaeus*	XP_007532909	3.89E-07
CendocDNA46	*C. dorsispinatus*	60S ribosomal protein L11	*Tribolium castaneum*	XP_971759	1.69E-109
CendocDNA48	*C. dorsispinatus*	60S ribosomal protein L39	*Saprolegnia diclina VS20*	XP_008614346	5.96E-20
CendocDNA49	*C. dorsispinatus*	60S ribosomal protein L9	*Acartia pacifica*	AGN29667	1.10E-104
CendocDNA5	*C. dorsispinatus*	unknown protein			
CendocDNA51	*C. dorsispinatus*	40S ribosomal protein S3a	*Lepeophtheirus salmonis*	ACO12723	5.91E-140
CendocDNA52	*C. dorsispinatus*	60S acidic ribosomal protein P2	*Drosophila melanogaster*	ACK77644	7.26E-14
CendocDNA8	*C. dorsispinatus*	unknown protein			
CentecDNA1	*Centropages tenuiremis*	60S acidic ribosomal protein P1	*Ixodes scapularis*	XP_002435967	3.64E-18
CentecDNA10	*C. tenuiremis*	60S ribosomal protein L35a	*Lepeophtheirus salmonis*	ACO12970	1.92E-51
CentecDNA12	*C. tenuiremis*	NADH dehydrogenase	*ubiquinone*	XP_001512375	2.26E-33
CentecDNA14	*C. tenuiremis*	60S acidic ribosomal protein P2	*Oikopleura dioica*	CBY33355	5.28E-12
CentecDNA15	*C. tenuiremis*	40S ribosomal protein S10	*Acartia pacifica*	AGN29661	2.00E-62
CentecDNA16	*C. tenuiremis*	60S ribosomal protein L37	*Acartia pacifica*	AGN29663	6.92E-44
CentecDNA17	*C. tenuiremis*	60S ribosomal protein L10	*Caligus clemensi*	ACO14837	1.31E-133
CentecDNA18	*C. tenuiremis*	cytochrome c	*Spodoptera litura*	AFS31125	7.70E-51
CentecDNA19	*C. tenuiremis*	unknown protein			
CentecDNA20	*C. tenuiremis*	60S acidic ribosomal protein P1	*Ixodes scapularis*	XP_002435967	3.55E-18
CentecDNA21	*C. tenuiremis*	translocon-associated protein subunit gamma	*Riptortus pedestris*	BAN20565	8.55E-74
CentecDNA24	*C. tenuiremis*	40S ribosomal protein S5	*Acyrthosiphon pisum*	XP_008184848	3.56E-122
CentecDNA25	*C. tenuiremis*	60S acidic ribosomal protein P2	*Oikopleura dioica*	CBY33355	1.23E-12
CentecDNA26	*C. tenuiremis*	60S ribosomal protein L40, isoform A	*Drosophila melanogaster*	NP_476776	1.41E-84
CentecDNA27	*C. tenuiremis*	cytochrome c oxidase subunit 5B, mitochondrial precursor	*Lepeophtheirus salmonis*	ACO12623	6.40E-45

CentecDNA29	*C. tenuiremis*	40S ribosomal protein S16	*Zootermopsis nevadensis*	KDR22490	4.98E-82
CentecDNA3	*C. tenuiremis*	60S ribosomal protein L35a	*Lepeophtheirus salmonis*	ACO12970	2.38E-53
CentecDNA30	*C. tenuiremis*	60S ribosomal protein L37	*Acartia pacifica*	AGN29663	6.67E-44
CentecDNA31	*C. tenuiremis*	60S ribosomal protein L23	*Acartia pacifica*	AGN29657	5.86E-92
CentecDNA32	*C. tenuiremis*	60S ribosomal protein L32	*Bombus terrestris*	XP_003398978	1.50E-64
CentecDNA33	*C. tenuiremis*	protein translation factor SUI1 homolog	*Acyrthosiphon pisum*	XP_001948896	1.56E-57
CentecDNA34	*C. tenuiremis*	60S ribosomal protein L22	*Acartia pacifica*	AGN29654	6.19E-43
CentecDNA36	*C. tenuiremis*	40S ribosomal protein S21	*Acartia pacifica*	AGN29649	1.08E-48
CentecDNA37	*C. tenuiremis*	40S ribosomal protein S21	*Acartia pacifica*	AGN29649	1.30E-48
CentecDNA38	*C. tenuiremis*	CentecDNA38	*proactivator polypeptide*	Ixodes scapularis	XP_002412 058
CentecDNA39	*C. tenuiremis*	40S ribosomal protein S21	*Acartia pacifica*	AGN29649	1.15E-48
CentecDNA4	*C. tenuiremis*	60S ribosomal protein L37	*Acartia pacifica*	AGN29663	3.34E-43
CentecDNA40	*C. tenuiremis*	60S ribosomal protein L11	*Tribolium castaneum*	XP_971759	6.25E-107
CentecDNA41	*C. tenuiremis*	40S ribosomal protein S3	*Haplochromis burtoni*	XP_005938027	2.20E-148
CentecDNA46	*C. tenuiremis*	ATP synthase lipid-binding protein, mitochondrial precursor	*Caligus rogercresseyi*	ACO10993	4.99E-20
CentecDNA47	*C. tenuiremis*	60S ribosomal protein L11	*Drosophila willistoni*	XP_002063810	3.52E-93
CentecDNA49	*C. tenuiremis*	40S ribosomal protein S18	*Caligus rogercresseyi*	ACO11045	1.34E-76
CentecDNA5	*C. tenuiremis*	60S acidic ribosomal protein P2	*Oikopleura dioica*	CBY33355	1.26E-12
CentecDNA50	*C. tenuiremis*	up-regulated during skeletal muscle growth protein 5	*Zootermopsis nevadensis*	KDR11975	6.59E-06
CentecDNA51	*C. tenuiremis*	60S ribosomal protein L12	*Anopheles sinensis*	KFB44942	9.40E-68
CentecDNA53	*C. tenuiremis*	40S ribosomal protein S21	*Acartia pacifica*	AGN29649	1.30E-48
CentecDNA54	*C. tenuiremis*	translocon-associated protein subunit gamma	*Riptortus pedestris*	BAN20565	7.48E-74
CentecDNA55	*C. tenuiremis*	60S acidic ribosomal protein P1	*Ixodes scapularis*	XP_002435967	3.64E-18
CentecDNA56	*C. tenuiremis*	CentecDNA56	*PIN2/TERF1-interacting telomerase inhibitor 1*	Daphnia pulex	EFX84088
CentecDNA57	*C. tenuiremis*	40S ribosomal protein S9	*Acyrthosiphon pisum*	XP_001945527	7.14E-103
CentecDNA58	*C. tenuiremis*	60S ribosomal protein L37a	*Lepeophtheirus salmonis*	ADD38662	4.13E-41
CentecDNA59	*C. tenuiremis*	60S ribosomal protein L35a	*Lepeophtheirus salmonis*	ACO12970	2.38E-53
CentecDNA6	*C. tenuiremis*	60S ribosomal protein L26	*Zootermopsis nevadensis*	KDR23279	4.18E-78
CentecDNA60	*C. tenuiremis*	60S ribosomal protein L38	*Apis mellifera*	XP_001120263	2.51E-37
CentecDNA61	*C. tenuiremis*	40S ribosomal protein S8	*Lepeophtheirus salmonis*	ACO12033	1.97E-116
CentecDNA62	*C. tenuiremis*	40S ribosomal protein S27	*Acartia pacifica*	AGN29666	3.65E-52
CentecDNA63	*C. tenuiremis*	40S ribosomal protein S19	*Daphnia pulex*	EFX64730	1.86E-69
CentecDNA64	*C. tenuiremis*	40S ribosomal protein S27	*Acartia pacifica*	AGN29666	3.52E-52
CentecDNA65	*C. tenuiremis*	40S ribosomal protein S12	*Daphnia pulex*	EFX90257	2.89E-65
CentecDNA66	*C. tenuiremis*	40S ribosomal protein S4	*Lepeophtheirus salmonis*	ACO12708	1.37E-145
CentecDNA67	*C. tenuiremis*	60S ribosomal protein L24	*Tribolium castaneum*	XP_966448	7.69E-42
CentecDNA68	*C. tenuiremis*	CKLF-like MARVEL transmembrane domain-containing protein 4-like	*Caligus clemensi*	ACO15478	3.96E-38
CentecDNA69	*C. tenuiremis*	40S ribosomal protein S16	*Zootermopsis nevadensis*	KDR22490	4.98E-82
CentecDNA7	*C. tenuiremis*	60S ribosomal protein L36	*Lepeophtheirus salmonis*	ACO12212	3.15E-45
CentecDNA70	*C. tenuiremis*	60S ribosomal protein L17	*Caligus rogercresseyi*	ACO11567	7.23E-100
CentecDNA72	*C. tenuiremis*	eukaryotic translation initiation factor 5A	*Lepeophtheirus salmonis*	ACO12518	4.31E-63

CentecDNA73	*C. tenuiremis*	60S ribosomal protein L35a	*Lepeophtheirus salmonis*	ACO12970	6.73E-53
CentecDNA74	*C. tenuiremis*	60S ribosomal protein L38	*Apis mellifera*	XP_001120263	2.71E-37
CentecDNA75	*C. tenuiremis*	60S ribosomal protein L37a	*Lepeophtheirus salmonis*	ADD38662	1.26E-40
CentecDNA76	*C. tenuiremis*	cytochrome c oxidase subunit VIIA putative	*Scylla paramamosain*	ACY66414	8.00E-05
CentecDNA77	*C. tenuiremis*	60S ribosomal protein L18	*Nasonia vitripennis*	XP_008201914	2.53E-94
CentecDNA78	*C. tenuiremis*	60S ribosomal protein L14	*Lepeophtheirus salmonis*	ACO12382	2.66E-48
LabbicDNA10(Lbi10)	*Labidocera rotunda (previously L. bipinnata)*	40S ribosomal protein S25	*Acartia pacifica*	AGN29668	6.75E-40
LabbicDNA12(Lbi12)	*L. rotunda*	60S ribosomal protein L15	*Lepeophtheirus salmonis*	ACO12365	3.68E-115
LabbicDNA13(Lbi13)	*L. rotunda*	60S ribosomal protein L35a	*Lepeophtheirus salmonis*	ACO12970	1.05E-57
LabbicDNA14(Lbi14)	*L. rotunda*	60S ribosomal protein L37a	*Lepeophtheirus salmonis*	ADD38662	8.99E-42
LabbicDNA15(Lbi15)	*L. rotunda*	60S ribosomal protein L19	*Cerapachys biroi*	EZA54050	4.51E-85
LabbicDNA16(Lbi16)	*L. rotunda*	dolichyl-diphosphooligosaccharide--protein glycosyltransferase subunit DAD1-like	*Apis dorsata*	XP_006624759	7.83E-59
LabbicDNA18(Lbi18)no	*L. rotunda*	unknown protein			
LabbicDNA2(Lbi2)	*L. rotunda*	60S acidic ribosomal protein P2	*Lepeophtheirus salmonis*	ACO12871	2.18E-17
LabbicDNA21(Lbi21)	*L. rotunda*	40S ribosomal protein S21	*Acartia pacifica*	AGN29649	6.47E-50
LabbicDNA23(Lbi23)?13	*L. rotunda*	60S ribosomal protein L35a	*Lepeophtheirus salmonis*	ACO12970	2.30E-53
LabbicDNA25(Lbi25)	*L. rotunda*	60S acidic ribosomal protein P1	*Diaphorina citri*	XP_008479537	3.19E-25
LabbicDNA3(Lbi3)	*L. rotunda*	LabbicDNA3(Lbi3)	*Na(+)/H(+) exchange regulatory cofactor NHE-RF2*	Zootermopsis nevadensis	KDR09705
LabbicDNA30(Lbi30)	*L. rotunda*	cytochrome c oxidase subunit 6C-1-like isoform X1	*Ceratitis capitata*	XP_004524711	1.35E-18
LabbicDNA32(Lbi32)	*L. rotunda*	40S ribosomal protein S13	*Xenopus laevis*	NP_001080351	2.10E-94
LabbicDNA33(Lbi33)	*L. rotunda*	eukaryotic translation initiation factor 3 subunit F-1-like	*Lepeophtheirus salmonis*	ADD24199	1.34E-114
LabbicDNA34(Lbi34)	*L. rotunda*	60S ribosomal protein L29	*Lepeophtheirus salmonis*	ADD24190	7.24E-19
LabbicDNA35(Lbi35)	*L. rotunda*	60S ribosomal protein L9	*Acartia pacifica*	AGN29667	9.59E-107
LabbicDNA36(Lbi36)	*L. rotunda*	eukaryotic translation initiation factor 3 subunit K	*Lepeophtheirus salmonis*	ACO12922	4.70E-88
LabbicDNA37(Lbi37)	*L. rotunda*	40S ribosomal protein S29	*Acartia pacifica*	AGN29639	1.02E-29
LabbicDNA39(Lbi39)	*L. rotunda*	39S ribosomal protein L54, mitochondrial	*Drosophila virilis*	XP_002050958	2.75E-15
LabbicDNA4(Lbi4)	*L. rotunda*	60S ribosomal protein L23	*Acartia pacifica*	AGN29657	1.62E-91

LabbicDNA40(Lbi40)	*L. rotunda*	60S ribosomal protein L35a	*Lepeophtheirus salmonis*	ACO12970	4.76E-52
LabbicDNA41(Lbi41)	*L. rotunda*	40S ribosomal protein S23	*Lepeophtheirus salmonis*	ACO12024	9.37E-92
LabbicDNA42(Lbi42)	*L. rotunda*	40S ribosomal protein S25	*Acartia pacifica*	AGN29668	2.20E-40
LabbicDNA43(Lbi43)	*L. rotunda*	60S ribosomal protein L14	*Papilio polytes*	BAM18938	5.06E-45
LabbicDNA44(Lbi44)	*L. rotunda*	40S ribosomal protein S18	*Caligus rogercresseyi*	ACO11045	1.36E-80
LabbicDNA45(Lbi45)	*L. rotunda*	40S ribosomal protein S12	*Tribolium castaneum*	EFA06382	2.09E-68
LabbicDNA47(Lbi47)	*L. rotunda*	14-3-3 zeta	*Acartia pacifica*	AGN29628	2.36E-160
LabbicDNA49(Lbi49)	*L. rotunda*	40S ribosomal protein S17	*Caligus rogercresseyi*	ACO10326	1.01E-66
LabbicDNA50(Lbi50)	*L. rotunda*	60S ribosomal protein L27	*Latrodectus hesperus*	ADV40079	8.90E-50
LabbicDNA51(Lbi51)	*L. rotunda*	60S ribosomal protein L22	*Acartia pacifica*	AGN29654	4.73E-42
LabbicDNA52(Lbi52)	*L. rotunda*	60S ribosomal protein L27	*Latrodectus hesperus*	ADV40079	3.43E-49
LabbicDNA53(Lbi53)	*L. rotunda*	LabbicDNA53(Lbi53)	*DNA polymerase epsilon subunit 3*	Lepeophtheirus salmonis	ADD38397
LabbicDNA7(Lbi7)	*L. rotunda*	cytochrome b-c1 complex subunit 9	*Caligus rogercresseyi*	ACO10297	3.31E-16
LabbicDNA9(Lbi9)	*L. rotunda*	40S ribosomal protein S18	*Caligus rogercresseyi*	ACO11045	1.25E-80
ParpacDNA1	*Paracalanus parvus*	60S ribosomal protein L26	*Curculio glandium*	CAJ17322	5.66E-79
ParpacDNA10(Ppa10)	*P. parvus*	SWIB domain-containing protein	*Tigriopus japonicus*	ACS36127	8.61E-18
ParpacDNA11(Ppa11)	*P. parvus*	isoform A	*Culicoides sonorensis*	AAU06505	8.14E-08
ParpacDNA12(Ppa12)	*P. parvus*	selenium-dependent glutathione peroxidase	*Sepiella maindroni*	AEK48346	1.57E-76
ParpacDNA13(Ppa13)	*P. parvus*	40S ribosomal protein S28	*Megachile rotundata*	XP_003700807	7.48E-23
ParpacDNA14(Ppa14)	*P. parvus*	peroxiredoxin 6	*Saccoglossus kowalevskii*	XP_002733706	5.01E-113
ParpacDNA16	*P. parvus*	ParpacDNA16	*lamin B receptor*	Xenopus laevis	NP_001079301
ParpacDNA17(Ppa17)	*P. parvus*	40S ribosomal protein S25	*Acartia pacifica*	AGN29668	2.69E-30
ParpacDNA18	*P. parvus*	FKBP-type peptidyl-prolyl cis-trans isomerase-12	*Ancylostoma ceylanicum*	EYB97065	2.90E-48
ParpacDNA19	*P. parvus*	small ubiquitin-related modifier-like	*Caligus rogercresseyi*	ACO11701	6.46E-46
ParpacDNA2(Ppa2)	*P. parvus*	ATP synthase F(0) complex subunit C3, mitochondrial-like	*Lepisosteus oculatus*	XP_006636561	2.33E-17
ParpacDNA20(Ppa20)	*P. parvus*	unknown protein			
ParpacDNA21(Ppa21)	*P. parvus*	SWIB domain-containing protein	*Tigriopus japonicus*	ACS36127	8.77E-20
ParpacDNA22(Ppa22)	*P. parvus*	SWIB domain-containing protein	*Tigriopus japonicus*	ACS36127	8.53E-20

ParpacDNA24(Ppa24)	*P. parvus*	cytochrome c oxidase subunit 6C-1-like isoform X1	*Ceratitis capitata*	XP_004524711	1.37E-21
ParpacDNA26	*P. parvus*	ubiquinol-cytochrome c reductase binding protein	*Acyrthosiphon pisum*	NP_001155372	3.04E-33
ParpacDNA28	*P. parvus*	unknown protein			
ParpacDNA29(Ppa29)	*P. parvus*	unknown protein			
ParpacDNA3	*P. parvus*	NEB- isoform A	*Drosophila virilis*	XP_002059507	9.91E-06
ParpacDNA30	*P. parvus*	60S ribosomal protein L34	*Caligus clemensi*	ACO14672	4.13E-48
ParpacDNA31(Ppa31)	*P. parvus*	60S ribosomal protein L19	*Nasonia vitripennis*	XP_001602817	5.80E-97
ParpacDNA33	*P. parvus*	U1 small nuclear ribonucleoprotein C, partial	*Pongo abelii*	XP_002816827	8.61E-33
ParpacDNA34	*P. parvus*	40S ribosomal protein S3	*Plecoglossus altivelis*	CBL52984	6.00E-154
ParpacDNA36(Ppa36)	*P. parvus*	ubiquitin carboxyl-terminal hydrolase 30	*Ascaris suum*	ERG87848	3.06E-08
ParpacDNA37(Ppa37)	*P. parvus*	40S ribosomal protein S28	*Megachile rotundata*	XP_003700807	3.90E-23
ParpacDNA38(Ppa38)	*P. parvus*	protein kish-A	*Capra hircus*	XP_005677140	1.64E-29
ParpacDNA4	*P. parvus*	selenium-dependent glutathione peroxidase	*Sepiella maindroni*	AEK48346	1.57E-76
ParpacDNA40(Ppa40)	*P. parvus*	U6 snRNA-associated Sm-like protein LSm1	*Lepeophtheirus salmonis*	ADD38614	1.67E-54
ParpacDNA41(Ppa41)	*P. parvus*	cytochrome c oxidase polypeptide	*Tribolium castaneum*	EFA07589	6.07E-12
ParpacDNA42	*P. parvus*	40S ribosomal protein S28	*Megachile rotundata*	XP_003700807	1.10E-22
ParpacDNA43(Ppa43)	*P. parvus*	NADH dehydrogenase	*Tetraodon nigroviridis*	CAF92313	7.93E-32
ParpacDNA44	*P. parvus*	40S ribosomal protein S15	*Lineus viridis*	ABZ04266	1.01E-65
ParpacDNA45	*P. parvus*	40S ribosomal protein S28	*Megachile rotundata*	XP_003700807	1.10E-22
ParpacDNA46	*P. parvus*	unknown protein			
ParpacDNA47(Ppa47)	*P. parvus*	nuclear protein p8	*Lepeophtheirus salmonis*	ABU41057	1.17E-17
ParpacDNA48	*P. parvus*	60S ribosomal protein L35	*Caligus rogercresseyi*	ACO10367	2.80E-59
ParpacDNA49	*P. parvus*	ubiquinol-cytochrome c reductase binding protein	*Acyrthosiphon pisum*	NP_001155372	3.04E-33
ParpacDNA5	*P. parvus*	unknown protein			
ParpacDNA50	*P. parvus*	60S ribosomal protein L35a	*Lepeophtheirus salmonis*	ACO12970	2.55E-55
ParpacDNA52	*P. parvus*	histone-lysine N-methyltransferase SMYD3 isoform X2	*Canis lupus familiaris*	XP_537223	4.61E-11
ParpacDNA53	*P. parvus*	60S ribosomal protein L39	*Dasypus novemcinctus*	XP_004482841	1.70E-23
ParpacDNA54(Ppa54)	*P. parvus*	unknown protein			
ParpacDNA55(Ppa55)	*P. parvus*	ribonuclease kappa	*Capitella teleta*	ELU15414	4.56E-11
ParpacDNA56	*P. parvus*	40S ribosomal protein S29	*Acartia pacifica*	AGN29639	3.11E-27
ParpacDNA57	*P. parvus*	60S ribosomal protein L31	*Bipolaris oryzae ATCC 44560*	XP_007686654	0.00011831
ParpacDNA58	*P. parvus*	ubiquitin-40S ribosomal protein S27a	*Novocrania anomala*	ACD65107	7.83E-69
ParpacDNA59	*P. parvus*	ATP synthase lipid-binding protein, mitochondrial precursor	*Pseudodiaptomus annandalei*	AGT28475	8.60E-21
ParpacDNA6	*P. parvus*	DNA-directed RNA polymerase	*Aedes aegypti*	XP_001658447	2.46E-75

ParpacDNA60(Ppa60)	*P. parvus*	small ubiquitin-related modifier-like	*Caligus rogercresseyi*	ACO11701	8.49E-46
ParpacDNA7(Ppa7)	*P. parvus*	protein TEX261	*Zootermopsis nevadensis*	KDR08618	7.42E-77
ParpacDNA8(Ppa8)	*P. parvus*	unknown protein			
ParpacDNA9(Ppa9)	*P. parvus*	ParpacDNA9(Ppa9)	*unknown protein*		
Ppo_cDNA	*Pseudodiaptomus poplesia*	Ppo_cDNA	*translation initiation factor eIF2B epsilon subunit?*	Batrachochytrium dendrobatidis JAM81	XP_006676013
Ppo_cDNA1(2)	*P. poplesia*	putative 26S proteasome complex subunit DSS1	*Homo sapiens*	P60896	0.00003
Ppo_cDNA10(2)	*P. poplesia*	RNA-binding protein squid	*Lepeophtheirus salmonis*	ACO11942	1.82E-46
Ppo_cDNA100	*P. poplesia*	eukaryotic translation initiation factor 3 subunit G	*Penaeus monodon*	ACO59906	1.18E-96
Ppo_cDNA101	*P. poplesia*	RNA-binding protein squid	*Lepeophtheirus salmonis*	ACO12371	5.19E-61
Ppo_cDNA102	*P. poplesia*	F-actin-capping protein subunit beta isoform X1	*Nasonia vitripennis*	XP_001607231	3.23E-162
Ppo_cDNA103	*P. poplesia*	peptidyl-prolyl cis-trans isomerase	*Riptortus pedestris*	BAN20396	3.80E-92
Ppo_cDNA104	*P. poplesia*	histone H2A.Z	*Lepeophtheirus salmonis*	ACO11883	2.38E-66
Ppo_cDNA105	*P. poplesia*	histone H3.3 isoform 1	*Ciona intestinalis*	XP_002131734	1.19E-80
Ppo_cDNA106	*P. poplesia*	succinate dehydrogenase	*Lepeophtheirus salmonis*	ACO12832	9.85E-44
Ppo_cDNA107	*P. poplesia*	succinate dehydrogenase cytochrome b560 subunit	*Oreochromis niloticus*	XP_003447506	1.86E-37
Ppo_cDNA108	*P. poplesia*	triosephosphate isomerase	*Lepeophtheirus salmonis*	ACO12903	2.36E-129
Ppo_cDNA109	*P. poplesia*	methylmalonate-semialdehyde dehydrogenase	*Lepeophtheirus salmonis*	ADD38034	0
Ppo_cDNA11(2)	*P. poplesia*	microsomal glutathione s-transferase	*Culex quinquefasciatus*	XP_001863047	3.11E-43
Ppo_cDNA110	*P. poplesia*	enolase	*Musca domestica*	XP_005179742	0
Ppo_cDNA111	*P. poplesia*	probable citrate synthase mitochondrial	*Lepeophtheirus salmonis*	ACO12464	0
Ppo_cDNA112	*P. poplesia*	ubiquinol-cytochrome C reductase	*Locusta migratoria manilensis*	ADC54384	5.43E-99
Ppo_cDNA113	*P. poplesia*	RNA-binding protein 3	*Lepeophtheirus salmonis*	ACO11756	1.33E-11
Ppo_cDNA114	*P. poplesia*	cyclin-dependent kinase 2 isoform X2	*Orycteropus afer afer*	XP_007953285	3.81E-134
Ppo_cDNA115	*P. poplesia*	phosphoethanolamine N-methyltransferase 3	*Arabidopsis thaliana*	AAG52075	1.71E-81
Ppo_cDNA116	*P. poplesia*	phospholipid-hydroperoxide glutathione peroxidase	*Rhipicephalus microplus*	ABA25916	3.74E-76
Ppo_cDNA1166	*P. poplesia*	Ppo_cDNA1166	*transcriptional regulator atrx homolog*	Zootermopsis nevadensis	KDR14479
Ppo_cDNA117	*P. poplesia*	gamma-glutamyl hydrolase	*Daphnia pulex*	EFX77408	4.87E-87
Ppo_cDNA118	*P. poplesia*	receptor expression-enhancing protein 5-like	*Ficedula albicollis*	XP_005060216	1.01E-60
Ppo_cDNA119	*P. poplesia*	Phosphate carrier protein, mitochondrial precursor	*Lepeophtheirus salmonis*	ACO12540	0
Ppo_cDNA12(2)	*P. poplesia*	ATP synthase subunit b, mitochondrial precursor	*Caligus clemensi*	ACO14593	1.23E-81

Ppo_cDNA120	*P. poplesia*	60S ribosomal protein L7a/NHP2-like protein 1	*Caligus rogercresseyi*	ACO10185	1.29E-62
Ppo_cDNA121	*P. poplesia*	PHD finger-like domain-containing protein 5A-like	*Neolamprologus brichardi*	XP_006807353	6.23E-68
Ppo_cDNA123	*P. poplesia*	serine proteinase stubble	*Cerapachys biroi*	EZA48555	6.72E-15
Ppo_cDNA124	*P. poplesia*	PREDICTED:?synaptotagmin 1 isoform X1?	*Nasonia vitripennis*	XP_008207924	3.97E-131
Ppo_cDNA125	*P. poplesia*	aspartate aminotransferase, mitochondrial precursor	*Caligus rogercresseyi*	ACO10233	0
Ppo_cDNA126-1(3)	*P. poplesia*	60S acidic ribosomal protein P2	*Dendroctonus ponderosae*	ERL95076	7.09E-17
Ppo_cDNA126-2	*P. poplesia*	60S acidic ribosomal protein P2	*Dendroctonus ponderosae*	ERL95076	8.52E-16
Ppo_cDNA127	*P. poplesia*	pneumococcal surface protein C	*Nematostella vectensis*	XP_001628416	4.92E-25
Ppo_cDNA128	*P. poplesia*	39S ribosomal protein L36, mitochondrial, putative	*Daphnia pulex*	EFX68183	1.17E-08
Ppo_cDNA129	*P. poplesia*	unknown protein			
Ppo_cDNA13(2)	*P. poplesia*	cytochrome b-c1 complex subunit 9	*Diaphorina citri*	XP_008468754	1.73E-17
Ppo_cDNA130	*P. poplesia*	simila to cg4702	*Microplitis demolitor*	XP_008543068	1.28E-08
Ppo_cDNA131-1(4)	*P. poplesia*	unknown protein			
Ppo_cDNA131-2	*P. poplesia*	unknown protein			
Ppo_cDNA131-3	*P. poplesia*	unknown protein			
Ppo_cDNA132(2)	*P. poplesia*	unknown protein			
Ppo_cDNA133(2)	*P. poplesia*	unknown protein			
Ppo_cDNA134	*P. poplesia*	unknown protein			
Ppo_cDNA135	*P. poplesia*	unknown protein			
Ppo_cDNA136	*P. poplesia*	unknown protein			
Ppo_cDNA137	*P. poplesia*	unknown protein			
Ppo_cDNA138	*P. poplesia*	unknown protein			
Ppo_cDNA139	*P. poplesia*	unknown protein			
Ppo_cDNA140	*P. poplesia*	unknown protein			
Ppo_cDNA141	*P. poplesia*	unknown protein			
Ppo_cDNA14-1	*P. poplesia*	ATP synthase lipid-binding protein, mitochondrial precursor	*Pseudodiaptomus annandalei*	AGT28475	1.57E-36
Ppo_cDNA142	*P. poplesia*	unknown protein			
Ppo_cDNA14-2(5)	*P. poplesia*	ATP synthase lipid-binding protein, mitochondrial precursor	*Caligus rogercresseyi*	ACO10993	4.35E-19
Ppo_cDNA143	*P. poplesia*	unknown protein			
Ppo_cDNA14-3	*P. poplesia*	ATP synthase lipid-binding protein, mitochondrial precursor	*Caligus rogercresseyi*	ACO10993	1.22E-18
Ppo_cDNA144	*P. poplesia*	unknown protein			
Ppo_cDNA145	*P. poplesia*	unknown protein			
Ppo_cDNA146	*P. poplesia*	unknown protein			
Ppo_cDNA147	*P. poplesia*	unknown protein			
Ppo_cDNA148	*P. poplesia*	unknown protein			
Ppo_cDNA149	*P. poplesia*	unknown protein			
Ppo_cDNA150	*P. poplesia*	unknown protein			
Ppo_cDNA151	*P. poplesia*	transcriptional activator protein pur-alpha	*Ovis aries*	NP_001009447	0.00097858
Ppo_cDNA15-1(2)	*P. poplesia*	40S ribosomal protein S3	*Zootermopsis nevadensis*	KDR16813	1.14E-140
Ppo_cDNA152	*P. poplesia*	unknown protein			

Ppo_cDNA15-2(2)	*P. poplesia*	40S ribosomal protein S3	*Zootermopsis nevadensis*	KDR16813	1.06E-140
Ppo_cDNA153	*P. poplesia*	unknown protein			
Ppo_cDNA154	*P. poplesia*	unknown protein			
Ppo_cDNA155	*P. poplesia*	unknown protein			
Ppo_cDNA156	*P. poplesia*	unknown protein			
Ppo_cDNA157	*P. poplesia*	unknown protein			
Ppo_cDNA158	*P. poplesia*	unknown protein			
Ppo_cDNA159	*P. poplesia*	RWD domain-containing protein 1	*Lepeophtheirus salmonis*	ACO12724	4.91E-34
Ppo_cDNA16	*P. poplesia*	40S ribosomal protein S5	*Timarcha balearica*	CAH04317	2.32E-120
Ppo_cDNA160	*P. poplesia*	paladin-like isoform X1	*Selaginella moellendorffii*	XP_002966937	7.70E-31
Ppo_cDNA161	*P. poplesia*	kunitz-type protease inhibitor 1-like	*Drosophila erecta*	XP_001968603	2.91E-07
Ppo_cDNA162	*P. poplesia*	unknown protein			
Ppo_cDNA163	*P. poplesia*	unknown protein			
Ppo_cDNA164	*P. poplesia*	Ppo_cDNA164	*PREDICTED: uncharacterized protein LOC100180415*	Ciona intestinalis	XP_002120607
Ppo_cDNA165	*P. poplesia*	Ppo_cDNA165	*unknown protein*		
Ppo_cDNA167	*P. poplesia*	Ppo_cDNA167	*succinate dehydrogenase*	Culex quinquefasciatus	XP_001847611
Ppo_cDNA168	*P. poplesia*	Ppo_cDNA168	*protein disulfide isomerase*	Litopenaeus vannamei	ACN89260
Ppo_cDNA169	*P. poplesia*	Ppo_cDNA169	*putative methyltransferase DDB_G0268948-like*	Chelonia mydas	XP_007054116
Ppo_cDNA17	*P. poplesia*	40S ribosomal protein S6	*Riptortus pedestris*	BAN20299	4.60E-120
Ppo_cDNA170	*P. poplesia*	Ppo_cDNA170	*prostaglandin reductase 1-like*	Papilio polytes	BAM20599
Ppo_cDNA171	*P. poplesia*	Ppo_cDNA171	*transitional endoplasmic reticulum ATPase*		3QQ7_A
Ppo_cDNA172	*P. poplesia*	Ppo_cDNA172	*acyl-coenzyme A thioesterase 13*	Fenneropenaeus chinensis	AEL79850
Ppo_cDNA173	*P. poplesia*	Ppo_cDNA173	*epsin-like protein*	Daphnia pulex	EFX85878
Ppo_cDNA18(3)	*P. poplesia*	40S ribosomal protein S7	*Acartia pacifica*	AGN29653	3.39E-116
Ppo_cDNA19	*P. poplesia*	40S ribosomal protein S8	*Bombyx mori*	NP_001037263	5.12E-110
Ppo_cDNA20(2)	*P. poplesia*	40S ribosomal protein S9	*Meladema coriacea*	CAH04322	3.77E-114
Ppo_cDNA2-1	*P. poplesia*	fructose-bisphosphate aldolase	*Lepeophtheirus salmonis*	ACO12340	0
Ppo_cDNA21(2)	*P. poplesia*	40S ribosomal protein S10	*Xenopsylla cheopis*	ABM55456	3.51E-54
Ppo_cDNA22	*P. poplesia*	40S ribosomal protein S11	*Acartia pacifica*	AGN29665	7.69E-95
Ppo_cDNA23	*P. poplesia*	40S ribosomal protein S12	*Harpegnathos saltator*	EFN80764	2.58E-64
Ppo_cDNA2-3	*P. poplesia*	fructose-bisphosphate aldolase	*Caligus clemensi*	ACO15303	4.77E-114
Ppo_cDNA24(5)	*P. poplesia*	40S ribosomal protein S14	*Caligus clemensi*	ACO14952	8.08E-86
Ppo_cDNA25(3)	*P. poplesia*	40S ribosomal protein S15	*Capitella teleta*	ELU08552	7.26E-60
Ppo_cDNA26-1	*P. poplesia*	40S ribosomal protein S15a	*Acartia pacifica*	AGN29647	4.44E-87
Ppo_cDNA26-2(3)	*P. poplesia*	40S ribosomal protein S16a	*Acartia pacifica*	AGN29647	4.62E-87
Ppo_cDNA27(2)	*P. poplesia*	40S ribosomal protein S16	*Xenopsylla cheopis*	ABM55425	8.68E-82
Ppo_cDNA28-1(4)	*P. poplesia*	40S ribosomal protein S17	*Caligus clemensi*	ACO14607	1.69E-67
Ppo_cDNA29(2)	*P. poplesia*	40S ribosomal protein S18	*Caligus rogercresseyi*	ACO11045	2.35E-76
Ppo_cDNA30	*P. poplesia*	40S ribosomal protein S19	*Lineus viridis*	ABZ04268	9.77E-70

Ppo_cDNA3-1(3)	*P. poplesia*	ATP synthase subunit g, mitochondrial	*Helobdella robusta*	ESN96292	5.10E-17
Ppo_cDNA31(4)	*P. poplesia*	40S ribosomal protein S21	*Acartia pacifica*	AGN29649	8.55E-45
Ppo_cDNA3-2	*P. poplesia*	ATP synthase subunit g, mitochondrial	*Palaemon varians*	ACR54103	2.41E-16
Ppo_cDNA32-1(6)	*P. poplesia*	40S ribosomal protein S23	*Lepeophtheirus salmonis*	ACO12024	1.29E-90
Ppo_cDNA32-2(3)	*P. poplesia*	40S ribosomal protein S23	*Lepeophtheirus salmonis*	ACO12024	1.20E-90
Ppo_cDNA3-3	*P. poplesia*	ATP synthase subunit g, mitochondrial	*Palaemon varians*	ACR54103	2.11E-25
Ppo_cDNA33-1(2)	*P. poplesia*	40S ribosomal protein S24	*Lepeophtheirus salmonis*	ACO12534	2.83E-49
Ppo_cDNA33-2	*P. poplesia*	40S ribosomal protein S24	*Lepeophtheirus salmonis*	ACO12534	3.72E-49
Ppo_cDNA34(2)	*P. poplesia*	40S ribosomal protein S25	*Acartia pacifica*	AGN29668	1.49E-33
Ppo_cDNA35(3)	*P. poplesia*	40S ribosomal protein S26	*Lepeophtheirus salmonis*	ACO12442	3.68E-47
Ppo_cDNA36(2)	*P. poplesia*	ubiquitin-40S ribosomal protein S27a	*Saccoglossus kowalevskii*	XP_002733357	2.90E-75
Ppo_cDNA37-1	*P. poplesia*	40S ribosomal protein S28	*Megachile rotundata*	XP_003700807	1.49E-24
Ppo_cDNA37-2(2)	*P. poplesia*	40S ribosomal protein S28	*Megachile rotundata*	XP_003700807	2.15E-24
Ppo_cDNA38(3)	*P. poplesia*	40S ribosomal protein S29	*Acartia pacifica*	AGN29639	5.96E-32
Ppo_cDNA39(2)	*P. poplesia*	40S ribosomal protein S30	*Acartia pacifica*	AGN29662	6.74E-47
Ppo_cDNA4(3)	*P. poplesia*	elongation factor 1-alpha	*Nasonia vitripennis*	NP_001166227	0
Ppo_cDNA40	*P. poplesia*	60S acidic ribosomal protein P0	*Lepeophtheirus salmonis*	ACO12290	6.08E-143
Ppo_cDNA41(5)	*P. poplesia*	60S acidic ribosomal protein P1	*Xenopsylla cheopis*	ABM55444	7.17E-18
Ppo_cDNA42	*P. poplesia*	60S ribosomal protein L5	*Riptortus pedestris*	BAN20290	3.12E-142
Ppo_cDNA43(3)	*P. poplesia*	60S ribosomal protein L6	*Zootermopsis nevadensis*	KDR23771	1.24E-81
Ppo_cDNA44	*P. poplesia*	60S ribosomal protein L7	*Lepeophtheirus salmonis*	ADD38610	1.83E-131
Ppo_cDNA45(6)	*P. poplesia*	60S ribosomal protein L10	*Caligus clemensi*	ACO14837	1.32E-135
Ppo_cDNA46-1	*P. poplesia*	60S ribosomal protein L11	*Tribolium castaneum*	XP_971759	3.95E-110
Ppo_cDNA46-2(2)	*P. poplesia*	60S ribosomal protein L11	*Tribolium castaneum*	XP_971759	3.31E-111
Ppo_cDNA46-3	*P. poplesia*	60S ribosomal protein L11	*Tribolium castaneum*	XP_971759	2.18E-110
Ppo_cDNA47(2)	*P. poplesia*	60S ribosomal protein L12	*Anopheles gambiae str. PEST*	XP_319222	1.97E-85
Ppo_cDNA48	*P. poplesia*	60S ribosomal protein L13	*Lepeophtheirus salmonis*	ADD38054	1.15E-85
Ppo_cDNA49	*P. poplesia*	60S ribosomal protein L13a	*Caligus rogercresseyi*	ACO11694	3.54E-105
Ppo_cDNA5(3)	*P. poplesia*	ADP,ATP carrier protein 3	*Lepeophtheirus salmonis*	ACO12488	3.88E-159
Ppo_cDNA50	*P. poplesia*	60S ribosomal protein L14	*Lepeophtheirus salmonis*	ACO12382	2.46E-44
Ppo_cDNA51	*P. poplesia*	60S ribosomal protein L15	*Caligus clemensi*	ACO14762	2.03E-115
Ppo_cDNA52	*P. poplesia*	60S ribosomal protein L17	*Caligus rogercresseyi*	ACO11567	1.54E-102
Ppo_cDNA53-1(4)	*P. poplesia*	60S ribosomal protein L18	*Nasonia vitripennis*	XP_008201914	1.68E-93
Ppo_cDNA53-2	*P. poplesia*	60S ribosomal protein L18	*Nasonia vitripennis*	XP_008201914	5.42E-95
Ppo_cDNA54	*P. poplesia*	Ppo_cDNA54	*60S ribosomal protein L19*	Nasonia vitripennis	XP_008210052
Ppo_cDNA55(8)	*P. poplesia*	60S ribosomal protein L21	*Hydra vulgaris*	XP_002166786	3.54E-67
Ppo_cDNA56-1	*P. poplesia*	60S ribosomal protein L22	*Acartia pacifica*	AGN29654	3.08E-39
Ppo_cDNA56-2(4)	*P. poplesia*	60S ribosomal protein L22	*Acartia pacifica*	AGN29654	2.56E-37

Ppo_cDNA57-1(2)	*P. poplesia*	60S ribosomal protein L23a	*Caligus clemensi*	ACO15087	2.48E-76
Ppo_cDNA57-2	*P. poplesia*	60S ribosomal protein L23a	*Caligus clemensi*	ACO15087	5.26E-76
Ppo_cDNA58	*P. poplesia*	60S ribosomal protein L24	*Chrysomela tremula*	ACY71250	2.00E-51
Ppo_cDNA59(3)	*P. poplesia*	60S ribosomal protein L26	*Daphnia pulex*	EFX71534	1.03E-80
Ppo_cDNA6(2)	*P. poplesia*	ATP synthase subunit d, mitochondrial	*Lepeophtheirus salmonis*	ACO12468	4.55E-41
Ppo_cDNA60(6)	*P. poplesia*	60S ribosomal protein L27	*Latrodectus hesperus*	ADV40079	3.86E-57
Ppo_cDNA61-1	*P. poplesia*	60S ribosomal protein L27a	*Lepeophtheirus salmonis*	ACO12227	5.94E-65
Ppo_cDNA61-2	*P. poplesia*	60S ribosomal protein L27a	*Lepeophtheirus salmonis*	ACO12227	1.31E-66
Ppo_cDNA61-3(3)	*P. poplesia*	60S ribosomal protein L27a	*Lepeophtheirus salmonis*	ACO12227	2.35E-69
Ppo_cDNA62	*P. poplesia*	60S ribosomal protein L29	*Neodiplogaster sp. WEM-2009*	ACV20954	3.73E-16
Ppo_cDNA63	*P. poplesia*	60S ribosomal protein L30	*Argopecten irradians*	AAN05584	7.45E-63
Ppo_cDNA64(4)	*P. poplesia*	60S ribosomal protein L32	*Bombus terrestris*	XP_003398978	3.22E-76
Ppo_cDNA65	*P. poplesia*	60S ribosomal protein L34	*Lepeophtheirus salmonis*	ACO13005	2.16E-48
Ppo_cDNA66(6)	*P. poplesia*	unknown protein			
Ppo_cDNA67-1	*P. poplesia*	60S ribosomal protein L36	*Caligus rogercresseyi*	ACO11656	2.36E-41
Ppo_cDNA67-2(3)	*P. poplesia*	60S ribosomal protein L36	*Caligus rogercresseyi*	ACO11656	2.70E-43
Ppo_cDNA68-1(5)	*P. poplesia*	60S ribosomal protein L37	*Acartia pacifica*	AGN29663	3.12E-41
Ppo_cDNA68-2	*P. poplesia*	60S ribosomal protein L37a	*Lepeophtheirus salmonis*	ADD38662	1.53E-42
Ppo_cDNA69-1(3)	*P. poplesia*	60S ribosomal protein L38	*Apis mellifera*	XP_001120263	2.41E-37
Ppo_cDNA69-2	*P. poplesia*	60S ribosomal protein L38	*Apis mellifera*	XP_001120263	5.50E-37
Ppo_cDNA7(2)	*P. poplesia*	Cellular retinoic acid-binding protein 2	*Caligus rogercresseyi*	ACO11291	6.60E-31
Ppo_cDNA70	*P. poplesia*	60S ribosomal protein L39	*Lineus viridis*	ABZ04248	3.58E-22
Ppo_cDNA71(4)	*P. poplesia*	ubiquitin-60S ribosomal protein L40	*Tupaia chinensis*	ELW67145	1.48E-80
Ppo_cDNA72(2)	*P. poplesia*	60S ribosomal protein L44	*Acartia pacifica*	AGN29651	5.64E-55
Ppo_cDNA73	*P. poplesia*	39S ribosomal protein L16, mitochondrial	*Lepeophtheirus salmonis*	ACO12497	7.75E-63
Ppo_cDNA74	*P. poplesia*	26S proteasome non-ATPase regulatory subunit 14	*Branchiostoma floridae*	XP_002611030	0
Ppo_cDNA75	*P. poplesia*	eukaryotic translation initiation factor 5A	*Lepeophtheirus salmonis*	ACO12518	4.21E-78
Ppo_cDNA76	*P. poplesia*	cytochrome c	*Tigriopus californicus*	AAC80545	6.28E-52
Ppo_cDNA77	*P. poplesia*	protein nap	*Caligus rogercresseyi*	ACO11029	7.54E-99
Ppo_cDNA78	*P. poplesia*	ATP synthase subunit f, mitochondrial	*Lepeophtheirus salmonis*	ACO12343	1.80E-40
Ppo_cDNA79	*P. poplesia*	macrophage migration inhibitory factor	*Acyrthosiphon pisum*	XP_008184019	1.78E-38
Ppo_cDNA8(2)	*P. poplesia*	cytochrome c oxidase subunit Va protein-like protein	*Artemia franciscana*	ACT31598	2.47E-38
Ppo_cDNA80	*P. poplesia*	paxillin-like isoform X4	*Bombyx mori*	XP_004921621	1.25E-41
Ppo_cDNA81	*P. poplesia*	peroxiredoxin 6	*Saccoglossus kowalevskii*	XP_002733706	7.57E-110
Ppo_cDNA82	*P. poplesia*	proactivator polypeptide, putative	*Acanthamoeba castellanii str. Neff*	XP_004355028	3.46E-08
Ppo_cDNA83	*P. poplesia*	NADH dehydrogenase	*ubiquinone*	XP_003744447	1.60E-29
Ppo_cDNA84	*P. poplesia*	cytochrome b-c1 complex subunit 6, mitochondrial	*Lepeophtheirus salmonis*	ADD37932	1.93E-23

Ppo_cDNA85	*P. poplesia*	PREDICTED: ribonuclease UK114	*Stegastes partitus*	XP_008290399	5.62E-60
Ppo_cDNA86	*P. poplesia*	ubiquinol-cytochrome C reductase	*Tribolium castaneum*	XP_008201548	6.72E-05
Ppo_cDNA87	*P. poplesia*	unkown protein	*Riptortus pedestris*	BAN20403	2.80E-05
Ppo_cDNA88	*P. poplesia*	cytochrome c oxidase subunit 5B, mitochondrial precursor	*Lepeophtheirus salmonis*	ACO12623	8.21E-45
Ppo_cDNA89	*P. poplesia*	elongation factor 1-delta	*Lepeophtheirus salmonis*	ACO11944	3.77E-62
Ppo_cDNA90	*P. poplesia*	14-3-3 zeta	*Megachile rotundata*	AEW70348	4.21E-149
Ppo_cDNA91	*P. poplesia*	GTP-binding nuclear protein Ran	*Solenopsis invicta*	EFZ10904	3.86E-148
Ppo_cDNA9-1	*P. poplesia*	ATP synthase delta chain, mitochondrial	*Aedes aegypti*	XP_001655447	1.33E-46
Ppo_cDNA92	*P. poplesia*	lethal 35Di	*Microplitis demolitor*	XP_008547855	1.45E-20
Ppo_cDNA9-2(2)	*P. poplesia*	ATP synthase delta chain, mitochondrial	*Aedes aegypti*	XP_001655447	9.92E-47
Ppo_cDNA93	*P. poplesia*	perlucin 5-like protein	*Acartia pacifica*	AGN29615	6.50E-63
Ppo_cDNA94	*P. poplesia*	translocon-associated protein subunit gamma-like	*Musca domestica*	XP_005188284	3.08E-64
Ppo_cDNA95	*P. poplesia*	28S ribosomal protein L42, mitochondrial	*Zootermopsis nevadensis*	KDR11909	1.54E-13
Ppo_cDNA96	*P. poplesia*	endoplasmic reticulum membrane protein YGL010W	*Lepeophtheirus salmonis*	ACO12654	9.02E-45
Ppo_cDNA97	*P. poplesia*	proteasome subunit beta type-1	*Microplitis demolitor*	XP_008553778	3.63E-104
Ppo_cDNA98	*P. poplesia*	cytochrome c oxidase polypeptide IV	*Lepeophtheirus salmonis*	ADD38391	5.62E-59
Ppo_cDNA99	*P. poplesia*	unknown protein			
Ppo16	*P. poplesia*	60S ribosomal protein L34	*Caligus clemensi*	ACO14672	4.62E-54
Ppo26(3)	*P. poplesia*	60S ribosomal protein L27	*Latrodectus hesperus*	ADV40079	4.57E-57
PPON_ZN78	*P. poplesia*	low quality protein: glutathione peroxidase 2	*Ornithorhynchus anatinus*	XP_007661684	2.03E-58
PPON1(8)	*P. poplesia*	40S ribosomal protein S18	*Caligus rogercresseyi*	ACO11045	1.94E-75
PPON10(4)	*P. poplesia*	60S ribosomal protein L39	*Lineus viridis*	ABZ04248	3.58E-22
PPON100	*P. poplesia*	unknown protein			
PPON101(7)	*P. poplesia*	60S ribosomal protein L27a	*Lepeophtheirus salmonis*	ACO12227	9.46E-66
PPON102(2)	*P. poplesia*	40S ribosomal protein S11	*Acartia pacifica*	AGN29665	6.85E-93
PPON103(2)	*P. poplesia*	39S ribosomal protein L36, mitochondrial, putative	*Daphnia pulex*	EFX68183	8.55E-09
PPON104	*P. poplesia*	unknown protein			
PPON105	*P. poplesia*	PPON105	*probable RNA-binding protein 19-like*	Zootermopsis nevadensis	KDR21962
PPON106	*P. poplesia*	unknown protein			
PPON107	*P. poplesia*	NADH dehydrogenase	*ubiquinone*	EFN66709	8.14E-12
PPON108(2)	*P. poplesia*	60S ribosomal protein L8	*Acartia pacifica*	AGN29659	2.63E-165
PPON109	*P. poplesia*	PPON109	*T-complex protein 1 subunit gamma*	Diaphorina citri	XP_008479 409
PPON110	*P. poplesia*	60S ribosomal protein L12	*Anopheles gambiae str. PEST*	XP_319222	1.97E-85
PPON111	*P. poplesia*	unknown protein			
PPON11-1(2)	*P. poplesia*	unknown protein			
PPON11-2	*P. poplesia*	unknown protein			
PPON11-3(2)	*P. poplesia*	unknown protein			
PPON12	*P. poplesia*	NIF3-like protein 1	*Crassostrea gigas*	EKC35256	9.31E-66
PPON13(4)	*P. poplesia*	60S ribosomal protein L10	*Caligus clemensi*	ACO14837	9.43E-136
PPON14(3)	*P. poplesia*	40S ribosomal protein S26	*Lepeophtheirus salmonis*	ACO12442	1.09E-46

PPON15(3)	*P. poplesia*	40S ribosomal protein S19	*Lineus viridis*	ABZ04268	4.94E-70
PPON16(2)	*P. poplesia*	40S ribosomal protein S3a	*Lepeophtheirus salmonis*	ACO12723	3.66E-141
PPON17(5)	*P. poplesia*	60S ribosomal protein L28	*Acartia pacifica*	AGN29640	2.01E-55
PPON18(2)	*P. poplesia*	60S ribosomal protein L27	*Latrodectus hesperus*	ADV40079	3.60E-57
PPON19(8)	*P. poplesia*	60S ribosomal protein L21	*Hydra vulgaris*	XP_002166786	1.05E-66
PPON2	*P. poplesia*	60S ribosomal protein L5	*Riptortus pedestris*	BAN20290	2.76E-110
PPON20	*P. poplesia*	phenylalanine-4-hydroxylase	*Culex quinquefasciatus*	XP_001843416	4.90E-175
PPON21	*P. poplesia*	FKBP-type peptidyl-prolyl cis-trans isomerase-12	*Acartia pacifica*	AGN29631	1.39E-32
PPON22	*P. poplesia*	60S ribosomal protein L6	*Zootermopsis nevadensis*	KDR23771	2.39E-87
PPON23(7)	*P. poplesia*	40S ribosomal protein S30	*Acartia pacifica*	AGN29662	6.53E-47
PPON24	*P. poplesia*	14-3-3 zeta	*Apis florea*	AEW70339	3.71E-153
PPON25(8)	*P. poplesia*	60S ribosomal protein L30	*Daphnia pulex*	EFX64469	1.57E-63
PPON26	*P. poplesia*	60S ribosomal protein L14	*Lepeophtheirus salmonis*	ACO12382	1.72E-44
PPON27(4)	*P. poplesia*	60S ribosomal protein L35a	*Lepeophtheirus salmonis*	ACO12970	1.87E-58
PPON28(2)	*P. poplesia*	60S ribosomal protein L18	*Nasonia vitripennis*	XP_008201914	3.32E-94
PPON29(5)	*P. poplesia*	60S ribosomal protein L37	*Acartia pacifica*	AGN29663	4.00E-41
PPON3(8)	*P. poplesia*	60S ribosomal protein L37a	*Lepeophtheirus salmonis*	ADD38662	7.99E-43
PPON30	*P. poplesia*	dolichyl-diphosphooligosaccharide--protein glycosyltransferase subunit DAD1-like	*Aplysia californica*	XP_005113102	3.76E-53
PPON31-1(4)	*P. poplesia*	Cellular retinoic acid-binding protein 2	*Caligus rogercresseyi*	ACO11291	6.51E-30
PPON31-2	*P. poplesia*	Cellular retinoic acid-binding protein 2	*Caligus rogercresseyi*	ACO11291	5.62E-37
PPON32	*P. poplesia*	60S ribosomal protein L9	*Acartia pacifica*	AGN29667	2.56E-99
PPON33-1	*P. poplesia*	60S ribosomal protein L24	*Tribolium castaneum*	XP_966448	4.71E-45
PPON34-2(7)	*P. poplesia*	60S ribosomal protein L24	*Chrysomela tremula*	ACY71250	1.99E-51
PPON35(4)	*P. poplesia*	60S ribosomal protein L23	*Acartia pacifica*	AGN29657	7.27E-86
PPON36(4)	*P. poplesia*	60S ribosomal protein L18a	*Caligus clemensi*	ACO14776	6.91E-98
PPON37(2)	*P. poplesia*	40S ribosomal protein S21	*Acartia pacifica*	AGN29649	7.97E-45
PPON38	*P. poplesia*	DNA-directed RNA polymerases I and III subunit RPAC1-like	*Ceratitis capitata*	XP_004531428	8.72E-105
PPON39(3)	*P. poplesia*	60S ribosomal protein L7	*Lepeophtheirus salmonis*	ADD38610	8.26E-124
PPON40	*P. poplesia*	60S ribosomal protein L38	*Apis mellifera*	XP_001120263	2.17E-37
PPON41(6)	*P. poplesia*	40S ribosomal protein S28	*Megachile rotundata*	XP_003700807	2.09E-24
PPON42(4)	*P. poplesia*	60S ribosomal protein L15	*Caligus clemensi*	ACO14762	2.03E-115
PPON43(2)	*P. poplesia*	40S ribosomal protein S14	*Caligus clemensi*	ACO14952	1.56E-86
PPON44	*P. poplesia*	40S ribosomal protein S13	*Branchiostoma belcheri*	AAN52387	8.52E-96
PPON45	*P. poplesia*	NADH dehydrogenase	*Lepeophtheirus salmonis*	ACO12839	5.98E-98
PPON46	*P. poplesia*	ATP synthase subunit epsilon, mitochondrial	*Lepeophtheirus salmonis*	ADD38640	1.70E-15
PPON47(8)	*P. poplesia*	ubiquitin-40S ribosomal protein S27a	*Saccoglossus kowalevskii*	XP_002733357	8.02E-75
PPON48	*P. poplesia*	PPON48	*bifunctional aminoacyl-tRNA synthetase*	Anopheles sinensis	KFB52703
PPON49(2)	*P. poplesia*	40S ribosomal protein S5	*Timarcha balearica*	CAH04317	2.08E-120
PPON5(6)	*P. poplesia*	60S ribosomal protein L13	*Lepeophtheirus salmonis*	ADD38054	1.14E-84
PPON50	*P. poplesia*	ATP synthase subunit d, mitochondrial	*Lepeophtheirus salmonis*	ACO12468	1.97E-41
PPON51(2)	*P. poplesia*	PPON51(2)	*unknown protein*		

PPON52	*P. poplesia*	60S ribosomal protein L7a/NHP2-like protein 1	*Caligus rogercresseyi*	ACO10185	1.16E-62
PPON53(5)	*P. poplesia*	40S ribosomal protein S15	*Barentsia elongata*	ABW90420	5.92E-60
PPON54	*P. poplesia*	ras-related C3 botulinum toxin substrate 1 isoform X2	*Daphnia pulex*	EFX90140	4.59E-126
PPON55(6)	*P. poplesia*	unknown protein			
PPON56	*P. poplesia*	40S ribosomal protein S17a	*Acartia pacifica*	AGN29647	4.12E-87
PPON57	*P. poplesia*	isoform A	*Tribolium castaneum*	XP_008192720	8.61E-07
PPON58	*P. poplesia*	unknown protein			
PPON59	*P. poplesia*	unknown protein			
PPON60	*P. poplesia*	unknown protein			
PPON61	*P. poplesia*	profilin	*Pseudodiaptomus annandalei*	AGT28477	4.84E-71
PPON62	*P. poplesia*	PPON62	*PREDICTED: probable RISC-loading complex subunit BRAFLDRAFT_242885 -like isoform X1*	Aplysia californica	XP_005098 096
PPON63	*P. poplesia*	cytochrome b-c1 complex subunit 6, mitochondrial	*Lepeophtheirus salmonis*	ADD37932	4.51E-23
PPON64(3)	*P. poplesia*	60S ribosomal protein L17	*Caligus rogercresseyi*	ACO11567	1.76E-101
PPON65	*P. poplesia*	28S ribosomal protein S23, mitochondrial	*Zootermopsis nevadensis*	KDR18398	7.35E-30
PPON66(3)	*P. poplesia*	40S ribosomal protein S3	*Zootermopsis nevadensis*	KDR16813	6.99E-148
PPON67	*P. poplesia*	unknown protein			
PPON68	*P. poplesia*	40S ribosomal protein S10	*Xenopsylla cheopis*	ABM55456	7.90E-69
PPON69	*P. poplesia*	60S ribosomal protein L19	*Harpegnathos saltator*	EFN86617	1.19E-82
PPON70	*P. poplesia*	unknown protein			
PPON71	*P. poplesia*	microtubule-associated proteins 1A/1B light chain 3B	*Poecilia formosa*	XP_007553263	1.32E-10
PPON72	*P. poplesia*	40S ribosomal protein S8	*Bombyx mori*	NP_001037263	3.55E-111
PPON73(2)	*P. poplesia*	nucleoside diphosphate kinase A 1	*Lepeophtheirus salmonis*	ACO12519	1.37E-91
PPON74(2)	*P. poplesia*	40S ribosomal protein S17	*Lepeophtheirus salmonis*	ADD24334	1.52E-67
PPON75-1(3)	*P. poplesia*	40S ribosomal protein S21	*Acartia pacifica*	AGN29649	1.84E-44
PPON75-2(2)	*P. poplesia*	40S ribosomal protein S21	*Acartia pacifica*	AGN29649	7.97E-45
PPON76	*P. poplesia*	60S ribosomal protein L29	*Solea senegalensis*	BAF98680	2.39E-18
PPON77(4)	*P. poplesia*	ATP synthase lipid-binding protein, mitochondrial precursor	*Salmo salar*	ACI69678	7.63E-18
PPON78	*P. poplesia*	unknown protein			
PPON79	*P. poplesia*	60S ribosomal protein L38	*Apis mellifera*	XP_001120263	3.30E-37
PPON8(4)	*P. poplesia*	40S ribosomal protein S16	*Xenopsylla cheopis*	ABM55425	8.68E-82
PPON80	*P. poplesia*	H/ACA ribonucleoprotein complex subunit 2-like	*Apis mellifera*	XP_001120529	1.47E-30
PPON81(2)	*P. poplesia*	40S ribosomal protein S20	*Nematostella vectensis*	XP_001629850	3.34E-57
PPON82(5)	*P. poplesia*	60S ribosomal protein L35	*Dendroctonus ponderosae*	AEE61452	8.31E-61
PPON83(3)	*P. poplesia*	ubiquitin-60S ribosomal protein L40	*Tupaia chinensis*	ELW67145	1.24E-80
PPON84(3)	*P. poplesia*	ATP synthase subunit g, mitochondrial	*Helobdella robusta*	ESN96292	1.91E-17
PPON85	*P. poplesia*	U6 snRNA-associated Sm-like protein LSm1	*Lepeophtheirus salmonis*	ADD38614	1.17E-36
PPON86(2)	*P. poplesia*	60S ribosomal protein L26	*Daphnia pulex*	EFX71534	5.64E-80
PPON87(7)	*P. poplesia*	60S ribosomal protein L44	*Acartia pacifica*	AGN29651	5.64E-55
PPON88	*P. poplesia*	unknown protein			

PPON89	*P. poplesia*	ubiquinol-cytochrome C reductase	*Ixodes scapularis*	AAY66878	5.42E-05
PPON90(8)	*P. poplesia*	60S ribosomal protein L32	*Bombus terrestris*	XP_003398978	6.33E-73
PPON91	*P. poplesia*	39S ribosomal protein L55, mitochondrial	*Ixodes scapularis*	XP_002408652	2.49E-20
PPON9-1(5)	*P. poplesia*	40S ribosomal protein S23	*Lepeophtheirus salmonis*	ACO12024	1.24E-90
PPON92	*P. poplesia*	40S ribosomal protein S30	*Danaus plexippus*	EHJ69365	4.97E-37
PPON9-2	*P. poplesia*	40S ribosomal protein S23	*Lepeophtheirus salmonis*	ACO12024	8.04E-92
PPON93(3)	*P. poplesia*	60S ribosomal protein L36	*Caligus rogercresseyi*	ACO11656	1.35E-43
PPON94	*P. poplesia*	40S ribosomal protein S24	*Lepeophtheirus salmonis*	ACO12534	2.83E-49
PPON95	*P. poplesia*	small integral membrane protein 15	*Ixodes scapularis*	XP_002435047	6.19E-06
PPON96	*P. poplesia*	28S ribosomal protein L42, mitochondrial	*Zootermopsis nevadensis*	KDR11909	1.71E-13
PPON97	*P. poplesia*	unknown protein			
PPON98	*P. poplesia*	unknown protein			
PPON99	*P. poplesia*	60S ribosomal protein L11	*Tribolium castaneum*	XP_971759	5.44E-111
PPOP1(4)	*P. poplesia*	60S ribosomal protein L32	*Daphnia pulex*	EFX89085	1.57E-63
PPOP10	*P. poplesia*	60S ribosomal protein L36	*Caligus rogercresseyi*	ACO11656	1.26E-43
PPOP100	*P. poplesia*	isoform A	*Tribolium castaneum*	XP_008192720	1.58E-08
PPOP101(2)	*P. poplesia*	40S ribosomal protein S15	*Barentsia elongata*	ABW90420	5.92E-60
PPOP102	*P. poplesia*	unknown protein			
PPOP104(2)	*P. poplesia*	60S ribosomal protein L13	*Lepeophtheirus salmonis*	ADD38054	1.33E-85
PPOP105	*P. poplesia*	CDP-diacylglycerol--inositol 3-phosphatidyltransferase-like	*Solenopsis invicta*	EFZ15295	1.39E-92
PPOP106	*P. poplesia*	ubiquitin-60S ribosomal protein L40	*Branchiostoma belcheri*	AAK91296	6.72E-80
PPOP107	*P. poplesia*	NADH dehydrogenase	*ubiquinone*	XP_003744447	1.47E-29
PPOP108	*P. poplesia*	60S ribosomal protein L18a	*Caligus clemensi*	ACO14776	8.01E-98
PPOP109	*P. poplesia*	60S ribosomal protein L24	*Chrysomela tremula*	ACY71250	1.99E-51
PPOP11	*P. poplesia*	NADH dehydrogenase	*Aedes aegypti*	XP_001659261	1.57E-17
PPOP110	*P. poplesia*	60S ribosomal protein L24	*Tribolium castaneum*	XP_966448	5.71E-44
PPOP111	*P. poplesia*	40S ribosomal protein S17	*Caligus clemensi*	ACO14607	1.63E-67
PPOP112(3)	*P. poplesia*	40S ribosomal protein S3a	*Lepeophtheirus salmonis*	ACO12723	5.27E-141
PPOP113	*P. poplesia*	PPOP113	*T-complex protein 1 subunit gamma*	Diaphorina citri	XP_008479 409
PPOP114	*P. poplesia*	cytochrome b-c1 complex subunit 9	*Diaphorina citri*	XP_008468754	1.60E-17
PPOP115	*P. poplesia*	unknown protein			
PPOP116	*P. poplesia*	unknown protein			
PPOP117	*P. poplesia*	40S ribosomal protein S20	*Nematostella vectensis*	XP_001629850	2.58E-57
PPOP12(2)	*P. poplesia*	60S ribosomal protein L14	*Spodoptera frugiperda*	AAK92157	1.36E-45
PPOP13-1(2)	*P. poplesia*	40S ribosomal protein S14	*Caligus clemensi*	ACO14952	1.60E-87
PPOP13-2	*P. poplesia*	40S ribosomal protein S14	*Caligus clemensi*	ACO14952	5.89E-86
PPOP13-3(2)	*P. poplesia*	40S ribosomal protein S14	*Caligus clemensi*	ACO14952	2.98E-86
PPOP14	*P. poplesia*	unknown protein			
PPOP15(2)	*P. poplesia*	unknown protein			
PPOP17(2)	*P. poplesia*	ATP synthase lipid-binding protein, mitochondrial precursor	*Caligus rogercresseyi*	ACO10993	7.69E-31
PPOP18(3)	*P. poplesia*	40S ribosomal protein S7	*Acartia pacifica*	AGN29653	1.08E-116
PPOP19(4)	*P. poplesia*	60S ribosomal protein L14	*Lepeophtheirus salmonis*	ACO12382	5.76E-44
PPOP2(4)	*P. poplesia*	40S ribosomal protein S26	*Lepeophtheirus salmonis*	ACO12442	9.61E-47
PPOP20	*P. poplesia*	40S ribosomal protein S10	*Acartia pacifica*	AGN29661	6.48E-58

PPOP21	*P. poplesia*	hypothetical protein	*Lepeophtheirus salmonis*	ABU41081	1.67E-15
PPOP22(4)	*P. poplesia*	60S ribosomal protein L37	*Acartia pacifica*	AGN29663	4.29E-41
PPOP23	*P. poplesia*	40S ribosomal protein S3	*Zootermopsis nevadensis*	KDR16813	1.27E-139
PPOP24	*P. poplesia*	cytochrome c	*Tigriopus californicus*	AAC80545	7.37E-52
PPOP25(2)	*P. poplesia*	60S ribosomal protein L38	*Apis mellifera*	XP_001120263	9.30E-37
PPOP27	*P. poplesia*	protein asterix	*Daphnia pulex*	EFX81706	1.76E-47
PPOP28(2)	*P. poplesia*	40S ribosomal protein S15	*Barentsia elongata*	ABW90420	8.01E-73
PPOP29	*P. poplesia*	60S ribosomal protein L32	*Bombus terrestris*	XP_003398978	3.34E-76
PPOP3	*P. poplesia*	unknown protein			
PPOP30	*P. poplesia*	translocon-associated protein subunit gamma-like	*Aplysia californica*	XP_005112740	5.04E-58
PPOP31(5)	*P. poplesia*	60S ribosomal protein L28	*Acartia pacifica*	AGN29640	1.88E-55
PPOP32	*P. poplesia*	reticulocalbin-2-like isoform X1	*Anopheles sinensis*	KFB45689	4.40E-21
PPOP33	*P. poplesia*	60S ribosomal protein L21	*Lepeophtheirus salmonis*	ACO12449	1.25E-66
PPOP34-1	*P. poplesia*	40S ribosomal protein S24	*Cerapachys biroi*	EZA57262	5.38E-35
PPOP34-2(2)	*P. poplesia*	40S ribosomal protein S24	*Lepeophtheirus salmonis*	ACO12534	2.82E-46
PPOP34-3	*P. poplesia*	40S ribosomal protein S24	*Lepeophtheirus salmonis*	ACO12534	1.87E-46
PPOP34-4(2)	*P. poplesia*	40S ribosomal protein S24	*Lepeophtheirus salmonis*	ACO12534	2.68E-44
PPOP35-1(4)	*P. poplesia*	40S ribosomal protein S25	*Acartia pacifica*	AGN29668	1.29E-33
PPOP35-2	*P. poplesia*	40S ribosomal protein S25	*Acartia pacifica*	AGN29668	2.21E-34
PPOP36(4)	*P. poplesia*	60S acidic ribosomal protein P1	*Ixodes scapularis*	XP_002435967	2.13E-26
PPOP37	*P. poplesia*	acyl carrier protein, mitochondrial	*Lepeophtheirus salmonis*	ADD38564	4.36E-34
PPOP38	*P. poplesia*	unknown protein			
PPOP39	*P. poplesia*	60S ribosomal protein L39	*Zootermopsis nevadensis*	KDR13442	8.87E-22
PPOP4	*P. poplesia*	60S ribosomal protein L17	*Caligus rogercresseyi*	ACO11567	1.60E-102
PPOP40(2)	*P. poplesia*	unknown protein			
PPOP41	*P. poplesia*	40S ribosomal protein S18a	*Acartia pacifica*	AGN29647	2.44E-82
PPOP42(3)	*P. poplesia*	60S ribosomal protein L15	*Caligus clemensi*	ACO14762	6.98E-114
PPOP43(2)	*P. poplesia*	40S ribosomal protein S19	*Lineus viridis*	ABZ04268	1.69E-69
PPOP44(9)	*P. poplesia*	60S ribosomal protein L27a	*Lepeophtheirus salmonis*	ACO12227	2.04E-69
PPOP45(8)	*P. poplesia*	60S ribosomal protein L21	*Hydra vulgaris*	XP_002166786	1.97E-66
PPOP46(2)	*P. poplesia*	60S ribosomal protein L18	*Caligus clemensi*	ACO15258	1.10E-72
PPOP47(2)	*P. poplesia*	40S ribosomal protein S30	*Acartia pacifica*	AGN29662	7.39E-47
PPOP48(3)	*P. poplesia*	40S ribosomal protein S21	*Acartia pacifica*	AGN29649	8.25E-45
PPOP49(2)	*P. poplesia*	60S ribosomal protein L26	*Tribolium castaneum*	XP_972048	9.67E-75
PPOP5(2)	*P. poplesia*	60S acidic ribosomal protein P2	*Oikopleura dioica*	CBY33355	2.71E-08
PPOP50(4)	*P. poplesia*	60S ribosomal protein L30	*Daphnia pulex*	EFX64469	1.57E-63
PPOP51	*P. poplesia*	PPOP51	*casein kinase II subunit alpha*	Ophiophagus hannah	ETE58132
PPOP52(2)	*P. poplesia*	40S ribosomal protein S14	*Caligus clemensi*	ACO14952	2.98E-86
PPOP53	*P. poplesia*	40S ribosomal protein S12	*Spadella cephaloptera*	CAL69098	1.39E-57
PPOP54(3)	*P. poplesia*	40S ribosomal protein S20	*Azumapecten farreri*	AAM94275	7.26E-58
PPOP55(2)	*P. poplesia*	60S ribosomal protein L7	*Lepeophtheirus salmonis*	ADD38610	5.00E-132
PPOP56	*P. poplesia*	60S ribosomal protein L18	*Nasonia vitripennis*	XP_008201914	3.21E-94
PPOP57	*P. poplesia*	ATP synthase subunit b, mitochondrial precursor	*Caligus clemensi*	ACO14593	1.27E-81
PPOP58(3)	*P. poplesia*	60S ribosomal protein L29	*Solea senegalensis*	BAF98680	2.32E-18
PPOP59(7)	*P. poplesia*	60S ribosomal protein L44	*Acartia pacifica*	AGN29651	5.05E-55

PPOP6(3)	*P. poplesia*	40S ribosomal protein S16	*Xenopsylla cheopis*	ABM55425	8.68E-82
PPOP60(2)	*P. poplesia*	phosphoethanolamine N-methyltransferase 3-like isoform X1	*Arabidopsis thaliana*	AAG52075	7.29E-82
PPOP61-1	*P. poplesia*	60S ribosomal protein L37a	*Lepeophtheirus salmonis*	ADD38662	1.58E-42
PPOP61-2(4)	*P. poplesia*	60S ribosomal protein L37a	*Lepeophtheirus salmonis*	ADD38662	7.99E-43
PPOP62-1(6)	*P. poplesia*	40S ribosomal protein S23	*Lepeophtheirus salmonis*	ACO12024	1.11E-90
PPOP62-2	*P. poplesia*	40S ribosomal protein S23	*Lepeophtheirus salmonis*	ACO12024	8.04E-92
PPOP63(4)	*P. poplesia*	60S ribosomal protein L22	*Acartia pacifica*	AGN29654	2.42E-37
PPOP64	*P. poplesia*	unknown protein			
PPOP65	*P. poplesia*	unknown protein			
PPOP66	*P. poplesia*	RNA-binding protein squid	*Lepeophtheirus salmonis*	ACO12371	3.63E-43
PPOP67	*P. poplesia*	ATP synthase E chain	*Acartia pacifica*	AGN29660	4.98E-31
PPOP68	*P. poplesia*	60S ribosomal protein L7a/NHP2-like protein 1	*Caligus rogercresseyi*	ACO10185	1.24E-62
PPOP69(4)	*P. poplesia*	nucleoside diphosphate kinase A 1	*Lepeophtheirus salmonis*	ACO12519	1.08E-90
PPOP70	*P. poplesia*	60S ribosomal protein L11	*Microplitis demolitor*	XP_008547261	8.95E-108
PPOP71	*P. poplesia*	ATP synthase-coupling factor 6, mitochondrial	*Tribolium castaneum*	XP_008195395	1.17E-28
PPOP72	*P. poplesia*	unknown protein			
PPOP73	*P. poplesia*	mRNA turnover 4 homolog	*Branchiostoma floridae*	XP_002590248	3.50E-87
PPOP74(3)	*P. poplesia*	40S ribosomal protein S12	*Nephrops norvegicus*	CCE46016	1.40E-54
PPOP75	*P. poplesia*	60S ribosomal protein L35	*Lepeophtheirus salmonis*	ADD38309	2.85E-49
PPOP76	*P. poplesia*	60S ribosomal protein L8	*Acartia pacifica*	AGN29659	5.52E-120
PPOP77	*P. poplesia*	protein FAM177A1-like	*Ixodes scapularis*	XP_002435221	3.48E-08
PPOP78(2)	*P. poplesia*	40S ribosomal protein S13	*Equus caballus*	XP_001504999	2.98E-95
PPOP79(2)	*P. poplesia*	40S ribosomal protein S18	*Caligus rogercresseyi*	ACO11045	3.79E-77
PPOP80	*P. poplesia*	unknown protein			
PPOP81	*P. poplesia*	unknown protein			
PPOP82	*P. poplesia*	40S ribosomal protein S24	*Lepeophtheirus salmonis*	ACO12534	1.87E-46
PPOP83	*P. poplesia*	unknown protein			
PPOP84(6)	*P. poplesia*	40S ribosomal protein S19a	*Acartia pacifica*	AGN29647	4.44E-87
PPOP85	*P. poplesia*	60S ribosomal protein L13a	*Caligus rogercresseyi*	ACO11694	2.35E-105
PPOP86(5)	*P. poplesia*	40S ribosomal protein S28	*Megachile rotundata*	XP_003700807	6.26E-24
PPOP87(3)	*P. poplesia*	60S ribosomal protein L11	*Tribolium castaneum*	XP_971759	1.13E-109
PPOP88(3)	*P. poplesia*	40S ribosomal protein S11	*Acartia pacifica*	AGN29665	3.82E-93
PPOP89(2)	*P. poplesia*	40S ribosomal protein S18	*Caligus rogercresseyi*	ACO11045	2.90E-78
PPOP9	*P. poplesia*	ATP synthase subunit g, mitochondrial	*Helobdella robusta*	ESN96292	1.97E-17
PPOP90(2)	*P. poplesia*	60S ribosomal protein L6	*Zootermopsis nevadensis*	KDR23771	1.88E-77
PPOP91(4)	*P. poplesia*	nucleoside diphosphate kinase A 1	*Lepeophtheirus salmonis*	ACO12519	5.70E-66
PPOP92-1	*P. poplesia*	60S ribosomal protein L35a	*Lepeophtheirus salmonis*	ACO12970	5.12E-57
PPOP92-2(3)	*P. poplesia*	60S ribosomal protein L35a	*Lepeophtheirus salmonis*	ACO12970	1.46E-58
PPOP92-3	*P. poplesia*	60S ribosomal protein L35a	*Lepeophtheirus salmonis*	ACO12970	3.23E-43
PPOP93	*P. poplesia*	Cellular retinoic acid-binding protein 2	*Caligus rogercresseyi*	ACO11291	2.85E-30
PPOP94	*P. poplesia*	unknown protein			
PPOP95	*P. poplesia*	PPOP95	*zinc finger protein 271-like*	Bathycoccus prasinos	XP_007508 450

PPOP96	*P. poplesia*	40S ribosomal protein S7	*Acartia pacifica*	AGN29653	8.19E-118
PPOP97(2)	*P. poplesia*	MORN repeat-containing protein	*Strigomonas culicis*	EPY19499	3.46E-23
PPOP98	*P. poplesia*	40S ribosomal protein S25	*Acartia pacifica*	AGN29668	9.60E-37
PPOP99	*P. poplesia*	60S ribosomal protein L24	*Tribolium castaneum*	XP_966448	5.71E-44
PsepoRPL10A	*P. poplesia*	60S ribosomal protein L10a	*Pseudodiaptomus annandalei*	AGT28476	3.00E-131
PsepoRPL3	*P. poplesia*	60S ribosomal protein L3	*Pseudodiaptomus annandalei*	AGT28480	0
PsepoRPL31	*P. poplesia*	60S ribosomal protein L31	*Lepeophtheirus salmonis*	ACO11787	3.00E-58
PsepoRPL4	*P. poplesia*	60S ribosomal protein L4	*Lepeophtheirus salmonis*	ACO12528	0
PsepoRPL7A	*P. poplesia*	60S ribosomal protein L7A	*Stegodyphus mimosarum*	KFM60603	3.00E-117
PsepoRPS2	*P. poplesia*	40S ribosomal protein S2	*Acartia pacifica*	AGN29613	2.00E-158
PsepoRPS27	*P. poplesia*	40S ribosomal protein S27	*Zootermopsis nevadensis*	KDR18843	1.00E-51
PsepoRPS4	*P. poplesia*	40S ribosomal protein S4	*Lepeophtheirus salmonis*	ACO12708	5.00E-141
PsepoRPSA	*P. poplesia*	40S ribosomal protein SA	*Diaphorina citri*	Q0PXX8	5.00E-107
TordecDNA1	*Tortanus dextrilobatus*	40S ribosomal protein S15	*Barentsia elongata*	ABW90420	3.77E-77
TordecDNA10	*T. dextrilobatus*	60S ribosomal protein L32	*Daphnia pulex*	EFX89085	5.08E-63
TordecDNA11	*T. dextrilobatus*	NADH dehydrogenase	*ubiquinone*	XP_003969004	2.97E-23
TordecDNA12	*T. dextrilobatus*	60S ribosomal protein L37a	*Lepeophtheirus salmonis*	ADD38662	1.94E-42
TordecDNA13	*T. dextrilobatus*	60S ribosomal protein L35a	*Lepeophtheirus salmonis*	ACO12970	5.53E-57
TordecDNA14	*T. dextrilobatus*	vacuolar ATP synthase subunit e 2	*Caligus clemensi*	ACO15164	1.13E-29
TordecDNA15	*T. dextrilobatus*	NADH dehydrogenase	*ubiquinone*	NP_001165794	2.54E-14
TordecDNA16	*T. dextrilobatus*	60S ribosomal protein L37	*Acartia pacifica*	AGN29663	4.36E-41
TordecDNA17	*T. dextrilobatus*	60S ribosomal protein L22	*Acartia pacifica*	AGN29654	2.28E-42
TordecDNA18	*T. dextrilobatus*	60S ribosomal protein L10	*Caligus clemensi*	ACO14837	2.17E-130
TordecDNA19	*T. dextrilobatus*	unknown protein			
TordecDNA2	*T. dextrilobatus*	60S ribosomal protein L37	*Acartia pacifica*	AGN29663	7.46E-42
TordecDNA21	*T. dextrilobatus*	60S acidic ribosomal protein P1	*Ixodes scapularis*	XP_002435967	3.48E-26
TordecDNA22	*T. dextrilobatus*	60S ribosomal protein L23a	*Caligus clemensi*	ACO15087	2.17E-74
TordecDNA23(Tde23)	*T. dextrilobatus*	10 kDa heat shock protein, mitochondrial	*Zootermopsis nevadensis*	KDR14059	6.31E-39
TordecDNA25	*T. dextrilobatus*	60S ribosomal protein L38	*Apis mellifera*	XP_001120263	1.36E-34
TordecDNA26	*T. dextrilobatus*	39S ribosomal protein L36, mitochondrial, putative	*Daphnia pulex*	EFX68183	2.43E-09
TordecDNA27	*T. dextrilobatus*	40S ribosomal protein S25	*Acartia pacifica*	AGN29668	9.93E-37
TordecDNA28	*T. dextrilobatus*	60S ribosomal protein L35a	*Lepeophtheirus salmonis*	ACO12970	8.02E-57
TordecDNA29	*T. dextrilobatus*	40S ribosomal protein S13	*Danio rerio*	NP_001002079	8.69E-95
TordecDNA3	*T. dextrilobatus*	cytochrome c	*Spodoptera litura*	AFS31125	2.06E-52
TordecDNA30	*T. dextrilobatus*	60S ribosomal protein L10	*Caligus clemensi*	ACO14837	7.99E-130
TordecDNA31	*T. dextrilobatus*	40S ribosomal protein S16	*Heliconius melpomene*	ABS57443	8.45E-82
TordecDNA32	*T. dextrilobatus*	60S ribosomal protein L11	*Coptotermes formosanus*	AGM32143	1.34E-110
TordecDNA33	*T. dextrilobatus*	40S ribosomal protein S11	*Acartia pacifica*	AGN29665	9.31E-73
TordecDNA34	*T. dextrilobatus*	60S ribosomal protein L24	*Tribolium castaneum*	XP_966448	5.90E-44
TordecDNA35	*T. dextrilobatus*	40S ribosomal protein S12	*Spadella cephaloptera*	CAL69098	1.44E-57
TordecDNA36	*T. dextrilobatus*	CCHC-type zinc finger protein	*Zootermopsis nevadensis*	KDR10130	2.05E-29
TordecDNA37	*T. dextrilobatus*	unknown protein			

TordecDNA38	*T. dextrilobatus*	ATP synthase lipid-binding protein, mitochondrial precursor	*Caligus rogercresseyi*	ACO10993	6.77E-20
TordecDNA40	*T. dextrilobatus*	40S ribosomal protein S26	*Lepeophtheirus salmonis*	ACO12442	1.73E-46
TordecDNA41	*T. dextrilobatus*	DET1- and DDB1-associated protein 1-like	*Culex quinquefasciatus*	XP_001843665	7.57E-21
TordecDNA42	*T. dextrilobatus*	ecdysteroid-regulated protein	*Litopenaeus vannamei*	ADX33319	3.15E-09
TordecDNA45	*T. dextrilobatus*	unknown protein			
TordecDNA47	*T. dextrilobatus*	unknown protein			
TordecDNA48	*T. dextrilobatus*	60S ribosomal protein L32	*Daphnia pulex*	EFX89085	1.59E-62
TordecDNA49	*T. dextrilobatus*	40S ribosomal protein S28	*Culex quinquefasciatus*	XP_001865978	6.52E-24
TordecDNA50	*T. dextrilobatus*	60S ribosomal protein L26	*Zootermopsis nevadensis*	KDR23279	7.56E-75
TordecDNA51	*T. dextrilobatus*	60S ribosomal protein L26	*Zootermopsis nevadensis*	KDR23279	2.96E-52
TordecDNA52	*T. dextrilobatus*	elongation factor 1-alpha	*Tetraodon nigroviridis*	CAF89666	0
TordecDNA53	*T. dextrilobatus*	60S ribosomal protein L35a	*Lepeophtheirus salmonis*	ACO12970	5.32E-57
TordecDNA55	*T. dextrilobatus*	TordecDNA55	*C2 domain-containing protein 3-like*	Aplysia californica	XP_005108991
TordecDNA56	*T. dextrilobatus*	60S ribosomal protein L37a	*Lepeophtheirus salmonis*	ADD38662	1.64E-42
TordecDNA57	*T. dextrilobatus*	60S ribosomal protein L27	*Caligus rogercresseyi*	ACO11360	2.41E-53
TordecDNA58(Tde58)	*T. dextrilobatus*	60S ribosomal protein L38	*Apis mellifera*	XP_001120263	1.69E-36
TordecDNA59	*T. dextrilobatus*	ubiquitin-60S ribosomal protein L40 isoform X2	*Rattus norvegicus*	XP_006252996	1.10E-82
TordecDNA6	*T. dextrilobatus*	40S ribosomal protein S23	*Lepeophtheirus salmonis*	ACO12024	8.36E-92
TordecDNA61	*T. dextrilobatus*	60S ribosomal protein L3	*Acartia pacifica*	AGN29632	2.69E-40
TordecDNA62	*T. dextrilobatus*	unknown protein			
TordecDNA63	*T. dextrilobatus*	60S ribosomal protein L27	*Caligus rogercresseyi*	ACO11360	2.41E-53
TordecDNA65	*T. dextrilobatus*	40S ribosomal protein S27	*Acartia pacifica*	AGN29666	1.14E-27
TordecDNA68	*T. dextrilobatus*	60S ribosomal protein L37	*Acartia pacifica*	AGN29663	1.51E-48
TordecDNA69	*T. dextrilobatus*	40S ribosomal protein S18	*Caligus rogercresseyi*	ACO11045	3.16E-78
TordecDNA7	*T. dextrilobatus*	60S ribosomal protein L44	*Acartia pacifica*	AGN29651	2.93E-64
TordecDNA70	*T. dextrilobatus*	unknown protein			
TordecDNA71(Tde71)	*T. dextrilobatus*	60S ribosomal protein L35a	*Lepeophtheirus salmonis*	ACO12970	5.74E-57
TordecDNA72	*T. dextrilobatus*	40S ribosomal protein S9	*Acyrthosiphon pisum*	XP_001945527	3.50E-113
TordecDNA73(Tde73)	*T. dextrilobatus*	60S ribosomal protein L9	*Acartia pacifica*	AGN29667	6.83E-99
TordecDNA74	*T. dextrilobatus*	unknown protein			
TordecDNA75	*T. dextrilobatus*	40S ribosomal protein S11	*Acartia pacifica*	AGN29665	1.96E-100
TordecDNA76(Tde76)	*T. dextrilobatus*	40S ribosomal protein S26	*Lepeophtheirus salmonis*	ACO12442	7.29E-47
TordecDNA77(Tde77)	*T. dextrilobatus*	60S ribosomal protein L7a/NHP2-like protein 1	*Caligus rogercresseyi*	ACO10185	1.13E-62
TordecDNA8	*T. dextrilobatus*	39S ribosomal protein L17, mitochondrial	*Aedes aegypti*	XP_001656763	5.39E-52
TordecDNA9	*T. dextrilobatus*	unknown protein			
TorfocDNA10(Tfo10)	*Tortanus forcipatus*	transcription elongation factor B polypeptide 1	*Lepeophtheirus salmonis*	ADD37877	6.48E-56
TorfocDNA11	*T. forcipatus*	deoxyuridine 5 -triphosphate mitochondrial isoform X2	*Daphnia pulex*	EFX81802	3.36E-65
TorfocDNA12	*T. forcipatus*	unknown protein			
TorfocDNA14	*T. forcipatus*	TorfocDNA14	*putative zinc finger protein*	Schistosoma mansoni	CCD80531

TorfocDNA15	*T. forcipatus*	40S ribosomal protein S26	*Lepeophtheirus salmonis*	ACO12442	1.23E-46
TorfocDNA16	*T. forcipatus*	60S ribosomal protein L35	*Zootermopsis nevadensis*	KDR15109	7.17E-48
TorfocDNA17	*T. forcipatus*	TorfocDNA17	*FCH domain only protein 2 isoform X5*	Nasonia vitripennis	XP_003425577
TorfocDNA18	*T. forcipatus*	60S ribosomal protein L14	*Spodoptera frugiperda*	AAK92157	2.93E-44
TorfocDNA19	*T. forcipatus*	40S ribosomal protein S9	*Meladema coriacea*	CAH04322	2.60E-115
TorfocDNA2(Tfo2)	*T. forcipatus*	pradc1-like isoform 2	*Zootermopsis nevadensis*	KDR22400	7.51E-58
TorfocDNA20(Tfo20)	*T. forcipatus*	40S ribosomal protein S27	*Acartia pacifica*	AGN29666	7.56E-48
TorfocDNA21	*T. forcipatus*	DNA-directed RNA polymerase II subunit RPB11	*Zootermopsis nevadensis*	KDR21907	5.39E-67
TorfocDNA22	*T. forcipatus*	glutathione S-transferase delta-epsilon 2	*Tigriopus japonicus*	ACE81245	1.07E-80
TorfocDNA23	*T. forcipatus*	60S ribosomal protein L23	*Acartia pacifica*	AGN29657	9.63E-86
TorfocDNA26	*T. forcipatus*	WD repeat-containing protein 61	*Nasonia vitripennis*	XP_001605432	6.32E-113
TorfocDNA28	*T. forcipatus*	TorfocDNA28	*unknown protein*		
TorfocDNA29	*T. forcipatus*	40S ribosomal protein S28	*Culex quinquefasciatus*	XP_001865978	1.07E-21
TorfocDNA30	*T. forcipatus*	40S ribosomal protein S30	*Nasonia vitripennis*	XP_008215923	4.25E-50
TorfocDNA31	*T. forcipatus*	unknown protein			
TorfocDNA32	*T. forcipatus*	40S ribosomal protein S15a	*Acartia pacifica*	AGN29647	3.16E-86
TorfocDNA33	*T. forcipatus*	NADH dehydrogenase	*ubiquinone*	XP_004932137	1.40E-42
TorfocDNA34(Tfo34)	*T. forcipatus*	ubiquitin-conjugating enzyme E2	*Daphnia pulex*	EFX70655	3.52E-94
TorfocDNA35	*T. forcipatus*	60S ribosomal protein L13a	*Caligus rogercresseyi*	ACO11694	2.46E-106
TorfocDNA36(Tfo36)	*T. forcipatus*	unknown protein			
TorfocDNA37	*T. forcipatus*	unknown protein			
TorfocDNA38	*T. forcipatus*	40S ribosomal protein S24	*Lepeophtheirus salmonis*	ACO12534	4.66E-40
TorfocDNA39	*T. forcipatus*	TorfocDNA39	*proteasome subunit alpha type-4*	Cerapachys biroi	EZA55885
TorfocDNA4(Tfo4)	*T. forcipatus*	small nuclear ribonucleoprotein e	*Crassostrea gigas*	EKC36056	6.89E-41
TorfocDNA42	*T. forcipatus*	40S ribosomal protein S9	*Meladema coriacea*	CAH04322	1.39E-115
TorfocDNA43	*T. forcipatus*	unknown protein			
TorfocDNA45(Tfo45)	*T. forcipatus*	U6 snRNA-associated Sm-like protein LSm3	*Zootermopsis nevadensis*	KDR18195	4.23E-40
TorfocDNA47	*T. forcipatus*	40S ribosomal protein S26	*Lepeophtheirus salmonis*	ACO12442	1.23E-46
TorfocDNA8(Tfo8)	*T. forcipatus*	60S ribosomal protein L35a	*Lepeophtheirus salmonis*	ACO12970	3.32E-56
TorfocDNA9	*T. forcipatus*	tubulin alpha	*Mytilus galloprovincialis*	AIJ27191	2.06E-116

Table S3a1. nblast of CopepodSL GTCTAAWACGACCAAGCTWAWWTTGCTTGATTCACTTCWWYAAGAG against DDBJ/EMBL/GenBank nr/nt, EST and TSA databases.
Note 1: Database: nt+tsa+est; 106,461,619 sequences; 119,623,300,248 total letters. Matrix: blastn matrix: 1 -3; Gap Penalties: Existence: 5, Extension: 2.
Note 2: Total hits 1220, including 1121 hits to *Calanus finmarchicus* sequences, 33 to *Calanus helgolandicus*, 1 to *Calanus glacialis*, 1 to *Eurytemora affinis*, and 64 to non-copepod species.
Note 3: For the 64 non-copepod sequences; tblastx of these sequences all hit copepod transcripts except 11 sequences without hit to any known seqeunces. For details see Table S3a2.

>*Calanus glacialis* mitochondrial heat shock protein 60 (HSP60) mRNA, complete cds; nuclear gene for mitochondrial product. Sequence ID: gb|EU263637. Length: 2092. Number of Matches: 1
58.0 bits(29) 2e-09 35/37(95%) 0/37(0%) Plus/Plus

```
Query  2   TCTAAAACGACCAAGCTWAWWTTGCTTGATTCACTTC  38
           ||||||||||||||||| |  ||||||||||||||||
Sbjct  1   TCTAAAACGACCAAGCTAATATTGCTTGATTCACTTC  37
```

>*Eurytemora affinis* glucose-regulated protein 78kDa (GRP78) mRNA, complete cds Sequence ID: gb|JF811334. Length: 2370. Number of Matches: 1
28.2 bits(14) 1.5 14/14(100%) 0/14(0%) Plus/Plus

```
Query  25  GCTTGATTCACTTCCTTCWWYAAGAG  46
           ||||||||||||||
Sbjct  1   GCTTGATTCACTTC----taaaagag  22
```

>JZ760438|JZ760438.1 awis5P0020M09_F.ab1 Sea ice eukaryotic metatranscriptome Weddell Sea 2 uncultured organism cDNA, mRNA sequence. Length = 523; score = 68.8 bits (31), expect = 2e-09; identities = 38/45 (84%); strand = Plus / Plus

```
Query: 2  tctaawacgaccaagctwawwttgcttgattcacttcwwyaagag 46
          ||||| ||||||||||| |  |||||||||||||||   |||||
Sbjct: 1  tctaaaacgaccaagctaatattgcttgattcacttctacaagag 45
```

>JZ758879|JZ758879.1 awis5P0013P12_F.ab1 Sea ice eukaryotic metatranscriptome Weddell Sea 2 uncultured organism cDNA, mRNA sequence. Length = 683; score = 68.8 bits (31), expect = 2e-09; identities = 38/45 (84%); strand = Plus / Plus

```
Query: 2  tctaawacgaccaagctwawwttgcttgattcacttcwwyaagag 46
          ||||| ||||||||||| |  |||||||||||||||   |||||
Sbjct: 1  tctaaaacgaccaagctaatattgcttgattcacttctacaagag 45
```

>JZ757307|JZ757307.1 awis5P0007A02_F.ab1 Sea ice eukaryotic metatranscriptome Weddell Sea 2 uncultured organism cDNA, mRNA sequence. Length = 603; score = 68.8 bits (31), expect = 2e-09; identities = 38/45 (84%); strand = Plus / Plus

```
Query: 2  tctaawacgaccaagctwawwttgcttgattcacttcwwyaagag 46
```

||||| ||||||||||| | ||||||||||||||| |||||
Sbjct: 1 tctaaaacgaccaagctaatattgcttgattcacttctacaagag 45

>JZ757041|JZ757041.1 awis5P0006D06_F.ab1 Sea ice eukaryotic metatranscriptome Weddell Sea 2 uncultured organism cDNA, mRNA sequence. Length = 628; score = 68.8 bits (31), expect = 2e-09; identities = 38/45 (84%); strand = Plus / Plus
Query: 2 tctaawacgaccaagctwawwttgcttgattcacttcwwyaagag 46
||||| ||||||||||| | ||||||||||||||| |||||
Sbjct: 1 tctaaaacgaccaagctaatattgcttgattcacttctacaagag 45

>JZ756818|JZ756818.1 awis5P0005I09_F.ab1 Sea ice eukaryotic metatranscriptome Weddell Sea 2 uncultured organism cDNA, mRNA sequence. Length = 503; score = 68.8 bits (31), expect = 2e-09; identities = 38/45 (84%); strand = Plus / Plus
Query: 2 tctaawacgaccaagctwawwttgcttgattcacttcwwyaagag 46
||||| ||||||||||| | ||||||||||||||| |||||
Sbjct: 1 tctaaaacgaccaagctaatattgcttgattcacttctacaagag 45

>JZ756648|JZ756648.1 awis5P0004P22_F.ab1 Sea ice eukaryotic metatranscriptome Weddell Sea 2 uncultured organism cDNA, mRNA sequence. Length = 681; score = 68.8 bits (31), expect = 2e-09; identities = 38/45 (84%); strand = Plus / Plus
Query: 2 tctaawacgaccaagctwawwttgcttgattcacttcwwyaagag 46
||||| ||||||||||| | ||||||||||||||| |||||
Sbjct: 1 tctaaaacgaccaagctaatattgcttgattcacttctacaagag 45

>JZ756141|JZ756141.1 awis5P0003G13_F.ab1 Sea ice eukaryotic metatranscriptome Weddell Sea 2 uncultured organism cDNA, mRNA sequence. Length = 634; score = 68.8 bits (31), expect = 2e-09; identities = 38/45 (84%); strand = Plus / Plus
Query: 2 tctaawacgaccaagctwawwttgcttgattcacttcwwyaagag 46
||||| ||||||||||| | ||||||||||||||| |||||
Sbjct: 1 tctaaaacgaccaagctaatattgcttgattcacttctacaagag 45

>JZ755703|JZ755703.1 awis5P0002A19_F.ab1 Sea ice eukaryotic metatranscriptome Weddell Sea 2 uncultured organism cDNA, mRNA sequence. Length = 636; score = 68.8 bits (31), expect = 2e-09; identities = 38/45 (84%); strand = Plus / Plus
Query: 2 tctaawacgaccaagctwawwttgcttgattcacttcwwyaagag 46
||||| ||||||||||| | ||||||||||||||| |||||
Sbjct: 1 tctaaaacgaccaagctaatattgcttgattcacttctacaagag 45

>JZ755442|JZ755442.1 awis5P0001E02_F.ab1 Sea ice eukaryotic metatranscriptome Weddell Sea 2 uncultured organism cDNA, mRNA sequence. Length = 600; score = 68.8 bits (31), expect = 2e-09; identities = 38/45 (84%); strand = Plus / Plus
Query: 2 tctaawacgaccaagctwawwttgcttgattcacttcwwyaagag 46
||||| ||||||||||| | ||||||||||||||| |||||
Sbjct: 1 tctaaaacgaccaagctaatattgcttgattcacttctacaagag 45

>JZ760213|JZ760213.1 awis5P0020B21_F.ab1 Sea ice eukaryotic metatranscriptome Weddell Sea 2 uncultured organism cDNA, mRNA sequence. Length = 633; score = 59.9 bits (27), expect = 9e-07; identities = 37/45 (82%); strand = Plus / Plus
Query: 2 tctaawacgaccaagctwawwttgcttgattcacttcwwyaagag 46

          ||||| ||||||||||| ||||||||||||||| |||||
Sbjct: 1 tctaaaacgaccaagcttttattgcttgattcacttctacaagag 45

>JZ755909|JZ755909.1 awis5P0002K18_F.ab1 Sea ice eukaryotic metatranscriptome Weddell Sea 2 uncultured organism cDNA, mRNA sequence. Length = 645; score = 59.9 bits (27), expect = 9e-07; identities = 37/45 (82%); strand = Plus / Plus

Query: 2 tctaawacgaccaagctwawwttgcttgattcacttcwwyaagag 46
          ||||| ||||||||||| ||||||||||||||| |||||
Sbjct: 1 tctaaaacgaccaagcttttattgcttgattcacttctacaagag 45

>JZ755697|JZ755697.1 awis5P0002A11_F.ab1 Sea ice eukaryotic metatranscriptome Weddell Sea 2 uncultured organism cDNA, mRNA sequence. Length = 523; score = 59.9 bits (27), expect = 9e-07; identities = 37/45 (82%); strand = Plus / Plus

Query: 2 tctaawacgaccaagctwawwttgcttgattcacttcwwyaagag 46
          ||||| ||||||||||| ||||||||||||||| |||||
Sbjct: 1 tctaaaacgaccaagcttttattgcttgattcacttctacaagag 45

>JZ752877|JZ752877.1 awig5P0012L04_F.ab1 Sea ice eukaryotic metatranscriptome Weddell Sea 1 uncultured organism cDNA, mRNA sequence. Length = 580; score = 59.9 bits (27), expect = 9e-07; identities = 37/45 (82%); strand = Plus / Plus

Query: 2 tctaawacgaccaagctwawwttgcttgattcacttcwwyaagag 46
          ||||| ||||||||||| ||||||||||||||| |||||
Sbjct: 1 tctaaaacgaccaagcttttattgcttgattcacttctacaagag 45

>FK670511|FK670511.1 CF_W00_99D11_SP6 Copepod Whole Organism, Normalized *Calanus finmarchicus* cDNA clone CF_W00_99D11; 5' similar to ref|NP_722858.1| CG31694 CG31694-PA - Drosophila melanogaster. Score = 97.4 bits (241), E. Length = 664; score = 55.5 bits (25), expect = 2e-05; identities = 31/37 (83%); strand = Plus / Plus

Query: 10 gaccaagctwawwttgcttgattcacttcwwyaagag 46
          ||||||||| | ||||||||||||||| |||||
Sbjct: 1 gaccaagctaatattgcttgattcacttctacaagag 37

>FG985503|FG985503.1 CF_W00_81G05_SP6 Copepod Whole Organism, Normalized *Calanus finmarchicus* cDNA clone CF_W00_81G05; 5' similar to ref|XP_967788.1| DIVDICTED: similar to 26S protease regulatory subunit S10b. Score = 352 Length = 655; score = 55.5 bits (25), expect = 2e-05; identities = 31/37 (83%); strand = Plus / Plus

Query: 10 gaccaagctwawwttgcttgattcacttcwwyaagag 46
          ||||||||| | ||||||||||||||| |||||
Sbjct: 3 gaccaagctaatattgcttgattcacttctacaagag 39

>EL773798|EL773798.1 CF_W00_40c05_SP6 Copepod Whole Organism, Normalized *Calanus finmarchicus* cDNA clone CF_W00_40c05; 5' similar to ref|XP_787033.2| DIVDICTED: similar to myosin light chain kinase [Strongylocentrotus. Sco Length = 663; score = 55.5 bits (25), expect = 2e-05; identities = 31/37 (83%); strand = Plus / Plus

Query: 10 gaccaagctwawwttgcttgattcacttcwwyaagag 46
          ||||||||| | ||||||||||||||| |||||

```
Sbjct: 1  gaccaagctaatattgcttgattcacttctacaagag 37
```

>EH666896|EH666896.1 CF_W00_07g07_SP6 Copepod Whole Organism, Normalized *Calanus finmarchicus* cDNA clone CF_W00_07g07; 5' similar to ref|XP_397345.2| DIVDICTED: similar to karmoisin CG12286-PA - Apis mellifera. Score = 85. Length = 538; score = 55.5 bits (25), expect = 2e-05; identities = 31/37 (83%); strand = Plus / Plus

```
Query: 10 gaccaagctwawwttgcttgattcacttcwwyaagag 46
          ||||||||| |  |||||||||||||||   |||||
Sbjct: 3  gaccaagctaatattgcttgattcacttctacaagag 39
```

>FG985387|FG985387.1 CF_W00_80D08_SP6 Copepod Whole Organism, Normalized *Calanus finmarchicus* cDNA clone CF_W00_80D08; 5', mRNA sequence. Length = 372; score = 53.3 bits (24), expect = 9e-05; identities = 30/36 (83%); strand = Plus / Plus

```
Query: 11 accaagctwawwttgcttgattcacttcwwyaagag 46
          |||||||| |  |||||||||||||||   |||||
Sbjct: 3  accaagctaatattgcttgattcacttctacaagag 38
```

>ES237671|ES237671.1 CF_W00_58g07_SP6 Copepod Whole Organism, Normalized *Calanus finmarchicus* cDNA clone CF_W00_58g07; 5' similar to ref|XP_974958.1| DIVDICTED: similar to CG6320-PD, isoform D - Tribolium castaneum. Score Length = 659; score = 53.3 bits (24), expect = 9e-05; identities = 30/36 (83%); strand = Plus / Plus

```
Query: 11 accaagctwawwttgcttgattcacttcwwyaagag 46
          |||||||| |  |||||||||||||||   |||||
Sbjct: 1  accaagctaatattgcttgattcacttctacaagag 36
```

>EL965951|EL965951.1 CF_W00_51e05_SP6 Copepod Whole Organism, Normalized *Calanus finmarchicus* cDNA clone CF_W00_51e05; 5' similar to ref|XP_623554.2| DIVDICTED: similar to G protein-coupled receptor 89 [Apis. Score = 274 b Length = 680; score = 53.3 bits (24), expect = 9e-05; identities = 30/36 (83%); strand = Plus / Plus

```
Query: 11 accaagctwawwttgcttgattcacttcwwyaagag 46
          |||||||| |  |||||||||||||||   |||||
Sbjct: 2  accaagctaatattgcttgattcacttctacaagag 37
```

>EL585516|EL585516.1 CF_W00_12c05_SP6 Copepod Whole Organism, Normalized *Calanus finmarchicus* cDNA clone CF_W00_12c05; 5' similar to O73723 Actin-like protein 3 (Actin-related protein 3). Score=320.09, Expect=4.80E-86, mRN Length = 712; score = 53.3 bits (24), expect = 9e-05; identities = 30/36 (83%); strand = Plus / Plus

```
Query: 11 accaagctwawwttgcttgattcacttcwwyaagag 46
          |||||||| |  |||||||||||||||   |||||
Sbjct: 1  accaagctaatattgcttgattcacttctacaagag 36
```

>JZ758795|JZ758795.1 awis5P0013L03_F.ab1 Sea ice eukaryotic metatranscriptome Weddell Sea 2 uncultured organism cDNA, mRNA sequence. Length = 709; score = 51.1 bits (23), expect = 4e-04; identities = 36/45 (80%); strand = Plus / Plus

Query: 2 tctaawacgaccaagctwawwttgcttgattcacttcwwyaagag 46
         ||||| ||||||||||| |||||||| ||||||| |||||
Sbjct: 1 tctaaaacgaccaagcttttattgcttgactcacttctacaagag 45

>JZ752592|JZ752592.1 awig5P0011N19_F.ab1 Sea ice eukaryotic metatranscriptome Weddell Sea 1 uncultured organism cDNA, mRNA sequence. Length = 711; score = 51.1 bits (23), expect = 4e-04; identities = 36/45 (80%); strand = Plus / Plus
Query: 2 tctaawacgaccaagctwawwttgcttgattcacttcwwyaagag 46
         ||||| ||||||||||| |||| ||||||||||| |||||
Sbjct: 1 tctaaaacgaccaagcttttattgcctgattcacttctacaagag 45

>JZ533079|JZ533079.1 SSH_SKE5_G3_T7_ab1 Cal SSH_SKE_RHO 2d *Calanus helgolandicus* cDNA clone SSH_SKE5_G3_T7_ab1 similar to 40s ribosomal protein s4, mRNA sequence. Length = 369; score = 51.1 bits (23), expect = 4e-04; identities = 29/35 (82%); strand = Plus / Plus
Query: 12 ccaagctwawwttgcttgattcacttcwwyaagag 46
         ||||||| | |||||||||||||||| |||||
Sbjct: 1 ccaagctaatattgcttgattcacttctacaagag 35

>JZ532696|JZ532696.1 SSH_SKE10_F4_T7_ab1 Cal SSH_SKE_RHO 2d *Calanus helgolandicus* cDNA clone SSH_SKE10_F4_T7_ab1 similar to 40s ribosomal protein s4, mRNA sequence. Length = 531; score = 51.1 bits (23), expect = 4e-04; identities = 29/35 (82%); strand = Plus / Plus

Query: 12 ccaagctwawwttgcttgattcacttcwwyaagag 46
           ||||||| | |||||||||||||||| |||||
Sbjct: 211 ccaagctaatattgcttgattcacttctacaagag 245

>FK041321|FK041321.1 CF_WO0_93A06_SP6 Copepod Whole Organism, Normalized *Calanus finmarchicus* cDNA clone CF_WO0_93A06; 5', mRNA sequence. Length = 727; score = 51.1 bits (23), expect = 4e-04; identities = 29/35 (82%); strand = Plus / Plus
Query: 12 ccaagctwawwttgcttgattcacttcwwyaagag 46
         ||||||| | |||||||||||||||| |||||
Sbjct: 3 ccaagctaatattgcttgattcacttctacaagag 37

>FG632996|FG632996.1 CF_WO0_76c05_SP6 Copepod Whole Organism, Normalized *Calanus finmarchicus* cDNA clone CF_WO0_76c05; 5', mRNA sequence. Length = 647; score = 51.1 bits (23), expect = 4e-04; identities = 29/35 (82%); strand = Plus / Plus
Query: 12 ccaagctwawwttgcttgattcacttcwwyaagag 46
         ||||||| | |||||||||||||||| |||||
Sbjct: 3 ccaagctaatattgcttgattcacttctacaagag 37

>FG342679|FG342679.1 CF_WO0_66e10_SP6 Copepod Whole Organism, Normalized *Calanus finmarchicus* cDNA clone CF_WO0_66e10; 5' similar to ref|XP_311433.4| AGAP010719-PA -Anopheles gambiae str. PEST. Score = 270 bits (689), Exp Length = 545; score = 51.1 bits (23), expect = 4e-04; identities = 29/35 (82%); strand = Plus / Plus
Query: 12 ccaagctwawwttgcttgattcacttcwwyaagag 46
         ||||||| | |||||||||||||||| |||||
Sbjct: 3 ccaagctaatattgcttgattcacttctacaagag 37

>EL965582|EL965582.1 CF_W00_47e11_SP6 Copepod Whole Organism, Normalized *Calanus finmarchicus* cDNA clone CF_W00_47e11; 5' similar to ref|XP_595830.3| DIVDICTED: hypothetical protein - Bos taurus. Score = 309 bits (791), Ex Length = 661; score = 51.1 bits (23), expect = 4e-04; identities = 29/35 (82%); strand = Plus / Plus

```
Query: 12 ccaagctwawwttgcttgattcacttcwwyaagag 46
          ||||||| |  |||||||||||||||   |||||
Sbjct: 3  ccaagctaatattgcttgattcacttctacaagag 37
```

>EL774038|EL774038.1 CF_W00_42g12_SP6 Copepod Whole Organism, Normalized *Calanus finmarchicus* cDNA clone CF_W00_42g12; 5', mRNA sequence. Length = 577; score = 51.1 bits (23), expect = 4e-04; identities = 29/35 (82%); strand = Plus / Plus

```
Query: 12 ccaagctwawwttgcttgattcacttcwwyaagag 46
          ||||||| |  |||||||||||||||   |||||
Sbjct: 3  ccaagctaatattgcttgattcacttctacaagag 37
```

>EL773743|EL773743.1 CF_W00_39f07_SP6 Copepod Whole Organism, Normalized *Calanus finmarchicus* cDNA clone CF_W00_39f07; 5' similar to ref|XP_625099.1| DIVDICTED: similar to Tetraspanin 39D CG8666-PA - Apis mellifera. Score Length = 698; score = 51.1 bits (23), expect = 4e-04; identities = 29/35 (82%); strand = Plus / Plus

```
Query: 12 ccaagctwawwttgcttgattcacttcwwyaagag 46
          ||||||| |  |||||||||||||||   |||||
Sbjct: 1  ccaagctaatattgcttgattcacttctacaagag 35
```

>EL696897|EL696897.1 CF_W00_28c05_SP6 Copepod Whole Organism, Normalized *Calanus finmarchicus* cDNA clone CF_W00_28c05; 5' similar to ref|XP_001363602.1| DIVDICTED: similar to casein kinase alpha subunit [Monodelphis. Score Length = 730; score = 51.1 bits (23), expect = 4e-04; identities = 29/35 (82%); strand = Plus / Plus

```
Query: 12 ccaagctwawwttgcttgattcacttcwwyaagag 46
          ||||||| |  |||||||||||||||   |||||
Sbjct: 1  ccaagctaatattgcttgattcacttctacaagag 35
```

>EL696854|EL696854.1 CF_W00_27g09_SP6 Copepod Whole Organism, Normalized *Calanus finmarchicus* cDNA clone CF_W00_27g09; 5' similar to ref|XP_783899.1| DIVDICTED: similar to Ribophorin II - Strongylocentrotus purpuratus. Length = 652; score = 51.1 bits (23), expect = 4e-04; identities = 29/35 (82%); strand = Plus / Plus

```
Query: 12 ccaagctwawwttgcttgattcacttcwwyaagag 46
          ||||||| |  |||||||||||||||   |||||
Sbjct: 3  ccaagctaatattgcttgattcacttctacaagag 37
```

>EL586220|EL586220.1 CF_W00_20a11_SP6 Copepod Whole Organism, Normalized *Calanus finmarchicus* cDNA clone CF_W00_20a11; 5' similar to XP_624697.1 26S proteasome non-ATPase regulatory subunit 6 (26S proteasome regulatory sub Length = 743; score = 51.1 bits (23), expect = 4e-04; identities = 29/35 (82%); strand = Plus / Plus

```
Query: 12 ccaagctwawwttgcttgattcacttcwwyaagag 46
          ||||||| |  |||||||||||||||   |||||
Sbjct: 1  ccaagctaatattgcttgattcacttctacaagag 35
```

>EL586072|EL586072.1 CF_W00_18d10_SP6 Copepod Whole Organism, Normalized *Calanus finmarchicus* cDNA clone CF_W00_18d10; 5' similar to XP_972905.1 Dolichyl-diphosphooligosaccharide-protein

glycosyltransferase 67 kDa subunit Length = 782; score = 51.1 bits (23), expect = 4e-04; identities = 29/35 (82%); strand = Plus / Plus

```
Query: 12 ccaagctwawwttgcttgattcacttcwwyaagag 46
          ||||||| |  ||||||||||||||||   |||||
Sbjct: 1  ccaagctaatattgcttgattcacttctacaagag 35
```

>EL585843|EL585843.1 CF_W00_15h09_SP6 Copepod Whole Organism, Normalized *Calanus finmarchicus* cDNA clone CF_W00_15h09; 5’ similar to EAT36851.1 adducin [Aedes aegypti].Score=87.43, Expect=4.10E-16, mRNA sequence. Length = 633; score = 51.1 bits (23), expect = 4e-04; identities = 29/35 (82%); strand = Plus / Plus

```
Query: 12 ccaagctwawwttgcttgattcacttcwwyaagag 46
          ||||||| |  ||||||||||||||||   |||||
Sbjct: 3  ccaagctaatattgcttgattcacttctacaagag 37
```

>EL585483|EL585483.1 CF_W00_11h06_SP6 Copepod Whole Organism, Normalized *Calanus finmarchicus* cDNA clone CF_W00_11h06; 5’ similar to NP_054768.2 acyl-Coenzyme A dehydrogenase family, member 9 [Homo sapiens]. Score=84.34, E. Length = 676; score = 51.1 bits (23), expect = 4e-04; identities = 29/35 (82%); strand = Plus / Plus

```
Query: 12 ccaagctwawwttgcttgattcacttcwwyaagag 46
          ||||||| |  ||||||||||||||||   |||||
Sbjct: 3  ccaagctaatattgcttgattcacttctacaagag 37
```

>EH667167|EH667167.1 CF_W00_10g04_SP6 Copepod Whole Organism, Normalized *Calanus finmarchicus* cDNA clone CF_W00_10g04; 5’ similar to emb|CAH90615.1| hypothetical protein - Pongo pygmaeus. Score = 181 bits (460), expect = 2 Length = 705; score = 51.1 bits (23), expect = 4e-04; identities = 29/35 (82%); strand = Plus / Plus

```
Query: 12 ccaagctwawwttgcttgattcacttcwwyaagag 46
          ||||||| |  ||||||||||||||||   |||||
Sbjct: 3  ccaagctaatattgcttgattcacttctacaagag 37
```

>EH667127|EH667127.1 CF_W00_10c10_SP6 Copepod Whole Organism, Normalized *Calanus finmarchicus* cDNA clone CF_W00_10c10; 5’, mRNA sequence. Length = 701; score = 51.1 bits (23), expect = 4e-04; identities = 29/35 (82%); strand = Plus / Plus

```
Query: 12 ccaagctwawwttgcttgattcacttcwwyaagag 46
          ||||||| |  ||||||||||||||||   |||||
Sbjct: 3  ccaagctaatattgcttgattcacttctacaagag 37
```

>JZ533381|JZ533381.1 SSH_RH01_F9_T7_ab1 Cal SSH_SKE_RHO 2d *Calanus helgolandicus* cDNA clone SSH_RH01_F9_T7_ab1 similar to ribosomal protein s5, mRNA sequence. Length = 219; score = 48.9 bits (22), expect = 0.002; identities = 28/34 (82%); strand = Plus / Minus

```
Query: 13  caagctwawwttgcttgattcacttcwwyaagag 46
           |||||| |  ||||||||||||||||   |||||
Sbjct: 219 caagctaatattgcttgattcacttctacaagag 186
```

>BW778077|BW778077.1 Branchiostoma floridae cDNA, gastrula clone: bfga048h24, 5’ end. Length = 327; score = 48.9 bits (22), expect = 0.002; identities = 28/34 (82%); strand = Plus / Plus

```
Query: 13 caagctwawwttgcttgattcacttcwwyaagag 46
          |||||| |  ||||||||||||||||   |||||
Sbjct: 1  caagctaatattgcttgattcacttctacaagag 34
```

>BW777469|BW777469.1 Branchiostoma floridae cDNA, gastrula clone:bfga045p12, 5' end. Length = 461; score = 48.9 bits (22), expect = 0.002; identities = 28/34 (82%); strand = Plus / Plus

```
Query: 13 caagctwawwttgcttgattcacttcwwyaagag 46
          |||||| |  |||||||||||||||   |||||
Sbjct: 1  caagctaatattgcttgattcacttctacaagag 34
```

>GR411243|GR411243.1 CF_W01_59d03_SP6 Copepod Whole Organism, Normalized *Calanus finmarchicus* cDNA clone CF_W01_59d03; 5' , mRNA sequence. Length = 651; score = 48.9 bits (22), expect = 0.002; identities = 28/34 (82%); strand = Plus / Plus

```
Query: 13 caagctwawwttgcttgattcacttcwwyaagag 46
          |||||| |  |||||||||||||||   |||||
Sbjct: 1  caagctaatattgcttgattcacttctacaagag 34
```

>FK868340|FK868340.1 CF_W01_20D07_SP6 Copepod Whole Organism, Normalized *Calanus finmarchicus* cDNA clone CF_W01_20D07; 5' , mRNA sequence. Length = 729; score = 48.9 bits (22), expect = 0.002; identities = 28/34 (82%); strand = Plus / Plus

```
Query: 13 caagctwawwttgcttgattcacttcwwyaagag 46
          |||||| |  |||||||||||||||   |||||
Sbjct: 3  caagctaatattgcttgattcacttctacaagag 36
```

>FK868094|FK868094.1 CF_W01_16G02_SP6 Copepod Whole Organism, Normalized *Calanus finmarchicus* cDNA clone CF_W01_16G02; 5' similar to gb|EDV35017.1| GF22422 - Drosophila ananassae. Score = 233 bits (595), expect = 7e-60, mR Length = 675; score = 48.9 bits (22), expect = 0.002; identities = 28/34 (82%); strand = Plus / Plus

```
Query: 13 caagctwawwttgcttgattcacttcwwyaagag 46
          |||||| |  |||||||||||||||   |||||
Sbjct: 1  caagctaatattgcttgattcacttctacaagag 34
```

>FK868036|FK868036.1 CF_W01_16A10_SP6 Copepod Whole Organism, Normalized *Calanus finmarchicus* cDNA clone CF_W01_16A10; 5' , mRNA sequence. Length = 684; score = 48.9 bits (22), expect = 0.002; identities = 28/34 (82%); strand = Plus / Plus

```
Query: 13 caagctwawwttgcttgattcacttcwwyaagag 46
          |||||| |  |||||||||||||||   |||||
Sbjct: 3  caagctaatattgcttgattcacttctacaagag 36
```

>FK670397|FK670397.1 CF_W00_98A01_SP6 Copepod Whole Organism, Normalized *Calanus finmarchicus* cDNA clone CF_W00_98A01; 5' similar to ref|NP_001096540.1| hypothetical protein LOC100125184 - Xenopus tropicalis. Score = 184 b Length = 737; score = 48.9 bits (22), expect = 0.002; identities = 28/34 (82%); strand = Plus / Plus

```
Query: 13 caagctwawwttgcttgattcacttcwwyaagag 46
          |||||| |  |||||||||||||||   |||||
Sbjct: 36 caagctaatattgcttgattcacttctacaagag 69
```

>FK041641|FK041641.1 CF_W00_96F06_SP6 Copepod Whole Organism, Normalized *Calanus finmarchicus* cDNA clone CF_W00_96F06; 5' similar to ref|XP_001607504.1| DIVDICTED: similar to nad dehydrogenase - Nasonia vitripennis. Score Length = 641; score = 48.9 bits (22), expect = 0.002; identities = 28/34 (82%); strand = Plus / Plus

```
Query: 13 caagctwawwttgcttgattcacttcwwyaagag 46
```

        |||||| | ||||||||||||||| |||||
Sbjct: 3 caagctaatattgcttgattcacttctacaagag 36

>FK041182|FK041182.1 CF_W00_91E07_SP6 Copepod Whole Organism, Normalized *Calanus finmarchicus* cDNA clone CF_W00_91E07; 5' similar to ref|XP_001742833.1| predicted protein - Monosiga brevicollis MX1. Score = 181 bits (458), Length = 650; score = 48.9 bits (22), expect = 0.002; identities = 28/34 (82%); strand = Plus / Plus
Query: 13 caagctwawwttgcttgattcacttcwwyaagag 46
        |||||| | ||||||||||||||| |||||
Sbjct: 3 caagctaatattgcttgattcacttctacaagag 36

>FK040996|FK040996.1 CF_W00_89B06_SP6 Copepod Whole Organism, Normalized *Calanus finmarchicus* cDNA clone CF_W00_89B06; 5' similar to ref|XP_001605831.1| DIVDICTED: similar to aspartate aminotransferase [Nasonia. Score = 21 Length = 680; score = 48.9 bits (22), expect = 0.002; identities = 28/34 (82%); strand = Plus / Plus
Query: 13 caagctwawwttgcttgattcacttcwwyaagag 46
        |||||| | ||||||||||||||| |||||
Sbjct: 1 caagctaatattgcttgattcacttctacaagag 34

>FK040831|FK040831.1 CF_W00_87B12_SP6 Copepod Whole Organism, Normalized *Calanus finmarchicus* cDNA clone CF_W00_87B12; 5' similar to ref|XP_623819.1| DIVDICTED: similar to MLF1-adaptor molecule CG1098-PA [Apis. Score = 112 Length = 394; score = 48.9 bits (22), expect = 0.002; identities = 28/34 (82%); strand = Plus / Plus
Query: 13 caagctwawwttgcttgattcacttcwwyaagag 46
        |||||| | ||||||||||||||| |||||
Sbjct: 6 caagctaatattgcttgattcacttctacaagag 39

>FG342789|FG342789.1 CF_W00_67g09_SP6 Copepod Whole Organism, Normalized *Calanus finmarchicus* cDNA clone CF_W00_67g09; 5', mRNA sequence. Length = 382; score = 48.9 bits (22), expect = 0.002; identities = 28/34 (82%); strand = Plus / Plus
Query: 13 caagctwawwttgcttgattcacttcwwyaagag 46
        |||||| | ||||||||||||||| |||||
Sbjct: 3 caagctaatattgcttgattcacttctacaagag 36

>FG342348|FG342348.1 CF_W00_62g11_SP6 Copepod Whole Organism, Normalized *Calanus finmarchicus* cDNA clone CF_W00_62g11; 5' similar to ref|XP_968685.1| DIVDICTED: similar to CG8553-PA, isoform A - Tribolium castaneum. Score Length = 628; score = 48.9 bits (22), expect = 0.002; identities = 28/34 (82%); strand = Plus / Plus
Query: 13 caagctwawwttgcttgattcacttcwwyaagag 46
        |||||| | ||||||||||||||| |||||
Sbjct: 3 caagctaatattgcttgattcacttctacaagag 36

>FG342196|FG342196.1 CF_W00_61b12_SP6 Copepod Whole Organism, Normalized *Calanus finmarchicus* cDNA clone CF_W00_61b12; 5' similar to ref|XP_321739.3| AGAP001396-PA - Anopheles gambiae str. PEST. Score = 176 bits (447), Exp Length = 730; score = 48.9 bits (22), expect = 0.002; identities = 28/34 (82%); strand = Plus / Plus
Query: 13 caagctwawwttgcttgattcacttcwwyaagag 46
        |||||| | ||||||||||||||| |||||
Sbjct: 1 caagctaatattgcttgattcacttctacaagag 34

>ES237496|ES237496.1 CF_W00_56h07_SP6 Copepod Whole Organism, Normalized *Calanus finmarchicus* cDNA clone CF_W00_56h07; 5’ similar to ref|XP_969882.1| DIVDICTED: similar to aldehyde dehydrogenase 7 family, member A1. Score Length = 716; score = 48.9 bits (22), expect = 0.002; identities = 28/34 (82%); strand = Plus / Plus

```
Query: 13 caagctwawwttgcttgattcacttcwwyaagag 46
          |||||| |  ||||||||||||||||   |||||
Sbjct: 3  caagctaatattgcttgattcacttctacaagag 36
```

>EL966247|EL966247.1 CF_W00_54g01_SP6 Copepod Whole Organism, Normalized *Calanus finmarchicus* cDNA clone CF_W00_54g01; 5’ , mRNA sequence. Length = 683; score = 48.9 bits (22), expect = 0.002; identities = 28/34 (82%); strand = Plus / Plus

```
Query: 13 caagctwawwttgcttgattcacttcwwyaagag 46
          |||||| |  ||||||||||||||||   |||||
Sbjct: 3  caagctaatattgcttgattcacttctacaagag 36
```

>EL965899|EL965899.1 CF_W00_50h11_SP6 Copepod Whole Organism, Normalized *Calanus finmarchicus* cDNA clone CF_W00_50h11; 5’ , mRNA sequence. Length = 689; score = 48.9 bits (22), expect = 0.002; identities = 28/34 (82%); strand = Plus / Plus

```
Query: 13 caagctwawwttgcttgattcacttcwwyaagag 46
          |||||| |  ||||||||||||||||   |||||
Sbjct: 3  caagctaatattgcttgattcacttctacaagag 36
```

>EL965852|EL965852.1 CF_W00_50d12_SP6 Copepod Whole Organism, Normalized *Calanus finmarchicus* cDNA clone CF_W00_50d12; 5’ similar to ref|NP_609299.1| CG4389-PA, isoform A - Drosophila melanogaster. Score = 247 bits (630), Length = 710; score = 48.9 bits (22), expect = 0.002; identities = 28/34 (82%); strand = Plus / Plus

```
Query: 13 caagctwawwttgcttgattcacttcwwyaagag 46
          |||||| |  ||||||||||||||||   |||||
Sbjct: 1  caagctaatattgcttgattcacttctacaagag 34
```

>EL965459|EL965459.1 CF_W00_46c07_SP6 Copepod Whole Organism, Normalized *Calanus finmarchicus* cDNA clone CF_W00_46c07; 5’ similar to ref|XP_973290.1| DIVDICTED: similar to CG1309-PA - Tribolium castaneum. Score = 132 bits Length = 681; score = 48.9 bits (22), expect = 0.002; identities = 28/34 (82%); strand = Plus / Plus

```
Query: 13 caagctwawwttgcttgattcacttcwwyaagag 46
          |||||| |  ||||||||||||||||   |||||
Sbjct: 3  caagctaatattgcttgattcacttctacaagag 36
```

>EL773494|EL773494.1 CF_W00_37a08_SP6 Copepod Whole Organism, Normalized *Calanus finmarchicus* cDNA clone CF_W00_37a08; 5’ similar to ref|XP_697740.2| DIVDICTED: hypothetical protein - Danio rerio. Score = 214 bits (546), E. Length = 717; score = 48.9 bits (22), expect = 0.002; identities = 28/34 (82%); strand = Plus / Plus

```
Query: 13 caagctwawwttgcttgattcacttcwwyaagag 46
          |||||| |  ||||||||||||||||   |||||
Sbjct: 1  caagctaatattgcttgattcacttctacaagag 34
```

>EL696910|EL696910.1 CF_W00_28d07_SP6 Copepod Whole Organism, Normalized *Calanus finmarchicus* cDNA clone CF_W00_28d07; 5’ similar to ref|XP_972968.1| DIVDICTED: similar to Leukotriene A-

4 hydrolase (LTA-4 hydrolase). Score Length = 749; score = 48.9 bits (22), expect = 0.002; identities = 28/34 (82%); strand = Plus / Plus

```
Query: 13 caagctwawwttgcttgattcacttcwwyaagag 46
          |||||| |  |||||||||||||||   |||||
Sbjct: 21 caagctaatattgcttgattcacttctacaagag 54
```

>EL586548|EL586548.1 CF_W00_23e12_SP6 Copepod Whole Organism, Normalized *Calanus finmarchicus* cDNA clone CF_W00_23e12; 5' similar to XP_780162.1 MGC143224 protein [Strongylocentrotus purpuratus]. Score=218.01, Expect=2.60E. Length = 714; score = 48.9 bits (22), expect = 0.002; identities = 28/34 (82%); strand = Plus / Plus

```
Query: 13 caagctwawwttgcttgattcacttcwwyaagag 46
          |||||| |  |||||||||||||||   |||||
Sbjct: 2  caagctaatattgcttgattcacttctacaagag 35
```

>EL586512|EL586512.1 CF_W00_23b08_SP6 Copepod Whole Organism, Normalized *Calanus finmarchicus* cDNA clone CF_W00_23b08; 5' similar to XP_318822.3 ENSANGP00000010217 [Anopheles gambiae str. PEST]. Score=270.78, Expect=3.60E- Length = 738; score = 48.9 bits (22), expect = 0.002; identities = 28/34 (82%); strand = Plus / Plus

```
Query: 13 caagctwawwttgcttgattcacttcwwyaagag 46
          |||||| |  |||||||||||||||   |||||
Sbjct: 3  caagctaatattgcttgattcacttctacaagag 36
```

>EL585988|EL585988.1 CF_W00_17e03_SP6 Copepod Whole Organism, Normalized *Calanus finmarchicus* cDNA clone CF_W00_17e03; 5' similar to NP_001072534.1 hypothetical protein LOC779989 [Xenopus tropicalis]. Score=224.17, Expect= Length = 639; score = 48.9 bits (22), expect = 0.002; identities = 28/34 (82%); strand = Plus / Plus

```
Query: 13 caagctwawwttgcttgattcacttcwwyaagag 46
          |||||| |  |||||||||||||||   |||||
Sbjct: 3  caagctaatattgcttgattcacttctacaagag 36
```

>EL585884|EL585884.1 CF_W00_16d03_SP6 Copepod Whole Organism, Normalized *Calanus finmarchicus* cDNA clone CF_W00_16d03; 5' similar to XP_757181.1 hypothetical protein UM01034.1 [Ustilago maydis 521]. Score=170.63, Expect=3. Length = 627; score = 48.9 bits (22), expect = 0.002; identities = 28/34 (82%); strand = Plus / Plus

```
Query: 13 caagctwawwttgcttgattcacttcwwyaagag 46
          |||||| |  |||||||||||||||   |||||
Sbjct: 3  caagctaatattgcttgattcacttctacaagag 36
```

>EL585862|EL585862.1 CF_W00_16b04_SP6 Copepod Whole Organism, Normalized *Calanus finmarchicus* cDNA clone CF_W00_16b04; 5' similar to XP_392962.1 Guanine nucleotide-binding protein subunit beta-like protein (Receptor of act Length = 465; score = 48.9 bits (22), expect = 0.002; identities = 28/34 (82%); strand = Plus / Plus

```
Query: 13 caagctwawwttgcttgattcacttcwwyaagag 46
          |||||| |  |||||||||||||||   |||||
Sbjct: 3  caagctaatattgcttgattcacttctacaagag 36
```

>EL585791|EL585791.1 CF_W00_15d03_SP6 Copepod Whole Organism, Normalized *Calanus finmarchicus* cDNA clone CF_W00_15d03; 5', mRNA sequence. Length = 703; score = 48.9 bits (22), expect = 0.002; identities = 28/34 (82%); strand = Plus / Plus

Query: 13 caagctwawwttgcttgattcacttcwwyaagag 46
          |||||| | |||||||||||||||| |||||
Sbjct: 3 caagctaatattgcttgattcacttctacaagag 36

>EL585618|EL585618.1 CF_W00_13e02_SP6 Copepod Whole Organism, Normalized *Calanus finmarchicus* cDNA clone CF_W00_13e02; 5' similar to CAE02816.1 OSJNBa0043A12.21 [Oryza sativa (japonica cultivar-group)]. Score=114.39, Expec Length = 658; score = 48.9 bits (22), expect = 0.002; identities = 28/34 (82%); strand = Plus / Plus
Query: 13 caagctwawwttgcttgattcacttcwwyaagag 46
          |||||| | |||||||||||||||| |||||
Sbjct: 1 caagctaatattgcttgattcacttctacaagag 34

>EL585576|EL585576.1 CF_W00_13a03_SP6 Copepod Whole Organism, Normalized *Calanus finmarchicus* cDNA clone CF_W00_13a03; 5' similar to AAP49384.1 glutamate dehydrogenase [Tigriopus californicus]. Score=280.8, Expect=3.20E-74 Length = 701; score = 48.9 bits (22), expect = 0.002; identities = 28/34 (82%); strand = Plus / Plus
Query: 13 caagctwawwttgcttgattcacttcwwyaagag 46
          |||||| | |||||||||||||||| |||||
Sbjct: 3 caagctaatattgcttgattcacttctacaagag 36

>EH667099|EH667099.1 CF_W00_10a06_SP6 Copepod Whole Organism, Normalized *Calanus finmarchicus* cDNA clone CF_W00_10a06; 5' similar to ref|XP_970856.1| DIVDICTED: similar to CG9140-PA - Tribolium castaneum. Score = 314 bits Length = 693; score = 48.9 bits (22), expect = 0.002; identities = 28/34 (82%); strand = Plus / Plus
Query: 13 caagctwawwttgcttgattcacttcwwyaagag 46
          |||||| | |||||||||||||||| |||||
Sbjct: 3 caagctaatattgcttgattcacttctacaagag 36

>EH666513|EH666513.1 CF_W00_03f04_SP6 Copepod Whole Organism, Normalized *Calanus finmarchicus* cDNA clone CF_W00_03f04; 5' similar to ref|XP_417682.1| DIVDICTED: similar to mitochondrial solute carrier protein [Gallus. Scor Length = 631; score = 48.9 bits (22), expect = 0.002; identities = 28/34 (82%); strand = Plus / Plus
Query: 13 caagctwawwttgcttgattcacttcwwyaagag 46
          |||||| | |||||||||||||||| |||||
Sbjct: 1 caagctaatattgcttgattcacttctacaagag 34

>EH666503|EH666503.1 CF_W00_03e06_SP6 Copepod Whole Organism, Normalized *Calanus finmarchicus* cDNA clone CF_W00_03e06; 5' similar to ref|XP_316638.3| ENSANGP00000010932 - Anopheles gambiae str. PEST. Score = 141 bits (356) Length = 676; score = 48.9 bits (22), expect = 0.002; identities = 28/34 (82%); strand = Plus / Plus
Query: 13 caagctwawwttgcttgattcacttcwwyaagag 46
          |||||| | |||||||||||||||| |||||
Sbjct: 3 caagctaatattgcttgattcacttctacaagag 36

>BW782010|BW782010.1 Branchiostoma floridae cDNA, gastrula clone:bfga040j16, 5' end. Length = 693; score = 46.7 bits (21), expect = 0.008; identities = 27/33 (81%); strand = Plus / Plus
Query: 14 aagctwawwttgcttgattcacttcwwyaagag 46
          ||||| | |||||||||||||||| |||||
Sbjct: 1 aagctaatattgcttgattcacttctacaagag 33

>FK867860|FK867860.1 CF_W01_13F03_SP6 Copepod Whole Organism, Normalized *Calanus finmarchicus* cDNA clone CF_W01_13F03; 5' similar to ref|XP_971811.1| DIVDICTED: similar to MGC79091 protein - Tribolium castaneum. Score = 71 Length = 661; score = 46.7 bits (21), expect = 0.008; identities = 27/33 (81%); strand = Plus / Plus

```
Query: 14 aagctwawwttgcttgattcacttcwwyaagag 46
          ||||| |  ||||||||||||||||   |||||
Sbjct: 1  aagctaatattgcttgattcacttctacaagag 33
```

>FK670449|FK670449.1 CF_W00_98E05_SP6 Copepod Whole Organism, Normalized *Calanus finmarchicus* cDNA clone CF_W00_98E05; 5' similar to ref|XP_001747618.1| predicted protein - Monosiga brevicollis MX1. Score = 119 bits (298), Length = 731; score = 46.7 bits (21), expect = 0.008; identities = 27/33 (81%); strand = Plus / Plus

```
Query: 14 aagctwawwttgcttgattcacttcwwyaagag 46
          ||||| |  ||||||||||||||||   |||||
Sbjct: 1  aagctaatattgcttgattcacttctacaagag 33
```

>FK041501|FK041501.1 CF_W00_95A07_SP6 Copepod Whole Organism, Normalized *Calanus finmarchicus* cDNA clone CF_W00_95A07; 5' similar to ref|XP_966947.1| DIVDICTED: similar to AGAP007687-PA isoform 1 - Tribolium castaneum. Sco Length = 783; score = 46.7 bits (21), expect = 0.008; identities = 27/33 (81%); strand = Plus / Plus

```
Query: 14 aagctwawwttgcttgattcacttcwwyaagag 46
          ||||| |  ||||||||||||||||   |||||
Sbjct: 3  aagctaatattgcttgattcacttctacaagag 35
```

>FG343071|FG343071.1 CF_W00_70h03_SP6 Copepod Whole Organism, Normalized *Calanus finmarchicus* cDNA clone CF_W00_70h03; 5' similar to ref|XP_966715.1| DIVDICTED: similar to CG9854-PA, isoform A - Tribolium castaneum. Score Length = 704; score = 46.7 bits (21), expect = 0.008; identities = 27/33 (81%); strand = Plus / Plus

```
Query: 14 aagctwawwttgcttgattcacttcwwyaagag 46
          ||||| |  ||||||||||||||||   |||||
Sbjct: 13 aagctaatattgcttgattcacttctacaagag 45
```

>FG342978|FG342978.1 CF_W00_69h03_SP6 Copepod Whole Organism, Normalized *Calanus finmarchicus* cDNA clone CF_W00_69h03; 5', mRNA sequence. Length = 613; score = 46.7 bits (21), expect = 0.008; identities = 27/33 (81%); strand = Plus / Plus

```
Query: 14 aagctwawwttgcttgattcacttcwwyaagag 46
          ||||| |  ||||||||||||||||   |||||
Sbjct: 3  aagctaatattgcttgattcacttctacaagag 35
```

>FG342232|FG342232.1 CF_W00_61e12_SP6 Copepod Whole Organism, Normalized *Calanus finmarchicus* cDNA clone CF_W00_61e12; 5' similar to dbj|BAE27597.1| unnamed protein product - Mus musculus. Score = 281 bits (718), expect = Length = 692; score = 46.7 bits (21), expect = 0.008; identities = 27/33 (81%); strand = Plus / Plus

```
Query: 14 aagctwawwttgcttgattcacttcwwyaagag 46
          ||||| |  ||||||||||||||||   |||||
Sbjct: 32 aagctaatattgcttgattcacttctacaagag 64
```

>FE575842|FE575842.1 CAXF9244.fwd Amphioxus Branchiostoma floridae unpublished cDNA library CAXF, gastrula whole animal Branchiostoma floridae cDNA clone CAXF9244 5', mRNA sequence. Length = 811; score = 46.7 bits (21), expect = 0.008; identities = 27/33 (81%); strand = Plus / Plus

```
Query: 14 aagctwawwttgcttgattcacttcwwyaagag 46
          ||||| |  |||||||||||||||   |||||
Sbjct: 1  aagctaatattgcttgattcacttctacaagag 33
```

>FE564053|FE564053.1 CAXF12510.fwd Amphioxus Branchiostoma floridae unpublished cDNA library CAXF, gastrula whole animal Branchiostoma floridae cDNA clone CAXF12510 5'-mRNA sequence. Length = 403; score = 46.7 bits (21), expect = 0.008; identities = 27/33 (81%); strand = Plus / Plus

```
Query: 14 aagctwawwttgcttgattcacttcwwyaagag 46
          ||||| |  |||||||||||||||   |||||
Sbjct: 1  aagctaatattgcttgattcacttctacaagag 33
```

>FE564052|FE564052.1 CAXF12510.rev Amphioxus Branchiostoma floridae unpublished cDNA library CAXF, gastrula whole animal Branchiostoma floridae cDNA clone CAXF12510 3', mRNA sequence. Length = 382; score = 46.7 bits (21), expect = 0.008; identities = 27/33 (81%); strand = Plus / Minus

```
Query: 14  aagctwawwttgcttgattcacttcwwyaagag 46
           ||||| |  |||||||||||||||   |||||
Sbjct: 382 aagctaatattgcttgattcacttctacaagag 350
```

>EL966043|EL966043.1 CF_W00_52e03_SP6 Copepod Whole Organism, Normalized *Calanus finmarchicus* cDNA clone CF_W00_52e03; 5' similar to ref|XP_792811.2| DIVDICTED: similar to E1-like protein [Strongylocentrotus. Score = 135 b Length = 707; score = 46.7 bits (21), expect = 0.008; identities = 27/33 (81%); strand = Plus / Plus

```
Query: 14 aagctwawwttgcttgattcacttcwwyaagag 46
          ||||| |  |||||||||||||||   |||||
Sbjct: 1  aagctaatattgcttgattcacttctacaagag 33
```

>EL965641|EL965641.1 CF_W00_48c01_SP6 Copepod Whole Organism, Normalized *Calanus finmarchicus* cDNA clone CF_W00_48c01; 5' similar to ref|XP_395098.3| DIVDICTED: similar to d4 CG2682-PA, isoform A - Apis mellifera. Score = Length = 631; score = 46.7 bits (21), expect = 0.008; identities = 27/33 (81%); strand = Plus / Plus

```
Query: 14 aagctwawwttgcttgattcacttcwwyaagag 46
          ||||| |  |||||||||||||||   |||||
Sbjct: 1  aagctaatattgcttgattcacttctacaagag 33
```

>EL965365|EL965365.1 CF_W00_45c06_SP6 Copepod Whole Organism, Normalized *Calanus finmarchicus* cDNA clone CF_W00_45c06; 5' similar to ref|NP_001072444.1| GalNAc transferase 10 - Xenopus tropicalis. Score = 149 bits (375), E. Length = 706; score = 46.7 bits (21), expect = 0.008; identities = 27/33 (81%); strand = Plus / Plus

```
Query: 14 aagctwawwttgcttgattcacttcwwyaagag 46
          ||||| |  |||||||||||||||   |||||
Sbjct: 1  aagctaatattgcttgattcacttctacaagag 33
```

>EL773723|EL773723.1 CF_WO0_39d11_SP6 Copepod Whole Organism, Normalized *Calanus finmarchicus* cDNA clone CF_WO0_39d11; 5' similar to ref|NP_001074214.1| diacylglycerol kinase gamma isoform 3 - Homo sapiens. Score = 116 bit Length = 724; score = 46.7 bits (21), expect = 0.008; identities = 27/33 (81%); strand = Plus / Plus

```
Query: 14 aagctwawwttgcttgattcacttcwwyaagag 46
          ||||| |  |||||||||||||||   |||||
Sbjct: 1  aagctaatattgcttgattcacttctacaagag 33
```

>EL697493|EL697493.1 CF_WO0_34e10_SP6 Copepod Whole Organism, Normalized *Calanus finmarchicus* cDNA clone CF_WO0_34e10; 5' , mRNA sequence. Length = 701; score = 46.7 bits (21), expect = 0.008; identities = 27/33 (81%); strand = Plus / Plus

```
Query: 14 aagctwawwttgcttgattcacttcwwyaagag 46
          ||||| |  |||||||||||||||   |||||
Sbjct: 1  aagctaatattgcttgattcacttctacaagag 33
```

>EL697378|EL697378.1 CF_WO0_33d02_SP6 Copepod Whole Organism, Normalized *Calanus finmarchicus* cDNA clone CF_WO0_33d02; 5' similar to ref|XP_970535.1| DIVDICTED: similar to CG17636-PA.3 - Tribolium castaneum. Score = 147 bi Length = 687; score = 46.7 bits (21), expect = 0.008; identities = 27/33 (81%); strand = Plus / Plus

```
Query: 14 aagctwawwttgcttgattcacttcwwyaagag 46
          ||||| |  |||||||||||||||   |||||
Sbjct: 1  aagctaatattgcttgattcacttctacaagag 33
```

>EL697099|EL697099.1 CF_WO0_30d08_SP6 Copepod Whole Organism, Normalized *Calanus finmarchicus* cDNA clone CF_WO0_30d08; 5' similar to ref|NP_001002360.1| hypothetical protein LOC436633 - Danio rerio. Score = 129 bits (325), Length = 686; score = 46.7 bits (21), expect = 0.008; identities = 27/33 (81%); strand = Plus / Plus

```
Query: 14 aagctwawwttgcttgattcacttcwwyaagag 46
          ||||| |  |||||||||||||||   |||||
Sbjct: 1  aagctaatattgcttgattcacttctacaagag 33
```

>EL696984|EL696984.1 CF_WO0_29c01_SP6 Copepod Whole Organism, Normalized *Calanus finmarchicus* cDNA clone CF_WO0_29c01; 5' similar to ref|XP_393540.2| DIVDICTED: similar to CG14224-PA - Apis mellifera. Score = 151 bits (382 Length = 716; score = 46.7 bits (21), expect = 0.008; identities = 27/33 (81%); strand = Plus / Plus

```
Query: 14 aagctwawwttgcttgattcacttcwwyaagag 46
          ||||| |  |||||||||||||||   |||||
Sbjct: 8  aagctaatattgcttgattcacttctacaagag 40
```

>EL696878|EL696878.1 CF_WO0_28a09_SP6 Copepod Whole Organism, Normalized *Calanus finmarchicus* cDNA clone CF_WO0_28a09; 5' similar to ref|NP_001012122.1| endothelial cell growth factor 1 (platelet-derived) [Rattus. Score = Length = 718; score = 46.7 bits (21), expect = 0.008; identities = 30/37 (81%); strand = Plus / Plus

```
Query: 10 gaccaagctwawwttgcttgattcacttcwwyaagag 46
          ||||||||| |  || |||||||||||||   |||||
Sbjct: 13 gaccaagctaatatttcttgattcacttctacaagag 49
```

>EL696715|EL696715.1 CF_WO0_26d01_SP6 Copepod Whole Organism, Normalized *Calanus finmarchicus* cDNA clone CF_WO0_26d01; 5' similar to ref|XP_623216.2| DIVDICTED: similar to Connector of

kinase to AP-1 CG7392-PA,. Score = 17 Length = 683; score = 46.7 bits (21), expect = 0.008; identities = 27/33 (81%); strand = Plus / Plus

```
Query: 14 aagctwawwttgcttgattcacttcwwyaagag 46
          ||||| |  |||||||||||||||   |||||
Sbjct: 1  aagctaatattgcttgattcacttctacaagag 33
```

>EL586590|EL586590.1 CF_W00_24a07_SP6 Copepod Whole Organism, Normalized *Calanus finmarchicus* cDNA clone CF_W00_24a07; 5' similar to XP_624812.2 CG5682-PA, partial [Apis mellifera]. Score=223.4, Expect=5.60E-57, mRNA seque Length = 677; score = 46.7 bits (21), expect = 0.008; identities = 27/33 (81%); strand = Plus / Plus

```
Query: 14 aagctwawwttgcttgattcacttcwwyaagag 46
          ||||| |  |||||||||||||||   |||||
Sbjct: 1  aagctaatattgcttgattcacttctacaagag 33
```

>EL585548|EL585548.1 CF_W00_12f08_SP6 Copepod Whole Organism, Normalized *Calanus finmarchicus* cDNA clone CF_W00_12f08; 5' , mRNA sequence. Length = 678; score = 46.7 bits (21), expect = 0.008; identities = 27/33 (81%); strand = Plus / Plus

```
Query: 14 aagctwawwttgcttgattcacttcwwyaagag 46
          ||||| |  |||||||||||||||   |||||
Sbjct: 2  aagctaatattgcttgattcacttctacaagag 34
```

>GR411269|GR411269.1 CF_W01_59g02_SP6 Copepod Whole Organism, Normalized *Calanus finmarchicus* cDNA clone CF_W01_59g02; 5' similar to gb|AC011426.1| COP9 signalosome complex subunit 2 - Caligus rogercresseyi. Score = 259 bi Length = 557; score = 44.5 bits (20), expect = 0.039; identities = 26/32 (81%); strand = Plus / Plus

```
Query: 15 agctwawwttgcttgattcacttcwwyaagag 46
          |||| |  |||||||||||||||   |||||
Sbjct: 1  agctaatattgcttgattcacttctacaagag 32
```

>GR411076|GR411076.1 CF_W01_57b04_SP6 Copepod Whole Organism, Normalized *Calanus finmarchicus* cDNA clone CF_W01_57b04; 5' , mRNA sequence. Length = 716; score = 44.5 bits (20), expect = 0.039; identities = 26/32 (81%); strand = Plus / Plus

```
Query: 15 agctwawwttgcttgattcacttcwwyaagag 46
          |||| |  |||||||||||||||   |||||
Sbjct: 2  agctaatattgcttgattcacttctacaagag 33
```

>FK868365|FK868365.1 CF_W01_20F09_SP6 Copepod Whole Organism, Normalized *Calanus finmarchicus* cDNA clone CF_W01_20F09; 5' , mRNA sequence. Length = 594; score = 44.5 bits (20), expect = 0.039; identities = 26/32 (81%); strand = Plus / Plus

```
Query: 15 agctwawwttgcttgattcacttcwwyaagag 46
          |||| |  |||||||||||||||   |||||
Sbjct: 3  agctaatattgcttgattcacttctacaagag 34
```

>FK868363|FK868363.1 CF_W01_20F07_SP6 Copepod Whole Organism, Normalized *Calanus finmarchicus* cDNA clone CF_W01_20F07; 5' , mRNA sequence. Length = 667; score = 44.5 bits (20), expect = 0.039; identities = 26/32 (81%); strand = Plus / Plus

```
Query: 15 agctwawwttgcttgattcacttcwwyaagag 46
          |||| |  |||||||||||||||   |||||
Sbjct: 3  agctaatattgcttgattcacttctacaagag 34
```

>FK868317|FK868317.1 CF_W01_20B08_SP6 Copepod Whole Organism, Normalized *Calanus finmarchicus* cDNA clone CF_W01_20B08; 5’ similar to ref|NP_001011072.1| F-box and leucine-rich repeat protein 5 - Xenopus tropicalis. Score = Length = 663; score = 44.5 bits (20), expect = 0.039; identities = 26/32 (81%); strand = Plus / Plus

```
Query: 15 agctwawwttgcttgattcacttcwwyaagag 46
          |||| |  |||||||||||||||   |||||
Sbjct: 3  agctaatattgcttgattcacttctacaagag 34
```

>FK868273|FK868273.1 CF_W01_19F05_SP6 Copepod Whole Organism, Normalized *Calanus finmarchicus* cDNA clone CF_W01_19F05; 5’ similar to ref|XP_796913.1| DIVDICTED: similar to Eukaryotic translation initiation factor 3,. Score Length = 627; score = 44.5 bits (20), expect = 0.039; identities = 26/32 (81%); strand = Plus / Plus

```
Query: 15 agctwawwttgcttgattcacttcwwyaagag 46
          |||| |  |||||||||||||||   |||||
Sbjct: 1  agctaatattgcttgattcacttctacaagag 32
```

>FK868025|FK868025.1 CF_W01_15H04_SP6 Copepod Whole Organism, Normalized *Calanus finmarchicus* cDNA clone CF_W01_15H04; 5’ similar to ref|XP_001649535.1| ral - Aedes aegypti. Score = 256 bits (653), expect = 1e-66, mRNA seq Length = 609; score = 44.5 bits (20), expect = 0.039; identities = 26/32 (81%); strand = Plus / Plus

```
Query: 15 agctwawwttgcttgattcacttcwwyaagag 46
          |||| |  |||||||||||||||   |||||
Sbjct: 1  agctaatattgcttgattcacttctacaagag 32
```

>FK867885|FK867885.1 CF_W01_13H06_SP6 Copepod Whole Organism, Normalized *Calanus finmarchicus* cDNA clone CF_W01_13H06; 5’ similar to ref|XP_975299.1| DIVDICTED: similar to chaperonin - Tribolium castaneum. Score = 289 bits Length = 624; score = 44.5 bits (20), expect = 0.039; identities = 26/32 (81%); strand = Plus / Plus

```
Query: 15 agctwawwttgcttgattcacttcwwyaagag 46
          |||| |  |||||||||||||||   |||||
Sbjct: 1  agctaatattgcttgattcacttctacaagag 32
```

>FK867745|FK867745.1 CF_W01_12C01_SP6 Copepod Whole Organism, Normalized *Calanus finmarchicus* cDNA clone CF_W01_12C01; 5’ similar to gb|ABU41071.1| receptor for activated protein kinase C-like protein. Score = 379 bits (97 Length = 661; score = 44.5 bits (20), expect = 0.039; identities = 26/32 (81%); strand = Plus / Plus

```
Query: 15 agctwawwttgcttgattcacttcwwyaagag 46
          |||| |  |||||||||||||||   |||||
Sbjct: 1  agctaatattgcttgattcacttctacaagag 32
```

>FK867560|FK867560.1 CF_W01_10A07_SP6 Copepod Whole Organism, Normalized *Calanus finmarchicus* cDNA clone CF_W01_10A07; 5' similar to ref|XP_623666.1| DIVDICTED: similar to CG5065-PA isoform 2 - Apis mellifera. Score = 192 Length = 733; score = 44.5 bits (20), expect = 0.039; identities = 26/32 (81%); strand = Plus / Plus

```
Query: 15 agctwawwttgcttgattcacttcwwyaagag 46
          |||| |  |||||||||||||||   |||||
Sbjct: 34 agctaatattgcttgattcacttctacaagag 65
```

>FK867515|FK867515.1 CF_W01_09E10_SP6 Copepod Whole Organism, Normalized *Calanus finmarchicus* cDNA clone CF_W01_09E10; 5' similar to gb|EDW70004.1| GJ11799 - Drosophila virilis. Score = 164 bits (416), expect = 3e-39, mRNA Length = 620; score = 44.5 bits (20), expect = 0.039; identities = 26/32 (81%); strand = Plus / Plus

```
Query: 15 agctwawwttgcttgattcacttcwwyaagag 46
          |||| |  |||||||||||||||   |||||
Sbjct: 1  agctaatattgcttgattcacttctacaagag 32
```

>FK671254|FK671254.1 CF_W01_08B01_SP6 Copepod Whole Organism, Normalized *Calanus finmarchicus* cDNA clone CF_W01_08B01; 5' similar to ref|XP_001948080.1| DIVDICTED: similar to CG5525 CG5525-PA - Acyrthosiphon pisum. Score = Length = 673; score = 44.5 bits (20), expect = 0.039; identities = 26/32 (81%); strand = Plus / Plus

```
Query: 15 agctwawwttgcttgattcacttcwwyaagag 46
          |||| |  |||||||||||||||   |||||
Sbjct: 1  agctaatattgcttgattcacttctacaagag 32
```

>FK671214|FK671214.1 CF_W01_07F02_SP6 Copepod Whole Organism, Normalized *Calanus finmarchicus* cDNA clone CF_W01_07F02; 5' similar to gb|EDV23485.1| hypothetical protein TRIADDRAFT_28052 - Trichoplax adhaerens. Score = 160 Length = 612; score = 44.5 bits (20), expect = 0.039; identities = 26/32 (81%); strand = Plus / Plus

```
Query: 15 agctwawwttgcttgattcacttcwwyaagag 46
          |||| |  |||||||||||||||   |||||
Sbjct: 3  agctaatattgcttgattcacttctacaagag 34
```

>FK670955|FK670955.1 CF_W01_04G01_SP6 Copepod Whole Organism, Normalized *Calanus finmarchicus* cDNA clone CF_W01_04G01; 5' similar to ref|XP_001854652.1| 60S ribosomal protein L5 - Culex pipiens quinquefasciatus. Score = 31 Length = 690; score = 44.5 bits (20), expect = 0.039; identities = 26/32 (81%); strand = Plus / Plus

```
Query: 15 agctwawwttgcttgattcacttcwwyaagag 46
          |||| |  |||||||||||||||   |||||
Sbjct: 3  agctaatattgcttgattcacttctacaagag 34
```

>FK670735|FK670735.1 CF_W01_02B09_SP6 Copepod Whole Organism, Normalized *Calanus finmarchicus* cDNA clone CF_W01_02B09; 5' similar to ref|XP_001850971.1| signal recognition particle 54

kda protein [Culex pipiens. Score = 32 Length = 604; score = 44.5 bits (20), expect = 0.039; identities = 26/32 (81%); strand = Plus / Plus

```
Query: 15 agctwawwttgcttgattcacttcwwyaagag 46
          |||| |  |||||||||||||||   |||||
Sbjct: 13 agctaatattgcttgattcacttctacaagag 44
```

>FK670674|FK670674.1 CF_W01_01E04_SP6 Copepod Whole Organism, Normalized *Calanus finmarchicus* cDNA clone CF_W01_01E04; 5' , mRNA sequence. Length = 662; score = 44.5 bits (20), expect = 0.039; identities = 26/32 (81%); strand = Plus / Plus

```
Query: 15 agctwawwttgcttgattcacttcwwyaagag 46
          |||| |  |||||||||||||||   |||||
Sbjct: 1  agctaatattgcttgattcacttctacaagag 32
```

>FK670448|FK670448.1 CF_W00_98E04_SP6 Copepod Whole Organism, Normalized *Calanus finmarchicus* cDNA clone CF_W00_98E04; 5' similar to ref|XP_001627933.1| predicted protein - Nematostella vectensis. Score = 110 bits (276), E. Length = 704; score = 44.5 bits (20), expect = 0.039; identities = 26/32 (81%); strand = Plus / Plus

```
Query: 15 agctwawwttgcttgattcacttcwwyaagag 46
          |||| |  |||||||||||||||   |||||
Sbjct: 3  agctaatattgcttgattcacttctacaagag 34
```

>FK041360|FK041360.1 CF_W00_93E01_SP6 Copepod Whole Organism, Normalized *Calanus finmarchicus* cDNA clone CF_W00_93E01; 5' similar to ref|XP_395332.2| DIVDICTED: similar to chromosome segregation 1-like [Apis. Score = 228 b Length = 653; score = 44.5 bits (20), expect = 0.039; identities = 26/32 (81%); strand = Plus / Plus

```
Query: 15 agctwawwttgcttgattcacttcwwyaagag 46
          |||| |  |||||||||||||||   |||||
Sbjct: 3  agctaatattgcttgattcacttctacaagag 34
```

>FK041173|FK041173.1 CF_W00_91D08_SP6 Copepod Whole Organism, Normalized *Calanus finmarchicus* cDNA clone CF_W00_91D08; 5' similar to ref|ZP_01694887.1| alkaline phosphatase D domain protein [Microscilla marina ATCC. Score Length = 708; score = 44.5 bits (20), expect = 0.039; identities = 26/32 (81%); strand = Plus / Plus

```
Query: 15 agctwawwttgcttgattcacttcwwyaagag 46
          |||| |  |||||||||||||||   |||||
Sbjct: 1  agctaatattgcttgattcacttctacaagag 32
```

>FK041149|FK041149.1 CF_W00_91B02_SP6 Copepod Whole Organism, Normalized *Calanus finmarchicus* cDNA clone CF_W00_91B02; 5' , mRNA sequence. Length = 724; score = 44.5 bits (20), expect = 0.039; identities = 26/32 (81%); strand = Plus / Plus

```
Query: 15 agctwawwttgcttgattcacttcwwyaagag 46
          |||| |  |||||||||||||||   |||||
Sbjct: 8  agctaatattgcttgattcacttctacaagag 39
```

>FK041102|FK041102.1 CF_WO0_90E09_SP6 Copepod Whole Organism, Normalized *Calanus finmarchicus* cDNA clone CF_WO0_90E09; 5' similar to gb|EDV49959.1| GG23811 - Drosophila erecta. Score = 106 bits (264), expect = 1e-21, mRNA Length = 608; score = 44.5 bits (20), expect = 0.039; identities = 26/32 (81%); strand = Plus / Plus

```
Query: 15 agctwawwttgcttgattcacttcwwyaagag 46
          |||| |  ||||||||||||||||   |||||
Sbjct: 3  agctaatattgcttgattcacttctacaagag 34
```

>FK040824|FK040824.1 CF_WO0_87B04_SP6 Copepod Whole Organism, Normalized *Calanus finmarchicus* cDNA clone CF_WO0_87B04; 5' similar to ref|XP_967617.1| DIVDICTED: similar to AGAP009119-PA - Tribolium castaneum. Score = 110 b Length = 650; score = 44.5 bits (20), expect = 0.039; identities = 26/32 (81%); strand = Plus / Plus

```
Query: 15 agctwawwttgcttgattcacttcwwyaagag 46
          |||| |  ||||||||||||||||   |||||
Sbjct: 3  agctaatattgcttgattcacttctacaagag 34
```

>FG985451|FG985451.1 CF_WO0_81B06_SP6 Copepod Whole Organism, Normalized *Calanus finmarchicus* cDNA clone CF_WO0_81B06; 5' similar to ref|XP_535180.2| DIVDICTED: similar to heat shock 70kDa protein 14 isoform 1 [Canis. Scor Length = 684; score = 44.5 bits (20), expect = 0.039; identities = 26/32 (81%); strand = Plus / Plus

```
Query: 15 agctwawwttgcttgattcacttcwwyaagag 46
          |||| |  ||||||||||||||||   |||||
Sbjct: 1  agctaatattgcttgattcacttctacaagag 32
```

>FG985377|FG985377.1 CF_WO0_80C10_SP6 Copepod Whole Organism, Normalized *Calanus finmarchicus* cDNA clone CF_WO0_80C10; 5' similar to gb|EDV35017.1| GF22422 - Drosophila ananassae. Score = 166 bits (419), expect = 1e-39, mR Length = 656; score = 44.5 bits (20), expect = 0.039; identities = 26/32 (81%); strand = Plus / Plus

```
Query: 15 agctwawwttgcttgattcacttcwwyaagag 46
          |||| |  ||||||||||||||||   |||||
Sbjct: 1  agctaatattgcttgattcacttctacaagag 32
```

>FG632523|FG632523.1 CF_WO0_71a12_SP6 Copepod Whole Organism, Normalized *Calanus finmarchicus* cDNA clone CF_WO0_71a12; 5' similar to ref|XP_001601846.1| DIVDICTED: similar to mitotic checkpoint protein and poly(a)+ RNA. Sc Length = 692; score = 44.5 bits (20), expect = 0.039; identities = 26/32 (81%); strand = Plus / Plus

```
Query: 15 agctwawwttgcttgattcacttcwwyaagag 46
          |||| |  ||||||||||||||||   |||||
Sbjct: 1  agctaatattgcttgattcacttctacaagag 32
```

>FG342907|FG342907.1 CF_WO0_69b02_SP6 Copepod Whole Organism, Normalized *Calanus finmarchicus* cDNA clone CF_WO0_69b02; 5', mRNA sequence. Length = 613; score = 44.5 bits (20), expect = 0.039; identities = 26/32 (81%); strand = Plus / Plus

```
Query: 15 agctwawwttgcttgattcacttcwwyaagag 46
          |||| |  |||||||||||||||   |||||
Sbjct: 1  agctaatattgcttgattcacttctacaagag 32
```

>FG342779|FG342779.1 CF_WO0_67f11_SP6 Copepod Whole Organism, Normalized *Calanus finmarchicus* cDNA clone CF_WO0_67f11; 5' similar to gb|AAG00308.1| Rh50 glycoprotein - Papio hamadryas. Score = 94.7 bits (234), expect = 2e- Length = 515; score = 44.5 bits (20), expect = 0.039; identities = 26/32 (81%); strand = Plus / Plus

```
Query: 15 agctwawwttgcttgattcacttcwwyaagag 46
          |||| |  |||||||||||||||   |||||
Sbjct: 1  agctaatattgcttgattcacttctacaagag 32
```

>FG342778|FG342778.1 CF_WO0_67f10_SP6 Copepod Whole Organism, Normalized *Calanus finmarchicus* cDNA clone CF_WO0_67f10; 5' similar to gb|AAG00308.1| Rh50 glycoprotein - Papio hamadryas. Score = 90.9 bits (224), expect = 3e- Length = 527; score = 44.5 bits (20), expect = 0.039; identities = 26/32 (81%); strand = Plus / Plus

```
Query: 15 agctwawwttgcttgattcacttcwwyaagag 46
          |||| |  |||||||||||||||   |||||
Sbjct: 19 agctaatattgcttgattcacttctacaagag 50
```

>FG342777|FG342777.1 CF_WO0_67f09_SP6 Copepod Whole Organism, Normalized *Calanus finmarchicus* cDNA clone CF_WO0_67f09; 5' , mRNA sequence. Length = 537; score = 44.5 bits (20), expect = 0.039; identities = 26/32 (81%); strand = Plus / Plus

```
Query: 15 agctwawwttgcttgattcacttcwwyaagag 46
          |||| |  |||||||||||||||   |||||
Sbjct: 1  agctaatattgcttgattcacttctacaagag 32
```

>FG342776|FG342776.1 CF_WO0_67f08_SP6 Copepod Whole Organism, Normalized *Calanus finmarchicus* cDNA clone CF_WO0_67f08; 5' similar to gb|AAG00308.1| Rh50 glycoprotein - Papio hamadryas. Score = 94.7 bits (234), expect = 2e- Length = 515; score = 44.5 bits (20), expect = 0.039; identities = 26/32 (81%); strand = Plus / Plus

```
Query: 15 agctwawwttgcttgattcacttcwwyaagag 46
          |||| |  |||||||||||||||   |||||
Sbjct: 1  agctaatattgcttgattcacttctacaagag 32
```

>FG342743|FG342743.1 CF_WO0_67c10_SP6 Copepod Whole Organism, Normalized *Calanus finmarchicus* cDNA clone CF_WO0_67c10; 5' similar to ref|XP_001653705.1| glutathione synthetase - Aedes aegypti. Score = 203 bits (517), Length = 607; score = 44.5 bits (20), expect = 0.039; identities = 26/32 (81%); strand = Plus / Plus

```
Query: 15 agctwawwttgcttgattcacttcwwyaagag 46
          |||| |  |||||||||||||||   |||||
Sbjct: 3  agctaatattgcttgattcacttctacaagag 34
```

>FG342593|FG342593.1 CF_WO0_65f06_SP6 Copepod Whole Organism, Normalized *Calanus finmarchicus* cDNA clone CF_WO0_65f06; 5' similar to ref|XP_393567.3| DIVDICTED: similar to CG4300-PA, isoform A isoform 1 [Apis. Score = 110 Length = 707; score = 44.5 bits (20), expect = 0.039; identities = 26/32 (81%); strand = Plus / Plus

```
Query: 15 agctwawwttgcttgattcacttcwwyaagag 46
          |||| |  |||||||||||||||   |||||
Sbjct: 3  agctaatattgcttgattcacttctacaagag 34
```

>FG342377|FG342377.1 CF_WO0_63b05_SP6 Copepod Whole Organism, Normalized *Calanus finmarchicus* cDNA clone CF_WO0_63b05; 5' similar to sp|Q9NB32|TCPD_AEDTR T-complex protein 1 subunit delta (TCP-1-delta) (CCT-delta). Score = Length = 611; score = 44.5 bits (20), expect = 0.039; identities = 26/32 (81%); strand = Plus / Plus

```
Query: 15 agctwawwttgcttgattcacttcwwyaagag 46
          |||| |  |||||||||||||||   |||||
Sbjct: 3  agctaatattgcttgattcacttctacaagag 34
```

>FG342356|FG342356.1 CF_WO0_62h07_SP6 Copepod Whole Organism, Normalized *Calanus finmarchicus* cDNA clone CF_WO0_62h07; 5' similar to ref|XP_559252.3| AGAP010739-PA - Anopheles gambiae str. PEST. Score = 198 bits (504), Exp Length = 659; score = 44.5 bits (20), expect = 0.039; identities = 26/32 (81%); strand = Plus / Plus

```
Query: 15 agctwawwttgcttgattcacttcwwyaagag 46
          |||| |  |||||||||||||||   |||||
Sbjct: 3  agctaatattgcttgattcacttctacaagag 34
```

>FG342343|FG342343.1 CF_WO0_62g06_SP6 Copepod Whole Organism, Normalized *Calanus finmarchicus* cDNA clone CF_WO0_62g06; 5' similar to ref|NP_001037538.1| translation initiation factor 5A - Bombyx mori. Score = 208 bits (529 Length = 607; score = 44.5 bits (20), expect = 0.039; identities = 26/32 (81%); strand = Plus / Plus

```
Query: 15 agctwawwttgcttgattcacttcwwyaagag 46
          |||| |  |||||||||||||||   |||||
Sbjct: 3  agctaatattgcttgattcacttctacaagag 34
```

>FE596722|FE596722.1 CAXG9756.fwd Amphioxus Branchiostoma floridae unpublished cDNA library CAXG, larva whole animal Branchiostoma floridae cDNA clone CAXG9756 5', mRNA sequence. Length = 863; score = 44.5 bits (20), expect = 0.039; identities = 26/32 (81%); strand = Plus / Plus

```
Query: 15 agctwawwttgcttgattcacttcwwyaagag 46
          |||| |  |||||||||||||||   |||||
Sbjct: 1  agctaatattgcttgattcacttctacaagag 32
```

>FE596360|FE596360.1 CAXG9496.fwd Amphioxus Branchiostoma floridae unpublished cDNA library CAXG, larva whole animal Branchiostoma floridae cDNA clone CAXG9496 5', mRNA sequence. Length = 764; score = 44.5 bits (20), expect = 0.039; identities = 26/32 (81%); strand = Plus / Plus

```
Query: 15 agctwawwttgcttgattcacttcwwyaagag 46
```

|||| | ||||||||||||||| |||||
Sbjct: 1 agctaatattgcttgattcacttctacaagag 32

>ES237862|ES237862.1 CF_WO0_60g11_SP6 Copepod Whole Organism, Normalized *Calanus finmarchicus* cDNA clone CF_WO0_60g11; 5' similar to ref|XP_001243976.1| hypothetical protein CIMG_03417 - Coccidioides immitis RS. Score = 72 Length = 699; score = 44.5 bits (20), expect = 0.039; identities = 26/32 (81%); strand = Plus / Plus

```
Query: 15 agctwawwttgcttgattcacttcwwyaagag 46
          ||||  |  |||||||||||||||    |||||
Sbjct: 3  agctaatattgcttgattcacttctacaagag 34
```

>ES237735|ES237735.1 CF_WO0_59e01_SP6 Copepod Whole Organism, Normalized *Calanus finmarchicus* cDNA clone CF_WO0_59e01; 5' similar to ref|XP_393249.2| DIVDICTED: similar to CG32528-PA - Apis mellifera. Score = 221 bits (562 Length = 675; score = 44.5 bits (20), expect = 0.039; identities = 26/32 (81%); strand = Plus / Plus

```
Query: 15 agctwawwttgcttgattcacttcwwyaagag 46
          ||||  |  |||||||||||||||    |||||
Sbjct: 1  agctaatattgcttgattcacttctacaagag 32
```

>ES237693|ES237693.1 CF_WO0_59a05_SP6 Copepod Whole Organism, Normalized *Calanus finmarchicus* cDNA clone CF_WO0_59a05; 5' similar to emb|CAF98199.1| unnamed protein product - Tetraodon nigroviridis. Score = 244 bits (622), Length = 725; score = 44.5 bits (20), expect = 0.039; identities = 26/32 (81%); strand = Plus / Plus

```
Query: 15 agctwawwttgcttgattcacttcwwyaagag 46
          ||||  |  |||||||||||||||    |||||
Sbjct: 3  agctaatattgcttgattcacttctacaagag 34
```

>ES237639|ES237639.1 CF_WO0_58d10_SP6 Copepod Whole Organism, Normalized *Calanus finmarchicus* cDNA clone CF_WO0_58d10; 5' similar to ref|XP_624373.2| DIVDICTED: similar to CG7564-PA - Apis mellifera. Score = 317 bits (812) Length = 672; score = 44.5 bits (20), expect = 0.039; identities = 23/26 (88%); strand = Plus / Plus

```
Query: 13 caagctwawwttgcttgattcacttc 38
          ||||||  |  |||||||||||||||
Sbjct: 3  caagctaatattgcttgattcacttc 28
```

>ES237323|ES237323.1 CF_WO0_55b01_SP6 Copepod Whole Organism, Normalized *Calanus finmarchicus* cDNA clone CF_WO0_55b01; 5' similar to ref|XP_968171.1| DIVDICTED: similar to CG32169-PA - Tribolium castaneum. Score = 270 bits Length = 698; score = 44.5 bits (20), expect = 0.039; identities = 26/32 (81%); strand = Plus / Plus

```
Query: 15 agctwawwttgcttgattcacttcwwyaagag 46
          ||||  |  |||||||||||||||    |||||
Sbjct: 1  agctaatattgcttgattcacttctacaagag 32
```

>EL966259|EL966259.1 CF_WO0_54h01_SP6 Copepod Whole Organism, Normalized *Calanus finmarchicus* cDNA clone CF_WO0_54h01; 5' similar to ref|XP_973173.1| DIVDICTED: similar to CG8114-PA, isoform A - Tribolium castaneum. Score Length = 623; score = 44.5 bits (20), expect = 0.039; identities = 26/32 (81%); strand = Plus / Plus

```
Query: 15 agctwawwttgcttgattcacttcwwyaagag 46
          |||| |  |||||||||||||||   |||||
Sbjct: 3  agctaatattgcttgattcacttctacaagag 34
```

>EL966203|EL966203.1 CF_WO0_54c03_SP6 Copepod Whole Organism, Normalized *Calanus finmarchicus* cDNA clone CF_WO0_54c03; 5' similar to gb|AAP49384.1| glutamate dehydrogenase - Tigriopus californicus. Score = 255 bits (651), Length = 664; score = 44.5 bits (20), expect = 0.039; identities = 26/32 (81%); strand = Plus / Plus

```
Query: 15 agctwawwttgcttgattcacttcwwyaagag 46
          |||| |  |||||||||||||||   |||||
Sbjct: 3  agctaatattgcttgattcacttctacaagag 34
```

>EL966185|EL966185.1 CF_WO0_54a08_SP6 Copepod Whole Organism, Normalized *Calanus finmarchicus* cDNA clone CF_WO0_54a08; 5' similar to pir|JC6567 actin-related protein - Japanese pufferfish. Score = 298 bits (762), expect = Length = 674; score = 44.5 bits (20), expect = 0.039; identities = 26/32 (81%); strand = Plus / Plus

```
Query: 15 agctwawwttgcttgattcacttcwwyaagag 46
          |||| |  |||||||||||||||   |||||
Sbjct: 3  agctaatattgcttgattcacttctacaagag 34
```

>EL966120|EL966120.1 CF_WO0_53c12_SP6 Copepod Whole Organism, Normalized *Calanus finmarchicus* cDNA clone CF_WO0_53c12; 5' similar to ref|NP_001003882.1| seryl-tRNA synthetase - Danio rerio. Score = 239 bits (609), expect = Length = 728; score = 44.5 bits (20), expect = 0.039; identities = 26/32 (81%); strand = Plus / Plus

```
Query: 15 agctwawwttgcttgattcacttcwwyaagag 46
          |||| |  |||||||||||||||   |||||
Sbjct: 3  agctaatattgcttgattcacttctacaagag 34
```

>EL966090|EL966090.1 CF_WO0_53a05_SP6 Copepod Whole Organism, Normalized *Calanus finmarchicus* cDNA clone CF_WO0_53a05; 5' similar to ref|XP_966500.1| DIVDICTED: similar to eukaryotic translation initiation factor 4A2. Scor Length = 717; score = 44.5 bits (20), expect = 0.039; identities = 26/32 (81%); strand = Plus / Plus

```
Query: 15 agctwawwttgcttgattcacttcwwyaagag 46
          |||| |  |||||||||||||||   |||||
Sbjct: 3  agctaatattgcttgattcacttctacaagag 34
```

>EL966086|EL966086.1 CF_WO0_53a01_SP6 Copepod Whole Organism, Normalized *Calanus finmarchicus* cDNA clone CF_WO0_53a01; 5', mRNA sequence. Length = 814; score = 44.5 bits (20), expect = 0.039; identities = 26/32 (81%); strand = Plus / Plus

```
Query: 15 agctwawwttgcttgattcacttcwwyaagag 46
          |||| |  |||||||||||||||   |||||
Sbjct: 36 agctaatattgcttgattcacttctacaagag 67
```

>EL965782|EL965782.1 CF_WO0_49g01_SP6 Copepod Whole Organism, Normalized *Calanus finmarchicus* cDNA clone CF_WO0_49g01; 5' similar to ref|XP_394882.2| DIVDICTED: similar to CG9245-PA, isoform A - Apis mellifera. Score = 271 Length = 675; score = 44.5 bits (20), expect = 0.039; identities = 23/26 (88%); strand = Plus / Plus

```
Query: 12 ccaagctwawwttgcttgattcactt 37
          ||||||| |  ||||||||||||||
Sbjct: 1  ccaagctaatattgcttgattcactt 26
```

>EL965758|EL965758.1 CF_WO0_49e01_SP6 Copepod Whole Organism, Normalized *Calanus finmarchicus* cDNA clone CF_WO0_49e01; 5' similar to ref|XP_312889.2| ENSANGP00000014748 - Anopheles gambiae str. PEST. Score = 310 bits (794) Length = 730; score = 44.5 bits (20), expect = 0.039; identities = 26/32 (81%); strand = Plus / Plus

```
Query: 15 agctwawwttgcttgattcacttcwwyaagag 46
          |||| |  |||||||||||||||   |||||
Sbjct: 1  agctaatattgcttgattcacttctacaagag 32
```

>EL965580|EL965580.1 CF_WO0_47e09_SP6 Copepod Whole Organism, Normalized *Calanus finmarchicus* cDNA clone CF_WO0_47e09; 5', mRNA sequence. Length = 686; score = 44.5 bits (20), expect = 0.039; identities = 26/32 (81%); strand = Plus / Plus

```
Query: 15 agctwawwttgcttgattcacttcwwyaagag 46
          |||| |  |||||||||||||||   |||||
Sbjct: 3  agctaatattgcttgattcacttctacaagag 34
```

>EL965552|EL965552.1 CF_WO0_47c05_SP6 Copepod Whole Organism, Normalized *Calanus finmarchicus* cDNA clone CF_WO0_47c05; 5' similar to gb|AAH82484.1| MGC85351 protein - Xenopus laevis. Score = 149 bits (376), expect = 5e-35, Length = 492; score = 44.5 bits (20), expect = 0.039; identities = 26/32 (81%); strand = Plus / Plus

```
Query: 15 agctwawwttgcttgattcacttcwwyaagag 46
          |||| |  |||||||||||||||   |||||
Sbjct: 5  agctaatattgcttgattcacttctacaagag 36
```

>EL774161|EL774161.1 CF_WO0_44b04_SP6 Copepod Whole Organism, Normalized *Calanus finmarchicus* cDNA clone CF_WO0_44b04; 5' similar to gb|AAS18673.1| gamma-glutamyl transpeptidase precursor - Brugia pahangi. Score = 95.9 bit Length = 722; score = 44.5 bits (20), expect = 0.039; identities = 26/32 (81%); strand = Plus / Plus

```
Query: 15 agctwawwttgcttgattcacttcwwyaagag 46
          |||| |  |||||||||||||||   |||||
Sbjct: 1  agctaatattgcttgattcacttctacaagag 32
```

>EL774047|EL774047.1 CF_WO0_42h09_SP6 Copepod Whole Organism, Normalized *Calanus finmarchicus* cDNA clone CF_WO0_42h09; 5’ similar to ref|XP_968140.1| DIVDICTED: similar to Phosphoglycerate kinase - Tribolium castaneum. Sco Length = 684; score = 44.5 bits (20), expect = 0.039; identities = 26/32 (81%); strand = Plus / Plus

```
Query: 15 agctwawwttgcttgattcacttcwwyaagag 46
          |||| |  ||||||||||||||||   |||||
Sbjct: 3  agctaatattgcttgattcacttctacaagag 34
```

>EL773747|EL773747.1 CF_WO0_39f11_SP6 Copepod Whole Organism, Normalized *Calanus finmarchicus* cDNA clone CF_WO0_39f11; 5’ similar to gb|AAP49384.1| glutamate dehydrogenase - Tigriopus californicus. Score = 259 bits (661), Length = 659; score = 44.5 bits (20), expect = 0.039; identities = 26/32 (81%); strand = Plus / Plus

```
Query: 15 agctwawwttgcttgattcacttcwwyaagag 46
          |||| |  ||||||||||||||||   |||||
Sbjct: 1  agctaatattgcttgattcacttctacaagag 32
```

>EL773741|EL773741.1 CF_WO0_39f05_SP6 Copepod Whole Organism, Normalized *Calanus finmarchicus* cDNA clone CF_WO0_39f05; 5’ similar to ref|XP_973797.1| DIVDICTED: similar to NADH-ubiquinone oxidoreductase 75 kDa. Score = 329 Length = 644; score = 44.5 bits (20), expect = 0.039; identities = 26/32 (81%); strand = Plus / Plus

```
Query: 15 agctwawwttgcttgattcacttcwwyaagag 46
          |||| |  ||||||||||||||||   |||||
Sbjct: 1  agctaatattgcttgattcacttctacaagag 32
```

>EL773669|EL773669.1 CF_WO0_38h05_SP6 Copepod Whole Organism, Normalized *Calanus finmarchicus* cDNA clone CF_WO0_38h05; 5’, mRNA sequence. Length = 655; score = 44.5 bits (20), expect = 0.039; identities = 26/32 (81%); strand = Plus / Plus

```
Query: 15 agctwawwttgcttgattcacttcwwyaagag 46
          |||| |  ||||||||||||||||   |||||
Sbjct: 1  agctaatattgcttgattcacttctacaagag 32
```

>EL773489|EL773489.1 CF_WO0_37a03_SP6 Copepod Whole Organism, Normalized *Calanus finmarchicus* cDNA clone CF_WO0_37a03; 5’ similar to emb|CAE64042.1| Hypothetical protein CBG08640 - Caenorhabditis briggsae. Score = 55.1 bit Length = 688; score = 44.5 bits (20), expect = 0.039; identities = 26/32 (81%); strand = Plus / Plus

```
Query: 15 agctwawwttgcttgattcacttcwwyaagag 46
          |||| |  ||||||||||||||||   |||||
Sbjct: 8  agctaatattgcttgattcacttctacaagag 39
```

>EL773418|EL773418.1 CF_WO0_36c02_SP6 Copepod Whole Organism, Normalized *Calanus finmarchicus* cDNA clone CF_WO0_36c02; 5’ similar to ref|XP_966900.1| DIVDICTED: similar to RAN binding protein 9 - Tribolium castaneum. Score Length = 670; score = 44.5 bits (20), expect = 0.039; identities = 26/32 (81%); strand = Plus / Plus

```
Query: 15 agctwawwttgcttgattcacttcwwyaagag 46
          |||| |  |||||||||||||||   |||||
Sbjct: 1  agctaatattgcttgattcacttctacaagag 32
```

>EL773370|EL773370.1 CF_W00_35g02_SP6 Copepod Whole Organism, Normalized *Calanus finmarchicus* cDNA clone CF_W00_35g02; 5’ similar to gb|AAH71516.1| FK506 binding protein 4 - Danio rerio. Score = 63.5 bits (153), expect = 9 Length = 717; score = 44.5 bits (20), expect = 0.039; identities = 26/32 (81%); strand = Plus / Plus

```
Query: 15 agctwawwttgcttgattcacttcwwyaagag 46
          |||| |  |||||||||||||||   |||||
Sbjct: 1  agctaatattgcttgattcacttctacaagag 32
```

>EL697505|EL697505.1 CF_W00_34f10_SP6 Copepod Whole Organism, Normalized *Calanus finmarchicus* cDNA clone CF_W00_34f10; 5’ similar to dbj|BAA86911.1| homologue of Sarcophaga 26,29kDa proteinase - Periplaneta americana. Scor Length = 681; score = 44.5 bits (20), expect = 0.039; identities = 26/32 (81%); strand = Plus / Plus

```
Query: 15 agctwawwttgcttgattcacttcwwyaagag 46
          |||| |  |||||||||||||||   |||||
Sbjct: 1  agctaatattgcttgattcacttctacaagag 32
```

>EL697417|EL697417.1 CF_W00_33g05_SP6 Copepod Whole Organism, Normalized *Calanus finmarchicus* cDNA clone CF_W00_33g05; 5’ similar to gb|AAI23151.1| MGC154345 protein - Xenopus laevis. Score = 214 bits (544), expect = 4e-54 Length = 677; score = 44.5 bits (20), expect = 0.039; identities = 26/32 (81%); strand = Plus / Plus

```
Query: 15 agctwawwttgcttgattcacttcwwyaagag 46
          |||| |  |||||||||||||||   |||||
Sbjct: 3  agctaatattgcttgattcacttctacaagag 34
```

>EL697379|EL697379.1 CF_W00_33d03_SP6 Copepod Whole Organism, Normalized *Calanus finmarchicus* cDNA clone CF_W00_33d03; 5’ similar to ref|XP_396463.2| DIVDICTED: similar to Putative Achaete Scute Target 1 CG6148-PB,. Score Length = 666; score = 44.5 bits (20), expect = 0.039; identities = 26/32 (81%); strand = Plus / Plus

```
Query: 15 agctwawwttgcttgattcacttcwwyaagag 46
          |||| |  |||||||||||||||   |||||
Sbjct: 3  agctaatattgcttgattcacttctacaagag 34
```

>EL697370|EL697370.1 CF_W00_33c06_SP6 Copepod Whole Organism, Normalized *Calanus finmarchicus* cDNA clone CF_W00_33c06; 5’ similar to ref|XP_396371.3| DIVDICTED: similar to TATA box binding protein-related factor 2. Score = Length = 656; score = 44.5 bits (20), expect = 0.039; identities = 26/32 (81%); strand = Plus / Plus

```
Query: 15 agctwawwttgcttgattcacttcwwyaagag 46
          |||| |  |||||||||||||||   |||||
Sbjct: 3  agctaatattgcttgattcacttctacaagag 34
```

>EL697122|EL697122.1 CF_WO0_30f07_SP6 Copepod Whole Organism, Normalized *Calanus finmarchicus* cDNA clone CF_WO0_30f07; 5' similar to ref|XP_001335567.1| DIVDICTED: hypothetical protein - Danio rerio. Score = 184 bits (466) Length = 669; score = 44.5 bits (20), expect = 0.039; identities = 26/32 (81%); strand = Plus / Plus

```
Query: 15 agctwawwttgcttgattcacttcwwyaagag 46
          |||| |  |||||||||||||||   |||||
Sbjct: 20 agctaatattgcttgattcacttctacaagag 51
```

>EL697119|EL697119.1 CF_WO0_30f04_SP6 Copepod Whole Organism, Normalized *Calanus finmarchicus* cDNA clone CF_WO0_30f04; 5' similar to emb|CAG05526.1| unnamed protein product - Tetraodon nigroviridis. Score = 103 bits (256), Length = 696; score = 44.5 bits (20), expect = 0.039; identities = 26/32 (81%); strand = Plus / Plus

```
Query: 15 agctwawwttgcttgattcacttcwwyaagag 46
          |||| |  |||||||||||||||   |||||
Sbjct: 1  agctaatattgcttgattcacttctacaagag 32
```

>EL697063|EL697063.1 CF_WO0_30a08_SP6 Copepod Whole Organism, Normalized *Calanus finmarchicus* cDNA clone CF_WO0_30a08; 5' similar to ref|NP_989982.1| adhesion regulating molecule 1 - Gallus gallus. Score = 176 bits (447), Length = 702; score = 44.5 bits (20), expect = 0.039; identities = 26/32 (81%); strand = Plus / Plus

```
Query: 15 agctwawwttgcttgattcacttcwwyaagag 46
          |||| |  |||||||||||||||   |||||
Sbjct: 3  agctaatattgcttgattcacttctacaagag 34
```

>EL696917|EL696917.1 CF_WO0_28e02_SP6 Copepod Whole Organism, Normalized *Calanus finmarchicus* cDNA clone CF_WO0_28e02; 5' similar to emb|CAG13129.1| unnamed protein product - Tetraodon nigroviridis. Score = 106 bits (264), Length = 606; score = 44.5 bits (20), expect = 0.039; identities = 26/32 (81%); strand = Plus / Plus

```
Query: 15 agctwawwttgcttgattcacttcwwyaagag 46
          |||| |  |||||||||||||||   |||||
Sbjct: 1  agctaatattgcttgattcacttctacaagag 32
```

>EL696791|EL696791.1 CF_WO0_27b06_SP6 Copepod Whole Organism, Normalized *Calanus finmarchicus* cDNA clone CF_WO0_27b06; 5' similar to gb|ABK59856.1| ME1 - Tigriopus californicus. Score = 290 bits (741), expect = 6e-77-mRNA Length = 732; score = 44.5 bits (20), expect = 0.039; identities = 26/32 (81%); strand = Plus / Plus

```
Query: 15 agctwawwttgcttgattcacttcwwyaagag 46
          |||| |  |||||||||||||||   |||||
Sbjct: 3  agctaatattgcttgattcacttctacaagag 34
```

>EL696789|EL696789.1 CF_WO0_27b04_SP6 Copepod Whole Organism, Normalized *Calanus finmarchicus* cDNA clone CF_WO0_27b04; 5' similar to dbj|BAE31182.1| unnamed protein product - Mus musculus. Score = 325 bits (833), expect = Length = 697; score = 44.5 bits (20), expect = 0.039; identities = 26/32 (81%); strand = Plus / Plus

```
Query: 15 agctwawwttgcttgattcacttcwwyaagag 46
          |||| |  ||||||||||||||||   |||||
Sbjct: 1  agctaatattgcttgattcacttctacaagag 32
```

>EL696720|EL696720.1 CF_WO0_26d06_SP6 Copepod Whole Organism, Normalized *Calanus finmarchicus* cDNA clone CF_WO0_26d06; 5' similar to ref|XP_001352921.1| GA14810-PA - Drosophila pseudoobscura. Score = 85.5 bits (210), Expec Length = 638; score = 44.5 bits (20), expect = 0.039; identities = 26/32 (81%); strand = Plus / Plus

```
Query: 15 agctwawwttgcttgattcacttcwwyaagag 46
          |||| |  ||||||||||||||||   |||||
Sbjct: 3  agctaatattgcttgattcacttctacaagag 34
```

>EL586651|EL586651.1 CF_WO0_24g02_SP6 Copepod Whole Organism, Normalized *Calanus finmarchicus* cDNA clone CF_WO0_24g02; 5' similar to XP_623672.1 CG5525-PA isoform 1 [Apis mellifera]. Score=252.68, Expect=8.20E-66, mRNA seq Length = 667; score = 44.5 bits (20), expect = 0.039; identities = 26/32 (81%); strand = Plus / Plus

```
Query: 15 agctwawwttgcttgattcacttcwwyaagag 46
          |||| |  ||||||||||||||||   |||||
Sbjct: 3  agctaatattgcttgattcacttctacaagag 34
```

>EL586635|EL586635.1 CF_WO0_24e07_SP6 Copepod Whole Organism, Normalized *Calanus finmarchicus* cDNA clone CF_WO0_24e07; 5' , mRNA sequence. Length = 708; score = 44.5 bits (20), expect = 0.039; identities = 26/32 (81%); strand = Plus / Plus

```
Query: 15 agctwawwttgcttgattcacttcwwyaagag 46
          |||| |  ||||||||||||||||   |||||
Sbjct: 1  agctaatattgcttgattcacttctacaagag 32
```

>EL586624|EL586624.1 CF_WO0_24d05_SP6 Copepod Whole Organism, Normalized *Calanus finmarchicus* cDNA clone CF_WO0_24d05; 5' similar to XP_394383.1 CG6876-PA isoform 1 [Apis mellifera]. Score=172.17, Expect=1.60E-41, mRNA seq Length = 705; score = 44.5 bits (20), expect = 0.039; identities = 26/32 (81%); strand = Plus / Plus

```
Query: 15 agctwawwttgcttgattcacttcwwyaagag 46
          |||| |  ||||||||||||||||   |||||
Sbjct: 3  agctaatattgcttgattcacttctacaagag 34
```

>EL586181|EL586181.1 CF_WO0_19f04_SP6 Copepod Whole Organism, Normalized *Calanus finmarchicus* cDNA clone CF_WO0_19f04; 5' similar to NP_873586.1 putative oxalate/formate antiporter [Haemophilus ducreyi 35000HP]. Score=124. Length = 686; score = 44.5 bits (20), expect = 0.039; identities = 26/32 (81%); strand = Plus / Plus

```
Query: 15 agctwawwttgcttgattcacttcwwyaagag 46
          |||| |  ||||||||||||||||   |||||
Sbjct: 1  agctaatattgcttgattcacttctacaagag 32
```

>EL586026|EL586026.1 CF_W00_17h06_SP6 Copepod Whole Organism, Normalized *Calanus finmarchicus* cDNA clone CF_W00_17h06; 5' similar to XP_974502.1 CG3051-PA, isoform A [Tribolium castaneum]. Score=403.29, Expect=4.50E-111, m Length = 728; score = 44.5 bits (20), expect = 0.039; identities = 26/32 (81%); strand = Plus / Plus

```
Query: 15 agctwawwttgcttgattcacttcwwyaagag 46
          |||| |  |||||||||||||||   |||||
Sbjct: 3  agctaatattgcttgattcacttctacaagag 34
```

>EL585799|EL585799.1 CF_W00_15d11_SP6 Copepod Whole Organism, Normalized *Calanus finmarchicus* cDNA clone CF_W00_15d11; 5' similar to EAL31502.1 GA15916-PA [Drosophila pseudoobscura]. Score=140.58, Expect=4.50E-32, mRNA seq Length = 655; score = 44.5 bits (20), expect = 0.039; identities = 26/32 (81%); strand = Plus / Plus

```
Query: 15 agctwawwttgcttgattcacttcwwyaagag 46
          |||| |  |||||||||||||||   |||||
Sbjct: 1  agctaatattgcttgattcacttctacaagag 32
```

>EL585725|EL585725.1 CF_W00_14f05_SP6 Copepod Whole Organism, Normalized *Calanus finmarchicus* cDNA clone CF_W00_14f05; 5' similar to CAG09753.1 unnamed protein product [Tetraodon nigroviridis]. Score=145.98, Expect=1.40E-3 Length = 749; score = 44.5 bits (20), expect = 0.039; identities = 26/32 (81%); strand = Plus / Plus

```
Query: 15 agctwawwttgcttgattcacttcwwyaagag 46
          |||| |  |||||||||||||||   |||||
Sbjct: 1  agctaatattgcttgattcacttctacaagag 32
```

>EL585644|EL585644.1 CF_W00_13g07_SP6 Copepod Whole Organism, Normalized *Calanus finmarchicus* cDNA clone CF_W00_13g07; 5' similar to NP_001073548.1 hypothetical protein LOC790934 [Danio rerio]. Score=70.86, Expect=3.60E-11 Length = 605; score = 44.5 bits (20), expect = 0.039; identities = 26/32 (81%); strand = Plus / Plus

```
Query: 15 agctwawwttgcttgattcacttcwwyaagag 46
          |||| |  |||||||||||||||   |||||
Sbjct: 3  agctaatattgcttgattcacttctacaagag 34
```

>EL585575|EL585575.1 CF_W00_13a02_SP6 Copepod Whole Organism, Normalized *Calanus finmarchicus* cDNA clone CF_W00_13a02; 5' similar to NP_001027725.1 Ci-META1 [Ciona intestinalis]. Score=77.41, Expect=5.30E-13, mRNA sequence Length = 701; score = 44.5 bits (20), expect = 0.039; identities = 26/32 (81%); strand = Plus / Plus

```
Query: 15 agctwawwttgcttgattcacttcwwyaagag 46
          |||| |  |||||||||||||||   |||||
Sbjct: 1  agctaatattgcttgattcacttctacaagag 32
```

>EL585528|EL585528.1 CF_W00_12d11_SP6 Copepod Whole Organism, Normalized *Calanus finmarchicus* cDNA clone CF_W00_12d11; 5' similar to XP_311871.2 ENSANGP00000023984 [Anopheles gambiae str. PEST]. Score=293.89, Expect=3.10E- Length = 650; score = 44.5 bits (20), expect = 0.039; identities = 26/32 (81%); strand = Plus / Plus

```
Query: 15 agctwawwttgcttgattcacttcwwyaagag 46
          |||| |  |||||||||||||||   |||||
Sbjct: 3  agctaatattgcttgattcacttctacaagag 34
```

>EL585518|EL585518.1 CF_WO0_12c10_SP6 Copepod Whole Organism, Normalized *Calanus finmarchicus* cDNA clone CF_WO0_12c10; 5' similar to EAL30496.1 GA18460-PA [Drosophila pseudoobscura]. Score=198.36, Expect=2.10E-49, mRNA seq Length = 713; score = 44.5 bits (20), expect = 0.039; identities = 26/32 (81%); strand = Plus / Plus

```
Query: 15 agctwawwttgcttgattcacttcwwyaagag 46
          |||| |  |||||||||||||||   |||||
Sbjct: 3  agctaatattgcttgattcacttctacaagag 34
```

>EL585451|EL585451.1 CF_WO0_11e09_SP6 Copepod Whole Organism, Normalized *Calanus finmarchicus* cDNA clone CF_WO0_11e09; 5' , mRNA sequence. Length = 639; score = 44.5 bits (20), expect = 0.039; identities = 26/32 (81%); strand = Plus / Plus

```
Query: 15 agctwawwttgcttgattcacttcwwyaagag 46
          |||| |  |||||||||||||||   |||||
Sbjct: 3  agctaatattgcttgattcacttctacaagag 34
```

>EH667156|EH667156.1 CF_WO0_10f05_SP6 Copepod Whole Organism, Normalized *Calanus finmarchicus* cDNA clone CF_WO0_10f05; 5' similar to ref|NP_609898.1| CG10341-PA - Drosophila melanogaster. Score = 56.2 bits (134), expect = Length = 678; score = 44.5 bits (20), expect = 0.039; identities = 26/32 (81%); strand = Plus / Plus

```
Query: 15 agctwawwttgcttgattcacttcwwyaagag 46
          |||| |  |||||||||||||||   |||||
Sbjct: 1  agctaatattgcttgattcacttctacaagag 32
```

>EH667086|EH667086.1 CF_WO0_09h05_SP6 Copepod Whole Organism, Normalized *Calanus finmarchicus* cDNA clone CF_WO0_09h05; 5' similar to gb|EAL32488.1| GA13249-PA - Drosophila pseudoobscura. Score = 292 bits (748), expect = 8e Length = 686; score = 44.5 bits (20), expect = 0.039; identities = 26/32 (81%); strand = Plus / Plus

```
Query: 15 agctwawwttgcttgattcacttcwwyaagag 46
          |||| |  |||||||||||||||   |||||
Sbjct: 27 agctaatattgcttgattcacttctacaagag 58
```

>EH666815|EH666815.1 CF_WO0_06h09_SP6 Copepod Whole Organism, Normalized *Calanus finmarchicus* cDNA clone CF_WO0_06h09; 5' similar to ref|XP_417682.1| DIVDICTED: similar to mitochondrial solute carrier protein [Gallus. Scor Length = 660; score = 44.5 bits (20), expect = 0.039; identities = 26/32 (81%); strand = Plus / Plus

```
Query: 15 agctwawwttgcttgattcacttcwwyaagag 46
          |||| |  |||||||||||||||   |||||
Sbjct: 3  agctaatattgcttgattcacttctacaagag 34
```

>EH666743|EH666743.1 CF_W00_06b06_SP6 Copepod Whole Organism, Normalized *Calanus finmarchicus* cDNA clone CF_W00_06b06; 5' similar to ref|XP_972566.1| DIVDICTED: similar to CG4685-PA, isoform A - Tribolium castaneum. Score Length = 395; score = 44.5 bits (20), expect = 0.039; identities = 26/32 (81%); strand = Plus / Plus

```
Query: 15 agctwawwttgcttgattcacttcwwyaagag 46
          |||| |  ||||||||||||||||   |||||
Sbjct: 3  agctaatattgcttgattcacttctacaagag 34
```

>EH666649|EH666649.1 CF_W00_05b06_SP6 Copepod Whole Organism, Normalized *Calanus finmarchicus* cDNA clone CF_W00_05b06; 5' , mRNA sequence. Length = 696; score = 44.5 bits (20), expect = 0.039; identities = 26/32 (81%); strand = Plus / Plus

```
Query: 15 agctwawwttgcttgattcacttcwwyaagag 46
          |||| |  ||||||||||||||||   |||||
Sbjct: 3  agctaatattgcttgattcacttctacaagag 34
```

>EH666638|EH666638.1 CF_W00_05a07_SP6 Copepod Whole Organism, Normalized *Calanus finmarchicus* cDNA clone CF_W00_05a07; 5' , mRNA sequence. Length = 608; score = 44.5 bits (20), expect = 0.039; identities = 26/32 (81%); strand = Plus / Plus

```
Query: 15 agctwawwttgcttgattcacttcwwyaagag 46
          |||| |  ||||||||||||||||   |||||
Sbjct: 1  agctaatattgcttgattcacttctacaagag 32
```

>EH666606|EH666606.1 CF_W00_04f07_SP6 Copepod Whole Organism, Normalized *Calanus finmarchicus* cDNA clone CF_W00_04f07; 5' similar to ref|XP_788451.1| DIVDICTED: hypothetical protein - Strongylocentrotus purpuratus. Score = Length = 657; score = 44.5 bits (20), expect = 0.039; identities = 26/32 (81%); strand = Plus / Plus

```
Query: 15 agctwawwttgcttgattcacttcwwyaagag 46
          |||| |  ||||||||||||||||   |||||
Sbjct: 1  agctaatattgcttgattcacttctacaagag 32
```

>EH666543|EH666543.1 CF_W00_03h10_SP6 Copepod Whole Organism, Normalized *Calanus finmarchicus* cDNA clone CF_W00_03h10; 5' similar to gb|EAT38719.1| NADH-ubiquinone oxidoreductase 39 kda subunit - Aedes aegypti. Score = 161 Length = 573; score = 44.5 bits (20), expect = 0.039; identities = 26/32 (81%); strand = Plus / Plus

```
Query: 15 agctwawwttgcttgattcacttcwwyaagag 46
          |||| |  ||||||||||||||||   |||||
Sbjct: 9  agctaatattgcttgattcacttctacaagag 40
```

>EH666537|EH666537.1 CF_W00_03h04_SP6 Copepod Whole Organism, Normalized *Calanus finmarchicus* cDNA clone CF_W00_03h04; 5' , mRNA sequence. Length = 546; score = 44.5 bits (20), expect = 0.039; identities = 26/32 (81%); strand = Plus / Plus

```
Query: 15 agctwawwttgcttgattcacttcwwyaagag 46
          |||| |  ||||||||||||||||   |||||
```

```
Sbjct: 1  agctaatattgcttgattcacttctacaagag 32
```

>EH666394|EH666394.1 CF_WO0_02c12_SP6 Copepod Whole Organism, Normalized *Calanus finmarchicus* cDNA clone CF_WO0_02c12; 5' similar to gb|AAY87330.1| predicted histidinol dehydrogenase - uncultured bacterium BAC17H8. Score = Length = 772; score = 44.5 bits (20), expect = 0.039; identities = 26/32 (81%); strand = Plus / Plus

```
Query: 15 agctwawwttgcttgattcacttcwwyaagag 46
          |||| |  |||||||||||||||   |||||
Sbjct: 3  agctaatattgcttgattcacttctacaagag 34
```

>EH666356|EH666356.1 CF_WO0_01h10_SP6 Copepod Whole Organism, Normalized *Calanus finmarchicus* cDNA clone CF_WO0_01h10; 5' , mRNA sequence. Length = 598; score = 44.5 bits (20), expect = 0.039; identities = 26/32 (81%); strand = Plus / Plus

```
Query: 15 agctwawwttgcttgattcacttcwwyaagag 46
          |||| |  |||||||||||||||   |||||
Sbjct: 10 agctaatattgcttgattcacttctacaagag 41
```

>EH666354|EH666354.1 CF_WO0_01h08_SP6 Copepod Whole Organism, Normalized *Calanus finmarchicus* cDNA clone CF_WO0_01h08; 5' similar to ref|XP_973194.1| DIVDICTED: similar to CG17299-PA, isoform A - Tribolium castaneum. Score Length = 598; score = 44.5 bits (20), expect = 0.039; identities = 26/32 (81%); strand = Plus / Plus

```
Query: 15 agctwawwttgcttgattcacttcwwyaagag 46
          |||| |  |||||||||||||||   |||||
Sbjct: 3  agctaatattgcttgattcacttctacaagag 34
```

>EB740555|EB740555.1 Sa_mx1_34h03_SP6 Dogfish Shark Multiple Tissues, Normalized Squalus acanthias cDNA clone Sa_mx1_34h03 5' similar to ref|XP_395978.2| DIVDICTED: similar to out at first, partial - Apis mellifera. Score Length = 672; score = 44.5 bits (20), expect = 0.039; identities = 26/32 (81%); strand = Plus / Plus

```
Query: 15 agctwawwttgcttgattcacttcwwyaagag 46
          |||| |  |||||||||||||||   |||||
Sbjct: 3  agctaatattgcttgattcacttctacaagag 34
```

>DY656934|DY656934.1 Cm_mx1_86f06_SP6 Green Shore Crab Multiple Tissue, Normalized Carcinus maenas cDNA clone Cm_mx1_86f06 5' similar to Similar to ref|NP_524469.2| Proteasome 26S subunit subunit 4 ATPase CG5289-PA [Droso Length = 735; score = 44.5 bits (20), expect = 0.039; identities = 26/32 (81%); strand = Plus / Plus

```
Query: 15 agctwawwttgcttgattcacttcwwyaagag 46
          |||| |  |||||||||||||||   |||||
Sbjct: 3  agctaatattgcttgattcacttctacaagag 34
```

>DY656747|DY656747.1 Cm_mx1_84f04_SP6 Green Shore Crab Multiple Tissue, Normalized Carcinus maenas cDNA clone Cm_mx1_84f04 5' similar to Similar to ref|NP_524469.2| Proteasome 26S

subunit subunit 4 ATPase CG5289-PA [Droso Length = 695; score = 44.5 bits (20), expect = 0.039; identities = 26/32 (81%); strand = Plus / Plus

```
Query: 15 agctwawwttgcttgattcacttcwwyaagag 46
          |||| |  ||||||||||||||||   |||||
Sbjct: 3  agctaatattgcttgattcacttctacaagag 34
```

>DW250273|DW250273.1 Cm_mx1_32b03_SP6 Green Shore Crab Multiple Tissue, Normalized Carcinus maenas cDNA clone Cm_mx1_32b03 5', mRNA sequence. Length = 293; score = 44.5 bits (20), expect = 0.039; identities = 26/32 (81%); strand = Plus / Plus

```
Query: 15 agctwawwttgcttgattcacttcwwyaagag 46
          |||| |  ||||||||||||||||   |||||
Sbjct: 1  agctaatattgcttgattcacttctacaagag 32
```

>DW250218|DW250218.1 Cm_mx1_31e08_SP6 Green Shore Crab Multiple Tissue, Normalized Carcinus maenas cDNA clone Cm_mx1_31e08 5', mRNA sequence. Length = 302; score = 44.5 bits (20), expect = 0.039; identities = 26/32 (81%); strand = Plus / Plus

```
Query: 15 agctwawwttgcttgattcacttcwwyaagag 46
          |||| |  ||||||||||||||||   |||||
Sbjct: 10 agctaatattgcttgattcacttctacaagag 41
```

>JZ533424|JZ533424.1 SSH_RH03_H08_ab1 Cal SSH_SKE_RHO 2d *Calanus helgolandicus* cDNA clone SSH_RH03_H08_ab1 similar to 60s ribosomal protein l17, mRNA sequence. Length = 284; score = 42.3 bits (19), expect = 0.18; identities = 25/31 (80%); strand = Plus / Minus

```
Query: 16  gctwawwttgcttgattcacttcwwyaagag 46
           ||| |  ||||||||||||||||   |||||
Sbjct: 276 gctaatattgcttgattcacttctacaagag 246
```

>JZ533401|JZ533401.1 SSH_RH02_D10_T7_ab1 Cal SSH_SKE_RHO 2d *Calanus helgolandicus* cDNA clone SSH_RH02_D10_T7_ab1 similar to actin-depolymerizing factor 4, mRNA sequence. Length = 316; score = 42.3 bits (19), expect = 0.18; identities = 25/31 (80%); strand = Plus / Plus

```
Query: 16 gctwawwttgcttgattcacttcwwyaagag 46
          ||| |  ||||||||||||||||   |||||
Sbjct: 1  gctaatattgcttgattcacttctacaagag 31
```

>JZ532999|JZ532999.1 SSH_SKE4_G12_T7_ab1 Cal SSH_SKE_RHO 2d *Calanus helgolandicus* cDNA clone SSH_SKE4_G12_T7_ab1 similar to voltage-dependent anion-selective channel-mRNA sequence. Length = 526; score = 42.3 bits (19), expect = 0.18; identities = 25/31 (80%); strand = Plus / Plus

```
Query: 16 gctwawwttgcttgattcacttcwwyaagag 46
          ||| |  ||||||||||||||||   |||||
Sbjct: 1  gctaatattgcttgattcacttctacaagag 31
```

>BW849092|BW849092.1 Branchiostoma floridae cDNA, neurula clone:bfne035p19, 5’ end. Length = 389; score = 42.3 bits (19), expect = 0.18; identities = 25/31 (80%); strand = Plus / Plus

```
Query: 16 gctwawwttgcttgattcacttcwwyaagag 46
          ||| |  |||||||||||||||   |||||
Sbjct: 1  gctaatattgcttgattcacttctacaagag 31
```

>BW838699|BW838699.1 Branchiostoma floridae cDNA, neurula clone:bfne001b15, 5’ end. Length = 221; score = 42.3 bits (19), expect = 0.18; identities = 25/31 (80%); strand = Plus / Plus

```
Query: 16 gctwawwttgcttgattcacttcwwyaagag 46
          ||| |  |||||||||||||||   |||||
Sbjct: 1  gctaatattgcttgattcacttctacaagag 31
```

>BW770801|BW770801.1 Branchiostoma floridae cDNA, gastrula clone:bfga016d06, 5’ end. Length = 680; score = 42.3 bits (19), expect = 0.18; identities = 25/31 (80%); strand = Plus / Plus

```
Query: 16 gctwawwttgcttgattcacttcwwyaagag 46
          ||| |  |||||||||||||||   |||||
Sbjct: 1  gctaatattgcttgattcacttctacaagag 31
```

>GR411206|GR411206.1 CF_WO1_58h04_SP6 Copepod Whole Organism, Normalized *Calanus finmarchicus* cDNA clone CF_WO1_58h04; 5’ similar to gb|ACO10233.1| Aspartate aminotransferase, mitochondrial precursor [Caligus. Score = 357 Length = 803; score = 42.3 bits (19), expect = 0.18; identities = 25/31 (80%); strand = Plus / Plus

```
Query: 16 gctwawwttgcttgattcacttcwwyaagag 46
          ||| |  |||||||||||||||   |||||
Sbjct: 1  gctaatattgcttgattcacttctacaagag 31
```

>GR411044|GR411044.1 CF_WO1_56f06_SP6 Copepod Whole Organism, Normalized *Calanus finmarchicus* cDNA clone CF_WO1_56f06; 5’, mRNA sequence. Length = 550; score = 42.3 bits (19), expect = 0.18; identities = 25/31 (80%); strand = Plus / Plus

```
Query: 16 gctwawwttgcttgattcacttcwwyaagag 46
          ||| |  |||||||||||||||   |||||
Sbjct: 1  gctaatattgcttgattcacttctacaagag 31
```

>GR410904|GR410904.1 CF_WO1_54e07_SP6 Copepod Whole Organism, Normalized *Calanus finmarchicus* cDNA clone CF_WO1_54e07; 5’ similar to gb|ABC84495.1| heat shock protein 40 - Locusta migratoria. Score = 214 bits (546), expect Length = 784; score = 42.3 bits (19), expect = 0.18; identities = 25/31 (80%); strand = Plus / Plus

```
Query: 16 gctwawwttgcttgattcacttcwwyaagag 46
          ||| |  |||||||||||||||   |||||
Sbjct: 1  gctaatattgcttgattcacttctacaagag 31
```

>GR410822|GR410822.1 CF_WO1_53c12_SP6 Copepod Whole Organism, Normalized *Calanus finmarchicus* cDNA clone CF_WO1_53c12; 5’ similar to ref|XP_966390.1| DIVDICTED: similar to GA17475-PA -

Tribolium castaneum. Score = 268 bits Length = 804; score = 42.3 bits (19), expect = 0.18; identities = 25/31 (80%); strand = Plus / Plus

```
Query: 16 gctwawwttgcttgattcacttcwwyaagag 46
          ||| |  ||||||||||||||||   |||||
Sbjct: 1  gctaatattgcttgattcacttctacaagag 31
```

>GR410810|GR410810.1 CF_W01_53b09_SP6 Copepod Whole Organism, Normalized *Calanus finmarchicus* cDNA clone CF_W01_53b09; 5' similar to ref|XP_972171.1| DIVDICTED: similar to mRNA capping enzyme - Tribolium castaneum. Score = Length = 839; score = 42.3 bits (19), expect = 0.18; identities = 25/31 (80%); strand = Plus / Plus

```
Query: 16 gctwawwttgcttgattcacttcwwyaagag 46
          ||| |  ||||||||||||||||   |||||
Sbjct: 1  gctaatattgcttgattcacttctacaagag 31
```

>GR410807|GR410807.1 CF_W01_53b06_SP6 Copepod Whole Organism, Normalized *Calanus finmarchicus* cDNA clone CF_W01_53b06; 5' similar to ref|XP_001605864.1| DIVDICTED: similar to suppressor of actin (sac) [Nasonia. Score = 222 Length = 778; score = 42.3 bits (19), expect = 0.18; identities = 25/31 (80%); strand = Plus / Plus

```
Query: 16 gctwawwttgcttgattcacttcwwyaagag 46
          ||| |  ||||||||||||||||   |||||
Sbjct: 1  gctaatattgcttgattcacttctacaagag 31
```

>FQ001105|FQ001105.1 Pleurobrachia pileus 5-PRIME EST from clone SQ0AAA96YL18 (SQ0AAA96YL18RM1). Length = 826; score = 42.3 bits (19), expect = 0.18; identities = 25/31 (80%); strand = Plus / Plus

```
Query: 16 gctwawwttgcttgattcacttcwwyaagag 46
          ||| |  ||||||||||||||||   |||||
Sbjct: 1  gctaatattgcttgattcacttctacaagag 31
```

>FK868220|FK868220.1 CF_W01_18H03_SP6 Copepod Whole Organism, Normalized *Calanus finmarchicus* cDNA clone CF_W01_18H03; 5' similar to ref|XP_001942661.1| DIVDICTED: similar to AGAP009403-PA - Acyrthosiphon pisum. Score = 66 Length = 582; score = 42.3 bits (19), expect = 0.18; identities = 25/31 (80%); strand = Plus / Plus

```
Query: 16 gctwawwttgcttgattcacttcwwyaagag 46
          ||| |  ||||||||||||||||   |||||
Sbjct: 1  gctaatattgcttgattcacttctacaagag 31
```

>FK868142|FK868142.1 CF_W01_18A05_SP6 Copepod Whole Organism, Normalized *Calanus finmarchicus* cDNA clone CF_W01_18A05; 5', mRNA sequence. Length = 494; score = 42.3 bits (19), expect = 0.18; identities = 25/31 (80%); strand = Plus / Plus

```
Query: 16 gctwawwttgcttgattcacttcwwyaagag 46
          ||| |  ||||||||||||||||   |||||
Sbjct: 3  gctaatattgcttgattcacttctacaagag 33
```

>FK868092|FK868092.1 CF_W01_16F12_SP6 Copepod Whole Organism, Normalized *Calanus finmarchicus* cDNA clone CF_W01_16F12; 5' similar to emb|CAF98951.1| unnamed protein product - Tetraodon nigroviridis. Score = 157 bits (397), Length = 671; score = 42.3 bits (19), expect = 0.18; identities = 25/31 (80%); strand = Plus / Plus

```
Query: 16 gctwawwttgcttgattcacttcwwyaagag 46
          ||| |  |||||||||||||||   |||||
Sbjct: 3  gctaatattgcttgattcacttctacaagag 33
```

>FK868057|FK868057.1 CF_W01_16C12_SP6 Copepod Whole Organism, Normalized *Calanus finmarchicus* cDNA clone CF_W01_16C12; 5' similar to ref|XP_972170.1| DIVDICTED: similar to spire CG10076-PB - Tribolium castaneum. Score = 11 Length = 666; score = 42.3 bits (19), expect = 0.18; identities = 25/31 (80%); strand = Plus / Plus

```
Query: 16 gctwawwttgcttgattcacttcwwyaagag 46
          ||| |  |||||||||||||||   |||||
Sbjct: 3  gctaatattgcttgattcacttctacaagag 33
```

>FK868021|FK868021.1 CF_W01_15G09_SP6 Copepod Whole Organism, Normalized *Calanus finmarchicus* cDNA clone CF_W01_15G09; 5' similar to ref|XP_001851260.1| ATPase n2b - Culex pipiens quinquefasciatus. Score = 72.4 bits (176), Length = 580; score = 42.3 bits (19), expect = 0.18; identities = 25/31 (80%); strand = Plus / Plus

```
Query: 16 gctwawwttgcttgattcacttcwwyaagag 46
          ||| |  |||||||||||||||   |||||
Sbjct: 3  gctaatattgcttgattcacttctacaagag 33
```

>FK868001|FK868001.1 CF_W01_15E10_SP6 Copepod Whole Organism, Normalized *Calanus finmarchicus* cDNA clone CF_W01_15E10; 5' similar to ref|NP_001097474.1| CG12004 CG12004-PC, isoform C - Drosophila melanogaster. Score = 136 Length = 630; score = 42.3 bits (19), expect = 0.18; identities = 25/31 (80%); strand = Plus / Plus

```
Query: 16 gctwawwttgcttgattcacttcwwyaagag 46
          ||| |  |||||||||||||||   |||||
Sbjct: 3  gctaatattgcttgattcacttctacaagag 33
```

>FK867998|FK867998.1 CF_W01_15E05_SP6 Copepod Whole Organism, Normalized *Calanus finmarchicus* cDNA clone CF_W01_15E05; 5' similar to ref|NP_001080077.1| hydroxyacyl-Coenzyme A dehydrogenase/3-ketoacyl-Coenzyme A. Score = 2 Length = 679; score = 42.3 bits (19), expect = 0.18; identities = 25/31 (80%); strand = Plus / Plus

```
Query: 16 gctwawwttgcttgattcacttcwwyaagag 46
          ||| |  |||||||||||||||   |||||
Sbjct: 3  gctaatattgcttgattcacttctacaagag 33
```

>FK867951|FK867951.1 CF_W01_14H10_SP6 Copepod Whole Organism, Normalized *Calanus finmarchicus* cDNA clone CF_W01_14H10; 5' similar to ref|XP_966498.1| DIVDICTED: similar to VAMP-

associated protein, putative [Tribolium. Scor Length = 598; score = 42.3 bits (19), expect = 0.18; identities = 25/31 (80%); strand = Plus / Plus

```
Query: 16 gctwawwttgcttgattcacttcwwyaagag 46
          ||| |  ||||||||||||||||   |||||
Sbjct: 3  gctaatattgcttgattcacttctacaagag 33
```

>FK867888|FK867888.1 CF_W01_13H09_SP6 Copepod Whole Organism, Normalized *Calanus finmarchicus* cDNA clone CF_W01_13H09; 5' , mRNA sequence. Length = 634; score = 42.3 bits (19), expect = 0.18; identities = 25/31 (80%); strand = Plus / Plus

```
Query: 16 gctwawwttgcttgattcacttcwwyaagag 46
          ||| |  ||||||||||||||||   |||||
Sbjct: 7  gctaatattgcttgattcacttctacaagag 37
```

>FK867852|FK867852.1 CF_W01_13E02_SP6 Copepod Whole Organism, Normalized *Calanus finmarchicus* cDNA clone CF_W01_13E02; 5' similar to ref|XP_966420.2| DIVDICTED: similar to membrane protein tms1d isoform 1 [Tribolium. Score Length = 628; score = 42.3 bits (19), expect = 0.18; identities = 25/31 (80%); strand = Plus / Plus

```
Query: 16 gctwawwttgcttgattcacttcwwyaagag 46
          ||| |  ||||||||||||||||   |||||
Sbjct: 3  gctaatattgcttgattcacttctacaagag 33
```

>FK867777|FK867777.1 CF_W01_12F01_SP6 Copepod Whole Organism, Normalized *Calanus finmarchicus* cDNA clone CF_W01_12F01; 5' similar to ref|XP_394615.2| DIVDICTED: similar to caseinolytic peptidase X - Apis mellifera. Score = Length = 656; score = 42.3 bits (19), expect = 0.18; identities = 25/31 (80%); strand = Plus / Plus

```
Query: 16 gctwawwttgcttgattcacttcwwyaagag 46
          ||| |  ||||||||||||||||   |||||
Sbjct: 3  gctaatattgcttgattcacttctacaagag 33
```

>FK867600|FK867600.1 CF_W01_10E04_SP6 Copepod Whole Organism, Normalized *Calanus finmarchicus* cDNA clone CF_W01_10E04; 5' , mRNA sequence. Length = 652; score = 42.3 bits (19), expect = 0.18; identities = 25/31 (80%); strand = Plus / Plus

```
Query: 16 gctwawwttgcttgattcacttcwwyaagag 46
          ||| |  ||||||||||||||||   |||||
Sbjct: 3  gctaatattgcttgattcacttctacaagag 33
```

>FK867523|FK867523.1 CF_W01_09F06_SP6 Copepod Whole Organism, Normalized *Calanus finmarchicus* cDNA clone CF_W01_09F06; 5' similar to ref|NP_001016454.2| hypothetical protein LOC549208 - Xenopus tropicalis. Score = 128 bits Length = 557; score = 42.3 bits (19), expect = 0.18; identities = 25/31 (80%); strand = Plus / Plus

```
Query: 16 gctwawwttgcttgattcacttcwwyaagag 46
          ||| |  ||||||||||||||||   |||||
Sbjct: 1  gctaatattgcttgattcacttctacaagag 31
```

>FK671146|FK671146.1 CF_W01_06H01_SP6 Copepod Whole Organism, Normalized *Calanus finmarchicus* cDNA clone CF_W01_06H01; 5’ similar to ref|XP_001354915.1| GA18281-PA - Drosophila pseudoobscura. Score = 127 bits (319), expect Length = 691; score = 42.3 bits (19), expect = 0.18; identities = 25/31 (80%); strand = Plus / Plus

```
Query: 16 gctwawwttgcttgattcacttcwwyaagag 46
          ||| |  ||||||||||||||||   |||||
Sbjct: 3  gctaatattgcttgattcacttctacaagag 33
```

>FK671115|FK671115.1 CF_W01_06E05_SP6 Copepod Whole Organism, Normalized *Calanus finmarchicus* cDNA clone CF_W01_06E05; 5’ similar to ref|XP_700365.3| DIVDICTED: similar to OMA1 homolog, zinc metallopeptidase [Danio. Score Length = 676; score = 42.3 bits (19), expect = 0.18; identities = 25/31 (80%); strand = Plus / Plus

```
Query: 16 gctwawwttgcttgattcacttcwwyaagag 46
          ||| |  ||||||||||||||||   |||||
Sbjct: 3  gctaatattgcttgattcacttctacaagag 33
```

>FK671090|FK671090.1 CF_W01_06C03_SP6 Copepod Whole Organism, Normalized *Calanus finmarchicus* cDNA clone CF_W01_06C03; 5’ similar to gb|EDV31170.1| GF15226 - Drosophila ananassae. Score = 108 bits (270), expect = 3e-22, mR Length = 639; score = 42.3 bits (19), expect = 0.18; identities = 25/31 (80%); strand = Plus / Plus

```
Query: 16 gctwawwttgcttgattcacttcwwyaagag 46
          ||| |  ||||||||||||||||   |||||
Sbjct: 3  gctaatattgcttgattcacttctacaagag 33
```

>FK670973|FK670973.1 CF_W01_04H07_SP6 Copepod Whole Organism, Normalized *Calanus finmarchicus* cDNA clone CF_W01_04H07; 5’ similar to ref|XP_314005.4| AGAP005124-PC - Anopheles gambiae str. PEST. Score = 160 bits (406), Exp Length = 560; score = 42.3 bits (19), expect = 0.18; identities = 25/31 (80%); strand = Plus / Plus

```
Query: 16 gctwawwttgcttgattcacttcwwyaagag 46
          ||| |  ||||||||||||||||   |||||
Sbjct: 3  gctaatattgcttgattcacttctacaagag 33
```

>FK670922|FK670922.1 CF_W01_04D03_SP6 Copepod Whole Organism, Normalized *Calanus finmarchicus* cDNA clone CF_W01_04D03; 5’, mRNA sequence. Length = 686; score = 42.3 bits (19), expect = 0.18; identities = 25/31 (80%); strand = Plus / Plus

```
Query: 16 gctwawwttgcttgattcacttcwwyaagag 46
          ||| |  ||||||||||||||||   |||||
Sbjct: 3  gctaatattgcttgattcacttctacaagag 33
```

>FK670904|FK670904.1 CF_W01_04B08_SP6 Copepod Whole Organism, Normalized *Calanus finmarchicus* cDNA clone CF_W01_04B08; 5’ similar to ref|NP_001007892.1| MGC89988 protein - Xenopus tropicalis. Score = 87.8 bits (216), Expec Length = 654; score = 42.3 bits (19), expect = 0.18; identities = 25/31 (80%); strand = Plus / Plus

```
Query: 16 gctwawwttgcttgattcacttcwwyaagag 46
          ||| |  |||||||||||||||   |||||
Sbjct: 3  gctaatattgcttgattcacttctacaagag 33
```

>FK670888|FK670888.1 CF_W01_04A03_SP6 Copepod Whole Organism, Normalized *Calanus finmarchicus* cDNA clone CF_W01_04A03; 5', mRNA sequence. Length = 703; score = 42.3 bits (19), expect = 0.18; identities = 25/31 (80%); strand = Plus / Plus

```
Query: 16 gctwawwttgcttgattcacttcwwyaagag 46
          ||| |  |||||||||||||||   |||||
Sbjct: 3  gctaatattgcttgattcacttctacaagag 33
```

>FK670729|FK670729.1 CF_W01_02B03_SP6 Copepod Whole Organism, Normalized *Calanus finmarchicus* cDNA clone CF_W01_02B03; 5', mRNA sequence. Length = 688; score = 42.3 bits (19), expect = 0.18; identities = 25/31 (80%); strand = Plus / Plus

```
Query: 16 gctwawwttgcttgattcacttcwwyaagag 46
          ||| |  |||||||||||||||   |||||
Sbjct: 1  gctaatattgcttgattcacttctacaagag 31
```

>FK670516|FK670516.1 CF_W00_99E06_SP6 Copepod Whole Organism, Normalized *Calanus finmarchicus* cDNA clone CF_W00_99E06; 5' similar to gb|EDV59455.1| GG23397 - Drosophila erecta. Score = 236 bits (602), expect = 1e-60, mRNA Length = 714; score = 42.3 bits (19), expect = 0.18; identities = 25/31 (80%); strand = Plus / Plus

```
Query: 16 gctwawwttgcttgattcacttcwwyaagag 46
          ||| |  |||||||||||||||   |||||
Sbjct: 1  gctaatattgcttgattcacttctacaagag 31
```

>FK670436|FK670436.1 CF_W00_98D04_SP6 Copepod Whole Organism, Normalized *Calanus finmarchicus* cDNA clone CF_W00_98D04; 5', mRNA sequence. Length = 703; score = 42.3 bits (19), expect = 0.18; identities = 25/31 (80%); strand = Plus / Plus

```
Query: 16 gctwawwttgcttgattcacttcwwyaagag 46
          ||| |  |||||||||||||||   |||||
Sbjct: 3  gctaatattgcttgattcacttctacaagag 33
```

>FK670434|FK670434.1 CF_W00_98D02_SP6 Copepod Whole Organism, Normalized *Calanus finmarchicus* cDNA clone CF_W00_98D02; 5', mRNA sequence. Length = 707; score = 42.3 bits (19), expect = 0.18; identities = 25/31 (80%); strand = Plus / Plus

```
Query: 16 gctwawwttgcttgattcacttcwwyaagag 46
          ||| |  |||||||||||||||   |||||
Sbjct: 1  gctaatattgcttgattcacttctacaagag 31
```

>FK670416|FK670416.1 CF_W00_98B08_SP6 Copepod Whole Organism, Normalized *Calanus finmarchicus* cDNA clone CF_W00_98B08; 5' similar to gb|AAD46404.1|AF096248_1 ethylene-responsive RNA

helicase - Lycopersicon esculentum. Scor Length = 690; score = 42.3 bits (19), expect = 0.18; identities = 25/31 (80%); strand = Plus / Plus

```
Query: 16 gctwawwttgcttgattcacttcwwyaagag 46
          ||| |  |||||||||||||||   |||||
Sbjct: 1  gctaatattgcttgattcacttctacaagag 31
```

>FK041665|FK041665.1 CF_WO0_96H06_SP6 Copepod Whole Organism, Normalized *Calanus finmarchicus* cDNA clone CF_WO0_96H06; 5' , mRNA sequence. Length = 664; score = 42.3 bits (19), expect = 0.18; identities = 25/31 (80%); strand = Plus / Plus

```
Query: 16 gctwawwttgcttgattcacttcwwyaagag 46
          ||| |  |||||||||||||||   |||||
Sbjct: 3  gctaatattgcttgattcacttctacaagag 33
```

>FK041410|FK041410.1 CF_WO0_94A08_SP6 Copepod Whole Organism, Normalized *Calanus finmarchicus* cDNA clone CF_WO0_94A08; 5' similar to gb|AAK50057.2|AF364404_1 Rh-like protein - Carcinus maenas. Score = 134 bits (337), Expec Length = 653; score = 42.3 bits (19), expect = 0.18; identities = 25/31 (80%); strand = Plus / Plus

```
Query: 16 gctwawwttgcttgattcacttcwwyaagag 46
          ||| |  |||||||||||||||   |||||
Sbjct: 5  gctaatattgcttgattcacttctacaagag 35
```

>FK041358|FK041358.1 CF_WO0_93D11_SP6 Copepod Whole Organism, Normalized *Calanus finmarchicus* cDNA clone CF_WO0_93D11; 5' similar to ref|NP_001079914.1| hypothetical protein LOC379604 - Xenopus laevis. Score = 203 bits (51 Length = 742; score = 42.3 bits (19), expect = 0.18; identities = 25/31 (80%); strand = Plus / Plus

```
Query: 16 gctwawwttgcttgattcacttcwwyaagag 46
          ||| |  |||||||||||||||   |||||
Sbjct: 1  gctaatattgcttgattcacttctacaagag 31
```

>FK041174|FK041174.1 CF_WO0_91D10_SP6 Copepod Whole Organism, Normalized *Calanus finmarchicus* cDNA clone CF_WO0_91D10; 5' similar to gb|EDV59774.1| GG10792 - Drosophila erecta. Score = 280 bits (715), expect = 6e-74, mRNA Length = 629; score = 42.3 bits (19), expect = 0.18; identities = 25/31 (80%); strand = Plus / Plus

```
Query: 16 gctwawwttgcttgattcacttcwwyaagag 46
          ||| |  |||||||||||||||   |||||
Sbjct: 1  gctaatattgcttgattcacttctacaagag 31
```

>FK041164|FK041164.1 CF_WO0_91C08_SP6 Copepod Whole Organism, Normalized *Calanus finmarchicus* cDNA clone CF_WO0_91C08; 5' similar to ref|XP_308308.4| AGAP007565-PB - Anopheles gambiae str. PEST. Score = 119 bits (297), Exp Length = 672; score = 42.3 bits (19), expect = 0.18; identities = 25/31 (80%); strand = Plus / Plus

```
Query: 16 gctwawwttgcttgattcacttcwwyaagag 46
          ||| |  |||||||||||||||   |||||
```

Sbjct: 3 gctaatattgcttgattcacttctacaagag 33

>FK041148|FK041148.1 CF_WO0_91B01_SP6 Copepod Whole Organism, Normalized *Calanus finmarchicus* cDNA clone CF_WO0_91B01; 5' similar to gb|EDV46376.1| GG19053 - Drosophila erecta. Score = 87.0 bits (214), expect = 1e-15, mRNA Length = 697; score = 42.3 bits (19), expect = 0.18; identities = 25/31 (80%); strand = Plus / Plus

```
Query: 16 gctwawwttgcttgattcacttcwwyaagag 46
          ||| |  |||||||||||||||   |||||
Sbjct: 3  gctaatattgcttgattcacttctacaagag 33
```

>FG985901|FG985901.1 CF_WO0_86G02_SP6 Copepod Whole Organism, Normalized *Calanus finmarchicus* cDNA clone CF_WO0_86G02; 5' similar to ref|XP_001659451.1| hypothetical protein AaeL_AAEL008718 - Aedes aegypti. Score = 180 bit Length = 667; score = 42.3 bits (19), expect = 0.18; identities = 25/31 (80%); strand = Plus / Plus

```
Query: 16 gctwawwttgcttgattcacttcwwyaagag 46
          ||| |  |||||||||||||||   |||||
Sbjct: 1  gctaatattgcttgattcacttctacaagag 31
```

>FG985809|FG985809.1 CF_WO0_85G02_SP6 Copepod Whole Organism, Normalized *Calanus finmarchicus* cDNA clone CF_WO0_85G02; 5' similar to ref|XP_971904.1| DIVDICTED: similar to LOC100037005 protein - Tribolium castaneum. Score Length = 593; score = 42.3 bits (19), expect = 0.18; identities = 25/31 (80%); strand = Plus / Plus

```
Query: 16 gctwawwttgcttgattcacttcwwyaagag 46
          ||| |  |||||||||||||||   |||||
Sbjct: 1  gctaatattgcttgattcacttctacaagag 31
```

>FG985728|FG985728.1 CF_WO0_84E12_SP6 Copepod Whole Organism, Normalized *Calanus finmarchicus* cDNA clone CF_WO0_84E12; 5' , mRNA sequence. Length = 525; score = 42.3 bits (19), expect = 0.18; identities = 25/31 (80%); strand = Plus / Plus

```
Query: 16 gctwawwttgcttgattcacttcwwyaagag 46
          ||| |  |||||||||||||||   |||||
Sbjct: 5  gctaatattgcttgattcacttctacaagag 35
```

>FG985678|FG985678.1 CF_WO0_84A04_SP6 Copepod Whole Organism, Normalized *Calanus finmarchicus* cDNA clone CF_WO0_84A04; 5' similar to gb|ABU41071.1| receptor for activated protein kinase C-like protein. Score = 393 bits (10 Length = 683; score = 42.3 bits (19), expect = 0.18; identities = 25/31 (80%); strand = Plus / Plus

```
Query: 16 gctwawwttgcttgattcacttcwwyaagag 46
          ||| |  |||||||||||||||   |||||
Sbjct: 3  gctaatattgcttgattcacttctacaagag 33
```

>FG985541|FG985541.1 CF_WO0_82B11_SP6 Copepod Whole Organism, Normalized *Calanus finmarchicus* cDNA clone CF_WO0_82B11; 5' , mRNA sequence. Length = 739; score = 42.3 bits (19), expect = 0.18; identities = 25/31 (80%); strand = Plus / Plus

```
Query: 16 gctwawwttgcttgattcacttcwwyaagag 46
          ||| |  |||||||||||||||   |||||
Sbjct: 1  gctaatattgcttgattcacttctacaagag 31
```

>FG985530|FG985530.1 CF_WO0_82A08_SP6 Copepod Whole Organism, Normalized *Calanus finmarchicus* cDNA clone CF_WO0_82A08; 5' similar to ref|NP_001011001.1| actin-related protein 10 homolog - Xenopus tropicalis. Score = 192 bi Length = 561; score = 42.3 bits (19), expect = 0.18; identities = 25/31 (80%); strand = Plus / Plus

```
Query: 16 gctwawwttgcttgattcacttcwwyaagag 46
          ||| |  |||||||||||||||   |||||
Sbjct: 1  gctaatattgcttgattcacttctacaagag 31
```

>FG985290|FG985290.1 CF_WO0_79B03_SP6 Copepod Whole Organism, Normalized *Calanus finmarchicus* cDNA clone CF_WO0_79B03; 5' similar to gb|EDV47396.1| GG19604 - Drosophila erecta. Score = 270 bits (691), expect = 4e-71, mRNA Length = 622; score = 42.3 bits (19), expect = 0.18; identities = 25/31 (80%); strand = Plus / Plus

```
Query: 16 gctwawwttgcttgattcacttcwwyaagag 46
          ||| |  |||||||||||||||   |||||
Sbjct: 1  gctaatattgcttgattcacttctacaagag 31
```

>FG633049|FG633049.1 CF_WO0_76h02_SP6 Copepod Whole Organism, Normalized *Calanus finmarchicus* cDNA clone CF_WO0_76h02; 5' similar to ref|XP_001845591.1| THO complex subunit 1 - Culex pipiens quinquefasciatus. Score = 143 b Length = 623; score = 42.3 bits (19), expect = 0.18; identities = 22/25 (88%); strand = Plus / Plus

```
Query: 13 caagctwawwttgcttgattcactt 37
          |||||| |  ||||||||||||||
Sbjct: 1  caagctaatattgcttgattcactt 25
```

>FG632907|FG632907.1 CF_WO0_75c08_SP6 Copepod Whole Organism, Normalized *Calanus finmarchicus* cDNA clone CF_WO0_75c08; 5' similar to ref|XP_001607895.1| DIVDICTED: similar to sugar transporter - Nasonia vitripennis. Score Length = 649; score = 42.3 bits (19), expect = 0.18; identities = 25/31 (80%); strand = Plus / Plus

```
Query: 16 gctwawwttgcttgattcacttcwwyaagag 46
          ||| |  |||||||||||||||   |||||
Sbjct: 1  gctaatattgcttgattcacttctacaagag 31
```

>FG632866|FG632866.1 CF_WO0_74g11_SP6 Copepod Whole Organism, Normalized *Calanus finmarchicus* cDNA clone CF_WO0_74g11; 5' similar to ref|XP_001638857.1| predicted protein - Nematostella vectensis. Score = 100 bits (248), E. Length = 663; score = 42.3 bits (19), expect = 0.18; identities = 25/31 (80%); strand = Plus / Plus

```
Query: 16 gctwawwttgcttgattcacttcwwyaagag 46
          ||| |  |||||||||||||||   |||||
Sbjct: 1  gctaatattgcttgattcacttctacaagag 31
```

>FG632850|FG632850.1 CF_WO0_74f07_SP6 Copepod Whole Organism, Normalized *Calanus finmarchicus* cDNA clone CF_WO0_74f07; 5’ similar to ref|XP_001641680.1| predicted protein - Nematostella vectensis. Score = 134 bits (337), E. Length = 695; score = 42.3 bits (19), expect = 0.18; identities = 25/31 (80%); strand = Plus / Plus

```
Query: 16 gctwawwttgcttgattcacttcwwyaagag 46
          ||| |  ||||||||||||||||   |||||
Sbjct: 1  gctaatattgcttgattcacttctacaagag 31
```

>FG632844|FG632844.1 CF_WO0_74f01_SP6 Copepod Whole Organism, Normalized *Calanus finmarchicus* cDNA clone CF_WO0_74f01; 5’ similar to gb|AAI54268.1| Zgc:110840 protein - Danio rerio. Score = 92.4 bits (228), expect = 2e-17, Length = 701; score = 42.3 bits (19), expect = 0.18; identities = 25/31 (80%); strand = Plus / Plus

```
Query: 16 gctwawwttgcttgattcacttcwwyaagag 46
          ||| |  ||||||||||||||||   |||||
Sbjct: 3  gctaatattgcttgattcacttctacaagag 33
```

>FG632842|FG632842.1 CF_WO0_74e10_SP6 Copepod Whole Organism, Normalized *Calanus finmarchicus* cDNA clone CF_WO0_74e10; 5’, mRNA sequence. Length = 686; score = 42.3 bits (19), expect = 0.18; identities = 25/31 (80%); strand = Plus / Plus

```
Query: 16 gctwawwttgcttgattcacttcwwyaagag 46
          ||| |  ||||||||||||||||   |||||
Sbjct: 1  gctaatattgcttgattcacttctacaagag 31
```

>FG632756|FG632756.1 CF_WO0_73f05_SP6 Copepod Whole Organism, Normalized *Calanus finmarchicus* cDNA clone CF_WO0_73f05; 5’, mRNA sequence. Length = 596; score = 42.3 bits (19), expect = 0.18; identities = 25/31 (80%); strand = Plus / Plus

```
Query: 16 gctwawwttgcttgattcacttcwwyaagag 46
          ||| |  ||||||||||||||||   |||||
Sbjct: 1  gctaatattgcttgattcacttctacaagag 31
```

>FG632743|FG632743.1 CF_WO0_73e04_SP6 Copepod Whole Organism, Normalized *Calanus finmarchicus* cDNA clone CF_WO0_73e04; 5’ similar to ref|XP_001358566.1| GA15194-PA - Drosophila pseudoobscura. Score = 128 bits (322), expect Length = 600; score = 42.3 bits (19), expect = 0.18; identities = 22/25 (88%); strand = Plus / Plus

```
Query: 13 caagctwawwttgcttgattcactt 37
          |||||| |  |||||||||||||||
Sbjct: 1  caagctaatattgcttgattcactt 25
```

>FG632719|FG632719.1 CF_WO0_73c02_SP6 Copepod Whole Organism, Normalized *Calanus finmarchicus* cDNA clone CF_WO0_73c02; 5’ similar to ref|XP_320057.3| AGAP009265-PA - Anopheles gambiae str. PEST. Score = 135 bits (340), Exp Length = 653; score = 42.3 bits (19), expect = 0.18; identities = 25/31 (80%); strand = Plus / Plus

Query: 16 gctwawwttgcttgattcacttcwwyaagag 46
         ||| | ||||||||||||||| |||||
Sbjct: 3 gctaatattgcttgattcacttctacaagag 33

>FG343028|FG343028.1 CF_W00_70d06_SP6 Copepod Whole Organism, Normalized *Calanus finmarchicus* cDNA clone CF_W00_70d06; 5' similar to ref|NP_956440.1| hypothetical protein LOC393115 - Danio rerio. Score = 187 bits (476), Ex Length = 670; score = 42.3 bits (19), expect = 0.18; identities = 25/31 (80%); strand = Plus / Plus

Query: 16 gctwawwttgcttgattcacttcwwyaagag 46
         ||| | ||||||||||||||| |||||
Sbjct: 3 gctaatattgcttgattcacttctacaagag 33

>FG343016|FG343016.1 CF_W00_70c05_SP6 Copepod Whole Organism, Normalized *Calanus finmarchicus* cDNA clone CF_W00_70c05; 5', mRNA sequence. Length = 687; score = 42.3 bits (19), expect = 0.18; identities = 25/31 (80%); strand = Plus / Plus

Query: 16 gctwawwttgcttgattcacttcwwyaagag 46
         ||| | ||||||||||||||| |||||
Sbjct: 3 gctaatattgcttgattcacttctacaagag 33

>FG342993|FG342993.1 CF_W00_70a06_SP6 Copepod Whole Organism, Normalized *Calanus finmarchicus* cDNA clone CF_W00_70a06; 5' similar to ref|NP_001083656.1| propionyl-CoA carboxylase - Xenopus laevis. Score = 300 bits (767), E. Length = 695; score = 42.3 bits (19), expect = 0.18; identities = 25/31 (80%); strand = Plus / Plus

Query: 16 gctwawwttgcttgattcacttcwwyaagag 46
         ||| | ||||||||||||||| |||||
Sbjct: 1 gctaatattgcttgattcacttctacaagag 31

>FG342913|FG342913.1 CF_W00_69b08_SP6 Copepod Whole Organism, Normalized *Calanus finmarchicus* cDNA clone CF_W00_69b08; 5', mRNA sequence. Length = 630; score = 42.3 bits (19), expect = 0.18; identities = 25/31 (80%); strand = Plus / Plus

Query: 16 gctwawwttgcttgattcacttcwwyaagag 46
         ||| | ||||||||||||||| |||||
Sbjct: 3 gctaatattgcttgattcacttctacaagag 33

>FG342851|FG342851.1 CF_W00_68e02_SP6 Copepod Whole Organism, Normalized *Calanus finmarchicus* cDNA clone CF_W00_68e02; 5', mRNA sequence. Length = 597; score = 42.3 bits (19), expect = 0.18; identities = 25/31 (80%); strand = Plus / Plus

Query: 16 gctwawwttgcttgattcacttcwwyaagag 46
         ||| | ||||||||||||||| |||||
Sbjct: 3 gctaatattgcttgattcacttctacaagag 33

>FG342825|FG342825.1 CF_W00_68b11_SP6 Copepod Whole Organism, Normalized *Calanus finmarchicus* cDNA clone CF_W00_68b11; 5' similar to ref|NP_001073456.1| transmembrane protein 41B - Danio

rerio. Score = 165 bits (417), Expe Length = 643; score = 42.3 bits (19), expect = 0.18; identities = 25/31 (80%); strand = Plus / Plus

```
Query: 16 gctwawwttgcttgattcacttcwwyaagag 46
          ||| |  |||||||||||||||   |||||
Sbjct: 1  gctaatattgcttgattcacttctacaagag 31
```

>FG342813|FG342813.1 CF_WO0_68a11_SP6 Copepod Whole Organism, Normalized *Calanus finmarchicus* cDNA clone CF_WO0_68a11; 5' similar to ref|XP_974826.1| DIVDICTED: similar to Myotubularin-related protein 2 [Tribolium. Score = Length = 619; score = 42.3 bits (19), expect = 0.18; identities = 25/31 (80%); strand = Plus / Plus

```
Query: 16 gctwawwttgcttgattcacttcwwyaagag 46
          ||| |  |||||||||||||||   |||||
Sbjct: 3  gctaatattgcttgattcacttctacaagag 33
```

>FG342726|FG342726.1 CF_WO0_67b03_SP6 Copepod Whole Organism, Normalized *Calanus finmarchicus* cDNA clone CF_WO0_67b03; 5' similar to ref|XP_783899.1| DIVDICTED: similar to Ribophorin II - Strongylocentrotus purpuratus. Sco Length = 573; score = 42.3 bits (19), expect = 0.18; identities = 25/31 (80%); strand = Plus / Plus

```
Query: 16 gctwawwttgcttgattcacttcwwyaagag 46
          ||| |  |||||||||||||||   |||||
Sbjct: 3  gctaatattgcttgattcacttctacaagag 33
```

>FG342632|FG342632.1 CF_WO0_66a09_SP6 Copepod Whole Organism, Normalized *Calanus finmarchicus* cDNA clone CF_WO0_66a09; 5' similar to ref|XP_001605423.1| DIVDICTED: similar to CG11866-PA - Nasonia vitripennis. Score = 145 b Length = 572; score = 42.3 bits (19), expect = 0.18; identities = 25/31 (80%); strand = Plus / Plus

```
Query: 16 gctwawwttgcttgattcacttcwwyaagag 46
          ||| |  |||||||||||||||   |||||
Sbjct: 3  gctaatattgcttgattcacttctacaagag 33
```

>FG342496|FG342496.1 CF_WO0_64e07_SP6 Copepod Whole Organism, Normalized *Calanus finmarchicus* cDNA clone CF_WO0_64e07; 5' similar to ref|XP_969225.1| DIVDICTED: similar to CG4666-PA - Tribolium castaneum. Score = 120 bits Length = 599; score = 42.3 bits (19), expect = 0.18; identities = 25/29 (86%); strand = Plus / Plus

```
Query: 10 gaccaagctwawwttgcttgattcacttc 38
          ||||||||| |  ||||||||||| ||||
Sbjct: 19 gaccaagctaatattgcttgattcccttc 47
```

>FG342440|FG342440.1 CF_WO0_63g12_SP6 Copepod Whole Organism, Normalized *Calanus finmarchicus* cDNA clone CF_WO0_63g12; 5' similar to ref|XP_970842.1| DIVDICTED: similar to CG7935-PA - Tribolium castaneum. Score = 304 bits Length = 716; score = 42.3 bits (19), expect = 0.18; identities = 25/31 (80%); strand = Plus / Plus

```
Query: 16 gctwawwttgcttgattcacttcwwyaagag 46
```

```
         ||| |  |||||||||||||||   |||||
Sbjct: 3  gctaatattgcttgattcacttctacaagag 33
```

>FG342428|FG342428.1 CF_WO0_63f12_SP6 Copepod Whole Organism, Normalized *Calanus finmarchicus* cDNA clone CF_WO0_63f12; 5' similar to ref|XP_970842.1| DIVDICTED: similar to CG7935-PA - Tribolium castaneum. Score = 306 bits Length = 716; score = 42.3 bits (19), expect = 0.18; identities = 25/31 (80%); strand = Plus / Plus

```
Query: 16 gctwawwttgcttgattcacttcwwyaagag 46
          ||| |  |||||||||||||||   |||||
Sbjct: 3  gctaatattgcttgattcacttctacaagag 33
```

>FG342402|FG342402.1 CF_WO0_63d08_SP6 Copepod Whole Organism, Normalized *Calanus finmarchicus* cDNA clone CF_WO0_63d08; 5' similar to ref|XP_001600419.1| DIVDICTED: similar to RE11240p - Nasonia vitripennis. Score = 90.9 bi Length = 637; score = 42.3 bits (19), expect = 0.18; identities = 25/31 (80%); strand = Plus / Plus

```
Query: 16 gctwawwttgcttgattcacttcwwyaagag 46
          ||| |  |||||||||||||||   |||||
Sbjct: 3  gctaatattgcttgattcacttctacaagag 33
```

>FG342401|FG342401.1 CF_WO0_63d07_SP6 Copepod Whole Organism, Normalized *Calanus finmarchicus* cDNA clone CF_WO0_63d07; 5' similar to ref|XP_001600419.1| DIVDICTED: similar to RE11240p - Nasonia vitripennis. Score = 86.3 bi Length = 633; score = 42.3 bits (19), expect = 0.18; identities = 25/31 (80%); strand = Plus / Plus

```
Query: 16 gctwawwttgcttgattcacttcwwyaagag 46
          ||| |  |||||||||||||||   |||||
Sbjct: 3  gctaatattgcttgattcacttctacaagag 33
```

>FG342345|FG342345.1 CF_WO0_62g08_SP6 Copepod Whole Organism, Normalized *Calanus finmarchicus* cDNA clone CF_WO0_62g08; 5' similar to emb|CAF96389.1| unnamed protein product - Tetraodon nigroviridis. Score = 77.8 bits (190) Length = 601; score = 42.3 bits (19), expect = 0.18; identities = 25/31 (80%); strand = Plus / Plus

```
Query: 16 gctwawwttgcttgattcacttcwwyaagag 46
          ||| |  |||||||||||||||   |||||
Sbjct: 3  gctaatattgcttgattcacttctacaagag 33
```

>FG342272|FG342272.1 CF_WO0_62a05_SP6 Copepod Whole Organism, Normalized *Calanus finmarchicus* cDNA clone CF_WO0_62a05; 5', mRNA sequence. Length = 718; score = 42.3 bits (19), expect = 0.18; identities = 25/31 (80%); strand = Plus / Plus

```
Query: 16 gctwawwttgcttgattcacttcwwyaagag 46
          ||| |  |||||||||||||||   |||||
Sbjct: 3  gctaatattgcttgattcacttctacaagag 33
```

>FG342227|FG342227.1 CF_WO0_61e07_SP6 Copepod Whole Organism, Normalized *Calanus finmarchicus* cDNA clone CF_WO0_61e07; 5' similar to ref|XP_623894.1| DIVDICTED: similar to CG7942-PA,

isoform A - Apis mellifera. Score = 253 Length = 624; score = 42.3 bits (19), expect = 0.18; identities = 25/31 (80%); strand = Plus / Plus

```
Query: 16 gctwawwttgcttgattcacttcwwyaagag 46
          ||| |  ||||||||||||||||   |||||
Sbjct: 3  gctaatattgcttgattcacttctacaagag 33
```

>FG342225|FG342225.1 CF_WO0_61e05_SP6 Copepod Whole Organism, Normalized *Calanus finmarchicus* cDNA clone CF_WO0_61e05; 5' similar to ref|XP_001603184.1| DIVDICTED: similar to CG4567-PA - Nasonia vitripennis. Score = 234 bi Length = 653; score = 42.3 bits (19), expect = 0.18; identities = 25/31 (80%); strand = Plus / Plus

```
Query: 16 gctwawwttgcttgattcacttcwwyaagag 46
          ||| |  ||||||||||||||||   |||||
Sbjct: 3  gctaatattgcttgattcacttctacaagag 33
```

>ES237773|ES237773.1 CF_WO0_59h03_SP6 Copepod Whole Organism, Normalized *Calanus finmarchicus* cDNA clone CF_WO0_59h03; 5' similar to gb|AAH62854.1| Rplp0 protein - Danio rerio. Score = 311 bits (797), expect = 2e-83, mRNA Length = 705; score = 42.3 bits (19), expect = 0.18; identities = 25/31 (80%); strand = Plus / Plus

```
Query: 16 gctwawwttgcttgattcacttcwwyaagag 46
          ||| |  ||||||||||||||||   |||||
Sbjct: 3  gctaatattgcttgattcacttctacaagag 33
```

>ES237691|ES237691.1 CF_WO0_59a03_SP6 Copepod Whole Organism, Normalized *Calanus finmarchicus* cDNA clone CF_WO0_59a03; 5' similar to ref|XP_312273.3| ENSANGP00000021082 - Anopheles gambiae str. PEST. Score = 219 bits (557) Length = 664; score = 42.3 bits (19), expect = 0.18; identities = 25/31 (80%); strand = Plus / Plus

```
Query: 16 gctwawwttgcttgattcacttcwwyaagag 46
          ||| |  ||||||||||||||||   |||||
Sbjct: 1  gctaatattgcttgattcacttctacaagag 31
```

>ES237665|ES237665.1 CF_WO0_58g01_SP6 Copepod Whole Organism, Normalized *Calanus finmarchicus* cDNA clone CF_WO0_58g01; 5' similar to sp|Q612F5|PCCA_CAEBR Propionyl-CoA carboxylase alpha chain, mitochondrial precursor. Scor Length = 683; score = 42.3 bits (19), expect = 0.18; identities = 25/31 (80%); strand = Plus / Plus

```
Query: 16 gctwawwttgcttgattcacttcwwyaagag 46
          ||| |  ||||||||||||||||   |||||
Sbjct: 3  gctaatattgcttgattcacttctacaagag 33
```

>ES237513|ES237513.1 CF_WO0_57b01_SP6 Copepod Whole Organism, Normalized *Calanus finmarchicus* cDNA clone CF_WO0_57b01; 5' , mRNA sequence. Length = 696; score = 42.3 bits (19), expect = 0.18; identities = 25/31 (80%); strand = Plus / Plus

```
Query: 16 gctwawwttgcttgattcacttcwwyaagag 46
          ||| |  ||||||||||||||||   |||||
```

Sbjct: 1 gctaatattgcttgattcacttctacaagag 31

>ES237455|ES237455.1 CF_WO0_56e01_SP6 Copepod Whole Organism, Normalized *Calanus finmarchicus* cDNA clone CF_WO0_56e01; 5’ similar to ref|XP_974487.1| DIVDICTED: similar to CG3194-PA - Tribolium castaneum. Score = 148 bits Length = 755; score = 42.3 bits (19), expect = 0.18; identities = 25/31 (80%); strand = Plus / Plus

```
Query: 16 gctwawwttgcttgattcacttcwwyaagag 46
          ||| |  |||||||||||||||   |||||
Sbjct: 3  gctaatattgcttgattcacttctacaagag 33
```

>ES237392|ES237392.1 CF_WO0_55g10_SP6 Copepod Whole Organism, Normalized *Calanus finmarchicus* cDNA clone CF_WO0_55g10; 5’ similar to ref|XP_972672.1| DIVDICTED: similar to CG1424-PA - Tribolium castaneum. Score = 148 bits Length = 675; score = 42.3 bits (19), expect = 0.18; identities = 25/31 (80%); strand = Plus / Plus

```
Query: 16 gctwawwttgcttgattcacttcwwyaagag 46
          ||| |  |||||||||||||||   |||||
Sbjct: 3  gctaatattgcttgattcacttctacaagag 33
```

>ES237370|ES237370.1 CF_WO0_55e12_SP6 Copepod Whole Organism, Normalized *Calanus finmarchicus* cDNA clone CF_WO0_55e12; 5’ similar to sp|O02654|ENO_LOLPE Enolase (2-phosphoglycerate dehydratase) (2-phospho-D-glycerate. Scor Length = 665; score = 42.3 bits (19), expect = 0.18; identities = 25/31 (80%); strand = Plus / Plus

```
Query: 16 gctwawwttgcttgattcacttcwwyaagag 46
          ||| |  |||||||||||||||   |||||
Sbjct: 3  gctaatattgcttgattcacttctacaagag 33
```

>ES237352|ES237352.1 CF_WO0_55d06_SP6 Copepod Whole Organism, Normalized *Calanus finmarchicus* cDNA clone CF_WO0_55d06; 5’, mRNA sequence. Length = 706; score = 42.3 bits (19), expect = 0.18; identities = 25/31 (80%); strand = Plus / Plus

```
Query: 16 gctwawwttgcttgattcacttcwwyaagag 46
          ||| |  |||||||||||||||   |||||
Sbjct: 31 gctaatattgcttgattcacttctacaagag 61
```

>ES237338|ES237338.1 CF_WO0_55c04_SP6 Copepod Whole Organism, Normalized *Calanus finmarchicus* cDNA clone CF_WO0_55c04; 5’ similar to gb|AAG40239.1|AF302777_1 carboxylesterase precursor - Nilaparvata lugens. Score = 111 bit Length = 710; score = 42.3 bits (19), expect = 0.18; identities = 25/31 (80%); strand = Plus / Plus

```
Query: 16 gctwawwttgcttgattcacttcwwyaagag 46
          ||| |  |||||||||||||||   |||||
Sbjct: 3  gctaatattgcttgattcacttctacaagag 33
```

>ES237329|ES237329.1 CF_WO0_55b07_SP6 Copepod Whole Organism, Normalized *Calanus finmarchicus* cDNA clone CF_WO0_55b07; 5’ similar to ref|XP_966950.1| DIVDICTED: similar to CG9761-PA

isoform 1 - Tribolium castaneum. Score = Length = 778; score = 42.3 bits (19), expect = 0.18; identities = 25/31 (80%); strand = Plus / Plus

```
Query: 16 gctwawwttgcttgattcacttcwwyaagag 46
          ||| |  |||||||||||||||   |||||
Sbjct: 36 gctaatattgcttgattcacttctacaagag 66
```

>EL966232|EL966232.1 CF_WO0_54e09_SP6 Copepod Whole Organism, Normalized *Calanus finmarchicus* cDNA clone CF_WO0_54e09; 5' similar to ref|NP_572375.1| innexin 2 CG4590-PA, isoform A - Drosophila melanogaster. Score = 64.3 b Length = 719; score = 42.3 bits (19), expect = 0.18; identities = 25/31 (80%); strand = Plus / Plus

```
Query: 16 gctwawwttgcttgattcacttcwwyaagag 46
          ||| |  |||||||||||||||   |||||
Sbjct: 3  gctaatattgcttgattcacttctacaagag 33
```

>EL966174|EL966174.1 CF_WO0_53h08_SP6 Copepod Whole Organism, Normalized *Calanus finmarchicus* cDNA clone CF_WO0_53h08; 5' similar to ref|XP_968140.1| DIVDICTED: similar to Phosphoglycerate kinase - Tribolium castaneum. Sco Length = 651; score = 42.3 bits (19), expect = 0.18; identities = 25/31 (80%); strand = Plus / Plus

```
Query: 16 gctwawwttgcttgattcacttcwwyaagag 46
          ||| |  |||||||||||||||   |||||
Sbjct: 3  gctaatattgcttgattcacttctacaagag 33
```

>EL966147|EL966147.1 CF_WO0_53f04_SP6 Copepod Whole Organism, Normalized *Calanus finmarchicus* cDNA clone CF_WO0_53f04; 5' similar to ref|XP_394516.2| DIVDICTED: similar to Mediator complex subunit 17 CG7957-PA [Apis. Score Length = 682; score = 42.3 bits (19), expect = 0.18; identities = 25/31 (80%); strand = Plus / Plus

```
Query: 16 gctwawwttgcttgattcacttcwwyaagag 46
          ||| |  |||||||||||||||   |||||
Sbjct: 3  gctaatattgcttgattcacttctacaagag 33
```

>EL966074|EL966074.1 CF_WO0_52h01_SP6 Copepod Whole Organism, Normalized *Calanus finmarchicus* cDNA clone CF_WO0_52h01; 5' similar to ref|XP_967117.1| DIVDICTED: similar to CG10590-PA - Tribolium castaneum. Score = 294 bits Length = 729; score = 42.3 bits (19), expect = 0.18; identities = 25/31 (80%); strand = Plus / Plus

```
Query: 16 gctwawwttgcttgattcacttcwwyaagag 46
          ||| |  |||||||||||||||   |||||
Sbjct: 3  gctaatattgcttgattcacttctacaagag 33
```

>EL965994|EL965994.1 CF_WO0_52a01_SP6 Copepod Whole Organism, Normalized *Calanus finmarchicus* cDNA clone CF_WO0_52a01; 5' similar to ref|XP_624029.2| DIVDICTED: similar to CG16953-PA isoform 1 - Apis mellifera. Score = 70. Length = 684; score = 42.3 bits (19), expect = 0.18; identities = 25/31 (80%); strand = Plus / Plus

```
Query: 16 gctwawwttgcttgattcacttcwwyaagag 46
```

```
         ||| |  |||||||||||||||   |||||
Sbjct: 1  gctaatattgcttgattcacttctacaagag 31
```

>EL965985|EL965985.1 CF_WO0_51h04_SP6 Copepod Whole Organism, Normalized *Calanus finmarchicus* cDNA clone CF_WO0_51h04; 5' , mRNA sequence. Length = 712; score = 42.3 bits (19), expect = 0.18; identities = 25/31 (80%); strand = Plus / Plus

```
Query: 16 gctwawwttgcttgattcacttcwwyaagag 46
          ||| |  |||||||||||||||   |||||
Sbjct: 3  gctaatattgcttgattcacttctacaagag 33
```

>EL965800|EL965800.1 CF_WO0_49h07_SP6 Copepod Whole Organism, Normalized *Calanus finmarchicus* cDNA clone CF_WO0_49h07; 5' similar to ref|XP_319349.3| ENSANGP00000012398 - Anopheles gambiae str. PEST. Score = 223 bits (568) Length = 638; score = 42.3 bits (19), expect = 0.18; identities = 25/31 (80%); strand = Plus / Plus

```
Query: 16 gctwawwttgcttgattcacttcwwyaagag 46
          ||| |  |||||||||||||||   |||||
Sbjct: 3  gctaatattgcttgattcacttctacaagag 33
```

>EL965756|EL965756.1 CF_WO0_49d11_SP6 Copepod Whole Organism, Normalized *Calanus finmarchicus* cDNA clone CF_WO0_49d11; 5' similar to ref|XP_416025.1| DIVDICTED: similar to inner mitochondrial membrane peptidase 2. Score = Length = 737; score = 42.3 bits (19), expect = 0.18; identities = 25/31 (80%); strand = Plus / Plus

```
Query: 16 gctwawwttgcttgattcacttcwwyaagag 46
          ||| |  |||||||||||||||   |||||
Sbjct: 3  gctaatattgcttgattcacttctacaagag 33
```

>EL965752|EL965752.1 CF_WO0_49d07_SP6 Copepod Whole Organism, Normalized *Calanus finmarchicus* cDNA clone CF_WO0_49d07; 5' similar to ref|XP_001122102.1| DIVDICTED: similar to CG6729-PA - Apis mellifera. Score = 133 bits (3 Length = 657; score = 42.3 bits (19), expect = 0.18; identities = 25/31 (80%); strand = Plus / Plus

```
Query: 16 gctwawwttgcttgattcacttcwwyaagag 46
          ||| |  |||||||||||||||   |||||
Sbjct: 1  gctaatattgcttgattcacttctacaagag 31
```

>EL965746|EL965746.1 CF_WO0_49d01_SP6 Copepod Whole Organism, Normalized *Calanus finmarchicus* cDNA clone CF_WO0_49d01; 5' similar to ref|XP_623929.2| DIVDICTED: similar to CG5033-PA, isoform A - Apis mellifera. Score = 103 Length = 703; score = 42.3 bits (19), expect = 0.18; identities = 25/31 (80%); strand = Plus / Plus

```
Query: 16 gctwawwttgcttgattcacttcwwyaagag 46
          ||| |  |||||||||||||||   |||||
Sbjct: 1  gctaatattgcttgattcacttctacaagag 31
```

>EL965732|EL965732.1 CF_WO0_49b10_SP6 Copepod Whole Organism, Normalized *Calanus finmarchicus* cDNA clone CF_WO0_49b10; 5' similar to ref|XP_623368.1| DIVDICTED: similar to CG32699-PA,

partial - Apis mellifera. Score = 189 Length = 702; score = 42.3 bits (19), expect = 0.18; identities = 25/31 (80%); strand = Plus / Plus

```
Query: 16 gctwawwttgcttgattcacttcwwyaagag 46
          ||| |  |||||||||||||||   |||||
Sbjct: 1  gctaatattgcttgattcacttctacaagag 31
```

>EL965706|EL965706.1 CF_W00_48h07_SP6 Copepod Whole Organism, Normalized *Calanus finmarchicus* cDNA clone CF_W00_48h07; 5' similar to gb|EAT33766.1| Methylmalonyl-CoA carboxyltransferase 12S subunit, putative [Aedes. Score Length = 712; score = 42.3 bits (19), expect = 0.18; identities = 25/31 (80%); strand = Plus / Plus

```
Query: 16 gctwawwttgcttgattcacttcwwyaagag 46
          ||| |  |||||||||||||||   |||||
Sbjct: 1  gctaatattgcttgattcacttctacaagag 31
```

>EL965657|EL965657.1 CF_W00_48d05_SP6 Copepod Whole Organism, Normalized *Calanus finmarchicus* cDNA clone CF_W00_48d05; 5' similar to gb|AAH45222.1| Thiolase-prov protein - Xenopus laevis. Score = 291 bits (745), expect = 2 Length = 693; score = 42.3 bits (19), expect = 0.18; identities = 25/31 (80%); strand = Plus / Plus

```
Query: 16 gctwawwttgcttgattcacttcwwyaagag 46
          ||| |  |||||||||||||||   |||||
Sbjct: 1  gctaatattgcttgattcacttctacaagag 31
```

>EL965630|EL965630.1 CF_W00_48b02_SP6 Copepod Whole Organism, Normalized *Calanus finmarchicus* cDNA clone CF_W00_48b02; 5' similar to sp|Q9NB32|TCPD_AEDTR T-complex protein 1 subunit delta (TCP-1-delta) (CCT-delta). Score = Length = 610; score = 42.3 bits (19), expect = 0.18; identities = 25/31 (80%); strand = Plus / Plus

```
Query: 16 gctwawwttgcttgattcacttcwwyaagag 46
          ||| |  |||||||||||||||   |||||
Sbjct: 3  gctaatattgcttgattcacttctacaagag 33
```

>EL965612|EL965612.1 CF_W00_47h07_SP6 Copepod Whole Organism, Normalized *Calanus finmarchicus* cDNA clone CF_W00_47h07; 5' similar to ref|XP_974502.1| DIVDICTED: similar to CG3051-PA, isoform A - Tribolium castaneum. Score Length = 715; score = 42.3 bits (19), expect = 0.18; identities = 25/31 (80%); strand = Plus / Plus

```
Query: 16 gctwawwttgcttgattcacttcwwyaagag 46
          ||| |  |||||||||||||||   |||||
Sbjct: 35 gctaatattgcttgattcacttctacaagag 65
```

>EL965609|EL965609.1 CF_W00_47h04_SP6 Copepod Whole Organism, Normalized *Calanus finmarchicus* cDNA clone CF_W00_47h04; 5' similar to ref|XP_001344937.1| DIVDICTED: hypothetical protein - Danio rerio. Score = 209 bits (531) Length = 708; score = 42.3 bits (19), expect = 0.18; identities = 25/31 (80%); strand = Plus / Plus

```
Query: 16 gctwawwttgcttgattcacttcwwyaagag 46
```

```
         ||| |  |||||||||||||||   |||||
Sbjct: 3  gctaatattgcttgattcacttctacaagag 33
```

>EL965588|EL965588.1 CF_WO0_47f05_SP6 Copepod Whole Organism, Normalized *Calanus finmarchicus* cDNA clone CF_WO0_47f05; 5' similar to ref|XP_974835.1| DIVDICTED: similar to CG9426-PA - Tribolium castaneum. Score = 98.2 bits Length = 693; score = 42.3 bits (19), expect = 0.18; identities = 25/31 (80%); strand = Plus / Plus

```
Query: 16 gctwawwttgcttgattcacttcwwyaagag 46
          ||| |  |||||||||||||||   |||||
Sbjct: 8  gctaatattgcttgattcacttctacaagag 38
```

>EL965509|EL965509.1 CF_WO0_46g10_SP6 Copepod Whole Organism, Normalized *Calanus finmarchicus* cDNA clone CF_WO0_46g10; 5' similar to ref|XP_783899.1| DIVDICTED: similar to Ribophorin II - Strongylocentrotus purpuratus. Sco Length = 684; score = 42.3 bits (19), expect = 0.18; identities = 28/35 (80%); strand = Plus / Plus

```
Query: 12 ccaagctwawwttgcttgattcacttcwwyaagag 46
          ||||||| |  ||||||||||| ||||   |||||
Sbjct: 3  ccaagctaatattgcttgattctcttctacaagag 37
```

>EL965457|EL965457.1 CF_WO0_46c04_SP6 Copepod Whole Organism, Normalized *Calanus finmarchicus* cDNA clone CF_WO0_46c04; 5' similar to ref|XP_969275.1| DIVDICTED: similar to CG33166-PB, isoform B - Tribolium castaneum. Score Length = 701; score = 42.3 bits (19), expect = 0.18; identities = 25/31 (80%); strand = Plus / Plus

```
Query: 16 gctwawwttgcttgattcacttcwwyaagag 46
          ||| |  |||||||||||||||   |||||
Sbjct: 3  gctaatattgcttgattcacttctacaagag 33
```

>EL965444|EL965444.1 CF_WO0_46b03_SP6 Copepod Whole Organism, Normalized *Calanus finmarchicus* cDNA clone CF_WO0_46b03; 5' similar to ref|XP_967219.1| DIVDICTED: similar to CG8036-PB, isoform B isoform 1 [Tribolium. Score = Length = 724; score = 42.3 bits (19), expect = 0.18; identities = 25/31 (80%); strand = Plus / Plus

```
Query: 16 gctwawwttgcttgattcacttcwwyaagag 46
          ||| |  |||||||||||||||   |||||
Sbjct: 3  gctaatattgcttgattcacttctacaagag 33
```

>EL965386|EL965386.1 CF_WO0_45e03_SP6 Copepod Whole Organism, Normalized *Calanus finmarchicus* cDNA clone CF_WO0_45e03; 5' similar to ref|XP_968082.1| DIVDICTED: similar to CG9581-PA - Tribolium castaneum. Score = 154 bits Length = 685; score = 42.3 bits (19), expect = 0.18; identities = 25/31 (80%); strand = Plus / Plus

```
Query: 16 gctwawwttgcttgattcacttcwwyaagag 46
          ||| |  |||||||||||||||   |||||
Sbjct: 3  gctaatattgcttgattcacttctacaagag 33
```

>EL965377|EL965377.1 CF_WO0_45d06_SP6 Copepod Whole Organism, Normalized *Calanus finmarchicus* cDNA clone CF_WO0_45d06; 5' similar to ref|XP_319583.3| ENSANGP00000016715 - Anopheles

gambiae str. PEST. Score = 77.4 bits (189 Length = 742; score = 42.3 bits (19), expect = 0.18; identities = 25/31 (80%); strand = Plus / Plus

```
Query: 16 gctwawwttgcttgattcacttcwwyaagag 46
          ||| |  ||||||||||||||||   |||||
Sbjct: 3  gctaatattgcttgattcacttctacaagag 33
```

>EL965360|EL965360.1 CF_W00_45c01_SP6 Copepod Whole Organism, Normalized *Calanus finmarchicus* cDNA clone CF_W00_45c01; 5' , mRNA sequence. Length = 579; score = 42.3 bits (19), expect = 0.18; identities = 25/31 (80%); strand = Plus / Plus

```
Query: 16 gctwawwttgcttgattcacttcwwyaagag 46
          ||| |  ||||||||||||||||   |||||
Sbjct: 3  gctaatattgcttgattcacttctacaagag 33
```

>EL965357|EL965357.1 CF_W00_45b10_SP6 Copepod Whole Organism, Normalized *Calanus finmarchicus* cDNA clone CF_W00_45b10; 5' similar to gb|EAZ36294.1| hypothetical protein OsJ_019777 [Oryza sativa (japonica. Score = 56.6 bits Length = 680; score = 42.3 bits (19), expect = 0.18; identities = 25/31 (80%); strand = Plus / Plus

```
Query: 16 gctwawwttgcttgattcacttcwwyaagag 46
          ||| |  ||||||||||||||||   |||||
Sbjct: 3  gctaatattgcttgattcacttctacaagag 33
```

>EL774231|EL774231.1 CF_W00_44h02_SP6 Copepod Whole Organism, Normalized *Calanus finmarchicus* cDNA clone CF_W00_44h02; 5' , mRNA sequence. Length = 682; score = 42.3 bits (19), expect = 0.18; identities = 25/31 (80%); strand = Plus / Plus

```
Query: 16 gctwawwttgcttgattcacttcwwyaagag 46
          ||| |  ||||||||||||||||   |||||
Sbjct: 1  gctaatattgcttgattcacttctacaagag 31
```

>EL774195|EL774195.1 CF_W00_44e02_SP6 Copepod Whole Organism, Normalized *Calanus finmarchicus* cDNA clone CF_W00_44e02; 5' similar to ref|XP_972584.1| DIVDICTED: similar to CG8428-PC, isoform C - Tribolium castaneum. Score Length = 669; score = 42.3 bits (19), expect = 0.18; identities = 25/31 (80%); strand = Plus / Plus

```
Query: 16 gctwawwttgcttgattcacttcwwyaagag 46
          ||| |  ||||||||||||||||   |||||
Sbjct: 1  gctaatattgcttgattcacttctacaagag 31
```

>EL774102|EL774102.1 CF_W00_43e05_SP6 Copepod Whole Organism, Normalized *Calanus finmarchicus* cDNA clone CF_W00_43e05; 5' similar to gb|EAT47301.1| glutamate carboxypeptidase - Aedes aegypti. Score = 217 bits (552), expect Length = 728; score = 42.3 bits (19), expect = 0.18; identities = 25/31 (80%); strand = Plus / Plus

```
Query: 16 gctwawwttgcttgattcacttcwwyaagag 46
          ||| |  ||||||||||||||||   |||||
Sbjct: 3  gctaatattgcttgattcacttctacaagag 33
```

>EL774095|EL774095.1 CF_W00_43d10_SP6 Copepod Whole Organism, Normalized *Calanus finmarchicus* cDNA clone CF_W00_43d10; 5' similar to gb|AAY55317.1| IP12579p - Drosophila melanogaster. Score = 116 bits (291), expect = 1e-24 Length = 738; score = 42.3 bits (19), expect = 0.18; identities = 25/31 (80%); strand = Plus / Plus

```
Query: 16 gctwawwttgcttgattcacttcwwyaagag 46
          ||| |  |||||||||||||||   |||||
Sbjct: 3  gctaatattgcttgattcacttctacaagag 33
```

>EL774027|EL774027.1 CF_W00_42g01_SP6 Copepod Whole Organism, Normalized *Calanus finmarchicus* cDNA clone CF_W00_42g01; 5' similar to ref|XP_317601.3| ENSANGP00000010004 - Anopheles gambiae str. PEST. Score = 72.8 bits (177 Length = 715; score = 42.3 bits (19), expect = 0.18; identities = 25/31 (80%); strand = Plus / Plus

```
Query: 16 gctwawwttgcttgattcacttcwwyaagag 46
          ||| |  |||||||||||||||   |||||
Sbjct: 3  gctaatattgcttgattcacttctacaagag 33
```

>EL774018|EL774018.1 CF_W00_42f04_SP6 Copepod Whole Organism, Normalized *Calanus finmarchicus* cDNA clone CF_W00_42f04; 5' similar to ref|XP_320246.3| ENSANGP00000023679 - Anopheles gambiae str. PEST. Score = 97.1 bits (240 Length = 742; score = 42.3 bits (19), expect = 0.18; identities = 25/31 (80%); strand = Plus / Plus

```
Query: 16 gctwawwttgcttgattcacttcwwyaagag 46
          ||| |  |||||||||||||||   |||||
Sbjct: 3  gctaatattgcttgattcacttctacaagag 33
```

>EL773936|EL773936.1 CF_W00_41g04_SP6 Copepod Whole Organism, Normalized *Calanus finmarchicus* cDNA clone CF_W00_41g04; 5' similar to ref|XP_624703.1| DIVDICTED: similar to germ cell-less CG8411-PA - Apis mellifera. Score = Length = 722; score = 42.3 bits (19), expect = 0.18; identities = 25/31 (80%); strand = Plus / Plus

```
Query: 16 gctwawwttgcttgattcacttcwwyaagag 46
          ||| |  |||||||||||||||   |||||
Sbjct: 3  gctaatattgcttgattcacttctacaagag 33
```

>EL773935|EL773935.1 CF_W00_41g03_SP6 Copepod Whole Organism, Normalized *Calanus finmarchicus* cDNA clone CF_W00_41g03; 5' similar to ref|XP_624703.1| DIVDICTED: similar to germ cell-less CG8411-PA - Apis mellifera. Score = Length = 643; score = 42.3 bits (19), expect = 0.18; identities = 25/31 (80%); strand = Plus / Plus

```
Query: 16 gctwawwttgcttgattcacttcwwyaagag 46
          ||| |  |||||||||||||||   |||||
Sbjct: 3  gctaatattgcttgattcacttctacaagag 33
```

>EL773934|EL773934.1 CF_W00_41g02_SP6 Copepod Whole Organism, Normalized *Calanus finmarchicus* cDNA clone CF_W00_41g02; 5' similar to ref|XP_624703.1| DIVDICTED: similar to germ cell-less

CG8411-PA - Apis mellifera. Score = Length = 737; score = 42.3 bits (19), expect = 0.18; identities = 25/31 (80%); strand = Plus / Plus

```
Query: 16 gctwawwttgcttgattcacttcwwyaagag 46
          ||| |  ||||||||||||||||   |||||
Sbjct: 3  gctaatattgcttgattcacttctacaagag 33
```

>EL773876|EL773876.1 CF_W00_41b02_SP6 Copepod Whole Organism, Normalized *Calanus finmarchicus* cDNA clone CF_W00_41b02; 5' similar to ref|XP_394995.2| DIVDICTED: similar to MAP kinase-activated protein kinase 2. Score = 138 Length = 700; score = 42.3 bits (19), expect = 0.18; identities = 25/31 (80%); strand = Plus / Plus

```
Query: 16 gctwawwttgcttgattcacttcwwyaagag 46
          ||| |  ||||||||||||||||   |||||
Sbjct: 3  gctaatattgcttgattcacttctacaagag 33
```

>EL773835|EL773835.1 CF_W00_40f07_SP6 Copepod Whole Organism, Normalized *Calanus finmarchicus* cDNA clone CF_W00_40f07; 5' similar to ref|XP_320057.3| ENSANGP00000012341 - Anopheles gambiae str. PEST. Score = 150 bits (378) Length = 694; score = 42.3 bits (19), expect = 0.18; identities = 25/31 (80%); strand = Plus / Plus

```
Query: 16 gctwawwttgcttgattcacttcwwyaagag 46
          ||| |  ||||||||||||||||   |||||
Sbjct: 3  gctaatattgcttgattcacttctacaagag 33
```

>EL773752|EL773752.1 CF_W00_39g04_SP6 Copepod Whole Organism, Normalized *Calanus finmarchicus* cDNA clone CF_W00_39g04; 5' similar to gb|AAU84927.1| putative 26S protease regulatory subunit 8 - Toxoptera citricida. Score = Length = 691; score = 42.3 bits (19), expect = 0.18; identities = 25/31 (80%); strand = Plus / Plus

```
Query: 16 gctwawwttgcttgattcacttcwwyaagag 46
          ||| |  ||||||||||||||||   |||||
Sbjct: 3  gctaatattgcttgattcacttctacaagag 33
```

>EL773705|EL773705.1 CF_W00_39c05_SP6 Copepod Whole Organism, Normalized *Calanus finmarchicus* cDNA clone CF_W00_39c05; 5' similar to ref|XP_970856.1| DIVDICTED: similar to CG9140-PA - Tribolium castaneum. Score = 322 bits Length = 701; score = 42.3 bits (19), expect = 0.18; identities = 25/31 (80%); strand = Plus / Plus

```
Query: 16 gctwawwttgcttgattcacttcwwyaagag 46
          ||| |  ||||||||||||||||   |||||
Sbjct: 1  gctaatattgcttgattcacttctacaagag 31
```

>EL773648|EL773648.1 CF_W00_38f08_SP6 Copepod Whole Organism, Normalized *Calanus finmarchicus* cDNA clone CF_W00_38f08; 5' similar to ref|XP_970345.1| DIVDICTED: similar to CG11199-PA, isoform A - Tribolium castaneum. Score Length = 694; score = 42.3 bits (19), expect = 0.18; identities = 25/31 (80%); strand = Plus / Plus

```
Query: 16 gctwawwttgcttgattcacttcwwyaagag 46
```

||| | ||||||||||||||| |||||
Sbjct: 3 gctaatattgcttgattcacttctacaagag 33

>EL773609|EL773609.1 CF_WOO_38c05_SP6 Copepod Whole Organism, Normalized *Calanus finmarchicus* cDNA clone CF_WOO_38c05; 5' , mRNA sequence. Length = 686; score = 42.3 bits (19), expect = 0.18; identities = 25/31 (80%); strand = Plus / Plus

Query: 16 gctwawwttgcttgattcacttcwwyaagag 46
         ||| | ||||||||||||||| |||||
Sbjct: 3 gctaatattgcttgattcacttctacaagag 33

>EL773607|EL773607.1 CF_WOO_38c03_SP6 Copepod Whole Organism, Normalized *Calanus finmarchicus* cDNA clone CF_WOO_38c03; 5' similar to sp|Q9NB32|TCPD_AEDTR T-complex protein 1 subunit delta (TCP-1-delta) (CCT-delta). Score = Length = 609; score = 42.3 bits (19), expect = 0.18; identities = 25/31 (80%); strand = Plus / Plus

Query: 16 gctwawwttgcttgattcacttcwwyaagag 46
         ||| | ||||||||||||||| |||||
Sbjct: 3 gctaatattgcttgattcacttctacaagag 33

>EL773606|EL773606.1 CF_WOO_38c02_SP6 Copepod Whole Organism, Normalized *Calanus finmarchicus* cDNA clone CF_WOO_38c02; 5' , mRNA sequence. Length = 638; score = 42.3 bits (19), expect = 0.18; identities = 25/31 (80%); strand = Plus / Plus

Query: 16 gctwawwttgcttgattcacttcwwyaagag 46
         ||| | ||||||||||||||| |||||
Sbjct: 1 gctaatattgcttgattcacttctacaagag 31

>EL773605|EL773605.1 CF_WOO_38c01_SP6 Copepod Whole Organism, Normalized *Calanus finmarchicus* cDNA clone CF_WOO_38c01; 5' , mRNA sequence. Length = 634; score = 42.3 bits (19), expect = 0.18; identities = 25/31 (80%); strand = Plus / Plus

Query: 16 gctwawwttgcttgattcacttcwwyaagag 46
         ||| | ||||||||||||||| |||||
Sbjct: 1 gctaatattgcttgattcacttctacaagag 31

>EL773599|EL773599.1 CF_WOO_38b07_SP6 Copepod Whole Organism, Normalized *Calanus finmarchicus* cDNA clone CF_WOO_38b07; 5' similar to ref|XP_975557.1| DIVDICTED: similar to CG9060-PA - Tribolium castaneum. Score = 259 bits Length = 719; score = 42.3 bits (19), expect = 0.18; identities = 25/31 (80%); strand = Plus / Plus

Query: 16 gctwawwttgcttgattcacttcwwyaagag 46
         ||| | ||||||||||||||| |||||
Sbjct: 3 gctaatattgcttgattcacttctacaagag 33

>EL773520|EL773520.1 CF_WOO_37c10_SP6 Copepod Whole Organism, Normalized *Calanus finmarchicus* cDNA clone CF_WOO_37c10; 5' similar to ref|XP_972566.1| DIVDICTED: similar to CG4685-PA, isoform A - Tribolium castaneum. Score Length = 705; score = 42.3 bits (19), expect = 0.18; identities = 25/31 (80%); strand = Plus / Plus

```
Query: 16 gctwawwttgcttgattcacttcwwyaagag 46
          ||| |  |||||||||||||||   |||||
Sbjct: 3  gctaatattgcttgattcacttctacaagag 33
```

>EL773506|EL773506.1 CF_W00_37b08_SP6 Copepod Whole Organism, Normalized *Calanus finmarchicus* cDNA clone CF_W00_37b08; 5' , mRNA sequence. Length = 650; score = 42.3 bits (19), expect = 0.18; identities = 25/31 (80%); strand = Plus / Plus

```
Query: 16 gctwawwttgcttgattcacttcwwyaagag 46
          ||| |  |||||||||||||||   |||||
Sbjct: 3  gctaatattgcttgattcacttctacaagag 33
```

>EL773482|EL773482.1 CF_W00_36h08_SP6 Copepod Whole Organism, Normalized *Calanus finmarchicus* cDNA clone CF_W00_36h08; 5' similar to ref|XP_001372429.1| DIVDICTED: similar to myosin light chain kinase [Monodelphis. Score = Length = 627; score = 42.3 bits (19), expect = 0.18; identities = 25/31 (80%); strand = Plus / Plus

```
Query: 16 gctwawwttgcttgattcacttcwwyaagag 46
          ||| |  |||||||||||||||   |||||
Sbjct: 3  gctaatattgcttgattcacttctacaagag 33
```

>EL773435|EL773435.1 CF_W00_36d07_SP6 Copepod Whole Organism, Normalized *Calanus finmarchicus* cDNA clone CF_W00_36d07; 5' similar to ref|NP_001072639.1| hypothetical protein LOC780095 - Xenopus tropicalis. Score = 217 bits Length = 705; score = 42.3 bits (19), expect = 0.18; identities = 25/31 (80%); strand = Plus / Plus

```
Query: 16 gctwawwttgcttgattcacttcwwyaagag 46
          ||| |  |||||||||||||||   |||||
Sbjct: 3  gctaatattgcttgattcacttctacaagag 33
```

>EL773371|EL773371.1 CF_W00_35g03_SP6 Copepod Whole Organism, Normalized *Calanus finmarchicus* cDNA clone CF_W00_35g03; 5' , mRNA sequence. Length = 698; score = 42.3 bits (19), expect = 0.18; identities = 25/31 (80%); strand = Plus / Plus

```
Query: 16 gctwawwttgcttgattcacttcwwyaagag 46
          ||| |  |||||||||||||||   |||||
Sbjct: 3  gctaatattgcttgattcacttctacaagag 33
```

>EL697512|EL697512.1 CF_W00_34g06_SP6 Copepod Whole Organism, Normalized *Calanus finmarchicus* cDNA clone CF_W00_34g06; 5' , mRNA sequence. Length = 619; score = 42.3 bits (19), expect = 0.18; identities = 25/31 (80%); strand = Plus / Plus

```
Query: 16 gctwawwttgcttgattcacttcwwyaagag 46
          ||| |  |||||||||||||||   |||||
Sbjct: 3  gctaatattgcttgattcacttctacaagag 33
```

>EL697511|EL697511.1 CF_WO0_34g04_SP6 Copepod Whole Organism, Normalized *Calanus finmarchicus* cDNA clone CF_WO0_34g04; 5’ , mRNA sequence. Length = 673; score = 42.3 bits (19), expect = 0.18; identities = 25/31 (80%); strand = Plus / Plus

Query: 16 gctwawwttgcttgattcacttcwwyaagag 46
          ||| | |||||||||||||||| |||||
Sbjct: 3 gctaatattgcttgattcacttctacaagag 33

>EL697482|EL697482.1 CF_WO0_34d11_SP6 Copepod Whole Organism, Normalized *Calanus finmarchicus* cDNA clone CF_WO0_34d11; 5’ , mRNA sequence. Length = 688; score = 42.3 bits (19), expect = 0.18; identities = 25/31 (80%); strand = Plus / Plus

Query: 16 gctwawwttgcttgattcacttcwwyaagag 46
          ||| | |||||||||||||||| |||||
Sbjct: 3 gctaatattgcttgattcacttctacaagag 33

>EL697463|EL697463.1 CF_WO0_34c04_SP6 Copepod Whole Organism, Normalized *Calanus finmarchicus* cDNA clone CF_WO0_34c04; 5’ similar to ref|XP_395835.2| DIVDICTED: similar to CG11990-PA - Apis mellifera. Score = 248 bits (634 Length = 701; score = 42.3 bits (19), expect = 0.18; identities = 25/31 (80%); strand = Plus / Plus

Query: 16 gctwawwttgcttgattcacttcwwyaagag 46
          ||| | |||||||||||||||| |||||
Sbjct: 3 gctaatattgcttgattcacttctacaagag 33

>EL697402|EL697402.1 CF_WO0_33f02_SP6 Copepod Whole Organism, Normalized *Calanus finmarchicus* cDNA clone CF_WO0_33f02; 5’ similar to gb|EAT40665.1| toll - Aedes aegypti. Score = 79.7 bits (195), expect = 1e-13, mRNA sequen Length = 677; score = 42.3 bits (19), expect = 0.18; identities = 25/31 (80%); strand = Plus / Plus

Query: 16 gctwawwttgcttgattcacttcwwyaagag 46
          ||| | |||||||||||||||| |||||
Sbjct: 3 gctaatattgcttgattcacttctacaagag 33

>EL697400|EL697400.1 CF_WO0_33e12_SP6 Copepod Whole Organism, Normalized *Calanus finmarchicus* cDNA clone CF_WO0_33e12; 5’ , mRNA sequence. Length = 537; score = 42.3 bits (19), expect = 0.18; identities = 25/31 (80%); strand = Plus / Plus

Query: 16 gctwawwttgcttgattcacttcwwyaagag 46
          ||| | |||||||||||||||| |||||
Sbjct: 3 gctaatattgcttgattcacttctacaagag 33

>EL697397|EL697397.1 CF_WO0_33e09_SP6 Copepod Whole Organism, Normalized *Calanus finmarchicus* cDNA clone CF_WO0_33e09; 5’ similar to gb|AAI14082.1| NAPE-PLD protein - Bos taurus. Score = 125 bits (314), expect = 2e-27, mRN Length = 709; score = 42.3 bits (19), expect = 0.18; identities = 25/31 (80%); strand = Plus / Plus

Query: 16 gctwawwttgcttgattcacttcwwyaagag 46
          ||| | |||||||||||||||| |||||

```
Sbjct: 3  gctaatattgcttgattcacttcttcaagag 33
```

>EL697362|EL697362.1 CF_WO0_33b10_SP6 Copepod Whole Organism, Normalized *Calanus finmarchicus* cDNA clone CF_WO0_33b10; 5' similar to ref|XP_969775.1| DIVDICTED: similar to CG4420-PA - Tribolium castaneum. Score = 184 bits Length = 685; score = 42.3 bits (19), expect = 0.18; identities = 25/31 (80%); strand = Plus / Plus

```
Query: 16 gctwawwttgcttgattcacttcwwyaagag 46
          ||| |  ||||||||||||||||   |||||
Sbjct: 1  gctaatattgcttgattcacttctacaagag 31
```

>EL697144|EL697144.1 CF_WO0_30h05_SP6 Copepod Whole Organism, Normalized *Calanus finmarchicus* cDNA clone CF_WO0_30h05; 5' similar to ref|XP_623365.1| DIVDICTED: similar to UBL3 CG9038-PA, isoform A isoform 2 [Apis. Score = Length = 719; score = 42.3 bits (19), expect = 0.18; identities = 25/31 (80%); strand = Plus / Plus

```
Query: 16 gctwawwttgcttgattcacttcwwyaagag 46
          ||| |  ||||||||||||||||   |||||
Sbjct: 1  gctaatattgcttgattcacttctacaagag 31
```

>EL697074|EL697074.1 CF_WO0_30b07_SP6 Copepod Whole Organism, Normalized *Calanus finmarchicus* cDNA clone CF_WO0_30b07; 5' similar to ref|XP_972453.1| DIVDICTED: similar to CG6601-PA - Tribolium castaneum. Score = 367 bits Length = 656; score = 42.3 bits (19), expect = 0.18; identities = 25/31 (80%); strand = Plus / Plus

```
Query: 16 gctwawwttgcttgattcacttcwwyaagag 46
          ||| |  ||||||||||||||||   |||||
Sbjct: 3  gctaatattgcttgattcacttctacaagag 33
```

>EL697001|EL697001.1 CF_WO0_29d06_SP6 Copepod Whole Organism, Normalized *Calanus finmarchicus* cDNA clone CF_WO0_29d06; 5' similar to ref|XP_001031569.1| hypothetical protein TTHERM_00773160 [Tetrahymena thermophila. Score Length = 704; score = 42.3 bits (19), expect = 0.18; identities = 25/31 (80%); strand = Plus / Plus

```
Query: 16 gctwawwttgcttgattcacttcwwyaagag 46
          ||| |  ||||||||||||||||   |||||
Sbjct: 3  gctaatattgcttgattcacttctacaagag 33
```

>EL696997|EL696997.1 CF_WO0_29d02_SP6 Copepod Whole Organism, Normalized *Calanus finmarchicus* cDNA clone CF_WO0_29d02; 5' similar to gb|ABG02632.1| CG5976 - Drosophila melanogaster. Score = 152 bits (385), expect = 1e-35, Length = 706; score = 42.3 bits (19), expect = 0.18; identities = 25/31 (80%); strand = Plus / Plus

```
Query: 16 gctwawwttgcttgattcacttcwwyaagag 46
          ||| |  ||||||||||||||||   |||||
Sbjct: 1  gctaatattgcttgattcacttctacaagag 31
```

>EL696948|EL696948.1 CF_WO0_28g10_SP6 Copepod Whole Organism, Normalized *Calanus finmarchicus* cDNA clone CF_WO0_28g10; 5' similar to ref|XP_001353863.1| GA10406-PA - Drosophila

pseudoobscura. Score = 109 bits (273), expect Length = 668; score = 42.3 bits (19), expect = 0.18; identities = 25/31 (80%); strand = Plus / Plus

```
Query: 16 gctwawwttgcttgattcacttcwwyaagag 46
          ||| |  |||||||||||||||   |||||
Sbjct: 1  gctaatattgcttgattcacttctacaagag 31
```

>EL696830|EL696830.1 CF_W00_27e09_SP6 Copepod Whole Organism, Normalized *Calanus finmarchicus* cDNA clone CF_W00_27e09; 5' similar to ref|XP_795021.2| DIVDICTED: hypothetical protein - Strongylocentrotus purpuratus. Score = Length = 523; score = 42.3 bits (19), expect = 0.18; identities = 25/31 (80%); strand = Plus / Plus

```
Query: 16 gctwawwttgcttgattcacttcwwyaagag 46
          ||| |  |||||||||||||||   |||||
Sbjct: 3  gctaatattgcttgattcacttctacaagag 33
```

>EL696823|EL696823.1 CF_W00_27e02_SP6 Copepod Whole Organism, Normalized *Calanus finmarchicus* cDNA clone CF_W00_27e02; 5' similar to ref|XP_971904.1| DIVDICTED: similar to CG18769-PB, isoform B - Tribolium castaneum. Score Length = 759; score = 42.3 bits (19), expect = 0.18; identities = 25/31 (80%); strand = Plus / Plus

```
Query: 16 gctwawwttgcttgattcacttcwwyaagag 46
          ||| |  |||||||||||||||   |||||
Sbjct: 3  gctaatattgcttgattcacttctacaagag 33
```

>EL696767|EL696767.1 CF_W00_26h06_SP6 Copepod Whole Organism, Normalized *Calanus finmarchicus* cDNA clone CF_W00_26h06; 5' similar to ref|XP_534306.2| DIVDICTED: similar to CDA02 protein isoform 1 - Canis familiaris. Score Length = 686; score = 42.3 bits (19), expect = 0.18; identities = 25/31 (80%); strand = Plus / Plus

```
Query: 16 gctwawwttgcttgattcacttcwwyaagag 46
          ||| |  |||||||||||||||   |||||
Sbjct: 3  gctaatattgcttgattcacttctacaagag 33
```

>EL696733|EL696733.1 CF_W00_26e08_SP6 Copepod Whole Organism, Normalized *Calanus finmarchicus* cDNA clone CF_W00_26e08; 5' similar to dbj|BAE27597.1| unnamed protein product - Mus musculus. Score = 278 bits (710), expect = Length = 657; score = 42.3 bits (19), expect = 0.18; identities = 25/31 (80%); strand = Plus / Plus

```
Query: 16 gctwawwttgcttgattcacttcwwyaagag 46
          ||| |  |||||||||||||||   |||||
Sbjct: 3  gctaatattgcttgattcacttctacaagag 33
```

>EL586603|EL586603.1 CF_W00_24b08_SP6 Copepod Whole Organism, Normalized *Calanus finmarchicus* cDNA clone CF_W00_24b08; 5' similar to XP_001121272.1 CG7759-PB, isoform B [Apis mellifera]. Score=50.83, Expect=0.000013, mRNA Length = 384; score = 42.3 bits (19), expect = 0.18; identities = 25/31 (80%); strand = Plus / Plus

```
Query: 16 gctwawwttgcttgattcacttcwwyaagag 46
```

```
         ||| |  |||||||||||||||   |||||
Sbjct: 3  gctaatattgcttgattcacttctacaagag 33
```

>EL586495|EL586495.1 CF_W00_23a03_SP6 Copepod Whole Organism, Normalized *Calanus finmarchicus* cDNA clone CF_W00_23a03; 5' , mRNA sequence. Length = 680; score = 42.3 bits (19), expect = 0.18; identities = 25/31 (80%); strand = Plus / Plus

```
Query: 16 gctwawwttgcttgattcacttcwwyaagag 46
          ||| |  |||||||||||||||   |||||
Sbjct: 3  gctaatattgcttgattcacttctacaagag 33
```

>EL586488|EL586488.1 CF_W00_22h08_SP6 Copepod Whole Organism, Normalized *Calanus finmarchicus* cDNA clone CF_W00_22h08; 5' similar to XP_396337.1 SP2637 CG5473-PA, isoform A [Apis mellifera]. Score=209.92, Expect=6.30E-53, Length = 674; score = 42.3 bits (19), expect = 0.18; identities = 25/31 (80%); strand = Plus / Plus

```
Query: 16 gctwawwttgcttgattcacttcwwyaagag 46
          ||| |  |||||||||||||||   |||||
Sbjct: 3  gctaatattgcttgattcacttctacaagag 33
```

>EL586444|EL586444.1 CF_W00_22d12_SP6 Copepod Whole Organism, Normalized *Calanus finmarchicus* cDNA clone CF_W00_22d12; 5' similar to NP_001011679.1 betaine-homocysteine methyltransferase [Bos taurus]. Score=165.62, Expect= Length = 683; score = 42.3 bits (19), expect = 0.18; identities = 25/31 (80%); strand = Plus / Plus

```
Query: 16 gctwawwttgcttgattcacttcwwyaagag 46
          ||| |  |||||||||||||||   |||||
Sbjct: 3  gctaatattgcttgattcacttctacaagag 33
```

>EL586435|EL586435.1 CF_W00_22d02_SP6 Copepod Whole Organism, Normalized *Calanus finmarchicus* cDNA clone CF_W00_22d02; 5' similar to XP_974487.1 CG3194-PA [Tribolium castaneum]. Score=147.52, Expect=4.10E-34, mRNA sequence Length = 691; score = 42.3 bits (19), expect = 0.18; identities = 25/31 (80%); strand = Plus / Plus

```
Query: 16 gctwawwttgcttgattcacttcwwyaagag 46
          ||| |  |||||||||||||||   |||||
Sbjct: 1  gctaatattgcttgattcacttctacaagag 31
```

>EL586193|EL586193.1 CF_W00_19g04_SP6 Copepod Whole Organism, Normalized *Calanus finmarchicus* cDNA clone CF_W00_19g04; 5' similar to XP_396195.2 Imitation SWI CG8625-PA, isoform A isoform 1 [Apis mellifera]. Score=207.22, Length = 671; score = 42.3 bits (19), expect = 0.18; identities = 25/31 (80%); strand = Plus / Plus

```
Query: 16 gctwawwttgcttgattcacttcwwyaagag 46
          ||| |  |||||||||||||||   |||||
Sbjct: 1  gctaatattgcttgattcacttctacaagag 31
```

>EL586100|EL586100.1 CF_W00_18g04_SP6 Copepod Whole Organism, Normalized *Calanus finmarchicus* cDNA clone CF_W00_18g04; 5' similar to XP_972453.1 CG6601-PA [Tribolium castaneum].

Score=211.46, Expect=2.30E-53, mRNA sequence Length = 699; score = 42.3 bits (19), expect = 0.18; identities = 25/31 (80%); strand = Plus / Plus

```
Query: 16 gctwawwttgcttgattcacttcwwyaagag 46
          ||| |  |||||||||||||||   |||||
Sbjct: 3  gctaatattgcttgattcacttctacaagag 33
```

>EL586038|EL586038.1 CF_WO0_18a07_SP6 Copepod Whole Organism, Normalized *Calanus finmarchicus* cDNA clone CF_WO0_18a07; 5' similar to XP_001254080.1 methylcrotonoyl-Coenzyme A carboxylase 2 (beta), partial [Bos taurus]. Sco Length = 711; score = 42.3 bits (19), expect = 0.18; identities = 25/31 (80%); strand = Plus / Plus

```
Query: 16 gctwawwttgcttgattcacttcwwyaagag 46
          ||| |  |||||||||||||||   |||||
Sbjct: 3  gctaatattgcttgattcacttctacaagag 33
```

>EL586025|EL586025.1 CF_WO0_17h05_SP6 Copepod Whole Organism, Normalized *Calanus finmarchicus* cDNA clone CF_WO0_17h05; 5', mRNA sequence. Length = 721; score = 42.3 bits (19), expect = 0.18; identities = 25/31 (80%); strand = Plus / Plus

```
Query: 16 gctwawwttgcttgattcacttcwwyaagag 46
          ||| |  |||||||||||||||   |||||
Sbjct: 3  gctaatattgcttgattcacttctacaagag 33
```

>EL585984|EL585984.1 CF_WO0_17d11_SP6 Copepod Whole Organism, Normalized *Calanus finmarchicus* cDNA clone CF_WO0_17d11; 5' similar to ABF18063.1 oligosaccharyltransferase gamma subunit [Aedes aegypti]. Score=225.71, Expect= Length = 678; score = 42.3 bits (19), expect = 0.18; identities = 25/31 (80%); strand = Plus / Plus

```
Query: 16 gctwawwttgcttgattcacttcwwyaagag 46
          ||| |  |||||||||||||||   |||||
Sbjct: 3  gctaatattgcttgattcacttctacaagag 33
```

>EL585922|EL585922.1 CF_WO0_16g08_SP6 Copepod Whole Organism, Normalized *Calanus finmarchicus* cDNA clone CF_WO0_16g08; 5', mRNA sequence. Length = 668; score = 42.3 bits (19), expect = 0.18; identities = 25/31 (80%); strand = Plus / Plus

```
Query: 16 gctwawwttgcttgattcacttcwwyaagag 46
          ||| |  |||||||||||||||   |||||
Sbjct: 3  gctaatattgcttgattcacttctacaagag 33
```

>EL585919|EL585919.1 CF_WO0_16g05_SP6 Copepod Whole Organism, Normalized *Calanus finmarchicus* cDNA clone CF_WO0_16g05; 5' similar to NP_610562.1 CG12214-PA, isoform A [Drosophila melanogaster]. Score=73.56, Expect=6.50E-12 Length = 647; score = 42.3 bits (19), expect = 0.18; identities = 25/31 (80%); strand = Plus / Plus

```
Query: 16 gctwawwttgcttgattcacttcwwyaagag 46
          ||| |  |||||||||||||||   |||||
Sbjct: 3  gctaatattgcttgattcacttctacaagag 33
```

>EL585820|EL585820.1 CF_W00_15f09_SP6 Copepod Whole Organism, Normalized *Calanus finmarchicus* cDNA clone CF_W00_15f09; 5' similar to NP_001074106.1 hypothetical protein LOC791155 [Danio rerio]. Score=131.34, Expect=2.80E-2 Length = 673; score = 42.3 bits (19), expect = 0.18; identities = 25/31 (80%); strand = Plus / Plus

```
Query: 16 gctwawwttgcttgattcacttcwwyaagag 46
          ||| |  |||||||||||||||   |||||
Sbjct: 1  gctaatattgcttgattcacttctacaagag 31
```

>EL585768|EL585768.1 CF_W00_15b01_SP6 Copepod Whole Organism, Normalized *Calanus finmarchicus* cDNA clone CF_W00_15b01; 5' similar to XP_970938.1 centaurin, alpha 1 [Tribolium castaneum]. Score=234.19, Expect=2.60E-60, mRNA Length = 622; score = 42.3 bits (19), expect = 0.18; identities = 25/31 (80%); strand = Plus / Plus

```
Query: 16 gctwawwttgcttgattcacttcwwyaagag 46
          ||| |  |||||||||||||||   |||||
Sbjct: 3  gctaatattgcttgattcacttctacaagag 33
```

>EL585678|EL585678.1 CF_W00_14b06_SP6 Copepod Whole Organism, Normalized *Calanus finmarchicus* cDNA clone CF_W00_14b06; 5' similar to NP_990723.1 myosin light chain kinase 2, skeletal muscle [Gallus gallus]. Score=75.87, Ex Length = 573; score = 42.3 bits (19), expect = 0.18; identities = 25/31 (80%); strand = Plus / Plus

```
Query: 16 gctwawwttgcttgattcacttcwwyaagag 46
          ||| |  |||||||||||||||   |||||
Sbjct: 3  gctaatattgcttgattcacttctacaagag 33
```

>EL585670|EL585670.1 CF_W00_14a10_SP6 Copepod Whole Organism, Normalized *Calanus finmarchicus* cDNA clone CF_W00_14a10; 5' similar to XP_310169.3 ENSANGP00000019950 [Anopheles gambiae str. PEST]. Score=135.58, Expect=1.60E- Length = 702; score = 42.3 bits (19), expect = 0.18; identities = 25/31 (80%); strand = Plus / Plus

```
Query: 16 gctwawwttgcttgattcacttcwwyaagag 46
          ||| |  |||||||||||||||   |||||
Sbjct: 3  gctaatattgcttgattcacttctacaagag 33
```

>EL585652|EL585652.1 CF_W00_13h03_SP6 Copepod Whole Organism, Normalized *Calanus finmarchicus* cDNA clone CF_W00_13h03; 5' similar to XP_001021675.1 ATP:guanido phosphotransferase, C-terminal catalytic domain containing pro Length = 644; score = 42.3 bits (19), expect = 0.18; identities = 25/31 (80%); strand = Plus / Plus

```
Query: 16 gctwawwttgcttgattcacttcwwyaagag 46
          ||| |  |||||||||||||||   |||||
Sbjct: 3  gctaatattgcttgattcacttctacaagag 33
```

>EL585566|EL585566.1 CF_W00_12h04_SP6 Copepod Whole Organism, Normalized *Calanus finmarchicus* cDNA clone CF_W00_12h04; 5', mRNA sequence. Length = 691; score = 42.3 bits (19), expect = 0.18; identities = 25/31 (80%); strand = Plus / Plus

```
Query: 16 gctwawwttgcttgattcacttcwwyaagag 46
          ||| |  |||||||||||||||   |||||
Sbjct: 3  gctaatattgcttgattcacttctacaagag 33
```

>EL585540|EL585540.1 CF_WO0_12e11_SP6 Copepod Whole Organism, Normalized *Calanus finmarchicus* cDNA clone CF_WO0_12e11; 5' similar to EAT37397.1 modifier of mdg4 [Aedes aegypti]. Score=106.3, Expect=1.20E-21, mRNA sequence. Length = 736; score = 42.3 bits (19), expect = 0.18; identities = 25/31 (80%); strand = Plus / Plus

```
Query: 16 gctwawwttgcttgattcacttcwwyaagag 46
          ||| |  |||||||||||||||   |||||
Sbjct: 3  gctaatattgcttgattcacttctacaagag 33
```

>EL585522|EL585522.1 CF_WO0_12d02_SP6 Copepod Whole Organism, Normalized *Calanus finmarchicus* cDNA clone CF_WO0_12d02; 5' similar to XP_967617.1 CG2947-PA, isoform A [Tribolium castaneum]. Score=119.4, Expect=1.20E-25, mRN Length = 685; score = 42.3 bits (19), expect = 0.18; identities = 25/31 (80%); strand = Plus / Plus

```
Query: 16 gctwawwttgcttgattcacttcwwyaagag 46
          ||| |  |||||||||||||||   |||||
Sbjct: 3  gctaatattgcttgattcacttctacaagag 33
```

>EL585520|EL585520.1 CF_WO0_12c12_SP6 Copepod Whole Organism, Normalized *Calanus finmarchicus* cDNA clone CF_WO0_12c12; 5' similar to XP_623198.1 Coatomer subunit alpha (Alpha-coat protein) (Alpha-COP) (HEPCOP) (HEP-COP) is Length = 698; score = 42.3 bits (19), expect = 0.18; identities = 25/31 (80%); strand = Plus / Plus

```
Query: 16 gctwawwttgcttgattcacttcwwyaagag 46
          ||| |  |||||||||||||||   |||||
Sbjct: 3  gctaatattgcttgattcacttctacaagag 33
```

>EL585488|EL585488.1 CF_WO0_11h11_SP6 Copepod Whole Organism, Normalized *Calanus finmarchicus* cDNA clone CF_WO0_11h11; 5' similar to XP_391841.1 14-3-3-like protein (Leonardo protein) (14-3-3 zeta) isoform 1 [Apis mellifer Length = 679; score = 42.3 bits (19), expect = 0.18; identities = 25/31 (80%); strand = Plus / Plus

```
Query: 16 gctwawwttgcttgattcacttcwwyaagag 46
          ||| |  |||||||||||||||   |||||
Sbjct: 1  gctaatattgcttgattcacttctacaagag 31
```

>EL585405|EL585405.1 CF_WO0_11a10_SP6 Copepod Whole Organism, Normalized *Calanus finmarchicus* cDNA clone CF_WO0_11a10; 5' similar to AAH82397.1 MGC81928 protein [Xenopus laevis]. Score=224.94, Expect=1.60E-57, mRNA sequenc Length = 624; score = 42.3 bits (19), expect = 0.18; identities = 25/31 (80%); strand = Plus / Plus

```
Query: 16 gctwawwttgcttgattcacttcwwyaagag 46
          ||| |  |||||||||||||||   |||||
Sbjct: 1  gctaatattgcttgattcacttctacaagag 31
```

>EL585399|EL585399.1 CF_WO0_11a04_SP6 Copepod Whole Organism, Normalized *Calanus finmarchicus* cDNA clone CF_WO0_11a04; 5' similar to XP_966933.1 CG10189-PA [Tribolium castaneum]. Score=99.75, Expect=1.00E-19, mRNA sequence Length = 711; score = 42.3 bits (19), expect = 0.18; identities = 25/31 (80%); strand = Plus / Plus

Query: 16 gctwawwttgcttgattcacttcwwyaagag 46
          ||| | |||||||||||||||| |||||
Sbjct: 1 gctaatattgcttgattcacttctacaagag 31

>EH667179|EH667179.1 CF_WO0_10h04_SP6 Copepod Whole Organism, Normalized *Calanus finmarchicus* cDNA clone CF_WO0_10h04; 5' , mRNA sequence. Length = 743; score = 42.3 bits (19), expect = 0.18; identities = 25/31 (80%); strand = Plus / Plus

Query: 16 gctwawwttgcttgattcacttcwwyaagag 46
          ||| | |||||||||||||||| |||||
Sbjct: 3 gctaatattgcttgattcacttctacaagag 33

>EH667162|EH667162.1 CF_WO0_10f11_SP6 Copepod Whole Organism, Normalized *Calanus finmarchicus* cDNA clone CF_WO0_10f11; 5' , mRNA sequence. Length = 696; score = 42.3 bits (19), expect = 0.18; identities = 25/31 (80%); strand = Plus / Plus

Query: 16 gctwawwttgcttgattcacttcwwyaagag 46
          ||| | |||||||||||||||| |||||
Sbjct: 3 gctaatattgcttgattcacttctacaagag 33

>EH667147|EH667147.1 CF_WO0_10e08_SP6 Copepod Whole Organism, Normalized *Calanus finmarchicus* cDNA clone CF_WO0_10e08; 5' similar to ref|XP_001031569.1| hypothetical protein TTHERM_00773160 [Tetrahymena thermophila. Score Length = 707; score = 42.3 bits (19), expect = 0.18; identities = 25/31 (80%); strand = Plus / Plus

Query: 16 gctwawwttgcttgattcacttcwwyaagag 46
          ||| | |||||||||||||||| |||||
Sbjct: 3 gctaatattgcttgattcacttctacaagag 33

>EH667135|EH667135.1 CF_WO0_10d07_SP6 Copepod Whole Organism, Normalized *Calanus finmarchicus* cDNA clone CF_WO0_10d07; 5' , mRNA sequence. Length = 658; score = 42.3 bits (19), expect = 0.18; identities = 25/31 (80%); strand = Plus / Plus

Query: 16 gctwawwttgcttgattcacttcwwyaagag 46
          ||| | |||||||||||||||| |||||
Sbjct: 3 gctaatattgcttgattcacttctacaagag 33

>EH667087|EH667087.1 CF_WO0_09h06_SP6 Copepod Whole Organism, Normalized *Calanus finmarchicus* cDNA clone CF_WO0_09h06; 5' similar to ref|ZP_01627032.1| esterase - marine gamma proteobacterium HTCC2080. Score = 108 bits (27 Length = 617; score = 42.3 bits (19), expect = 0.18; identities = 25/31 (80%); strand = Plus / Plus

Query: 16 gctwawwttgcttgattcacttcwwyaagag 46

```
         ||| |  |||||||||||||||   |||||
Sbjct: 3  gctaatattgcttgattcacttctacaagag 33
```

>EH667041|EH667041.1 CF_W00_09d01_SP6 Copepod Whole Organism, Normalized *Calanus finmarchicus* cDNA clone CF_W00_09d01; 5' similar to gb|EAL32560.1| GA14216-PA - Drosophila pseudoobscura. Score = 285 bits (729), expect = 1e Length = 622; score = 42.3 bits (19), expect = 0.18; identities = 25/31 (80%); strand = Plus / Plus

```
Query: 16 gctwawwttgcttgattcacttcwwyaagag 46
          ||| |  |||||||||||||||   |||||
Sbjct: 1  gctaatattgcttgattcacttctacaagag 31
```

>EH667033|EH667033.1 CF_W00_09c03_SP6 Copepod Whole Organism, Normalized *Calanus finmarchicus* cDNA clone CF_W00_09c03; 5', mRNA sequence. Length = 668; score = 42.3 bits (19), expect = 0.18; identities = 25/31 (80%); strand = Plus / Plus

```
Query: 16 gctwawwttgcttgattcacttcwwyaagag 46
          ||| |  |||||||||||||||   |||||
Sbjct: 3  gctaatattgcttgattcacttctacaagag 33
```

>EH666832|EH666832.1 CF_W00_07b02_SP6 Copepod Whole Organism, Normalized *Calanus finmarchicus* cDNA clone CF_W00_07b02; 5' similar to ref|XP_313810.2| ENSANGP00000011692 - Anopheles gambiae str. PEST. Score = 240 bits (612) Length = 702; score = 42.3 bits (19), expect = 0.18; identities = 25/31 (80%); strand = Plus / Plus

```
Query: 16 gctwawwttgcttgattcacttcwwyaagag 46
          ||| |  |||||||||||||||   |||||
Sbjct: 3  gctaatattgcttgattcacttctacaagag 33
```

>EH666787|EH666787.1 CF_W00_06f04_SP6 Copepod Whole Organism, Normalized *Calanus finmarchicus* cDNA clone CF_W00_06f04; 5' similar to ref|XP_393706.3| DIVDICTED: similar to CG7650-PA isoform 1 - Apis mellifera. Score = 129 Length = 761; score = 42.3 bits (19), expect = 0.18; identities = 25/31 (80%); strand = Plus / Plus

```
Query: 16 gctwawwttgcttgattcacttcwwyaagag 46
          ||| |  |||||||||||||||   |||||
Sbjct: 3  gctaatattgcttgattcacttctacaagag 33
```

>EH666738|EH666738.1 CF_W00_06b01_SP6 Copepod Whole Organism, Normalized *Calanus finmarchicus* cDNA clone CF_W00_06b01; 5' similar to ref|XP_624581.1| DIVDICTED: similar to WD repeat domain, phosphoinositide. Score = 271 bi Length = 718; score = 42.3 bits (19), expect = 0.18; identities = 25/31 (80%); strand = Plus / Plus

```
Query: 16 gctwawwttgcttgattcacttcwwyaagag 46
          ||| |  |||||||||||||||   |||||
Sbjct: 3  gctaatattgcttgattcacttctacaagag 33
```

>EH666731|EH666731.1 CF_W00_06a06_SP6 Copepod Whole Organism, Normalized *Calanus finmarchicus* cDNA clone CF_W00_06a06; 5' similar to gb|EAT39488.1| conserved hypothetical protein - Aedes

aegypti. Score = 149 bits (375), Ex Length = 745; score = 42.3 bits (19), expect = 0.18; identities = 25/31 (80%); strand = Plus / Plus

```
Query: 16 gctwawwttgcttgattcacttcwwyaagag 46
          ||| |  |||||||||||||||   |||||
Sbjct: 1  gctaatattgcttgattcacttctacaagag 31
```

>EH666717|EH666717.1 CF_W00_05h04_SP6 Copepod Whole Organism, Normalized *Calanus finmarchicus* cDNA clone CF_W00_05h04; 5' similar to ref|XP_391645.1| hypothetical protein FG11469.1 - Gibberella zeae PH-1. Score = 213 bits Length = 595; score = 42.3 bits (19), expect = 0.18; identities = 25/31 (80%); strand = Plus / Plus

```
Query: 16 gctwawwttgcttgattcacttcwwyaagag 46
          ||| |  |||||||||||||||   |||||
Sbjct: 3  gctaatattgcttgattcacttctacaagag 33
```

>EH666705|EH666705.1 CF_W00_05g04_SP6 Copepod Whole Organism, Normalized *Calanus finmarchicus* cDNA clone CF_W00_05g04; 5' similar to ref|NP_001039853.1| heat shock 70kDa protein 14 - Bos taurus. Score = 175 bits (444), Exp Length = 710; score = 42.3 bits (19), expect = 0.18; identities = 25/31 (80%); strand = Plus / Plus

```
Query: 16 gctwawwttgcttgattcacttcwwyaagag 46
          ||| |  |||||||||||||||   |||||
Sbjct: 3  gctaatattgcttgattcacttctacaagag 33
```

>EH666570|EH666570.1 CF_W00_04c04_SP6 Copepod Whole Organism, Normalized *Calanus finmarchicus* cDNA clone CF_W00_04c04; 5' similar to ref|XP_974926.1| DIVDICTED: similar to CG2103-PA, isoform A - Tribolium castaneum. Score Length = 665; score = 42.3 bits (19), expect = 0.18; identities = 25/31 (80%); strand = Plus / Plus

```
Query: 16 gctwawwttgcttgattcacttcwwyaagag 46
          ||| |  |||||||||||||||   |||||
Sbjct: 1  gctaatattgcttgattcacttctacaagag 31
```

>EH666565|EH666565.1 CF_W00_04b11_SP6 Copepod Whole Organism, Normalized *Calanus finmarchicus* cDNA clone CF_W00_04b11; 5' similar to ref|XP_396005.3| DIVDICTED: similar to ring finger protein 34 isoform 2 isoform 1. Score Length = 670; score = 42.3 bits (19), expect = 0.18; identities = 25/31 (80%); strand = Plus / Plus

```
Query: 16 gctwawwttgcttgattcacttcwwyaagag 46
          ||| |  |||||||||||||||   |||||
Sbjct: 3  gctaatattgcttgattcacttctacaagag 33
```

>EH666546|EH666546.1 CF_W00_04a03_SP6 Copepod Whole Organism, Normalized *Calanus finmarchicus* cDNA clone CF_W00_04a03; 5' , mRNA sequence. Length = 669; score = 42.3 bits (19), expect = 0.18; identities = 25/31 (80%); strand = Plus / Plus

```
Query: 16 gctwawwttgcttgattcacttcwwyaagag 46
          ||| |  |||||||||||||||   |||||
```

```
Sbjct: 1  gctaatattgcttgattcacttctacaagag 31
```

>EH666368|EH666368.1 CF_WO0_02a10_SP6 Copepod Whole Organism, Normalized *Calanus finmarchicus* cDNA clone CF_WO0_02a10; 5' similar to ref|XP_316915.3| ENSANGP00000010956 - Anopheles gambiae str. PEST. Score = 116 bits (290) Length = 706; score = 42.3 bits (19), expect = 0.18; identities = 25/31 (80%); strand = Plus / Plus

```
Query: 16 gctwawwttgcttgattcacttcwwyaagag 46
          ||| |  ||||||||||||||||   |||||
Sbjct: 1  gctaatattgcttgattcacttctacaagag 31
```

>JZ751777|JZ751777.1 awig5P0008H18_F.ab1 Sea ice eukaryotic metatranscriptome Weddell Sea 1 uncultured organism cDNA, mRNA sequence. Length = 450; score = 40.1 bits (18), expect = 0.83; identities = 21/24 (87%); strand = Plus / Plus

```
Query: 23 ttgcttgattcacttcwwyaagag 46
          ||||||||||||||||   |||||
Sbjct: 4  ttgcttgattcacttctacaagag 27
```

>JZ533412|JZ533412.1 SSH_RHO2_F3_T7_ab1 Cal SSH_SKE_RHO 2d *Calanus helgolandicus* cDNA clone SSH_RHO2_F3_T7_ab1, mRNA sequence. Length = 354; score = 40.1 bits (18), expect = 0.83; identities = 21/24 (87%); strand = Plus / Plus

```
Query: 23 ttgcttgattcacttcwwyaagag 46
          ||||||||||||||||   |||||
Sbjct: 2  ttgcttgattcacttctttaagag 25
```

>JZ533383|JZ533383.1 SSH_RHO1_G3_T7_ab1 Cal SSH_SKE_RHO 2d *Calanus helgolandicus* cDNA clone SSH_RHO1_G3_T7_ab1 similar to ADP,ATP carrier protein 3, mRNA sequence. Length = 419; score = 40.1 bits (18), expect = 0.83; identities = 21/24 (87%); strand = Plus / Plus

```
Query: 23 ttgcttgattcacttcwwyaagag 46
          ||||||||||||||||   |||||
Sbjct: 4  ttgcttgattcacttctacaagag 27
```

>JZ533373|JZ533373.1 SSH_RHO1_F10_T7_ab1 Cal SSH_SKE_RHO 2d *Calanus helgolandicus* cDNA clone SSH_RHO1_F10_T7_ab1 similar to ADP,ATP carrier protein 3, mRNA sequence. Length = 417; score = 40.1 bits (18), expect = 0.83; identities = 21/24 (87%); strand = Plus / Plus

```
Query: 23 ttgcttgattcacttcwwyaagag 46
          ||||||||||||||||   |||||
Sbjct: 2  ttgcttgattcacttctacaagag 25
```

>JZ533350|JZ533350.1 SSH_RHO1_A6_T7_ab1 Cal SSH_SKE_RHO 2d *Calanus helgolandicus* cDNA clone SSH_RHO1_A6_T7_ab1 similar to ADP,ATP carrier protein 3, mRNA sequence. Length = 422; score = 40.1 bits (18), expect = 0.83; identities = 21/24 (87%); strand = Plus / Plus

```
Query: 23 ttgcttgattcacttcwwyaagag 46
          ||||||||||||||||   |||||
```

Sbjct: 7 ttgcttgattcacttctacaagag 30

>JZ533220|JZ533220.1 SSH_SKE7_F2_T7_ab1 Cal SSH_SKE_RHO 2d *Calanus helgolandicus* cDNA clone SSH_SKE7_F2_T7_ab1 similar to a chain human ck2 catalytic domain, mRNA sequence. Length = 205; score = 40.1 bits (18), expect = 0.83; identities = 21/24 (87%); strand = Plus / Minus

```
Query: 23  ttgcttgattcacttcwwyaagag 46
           ||||||||||||||||   |||||
Sbjct: 198 ttgcttgattcacttctacaagag 175
```

>JZ533160|JZ533160.1 SSH_SKE6_G9_T7_ab1 Cal SSH_SKE_RHO 2d *Calanus helgolandicus* cDNA clone SSH_SKE6_G9_T7_ab1 similar to a chain human ck2 catalytic domain, mRNA sequence. Length = 403; score = 40.1 bits (18), expect = 0.83; identities = 21/24 (87%); strand = Plus / Minus

```
Query: 23  ttgcttgattcacttcwwyaagag 46
           ||||||||||||||||   |||||
Sbjct: 396 ttgcttgattcacttctacaagag 373
```

>JZ533148|JZ533148.1 SSH_SKE6_F9_T7_ab1 Cal SSH_SKE_RHO 2d *Calanus helgolandicus* cDNA clone SSH_SKE6_F9_T7_ab1 similar to a chain human ck2 catalytic domain, mRNA sequence. Length = 403; score = 40.1 bits (18), expect = 0.83; identities = 21/24 (87%); strand = Plus / Minus

```
Query: 23  ttgcttgattcacttcwwyaagag 46
           ||||||||||||||||   |||||
Sbjct: 396 ttgcttgattcacttctacaagag 373
```

>JZ533122|JZ533122.1 SSH_SKE6_D12_T7_ab1 Cal SSH_SKE_RHO 2d *Calanus helgolandicus* cDNA clone SSH_SKE6_D12_T7_ab1-mRNA sequence. Length = 378; score = 40.1 bits (18), expect = 0.83; identities = 21/24 (87%); strand = Plus / Plus

```
Query: 23 ttgcttgattcacttcwwyaagag 46
          ||||||||||||||||   |||||
Sbjct: 3  ttgcttgattcacttctacaagag 26
```

>JZ533054|JZ533054.1 SSH_SKE5_E10_T7_ab1 Cal SSH_SKE_RHO 2d *Calanus helgolandicus* cDNA clone SSH_SKE5_E10_T7_ab1-mRNA sequence. Length = 390; score = 40.1 bits (18), expect = 0.83; identities = 21/24 (87%); strand = Plus / Plus

```
Query: 23 ttgcttgattcacttcwwyaagag 46
          ||||||||||||||||   |||||
Sbjct: 6  ttgcttgattcacttctacaagag 29
```

>JZ533052|JZ533052.1 SSH_SKE5_D7_T7_ab1 Cal SSH_SKE_RHO 2d *Calanus helgolandicus* cDNA clone SSH_SKE5_D7_T7_ab1 similar to a chain human ck2 catalytic domain, mRNA sequence. Length = 210; score = 40.1 bits (18), expect = 0.83; identities = 21/24 (87%); strand = Plus / Plus

```
Query: 23 ttgcttgattcacttcwwyaagag 46
          ||||||||||||||||   |||||
Sbjct: 8  ttgcttgattcacttctacaagag 31
```

>JZ533050|JZ533050.1 SSH_SKE5_D5_T7_ab1 Cal SSH_SKE_RHO 2d *Calanus helgolandicus* cDNA clone SSH_SKE5_D5_T7_ab1, mRNA sequence. Length = 362; score = 40.1 bits (18), expect = 0.83; identities = 21/24 (87%); strand = Plus / Minus

```
Query: 23  ttgcttgattcacttcwwyaagag 46
            ||||||||||||||||   |||||
Sbjct: 357 ttgcttgattcacttctacaagag 334
```

>JZ533028|JZ533028.1 SSH_SKE5_B2_T7_ab1 Cal SSH_SKE_RHO 2d *Calanus helgolandicus* cDNA clone SSH_SKE5_B2_T7_ab1, mRNA sequence. Length = 381; score = 40.1 bits (18), expect = 0.83; identities = 21/24 (87%); strand = Plus / Plus

```
Query: 23 ttgcttgattcacttcwwyaagag 46
          ||||||||||||||||   |||||
Sbjct: 6  ttgcttgattcacttctacaagag 29
```

>JZ533027|JZ533027.1 SSH_SKE5_B1_T7_ab1 Cal SSH_SKE_RHO 2d *Calanus helgolandicus* cDNA clone SSH_SKE5_B1_T7_ab1, mRNA sequence. Length = 381; score = 40.1 bits (18), expect = 0.83; identities = 21/24 (87%); strand = Plus / Plus

```
Query: 23 ttgcttgattcacttcwwyaagag 46
          ||||||||||||||||   |||||
Sbjct: 6  ttgcttgattcacttctacaagag 29
```

>JZ533005|JZ533005.1 SSH_SKE4_G6_T7_ab1 Cal SSH_SKE_RHO 2d *Calanus helgolandicus* cDNA clone SSH_SKE4_G6_T7_ab1 similar to a chain human ck2 catalytic domain, mRNA sequence. Length = 210; score = 40.1 bits (18), expect = 0.83; identities = 21/24 (87%); strand = Plus / Minus

```
Query: 23  ttgcttgattcacttcwwyaagag 46
            ||||||||||||||||   |||||
Sbjct: 203 ttgcttgattcacttctacaagag 180
```

>JZ533000|JZ533000.1 SSH_SKE4_G1_T7_ab1 Cal SSH_SKE_RHO 2d *Calanus helgolandicus* cDNA clone SSH_SKE4_G1_T7_ab1, mRNA sequence. Length = 376; score = 40.1 bits (18), expect = 0.83; identities = 21/24 (87%); strand = Plus / Plus

```
Query: 23 ttgcttgattcacttcwwyaagag 46
          ||||||||||||||||   |||||
Sbjct: 1  ttgcttgattcacttctacaagag 24
```

>JZ532936|JZ532936.1 SSH_SKE3_H8_T7_ab1 Cal SSH_SKE_RHO 2d *Calanus helgolandicus* cDNA clone SSH_SKE3_H8_T7_ab1 similar to a chain human ck2 catalytic domain, mRNA sequence. Length = 201; score = 40.1 bits (18), expect = 0.83; identities = 21/24 (87%); strand = Plus / Minus

```
Query: 23  ttgcttgattcacttcwwyaagag 46
            ||||||||||||||||   |||||
Sbjct: 194 ttgcttgattcacttctacaagag 171
```

>JZ532908|JZ532908.1 SSH_SKE3_E7_T7_ab1 Cal SSH_SKE_RHO 2d *Calanus helgolandicus* cDNA clone SSH_SKE3_E7_T7_ab1 similar to a chain human ck2 catalytic domain, mRNA sequence. Length = 201; score = 40.1 bits (18), expect = 0.83; identities = 21/24 (87%); strand = Plus / Minus

```
Query: 23  ttgcttgattcacttcwwyaagag 46
           ||||||||||||||||   |||||
Sbjct: 194 ttgcttgattcacttctacaagag 171
```

>JZ532838|JZ532838.1 SSH_SKE2_F2_T7_ab1 Cal SSH_SKE_RHO 2d *Calanus helgolandicus* cDNA clone SSH_SKE2_F2_T7_ab1 similar to a chain human ck2 catalytic domain, mRNA sequence. Length = 210; score = 40.1 bits (18), expect = 0.83; identities = 21/24 (87%); strand = Plus / Plus

```
Query: 23 ttgcttgattcacttcwwyaagag 46
          ||||||||||||||||   |||||
Sbjct: 8  ttgcttgattcacttctacaagag 31
```

>JZ532823|JZ532823.1 SSH_SKE2_D7_T7_ab1 Cal SSH_SKE_RHO 2d *Calanus helgolandicus* cDNA clone SSH_SKE2_D7_T7_ab1 similar to a chain human ck2 catalytic domain, mRNA sequence. Length = 390; score = 40.1 bits (18), expect = 0.83; identities = 21/24 (87%); strand = Plus / Plus

```
Query: 23 ttgcttgattcacttcwwyaagag 46
          ||||||||||||||||   |||||
Sbjct: 8  ttgcttgattcacttctacaagag 31
```

>JZ532801|JZ532801.1 SSH_SKE2_B7_T7_ab1 Cal SSH_SKE_RHO 2d *Calanus helgolandicus* cDNA clone SSH_SKE2_B7_T7_ab1 similar to a chain human ck2 catalytic domain, mRNA sequence. Length = 202; score = 40.1 bits (18), expect = 0.83; identities = 21/24 (87%); strand = Plus / Minus

```
Query: 23  ttgcttgattcacttcwwyaagag 46
           ||||||||||||||||   |||||
Sbjct: 195 ttgcttgattcacttctacaagag 172
```

>JZ532794|JZ532794.1 SSH_SKE2_A7_T7_ab1 Cal SSH_SKE_RHO 2d *Calanus helgolandicus* cDNA clone SSH_SKE2_A7_T7_ab1 similar to a chain human ck2 catalytic domain, mRNA sequence. Length = 210; score = 40.1 bits (18), expect = 0.83; identities = 21/24 (87%); strand = Plus / Plus

```
Query: 23 ttgcttgattcacttcwwyaagag 46
          ||||||||||||||||   |||||
Sbjct: 8  ttgcttgattcacttctacaagag 31
```

>JZ532763|JZ532763.1 SSH_SKE1_F8_T7_ab1 Cal SSH_SKE_RHO 2d *Calanus helgolandicus* cDNA clone SSH_SKE1_F8_T7_ab1 similar to a chain human ck2 catalytic domain, mRNA sequence. Length = 210; score = 40.1 bits (18), expect = 0.83; identities = 21/24 (87%); strand = Plus / Plus

```
Query: 23 ttgcttgattcacttcwwyaagag 46
          ||||||||||||||||   |||||
Sbjct: 8  ttgcttgattcacttctacaagag 31
```

>JZ532686|JZ532686.1 SSH_SKE10_D9_T7_ab1 Cal SSH_SKE_RHO 2d *Calanus helgolandicus* cDNA clone SSH_SKE10_D9_T7_ab1 similar to a chain human ck2 catalytic domain, mRNA sequence. Length = 563; score = 40.1 bits (18), expect = 0.83; identities = 21/24 (87%); strand = Plus / Plus

```
Query: 23 ttgcttgattcacttcwwyaagag 46
          ||||||||||||||||   |||||
Sbjct: 8  ttgcttgattcacttctacaagag 31
```

>BW813862|BW813862.1 Branchiostoma floridae cDNA, larva clone:bflv057d24, 5' end. Length = 388; score = 40.1 bits (18), expect = 0.83; identities = 21/24 (87%); strand = Plus / Plus

```
Query: 23 ttgcttgattcacttcwwyaagag 46
          ||||||||||||||||   |||||
Sbjct: 6  ttgcttgattcacttctacaagag 29
```

>BW809910|BW809910.1 Branchiostoma floridae cDNA, larva clone:bflv045p11, 5' end. Length = 343; score = 40.1 bits (18), expect = 0.83; identities = 21/24 (87%); strand = Plus / Plus

```
Query: 23 ttgcttgattcacttcwwyaagag 46
          ||||||||||||||||   |||||
Sbjct: 7  ttgcttgattcacttctacaagag 30
```

>BW804361|BW804361.1 Branchiostoma floridae cDNA, larva clone:bflv030d22, 5' end. Length = 366; score = 40.1 bits (18), expect = 0.83; identities = 21/24 (87%); strand = Plus / Plus

```
Query: 23 ttgcttgattcacttcwwyaagag 46
          ||||||||||||||||   |||||
Sbjct: 6  ttgcttgattcacttctacaagag 29
```

>BW802526|BW802526.1 Branchiostoma floridae cDNA, larva clone:bflv024l18, 5' end. Length = 498; score = 40.1 bits (18), expect = 0.83; identities = 21/24 (87%); strand = Plus / Plus

```
Query: 23 ttgcttgattcacttcwwyaagag 46
          ||||||||||||||||   |||||
Sbjct: 7  ttgcttgattcacttctacaagag 30
```

>BW792514|BW792514.1 Branchiostoma floridae cDNA, gastrula clone:bfga054j09, 3' end. Length = 426; score = 40.1 bits (18), expect = 0.83; identities = 21/24 (87%); strand = Plus / Minus

```
Query: 23  ttgcttgattcacttcwwyaagag 46
           ||||||||||||||||   |||||
Sbjct: 421 ttgcttgattcacttctacaagag 398
```

>BW785609|BW785609.1 Branchiostoma floridae cDNA, gastrula clone:bfga021o05, 3' end. Length = 505; score = 40.1 bits (18), expect = 0.83; identities = 21/24 (87%); strand = Plus / Minus

```
Query: 23  ttgcttgattcacttcwwyaagag 46
           ||||||||||||||||   |||||
Sbjct: 499 ttgcttgattcacttctacaagag 476
```

>BW779325|BW779325.1 Branchiostoma floridae cDNA, gastrula clone:bfga054j09, 5' end. Length = 465; score = 40.1 bits (18), expect = 0.83; identities = 21/24 (87%); strand = Plus / Plus

```
Query: 23 ttgcttgattcacttcwwyaagag 46
          ||||||||||||||||   |||||
Sbjct: 6  ttgcttgattcacttctacaagag 29
```

>BW778946|BW778946.1 Branchiostoma floridae cDNA, gastrula clone:bfga053b23, 5' end. Length = 491; score = 40.1 bits (18), expect = 0.83; identities = 21/24 (87%); strand = Plus / Plus

```
Query: 23 ttgcttgattcacttcwwyaagag 46
          ||||||||||||||||   |||||
Sbjct: 6  ttgcttgattcacttctacaagag 29
```

>BW773653|BW773653.1 Branchiostoma floridae cDNA, gastrula clone:bfga007k20, 5' end. Length = 693; score = 40.1 bits (18), expect = 0.83; identities = 21/24 (87%); strand = Plus / Plus

```
Query: 23 ttgcttgattcacttcwwyaagag 46
          ||||||||||||||||   |||||
Sbjct: 6  ttgcttgattcacttctacaagag 29
```

>BW772893|BW772893.1 Branchiostoma floridae cDNA, gastrula clone:bfga004j21, 5' end. Length = 601; score = 40.1 bits (18), expect = 0.83; identities = 21/24 (87%); strand = Plus / Plus

```
Query: 23 ttgcttgattcacttcwwyaagag 46
          ||||||||||||||||   |||||
Sbjct: 6  ttgcttgattcacttctacaagag 29
```

>BW772235|BW772235.1 Branchiostoma floridae cDNA, gastrula clone:bfga021o05, 5' end. Length = 554; score = 40.1 bits (18), expect = 0.83; identities = 21/24 (87%); strand = Plus / Plus

```
Query: 23 ttgcttgattcacttcwwyaagag 46
          ||||||||||||||||   |||||
Sbjct: 7  ttgcttgattcacttctacaagag 30
```

>GR411285|GR411285.1 CF_WO1_59h10_SP6 Copepod Whole Organism, Normalized *Calanus finmarchicus* cDNA clone CF_WO1_59h10; 5' similar to ref|XP_625117.1| DIVDICTED: similar to farnesyltransferase, CAAX box, beta [Apis. Score = Length = 897; score = 40.1 bits (18), expect = 0.83; identities = 21/24 (87%); strand = Plus / Plus

```
Query: 23 ttgcttgattcacttcwwyaagag 46
          ||||||||||||||||   |||||
Sbjct: 11 ttgcttgattcacttctacaagag 34
```

>GR411276|GR411276.1 CF_WO1_59g09_SP6 Copepod Whole Organism, Normalized *Calanus finmarchicus* cDNA clone CF_WO1_59g09; 5', mRNA sequence. Length = 862; score = 40.1 bits (18), expect = 0.83; identities = 21/24 (87%); strand = Plus / Plus

Query: 23 ttgcttgattcacttcwwyaagag 46
         ||||||||||||||| |||||
Sbjct: 6 ttgcttgattcacttctacaagag 29

>GR411258|GR411258.1 CF_W01_59e11_SP6 Copepod Whole Organism, Normalized *Calanus finmarchicus* cDNA clone CF_W01_59e11; 5’ similar to ref|XP_002047783.1| GJ11733 - Drosophila virilis. Score = 161 bits (407), expect = 9e-38, Length = 871; score = 40.1 bits (18), expect = 0.83; identities = 21/24 (87%); strand = Plus / Plus

Query: 23 ttgcttgattcacttcwwyaagag 46
         ||||||||||||||| |||||
Sbjct: 13 ttgcttgattcacttctacaagag 36

>GR411251|GR411251.1 CF_W01_59d12_SP6 Copepod Whole Organism, Normalized *Calanus finmarchicus* cDNA clone CF_W01_59d12; 5’ , mRNA sequence. Length = 835; score = 40.1 bits (18), expect = 0.83; identities = 21/24 (87%); strand = Plus / Plus

Query: 23 ttgcttgattcacttcwwyaagag 46
         ||||||||||||||| |||||
Sbjct: 6 ttgcttgattcacttctacaagag 29

>GR411235|GR411235.1 CF_W01_59c06_SP6 Copepod Whole Organism, Normalized *Calanus finmarchicus* cDNA clone CF_W01_59c06; 5’ , mRNA sequence. Length = 846; score = 40.1 bits (18), expect = 0.83; identities = 21/24 (87%); strand = Plus / Plus

Query: 23 ttgcttgattcacttcwwyaagag 46
         ||||||||||||||| |||||
Sbjct: 8 ttgcttgattcacttctacaagag 31

>GR411212|GR411212.1 CF_W01_58h10_SP6 Copepod Whole Organism, Normalized *Calanus finmarchicus* cDNA clone CF_W01_58h10; 5’ similar to ref|XP_316648.3| AGAP006617-PA - Anopheles gambiae str. PEST. Score = 64.3 bits (155), Ex Length = 803; score = 40.1 bits (18), expect = 0.83; identities = 21/24 (87%); strand = Plus / Plus

Query: 23 ttgcttgattcacttcwwyaagag 46
         ||||||||||||||| |||||
Sbjct: 7 ttgcttgattcacttctacaagag 30

>GR411114|GR411114.1 CF_W01_57f12_SP6 Copepod Whole Organism, Normalized *Calanus finmarchicus* cDNA clone CF_W01_57f12; 5’ , mRNA sequence. Length = 708; score = 40.1 bits (18), expect = 0.83; identities = 21/24 (87%); strand = Plus / Plus

Query: 23 ttgcttgattcacttcwwyaagag 46
         ||||||||||||||| |||||
Sbjct: 12 ttgcttgattcacttctacaagag 35

>GR411108|GR411108.1 CF_W01_57f06_SP6 Copepod Whole Organism, Normalized *Calanus finmarchicus* cDNA clone CF_W01_57f06; 5’ similar to ref|NP_001037171.1| protein disulfide isomerase -

Bombyx mori. Score = 138 bits (347), Ex Length = 384; score = 40.1 bits (18), expect = 0.83; identities = 21/24 (87%); strand = Plus / Plus

```
Query: 23 ttgcttgattcacttcwwyaagag 46
          |||||||||||||||   |||||
Sbjct: 7  ttgcttgattcacttctacaagag 30
```

>GR411093|GR411093.1 CF_W01_57d07_SP6 Copepod Whole Organism, Normalized *Calanus finmarchicus* cDNA clone CF_W01_57d07; 5' similar to gb|EEB13626.1| Coatomer subunit delta, putative - Pediculus humanus corporis. Score = 298 Length = 646; score = 40.1 bits (18), expect = 0.83; identities = 21/24 (87%); strand = Plus / Plus

```
Query: 23 ttgcttgattcacttcwwyaagag 46
          |||||||||||||||   |||||
Sbjct: 12 ttgcttgattcacttctacaagag 35
```

>GR411025|GR411025.1 CF_W01_56c06_SP6 Copepod Whole Organism, Normalized *Calanus finmarchicus* cDNA clone CF_W01_56c06; 5' similar to gb|ACJ69403.1| Vasa - Botryllus schlosseri. Score = 60.5 bits (145), expect = 1e-07-mRNA Length = 686; score = 40.1 bits (18), expect = 0.83; identities = 21/24 (87%); strand = Plus / Plus

```
Query: 23 ttgcttgattcacttcwwyaagag 46
          |||||||||||||||   |||||
Sbjct: 7  ttgcttgattcacttctacaagag 30
```

>GR411017|GR411017.1 CF_W01_56b03_SP6 Copepod Whole Organism, Normalized *Calanus finmarchicus* cDNA clone CF_W01_56b03; 5', mRNA sequence. Length = 762; score = 40.1 bits (18), expect = 0.83; identities = 21/24 (87%); strand = Plus / Plus

```
Query: 23 ttgcttgattcacttcwwyaagag 46
          |||||||||||||||   |||||
Sbjct: 7  ttgcttgattcacttctacaagag 30
```

>GR410993|GR410993.1 CF_W01_55g06_SP6 Copepod Whole Organism, Normalized *Calanus finmarchicus* cDNA clone CF_W01_55g06; 5', mRNA sequence. Length = 626; score = 40.1 bits (18), expect = 0.83; identities = 21/24 (87%); strand = Plus / Plus

```
Query: 23 ttgcttgattcacttcwwyaagag 46
          |||||||||||||||   |||||
Sbjct: 13 ttgcttgattcacttctacaagag 36
```

>GR410963|GR410963.1 CF_W01_55d05_SP6 Copepod Whole Organism, Normalized *Calanus finmarchicus* cDNA clone CF_W01_55d05; 5' similar to gb|EEB14172.1| F-box/LRR-repeat protein, putative - Pediculus humanus corporis. Score = 5 Length = 880; score = 40.1 bits (18), expect = 0.83; identities = 21/24 (87%); strand = Plus / Plus

```
Query: 23 ttgcttgattcacttcwwyaagag 46
          |||||||||||||||   |||||
Sbjct: 4  ttgcttgattcacttctacaagag 27
```

>GR410954|GR410954.1 CF_WO1_55c06_SP6 Copepod Whole Organism, Normalized *Calanus finmarchicus* cDNA clone CF_WO1_55c06; 5’ similar to ref|XP_001952084.1| DIVDICTED: similar to CG3209 CG3209-PB - Acyrthosiphon pisum. Score = Length = 867; score = 40.1 bits (18), expect = 0.83; identities = 21/24 (87%); strand = Plus / Plus

```
Query: 23 ttgcttgattcacttcwwyaagag 46
          |||||||||||||||   |||||
Sbjct: 5  ttgcttgattcacttctacaagag 28
```

>GR410953|GR410953.1 CF_WO1_55c05_SP6 Copepod Whole Organism, Normalized *Calanus finmarchicus* cDNA clone CF_WO1_55c05; 5’ similar to ref|XP_002199684.1| DIVDICTED: G2/M-phase specific E3 ubiquitin ligase [Taeniopygia. Scor Length = 615; score = 40.1 bits (18), expect = 0.83; identities = 21/24 (87%); strand = Plus / Plus

```
Query: 23 ttgcttgattcacttcwwyaagag 46
          |||||||||||||||   |||||
Sbjct: 8  ttgcttgattcacttcttcaagag 31
```

>GR410912|GR410912.1 CF_WO1_54f06_SP6 Copepod Whole Organism, Normalized *Calanus finmarchicus* cDNA clone CF_WO1_54f06; 5’ similar to gb|AAM09083.1| Na+/Cl- dependent neurotransmitter transporter-like protein. Score = 139 bits, Length = 586; score = 40.1 bits (18), expect = 0.83; identities = 21/24 (87%); strand = Plus / Plus

```
Query: 23 ttgcttgattcacttcwwyaagag 46
          |||||||||||||||   |||||
Sbjct: 9  ttgcttgattcacttctacaagag 32
```

>GR410910|GR410910.1 CF_WO1_54f04_SP6 Copepod Whole Organism, Normalized *Calanus finmarchicus* cDNA clone CF_WO1_54f04; 5’ similar to ref|XP_001661907.1| wd-repeat protein - Aedes aegypti. Score = 280 bits (717), expect = 4 Length = 570; score = 40.1 bits (18), expect = 0.83; identities = 21/24 (87%); strand = Plus / Plus

```
Query: 23 ttgcttgattcacttcwwyaagag 46
          |||||||||||||||   |||||
Sbjct: 19 ttgcttgattcacttctacaagag 42
```

>GR410907|GR410907.1 CF_WO1_54e11_SP6 Copepod Whole Organism, Normalized *Calanus finmarchicus* cDNA clone CF_WO1_54e11; 5’, mRNA sequence. Length = 721; score = 40.1 bits (18), expect = 0.83; identities = 21/24 (87%); strand = Plus / Plus

```
Query: 23 ttgcttgattcacttcwwyaagag 46
          |||||||||||||||   |||||
Sbjct: 15 ttgcttgattcacttctacaagag 38
```

>GR410895|GR410895.1 CF_WO1_54d06_SP6 Copepod Whole Organism, Normalized *Calanus finmarchicus* cDNA clone CF_WO1_54d06; 5’, mRNA sequence. Length = 178; score = 40.1 bits (18), expect = 0.83; identities = 21/24 (87%); strand = Plus / Plus

Query: 23 ttgcttgattcacttcwwyaagag 46
         |||||||||||||||| |||||
Sbjct: 18 ttgcttgattcacttctacaagag 41

>GR410869|GR410869.1 CF_W01_54a06_SP6 Copepod Whole Organism, Normalized *Calanus finmarchicus* cDNA clone CF_W01_54a06; 5' similar to ref|NP_001007892.1| MGC89988 protein - Xenopus (Silurana) tropicalis. Score = 93.6 bits (Length = 766; score = 40.1 bits (18), expect = 0.83; identities = 21/24 (87%); strand = Plus / Plus

Query: 23 ttgcttgattcacttcwwyaagag 46
         |||||||||||||||| |||||
Sbjct: 11 ttgcttgattcacttctacaagag 34

>GR410861|GR410861.1 CF_W01_53h07_SP6 Copepod Whole Organism, Normalized *Calanus finmarchicus* cDNA clone CF_W01_53h07; 5' similar to dbj|BAH56610.1| arginine kinase 3 - Neocaridina denticulata. Score = 419 bits (1077), Exp Length = 838; score = 40.1 bits (18), expect = 0.83; identities = 21/24 (87%); strand = Plus / Plus

Query: 23 ttgcttgattcacttcwwyaagag 46
         |||||||||||||||| |||||
Sbjct: 4 ttgcttgattcacttctacaagag 27

>GR410847|GR410847.1 CF_W01_53f11_SP6 Copepod Whole Organism, Normalized *Calanus finmarchicus* cDNA clone CF_W01_53f11; 5' similar to gb|AAI42562.1| LOC100101293 protein - Xenopus laevis. Score = 114 bits (285), expect = 9e Length = 750; score = 40.1 bits (18), expect = 0.83; identities = 21/24 (87%); strand = Plus / Plus

Query: 23 ttgcttgattcacttcwwyaagag 46
         |||||||||||||||| |||||
Sbjct: 9 ttgcttgattcacttctacaagag 32

>GR410801|GR410801.1 CF_W01_53a10_SP6 Copepod Whole Organism, Normalized *Calanus finmarchicus* cDNA clone CF_W01_53a10; 5' similar to gb|ACO12384.1| Nucleoside diphosphate kinase A 1 - Lepeophtheirus salmonis. Score = 171 b Length = 680; score = 40.1 bits (18), expect = 0.83; identities = 21/24 (87%); strand = Plus / Plus

Query: 23 ttgcttgattcacttcwwyaagag 46
         |||||||||||||||| |||||
Sbjct: 3 ttgcttgattcacttctacaagag 26

>GR410796|GR410796.1 CF_W01_53a05_SP6 Copepod Whole Organism, Normalized *Calanus finmarchicus* cDNA clone CF_W01_53a05; 5' similar to emb|CAG11740.1| unnamed protein product - Tetraodon nigroviridis. Score = 228 bits (582), Length = 873; score = 40.1 bits (18), expect = 0.83; identities = 21/24 (87%); strand = Plus / Plus

Query: 23 ttgcttgattcacttcwwyaagag 46
         |||||||||||||||| |||||
Sbjct: 8 ttgcttgattcacttctacaagag 31

>FQ001353|FQ001353.1 Pleurobrachia pileus 5-PRIME EST from clone SQ0AAA96YA03 (SQ0AAA96YA03RM1). Length = 794; score = 40.1 bits (18), expect = 0.83; identities = 21/24 (87%); strand = Plus / Plus

```
Query: 23 ttgcttgattcacttcwwyaagag 46
          ||||||||||||||||   |||||
Sbjct: 7  ttgcttgattcacttctacaagag 30
```

>FK868334|FK868334.1 CF_W01_20D01_SP6 Copepod Whole Organism, Normalized *Calanus finmarchicus* cDNA clone CF_W01_20D01; 5’ similar to gb|AAK50057.2|AF364404_1 Rh-like protein - Carcinus maenas. Score = 160 bits (404), Expec Length = 692; score = 40.1 bits (18), expect = 0.83; identities = 21/24 (87%); strand = Plus / Plus

```
Query: 23 ttgcttgattcacttcwwyaagag 46
          ||||||||||||||||   |||||
Sbjct: 15 ttgcttgattcacttctacaagag 38
```

>FK868314|FK868314.1 CF_W01_20B04_SP6 Copepod Whole Organism, Normalized *Calanus finmarchicus* cDNA clone CF_W01_20B04; 5’ , mRNA sequence. Length = 596; score = 40.1 bits (18), expect = 0.83; identities = 21/24 (87%); strand = Plus / Plus

```
Query: 23 ttgcttgattcacttcwwyaagag 46
          ||||||||||||||||   |||||
Sbjct: 12 ttgcttgattcacttctacaagag 35
```

>FK868313|FK868313.1 CF_W01_20B03_SP6 Copepod Whole Organism, Normalized *Calanus finmarchicus* cDNA clone CF_W01_20B03; 5’ similar to ref|XP_974670.1| DIVDICTED: similar to ribose-phosphate pyrophosphokinase 1,2. Score = 24 Length = 633; score = 40.1 bits (18), expect = 0.83; identities = 21/24 (87%); strand = Plus / Plus

```
Query: 23 ttgcttgattcacttcwwyaagag 46
          ||||||||||||||||   |||||
Sbjct: 10 ttgcttgattcacttctacaagag 33
```

>FK868296|FK868296.1 CF_W01_19H09_SP6 Copepod Whole Organism, Normalized *Calanus finmarchicus* cDNA clone CF_W01_19H09; 5’ , mRNA sequence. Length = 436; score = 40.1 bits (18), expect = 0.83; identities = 21/24 (87%); strand = Plus / Plus

```
Query: 23 ttgcttgattcacttcwwyaagag 46
          ||||||||||||||||   |||||
Sbjct: 6  ttgcttgattcacttctacaagag 29
```

>FK868270|FK868270.1 CF_W01_19F02_SP6 Copepod Whole Organism, Normalized *Calanus finmarchicus* cDNA clone CF_W01_19F02; 5’ , mRNA sequence. Length = 577; score = 40.1 bits (18), expect = 0.83; identities = 21/24 (87%); strand = Plus / Plus

```
Query: 23 ttgcttgattcacttcwwyaagag 46
          ||||||||||||||||   |||||
Sbjct: 6  ttgcttgattcacttctacaagag 29
```

>FK868246|FK868246.1 CF_W01_19C02_SP6 Copepod Whole Organism, Normalized *Calanus finmarchicus* cDNA clone CF_W01_19C02; 5' similar to gb|EDW84673.1| GK14239 - Drosophila willistoni. Score = 177 bits (448), expect = 9e-43, m Length = 740; score = 40.1 bits (18), expect = 0.83; identities = 21/24 (87%); strand = Plus / Plus

```
Query: 23 ttgcttgattcacttcwwyaagag 46
          ||||||||||||||||   |||||
Sbjct: 12 ttgcttgattcacttctacaagag 35
```

>FK868239|FK868239.1 CF_W01_19B02_SP6 Copepod Whole Organism, Normalized *Calanus finmarchicus* cDNA clone CF_W01_19B02; 5' similar to gb|EDV21370.1| hypothetical protein TRIADDRAFT_60323 - Trichoplax adhaerens. Score = 85.5 Length = 669; score = 40.1 bits (18), expect = 0.83; identities = 21/24 (87%); strand = Plus / Plus

```
Query: 23 ttgcttgattcacttcwwyaagag 46
          ||||||||||||||||   |||||
Sbjct: 8  ttgcttgattcacttctacaagag 31
```

>FK868237|FK868237.1 CF_W01_19A11_SP6 Copepod Whole Organism, Normalized *Calanus finmarchicus* cDNA clone CF_W01_19A11; 5', mRNA sequence. Length = 640; score = 40.1 bits (18), expect = 0.83; identities = 21/24 (87%); strand = Plus / Plus

```
Query: 23 ttgcttgattcacttcwwyaagag 46
          ||||||||||||||||   |||||
Sbjct: 8  ttgcttgattcacttctacaagag 31
```

>FK868203|FK868203.1 CF_W01_18F08_SP6 Copepod Whole Organism, Normalized *Calanus finmarchicus* cDNA clone CF_W01_18F08; 5', mRNA sequence. Length = 577; score = 40.1 bits (18), expect = 0.83; identities = 21/24 (87%); strand = Plus / Plus

```
Query: 23 ttgcttgattcacttcwwyaagag 46
          ||||||||||||||||   |||||
Sbjct: 4  ttgcttgattcacttctacaagag 27
```

>FK868196|FK868196.1 CF_W01_18F01_SP6 Copepod Whole Organism, Normalized *Calanus finmarchicus* cDNA clone CF_W01_18F01; 5' similar to ref|XP_971137.1| DIVDICTED: similar to eukaryotic translation initiation factor 3. Score Length = 598; score = 40.1 bits (18), expect = 0.83; identities = 21/24 (87%); strand = Plus / Plus

```
Query: 23 ttgcttgattcacttcwwyaagag 46
          ||||||||||||||||   |||||
Sbjct: 9  ttgcttgattcacttctacaagag 32
```

>FK868166|FK868166.1 CF_W01_18C06_SP6 Copepod Whole Organism, Normalized *Calanus finmarchicus* cDNA clone CF_W01_18C06; 5', mRNA sequence. Length = 565; score = 40.1 bits (18), expect = 0.83; identities = 21/24 (87%); strand = Plus / Plus

```
Query: 23 ttgcttgattcacttcwwyaagag 46
```

```
         |||||||||||||||   |||||
Sbjct: 9  ttgcttgattcacttctacaagag 32
```

>FK868125|FK868125.1 CF_W01_17A10_SP6 Copepod Whole Organism, Normalized *Calanus finmarchicus* cDNA clone CF_W01_17A10; 5' similar to ref|XP_001861875.1| 26S protease regulatory subunit 4 - Culex pipiens quinquefasciatus. S Length = 564; score = 40.1 bits (18), expect = 0.83; identities = 21/24 (87%); strand = Plus / Plus

```
Query: 23 ttgcttgattcacttcwwyaagag 46
          |||||||||||||||   |||||
Sbjct: 13 ttgcttgattcacttctacaagag 36
```

>FK868122|FK868122.1 CF_W01_17A07_SP6 Copepod Whole Organism, Normalized *Calanus finmarchicus* cDNA clone CF_W01_17A07; 5' similar to dbj|BAD46736.1| broad-complex A-NZ2 isoform - Bombyx mori. Score = 152 bits (385), expect Length = 550; score = 40.1 bits (18), expect = 0.83; identities = 21/24 (87%); strand = Plus / Plus

```
Query: 23 ttgcttgattcacttcwwyaagag 46
          |||||||||||||||   |||||
Sbjct: 2  ttgcttgattcacttctacaagag 25
```

>FK868116|FK868116.1 CF_W01_16H12_SP6 Copepod Whole Organism, Normalized *Calanus finmarchicus* cDNA clone CF_W01_16H12; 5' similar to gb|EDV49959.1| GG23811 - Drosophila erecta. Score = 105 bits (262), expect = 3e-21, mRNA Length = 681; score = 40.1 bits (18), expect = 0.83; identities = 21/24 (87%); strand = Plus / Plus

```
Query: 23 ttgcttgattcacttcwwyaagag 46
          |||||||||||||||   |||||
Sbjct: 7  ttgcttgattcacttctacaagag 30
```

>FK868111|FK868111.1 CF_W01_16H07_SP6 Copepod Whole Organism, Normalized *Calanus finmarchicus* cDNA clone CF_W01_16H07; 5' similar to ref|XP_001605215.1| DIVDICTED: similar to smad4 - Nasonia vitripennis. Score = 278 bits (Length = 666; score = 40.1 bits (18), expect = 0.83; identities = 21/24 (87%); strand = Plus / Plus

```
Query: 23 ttgcttgattcacttcwwyaagag 46
          |||||||||||||||   |||||
Sbjct: 9  ttgcttgattcacttctacaagag 32
```

>FK868095|FK868095.1 CF_W01_16G03_SP6 Copepod Whole Organism, Normalized *Calanus finmarchicus* cDNA clone CF_W01_16G03; 5' similar to ref|XP_976099.2| DIVDICTED: similar to ethanolaminephosphotransferase isoform 3. Score = Length = 644; score = 40.1 bits (18), expect = 0.83; identities = 21/24 (87%); strand = Plus / Plus

```
Query: 23 ttgcttgattcacttcwwyaagag 46
          |||||||||||||||   |||||
Sbjct: 9  ttgcttgattcacttctacaagag 32
```

>FK868084|FK868084.1 CF_WO1_16F04_SP6 Copepod Whole Organism, Normalized *Calanus finmarchicus* cDNA clone CF_WO1_16F04; 5' similar to ref|XP_001864663.1| alanine aminotransferase - Culex pipiens quinquefasciatus. Score = 26 Length = 692; score = 40.1 bits (18), expect = 0.83; identities = 21/24 (87%); strand = Plus / Plus

```
Query: 23 ttgcttgattcacttcwwyaagag 46
          ||||||||||||||||   |||||
Sbjct: 10 ttgcttgattcacttctacaagag 33
```

>FK868062|FK868062.1 CF_WO1_16D05_SP6 Copepod Whole Organism, Normalized *Calanus finmarchicus* cDNA clone CF_WO1_16D05; 5' similar to ref|XP_393677.2| DIVDICTED: similar to Downstream of son gene protein homolog [Apis. Scor Length = 702; score = 40.1 bits (18), expect = 0.83; identities = 21/24 (87%); strand = Plus / Plus

```
Query: 23 ttgcttgattcacttcwwyaagag 46
          ||||||||||||||||   |||||
Sbjct: 9  ttgcttgattcacttctacaagag 32
```

>FK867989|FK867989.1 CF_WO1_15D04_SP6 Copepod Whole Organism, Normalized *Calanus finmarchicus* cDNA clone CF_WO1_15D04; 5' similar to ref|XP_972584.1| DIVDICTED: similar to spinster CG8428-PC - Tribolium castaneum. Score = Length = 588; score = 40.1 bits (18), expect = 0.83; identities = 21/24 (87%); strand = Plus / Plus

```
Query: 23 ttgcttgattcacttcwwyaagag 46
          ||||||||||||||||   |||||
Sbjct: 13 ttgcttgattcacttctacaagag 36
```

>FK867977|FK867977.1 CF_WO1_15C03_SP6 Copepod Whole Organism, Normalized *Calanus finmarchicus* cDNA clone CF_WO1_15C03; 5', mRNA sequence. Length = 585; score = 40.1 bits (18), expect = 0.83; identities = 21/24 (87%); strand = Plus / Plus

```
Query: 23 ttgcttgattcacttcwwyaagag 46
          ||||||||||||||||   |||||
Sbjct: 11 ttgcttgattcacttctacaagag 34
```

>FK867967|FK867967.1 CF_WO1_15B04_SP6 Copepod Whole Organism, Normalized *Calanus finmarchicus* cDNA clone CF_WO1_15B04; 5', mRNA sequence. Length = 551; score = 40.1 bits (18), expect = 0.83; identities = 21/24 (87%); strand = Plus / Plus

```
Query: 23 ttgcttgattcacttcwwyaagag 46
          ||||||||||||||||   |||||
Sbjct: 15 ttgcttgattcacttctacaagag 38
```

>FK867918|FK867918.1 CF_WO1_14D03_SP6 Copepod Whole Organism, Normalized *Calanus finmarchicus* cDNA clone CF_WO1_14D03; 5' similar to ref|XP_001603688.1| DIVDICTED: similar to beta-1,4-galactosyltransferase [Nasonia. Score Length = 661; score = 40.1 bits (18), expect = 0.83; identities = 21/24 (87%); strand = Plus / Plus

```
Query: 23 ttgcttgattcacttcwwyaagag 46
```

```
         |||||||||||||||   |||||
Sbjct: 11 ttgcttgattcacttctacaagag 34
```

>FK867916|FK867916.1 CF_W01_14D01_SP6 Copepod Whole Organism, Normalized *Calanus finmarchicus* cDNA clone CF_W01_14D01; 5' similar to ref|NP_001098066.2| aubergine protein - Bombyx mori. Score = 153 bits (387), expect = 7e- Length = 628; score = 40.1 bits (18), expect = 0.83; identities = 21/24 (87%); strand = Plus / Plus

```
Query: 23 ttgcttgattcacttcwwyaagag 46
          |||||||||||||||   |||||
Sbjct: 8  ttgcttgattcacttctacaagag 31
```

>FK867832|FK867832.1 CF_W01_13C02_SP6 Copepod Whole Organism, Normalized *Calanus finmarchicus* cDNA clone CF_W01_13C02; 5' similar to ref|XP_001640945.1| predicted protein - Nematostella vectensis. Score = 360 bits (923), E. Length = 671; score = 40.1 bits (18), expect = 0.83; identities = 21/24 (87%); strand = Plus / Plus

```
Query: 23 ttgcttgattcacttcwwyaagag 46
          |||||||||||||||   |||||
Sbjct: 7  ttgcttgattcacttctacaagag 30
```

>FK867824|FK867824.1 CF_W01_13B04_SP6 Copepod Whole Organism, Normalized *Calanus finmarchicus* cDNA clone CF_W01_13B04; 5' similar to emb|CAG07918.1| unnamed protein product - Tetraodon nigroviridis. Score = 268 bits (684), Length = 639; score = 40.1 bits (18), expect = 0.83; identities = 21/24 (87%); strand = Plus / Plus

```
Query: 23 ttgcttgattcacttcwwyaagag 46
          |||||||||||||||   |||||
Sbjct: 8  ttgcttgattcacttctacaagag 31
```

>FK867816|FK867816.1 CF_W01_13A07_SP6 Copepod Whole Organism, Normalized *Calanus finmarchicus* cDNA clone CF_W01_13A07; 5' similar to gb|EDV21370.1| hypothetical protein TRIADDRAFT_60323 - Trichoplax adhaerens. Score = 80.9 Length = 647; score = 40.1 bits (18), expect = 0.83; identities = 21/24 (87%); strand = Plus / Plus

```
Query: 23 ttgcttgattcacttcwwyaagag 46
          |||||||||||||||   |||||
Sbjct: 9  ttgcttgattcacttctacaagag 32
```

>FK867812|FK867812.1 CF_W01_13A03_SP6 Copepod Whole Organism, Normalized *Calanus finmarchicus* cDNA clone CF_W01_13A03; 5', mRNA sequence. Length = 682; score = 40.1 bits (18), expect = 0.83; identities = 21/24 (87%); strand = Plus / Plus

```
Query: 23 ttgcttgattcacttcwwyaagag 46
          |||||||||||||||   |||||
Sbjct: 9  ttgcttgattcacttctacaagag 32
```

>FK867772|FK867772.1 CF_WO1_12E07_SP6 Copepod Whole Organism, Normalized *Calanus finmarchicus* cDNA clone CF_WO1_12E07; 5' , mRNA sequence. Length = 617; score = 40.1 bits (18), expect = 0.83; identities = 21/24 (87%); strand = Plus / Plus

```
Query: 23 ttgcttgattcacttcwwyaagag 46
          ||||||||||||||||   |||||
Sbjct: 2  ttgcttgattcacttctacaagag 25
```

>FK867740|FK867740.1 CF_WO1_12B07_SP6 Copepod Whole Organism, Normalized *Calanus finmarchicus* cDNA clone CF_WO1_12B07; 5' similar to ref|YP_960064.1| hypothetical protein Maqu_2802 - Marinobacter aquaeolei VT8. Score = 169 Length = 660; score = 40.1 bits (18), expect = 0.83; identities = 21/24 (87%); strand = Plus / Plus

```
Query: 23 ttgcttgattcacttcwwyaagag 46
          ||||||||||||||||   |||||
Sbjct: 17 ttgcttgattcacttctacaagag 40
```

>FK867631|FK867631.1 CF_WO1_10H01_SP6 Copepod Whole Organism, Normalized *Calanus finmarchicus* cDNA clone CF_WO1_10H01; 5' , mRNA sequence. Length = 619; score = 40.1 bits (18), expect = 0.83; identities = 21/24 (87%); strand = Plus / Plus

```
Query: 23 ttgcttgattcacttcwwyaagag 46
          ||||||||||||||||   |||||
Sbjct: 7  ttgcttgattcacttctacaagag 30
```

>FK867617|FK867617.1 CF_WO1_10F11_SP6 Copepod Whole Organism, Normalized *Calanus finmarchicus* cDNA clone CF_WO1_10F11; 5' similar to ref|XP_789229.1| DIVDICTED: hypothetical protein, partial [Strongylocentrotus. Score = 59 Length = 687; score = 40.1 bits (18), expect = 0.83; identities = 21/24 (87%); strand = Plus / Plus

```
Query: 23 ttgcttgattcacttcwwyaagag 46
          ||||||||||||||||   |||||
Sbjct: 14 ttgcttgattcacttctacaagag 37
```

>FK867584|FK867584.1 CF_WO1_10C12_SP6 Copepod Whole Organism, Normalized *Calanus finmarchicus* cDNA clone CF_WO1_10C12; 5' similar to ref|XP_001951828.1| DIVDICTED: similar to nuclear protein localization [Acyrthosiphon. Sc Length = 694; score = 40.1 bits (18), expect = 0.83; identities = 21/24 (87%); strand = Plus / Plus

```
Query: 23 ttgcttgattcacttcwwyaagag 46
          ||||||||||||||||   |||||
Sbjct: 7  ttgcttgattcacttctacaagag 30
```

>FK867559|FK867559.1 CF_WO1_10A06_SP6 Copepod Whole Organism, Normalized *Calanus finmarchicus* cDNA clone CF_WO1_10A06; 5' similar to ref|XP_001600933.1| DIVDICTED: similar to 6-phosphogluconate dehydrogenase [Nasonia. Scor Length = 752; score = 40.1 bits (18), expect = 0.83; identities = 21/24 (87%); strand = Plus / Plus

```
Query: 23 ttgcttgattcacttcwwyaagag 46
```

```
         |||||||||||||||   |||||
Sbjct: 7  ttgcttgattcacttctacaagag 30
```

>FK867554|FK867554.1 CF_W01_10A01_SP6 Copepod Whole Organism, Normalized *Calanus finmarchicus* cDNA clone CF_W01_10A01; 5' similar to ref|XP_001846318.1| zinc finger protein - Culex pipiens quinquefasciatus. Score = 137 bit Length = 648; score = 40.1 bits (18), expect = 0.83; identities = 21/24 (87%); strand = Plus / Plus

```
Query: 23 ttgcttgattcacttcwwyaagag 46
          |||||||||||||||   |||||
Sbjct: 6  ttgcttgattcacttctacaagag 29
```

>FK867528|FK867528.1 CF_W01_09F11_SP6 Copepod Whole Organism, Normalized *Calanus finmarchicus* cDNA clone CF_W01_09F11; 5' similar to gb|EDV45797.1| GG18546 - Drosophila erecta. Score = 58.2 bits (139), expect = 5e-07, mRNA Length = 689; score = 40.1 bits (18), expect = 0.83; identities = 21/24 (87%); strand = Plus / Plus

```
Query: 23 ttgcttgattcacttcwwyaagag 46
          |||||||||||||||   |||||
Sbjct: 15 ttgcttgattcacttctacaagag 38
```

>FK867508|FK867508.1 CF_W01_09E03_SP6 Copepod Whole Organism, Normalized *Calanus finmarchicus* cDNA clone CF_W01_09E03; 5' similar to ref|XP_001605076.1| DIVDICTED: similar to ENSANGP00000009647 - Nasonia vitripennis. Score Length = 612; score = 40.1 bits (18), expect = 0.83; identities = 21/24 (87%); strand = Plus / Plus

```
Query: 23 ttgcttgattcacttcwwyaagag 46
          |||||||||||||||   |||||
Sbjct: 1  ttgcttgattcacttctacaagag 24
```

>FK867483|FK867483.1 CF_W01_09C01_SP6 Copepod Whole Organism, Normalized *Calanus finmarchicus* cDNA clone CF_W01_09C01; 5', mRNA sequence. Length = 676; score = 40.1 bits (18), expect = 0.83; identities = 21/24 (87%); strand = Plus / Plus

```
Query: 23 ttgcttgattcacttcwwyaagag 46
          |||||||||||||||   |||||
Sbjct: 13 ttgcttgattcacttctacaagag 36
```

>FK671294|FK671294.1 CF_W01_08E07_SP6 Copepod Whole Organism, Normalized *Calanus finmarchicus* cDNA clone CF_W01_08E07; 5' similar to ref|XP_001500273.1| DIVDICTED: similar to boule - Equus caballus. Score = 65.1 bits (157) Length = 673; score = 40.1 bits (18), expect = 0.83; identities = 21/24 (87%); strand = Plus / Plus

```
Query: 23 ttgcttgattcacttcwwyaagag 46
          |||||||||||||||   |||||
Sbjct: 3  ttgcttgattcacttctacaagag 26
```

>FK671286|FK671286.1 CF_W01_08D10_SP6 Copepod Whole Organism, Normalized *Calanus finmarchicus* cDNA clone CF_W01_08D10; 5' similar to ref|XP_311527.2| AGAP010421-PA - Anopheles gambiae

str. PEST. Score = 267 bits (683), Exp Length = 557; score = 40.1 bits (18), expect = 0.83; identities = 21/24 (87%); strand = Plus / Plus

```
Query: 23 ttgcttgattcacttcwwyaagag 46
          ||||||||||||||||   |||||
Sbjct: 9  ttgcttgattcacttctacaagag 32
```

>FK671283|FK671283.1 CF_W01_08D07_SP6 Copepod Whole Organism, Normalized *Calanus finmarchicus* cDNA clone CF_W01_08D07; 5' , mRNA sequence. Length = 649; score = 40.1 bits (18), expect = 0.83; identities = 21/24 (87%); strand = Plus / Plus

```
Query: 23 ttgcttgattcacttcwwyaagag 46
          ||||||||||||||||   |||||
Sbjct: 9  ttgcttgattcacttctacaagag 32
```

>FK671247|FK671247.1 CF_W01_07H12_SP6 Copepod Whole Organism, Normalized *Calanus finmarchicus* cDNA clone CF_W01_07H12; 5' similar to ref|XP_973781.1| DIVDICTED: similar to actin - Tribolium castaneum. Score = 190 bits (482 Length = 624; score = 40.1 bits (18), expect = 0.83; identities = 21/24 (87%); strand = Plus / Plus

```
Query: 23 ttgcttgattcacttcwwyaagag 46
          ||||||||||||||||   |||||
Sbjct: 7  ttgcttgattcacttctacaagag 30
```

>FK671178|FK671178.1 CF_W01_07B11_SP6 Copepod Whole Organism, Normalized *Calanus finmarchicus* cDNA clone CF_W01_07B11; 5' similar to ref|XP_001943219.1| DIVDICTED: similar to conserved hypothetical protein [Acyrthosiphon. Length = 677; score = 40.1 bits (18), expect = 0.83; identities = 21/24 (87%); strand = Plus / Plus

```
Query: 23 ttgcttgattcacttcwwyaagag 46
          ||||||||||||||||   |||||
Sbjct: 12 ttgcttgattcacttctacaagag 35
```

>FK671164|FK671164.1 CF_W01_07A08_SP6 Copepod Whole Organism, Normalized *Calanus finmarchicus* cDNA clone CF_W01_07A08; 5' similar to ref|XP_625109.2| DIVDICTED: similar to Numb-associated kinase CG10637-PA, isoform A. Scor Length = 606; score = 40.1 bits (18), expect = 0.83; identities = 21/24 (87%); strand = Plus / Plus

```
Query: 23 ttgcttgattcacttcwwyaagag 46
          ||||||||||||||||   |||||
Sbjct: 8  ttgcttgattcacttctacaagag 31
```

>FK671162|FK671162.1 CF_W01_07A06_SP6 Copepod Whole Organism, Normalized *Calanus finmarchicus* cDNA clone CF_W01_07A06; 5' , mRNA sequence. Length = 677; score = 40.1 bits (18), expect = 0.83; identities = 21/24 (87%); strand = Plus / Plus

```
Query: 23 ttgcttgattcacttcwwyaagag 46
          ||||||||||||||||   |||||
Sbjct: 12 ttgcttgattcacttctacaagag 35
```

>FK671029|FK671029.1 CF_W01_05E12_SP6 Copepod Whole Organism, Normalized *Calanus finmarchicus* cDNA clone CF_W01_05E12; 5' similar to ref|XP_394284.2| DIVDICTED: similar to Ubiquitin ligase SIAH1 (Seven in absentia. Score = Length = 612; score = 40.1 bits (18), expect = 0.83; identities = 31/38 (81%), Gaps = 1/38 (2%); strand = Plus / Plus

```
Query: 10 gaccaagctwawwt-tgcttgattcacttcwwyaagag 46
          ||||||||| |  | |||||||||||||||   |||||
Sbjct: 3  gaccaagctaatatctgcttgattcacttctacaagag 40
```

>FK671028|FK671028.1 CF_W01_05E11_SP6 Copepod Whole Organism, Normalized *Calanus finmarchicus* cDNA clone CF_W01_05E11; 5' similar to ref|XP_394284.2| DIVDICTED: similar to Ubiquitin ligase SIAH1 (Seven in absentia. Score = Length = 659; score = 40.1 bits (18), expect = 0.83; identities = 31/38 (81%), Gaps = 1/38 (2%); strand = Plus / Plus

```
Query: 10 gaccaagctwawwt-tgcttgattcacttcwwyaagag 46
          ||||||||| |  | |||||||||||||||   |||||
Sbjct: 3  gaccaagctaatatctgcttgattcacttctacaagag 40
```

>FK671014|FK671014.1 CF_W01_05D07_SP6 Copepod Whole Organism, Normalized *Calanus finmarchicus* cDNA clone CF_W01_05D07; 5' similar to ref|XP_001898673.1| Protein-tyrosine phosphatase containing protein - Brugia malayi. Scor Length = 606; score = 40.1 bits (18), expect = 0.83; identities = 21/24 (87%); strand = Plus / Plus

```
Query: 23 ttgcttgattcacttcwwyaagag 46
          ||||||||||||||||   |||||
Sbjct: 10 ttgcttgattcacttctacaagag 33
```

>FK671010|FK671010.1 CF_W01_05D02_SP6 Copepod Whole Organism, Normalized *Calanus finmarchicus* cDNA clone CF_W01_05D02; 5' similar to gb|EDV23485.1| hypothetical protein TRIADDRAFT_28052 - Trichoplax adhaerens. Score = 154 Length = 608; score = 40.1 bits (18), expect = 0.83; identities = 21/24 (87%); strand = Plus / Plus

```
Query: 23 ttgcttgattcacttcwwyaagag 46
          ||||||||||||||||   |||||
Sbjct: 10 ttgcttgattcacttctacaagag 33
```

>FK671000|FK671000.1 CF_W01_05C01_SP6 Copepod Whole Organism, Normalized *Calanus finmarchicus* cDNA clone CF_W01_05C01; 5' similar to gb|EDV23485.1| hypothetical protein TRIADDRAFT_28052 - Trichoplax adhaerens. Score = 169 Length = 648; score = 40.1 bits (18), expect = 0.83; identities = 21/24 (87%); strand = Plus / Plus

```
Query: 23 ttgcttgattcacttcwwyaagag 46
          ||||||||||||||||   |||||
Sbjct: 9  ttgcttgattcacttctacaagag 32
```

>FK670960|FK670960.1 CF_W01_04G06_SP6 Copepod Whole Organism, Normalized *Calanus finmarchicus* cDNA clone CF_W01_04G06; 5' similar to ref|NP_001026523.1| pyruvate dehydrogenase kinase,

isoenzyme 1 - Gallus gallus. Score = 5 Length = 676; score = 40.1 bits (18), expect = 0.83; identities = 21/24 (87%); strand = Plus / Plus

```
Query: 23 ttgcttgattcacttcwwyaagag 46
          |||||||||||||||   |||||
Sbjct: 15 ttgcttgattcacttctacaagag 38
```

>FK670959|FK670959.1 CF_W01_04G05_SP6 Copepod Whole Organism, Normalized *Calanus finmarchicus* cDNA clone CF_W01_04G05; 5' , mRNA sequence. Length = 571; score = 40.1 bits (18), expect = 0.83; identities = 21/24 (87%); strand = Plus / Plus

```
Query: 23 ttgcttgattcacttcwwyaagag 46
          |||||||||||||||   |||||
Sbjct: 15 ttgcttgattcacttctacaagag 38
```

>FK670947|FK670947.1 CF_W01_04F05_SP6 Copepod Whole Organism, Normalized *Calanus finmarchicus* cDNA clone CF_W01_04F05; 5' similar to ref|NP_001121006.1| notchless homolog 1 - Rattus norvegicus. Score = 167 bits (423), Expe Length = 581; score = 40.1 bits (18), expect = 0.83; identities = 21/24 (87%); strand = Plus / Plus

```
Query: 23 ttgcttgattcacttcwwyaagag 46
          |||||||||||||||   |||||
Sbjct: 14 ttgcttgattcacttctacaagag 37
```

>FK670932|FK670932.1 CF_W01_04E01_SP6 Copepod Whole Organism, Normalized *Calanus finmarchicus* cDNA clone CF_W01_04E01; 5' similar to ref|XP_001848582.1| conserved hypothetical protein - Culex pipiens quinquefasciatus. Scor Length = 657; score = 40.1 bits (18), expect = 0.83; identities = 21/24 (87%); strand = Plus / Plus

```
Query: 23 ttgcttgattcacttcwwyaagag 46
          |||||||||||||||   |||||
Sbjct: 14 ttgcttgattcacttctacaagag 37
```

>FK670909|FK670909.1 CF_W01_04C02_SP6 Copepod Whole Organism, Normalized *Calanus finmarchicus* cDNA clone CF_W01_04C02; 5' similar to ref|XP_001606649.1| DIVDICTED: similar to GA19143-PA - Nasonia vitripennis. Score = 148 b Length = 679; score = 40.1 bits (18), expect = 0.83; identities = 21/24 (87%); strand = Plus / Plus

```
Query: 23 ttgcttgattcacttcwwyaagag 46
          |||||||||||||||   |||||
Sbjct: 10 ttgcttgattcacttctacaagag 33
```

>FK670895|FK670895.1 CF_W01_04A10_SP6 Copepod Whole Organism, Normalized *Calanus finmarchicus* cDNA clone CF_W01_04A10; 5' similar to ref|XP_969316.1| DIVDICTED: similar to tyrosine phosphatase, non-receptor type nt1. Score Length = 678; score = 40.1 bits (18), expect = 0.83; identities = 21/24 (87%); strand = Plus / Plus

```
Query: 23 ttgcttgattcacttcwwyaagag 46
          |||||||||||||||   |||||
```

Sbjct: 12 ttgcttgattcacttctacaagag 35

>FK670893|FK670893.1 CF_W01_04A08_SP6 Copepod Whole Organism, Normalized *Calanus finmarchicus* cDNA clone CF_W01_04A08; 5' similar to ref|XP_969316.1| DIVDICTED: similar to tyrosine phosphatase, non-receptor type nt1. Score Length = 690; score = 40.1 bits (18), expect = 0.83; identities = 21/24 (87%); strand = Plus / Plus

```
Query: 23 ttgcttgattcacttcwwyaagag 46
          |||||||||||||||   |||||
Sbjct: 12 ttgcttgattcacttctacaagag 35
```

>FK670886|FK670886.1 CF_W01_04A01_SP6 Copepod Whole Organism, Normalized *Calanus finmarchicus* cDNA clone CF_W01_04A01; 5' , mRNA sequence. Length = 686; score = 40.1 bits (18), expect = 0.83; identities = 21/24 (87%); strand = Plus / Plus

```
Query: 23 ttgcttgattcacttcwwyaagag 46
          |||||||||||||||   |||||
Sbjct: 10 ttgcttgattcacttctacaagag 33
```

>FK670860|FK670860.1 CF_W01_03F05_SP6 Copepod Whole Organism, Normalized *Calanus finmarchicus* cDNA clone CF_W01_03F05; 5' , mRNA sequence. Length = 623; score = 40.1 bits (18), expect = 0.83; identities = 21/24 (87%); strand = Plus / Plus

```
Query: 23 ttgcttgattcacttcwwyaagag 46
          |||||||||||||||   |||||
Sbjct: 14 ttgcttgattcacttctacaagag 37
```

>FK670807|FK670807.1 CF_W01_02H10_SP6 Copepod Whole Organism, Normalized *Calanus finmarchicus* cDNA clone CF_W01_02H10; 5' similar to gb|AAU84927.1| putative 26S protease regulatory subunit 8 - Toxoptera citricida. Score = Length = 654; score = 40.1 bits (18), expect = 0.83; identities = 21/24 (87%); strand = Plus / Plus

```
Query: 23 ttgcttgattcacttcwwyaagag 46
          |||||||||||||||   |||||
Sbjct: 12 ttgcttgattcacttctacaagag 35
```

>FK670790|FK670790.1 CF_W01_02G05_SP6 Copepod Whole Organism, Normalized *Calanus finmarchicus* cDNA clone CF_W01_02G05; 5' , mRNA sequence. Length = 645; score = 40.1 bits (18), expect = 0.83; identities = 21/24 (87%); strand = Plus / Plus

```
Query: 23 ttgcttgattcacttcwwyaagag 46
          |||||||||||||||   |||||
Sbjct: 2  ttgcttgattcacttctacaagag 25
```

>FK670749|FK670749.1 CF_W01_02C11_SP6 Copepod Whole Organism, Normalized *Calanus finmarchicus* cDNA clone CF_W01_02C11; 5' similar to sp|Q5ZHZ0|UAP56_CHICK Spliceosome RNA helicase BAT1 (DEAD box protein UAP56) (56 kDa. Sco Length = 631; score = 40.1 bits (18), expect = 0.83; identities = 21/24 (87%); strand = Plus / Plus

```
Query: 23 ttgcttgattcacttcwwyaagag 46
          |||||||||||||||   |||||
Sbjct: 12 ttgcttgattcacttctacaagag 35
```

>FK670731|FK670731.1 CF_W01_02B05_SP6 Copepod Whole Organism, Normalized *Calanus finmarchicus* cDNA clone CF_W01_02B05; 5' , mRNA sequence. Length = 680; score = 40.1 bits (18), expect = 0.83; identities = 21/24 (87%); strand = Plus / Plus

```
Query: 23 ttgcttgattcacttcwwyaagag 46
          |||||||||||||||   |||||
Sbjct: 9  ttgcttgattcacttctacaagag 32
```

>FK670700|FK670700.1 CF_W01_01G09_SP6 Copepod Whole Organism, Normalized *Calanus finmarchicus* cDNA clone CF_W01_01G09; 5' , mRNA sequence. Length = 545; score = 40.1 bits (18), expect = 0.83; identities = 21/24 (87%); strand = Plus / Plus

```
Query: 23 ttgcttgattcacttcwwyaagag 46
          |||||||||||||||   |||||
Sbjct: 10 ttgcttgattcacttctacaagag 33
```

>FK670599|FK670599.1 CF_W01_00D11_SP6 Copepod Whole Organism, Normalized *Calanus finmarchicus* cDNA clone CF_W01_00D11; 5' similar to ref|NP_648345.1| CG6767 CG6767-PA, isoform A - Drosophila melanogaster. Score = 377 bits Length = 675; score = 40.1 bits (18), expect = 0.83; identities = 21/24 (87%); strand = Plus / Plus

```
Query: 23 ttgcttgattcacttcwwyaagag 46
          |||||||||||||||   |||||
Sbjct: 2  ttgcttgattcacttctacaagag 25
```

>FK670591|FK670591.1 CF_W01_00D03_SP6 Copepod Whole Organism, Normalized *Calanus finmarchicus* cDNA clone CF_W01_00D03; 5' , mRNA sequence. Length = 696; score = 40.1 bits (18), expect = 0.83; identities = 21/24 (87%); strand = Plus / Plus

```
Query: 23 ttgcttgattcacttcwwyaagag 46
          |||||||||||||||   |||||
Sbjct: 2  ttgcttgattcacttctacaagag 25
```

>FK670590|FK670590.1 CF_W01_00D02_SP6 Copepod Whole Organism, Normalized *Calanus finmarchicus* cDNA clone CF_W01_00D02; 5' similar to ref|XP_001601157.1| DIVDICTED: similar to conserved hypothetical protein [Nasonia. Score Length = 704; score = 40.1 bits (18), expect = 0.83; identities = 21/24 (87%); strand = Plus / Plus

```
Query: 23 ttgcttgattcacttcwwyaagag 46
          |||||||||||||||   |||||
Sbjct: 13 ttgcttgattcacttctacaagag 36
```

>FK670577|FK670577.1 CF_W01_00C01_SP6 Copepod Whole Organism, Normalized *Calanus finmarchicus* cDNA clone CF_W01_00C01; 5' , mRNA sequence. Length = 688; score = 40.1 bits (18), expect = 0.83; identities = 21/24 (87%); strand = Plus / Plus

```
Query: 23 ttgcttgattcacttcwwyaagag 46
          ||||||||||||||||   |||||
Sbjct: 7  ttgcttgattcacttctacaagag 30
```

>FK670546|FK670546.1 CF_WO0_99H03_SP6 Copepod Whole Organism, Normalized *Calanus finmarchicus* cDNA clone CF_WO0_99H03; 5' , mRNA sequence. Length = 679; score = 40.1 bits (18), expect = 0.83; identities = 21/24 (87%); strand = Plus / Plus

```
Query: 23 ttgcttgattcacttcwwyaagag 46
          ||||||||||||||||   |||||
Sbjct: 10 ttgcttgattcacttctacaagag 33
```

>FK670522|FK670522.1 CF_WO0_99F02_SP6 Copepod Whole Organism, Normalized *Calanus finmarchicus* cDNA clone CF_WO0_99F02; 5' similar to gb|EDV27584.1| hypothetical protein TRIADDRAFT_20897 - Trichoplax adhaerens. Score = 156 Length = 612; score = 40.1 bits (18), expect = 0.83; identities = 21/24 (87%); strand = Plus / Plus

```
Query: 23 ttgcttgattcacttcwwyaagag 46
          ||||||||||||||||   |||||
Sbjct: 15 ttgcttgattcacttctacaagag 38
```

>FK670514|FK670514.1 CF_WO0_99E04_SP6 Copepod Whole Organism, Normalized *Calanus finmarchicus* cDNA clone CF_WO0_99E04; 5' similar to ref|XP_317563.2| AGAP007921-PA - Anopheles gambiae str. PEST. Score = 245 bits (626), Exp Length = 647; score = 40.1 bits (18), expect = 0.83; identities = 21/24 (87%); strand = Plus / Plus

```
Query: 23 ttgcttgattcacttcwwyaagag 46
          ||||||||||||||||   |||||
Sbjct: 5  ttgcttgattcacttctacaagag 28
```

>FK670484|FK670484.1 CF_WO0_98H09_SP6 Copepod Whole Organism, Normalized *Calanus finmarchicus* cDNA clone CF_WO0_98H09; 5' similar to dbj|BAF85529.1| unnamed protein product - Homo sapiens. Score = 58.9 bits (141), expect = Length = 581; score = 40.1 bits (18), expect = 0.83; identities = 21/24 (87%); strand = Plus / Plus

```
Query: 23 ttgcttgattcacttcwwyaagag 46
          ||||||||||||||||   |||||
Sbjct: 6  ttgcttgattcacttctacaagag 29
```

>FK670426|FK670426.1 CF_WO0_98C06_SP6 Copepod Whole Organism, Normalized *Calanus finmarchicus* cDNA clone CF_WO0_98C06; 5' similar to ref|ZP_02977152.1| conserved hypothetical protein - Cyanothece sp. PCC 7424. Score = 61.2 Length = 700; score = 40.1 bits (18), expect = 0.83; identities = 21/24 (87%); strand = Plus / Plus

```
Query: 23 ttgcttgattcacttcwwyaagag 46
          ||||||||||||||||   |||||
Sbjct: 10 ttgcttgattcacttctacaagag 33
```

>FK670421|FK670421.1 CF_WO0_98C01_SP6 Copepod Whole Organism, Normalized *Calanus finmarchicus* cDNA clone CF_WO0_98C01; 5' similar to ref|NP_001079462.1| similar to RNA, U transporter 1 - Xenopus laevis. Score = 118 bits (2 Length = 729; score = 40.1 bits (18), expect = 0.83; identities = 21/24 (87%); strand = Plus / Plus

```
Query: 23 ttgcttgattcacttcwwyaagag 46
          ||||||||||||||||   |||||
Sbjct: 13 ttgcttgattcacttctacaagag 36
```

>FK670419|FK670419.1 CF_WO0_98B11_SP6 Copepod Whole Organism, Normalized *Calanus finmarchicus* cDNA clone CF_WO0_98B11; 5' similar to gb|EDV35017.1| GF22422 - Drosophila ananassae. Score = 224 bits (572), expect = 3e-57, mR Length = 671; score = 40.1 bits (18), expect = 0.83; identities = 21/24 (87%); strand = Plus / Plus

```
Query: 23 ttgcttgattcacttcwwyaagag 46
          ||||||||||||||||   |||||
Sbjct: 13 ttgcttgattcacttctacaagag 36
```

>FK670413|FK670413.1 CF_WO0_98B05_SP6 Copepod Whole Organism, Normalized *Calanus finmarchicus* cDNA clone CF_WO0_98B05; 5' similar to ref|XP_001639996.1| predicted protein - Nematostella vectensis. Score = 243 bits (621), E. Length = 735; score = 40.1 bits (18), expect = 0.83; identities = 21/24 (87%); strand = Plus / Plus

```
Query: 23 ttgcttgattcacttcwwyaagag 46
          ||||||||||||||||   |||||
Sbjct: 10 ttgcttgattcacttctacaagag 33
```

>FK670375|FK670375.1 CF_WO0_97F11_SP6 Copepod Whole Organism, Normalized *Calanus finmarchicus* cDNA clone CF_WO0_97F11; 5' similar to ref|XP_969882.1| DIVDICTED: similar to aldehyde dehydrogenase 7 family, member A1. Score Length = 688; score = 40.1 bits (18), expect = 0.83; identities = 21/24 (87%); strand = Plus / Plus

```
Query: 23 ttgcttgattcacttcwwyaagag 46
          ||||||||||||||||   |||||
Sbjct: 7  ttgcttgattcacttctacaagag 30
```

>FK041651|FK041651.1 CF_WO0_96G04_SP6 Copepod Whole Organism, Normalized *Calanus finmarchicus* cDNA clone CF_WO0_96G04; 5' similar to ref|XP_392302.2| DIVDICTED: similar to Excitatory amino acid transporter 2. Score = 141 b Length = 669; score = 40.1 bits (18), expect = 0.83; identities = 21/24 (87%); strand = Plus / Plus

```
Query: 23 ttgcttgattcacttcwwyaagag 46
          ||||||||||||||||   |||||
Sbjct: 9  ttgcttgattcacttctacaagag 32
```

>FK041626|FK041626.1 CF_WO0_96E01_SP6 Copepod Whole Organism, Normalized *Calanus finmarchicus* cDNA clone CF_WO0_96E01; 5' similar to ref|XP_001621948.1| hypothetical protein NEMVEDRAFT_v1g143109 - Nematostella vectensis. S Length = 695; score = 40.1 bits (18), expect = 0.83; identities = 21/24 (87%); strand = Plus / Plus

```
Query: 23 ttgcttgattcacttcwwyaagag 46
          |||||||||||||||   |||||
Sbjct: 11 ttgcttgattcacttctacaagag 34
```

>FK041588|FK041588.1 CF_WO0_96A09_SP6 Copepod Whole Organism, Normalized *Calanus finmarchicus* cDNA clone CF_WO0_96A09; 5' similar to ref|XP_395364.2| DIVDICTED: similar to prolyl endopeptidase isoform 1 [Apis. Score = 255 Length = 713; score = 40.1 bits (18), expect = 0.83; identities = 21/24 (87%); strand = Plus / Plus

```
Query: 23 ttgcttgattcacttcwwyaagag 46
          |||||||||||||||   |||||
Sbjct: 7  ttgcttgattcacttctacaagag 30
```

>FK041557|FK041557.1 CF_WO0_95G01_SP6 Copepod Whole Organism, Normalized *Calanus finmarchicus* cDNA clone CF_WO0_95G01; 5' similar to ref|XP_001655206.1| transcription factor IIIB 90 kDa subunit (TFIIIB90) - Aedes aegypti. Length = 698; score = 40.1 bits (18), expect = 0.83; identities = 21/24 (87%); strand = Plus / Plus

```
Query: 23 ttgcttgattcacttcwwyaagag 46
          |||||||||||||||   |||||
Sbjct: 8  ttgcttgattcacttctacaagag 31
```

>FK041553|FK041553.1 CF_WO0_95F08_SP6 Copepod Whole Organism, Normalized *Calanus finmarchicus* cDNA clone CF_WO0_95F08; 5' similar to gb|EDV49959.1| GG23811 - Drosophila erecta. Score = 107 bits (268), expect = 5e-22, mRNA Length = 675; score = 40.1 bits (18), expect = 0.83; identities = 21/24 (87%); strand = Plus / Plus

```
Query: 23 ttgcttgattcacttcwwyaagag 46
          |||||||||||||||   |||||
Sbjct: 14 ttgcttgattcacttctacaagag 37
```

>FK041549|FK041549.1 CF_WO0_95F02_SP6 Copepod Whole Organism, Normalized *Calanus finmarchicus* cDNA clone CF_WO0_95F02; 5' , mRNA sequence. Length = 698; score = 40.1 bits (18), expect = 0.83; identities = 21/24 (87%); strand = Plus / Plus

```
Query: 23 ttgcttgattcacttcwwyaagag 46
          |||||||||||||||   |||||
Sbjct: 13 ttgcttgattcacttctacaagag 36
```

>FK041512|FK041512.1 CF_WO0_95B07_SP6 Copepod Whole Organism, Normalized *Calanus finmarchicus* cDNA clone CF_WO0_95B07; 5' similar to ref|XP_001600537.1| DIVDICTED: similar to ENSANGP00000018867 - Nasonia vitripennis. Score Length = 739; score = 40.1 bits (18), expect = 0.83; identities = 21/24 (87%); strand = Plus / Plus

```
Query: 23 ttgcttgattcacttcwwyaagag 46
          |||||||||||||||   |||||
Sbjct: 14 ttgcttgattcacttctacaagag 37
```

>FK041452|FK041452.1 CF_WO0_94E05_SP6 Copepod Whole Organism, Normalized *Calanus finmarchicus* cDNA clone CF_WO0_94E05; 5' similar to ref|XP_624111.1| DIVDICTED: similar to CG1416-PA, isoform A isoform 2 [Apis. Score = 203 Length = 669; score = 40.1 bits (18), expect = 0.83; identities = 21/24 (87%); strand = Plus / Plus

```
Query: 23 ttgcttgattcacttcwwyaagag 46
          ||||||||||||||||   |||||
Sbjct: 12 ttgcttgattcacttctacaagag 35
```

>FK041440|FK041440.1 CF_WO0_94D05_SP6 Copepod Whole Organism, Normalized *Calanus finmarchicus* cDNA clone CF_WO0_94D05; 5' similar to emb|CAF97808.1| unnamed protein product - Tetraodon nigroviridis. Score = 206 bits (523), Length = 613; score = 40.1 bits (18), expect = 0.83; identities = 21/24 (87%); strand = Plus / Plus

```
Query: 23 ttgcttgattcacttcwwyaagag 46
          ||||||||||||||||   |||||
Sbjct: 7  ttgcttgattcacttctacaagag 30
```

>FK041357|FK041357.1 CF_WO0_93D10_SP6 Copepod Whole Organism, Normalized *Calanus finmarchicus* cDNA clone CF_WO0_93D10; 5' similar to ref|XP_001175790.1| DIVDICTED: hypothetical protein isoform 2 [Strongylocentrotus. Score Length = 718; score = 40.1 bits (18), expect = 0.83; identities = 21/24 (87%); strand = Plus / Plus

```
Query: 23 ttgcttgattcacttcwwyaagag 46
          ||||||||||||||||   |||||
Sbjct: 7  ttgcttgattcacttctacaagag 30
```

>FK041347|FK041347.1 CF_WO0_93C10_SP6 Copepod Whole Organism, Normalized *Calanus finmarchicus* cDNA clone CF_WO0_93C10; 5' similar to ref|XP_966693.2| DIVDICTED: similar to vacuolar ATP synthase subunit h isoform 1. Score = Length = 737; score = 40.1 bits (18), expect = 0.83; identities = 21/24 (87%); strand = Plus / Plus

```
Query: 23 ttgcttgattcacttcwwyaagag 46
          ||||||||||||||||   |||||
Sbjct: 5  ttgcttgattcacttctacaagag 28
```

>FK041325|FK041325.1 CF_WO0_93A10_SP6 Copepod Whole Organism, Normalized *Calanus finmarchicus* cDNA clone CF_WO0_93A10; 5' similar to ref|XP_394933.3| DIVDICTED: similar to Arginine methyltransferase 4 CG5358-PA [Apis. Scor Length = 690; score = 40.1 bits (18), expect = 0.83; identities = 21/24 (87%); strand = Plus / Plus

```
Query: 23 ttgcttgattcacttcwwyaagag 46
          ||||||||||||||||   |||||
Sbjct: 18 ttgcttgattcacttctacaagag 41
```

>FK041322|FK041322.1 CF_WO0_93A07_SP6 Copepod Whole Organism, Normalized *Calanus finmarchicus* cDNA clone CF_WO0_93A07; 5' similar to ref|XP_001640945.1| predicted protein - Nematostella vectensis. Score = 288 bits (737), E. Length = 573; score = 40.1 bits (18), expect = 0.83; identities = 21/24 (87%); strand = Plus / Plus

Query: 23 ttgcttgattcacttcwwyaagag 46
         ||||||||||||||| |||||
Sbjct: 9 ttgcttgattcacttctacaagag 32

>FK041320|FK041320.1 CF_WOO_93A05_SP6 Copepod Whole Organism, Normalized *Calanus finmarchicus* cDNA clone CF_WOO_93A05; 5' similar to ref|NP_001002657.1| hypothetical protein LOC436930 - Danio rerio. Score = 112 bits (279), Length = 695; score = 40.1 bits (18), expect = 0.83; identities = 21/24 (87%); strand = Plus / Plus

Query: 23 ttgcttgattcacttcwwyaagag 46
         ||||||||||||||| |||||
Sbjct: 14 ttgcttgattcacttctacaagag 37

>FK041317|FK041317.1 CF_WOO_93A02_SP6 Copepod Whole Organism, Normalized *Calanus finmarchicus* cDNA clone CF_WOO_93A02; 5' similar to ref|XP_001605937.1| DIVDICTED: hypothetical protein - Nasonia vitripennis. Score = 177 bi Length = 687; score = 40.1 bits (18), expect = 0.83; identities = 21/24 (87%); strand = Plus / Plus

Query: 23 ttgcttgattcacttcwwyaagag 46
         ||||||||||||||| |||||
Sbjct: 8 ttgcttgattcacttctacaagag 31

>FK041301|FK041301.1 CF_WOO_92G10_SP6 Copepod Whole Organism, Normalized *Calanus finmarchicus* cDNA clone CF_WOO_92G10; 5' similar to ref|XP_001600252.1| DIVDICTED: similar to serine/threonine protein kinase [Nasonia. Score Length = 576; score = 40.1 bits (18), expect = 0.83; identities = 21/24 (87%); strand = Plus / Plus

Query: 23 ttgcttgattcacttcwwyaagag 46
         ||||||||||||||| |||||
Sbjct: 14 ttgcttgattcacttctacaagag 37

>FK041292|FK041292.1 CF_WOO_92G01_SP6 Copepod Whole Organism, Normalized *Calanus finmarchicus* cDNA clone CF_WOO_92G01; 5' similar to ref|XP_974234.1| DIVDICTED: similar to atypical protein kinase C [Tribolium. Score = 244 Length = 605; score = 40.1 bits (18), expect = 0.83; identities = 21/24 (87%); strand = Plus / Plus

Query: 23 ttgcttgattcacttcwwyaagag 46
         ||||||||||||||| |||||
Sbjct: 15 ttgcttgattcacttctacaagag 38

>FK041201|FK041201.1 CF_WOO_91G02_SP6 Copepod Whole Organism, Normalized *Calanus finmarchicus* cDNA clone CF_WOO_91G02; 5' similar to ref|XP_001352362.1| GA14746-PA - Drosophila pseudoobscura. Score = 56.2 bits (134), Expec Length = 626; score = 40.1 bits (18), expect = 0.83; identities = 21/24 (87%); strand = Plus / Plus

Query: 23 ttgcttgattcacttcwwyaagag 46
         ||||||||||||||| |||||
Sbjct: 17 ttgcttgattcacttctacaagag 40

>FK041199|FK041199.1 CF_WO0_91F12_SP6 Copepod Whole Organism, Normalized *Calanus finmarchicus* cDNA clone CF_WO0_91F12; 5' similar to ref|NP_726048.1| CG10069 CG10069-PB, isoform B - Drosophila melanogaster. Score = 193 bit Length = 683; score = 40.1 bits (18), expect = 0.83; identities = 21/24 (87%); strand = Plus / Plus

```
Query: 23 ttgcttgattcacttcwwyaagag 46
          ||||||||||||||||   |||||
Sbjct: 10 ttgcttgattcacttctacaagag 33
```

>FK041194|FK041194.1 CF_WO0_91F07_SP6 Copepod Whole Organism, Normalized *Calanus finmarchicus* cDNA clone CF_WO0_91F07; 5' similar to ref|XP_001607504.1| DIVDICTED: similar to nad dehydrogenase - Nasonia vitripennis. Score Length = 638; score = 40.1 bits (18), expect = 0.83; identities = 21/24 (87%); strand = Plus / Plus

```
Query: 23 ttgcttgattcacttcwwyaagag 46
          ||||||||||||||||   |||||
Sbjct: 10 ttgcttgattcacttctacaagag 33
```

>FK041190|FK041190.1 CF_WO0_91F03_SP6 Copepod Whole Organism, Normalized *Calanus finmarchicus* cDNA clone CF_WO0_91F03; 5' similar to ref|XP_001599178.1| DIVDICTED: similar to mRNA cleavage stimulating factor,. Score = 192 Length = 665; score = 40.1 bits (18), expect = 0.83; identities = 21/24 (87%); strand = Plus / Plus

```
Query: 23 ttgcttgattcacttcwwyaagag 46
          ||||||||||||||||   |||||
Sbjct: 15 ttgcttgattcacttctacaagag 38
```

>FK041189|FK041189.1 CF_WO0_91F02_SP6 Copepod Whole Organism, Normalized *Calanus finmarchicus* cDNA clone CF_WO0_91F02; 5' similar to ref|XP_001599178.1| DIVDICTED: similar to mRNA cleavage stimulating factor,. Score = 195 Length = 682; score = 40.1 bits (18), expect = 0.83; identities = 21/24 (87%); strand = Plus / Plus

```
Query: 23 ttgcttgattcacttcwwyaagag 46
          ||||||||||||||||   |||||
Sbjct: 13 ttgcttgattcacttctacaagag 36
```

>FK041184|FK041184.1 CF_WO0_91E09_SP6 Copepod Whole Organism, Normalized *Calanus finmarchicus* cDNA clone CF_WO0_91E09; 5' similar to ref|XP_971446.1| DIVDICTED: similar to DnaJ homolog subfamily A member 1 [Tribolium. Scor Length = 635; score = 40.1 bits (18), expect = 0.83; identities = 21/24 (87%); strand = Plus / Plus

```
Query: 23 ttgcttgattcacttcwwyaagag 46
          ||||||||||||||||   |||||
Sbjct: 5  ttgcttgattcacttctacaagag 28
```

>FK041162|FK041162.1 CF_WO0_91C04_SP6 Copepod Whole Organism, Normalized *Calanus finmarchicus* cDNA clone CF_WO0_91C04; 5' similar to ref|XP_001108130.1| DIVDICTED: solute carrier family

6, member 7 - Macaca mulatta. Score Length = 684; score = 40.1 bits (18), expect = 0.83; identities = 21/24 (87%); strand = Plus / Plus

```
Query: 23 ttgcttgattcacttcwwyaagag 46
          ||||||||||||||||   |||||
Sbjct: 9  ttgcttgattcacttctacaagag 32
```

>FK041136|FK041136.1 CF_WO0_90H07_SP6 Copepod Whole Organism, Normalized *Calanus finmarchicus* cDNA clone CF_WO0_90H07; 5' similar to gb|AAT77811.1| calpain B - Gecarcinus lateralis. Score = 169 bits (427), expect = 2e-40, Length = 649; score = 40.1 bits (18), expect = 0.83; identities = 21/24 (87%); strand = Plus / Plus

```
Query: 23 ttgcttgattcacttcwwyaagag 46
          ||||||||||||||||   |||||
Sbjct: 7  ttgcttgattcacttctacaagag 30
```

>FK041126|FK041126.1 CF_WO0_90G09_SP6 Copepod Whole Organism, Normalized *Calanus finmarchicus* cDNA clone CF_WO0_90G09; 5' similar to gb|AAO15914.1|AF510054_1 vasa-like - Schistocerca gregaria. Score = 171 bits (434), Expec Length = 590; score = 40.1 bits (18), expect = 0.83; identities = 21/24 (87%); strand = Plus / Plus

```
Query: 23 ttgcttgattcacttcwwyaagag 46
          ||||||||||||||||   |||||
Sbjct: 11 ttgcttgattcacttctacaagag 34
```

>FK041090|FK041090.1 CF_WO0_90D09_SP6 Copepod Whole Organism, Normalized *Calanus finmarchicus* cDNA clone CF_WO0_90D09; 5', mRNA sequence. Length = 622; score = 40.1 bits (18), expect = 0.83; identities = 21/24 (87%); strand = Plus / Plus

```
Query: 23 ttgcttgattcacttcwwyaagag 46
          ||||||||||||||||   |||||
Sbjct: 12 ttgcttgattcacttcttcaagag 35
```

>FK041072|FK041072.1 CF_WO0_90C01_SP6 Copepod Whole Organism, Normalized *Calanus finmarchicus* cDNA clone CF_WO0_90C01; 5', mRNA sequence. Length = 617; score = 40.1 bits (18), expect = 0.83; identities = 21/24 (87%); strand = Plus / Plus

```
Query: 23 ttgcttgattcacttcwwyaagag 46
          ||||||||||||||||   |||||
Sbjct: 8  ttgcttgattcacttctacaagag 31
```

>FK041068|FK041068.1 CF_WO0_90B09_SP6 Copepod Whole Organism, Normalized *Calanus finmarchicus* cDNA clone CF_WO0_90B09; 5' similar to gb|EDV37881.1| GF11198 - Drosophila ananassae. Score = 82.4 bits (202), expect = 2e-14, m Length = 576; score = 40.1 bits (18), expect = 0.83; identities = 21/24 (87%); strand = Plus / Plus

```
Query: 23 ttgcttgattcacttcwwyaagag 46
          ||||||||||||||||   |||||
Sbjct: 12 ttgcttgattcacttctacaagag 35
```

>FK041036|FK041036.1 CF_WO0_89G06_SP6 Copepod Whole Organism, Normalized *Calanus finmarchicus* cDNA clone CF_WO0_89G06; 5' similar to ref|XP_974174.1| DIVDICTED: similar to AGAP009114-PA - Tribolium castaneum. Score = 131 b Length = 724; score = 40.1 bits (18), expect = 0.83; identities = 21/24 (87%); strand = Plus / Plus

```
Query: 23 ttgcttgattcacttcwwyaagag 46
          ||||||||||||||||   |||||
Sbjct: 8  ttgcttgattcacttctacaagag 31
```

>FK041032|FK041032.1 CF_WO0_89G02_SP6 Copepod Whole Organism, Normalized *Calanus finmarchicus* cDNA clone CF_WO0_89G02; 5' , mRNA sequence. Length = 693; score = 40.1 bits (18), expect = 0.83; identities = 21/24 (87%); strand = Plus / Plus

```
Query: 23 ttgcttgattcacttcwwyaagag 46
          ||||||||||||||||   |||||
Sbjct: 6  ttgcttgattcacttctacaagag 29
```

>FK041026|FK041026.1 CF_WO0_89F04_SP6 Copepod Whole Organism, Normalized *Calanus finmarchicus* cDNA clone CF_WO0_89F04; 5' similar to gb|EDV36395.1| GF11977 - Drosophila ananassae. Score = 181 bits (459), expect = 3e-44, mR Length = 647; score = 40.1 bits (18), expect = 0.83; identities = 21/24 (87%); strand = Plus / Plus

```
Query: 23 ttgcttgattcacttcwwyaagag 46
          ||||||||||||||||   |||||
Sbjct: 14 ttgcttgattcacttctacaagag 37
```

>FK040975|FK040975.1 CF_WO0_88H04_SP6 Copepod Whole Organism, Normalized *Calanus finmarchicus* cDNA clone CF_WO0_88H04; 5' similar to emb|CAG01831.1| unnamed protein product - Tetraodon nigroviridis. Score = 164 bits (414), Length = 610; score = 40.1 bits (18), expect = 0.83; identities = 21/24 (87%); strand = Plus / Plus

```
Query: 23 ttgcttgattcacttcwwyaagag 46
          ||||||||||||||||   |||||
Sbjct: 12 ttgcttgattcacttctacaagag 35
```

>FK040893|FK040893.1 CF_WO0_87H08_SP6 Copepod Whole Organism, Normalized *Calanus finmarchicus* cDNA clone CF_WO0_87H08; 5' similar to ref|XP_001843239.1| dolichyl pyrophosphate Glc1Man9GlcNAc2. Score = 199 bits (506), Expec Length = 683; score = 40.1 bits (18), expect = 0.83; identities = 21/24 (87%); strand = Plus / Plus

```
Query: 23 ttgcttgattcacttcwwyaagag 46
          ||||||||||||||||   |||||
Sbjct: 10 ttgcttgattcacttctacaagag 33
```

>FK040892|FK040892.1 CF_WO0_87H06_SP6 Copepod Whole Organism, Normalized *Calanus finmarchicus* cDNA clone CF_WO0_87H06; 5' similar to ref|XP_001843239.1| dolichyl pyrophosphate Glc1Man9GlcNAc2. Score = 199 bits (506), Expec Length = 660; score = 40.1 bits (18), expect = 0.83; identities = 21/24 (87%); strand = Plus / Plus

Query: 23 ttgcttgattcacttcwwyaagag 46
         ||||||||||||||| |||||
Sbjct: 10 ttgcttgattcacttctacaagag 33

>FK040871|FK040871.1 CF_WO0_87F06_SP6 Copepod Whole Organism, Normalized *Calanus finmarchicus* cDNA clone CF_WO0_87F06; 5' similar to ref|XP_001868452.1| palmitoyltransferase ZDHHC17 - Culex pipiens quinquefasciatus. Score Length = 663; score = 40.1 bits (18), expect = 0.83; identities = 21/24 (87%); strand = Plus / Plus

Query: 23 ttgcttgattcacttcwwyaagag 46
         ||||||||||||||| |||||
Sbjct: 10 ttgcttgattcacttctacaagag 33

>FK040865|FK040865.1 CF_WO0_87E12_SP6 Copepod Whole Organism, Normalized *Calanus finmarchicus* cDNA clone CF_WO0_87E12; 5' similar to gb|EDV53832.1| GG11305 - Drosophila erecta. Score = 258 bits (659), expect = 2e-67, mRNA Length = 661; score = 40.1 bits (18), expect = 0.83; identities = 21/24 (87%); strand = Plus / Plus

Query: 23 ttgcttgattcacttcwwyaagag 46
         ||||||||||||||| |||||
Sbjct: 3 ttgcttgattcacttctacaagag 26

>FK040864|FK040864.1 CF_WO0_87E11_SP6 Copepod Whole Organism, Normalized *Calanus finmarchicus* cDNA clone CF_WO0_87E11; 5', mRNA sequence. Length = 604; score = 40.1 bits (18), expect = 0.83; identities = 21/24 (87%); strand = Plus / Plus

Query: 23 ttgcttgattcacttcwwyaagag 46
         ||||||||||||||| |||||
Sbjct: 12 ttgcttgattcacttctacaagag 35

>FK040839|FK040839.1 CF_WO0_87C08_SP6 Copepod Whole Organism, Normalized *Calanus finmarchicus* cDNA clone CF_WO0_87C08; 5' similar to ref|NP_001089444.1| hypothetical protein LOC734494 - Xenopus laevis. Score = 97.4 bits (2 Length = 616; score = 40.1 bits (18), expect = 0.83; identities = 21/24 (87%); strand = Plus / Plus

Query: 23 ttgcttgattcacttcwwyaagag 46
         ||||||||||||||| |||||
Sbjct: 12 ttgcttgattcacttctacaagag 35

>FG985906|FG985906.1 CF_WO0_86G07_SP6 Copepod Whole Organism, Normalized *Calanus finmarchicus* cDNA clone CF_WO0_86G07; 5', mRNA sequence. Length = 652; score = 40.1 bits (18), expect = 0.83; identities = 21/24 (87%); strand = Plus / Plus

Query: 23 ttgcttgattcacttcwwyaagag 46
         ||||||||||||||| |||||
Sbjct: 43 ttgcttgattcacttctacaagag 66

>FG985899|FG985899.1 CF_WOO_86F12_SP6 Copepod Whole Organism, Normalized *Calanus finmarchicus* cDNA clone CF_WOO_86F12; 5’ , mRNA sequence. Length = 687; score = 40.1 bits (18), expect = 0.83; identities = 21/24 (87%); strand = Plus / Plus

```
Query: 23 ttgcttgattcacttcwwyaagag 46
          ||||||||||||||||   |||||
Sbjct: 12 ttgcttgattcacttctacaagag 35
```

>FG985856|FG985856.1 CF_WOO_86C05_SP6 Copepod Whole Organism, Normalized *Calanus finmarchicus* cDNA clone CF_WOO_86C05; 5’ , mRNA sequence. Length = 709; score = 40.1 bits (18), expect = 0.83; identities = 21/24 (87%); strand = Plus / Plus

```
Query: 23 ttgcttgattcacttcwwyaagag 46
          ||||||||||||||||   |||||
Sbjct: 5  ttgcttgattcacttctacaagag 28
```

>FG985733|FG985733.1 CF_WOO_84F06_SP6 Copepod Whole Organism, Normalized *Calanus finmarchicus* cDNA clone CF_WOO_84F06; 5’ , mRNA sequence. Length = 590; score = 40.1 bits (18), expect = 0.83; identities = 21/24 (87%); strand = Plus / Plus

```
Query: 23 ttgcttgattcacttcwwyaagag 46
          ||||||||||||||||   |||||
Sbjct: 9  ttgcttgattcacttctacaagag 32
```

>FG985727|FG985727.1 CF_WOO_84E11_SP6 Copepod Whole Organism, Normalized *Calanus finmarchicus* cDNA clone CF_WOO_84E11; 5’ , mRNA sequence. Length = 642; score = 40.1 bits (18), expect = 0.83; identities = 21/24 (87%); strand = Plus / Plus

```
Query: 23 ttgcttgattcacttcwwyaagag 46
          ||||||||||||||||   |||||
Sbjct: 10 ttgcttgattcacttcttcaagag 33
```

>FG985721|FG985721.1 CF_WOO_84E04_SP6 Copepod Whole Organism, Normalized *Calanus finmarchicus* cDNA clone CF_WOO_84E04; 5’ similar to ref|XP_001847380.1| trafficking protein particle complex subunit 6B [Culex pipiens. Score Length = 654; score = 40.1 bits (18), expect = 0.83; identities = 21/24 (87%); strand = Plus / Plus

```
Query: 23 ttgcttgattcacttcwwyaagag 46
          ||||||||||||||||   |||||
Sbjct: 14 ttgcttgattcacttctacaagag 37
```

>FG985522|FG985522.1 CF_WOO_81H12_SP6 Copepod Whole Organism, Normalized *Calanus finmarchicus* cDNA clone CF_WOO_81H12; 5’ similar to emb|CAL57442.1| putative t-complex protein 1 theta chain (ISS) - Ostreococcus tauri. Scor Length = 611; score = 40.1 bits (18), expect = 0.83; identities = 21/24 (87%); strand = Plus / Plus

```
Query: 23 ttgcttgattcacttcwwyaagag 46
          ||||||||||||||||   |||||
Sbjct: 10 ttgcttgattcacttctacaagag 33
```

>FG985508|FG985508.1 CF_WO0_81G10_SP6 Copepod Whole Organism, Normalized *Calanus finmarchicus* cDNA clone CF_WO0_81G10; 5' , mRNA sequence. Length = 657; score = 40.1 bits (18), expect = 0.83; identities = 21/24 (87%); strand = Plus / Plus

```
Query: 23 ttgcttgattcacttcwwyaagag 46
          |||||||||||||||   |||||
Sbjct: 15 ttgcttgattcacttcaacaagag 38
```

>FG985493|FG985493.1 CF_WO0_81F07_SP6 Copepod Whole Organism, Normalized *Calanus finmarchicus* cDNA clone CF_WO0_81F07; 5' , mRNA sequence. Length = 645; score = 40.1 bits (18), expect = 0.83; identities = 21/24 (87%); strand = Plus / Plus

```
Query: 23 ttgcttgattcacttcwwyaagag 46
          |||||||||||||||   |||||
Sbjct: 8  ttgcttgattcacttctacaagag 31
```

>FG985475|FG985475.1 CF_WO0_81D11_SP6 Copepod Whole Organism, Normalized *Calanus finmarchicus* cDNA clone CF_WO0_81D11; 5' similar to ref|XP_001660406.1| organic anion transporter - Aedes aegypti. Score = 86.7 bits (213), E. Length = 647; score = 40.1 bits (18), expect = 0.83; identities = 21/24 (87%); strand = Plus / Plus

```
Query: 23 ttgcttgattcacttcwwyaagag 46
          |||||||||||||||   |||||
Sbjct: 9  ttgcttgattcacttctacaagag 32
```

>FG985465|FG985465.1 CF_WO0_81C11_SP6 Copepod Whole Organism, Normalized *Calanus finmarchicus* cDNA clone CF_WO0_81C11; 5' , mRNA sequence. Length = 473; score = 40.1 bits (18), expect = 0.83; identities = 21/24 (87%); strand = Plus / Plus

```
Query: 23 ttgcttgattcacttcwwyaagag 46
          |||||||||||||||   |||||
Sbjct: 8  ttgcttgattcacttctacaagag 31
```

>FG985431|FG985431.1 CF_WO0_80H05_SP6 Copepod Whole Organism, Normalized *Calanus finmarchicus* cDNA clone CF_WO0_80H05; 5' , mRNA sequence. Length = 685; score = 40.1 bits (18), expect = 0.83; identities = 21/24 (87%); strand = Plus / Plus

```
Query: 23 ttgcttgattcacttcwwyaagag 46
          |||||||||||||||   |||||
Sbjct: 10 ttgcttgattcacttctacaagag 33
```

>FG985422|FG985422.1 CF_WO0_80G08_SP6 Copepod Whole Organism, Normalized *Calanus finmarchicus* cDNA clone CF_WO0_80G08; 5' similar to ref|XP_001603179.1| DIVDICTED: similar to AMP dependent coa ligase [Nasonia. Score = 136 Length = 636; score = 40.1 bits (18), expect = 0.83; identities = 21/24 (87%); strand = Plus / Plus

```
Query: 23 ttgcttgattcacttcwwyaagag 46
          |||||||||||||||   |||||
```

Sbjct: 5 ttgcttgattcacttctacaagag 28

>FG985357|FG985357.1 CF_WO0_80B01_SP6 Copepod Whole Organism, Normalized *Calanus finmarchicus* cDNA clone CF_WO0_80B01; 5’ similar to ref|XP_784125.2| DIVDICTED: similar to Protein. Score = 243 bits (619), expect = 8e-63, m Length = 620; score = 40.1 bits (18), expect = 0.83; identities = 21/24 (87%); strand = Plus / Plus

```
Query: 23 ttgcttgattcacttcwwyaagag 46
          ||||||||||||||||   |||||
Sbjct: 1  ttgcttgattcacttctacaagag 24
```

>FG985355|FG985355.1 CF_WO0_80A11_SP6 Copepod Whole Organism, Normalized *Calanus finmarchicus* cDNA clone CF_WO0_80A11; 5’ similar to ref|XP_001676108.1| Hypothetical protein CBG17545 - Caenorhabditis briggsae AF16. Score = Length = 618; score = 40.1 bits (18), expect = 0.83; identities = 21/24 (87%); strand = Plus / Plus

```
Query: 23 ttgcttgattcacttcwwyaagag 46
          ||||||||||||||||   |||||
Sbjct: 5  ttgcttgattcacttctacaagag 28
```

>FG633193|FG633193.1 CF_WO0_78f11_SP6 Copepod Whole Organism, Normalized *Calanus finmarchicus* cDNA clone CF_WO0_78f11; 5’ similar to ref|XP_001604099.1| DIVDICTED: similar to smooth - Nasonia vitripennis. Score = 69.3 bits Length = 539; score = 40.1 bits (18), expect = 0.83; identities = 21/24 (87%); strand = Plus / Plus

```
Query: 23 ttgcttgattcacttcwwyaagag 46
          ||||||||||||||||   |||||
Sbjct: 39 ttgcttgattcacttctacaagag 62
```

>FG633151|FG633151.1 CF_WO0_78b04_SP6 Copepod Whole Organism, Normalized *Calanus finmarchicus* cDNA clone CF_WO0_78b04; 5’ similar to ref|XP_319655.2| AGAP008908-PA - Anopheles gambiae str. PEST. Score = 209 bits (531), Exp Length = 643; score = 40.1 bits (18), expect = 0.83; identities = 21/24 (87%); strand = Plus / Plus

```
Query: 23 ttgcttgattcacttcwwyaagag 46
          ||||||||||||||||   |||||
Sbjct: 9  ttgcttgattcacttctacaagag 32
```

>FG633127|FG633127.1 CF_WO0_77g08_SP6 Copepod Whole Organism, Normalized *Calanus finmarchicus* cDNA clone CF_WO0_77g08; 5’ similar to gb|EDL10915.1| DEAD (Asp-Glu-Ala-Asp) box polypeptide 39, isoform CRA_a [Mus. Score = 275 Length = 605; score = 40.1 bits (18), expect = 0.83; identities = 21/24 (87%); strand = Plus / Plus

```
Query: 23 ttgcttgattcacttcwwyaagag 46
          ||||||||||||||||   |||||
Sbjct: 10 ttgcttgattcacttctacaagag 33
```

>FG633110|FG633110.1 CF_WO0_77f02_SP6 Copepod Whole Organism, Normalized *Calanus finmarchicus* cDNA clone CF_WO0_77f02; 5’ similar to ref|XP_623642.1| DIVDICTED: similar to Tetraspanin

97E CG6323-PA, isoform A [Apis. Score Length = 549; score = 40.1 bits (18), expect = 0.83; identities = 21/24 (87%); strand = Plus / Plus

```
Query: 23 ttgcttgattcacttcwwyaagag 46
          ||||||||||||||||   |||||
Sbjct: 3  ttgcttgattcacttctacaagag 26
```

>FG633073|FG633073.1 CF_WO0_77b05_SP6 Copepod Whole Organism, Normalized *Calanus finmarchicus* cDNA clone CF_WO0_77b05; 5' similar to gb|EDM03508.1| rCG61685 - Rattus norvegicus. Score = 110 bits (276), expect = 3e-23-mRNA Length = 471; score = 40.1 bits (18), expect = 0.83; identities = 21/24 (87%); strand = Plus / Plus

```
Query: 23 ttgcttgattcacttcwwyaagag 46
          ||||||||||||||||   |||||
Sbjct: 8  ttgcttgattcacttctacaagag 31
```

>FG633031|FG633031.1 CF_WO0_76f07_SP6 Copepod Whole Organism, Normalized *Calanus finmarchicus* cDNA clone CF_WO0_76f07; 5' similar to ref|XP_001671730.1| Hypothetical protein CBG03527 - Caenorhabditis briggsae AF16. Score = Length = 624; score = 40.1 bits (18), expect = 0.83; identities = 21/24 (87%); strand = Plus / Plus

```
Query: 23 ttgcttgattcacttcwwyaagag 46
          ||||||||||||||||   |||||
Sbjct: 9  ttgcttgattcacttctacaagag 32
```

>FG633027|FG633027.1 CF_WO0_76f02_SP6 Copepod Whole Organism, Normalized *Calanus finmarchicus* cDNA clone CF_WO0_76f02; 5' similar to ref|XP_001671730.1| Hypothetical protein CBG03527 - Caenorhabditis briggsae AF16. Score = Length = 510; score = 40.1 bits (18), expect = 0.83; identities = 21/24 (87%); strand = Plus / Plus

```
Query: 23 ttgcttgattcacttcwwyaagag 46
          ||||||||||||||||   |||||
Sbjct: 9  ttgcttgattcacttctacaagag 32
```

>FG632938|FG632938.1 CF_WO0_75f05_SP6 Copepod Whole Organism, Normalized *Calanus finmarchicus* cDNA clone CF_WO0_75f05; 5', mRNA sequence. Length = 668; score = 40.1 bits (18), expect = 0.83; identities = 21/24 (87%); strand = Plus / Plus

```
Query: 23 ttgcttgattcacttcwwyaagag 46
          ||||||||||||||||   |||||
Sbjct: 7  ttgcttgattcacttctacaagag 30
```

>FG632926|FG632926.1 CF_WO0_75e05_SP6 Copepod Whole Organism, Normalized *Calanus finmarchicus* cDNA clone CF_WO0_75e05; 5' similar to ref|XP_001639131.1| predicted protein - Nematostella vectensis. Score = 300 bits (768), E. Length = 687; score = 40.1 bits (18), expect = 0.83; identities = 21/24 (87%); strand = Plus / Plus

```
Query: 23 ttgcttgattcacttcwwyaagag 46
          ||||||||||||||||   |||||
```

Sbjct: 2 ttgcttgattcacttctacaagag 25

>FG632913|FG632913.1 CF_WO0_75d02_SP6 Copepod Whole Organism, Normalized *Calanus finmarchicus* cDNA clone CF_WO0_75d02; 5’ similar to ref|NP_001120938.1| si:ch211-239d11.1 - Danio rerio. Score = 213 bits (543), expect = 6e- Length = 626; score = 40.1 bits (18), expect = 0.83; identities = 21/24 (87%); strand = Plus / Plus

```
Query: 23 ttgcttgattcacttcwwyaagag 46
          ||||||||||||||||   |||||
Sbjct: 12 ttgcttgattcacttctataagag 35
```

>FG632910|FG632910.1 CF_WO0_75c11_SP6 Copepod Whole Organism, Normalized *Calanus finmarchicus* cDNA clone CF_WO0_75c11; 5’ similar to ref|NP_001037203.1| innexin 2 - Bombyx mori. Score = 75.1 bits (183), expect = 3e-12, mRN Length = 637; score = 40.1 bits (18), expect = 0.83; identities = 21/24 (87%); strand = Plus / Plus

```
Query: 23 ttgcttgattcacttcwwyaagag 46
          ||||||||||||||||   |||||
Sbjct: 13 ttgcttgattcacttctacaagag 36
```

>FG632888|FG632888.1 CF_WO0_75a11_SP6 Copepod Whole Organism, Normalized *Calanus finmarchicus* cDNA clone CF_WO0_75a11; 5’ similar to ref|YP_721977.1| Methyltransferase type 11 - Trichodesmium erythraeum IMS101. Score = 102 Length = 680; score = 40.1 bits (18), expect = 0.83; identities = 21/24 (87%); strand = Plus / Plus

```
Query: 23 ttgcttgattcacttcwwyaagag 46
          ||||||||||||||||   |||||
Sbjct: 7  ttgcttgattcacttctacaagag 30
```

>FG632804|FG632804.1 CF_WO0_74b05_SP6 Copepod Whole Organism, Normalized *Calanus finmarchicus* cDNA clone CF_WO0_74b05; 5’ similar to ref|NP_649340.1| CG11306 CG11306-PA - Drosophila melanogaster. Score = 220 bits (560), Ex Length = 650; score = 40.1 bits (18), expect = 0.83; identities = 21/24 (87%); strand = Plus / Plus

```
Query: 23 ttgcttgattcacttcwwyaagag 46
          ||||||||||||||||   |||||
Sbjct: 1  ttgcttgattcacttctacaagag 24
```

>FG632694|FG632694.1 CF_WO0_73a01_SP6 Copepod Whole Organism, Normalized *Calanus finmarchicus* cDNA clone CF_WO0_73a01; 5’ similar to ref|XP_001603179.1| DIVDICTED: similar to AMP dependent coa ligase [Nasonia. Score = 134 Length = 688; score = 40.1 bits (18), expect = 0.83; identities = 21/24 (87%); strand = Plus / Plus

```
Query: 23 ttgcttgattcacttcwwyaagag 46
          ||||||||||||||||   |||||
Sbjct: 6  ttgcttgattcacttctacaagag 29
```

>FG632662|FG632662.1 CF_WO0_72f03_SP6 Copepod Whole Organism, Normalized *Calanus finmarchicus* cDNA clone CF_WO0_72f03; 5’ similar to ref|XP_001870703.1| T-complex protein 1 subunit gamma

- Culex pipiens quinquefasciatus. S Length = 659; score = 40.1 bits (18), expect = 0.83; identities = 21/24 (87%); strand = Plus / Plus

```
Query: 23 ttgcttgattcacttcwwyaagag 46
          |||||||||||||||   |||||
Sbjct: 9  ttgcttgattcacttctacaagag 32
```

>FG632582|FG632582.1 CF_WO0_71f12_SP6 Copepod Whole Organism, Normalized *Calanus finmarchicus* cDNA clone CF_WO0_71f12; 5' , mRNA sequence. Length = 682; score = 40.1 bits (18), expect = 0.83; identities = 21/24 (87%); strand = Plus / Plus

```
Query: 23 ttgcttgattcacttcwwyaagag 46
          |||||||||||||||   |||||
Sbjct: 27 ttgcttgattcacttctacaagag 50
```

>FG632576|FG632576.1 CF_WO0_71f06_SP6 Copepod Whole Organism, Normalized *Calanus finmarchicus* cDNA clone CF_WO0_71f06; 5' similar to ref|NP_001079462.1| similar to RNA, U transporter 1 - Xenopus laevis. Score = 103 bits (2 Length = 678; score = 40.1 bits (18), expect = 0.83; identities = 21/24 (87%); strand = Plus / Plus

```
Query: 23 ttgcttgattcacttcwwyaagag 46
          |||||||||||||||   |||||
Sbjct: 8  ttgcttgattcacttctacaagag 31
```

>FG343072|FG343072.1 CF_WO0_70h04_SP6 Copepod Whole Organism, Normalized *Calanus finmarchicus* cDNA clone CF_WO0_70h04; 5' similar to ref|XP_966512.1| DIVDICTED: similar to CG1404-PA, isoform A isoform 1 [Tribolium. Score = Length = 644; score = 40.1 bits (18), expect = 0.83; identities = 21/24 (87%); strand = Plus / Plus

```
Query: 23 ttgcttgattcacttcwwyaagag 46
          |||||||||||||||   |||||
Sbjct: 14 ttgcttgattcacttctacaagag 37
```

>FG343057|FG343057.1 CF_WO0_70f12_SP6 Copepod Whole Organism, Normalized *Calanus finmarchicus* cDNA clone CF_WO0_70f12; 5' similar to ref|NP_649145.1| CG8743 CG8743-PA - Drosophila melanogaster. Score = 81.3 bits (199), Exp Length = 650; score = 40.1 bits (18), expect = 0.83; identities = 21/24 (87%); strand = Plus / Plus

```
Query: 23 ttgcttgattcacttcwwyaagag 46
          |||||||||||||||   |||||
Sbjct: 5  ttgcttgattcacttctacaagag 28
```

>FG343052|FG343052.1 CF_WO0_70f06_SP6 Copepod Whole Organism, Normalized *Calanus finmarchicus* cDNA clone CF_WO0_70f06; 5' similar to sp|Q8AY35|SC61A_NOTAN Protein transport protein Sec61 subunit alpha. Score = 352 bits (90 Length = 630; score = 40.1 bits (18), expect = 0.83; identities = 21/24 (87%); strand = Plus / Plus

```
Query: 23 ttgcttgattcacttcwwyaagag 46
          |||||||||||||||   |||||
```

Sbjct: 8 ttgcttgattcacttctacaagag 31

>FG343033|FG343033.1 CF_WO0_70d11_SP6 Copepod Whole Organism, Normalized *Calanus finmarchicus* cDNA clone CF_WO0_70d11; 5' , mRNA sequence. Length = 689; score = 40.1 bits (18), expect = 0.83; identities = 21/24 (87%); strand = Plus / Plus

```
Query: 23 ttgcttgattcacttcwwyaagag 46
          |||||||||||||||   |||||
Sbjct: 10 ttgcttgattcacttctacaagag 33
```

>FG343029|FG343029.1 CF_WO0_70d07_SP6 Copepod Whole Organism, Normalized *Calanus finmarchicus* cDNA clone CF_WO0_70d07; 5' similar to ref|XP_001641680.1| predicted protein - Nematostella vectensis. Score = 129 bits (323), E. Length = 706; score = 40.1 bits (18), expect = 0.83; identities = 21/24 (87%); strand = Plus / Plus

```
Query: 23 ttgcttgattcacttcwwyaagag 46
          |||||||||||||||   |||||
Sbjct: 9  ttgcttgattcacttctacaagag 32
```

>FG343022|FG343022.1 CF_WO0_70c12_SP6 Copepod Whole Organism, Normalized *Calanus finmarchicus* cDNA clone CF_WO0_70c12; 5' similar to gb|AAI42562.1| LOC100101293 protein - Xenopus laevis. Score = 114 bits (284), expect = 7e Length = 703; score = 40.1 bits (18), expect = 0.83; identities = 21/24 (87%); strand = Plus / Plus

```
Query: 23 ttgcttgattcacttcwwyaagag 46
          |||||||||||||||   |||||
Sbjct: 7  ttgcttgattcacttctacaagag 30
```

>FG343015|FG343015.1 CF_WO0_70c04_SP6 Copepod Whole Organism, Normalized *Calanus finmarchicus* cDNA clone CF_WO0_70c04; 5' , mRNA sequence. Length = 635; score = 40.1 bits (18), expect = 0.83; identities = 21/24 (87%); strand = Plus / Plus

```
Query: 23 ttgcttgattcacttcwwyaagag 46
          |||||||||||||||   |||||
Sbjct: 5  ttgcttgattcacttctacaagag 28
```

>FG343010|FG343010.1 CF_WO0_70b11_SP6 Copepod Whole Organism, Normalized *Calanus finmarchicus* cDNA clone CF_WO0_70b11; 5' similar to gb|AAI42562.1| LOC100101293 protein - Xenopus laevis. Score = 114 bits (284), expect = 7e Length = 705; score = 40.1 bits (18), expect = 0.83; identities = 21/24 (87%); strand = Plus / Plus

```
Query: 23 ttgcttgattcacttcwwyaagag 46
          |||||||||||||||   |||||
Sbjct: 9  ttgcttgattcacttctacaagag 32
```

>FG342976|FG342976.1 CF_WO0_69h01_SP6 Copepod Whole Organism, Normalized *Calanus finmarchicus* cDNA clone CF_WO0_69h01; 5' similar to ref|XP_624161.1| DIVDICTED: similar to uninitiated CG4008-PA - Apis mellifera. Score = 16 Length = 628; score = 40.1 bits (18), expect = 0.83; identities = 21/24 (87%); strand = Plus / Plus

Query: 23 ttgcttgattcacttcwwyaagag 46
         ||||||||||||||| |||||
Sbjct: 13 ttgcttgattcacttctacaagag 36

>FG342866|FG342866.1 CF_WO0_68f06_SP6 Copepod Whole Organism, Normalized *Calanus finmarchicus* cDNA clone CF_WO0_68f06; 5' similar to ref|NP_001082065.1| reptin - Xenopus laevis. Score = 273 bits (697), expect = 9e-72, mRNA Length = 687; score = 40.1 bits (18), expect = 0.83; identities = 21/24 (87%); strand = Plus / Plus

Query: 23 ttgcttgattcacttcwwyaagag 46
         ||||||||||||||| |||||
Sbjct: 6 ttgcttgattcacttctacaagag 29

>FG342865|FG342865.1 CF_WO0_68f05_SP6 Copepod Whole Organism, Normalized *Calanus finmarchicus* cDNA clone CF_WO0_68f05; 5' similar to ref|NP_001082065.1| reptin - Xenopus laevis. Score = 305 bits (780), expect = 2e-81, mRNA Length = 690; score = 40.1 bits (18), expect = 0.83; identities = 21/24 (87%); strand = Plus / Plus

Query: 23 ttgcttgattcacttcwwyaagag 46
         ||||||||||||||| |||||
Sbjct: 6 ttgcttgattcacttctacaagag 29

>FG342812|FG342812.1 CF_WO0_68a10_SP6 Copepod Whole Organism, Normalized *Calanus finmarchicus* cDNA clone CF_WO0_68a10; 5' , mRNA sequence. Length = 651; score = 40.1 bits (18), expect = 0.83; identities = 21/24 (87%); strand = Plus / Plus

Query: 23 ttgcttgattcacttcwwyaagag 46
         ||||||||||||||| |||||
Sbjct: 15 ttgcttgattcacttctacaagag 38

>FG342809|FG342809.1 CF_WO0_68a07_SP6 Copepod Whole Organism, Normalized *Calanus finmarchicus* cDNA clone CF_WO0_68a07; 5' , mRNA sequence. Length = 652; score = 40.1 bits (18), expect = 0.83; identities = 21/24 (87%); strand = Plus / Plus

Query: 23 ttgcttgattcacttcwwyaagag 46
         ||||||||||||||| |||||
Sbjct: 15 ttgcttgattcacttctacaagag 38

>FG342786|FG342786.1 CF_WO0_67g06_SP6 Copepod Whole Organism, Normalized *Calanus finmarchicus* cDNA clone CF_WO0_67g06; 5' , mRNA sequence. Length = 611; score = 40.1 bits (18), expect = 0.83; identities = 21/24 (87%); strand = Plus / Plus

Query: 23 ttgcttgattcacttcwwyaagag 46
         ||||||||||||||| |||||
Sbjct: 10 ttgcttgattcacttctacaagag 33

>FG342785|FG342785.1 CF_WO0_67g05_SP6 Copepod Whole Organism, Normalized *Calanus finmarchicus* cDNA clone CF_WO0_67g05; 5' similar to ref|XP_788815.2| DIVDICTED: hypothetical protein -

Strongylocentrotus purpuratus. Score = Length = 692; score = 40.1 bits (18), expect = 0.83; identities = 21/24 (87%); strand = Plus / Plus

```
Query: 23 ttgcttgattcacttcwwyaagag 46
          ||||||||||||||||   |||||
Sbjct: 8  ttgcttgattcacttctacaagag 31
```

>FG342783|FG342783.1 CF_WO0_67g03_SP6 Copepod Whole Organism, Normalized *Calanus finmarchicus* cDNA clone CF_WO0_67g03; 5' similar to ref|XP_001623655.1| predicted protein - Nematostella vectensis. Score = 123 bits (308), E. Length = 490; score = 40.1 bits (18), expect = 0.83; identities = 21/24 (87%); strand = Plus / Plus

```
Query: 23 ttgcttgattcacttcwwyaagag 46
          ||||||||||||||||   |||||
Sbjct: 9  ttgcttgattcacttctacaagag 32
```

>FG342762|FG342762.1 CF_WO0_67e06_SP6 Copepod Whole Organism, Normalized *Calanus finmarchicus* cDNA clone CF_WO0_67e06; 5' , mRNA sequence. Length = 588; score = 40.1 bits (18), expect = 0.83; identities = 21/24 (87%); strand = Plus / Plus

```
Query: 23 ttgcttgattcacttcwwyaagag 46
          ||||||||||||||||   |||||
Sbjct: 9  ttgcttgattcacttctacaagag 32
```

>FG342727|FG342727.1 CF_WO0_67b04_SP6 Copepod Whole Organism, Normalized *Calanus finmarchicus* cDNA clone CF_WO0_67b04; 5' similar to ref|XP_001123261.1| DIVDICTED: similar to CG6070-PA, partial - Apis mellifera. Score = 19 Length = 654; score = 40.1 bits (18), expect = 0.83; identities = 21/24 (87%); strand = Plus / Plus

```
Query: 23 ttgcttgattcacttcwwyaagag 46
          ||||||||||||||||   |||||
Sbjct: 14 ttgcttgattcacttctacaagag 37
```

>FG342703|FG342703.1 CF_WO0_66g12_SP6 Copepod Whole Organism, Normalized *Calanus finmarchicus* cDNA clone CF_WO0_66g12; 5' , mRNA sequence. Length = 616; score = 40.1 bits (18), expect = 0.83; identities = 21/24 (87%); strand = Plus / Plus

```
Query: 23 ttgcttgattcacttcwwyaagag 46
          ||||||||||||||||   |||||
Sbjct: 7  ttgcttgattcacttctacaagag 30
```

>FG342648|FG342648.1 CF_WO0_66c02_SP6 Copepod Whole Organism, Normalized *Calanus finmarchicus* cDNA clone CF_WO0_66c02; 5' similar to ref|XP_780162.1| DIVDICTED: similar to MGC143224 protein [Strongylocentrotus. Score = 190 Length = 613; score = 40.1 bits (18), expect = 0.83; identities = 21/24 (87%); strand = Plus / Plus

```
Query: 23 ttgcttgattcacttcwwyaagag 46
          ||||||||||||||||   |||||
Sbjct: 3  ttgcttgattcacttctacaagag 26
```

>FG342602|FG342602.1 CF_WO0_65g03_SP6 Copepod Whole Organism, Normalized *Calanus finmarchicus* cDNA clone CF_WO0_65g03; 5' similar to emb|CAF90654.1| unnamed protein product - Tetraodon nigroviridis. Score = 129 bits (323), Length = 666; score = 40.1 bits (18), expect = 0.83; identities = 21/24 (87%); strand = Plus / Plus

```
Query: 23 ttgcttgattcacttcwwyaagag 46
          ||||||||||||||||   |||||
Sbjct: 2  ttgcttgattcacttctacaagag 25
```

>FG342599|FG342599.1 CF_WO0_65f12_SP6 Copepod Whole Organism, Normalized *Calanus finmarchicus* cDNA clone CF_WO0_65f12; 5' similar to ref|NP_001095404.1| hypothetical protein LOC510841 - Bos taurus. Score = 174 bits (441), Length = 681; score = 40.1 bits (18), expect = 0.83; identities = 21/24 (87%); strand = Plus / Plus

```
Query: 23 ttgcttgattcacttcwwyaagag 46
          ||||||||||||||||   |||||
Sbjct: 15 ttgcttgattcacttctacaagag 38
```

>FG342576|FG342576.1 CF_WO0_65d12_SP6 Copepod Whole Organism, Normalized *Calanus finmarchicus* cDNA clone CF_WO0_65d12; 5' similar to ref|XP_968492.1| DIVDICTED: similar to CG7340-PB, isoform B - Tribolium castaneum. Score Length = 656; score = 40.1 bits (18), expect = 0.83; identities = 21/24 (87%); strand = Plus / Plus

```
Query: 23 ttgcttgattcacttcwwyaagag 46
          ||||||||||||||||   |||||
Sbjct: 11 ttgcttgattcacttctacaagag 34
```

>FG342558|FG342558.1 CF_WO0_65c05_SP6 Copepod Whole Organism, Normalized *Calanus finmarchicus* cDNA clone CF_WO0_65c05; 5' similar to ref|XP_623063.1| DIVDICTED: similar to Transcription initiation factor IIA gamma. Score = Length = 642; score = 40.1 bits (18), expect = 0.83; identities = 21/24 (87%); strand = Plus / Plus

```
Query: 23 ttgcttgattcacttcwwyaagag 46
          ||||||||||||||||   |||||
Sbjct: 10 ttgcttgattcacttctacaagag 33
```

>FG342547|FG342547.1 CF_WO0_65b06_SP6 Copepod Whole Organism, Normalized *Calanus finmarchicus* cDNA clone CF_WO0_65b06; 5' similar to ref|XP_001660406.1| organic anion transporter - Aedes aegypti. Score = 87.0 bits (214), E. Length = 682; score = 40.1 bits (18), expect = 0.83; identities = 21/24 (87%); strand = Plus / Plus

```
Query: 23 ttgcttgattcacttcwwyaagag 46
          ||||||||||||||||   |||||
Sbjct: 5  ttgcttgattcacttctacaagag 28
```

>FG342501|FG342501.1 CF_WO0_64f01_SP6 Copepod Whole Organism, Normalized *Calanus finmarchicus* cDNA clone CF_WO0_64f01; 5', mRNA sequence. Length = 628; score = 40.1 bits (18), expect = 0.83; identities = 21/24 (87%); strand = Plus / Plus

```
Query: 23 ttgcttgattcacttcwwyaagag 46
          ||||||||||||||||   |||||
Sbjct: 8  ttgcttgattcacttctacaagag 31
```

>FG342433|FG342433.1 CF_WO0_63g05_SP6 Copepod Whole Organism, Normalized *Calanus finmarchicus* cDNA clone CF_WO0_63g05; 5’ similar to sp|Q9XYN1|INX2_SCHAM Innexin inx2 (Innexin-2) (G-Inx2). Score = 156 bits (394), expect = Length = 586; score = 40.1 bits (18), expect = 0.83; identities = 21/24 (87%); strand = Plus / Plus

```
Query: 23 ttgcttgattcacttcwwyaagag 46
          ||||||||||||||||   |||||
Sbjct: 9  ttgcttgattcacttctacaagag 32
```

>FG342429|FG342429.1 CF_WO0_63g01_SP6 Copepod Whole Organism, Normalized *Calanus finmarchicus* cDNA clone CF_WO0_63g01; 5’ , mRNA sequence. Length = 704; score = 40.1 bits (18), expect = 0.83; identities = 21/24 (87%); strand = Plus / Plus

```
Query: 23 ttgcttgattcacttcwwyaagag 46
          ||||||||||||||||   |||||
Sbjct: 10 ttgcttgattcacttctacaagag 33
```

>FG342412|FG342412.1 CF_WO0_63e07_SP6 Copepod Whole Organism, Normalized *Calanus finmarchicus* cDNA clone CF_WO0_63e07; 5’ similar to ref|XP_796182.1| DIVDICTED: hypothetical protein - Strongylocentrotus purpuratus. Score = Length = 661; score = 40.1 bits (18), expect = 0.83; identities = 21/24 (87%); strand = Plus / Plus

```
Query: 23 ttgcttgattcacttcwwyaagag 46
          ||||||||||||||||   |||||
Sbjct: 8  ttgcttgattcacttctacaagag 31
```

>FG342400|FG342400.1 CF_WO0_63d06_SP6 Copepod Whole Organism, Normalized *Calanus finmarchicus* cDNA clone CF_WO0_63d06; 5’ similar to ref|XP_972925.1| DIVDICTED: similar to CG4703-PA - Tribolium castaneum. Score = 221 bits Length = 686; score = 40.1 bits (18), expect = 0.83; identities = 21/24 (87%); strand = Plus / Plus

```
Query: 23 ttgcttgattcacttcwwyaagag 46
          ||||||||||||||||   |||||
Sbjct: 7  ttgcttgattcacttctacaagag 30
```

>FG342385|FG342385.1 CF_WO0_63c01_SP6 Copepod Whole Organism, Normalized *Calanus finmarchicus* cDNA clone CF_WO0_63c01; 5’ similar to ref|XP_969056.1| DIVDICTED: similar to ubiquitin specific protease 14 (predicted). Score Length = 663; score = 40.1 bits (18), expect = 0.83; identities = 21/24 (87%); strand = Plus / Plus

```
Query: 23 ttgcttgattcacttcwwyaagag 46
          ||||||||||||||||   |||||
Sbjct: 8  ttgcttgattcacttctacaagag 31
```

>FG342363|FG342363.1 CF_WO0_63a02_SP6 Copepod Whole Organism, Normalized *Calanus finmarchicus* cDNA clone CF_WO0_63a02; 5' similar to ref|XP_001607506.1| DIVDICTED: hypothetical protein - Nasonia vitripennis. Score = 162 bi Length = 650; score = 40.1 bits (18), expect = 0.83; identities = 21/24 (87%); strand = Plus / Plus

```
Query: 23 ttgcttgattcacttcwwyaagag 46
          ||||||||||||||||   |||||
Sbjct: 8  ttgcttgattcacttctacaagag 31
```

>FG342336|FG342336.1 CF_WO0_62f11_SP6 Copepod Whole Organism, Normalized *Calanus finmarchicus* cDNA clone CF_WO0_62f11; 5' similar to ref|XP_966498.1| DIVDICTED: similar to Vesicle-associated membrane. Score = 204 bits (519 Length = 644; score = 40.1 bits (18), expect = 0.83; identities = 21/24 (87%); strand = Plus / Plus

```
Query: 23 ttgcttgattcacttcwwyaagag 46
          ||||||||||||||||   |||||
Sbjct: 15 ttgcttgattcacttctacaagag 38
```

>FG342305|FG342305.1 CF_WO0_62d02_SP6 Copepod Whole Organism, Normalized *Calanus finmarchicus* cDNA clone CF_WO0_62d02; 5' similar to sp|P0C1J6|FKBP4_RHIOR FK506-binding protein 4 (Peptidyl-prolyl cis-trans isomerase). Scor Length = 711; score = 40.1 bits (18), expect = 0.83; identities = 21/24 (87%); strand = Plus / Plus

```
Query: 23 ttgcttgattcacttcwwyaagag 46
          ||||||||||||||||   |||||
Sbjct: 10 ttgcttgattcacttctataagag 33
```

>FG342250|FG342250.1 CF_WO0_61g06_SP6 Copepod Whole Organism, Normalized *Calanus finmarchicus* cDNA clone CF_WO0_61g06; 5' similar to ref|XP_394284.2| DIVDICTED: similar to Ubiquitin ligase SIAH1 (Seven in absentia. Score = Length = 623; score = 40.1 bits (18), expect = 0.83; identities = 21/24 (87%); strand = Plus / Plus

```
Query: 23 ttgcttgattcacttcwwyaagag 46
          ||||||||||||||||   |||||
Sbjct: 15 ttgcttgattcacttctacaagag 38
```

>FG342244|FG342244.1 CF_WO0_61f12_SP6 Copepod Whole Organism, Normalized *Calanus finmarchicus* cDNA clone CF_WO0_61f12; 5' similar to ref|XP_001664137.1| chromatin assembly factor i P60 subunit - Aedes aegypti. Score = 201 Length = 653; score = 40.1 bits (18), expect = 0.83; identities = 21/24 (87%); strand = Plus / Plus

```
Query: 23 ttgcttgattcacttcwwyaagag 46
          ||||||||||||||||   |||||
Sbjct: 14 ttgcttgattcacttctacaagag 37
```

>FG342237|FG342237.1 CF_WO0_61f05_SP6 Copepod Whole Organism, Normalized *Calanus finmarchicus* cDNA clone CF_WO0_61f05; 5' similar to ref|XP_001631605.1| predicted protein - Nematostella vectensis. Score = 193 bits (491), E. Length = 676; score = 40.1 bits (18), expect = 0.83; identities = 21/24 (87%); strand = Plus / Plus

```
Query: 23 ttgcttgattcacttcwwyaagag 46
          ||||||||||||||||   |||||
Sbjct: 9  ttgcttgattcacttctacaagag 32
```

>FG342208|FG342208.1 CF_WOO_61c12_SP6 Copepod Whole Organism, Normalized *Calanus finmarchicus* cDNA clone CF_WOO_61c12; 5' , mRNA sequence. Length = 634; score = 40.1 bits (18), expect = 0.83; identities = 21/24 (87%); strand = Plus / Plus

```
Query: 23 ttgcttgattcacttcwwyaagag 46
          ||||||||||||||||   |||||
Sbjct: 8  ttgcttgattcacttctacaagag 31
```

>FG342182|FG342182.1 CF_WOO_61a10_SP6 Copepod Whole Organism, Normalized *Calanus finmarchicus* cDNA clone CF_WOO_61a10; 5' similar to ref|XP_001605843.1| DIVDICTED: similar to LD23157p - Nasonia vitripennis. Score = 348 bit Length = 665; score = 40.1 bits (18), expect = 0.83; identities = 21/24 (87%); strand = Plus / Plus

```
Query: 23 ttgcttgattcacttcwwyaagag 46
          ||||||||||||||||   |||||
Sbjct: 26 ttgcttgattcacttctacaagag 49
```

>FG342177|FG342177.1 CF_WOO_61a05_SP6 Copepod Whole Organism, Normalized *Calanus finmarchicus* cDNA clone CF_WOO_61a05; 5' , mRNA sequence. Length = 643; score = 40.1 bits (18), expect = 0.83; identities = 21/24 (87%); strand = Plus / Plus

```
Query: 23 ttgcttgattcacttcwwyaagag 46
          ||||||||||||||||   |||||
Sbjct: 7  ttgcttgattcacttctacaagag 30
```

>FG342174|FG342174.1 CF_WOO_61a02_SP6 Copepod Whole Organism, Normalized *Calanus finmarchicus* cDNA clone CF_WOO_61a02; 5' , mRNA sequence. Length = 628; score = 40.1 bits (18), expect = 0.83; identities = 21/24 (87%); strand = Plus / Plus

```
Query: 23 ttgcttgattcacttcwwyaagag 46
          ||||||||||||||||   |||||
Sbjct: 8  ttgcttgattcacttctacaagag 31
```

>FE596692|FE596692.1 CAXG9737.fwd Amphioxus Branchiostoma floridae unpublished cDNA library CAXG, larva whole animal Branchiostoma floridae cDNA clone CAXG9737 5', mRNA sequence. Length = 834; score = 40.1 bits (18), expect = 0.83; identities = 21/24 (87%); strand = Plus / Plus

```
Query: 23 ttgcttgattcacttcwwyaagag 46
          ||||||||||||||||   |||||
Sbjct: 5  ttgcttgattcacttctacaagag 28
```

>FE591355|FE591355.1 CAXG5584.fwd Amphioxus Branchiostoma floridae unpublished cDNA library CAXG, larva whole animal Branchiostoma floridae cDNA clone CAXG5584 5', mRNA sequence. Length = 723; score = 40.1 bits (18), expect = 0.83; identities = 21/24 (87%); strand = Plus / Plus

Query: 23 ttgcttgattcacttcwwyaagag 46
         ||||||||||||||| |||||
Sbjct: 7 ttgcttgattcacttctacaagag 30

>FE573549|FE573549.1 CAXF7035.fwd Amphioxus Branchiostoma floridae unpublished cDNA library CAXF, gastrula whole animal Branchiostoma floridae cDNA clone CAXF7035 5', mRNA sequence. Length = 792; score = 40.1 bits (18), expect = 0.83; identities = 21/24 (87%); strand = Plus / Plus

Query: 23 ttgcttgattcacttcwwyaagag 46
         ||||||||||||||| |||||
Sbjct: 5 ttgcttgattcacttctacaagag 28

>FE568647|FE568647.1 CAXF17113.fwd Amphioxus Branchiostoma floridae unpublished cDNA library CAXF, gastrula whole animal Branchiostoma floridae cDNA clone CAXF17113 5'-mRNA sequence. Length = 763; score = 40.1 bits (18), expect = 0.83; identities = 21/24 (87%); strand = Plus / Plus

Query: 23 ttgcttgattcacttcwwyaagag 46
         ||||||||||||||| |||||
Sbjct: 5 ttgcttgattcacttctacaagag 28

>FE565967|FE565967.1 CAXF14625.fwd Amphioxus Branchiostoma floridae unpublished cDNA library CAXF, gastrula whole animal Branchiostoma floridae cDNA clone CAXF14625 5'-mRNA sequence. Length = 435; score = 40.1 bits (18), expect = 0.83; identities = 21/24 (87%); strand = Plus / Plus

Query: 23 ttgcttgattcacttcwwyaagag 46
         ||||||||||||||| |||||
Sbjct: 7 ttgcttgattcacttctacaagag 30

>FE563520|FE563520.1 CAXF12045.fwd Amphioxus Branchiostoma floridae unpublished cDNA library CAXF, gastrula whole animal Branchiostoma floridae cDNA clone CAXF12045 5'-mRNA sequence. Length = 711; score = 40.1 bits (18), expect = 0.83; identities = 21/24 (87%); strand = Plus / Plus

Query: 23 ttgcttgattcacttcwwyaagag 46
         ||||||||||||||| |||||
Sbjct: 5 ttgcttgattcacttctacaagag 28

>FE562105|FE562105.1 CAXF10707.fwd Amphioxus Branchiostoma floridae unpublished cDNA library CAXF, gastrula whole animal Branchiostoma floridae cDNA clone CAXF10707 5'-mRNA sequence. Length = 768; score = 40.1 bits (18), expect = 0.83; identities = 21/24 (87%); strand = Plus / Plus

Query: 23 ttgcttgattcacttcwwyaagag 46
         ||||||||||||||| |||||
Sbjct: 7 ttgcttgattcacttctacaagag 30

>FE546172|FE546172.1 CAXC14451.fwd Amphioxus Branchiostoma floridae unpublished cDNA library CAXC, neurula whole animal Branchiostoma floridae cDNA clone CAXC14451 5'-mRNA sequence. Length = 816; score = 40.1 bits (18), expect = 0.83; identities = 21/24 (87%); strand = Plus / Plus

```
Query: 23 ttgcttgattcacttcwwyaagag 46
          ||||||||||||||||   |||||
Sbjct: 7  ttgcttgattcacttctacaagag 30
```

>ES414815|ES414815.1 53A_E09 Calanomics library 1 *Calanus finmarchicus* cDNA clone Copepod-Subtr_53A_E09 similar to ubiquinol-cytochrome c reductase, mRNA sequence. Length = 482; score = 40.1 bits (18), expect = 0.83; identities = 21/24 (87%); strand = Plus / Plus

```
Query: 23 ttgcttgattcacttcwwyaagag 46
          ||||||||||||||||   |||||
Sbjct: 8  ttgcttgattcacttctacaagag 31
```

>ES237867|ES237867.1 CF_WO0_60h04_SP6 Copepod Whole Organism, Normalized *Calanus finmarchicus* cDNA clone CF_WO0_60h04; 5' , mRNA sequence. Length = 698; score = 40.1 bits (18), expect = 0.83; identities = 21/24 (87%); strand = Plus / Plus

```
Query: 23 ttgcttgattcacttcwwyaagag 46
          ||||||||||||||||   |||||
Sbjct: 9  ttgcttgattcacttctacaagag 32
```

>ES237829|ES237829.1 CF_WO0_60e02_SP6 Copepod Whole Organism, Normalized *Calanus finmarchicus* cDNA clone CF_WO0_60e02; 5' , mRNA sequence. Length = 643; score = 40.1 bits (18), expect = 0.83; identities = 21/24 (87%); strand = Plus / Plus

```
Query: 23 ttgcttgattcacttcwwyaagag 46
          ||||||||||||||||   |||||
Sbjct: 7  ttgcttgattcacttctacaagag 30
```

>ES237826|ES237826.1 CF_WO0_60d10_SP6 Copepod Whole Organism, Normalized *Calanus finmarchicus* cDNA clone CF_WO0_60d10; 5' similar to dbj|BAA07705.1| Dsrc41 - Drosophila melanogaster. Score = 98.2 bits (243), expect = 2e-19 Length = 546; score = 40.1 bits (18), expect = 0.83; identities = 21/24 (87%); strand = Plus / Plus

```
Query: 23 ttgcttgattcacttcwwyaagag 46
          ||||||||||||||||   |||||
Sbjct: 7  ttgcttgattcacttctacaagag 30
```

>ES237818|ES237818.1 CF_WO0_60d02_SP6 Copepod Whole Organism, Normalized *Calanus finmarchicus* cDNA clone CF_WO0_60d02; 5' similar to ref|XP_001357452.1| GA15447-PA - Drosophila pseudoobscura. Score = 250 bits (639), expect Length = 699; score = 40.1 bits (18), expect = 0.83; identities = 21/24 (87%); strand = Plus / Plus

```
Query: 23 ttgcttgattcacttcwwyaagag 46
```

```
         ||||||||||||||||   |||||
Sbjct: 9  ttgcttgattcacttctacaagag 32
```

>ES237791|ES237791.1 CF_WO0_60a10_SP6 Copepod Whole Organism, Normalized *Calanus finmarchicus* cDNA clone CF_WO0_60a10; 5' similar to ref|XP_001157734.1| DIVDICTED: cyclin J isoform 1 - Pan troglodytes. Score = 120 bits (30 Length = 700; score = 40.1 bits (18), expect = 0.83; identities = 21/24 (87%); strand = Plus / Plus

```
Query: 23 ttgcttgattcacttcwwyaagag 46
          ||||||||||||||||   |||||
Sbjct: 15 ttgcttgattcacttctacaagag 38
```

>ES237789|ES237789.1 CF_WO0_60a08_SP6 Copepod Whole Organism, Normalized *Calanus finmarchicus* cDNA clone CF_WO0_60a08; 5' similar to ref|XP_967085.1| DIVDICTED: similar to Fumarate hydratase, mitochondrial precursor. Score Length = 692; score = 40.1 bits (18), expect = 0.83; identities = 21/24 (87%); strand = Plus / Plus

```
Query: 23 ttgcttgattcacttcwwyaagag 46
          ||||||||||||||||   |||||
Sbjct: 5  ttgcttgattcacttctacaagag 28
```

>ES237788|ES237788.1 CF_WO0_60a07_SP6 Copepod Whole Organism, Normalized *Calanus finmarchicus* cDNA clone CF_WO0_60a07; 5' , mRNA sequence. Length = 676; score = 40.1 bits (18), expect = 0.83; identities = 21/24 (87%); strand = Plus / Plus

```
Query: 23 ttgcttgattcacttcwwyaagag 46
          ||||||||||||||||   |||||
Sbjct: 4  ttgcttgattcacttctacaagag 27
```

>ES237779|ES237779.1 CF_WO0_59h09_SP6 Copepod Whole Organism, Normalized *Calanus finmarchicus* cDNA clone CF_WO0_59h09; 5' similar to gb|AAR12911.1| cyclin B2 - Bufo gargarizans. Score = 113 bits (283), expect = 7e-24-mRNA Length = 665; score = 40.1 bits (18), expect = 0.83; identities = 21/24 (87%); strand = Plus / Plus

```
Query: 23 ttgcttgattcacttcwwyaagag 46
          ||||||||||||||||   |||||
Sbjct: 10 ttgcttgattcacttctacaagag 33
```

>ES237732|ES237732.1 CF_WO0_59d09_SP6 Copepod Whole Organism, Normalized *Calanus finmarchicus* cDNA clone CF_WO0_59d09; 5' similar to ref|XP_513592.2| DIVDICTED: RNA terminal phosphate cyclase domain 1 isoform 6 [Pan. Score Length = 627; score = 40.1 bits (18), expect = 0.83; identities = 21/24 (87%); strand = Plus / Plus

```
Query: 23 ttgcttgattcacttcwwyaagag 46
          ||||||||||||||||   |||||
Sbjct: 8  ttgcttgattcacttctacaagag 31
```

>ES237724|ES237724.1 CF_WO0_59d01_SP6 Copepod Whole Organism, Normalized *Calanus finmarchicus* cDNA clone CF_WO0_59d01; 5' , mRNA sequence. Length = 703; score = 40.1 bits (18), expect = 0.83; identities = 21/24 (87%); strand = Plus / Plus

```
Query: 23 ttgcttgattcacttcwwyaagag 46
          ||||||||||||||||   |||||
Sbjct: 1  ttgcttgattcacttctacaagag 24
```

>ES237685|ES237685.1 CF_WO0_58h09_SP6 Copepod Whole Organism, Normalized *Calanus finmarchicus* cDNA clone CF_WO0_58h09; 5' similar to gb|ABF51383.1| GTP binding translation release factor isoform 2 - Bombyx mori. Score = 62 Length = 690; score = 40.1 bits (18), expect = 0.83; identities = 21/24 (87%); strand = Plus / Plus

```
Query: 23 ttgcttgattcacttcwwyaagag 46
          ||||||||||||||||   |||||
Sbjct: 8  ttgcttgattcacttctacaagag 31
```

>ES237677|ES237677.1 CF_WO0_58h01_SP6 Copepod Whole Organism, Normalized *Calanus finmarchicus* cDNA clone CF_WO0_58h01; 5' similar to ref|XP_971811.1| DIVDICTED: similar to nicalin - Tribolium castaneum. Score = 71.2 bits (Length = 665; score = 40.1 bits (18), expect = 0.83; identities = 21/24 (87%); strand = Plus / Plus

```
Query: 23 ttgcttgattcacttcwwyaagag 46
          ||||||||||||||||   |||||
Sbjct: 11 ttgcttgattcacttctacaagag 34
```

>ES237669|ES237669.1 CF_WO0_58g05_SP6 Copepod Whole Organism, Normalized *Calanus finmarchicus* cDNA clone CF_WO0_58g05; 5' similar to gb|AAH88324.1| Eprs protein - Rattus norvegicus. Score = 388 bits (997), expect = e-106, Length = 688; score = 40.1 bits (18), expect = 0.83; identities = 21/24 (87%); strand = Plus / Plus

```
Query: 23 ttgcttgattcacttcwwyaagag 46
          ||||||||||||||||   |||||
Sbjct: 12 ttgcttgattcacttctacaagag 35
```

>ES237655|ES237655.1 CF_WO0_58f03_SP6 Copepod Whole Organism, Normalized *Calanus finmarchicus* cDNA clone CF_WO0_58f03; 5' similar to gb|EAT46224.1| cationic amino acid transporter - Aedes aegypti. Score = 252 bits (644), E. Length = 723; score = 40.1 bits (18), expect = 0.83; identities = 21/24 (87%); strand = Plus / Plus

```
Query: 23 ttgcttgattcacttcwwyaagag 46
          ||||||||||||||||   |||||
Sbjct: 9  ttgcttgattcacttctacaagag 32
```

>ES237612|ES237612.1 CF_WO0_58b06_SP6 Copepod Whole Organism, Normalized *Calanus finmarchicus* cDNA clone CF_WO0_58b06; 5' , mRNA sequence. Length = 672; score = 40.1 bits (18), expect = 0.83; identities = 21/24 (87%); strand = Plus / Plus

```
Query: 23 ttgcttgattcacttcwwyaagag 46
```

```
         |||||||||||||||   |||||
Sbjct: 7  ttgcttgattcacttctacaagag 30
```

>ES237605|ES237605.1 CF_WO0_58a11_SP6 Copepod Whole Organism, Normalized *Calanus finmarchicus* cDNA clone CF_WO0_58a11; 5' similar to ref|XP_416122.2| DIVDICTED: similar to Chromosome 12 open reading frame 26 [Gallus. Score Length = 694; score = 40.1 bits (18), expect = 0.83; identities = 21/24 (87%); strand = Plus / Plus

```
Query: 23 ttgcttgattcacttcwwyaagag 46
          |||||||||||||||   |||||
Sbjct: 11 ttgcttgattcacttctacaagag 34
```

>ES237595|ES237595.1 CF_WO0_58a01_SP6 Copepod Whole Organism, Normalized *Calanus finmarchicus* cDNA clone CF_WO0_58a01; 5' similar to ref|NP_001036906.1| pyruvate kinase - Bombyx mori. Score = 189 bits (481), expect = 8e-47 Length = 680; score = 40.1 bits (18), expect = 0.83; identities = 21/24 (87%); strand = Plus / Plus

```
Query: 23 ttgcttgattcacttcwwyaagag 46
          |||||||||||||||   |||||
Sbjct: 17 ttgcttgattcacttctacaagag 40
```

>ES237562|ES237562.1 CF_WO0_57f03_SP6 Copepod Whole Organism, Normalized *Calanus finmarchicus* cDNA clone CF_WO0_57f03; 5' similar to ref|XP_967453.1| DIVDICTED: similar to pyruvate dehydrogenase phosphatase regulatory. Sco Length = 636; score = 40.1 bits (18), expect = 0.83; identities = 21/24 (87%); strand = Plus / Plus

```
Query: 23 ttgcttgattcacttcwwyaagag 46
          |||||||||||||||   |||||
Sbjct: 10 ttgcttgattcacttctacaagag 33
```

>ES237484|ES237484.1 CF_WO0_56g07_SP6 Copepod Whole Organism, Normalized *Calanus finmarchicus* cDNA clone CF_WO0_56g07; 5' similar to ref|XP_001361520.1| GA21288-PA - Drosophila pseudoobscura. Score = 181 bits (460), expect Length = 714; score = 40.1 bits (18), expect = 0.83; identities = 21/24 (87%); strand = Plus / Plus

```
Query: 23 ttgcttgattcacttcwwyaagag 46
          |||||||||||||||   |||||
Sbjct: 2  ttgcttgattcacttctacaagag 25
```

>ES237481|ES237481.1 CF_WO0_56g04_SP6 Copepod Whole Organism, Normalized *Calanus finmarchicus* cDNA clone CF_WO0_56g04; 5' similar to ref|XP_791350.2| DIVDICTED: similar to Ribosomal protein L3 [Strongylocentrotus. Score = Length = 731; score = 40.1 bits (18), expect = 0.83; identities = 21/24 (87%); strand = Plus / Plus

```
Query: 23 ttgcttgattcacttcwwyaagag 46
          |||||||||||||||   |||||
Sbjct: 12 ttgcttgattcacttctacaagag 35
```

>ES237458|ES237458.1 CF_WO0_56e04_SP6 Copepod Whole Organism, Normalized *Calanus finmarchicus* cDNA clone CF_WO0_56e04; 5' similar to ref|NP_001015983.1| proteasome (prosome, macropain) 26S subunit, non-ATPase, 5 [Xenopus. Length = 733; score = 40.1 bits (18), expect = 0.83; identities = 21/24 (87%); strand = Plus / Plus

```
Query: 23 ttgcttgattcacttcwwyaagag 46
          ||||||||||||||||   |||||
Sbjct: 9  ttgcttgattcacttctacaagag 32
```

>ES237450|ES237450.1 CF_WO0_56d08_SP6 Copepod Whole Organism, Normalized *Calanus finmarchicus* cDNA clone CF_WO0_56d08; 5' similar to gb|EAT44948.1| glucose transporter - Aedes aegypti. Score = 188 bits (478), expect = 2e-4 Length = 655; score = 40.1 bits (18), expect = 0.83; identities = 21/24 (87%); strand = Plus / Plus

```
Query: 23 ttgcttgattcacttcwwyaagag 46
          ||||||||||||||||   |||||
Sbjct: 5  ttgcttgattcacttctacaagag 28
```

>ES237420|ES237420.1 CF_WO0_56b02_SP6 Copepod Whole Organism, Normalized *Calanus finmarchicus* cDNA clone CF_WO0_56b02; 5' similar to ref|NP_001036341.1| homer CG11324-PC, isoform C - Drosophila melanogaster. Score = 210 bi Length = 685; score = 40.1 bits (18), expect = 0.83; identities = 21/24 (87%); strand = Plus / Plus

```
Query: 23 ttgcttgattcacttcwwyaagag 46
          ||||||||||||||||   |||||
Sbjct: 8  ttgcttgattcacttctacaagag 31
```

>ES237408|ES237408.1 CF_WO0_56a02_SP6 Copepod Whole Organism, Normalized *Calanus finmarchicus* cDNA clone CF_WO0_56a02; 5' similar to gb|EAT43648.1| conserved hypothetical protein - Aedes aegypti. Score = 92.8 bits (229), E. Length = 714; score = 40.1 bits (18), expect = 0.83; identities = 21/24 (87%); strand = Plus / Plus

```
Query: 23 ttgcttgattcacttcwwyaagag 46
          ||||||||||||||||   |||||
Sbjct: 10 ttgcttgattcacttctacaagag 33
```

>ES237357|ES237357.1 CF_WO0_55d11_SP6 Copepod Whole Organism, Normalized *Calanus finmarchicus* cDNA clone CF_WO0_55d11; 5' similar to ref|XP_966481.1| DIVDICTED: similar to CG14648-PA, isoform A - Tribolium castaneum. Score Length = 702; score = 40.1 bits (18), expect = 0.83; identities = 21/24 (87%); strand = Plus / Plus

```
Query: 23 ttgcttgattcacttcwwyaagag 46
          ||||||||||||||||   |||||
Sbjct: 10 ttgcttgattcacttctacaagag 33
```

>ES237333|ES237333.1 CF_WO0_55b11_SP6 Copepod Whole Organism, Normalized *Calanus finmarchicus* cDNA clone CF_WO0_55b11; 5' similar to gb|AAX62435.1| ribosomal protein L4 - Lysiphlebus testaceipes. Score = 349 bits (895), Ex Length = 703; score = 40.1 bits (18), expect = 0.83; identities = 21/24 (87%); strand = Plus / Plus

Query: 23 ttgcttgattcacttcwwyaagag 46
         |||||||||||||||| |||||
Sbjct: 4 ttgcttgattcacttctacaagag 27

>ES237327|ES237327.1 CF_WO0_55b05_SP6 Copepod Whole Organism, Normalized *Calanus finmarchicus* cDNA clone CF_WO0_55b05; 5' , mRNA sequence. Length = 695; score = 40.1 bits (18), expect = 0.83; identities = 21/24 (87%); strand = Plus / Plus

Query: 23 ttgcttgattcacttcwwyaagag 46
         |||||||||||||||| |||||
Sbjct: 14 ttgcttgattcacttctacaagag 37

>EL966267|EL966267.1 CF_WO0_54h09_SP6 Copepod Whole Organism, Normalized *Calanus finmarchicus* cDNA clone CF_WO0_54h09; 5' , mRNA sequence. Length = 710; score = 40.1 bits (18), expect = 0.83; identities = 21/24 (87%); strand = Plus / Plus

Query: 23 ttgcttgattcacttcwwyaagag 46
         |||||||||||||||| |||||
Sbjct: 13 ttgcttgattcacttctacaagag 36

>EL966255|EL966255.1 CF_WO0_54g09_SP6 Copepod Whole Organism, Normalized *Calanus finmarchicus* cDNA clone CF_WO0_54g09; 5' similar to ref|XP_001355229.1| GA17462-PA - Drosophila pseudoobscura. Score = 179 bits (453), expect Length = 670; score = 40.1 bits (18), expect = 0.83; identities = 21/24 (87%); strand = Plus / Plus

Query: 23 ttgcttgattcacttcwwyaagag 46
         |||||||||||||||| |||||
Sbjct: 4 ttgcttgattcacttctacaagag 27

>EL966238|EL966238.1 CF_WO0_54f03_SP6 Copepod Whole Organism, Normalized *Calanus finmarchicus* cDNA clone CF_WO0_54f03; 5' similar to ref|XP_624580.1| DIVDICTED: similar to Chromatin assembly factor 1 subunit CG4236-PA. Sco Length = 675; score = 40.1 bits (18), expect = 0.83; identities = 21/24 (87%); strand = Plus / Plus

Query: 23 ttgcttgattcacttcwwyaagag 46
         |||||||||||||||| |||||
Sbjct: 15 ttgcttgattcacttctacaagag 38

>EL966211|EL966211.1 CF_WO0_54c11_SP6 Copepod Whole Organism, Normalized *Calanus finmarchicus* cDNA clone CF_WO0_54c11; 5' , mRNA sequence. Length = 711; score = 40.1 bits (18), expect = 0.83; identities = 21/24 (87%); strand = Plus / Plus

Query: 23 ttgcttgattcacttcwwyaagag 46
         |||||||||||||||| |||||
Sbjct: 3 ttgcttgattcacttctacaagag 26

>EL966192|EL966192.1 CF_WO0_54b03_SP6 Copepod Whole Organism, Normalized *Calanus finmarchicus* cDNA clone CF_WO0_54b03; 5' similar to ref|XP_314568.3| ENSANGP00000017986 - Anopheles

gambiae str. PEST. Score = 83.6 bits (205 Length = 672; score = 40.1 bits (18), expect = 0.83; identities = 21/24 (87%); strand = Plus / Plus

```
Query: 23 ttgcttgattcacttcwwyaagag 46
          ||||||||||||||||   |||||
Sbjct: 10 ttgcttgattcacttctacaagag 33
```

>EL966178|EL966178.1 CF_WO0_53h12_SP6 Copepod Whole Organism, Normalized *Calanus finmarchicus* cDNA clone CF_WO0_53h12; 5' similar to ref|XP_543416.2| DIVDICTED: similar to F-box only protein 21 isoform 2 [Canis. Score = 85 Length = 722; score = 40.1 bits (18), expect = 0.83; identities = 21/24 (87%); strand = Plus / Plus

```
Query: 23 ttgcttgattcacttcwwyaagag 46
          ||||||||||||||||   |||||
Sbjct: 14 ttgcttgattcacttctacaagag 37
```

>EL966144|EL966144.1 CF_WO0_53f01_SP6 Copepod Whole Organism, Normalized *Calanus finmarchicus* cDNA clone CF_WO0_53f01; 5' similar to gb|AAH83505.1| Arginyl-tRNA synthetase - Danio rerio. Score = 129 bits (324), expect = 1e Length = 665; score = 40.1 bits (18), expect = 0.83; identities = 21/24 (87%); strand = Plus / Plus

```
Query: 23 ttgcttgattcacttcwwyaagag 46
          ||||||||||||||||   |||||
Sbjct: 10 ttgcttgattcacttctacaagag 33
```

>EL966110|EL966110.1 CF_WO0_53c02_SP6 Copepod Whole Organism, Normalized *Calanus finmarchicus* cDNA clone CF_WO0_53c02; 5' similar to emb|CAF90090.1| unnamed protein product - Tetraodon nigroviridis. Score = 251 bits (642), Length = 652; score = 40.1 bits (18), expect = 0.83; identities = 21/24 (87%); strand = Plus / Plus

```
Query: 23 ttgcttgattcacttcwwyaagag 46
          ||||||||||||||||   |||||
Sbjct: 9  ttgcttgattcacttctacaagag 32
```

>EL966087|EL966087.1 CF_WO0_53a02_SP6 Copepod Whole Organism, Normalized *Calanus finmarchicus* cDNA clone CF_WO0_53a02; 5' similar to ref|XP_973312.1| DIVDICTED: similar to CG9805-PA - Tribolium castaneum. Score = 73.9 bits Length = 479; score = 40.1 bits (18), expect = 0.83; identities = 21/24 (87%); strand = Plus / Plus

```
Query: 23 ttgcttgattcacttcwwyaagag 46
          ||||||||||||||||   |||||
Sbjct: 6  ttgcttgattcacttctacaagag 29
```

>EL966053|EL966053.1 CF_WO0_52f01_SP6 Copepod Whole Organism, Normalized *Calanus finmarchicus* cDNA clone CF_WO0_52f01; 5' similar to ref|XP_001100583.1| DIVDICTED: similar to Ras association domain family 1 isoform A. Scor Length = 701; score = 40.1 bits (18), expect = 0.83; identities = 21/24 (87%); strand = Plus / Plus

```
Query: 23 ttgcttgattcacttcwwyaagag 46
```

```
         ||||||||||||||||   |||||
Sbjct: 7  ttgcttgattcacttctacaagag 30
```

>EL966047|EL966047.1 CF_WO0_52e07_SP6 Copepod Whole Organism, Normalized *Calanus finmarchicus* cDNA clone CF_WO0_52e07; 5’ similar to ref|XP_971904.1| DIVDICTED: similar to CG18769-PB, isoform B - Tribolium castaneum. Score Length = 701; score = 40.1 bits (18), expect = 0.83; identities = 21/24 (87%); strand = Plus / Plus

```
Query: 23 ttgcttgattcacttcwwyaagag 46
          ||||||||||||||||   |||||
Sbjct: 2  ttgcttgattcacttctacaagag 25
```

>EL966032|EL966032.1 CF_WO0_52d04_SP6 Copepod Whole Organism, Normalized *Calanus finmarchicus* cDNA clone CF_WO0_52d04; 5’ similar to ref|XP_967434.1| DIVDICTED: similar to CG7899-PA, isoform A - Tribolium castaneum. Score Length = 706; score = 40.1 bits (18), expect = 0.83; identities = 21/24 (87%); strand = Plus / Plus

```
Query: 23 ttgcttgattcacttcwwyaagag 46
          ||||||||||||||||   |||||
Sbjct: 17 ttgcttgattcacttctacaagag 40
```

>EL966018|EL966018.1 CF_WO0_52c02_SP6 Copepod Whole Organism, Normalized *Calanus finmarchicus* cDNA clone CF_WO0_52c02; 5’ similar to ref|XP_308308.3| ENSANGP00000010875 - Anopheles gambiae str. PEST. Score = 204 bits (519) Length = 740; score = 40.1 bits (18), expect = 0.83; identities = 21/24 (87%); strand = Plus / Plus

```
Query: 23 ttgcttgattcacttcwwyaagag 46
          ||||||||||||||||   |||||
Sbjct: 9  ttgcttgattcacttctacaagag 32
```

>EL965984|EL965984.1 CF_WO0_51h03_SP6 Copepod Whole Organism, Normalized *Calanus finmarchicus* cDNA clone CF_WO0_51h03; 5’, mRNA sequence. Length = 680; score = 40.1 bits (18), expect = 0.83; identities = 21/24 (87%); strand = Plus / Plus

```
Query: 23 ttgcttgattcacttcwwyaagag 46
          ||||||||||||||||   |||||
Sbjct: 10 ttgcttgattcacttctacaagag 33
```

>EL965971|EL965971.1 CF_WO0_51g02_SP6 Copepod Whole Organism, Normalized *Calanus finmarchicus* cDNA clone CF_WO0_51g02; 5’, mRNA sequence. Length = 716; score = 40.1 bits (18), expect = 0.83; identities = 21/24 (87%); strand = Plus / Plus

```
Query: 23 ttgcttgattcacttcwwyaagag 46
          ||||||||||||||||   |||||
Sbjct: 7  ttgcttgattcacttctacaagag 30
```

>EL965956|EL965956.1 CF_WO0_51e10_SP6 Copepod Whole Organism, Normalized *Calanus finmarchicus* cDNA clone CF_WO0_51e10; 5’ similar to gb|AAR99908.1| catalase - Litopenaeus vannamei. Score

= 372 bits (956), expect = e-102, m Length = 726; score = 40.1 bits (18), expect = 0.83; identities = 21/24 (87%); strand = Plus / Plus

```
Query: 23 ttgcttgattcacttcwwyaagag 46
          ||||||||||||||||   |||||
Sbjct: 8  ttgcttgattcacttctacaagag 31
```

>EL965931|EL965931.1 CF_WO0_51c09_SP6 Copepod Whole Organism, Normalized *Calanus finmarchicus* cDNA clone CF_WO0_51c09; 5' similar to ref|XP_392026.2| DIVDICTED: similar to ced-6 CG11804-PC, isoform C - Apis mellifera. Scor Length = 677; score = 40.1 bits (18), expect = 0.83; identities = 21/24 (87%); strand = Plus / Plus

```
Query: 23 ttgcttgattcacttcwwyaagag 46
          ||||||||||||||||   |||||
Sbjct: 8  ttgcttgattcacttctacaagag 31
```

>EL965928|EL965928.1 CF_WO0_51c06_SP6 Copepod Whole Organism, Normalized *Calanus finmarchicus* cDNA clone CF_WO0_51c06; 5' similar to ref|XP_971461.1| DIVDICTED: similar to CG4713-PA - Tribolium castaneum. Score = 85.9 bits Length = 739; score = 40.1 bits (18), expect = 0.83; identities = 21/24 (87%); strand = Plus / Plus

```
Query: 23 ttgcttgattcacttcwwyaagag 46
          ||||||||||||||||   |||||
Sbjct: 8  ttgcttgattcacttctacaagag 31
```

>EL965889|EL965889.1 CF_WO0_50h01_SP6 Copepod Whole Organism, Normalized *Calanus finmarchicus* cDNA clone CF_WO0_50h01; 5' , mRNA sequence. Length = 739; score = 40.1 bits (18), expect = 0.83; identities = 21/24 (87%); strand = Plus / Plus

```
Query: 23 ttgcttgattcacttcwwyaagag 46
          ||||||||||||||||   |||||
Sbjct: 6  ttgcttgattcacttctacaagag 29
```

>EL965878|EL965878.1 CF_WO0_50g02_SP6 Copepod Whole Organism, Normalized *Calanus finmarchicus* cDNA clone CF_WO0_50g02; 5' , mRNA sequence. Length = 738; score = 40.1 bits (18), expect = 0.83; identities = 21/24 (87%); strand = Plus / Plus

```
Query: 23 ttgcttgattcacttcwwyaagag 46
          ||||||||||||||||   |||||
Sbjct: 10 ttgcttgattcacttctacaagag 33
```

>EL965874|EL965874.1 CF_WO0_50f10_SP6 Copepod Whole Organism, Normalized *Calanus finmarchicus* cDNA clone CF_WO0_50f10; 5' , mRNA sequence. Length = 702; score = 40.1 bits (18), expect = 0.83; identities = 21/24 (87%); strand = Plus / Plus

```
Query: 23 ttgcttgattcacttcwwyaagag 46
          ||||||||||||||||   |||||
Sbjct: 9  ttgcttgattcacttctacaagag 32
```

>EL965873|EL965873.1 CF_WO0_50f09_SP6 Copepod Whole Organism, Normalized *Calanus finmarchicus* cDNA clone CF_WO0_50f09; 5’ similar to gb|EAT48688.1| heat shock protein - Aedes aegypti. Score = 193 bits (490), expect = 7e-48 Length = 696; score = 40.1 bits (18), expect = 0.83; identities = 21/24 (87%); strand = Plus / Plus

```
Query: 23 ttgcttgattcacttcwwyaagag 46
          ||||||||||||||||   |||||
Sbjct: 4  ttgcttgattcacttctacaagag 27
```

>EL965870|EL965870.1 CF_WO0_50f06_SP6 Copepod Whole Organism, Normalized *Calanus finmarchicus* cDNA clone CF_WO0_50f06; 5’ , mRNA sequence. Length = 626; score = 40.1 bits (18), expect = 0.83; identities = 21/24 (87%); strand = Plus / Plus

```
Query: 23 ttgcttgattcacttcwwyaagag 46
          ||||||||||||||||   |||||
Sbjct: 9  ttgcttgattcacttctacaagag 32
```

>EL965847|EL965847.1 CF_WO0_50d07_SP6 Copepod Whole Organism, Normalized *Calanus finmarchicus* cDNA clone CF_WO0_50d07; 5’ similar to ref|XP_609410.3| DIVDICTED: hypothetical protein - Bos taurus. Score = 99.0 bits (245), E. Length = 710; score = 40.1 bits (18), expect = 0.83; identities = 21/24 (87%); strand = Plus / Plus

```
Query: 23 ttgcttgattcacttcwwyaagag 46
          ||||||||||||||||   |||||
Sbjct: 10 ttgcttgattcacttctacaagag 33
```

>EL965833|EL965833.1 CF_WO0_50c05_SP6 Copepod Whole Organism, Normalized *Calanus finmarchicus* cDNA clone CF_WO0_50c05; 5’ similar to gb|EAT41627.1| 2-oxoglutarate dehydrogenase - Aedes aegypti. Score = 135 bits (341), Expe Length = 695; score = 40.1 bits (18), expect = 0.83; identities = 21/24 (87%); strand = Plus / Plus

```
Query: 23 ttgcttgattcacttcwwyaagag 46
          ||||||||||||||||   |||||
Sbjct: 10 ttgcttgattcacttctacaagag 33
```

>EL965826|EL965826.1 CF_WO0_50b10_SP6 Copepod Whole Organism, Normalized *Calanus finmarchicus* cDNA clone CF_WO0_50b10; 5’ similar to ref|XP_967114.1| DIVDICTED: similar to CG11896-PA - Tribolium castaneum. Score = 248 bits Length = 686; score = 40.1 bits (18), expect = 0.83; identities = 21/24 (87%); strand = Plus / Plus

```
Query: 23 ttgcttgattcacttcwwyaagag 46
          ||||||||||||||||   |||||
Sbjct: 27 ttgcttgattcacttctacaagag 50
```

>EL965789|EL965789.1 CF_WO0_49g08_SP6 Copepod Whole Organism, Normalized *Calanus finmarchicus* cDNA clone CF_WO0_49g08; 5’ similar to gb|EAT43642.1| lysosomal acid lipase, putative - Aedes aegypti. Score = 92.8 bits (229), Length = 727; score = 40.1 bits (18), expect = 0.83; identities = 21/24 (87%); strand = Plus / Plus

Query: 23 ttgcttgattcacttcwwyaagag 46
         |||||||||||||||| |||||
Sbjct: 8 ttgcttgattcacttctacaagag 31

>EL965786|EL965786.1 CF_WOO_49g05_SP6 Copepod Whole Organism, Normalized *Calanus finmarchicus* cDNA clone CF_WOO_49g05; 5' similar to emb|CAC42098.1| RBD protein - Chironomus tentans. Score = 105 bits (261), expect = 3e-21, Length = 675; score = 40.1 bits (18), expect = 0.83; identities = 21/24 (87%); strand = Plus / Plus

Query: 23 ttgcttgattcacttcwwyaagag 46
         |||||||||||||||| |||||
Sbjct: 3 ttgcttgattcacttctacaagag 26

>EL965765|EL965765.1 CF_WOO_49e08_SP6 Copepod Whole Organism, Normalized *Calanus finmarchicus* cDNA clone CF_WOO_49e08; 5' similar to ref|NP_989766.1| asparagine-linked glycosylation 6 - Gallus gallus. Score = 256 bits (653 Length = 665; score = 40.1 bits (18), expect = 0.83; identities = 21/24 (87%); strand = Plus / Plus

Query: 23 ttgcttgattcacttcwwyaagag 46
         |||||||||||||||| |||||
Sbjct: 9 ttgcttgattcacttctacaagag 32

>EL965759|EL965759.1 CF_WOO_49e02_SP6 Copepod Whole Organism, Normalized *Calanus finmarchicus* cDNA clone CF_WOO_49e02; 5' similar to ref|XP_395364.2| DIVDICTED: similar to prolyl endopeptidase isoform 1 [Apis. Score = 259 Length = 746; score = 40.1 bits (18), expect = 0.83; identities = 21/24 (87%); strand = Plus / Plus

Query: 23 ttgcttgattcacttcwwyaagag 46
         |||||||||||||||| |||||
Sbjct: 8 ttgcttgattcacttctacaagag 31

>EL965751|EL965751.1 CF_WOO_49d06_SP6 Copepod Whole Organism, Normalized *Calanus finmarchicus* cDNA clone CF_WOO_49d06; 5' similar to ref|XP_969453.1| DIVDICTED: similar to CG9556-PB, isoform B - Tribolium castaneum. Score Length = 681; score = 40.1 bits (18), expect = 0.83; identities = 21/24 (87%); strand = Plus / Plus

Query: 23 ttgcttgattcacttcwwyaagag 46
         |||||||||||||||| |||||
Sbjct: 11 ttgcttgattcacttctacaagag 34

>EL965692|EL965692.1 CF_WOO_48g05_SP6 Copepod Whole Organism, Normalized *Calanus finmarchicus* cDNA clone CF_WOO_48g05; 5', mRNA sequence. Length = 606; score = 40.1 bits (18), expect = 0.83; identities = 21/24 (87%); strand = Plus / Plus

Query: 23 ttgcttgattcacttcwwyaagag 46
         |||||||||||||||| |||||
Sbjct: 10 ttgcttgattcacttctacaagag 33

>EL965678|EL965678.1 CF_W00_48f03_SP6 Copepod Whole Organism, Normalized *Calanus finmarchicus* cDNA clone CF_W00_48f03; 5' , mRNA sequence. Length = 680; score = 40.1 bits (18), expect = 0.83; identities = 21/24 (87%); strand = Plus / Plus

```
Query: 23 ttgcttgattcacttcwwyaagag 46
          |||||||||||||||   |||||
Sbjct: 9  ttgcttgattcacttctacaagag 32
```

>EL965668|EL965668.1 CF_W00_48e04_SP6 Copepod Whole Organism, Normalized *Calanus finmarchicus* cDNA clone CF_W00_48e04; 5' similar to ref|XP_783607.1| DIVDICTED: hypothetical protein isoform 4 [Strongylocentrotus. Score = 7 Length = 704; score = 40.1 bits (18), expect = 0.83; identities = 21/24 (87%); strand = Plus / Plus

```
Query: 23 ttgcttgattcacttcwwyaagag 46
          |||||||||||||||   |||||
Sbjct: 15 ttgcttgattcacttctacaagag 38
```

>EL965667|EL965667.1 CF_W00_48e03_SP6 Copepod Whole Organism, Normalized *Calanus finmarchicus* cDNA clone CF_W00_48e03; 5' similar to gb|EAT35814.1| conserved hypothetical protein - Aedes aegypti. Score = 172 bits (435), Ex Length = 760; score = 40.1 bits (18), expect = 0.83; identities = 21/24 (87%); strand = Plus / Plus

```
Query: 23 ttgcttgattcacttcwwyaagag 46
          |||||||||||||||   |||||
Sbjct: 15 ttgcttgattcacttctacaagag 38
```

>EL965598|EL965598.1 CF_W00_47g03_SP6 Copepod Whole Organism, Normalized *Calanus finmarchicus* cDNA clone CF_W00_47g03; 5' similar to ref|NP_651578.1| CG5590-PA - Drosophila melanogaster. Score = 308 bits (788), expect = 2e Length = 678; score = 40.1 bits (18), expect = 0.83; identities = 21/24 (87%); strand = Plus / Plus

```
Query: 23 ttgcttgattcacttcwwyaagag 46
          |||||||||||||||   |||||
Sbjct: 15 ttgcttgattcacttctacaagag 38
```

>EL965589|EL965589.1 CF_W00_47f06_SP6 Copepod Whole Organism, Normalized *Calanus finmarchicus* cDNA clone CF_W00_47f06; 5' similar to ref|XP_001123261.1| DIVDICTED: similar to CG6070-PA, partial - Apis mellifera. Score = 19 Length = 712; score = 40.1 bits (18), expect = 0.83; identities = 21/24 (87%); strand = Plus / Plus

```
Query: 23 ttgcttgattcacttcwwyaagag 46
          |||||||||||||||   |||||
Sbjct: 8  ttgcttgattcacttctacaagag 31
```

>EL965586|EL965586.1 CF_W00_47f03_SP6 Copepod Whole Organism, Normalized *Calanus finmarchicus* cDNA clone CF_W00_47f03; 5' similar to emb|CAE71845.1| Hypothetical protein CBG18887 - Caenorhabditis briggsae. Score = 298 bits Length = 727; score = 40.1 bits (18), expect = 0.83; identities = 21/24 (87%); strand = Plus / Plus

Query: 23 ttgcttgattcacttcwwyaagag 46
|||||||||||||||| |||||
Sbjct: 22 ttgcttgattcacttctacaagag 45

>EL965578|EL965578.1 CF_W00_47e07_SP6 Copepod Whole Organism, Normalized *Calanus finmarchicus* cDNA clone CF_W00_47e07; 5' similar to ref|NP_651963.1| Sucb CG10622-PA, isoform A - Drosophila melanogaster. Score = 216 bits (Length = 624; score = 40.1 bits (18), expect = 0.83; identities = 21/24 (87%); strand = Plus / Plus

Query: 23 ttgcttgattcacttcwwyaagag 46
|||||||||||||||| |||||
Sbjct: 9 ttgcttgattcacttctataagag 32

>EL965573|EL965573.1 CF_W00_47e02_SP6 Copepod Whole Organism, Normalized *Calanus finmarchicus* cDNA clone CF_W00_47e02; 5' similar to ref|XP_318985.3| ENSANGP00000014807 - Anopheles gambiae str. PEST. Score = 73.2 bits (178 Length = 628; score = 40.1 bits (18), expect = 0.83; identities = 21/24 (87%); strand = Plus / Plus

Query: 23 ttgcttgattcacttcwwyaagag 46
|||||||||||||||| |||||
Sbjct: 3 ttgcttgattcacttctacaagag 26

>EL965546|EL965546.1 CF_W00_47b11_SP6 Copepod Whole Organism, Normalized *Calanus finmarchicus* cDNA clone CF_W00_47b11; 5', mRNA sequence. Length = 439; score = 40.1 bits (18), expect = 0.83; identities = 21/24 (87%); strand = Plus / Plus

Query: 23 ttgcttgattcacttcwwyaagag 46
|||||||||||||||| |||||
Sbjct: 6 ttgcttgattcacttctacaagag 29

>EL965539|EL965539.1 CF_W00_47b04_SP6 Copepod Whole Organism, Normalized *Calanus finmarchicus* cDNA clone CF_W00_47b04; 5', mRNA sequence. Length = 688; score = 40.1 bits (18), expect = 0.83; identities = 21/24 (87%); strand = Plus / Plus

Query: 23 ttgcttgattcacttcwwyaagag 46
|||||||||||||||| |||||
Sbjct: 8 ttgcttgattcacttctacaagag 31

>EL965493|EL965493.1 CF_W00_46f06_SP6 Copepod Whole Organism, Normalized *Calanus finmarchicus* cDNA clone CF_W00_46f06; 5' similar to ref|XP_392018.2| DIVDICTED: similar to CG33156-PB, isoform B isoform 1 [Apis. Score = 151 Length = 716; score = 40.1 bits (18), expect = 0.83; identities = 21/24 (87%); strand = Plus / Plus

Query: 23 ttgcttgattcacttcwwyaagag 46
|||||||||||||||| |||||
Sbjct: 12 ttgcttgattcacttctacaagag 35

>EL965452|EL965452.1 CF_W00_46b11_SP6 Copepod Whole Organism, Normalized *Calanus finmarchicus* cDNA clone CF_W00_46b11; 5' similar to ref|XP_973304.1| DIVDICTED: similar to CG8808-PA -

Tribolium castaneum. Score = 203 bits Length = 634; score = 40.1 bits (18), expect = 0.83; identities = 21/24 (87%); strand = Plus / Plus

```
Query: 23 ttgcttgattcacttcwwyaagag 46
          ||||||||||||||||   |||||
Sbjct: 7  ttgcttgattcacttctacaagag 30
```

>EL965442|EL965442.1 CF_WO0_46b01_SP6 Copepod Whole Organism, Normalized *Calanus finmarchicus* cDNA clone CF_WO0_46b01; 5' similar to sp|Q5ZHZ0|UAP56_CHICK Spliceosome RNA helicase BAT1 (DEAD box protein UAP56) (56 kDa. Sco Length = 653; score = 40.1 bits (18), expect = 0.83; identities = 21/24 (87%); strand = Plus / Plus

```
Query: 23 ttgcttgattcacttcwwyaagag 46
          ||||||||||||||||   |||||
Sbjct: 8  ttgcttgattcacttctacaagag 31
```

>EL965434|EL965434.1 CF_WO0_46a05_SP6 Copepod Whole Organism, Normalized *Calanus finmarchicus* cDNA clone CF_WO0_46a05; 5' similar to ref|XP_001123261.1| DIVDICTED: similar to CG6070-PA, partial - Apis mellifera. Score = 19 Length = 655; score = 40.1 bits (18), expect = 0.83; identities = 21/24 (87%); strand = Plus / Plus

```
Query: 23 ttgcttgattcacttcwwyaagag 46
          ||||||||||||||||   |||||
Sbjct: 5  ttgcttgattcacttctacaagag 28
```

>EL965432|EL965432.1 CF_WO0_46a03_SP6 Copepod Whole Organism, Normalized *Calanus finmarchicus* cDNA clone CF_WO0_46a03; 5', mRNA sequence. Length = 682; score = 40.1 bits (18), expect = 0.83; identities = 21/24 (87%); strand = Plus / Plus

```
Query: 23 ttgcttgattcacttcwwyaagag 46
          ||||||||||||||||   |||||
Sbjct: 14 ttgcttgattcacttctacaagag 37
```

>EL965408|EL965408.1 CF_WO0_45g02_SP6 Copepod Whole Organism, Normalized *Calanus finmarchicus* cDNA clone CF_WO0_45g02; 5', mRNA sequence. Length = 706; score = 40.1 bits (18), expect = 0.83; identities = 21/24 (87%); strand = Plus / Plus

```
Query: 23 ttgcttgattcacttcwwyaagag 46
          ||||||||||||||||   |||||
Sbjct: 10 ttgcttgattcacttctacaagag 33
```

>EL965404|EL965404.1 CF_WO0_45f10_SP6 Copepod Whole Organism, Normalized *Calanus finmarchicus* cDNA clone CF_WO0_45f10; 5' similar to ref|XP_624580.1| DIVDICTED: similar to Chromatin assembly factor 1 subunit CG4236-PA. Sco Length = 675; score = 40.1 bits (18), expect = 0.83; identities = 21/24 (87%); strand = Plus / Plus

```
Query: 23 ttgcttgattcacttcwwyaagag 46
          ||||||||||||||||   |||||
Sbjct: 13 ttgcttgattcacttctacaagag 36
```

>EL965393|EL965393.1 CF_WO0_45e10_SP6 Copepod Whole Organism, Normalized *Calanus finmarchicus* cDNA clone CF_WO0_45e10; 5' , mRNA sequence. Length = 686; score = 40.1 bits (18), expect = 0.83; identities = 21/24 (87%); strand = Plus / Plus

```
Query: 23 ttgcttgattcacttcwwyaagag 46
          ||||||||||||||||   |||||
Sbjct: 11 ttgcttgattcacttctacaagag 34
```

>EL965385|EL965385.1 CF_WO0_45e02_SP6 Copepod Whole Organism, Normalized *Calanus finmarchicus* cDNA clone CF_WO0_45e02; 5' similar to ref|XP_001190551.1| DIVDICTED: similar to Prt1 homolog - Strongylocentrotus purpuratus. S Length = 741; score = 40.1 bits (18), expect = 0.83; identities = 21/24 (87%); strand = Plus / Plus

```
Query: 23 ttgcttgattcacttcwwyaagag 46
          ||||||||||||||||   |||||
Sbjct: 5  ttgcttgattcacttctacaagag 28
```

>EL965355|EL965355.1 CF_WO0_45b08_SP6 Copepod Whole Organism, Normalized *Calanus finmarchicus* cDNA clone CF_WO0_45b08; 5' similar to ref|XP_971005.1| DIVDICTED: similar to CG3171-PA - Tribolium castaneum. Score = 175 bits Length = 720; score = 40.1 bits (18), expect = 0.83; identities = 21/24 (87%); strand = Plus / Plus

```
Query: 23 ttgcttgattcacttcwwyaagag 46
          ||||||||||||||||   |||||
Sbjct: 10 ttgcttgattcacttctacaagag 33
```

>EL774187|EL774187.1 CF_WO0_44d06_SP6 Copepod Whole Organism, Normalized *Calanus finmarchicus* cDNA clone CF_WO0_44d06; 5' similar to gb|EAT38720.1| ccr4-associated factor - Aedes aegypti. Score = 316 bits (809), expect = 8 Length = 708; score = 40.1 bits (18), expect = 0.83; identities = 21/24 (87%); strand = Plus / Plus

```
Query: 23 ttgcttgattcacttcwwyaagag 46
          ||||||||||||||||   |||||
Sbjct: 4  ttgcttgattcacttctacaagag 27
```

>EL774136|EL774136.1 CF_WO0_43h03_SP6 Copepod Whole Organism, Normalized *Calanus finmarchicus* cDNA clone CF_WO0_43h03; 5' similar to gb|AAH66855.1| RAD17 homolog (S. pombe) - Mus musculus. Score = 89.7 bits (221), expect = Length = 645; score = 40.1 bits (18), expect = 0.83; identities = 21/24 (87%); strand = Plus / Plus

```
Query: 23 ttgcttgattcacttcwwyaagag 46
          ||||||||||||||||   |||||
Sbjct: 10 ttgcttgattcacttctacaagag 33
```

>EL774103|EL774103.1 CF_WO0_43e06_SP6 Copepod Whole Organism, Normalized *Calanus finmarchicus* cDNA clone CF_WO0_43e06; 5' similar to ref|NP_649209.1| CG5585-PA - Drosophila melanogaster. Score = 317 bits (813), expect = 3e Length = 701; score = 40.1 bits (18), expect = 0.83; identities = 21/24 (87%); strand = Plus / Plus

```
Query: 23 ttgcttgattcacttcwwyaagag 46
          ||||||||||||||||   |||||
Sbjct: 10 ttgcttgattcacttctacaagag 33
```

>EL774058|EL774058.1 CF_WOO_43a08_SP6 Copepod Whole Organism, Normalized *Calanus finmarchicus* cDNA clone CF_WOO_43a08; 5' similar to gb|EAT43648.1| conserved hypothetical protein - Aedes aegypti. Score = 94.4 bits (233), E. Length = 724; score = 40.1 bits (18), expect = 0.83; identities = 21/24 (87%); strand = Plus / Plus

```
Query: 23 ttgcttgattcacttcwwyaagag 46
          ||||||||||||||||   |||||
Sbjct: 8  ttgcttgattcacttctacaagag 31
```

>EL774029|EL774029.1 CF_WOO_42g03_SP6 Copepod Whole Organism, Normalized *Calanus finmarchicus* cDNA clone CF_WOO_42g03; 5' similar to ref|XP_372429.5| DIVDICTED: similar to jumonji domain containing 2D - Homo sapiens. Score Length = 716; score = 40.1 bits (18), expect = 0.83; identities = 21/24 (87%); strand = Plus / Plus

```
Query: 23 ttgcttgattcacttcwwyaagag 46
          ||||||||||||||||   |||||
Sbjct: 3  ttgcttgattcacttcaacaagag 26
```

>EL774028|EL774028.1 CF_WOO_42g02_SP6 Copepod Whole Organism, Normalized *Calanus finmarchicus* cDNA clone CF_WOO_42g02; 5' similar to gb|AAH46839.1| Plk-prov protein - Xenopus laevis. Score = 151 bits (382), expect = 2e-35, Length = 681; score = 40.1 bits (18), expect = 0.83; identities = 21/24 (87%); strand = Plus / Plus

```
Query: 23 ttgcttgattcacttcwwyaagag 46
          ||||||||||||||||   |||||
Sbjct: 20 ttgcttgattcacttctacaagag 43
```

>EL774024|EL774024.1 CF_WOO_42f10_SP6 Copepod Whole Organism, Normalized *Calanus finmarchicus* cDNA clone CF_WOO_42f10; 5' similar to gb|EAT37914.1| conserved hypothetical protein - Aedes aegypti. Score = 179 bits (454), Ex Length = 670; score = 40.1 bits (18), expect = 0.83; identities = 21/24 (87%); strand = Plus / Plus

```
Query: 23 ttgcttgattcacttcwwyaagag 46
          ||||||||||||||||   |||||
Sbjct: 11 ttgcttgattcacttctacaagag 34
```

>EL774021|EL774021.1 CF_WOO_42f07_SP6 Copepod Whole Organism, Normalized *Calanus finmarchicus* cDNA clone CF_WOO_42f07; 5' similar to ref|NP_650111.3| CG14722-PA - Drosophila melanogaster. Score = 60.1 bits (144), expect = Length = 703; score = 40.1 bits (18), expect = 0.83; identities = 21/24 (87%); strand = Plus / Plus

```
Query: 23 ttgcttgattcacttcwwyaagag 46
          ||||||||||||||||   |||||
Sbjct: 6  ttgcttgattcacttctacaagag 29
```

>EL774006|EL774006.1 CF_WOO_42e03_SP6 Copepod Whole Organism, Normalized *Calanus finmarchicus* cDNA clone CF_WOO_42e03; 5’ similar to ref|XP_969453.1| DIVDICTED: similar to CG9556-PB, isoform B - Tribolium castaneum. Score Length = 655; score = 40.1 bits (18), expect = 0.83; identities = 21/24 (87%); strand = Plus / Plus

```
Query: 23 ttgcttgattcacttcwwyaagag 46
          ||||||||||||||||   |||||
Sbjct: 10 ttgcttgattcacttctacaagag 33
```

>EL774003|EL774003.1 CF_WOO_42d12_SP6 Copepod Whole Organism, Normalized *Calanus finmarchicus* cDNA clone CF_WOO_42d12; 5’ similar to ref|XP_396204.3| DIVDICTED: similar to CG8195-PA - Apis mellifera. Score = 110 bits (275) Length = 691; score = 40.1 bits (18), expect = 0.83; identities = 21/24 (87%); strand = Plus / Plus

```
Query: 23 ttgcttgattcacttcwwyaagag 46
          ||||||||||||||||   |||||
Sbjct: 15 ttgcttgattcacttctacaagag 38
```

>EL773976|EL773976.1 CF_WOO_42b09_SP6 Copepod Whole Organism, Normalized *Calanus finmarchicus* cDNA clone CF_WOO_42b09; 5’, mRNA sequence. Length = 717; score = 40.1 bits (18), expect = 0.83; identities = 21/24 (87%); strand = Plus / Plus

```
Query: 23 ttgcttgattcacttcwwyaagag 46
          ||||||||||||||||   |||||
Sbjct: 10 ttgcttgattcacttctacaagag 33
```

>EL773963|EL773963.1 CF_WOO_42a08_SP6 Copepod Whole Organism, Normalized *Calanus finmarchicus* cDNA clone CF_WOO_42a08; 5’ similar to ref|XP_394386.3| DIVDICTED: similar to CG17528-PC.3 - Apis mellifera. Score = 83.2 bits (Length = 689; score = 40.1 bits (18), expect = 0.83; identities = 21/24 (87%); strand = Plus / Plus

```
Query: 23 ttgcttgattcacttcwwyaagag 46
          ||||||||||||||||   |||||
Sbjct: 14 ttgcttgattcacttctacaagag 37
```

>EL773951|EL773951.1 CF_WOO_41h08_SP6 Copepod Whole Organism, Normalized *Calanus finmarchicus* cDNA clone CF_WOO_41h08; 5’ similar to emb|CAI12908.1| selenophosphate synthetase 1 - Homo sapiens. Score = 186 bits (473), Expe Length = 678; score = 40.1 bits (18), expect = 0.83; identities = 21/24 (87%); strand = Plus / Plus

```
Query: 23 ttgcttgattcacttcwwyaagag 46
          ||||||||||||||||   |||||
Sbjct: 7  ttgcttgattcacttctacaagag 30
```

>EL773933|EL773933.1 CF_WOO_41g01_SP6 Copepod Whole Organism, Normalized *Calanus finmarchicus* cDNA clone CF_WOO_41g01; 5’ similar to ref|XP_320073.3| ENSANGP00000012293 - Anopheles gambiae str. PEST. Score = 203 bits (516) Length = 700; score = 40.1 bits (18), expect = 0.83; identities = 21/24 (87%); strand = Plus / Plus

```
Query: 23 ttgcttgattcacttcwwyaagag 46
          ||||||||||||||||   |||||
Sbjct: 7  ttgcttgattcacttctacaagag 30
```

>EL773931|EL773931.1 CF_W00_41f11_SP6 Copepod Whole Organism, Normalized *Calanus finmarchicus* cDNA clone CF_W00_41f11; 5' similar to ref|XP_786719.1| DIVDICTED: similar to MGC80576 protein [Strongylocentrotus. Score = 114 Length = 719; score = 40.1 bits (18), expect = 0.83; identities = 21/24 (87%); strand = Plus / Plus

```
Query: 23 ttgcttgattcacttcwwyaagag 46
          ||||||||||||||||   |||||
Sbjct: 9  ttgcttgattcacttctacaagag 32
```

>EL773927|EL773927.1 CF_W00_41f07_SP6 Copepod Whole Organism, Normalized *Calanus finmarchicus* cDNA clone CF_W00_41f07; 5' , mRNA sequence. Length = 697; score = 40.1 bits (18), expect = 0.83; identities = 21/24 (87%); strand = Plus / Plus

```
Query: 23 ttgcttgattcacttcwwyaagag 46
          ||||||||||||||||   |||||
Sbjct: 36 ttgcttgattcacttcttcaagag 59
```

>EL773888|EL773888.1 CF_W00_41c02_SP6 Copepod Whole Organism, Normalized *Calanus finmarchicus* cDNA clone CF_W00_41c02; 5' similar to gb|AAW26012.1| unknown - Schistosoma japonicum. Score = 360 bits (923), expect = 5e-98, m Length = 704; score = 40.1 bits (18), expect = 0.83; identities = 21/24 (87%); strand = Plus / Plus

```
Query: 23 ttgcttgattcacttcwwyaagag 46
          ||||||||||||||||   |||||
Sbjct: 10 ttgcttgattcacttctacaagag 33
```

>EL773877|EL773877.1 CF_W00_41b03_SP6 Copepod Whole Organism, Normalized *Calanus finmarchicus* cDNA clone CF_W00_41b03; 5' similar to ref|XP_967903.1| DIVDICTED: similar to Bys protein - Tribolium castaneum. Score = 62.8 bi Length = 391; score = 40.1 bits (18), expect = 0.83; identities = 21/24 (87%); strand = Plus / Plus

```
Query: 23 ttgcttgattcacttcwwyaagag 46
          ||||||||||||||||   |||||
Sbjct: 4  ttgcttgattcacttctacaagag 27
```

>EL773857|EL773857.1 CF_W00_40h05_SP6 Copepod Whole Organism, Normalized *Calanus finmarchicus* cDNA clone CF_W00_40h05; 5' similar to ref|XP_001121499.1| DIVDICTED: similar to CG15786-PA - Apis mellifera. Score = 236 bits (Length = 652; score = 40.1 bits (18), expect = 0.83; identities = 21/24 (87%); strand = Plus / Plus

```
Query: 23 ttgcttgattcacttcwwyaagag 46
          ||||||||||||||||   |||||
Sbjct: 9  ttgcttgattcacttctacaagag 32
```

>EL773852|EL773852.1 CF_WO0_40g12_SP6 Copepod Whole Organism, Normalized *Calanus finmarchicus* cDNA clone CF_WO0_40g12; 5' similar to gb|EAT38719.1| NADH-ubiquinone oxidoreductase 39 kda subunit - Aedes aegypti. Score = 193 Length = 657; score = 40.1 bits (18), expect = 0.83; identities = 21/24 (87%); strand = Plus / Plus

```
Query: 23 ttgcttgattcacttcwwyaagag 46
          ||||||||||||||||   |||||
Sbjct: 7  ttgcttgattcacttctacaagag 30
```

>EL773740|EL773740.1 CF_WO0_39f04_SP6 Copepod Whole Organism, Normalized *Calanus finmarchicus* cDNA clone CF_WO0_39f04; 5' , mRNA sequence. Length = 721; score = 40.1 bits (18), expect = 0.83; identities = 21/24 (87%); strand = Plus / Plus

```
Query: 23 ttgcttgattcacttcwwyaagag 46
          ||||||||||||||||   |||||
Sbjct: 14 ttgcttgattcacttctacaagag 37
```

>EL773729|EL773729.1 CF_WO0_39e05_SP6 Copepod Whole Organism, Normalized *Calanus finmarchicus* cDNA clone CF_WO0_39e05; 5' , mRNA sequence. Length = 624; score = 40.1 bits (18), expect = 0.83; identities = 21/24 (87%); strand = Plus / Plus

```
Query: 23 ttgcttgattcacttcwwyaagag 46
          ||||||||||||||||   |||||
Sbjct: 4  ttgcttgattcacttctacaagag 27
```

>EL773704|EL773704.1 CF_WO0_39c04_SP6 Copepod Whole Organism, Normalized *Calanus finmarchicus* cDNA clone CF_WO0_39c04; 5' similar to gb|AAH45222.1| Thiolase-prov protein - Xenopus laevis. Score = 291 bits (745), expect = 2 Length = 688; score = 40.1 bits (18), expect = 0.83; identities = 21/24 (87%); strand = Plus / Plus

```
Query: 23 ttgcttgattcacttcwwyaagag 46
          ||||||||||||||||   |||||
Sbjct: 3  ttgcttgattcacttctacaagag 26
```

>EL773680|EL773680.1 CF_WO0_39a04_SP6 Copepod Whole Organism, Normalized *Calanus finmarchicus* cDNA clone CF_WO0_39a04; 5' , mRNA sequence. Length = 589; score = 40.1 bits (18), expect = 0.83; identities = 21/24 (87%); strand = Plus / Plus

```
Query: 23 ttgcttgattcacttcwwyaagag 46
          ||||||||||||||||   |||||
Sbjct: 10 ttgcttgattcacttctacaagag 33
```

>EL773659|EL773659.1 CF_WO0_38g07_SP6 Copepod Whole Organism, Normalized *Calanus finmarchicus* cDNA clone CF_WO0_38g07; 5' similar to ref|XP_880178.2| DIVDICTED: hypothetical protein isoform 3 - Bos taurus. Score = 248 bits Length = 706; score = 40.1 bits (18), expect = 0.83; identities = 21/24 (87%); strand = Plus / Plus

```
Query: 23 ttgcttgattcacttcwwyaagag 46
          ||||||||||||||||   |||||
```

Sbjct: 2 ttgcttgattcacttctacaagag 25

>EL773639|EL773639.1 CF_W00_38e11_SP6 Copepod Whole Organism, Normalized *Calanus finmarchicus* cDNA clone CF_W00_38e11; 5' similar to ref|XP_321804.3| ENSANGP00000022498 - Anopheles gambiae str. PEST. Score = 291 bits (745) Length = 655; score = 40.1 bits (18), expect = 0.83; identities = 21/24 (87%); strand = Plus / Plus

```
Query: 23 ttgcttgattcacttcwwyaagag 46
          ||||||||||||||||   |||||
Sbjct: 10 ttgcttgattcacttctacaagag 33
```

>EL773637|EL773637.1 CF_W00_38e09_SP6 Copepod Whole Organism, Normalized *Calanus finmarchicus* cDNA clone CF_W00_38e09; 5' similar to gb|AAH75179.1| Polr3c-prov protein - Xenopus laevis. Score = 97.4 bits (241), expect = 5e Length = 663; score = 40.1 bits (18), expect = 0.83; identities = 21/24 (87%); strand = Plus / Plus

```
Query: 23 ttgcttgattcacttcwwyaagag 46
          ||||||||||||||||   |||||
Sbjct: 14 ttgcttgattcacttcttcaagag 37
```

>EL773634|EL773634.1 CF_W00_38e06_SP6 Copepod Whole Organism, Normalized *Calanus finmarchicus* cDNA clone CF_W00_38e06; 5' similar to gb|AAH62498.1| Ddx47-prov protein - Xenopus tropicalis. Score = 277 bits (709), expect = Length = 717; score = 40.1 bits (18), expect = 0.83; identities = 21/24 (87%); strand = Plus / Plus

```
Query: 23 ttgcttgattcacttcwwyaagag 46
          ||||||||||||||||   |||||
Sbjct: 13 ttgcttgattcacttctacaagag 36
```

>EL773627|EL773627.1 CF_W00_38d11_SP6 Copepod Whole Organism, Normalized *Calanus finmarchicus* cDNA clone CF_W00_38d11; 5' similar to ref|XP_001344937.1| DIVDICTED: hypothetical protein - Danio rerio. Score = 223 bits (568) Length = 721; score = 40.1 bits (18), expect = 0.83; identities = 21/24 (87%); strand = Plus / Plus

```
Query: 23 ttgcttgattcacttcwwyaagag 46
          ||||||||||||||||   |||||
Sbjct: 9  ttgcttgattcacttctacaagag 32
```

>EL773618|EL773618.1 CF_W00_38d02_SP6 Copepod Whole Organism, Normalized *Calanus finmarchicus* cDNA clone CF_W00_38d02; 5' similar to ref|XP_001358355.1| GA14711-PA - Drosophila pseudoobscura. Score = 59.7 bits (143), Expec Length = 647; score = 40.1 bits (18), expect = 0.83; identities = 21/24 (87%); strand = Plus / Plus

```
Query: 23 ttgcttgattcacttcwwyaagag 46
          ||||||||||||||||   |||||
Sbjct: 7  ttgcttgattcacttctacaagag 30
```

>EL773611|EL773611.1 CF_W00_38c07_SP6 Copepod Whole Organism, Normalized *Calanus finmarchicus* cDNA clone CF_W00_38c07; 5' similar to gb|EAT44237.1| casein kinase - Aedes aegypti. Score =

374 bits (960), expect = e-102, mRN Length = 694; score = 40.1 bits (18), expect = 0.83; identities = 21/24 (87%); strand = Plus / Plus

```
Query: 23 ttgcttgattcacttcwwyaagag 46
          ||||||||||||||||   |||||
Sbjct: 9  ttgcttgattcacttctacaagag 32
```

>EL773596|EL773596.1 CF_W00_38b04_SP6 Copepod Whole Organism, Normalized *Calanus finmarchicus* cDNA clone CF_W00_38b04; 5' similar to ref|NP_648998.2| CG7510-PA - Drosophila melanogaster. Score = 189 bits (479), expect = 2e Length = 734; score = 40.1 bits (18), expect = 0.83; identities = 21/24 (87%); strand = Plus / Plus

```
Query: 23 ttgcttgattcacttcwwyaagag 46
          ||||||||||||||||   |||||
Sbjct: 9  ttgcttgattcacttctacaagag 32
```

>EL773585|EL773585.1 CF_W00_38a05_SP6 Copepod Whole Organism, Normalized *Calanus finmarchicus* cDNA clone CF_W00_38a05; 5' similar to ref|XP_970198.1| DIVDICTED: similar to CG7162-PA - Tribolium castaneum. Score = 126 bits Length = 651; score = 40.1 bits (18), expect = 0.83; identities = 21/24 (87%); strand = Plus / Plus

```
Query: 23 ttgcttgattcacttcwwyaagag 46
          ||||||||||||||||   |||||
Sbjct: 7  ttgcttgattcacttctacaagag 30
```

>EL773583|EL773583.1 CF_W00_38a03_SP6 Copepod Whole Organism, Normalized *Calanus finmarchicus* cDNA clone CF_W00_38a03; 5' similar to ref|XP_394940.2| DIVDICTED: similar to Probable cleavage and polyadenylation. Score = 313 Length = 691; score = 40.1 bits (18), expect = 0.83; identities = 21/24 (87%); strand = Plus / Plus

```
Query: 23 ttgcttgattcacttcwwyaagag 46
          ||||||||||||||||   |||||
Sbjct: 11 ttgcttgattcacttctacaagag 34
```

>EL773556|EL773556.1 CF_W00_37f12_SP6 Copepod Whole Organism, Normalized *Calanus finmarchicus* cDNA clone CF_W00_37f12; 5', mRNA sequence. Length = 700; score = 40.1 bits (18), expect = 0.83; identities = 21/24 (87%); strand = Plus / Plus

```
Query: 23 ttgcttgattcacttcwwyaagag 46
          ||||||||||||||||   |||||
Sbjct: 14 ttgcttgattcacttctacaagag 37
```

>EL773553|EL773553.1 CF_W00_37f09_SP6 Copepod Whole Organism, Normalized *Calanus finmarchicus* cDNA clone CF_W00_37f09; 5' similar to ref|XP_972674.1| DIVDICTED: similar to CG4760-PB, isoform B - Tribolium castaneum. Score Length = 710; score = 40.1 bits (18), expect = 0.83; identities = 21/24 (87%); strand = Plus / Plus

```
Query: 23 ttgcttgattcacttcwwyaagag 46
          ||||||||||||||||   |||||
```

Sbjct: 4 ttgcttgattcacttctacaagag 27

>EL773513|EL773513.1 CF_W00_37c03_SP6 Copepod Whole Organism, Normalized *Calanus finmarchicus* cDNA clone CF_W00_37c03; 5’ similar to ref|XP_797579.2| DIVDICTED: similar to glycogen debranching enzyme, partial. Score = 237 Length = 730; score = 40.1 bits (18), expect = 0.83; identities = 21/24 (87%); strand = Plus / Plus

Query: 23 ttgcttgattcacttcwwyaagag 46
          |||||||||||||||| |||||
Sbjct: 2 ttgcttgattcacttctacaagag 25

>EL773497|EL773497.1 CF_W00_37a11_SP6 Copepod Whole Organism, Normalized *Calanus finmarchicus* cDNA clone CF_W00_37a11; 5’ , mRNA sequence. Length = 655; score = 40.1 bits (18), expect = 0.83; identities = 21/24 (87%); strand = Plus / Plus

Query: 23 ttgcttgattcacttcwwyaagag 46
          |||||||||||||||| |||||
Sbjct: 15 ttgcttgattcacttctacaagag 38

>EL773487|EL773487.1 CF_W00_37a01_SP6 Copepod Whole Organism, Normalized *Calanus finmarchicus* cDNA clone CF_W00_37a01; 5’ similar to ref|NP_001016296.1| hypothetical protein LOC549050 - Xenopus tropicalis. Score = 106 bits Length = 676; score = 40.1 bits (18), expect = 0.83; identities = 21/24 (87%); strand = Plus / Plus

Query: 23 ttgcttgattcacttcwwyaagag 46
          |||||||||||||||| |||||
Sbjct: 15 ttgcttgattcacttctacaagag 38

>EL773480|EL773480.1 CF_W00_36h06_SP6 Copepod Whole Organism, Normalized *Calanus finmarchicus* cDNA clone CF_W00_36h06; 5’ similar to ref|XP_522200.2| DIVDICTED: trehalase (brush-border membrane glycoprotein) isoform 2. Sco Length = 712; score = 40.1 bits (18), expect = 0.83; identities = 21/24 (87%); strand = Plus / Plus

Query: 23 ttgcttgattcacttcwwyaagag 46
          |||||||||||||||| |||||
Sbjct: 2 ttgcttgattcacttctacaagag 25

>EL773455|EL773455.1 CF_W00_36f05_SP6 Copepod Whole Organism, Normalized *Calanus finmarchicus* cDNA clone CF_W00_36f05; 5’ similar to ref|XP_969681.1| DIVDICTED: similar to CG16941-PA - Tribolium castaneum. Score = 290 bits Length = 688; score = 40.1 bits (18), expect = 0.83; identities = 21/24 (87%); strand = Plus / Plus

Query: 23 ttgcttgattcacttcwwyaagag 46
          |||||||||||||||| |||||
Sbjct: 11 ttgcttgattcacttctacaagag 34

>EL773444|EL773444.1 CF_W00_36e05_SP6 Copepod Whole Organism, Normalized *Calanus finmarchicus* cDNA clone CF_W00_36e05; 5’ similar to ref|XP_966892.1| DIVDICTED: similar to CG9009-PA -

Tribolium castaneum. Score = 118 bits Length = 662; score = 40.1 bits (18), expect = 0.83; identities = 21/24 (87%); strand = Plus / Plus

```
Query: 23 ttgcttgattcacttcwwyaagag 46
          ||||||||||||||||   |||||
Sbjct: 14 ttgcttgattcacttctacaagag 37
```

>EL773434|EL773434.1 CF_W00_36d06_SP6 Copepod Whole Organism, Normalized *Calanus finmarchicus* cDNA clone CF_W00_36d06; 5' similar to ref|XP_968281.1| DIVDICTED: similar to CG4422-PA - Tribolium castaneum. Score = 338 bits Length = 685; score = 40.1 bits (18), expect = 0.83; identities = 21/24 (87%); strand = Plus / Plus

```
Query: 23 ttgcttgattcacttcwwyaagag 46
          ||||||||||||||||   |||||
Sbjct: 15 ttgcttgattcacttctacaagag 38
```

>EL773396|EL773396.1 CF_W00_36a04_SP6 Copepod Whole Organism, Normalized *Calanus finmarchicus* cDNA clone CF_W00_36a04; 5' similar to ref|NP_524274.1| Replication Protein A 70 CG9633-PA - Drosophila melanogaster. Score = 13 Length = 651; score = 40.1 bits (18), expect = 0.83; identities = 21/24 (87%); strand = Plus / Plus

```
Query: 23 ttgcttgattcacttcwwyaagag 46
          ||||||||||||||||   |||||
Sbjct: 8  ttgcttgattcacttctacaagag 31
```

>EL773351|EL773351.1 CF_W00_35e07_SP6 Copepod Whole Organism, Normalized *Calanus finmarchicus* cDNA clone CF_W00_35e07; 5' similar to ref|XP_971746.1| DIVDICTED: similar to PR domain containing 5 - Tribolium castaneum. Scor Length = 742; score = 40.1 bits (18), expect = 0.83; identities = 21/24 (87%); strand = Plus / Plus

```
Query: 23 ttgcttgattcacttcwwyaagag 46
          ||||||||||||||||   |||||
Sbjct: 8  ttgcttgattcacttctacaagag 31
```

>EL773338|EL773338.1 CF_W00_35d06_SP6 Copepod Whole Organism, Normalized *Calanus finmarchicus* cDNA clone CF_W00_35d06; 5' similar to ref|NP_001008637.1| hypothetical protein LOC494094 - Danio rerio. Score = 309 bits (792), Length = 642; score = 40.1 bits (18), expect = 0.83; identities = 21/24 (87%); strand = Plus / Plus

```
Query: 23 ttgcttgattcacttcwwyaagag 46
          ||||||||||||||||   |||||
Sbjct: 2  ttgcttgattcacttctacaagag 25
```

>EL773314|EL773314.1 CF_W00_35b06_SP6 Copepod Whole Organism, Normalized *Calanus finmarchicus* cDNA clone CF_W00_35b06; 5' similar to ref|XP_001355111.1| GA22116-PA - Drosophila pseudoobscura. Score = 217 bits (552), expect Length = 733; score = 40.1 bits (18), expect = 0.83; identities = 21/24 (87%); strand = Plus / Plus

```
Query: 23 ttgcttgattcacttcwwyaagag 46
```

|||||||||||||||| |||||
Sbjct: 38 ttgcttgattcacttctacaagag 61

>EL697524|EL697524.1 CF_WO0_34h07_SP6 Copepod Whole Organism, Normalized *Calanus finmarchicus* cDNA clone CF_WO0_34h07; 5' , mRNA sequence. Length = 672; score = 40.1 bits (18), expect = 0.83; identities = 21/24 (87%); strand = Plus / Plus

Query: 23 ttgcttgattcacttcwwyaagag 46
|||||||||||||||| |||||
Sbjct: 14 ttgcttgattcacttctacaagag 37

>EL697515|EL697515.1 CF_WO0_34g09_SP6 Copepod Whole Organism, Normalized *Calanus finmarchicus* cDNA clone CF_WO0_34g09; 5' similar to ref|XP_976083.1| DIVDICTED: similar to CG6016-PB, isoform B isoform 2 [Tribolium. Score = Length = 711; score = 40.1 bits (18), expect = 0.83; identities = 21/24 (87%); strand = Plus / Plus

Query: 23 ttgcttgattcacttcwwyaagag 46
|||||||||||||||| |||||
Sbjct: 14 ttgcttgattcacttctacaagag 37

>EL697494|EL697494.1 CF_WO0_34e11_SP6 Copepod Whole Organism, Normalized *Calanus finmarchicus* cDNA clone CF_WO0_34e11; 5' , mRNA sequence. Length = 679; score = 40.1 bits (18), expect = 0.83; identities = 21/24 (87%); strand = Plus / Plus

Query: 23 ttgcttgattcacttcwwyaagag 46
|||||||||||||||| |||||
Sbjct: 9 ttgcttgattcacttctacaagag 32

>EL697476|EL697476.1 CF_WO0_34d05_SP6 Copepod Whole Organism, Normalized *Calanus finmarchicus* cDNA clone CF_WO0_34d05; 5' , mRNA sequence. Length = 709; score = 40.1 bits (18), expect = 0.83; identities = 21/24 (87%); strand = Plus / Plus

Query: 23 ttgcttgattcacttcwwyaagag 46
|||||||||||||||| |||||
Sbjct: 9 ttgcttgattcacttctacaagag 32

>EL697465|EL697465.1 CF_WO0_34c06_SP6 Copepod Whole Organism, Normalized *Calanus finmarchicus* cDNA clone CF_WO0_34c06; 5' , mRNA sequence. Length = 709; score = 40.1 bits (18), expect = 0.83; identities = 21/24 (87%); strand = Plus / Plus

Query: 23 ttgcttgattcacttcwwyaagag 46
|||||||||||||||| |||||
Sbjct: 10 ttgcttgattcacttcttcaagag 33

>EL697428|EL697428.1 CF_WO0_33h04_SP6 Copepod Whole Organism, Normalized *Calanus finmarchicus* cDNA clone CF_WO0_33h04; 5' , mRNA sequence. Length = 667; score = 40.1 bits (18), expect = 0.83; identities = 21/24 (87%); strand = Plus / Plus

Query: 23 ttgcttgattcacttcwwyaagag 46

```
         |||||||||||||||   |||||
Sbjct: 5  ttgcttgattcacttctacaagag 28
```

>EL697427|EL697427.1 CF_WO0_33h03_SP6 Copepod Whole Organism, Normalized *Calanus finmarchicus* cDNA clone CF_WO0_33h03; 5' similar to ref|XP_976077.1| DIVDICTED: similar to CG8433-PB, isoform B isoform 2 [Tribolium. Score = Length = 665; score = 40.1 bits (18), expect = 0.83; identities = 21/24 (87%); strand = Plus / Plus

```
Query: 23 ttgcttgattcacttcwwyaagag 46
          |||||||||||||||   |||||
Sbjct: 15 ttgcttgattcacttctacaagag 38
```

>EL697389|EL697389.1 CF_WO0_33e01_SP6 Copepod Whole Organism, Normalized *Calanus finmarchicus* cDNA clone CF_WO0_33e01; 5' similar to ref|XP_969343.1| DIVDICTED: similar to CG1972-PA - Tribolium castaneum. Score = 430 bits Length = 733; score = 40.1 bits (18), expect = 0.83; identities = 21/24 (87%); strand = Plus / Plus

```
Query: 23 ttgcttgattcacttcwwyaagag 46
          |||||||||||||||   |||||
Sbjct: 10 ttgcttgattcacttctacaagag 33
```

>EL697363|EL697363.1 CF_WO0_33b11_SP6 Copepod Whole Organism, Normalized *Calanus finmarchicus* cDNA clone CF_WO0_33b11; 5' similar to ref|XP_623894.1| DIVDICTED: similar to CG7942-PA, isoform A - Apis mellifera. Score = 182 Length = 487; score = 40.1 bits (18), expect = 0.83; identities = 21/24 (87%); strand = Plus / Plus

```
Query: 23 ttgcttgattcacttcwwyaagag 46
          |||||||||||||||   |||||
Sbjct: 7  ttgcttgattcacttctacaagag 30
```

>EL697324|EL697324.1 CF_WO0_32g08_SP6 Copepod Whole Organism, Normalized *Calanus finmarchicus* cDNA clone CF_WO0_32g08; 5' , mRNA sequence. Length = 699; score = 40.1 bits (18), expect = 0.83; identities = 21/24 (87%); strand = Plus / Plus

```
Query: 23 ttgcttgattcacttcwwyaagag 46
          |||||||||||||||   |||||
Sbjct: 10 ttgcttgattcacttctacaagag 33
```

>EL697323|EL697323.1 CF_WO0_32g07_SP6 Copepod Whole Organism, Normalized *Calanus finmarchicus* cDNA clone CF_WO0_32g07; 5' similar to ref|XP_395026.2| DIVDICTED: similar to CG2938-PB - Apis mellifera. Score = 155 bits (392) Length = 718; score = 40.1 bits (18), expect = 0.83; identities = 21/24 (87%); strand = Plus / Plus

```
Query: 23 ttgcttgattcacttcwwyaagag 46
          |||||||||||||||   |||||
Sbjct: 9  ttgcttgattcacttctacaagag 32
```

>EL697312|EL697312.1 CF_WO0_32f08_SP6 Copepod Whole Organism, Normalized *Calanus finmarchicus* cDNA clone CF_WO0_32f08; 5' , mRNA sequence. Length = 682; score = 40.1 bits (18), expect = 0.83; identities = 21/24 (87%); strand = Plus / Plus

```
Query: 23 ttgcttgattcacttcwwyaagag 46
          ||||||||||||||||   |||||
Sbjct: 15 ttgcttgattcacttctacaagag 38
```

>EL697297|EL697297.1 CF_WO0_32e05_SP6 Copepod Whole Organism, Normalized *Calanus finmarchicus* cDNA clone CF_WO0_32e05; 5' similar to gb|ABI63548.1| receptor for activated protein kinase C-like - Blattella germanica. Score Length = 686; score = 40.1 bits (18), expect = 0.83; identities = 21/24 (87%); strand = Plus / Plus

```
Query: 23 ttgcttgattcacttcwwyaagag 46
          ||||||||||||||||   |||||
Sbjct: 9  ttgcttgattcacttctacaagag 32
```

>EL697227|EL697227.1 CF_WO0_31g05_SP6 Copepod Whole Organism, Normalized *Calanus finmarchicus* cDNA clone CF_WO0_31g05; 5' similar to ref|XP_966433.1| DIVDICTED: similar to CG11140-PI, isoform I isoform 1 [Tribolium. Score Length = 696; score = 40.1 bits (18), expect = 0.83; identities = 21/24 (87%); strand = Plus / Plus

```
Query: 23 ttgcttgattcacttcwwyaagag 46
          ||||||||||||||||   |||||
Sbjct: 2  ttgcttgattcacttctacaagag 25
```

>EL697179|EL697179.1 CF_WO0_31c04_SP6 Copepod Whole Organism, Normalized *Calanus finmarchicus* cDNA clone CF_WO0_31c04; 5' similar to emb|CAG10633.1| unnamed protein product - Tetraodon nigroviridis. Score = 130 bits (326), Length = 638; score = 40.1 bits (18), expect = 0.83; identities = 21/24 (87%); strand = Plus / Plus

```
Query: 23 ttgcttgattcacttcwwyaagag 46
          ||||||||||||||||   |||||
Sbjct: 10 ttgcttgattcacttctacaagag 33
```

>EL697174|EL697174.1 CF_WO0_31b11_SP6 Copepod Whole Organism, Normalized *Calanus finmarchicus* cDNA clone CF_WO0_31b11; 5' , mRNA sequence. Length = 518; score = 40.1 bits (18), expect = 0.83; identities = 21/24 (87%); strand = Plus / Plus

```
Query: 23 ttgcttgattcacttcwwyaagag 46
          ||||||||||||||||   |||||
Sbjct: 10 ttgcttgattcacttctacaagag 33
```

>EL697154|EL697154.1 CF_WO0_31a03_SP6 Copepod Whole Organism, Normalized *Calanus finmarchicus* cDNA clone CF_WO0_31a03; 5' similar to ref|XP_625202.1| DIVDICTED: similar to Brahma associated protein 55kD CG6546-PA. Score = Length = 697; score = 40.1 bits (18), expect = 0.83; identities = 21/24 (87%); strand = Plus / Plus

```
Query: 23 ttgcttgattcacttcwwyaagag 46
```

||||||||||||||| |||||
Sbjct: 34 ttgcttgattcacttctacaagag 57

>EL697130|EL697130.1 CF_WO0_30g03_SP6 Copepod Whole Organism, Normalized *Calanus finmarchicus* cDNA clone CF_WO0_30g03; 5' similar to ref|XP_309979.3| ENSANGP00000015940 - Anopheles gambiae str. PEST. Score = 156 bits (394) Length = 659; score = 40.1 bits (18), expect = 0.83; identities = 21/24 (87%); strand = Plus / Plus

Query: 23 ttgcttgattcacttcwwyaagag 46
||||||||||||||| |||||
Sbjct: 6 ttgcttgattcacttctacaagag 29

>EL697129|EL697129.1 CF_WO0_30g02_SP6 Copepod Whole Organism, Normalized *Calanus finmarchicus* cDNA clone CF_WO0_30g02; 5' similar to gb|AAI27434.1| Unknown (protein for MGC:154900) - Xenopus laevis. Score = 88.2 bits (217) Length = 678; score = 40.1 bits (18), expect = 0.83; identities = 21/24 (87%); strand = Plus / Plus

Query: 23 ttgcttgattcacttcwwyaagag 46
||||||||||||||| |||||
Sbjct: 25 ttgcttgattcacttctacaagag 48

>EL697115|EL697115.1 CF_WO0_30e12_SP6 Copepod Whole Organism, Normalized *Calanus finmarchicus* cDNA clone CF_WO0_30e12; 5', mRNA sequence. Length = 701; score = 40.1 bits (18), expect = 0.83; identities = 21/24 (87%); strand = Plus / Plus

Query: 23 ttgcttgattcacttcwwyaagag 46
||||||||||||||| |||||
Sbjct: 12 ttgcttgattcacttctacaagag 35

>EL697105|EL697105.1 CF_WO0_30e02_SP6 Copepod Whole Organism, Normalized *Calanus finmarchicus* cDNA clone CF_WO0_30e02; 5', mRNA sequence. Length = 702; score = 40.1 bits (18), expect = 0.83; identities = 21/24 (87%); strand = Plus / Plus

Query: 23 ttgcttgattcacttcwwyaagag 46
||||||||||||||| |||||
Sbjct: 8 ttgcttgattcacttctacaagag 31

>EL697100|EL697100.1 CF_WO0_30d09_SP6 Copepod Whole Organism, Normalized *Calanus finmarchicus* cDNA clone CF_WO0_30d09; 5', mRNA sequence. Length = 626; score = 40.1 bits (18), expect = 0.83; identities = 21/24 (87%); strand = Plus / Plus

Query: 23 ttgcttgattcacttcwwyaagag 46
||||||||||||||| |||||
Sbjct: 13 ttgcttgattcacttctacaagag 36

>EL697090|EL697090.1 CF_WO0_30c11_SP6 Copepod Whole Organism, Normalized *Calanus finmarchicus* cDNA clone CF_WO0_30c11; 5' similar to emb|CAM16186.1| novel protein (zgc:85685) - Danio rerio. Score = 289 bits (739), expect = Length = 708; score = 40.1 bits (18), expect = 0.83; identities = 21/24 (87%); strand = Plus / Plus

```
Query: 23 ttgcttgattcacttcwwyaagag 46
          ||||||||||||||||   |||||
Sbjct: 8  ttgcttgattcacttctacaagag 31
```

>EL697087|EL697087.1 CF_W00_30c08_SP6 Copepod Whole Organism, Normalized *Calanus finmarchicus* cDNA clone CF_W00_30c08; 5' , mRNA sequence. Length = 705; score = 40.1 bits (18), expect = 0.83; identities = 21/24 (87%); strand = Plus / Plus

```
Query: 23 ttgcttgattcacttcwwyaagag 46
          ||||||||||||||||   |||||
Sbjct: 9  ttgcttgattcacttctacaagag 32
```

>EL697084|EL697084.1 CF_W00_30c05_SP6 Copepod Whole Organism, Normalized *Calanus finmarchicus* cDNA clone CF_W00_30c05; 5' similar to ref|XP_001352580.1| GA18259-PA - Drosophila pseudoobscura. Score = 200 bits (509), expect Length = 673; score = 40.1 bits (18), expect = 0.83; identities = 21/24 (87%); strand = Plus / Plus

```
Query: 23 ttgcttgattcacttcwwyaagag 46
          ||||||||||||||||   |||||
Sbjct: 8  ttgcttgattcacttctacaagag 31
```

>EL697077|EL697077.1 CF_W00_30b10_SP6 Copepod Whole Organism, Normalized *Calanus finmarchicus* cDNA clone CF_W00_30b10; 5' similar to gb|AAO15914.1|AF510054_1 vasa-like - Schistocerca gregaria. Score = 219 bits (557), Expec Length = 712; score = 40.1 bits (18), expect = 0.83; identities = 21/24 (87%); strand = Plus / Plus

```
Query: 23 ttgcttgattcacttcwwyaagag 46
          ||||||||||||||||   |||||
Sbjct: 8  ttgcttgattcacttctacaagag 31
```

>EL697070|EL697070.1 CF_W00_30b03_SP6 Copepod Whole Organism, Normalized *Calanus finmarchicus* cDNA clone CF_W00_30b03; 5' similar to ref|XP_780362.2| DIVDICTED: similar to Golgi-associated microtubule-binding protein. Scor Length = 639; score = 40.1 bits (18), expect = 0.83; identities = 21/24 (87%); strand = Plus / Plus

```
Query: 23 ttgcttgattcacttcwwyaagag 46
          ||||||||||||||||   |||||
Sbjct: 8  ttgcttgattcacttctacaagag 31
```

>EL697069|EL697069.1 CF_W00_30b02_SP6 Copepod Whole Organism, Normalized *Calanus finmarchicus* cDNA clone CF_W00_30b02; 5' similar to ref|XP_001121180.1| DIVDICTED: similar to MAK10 homolog, amino-acid N-acetyltransferase. Length = 626; score = 40.1 bits (18), expect = 0.83; identities = 21/24 (87%); strand = Plus / Plus

```
Query: 23 ttgcttgattcacttcwwyaagag 46
          ||||||||||||||||   |||||
Sbjct: 12 ttgcttgattcacttctacaagag 35
```

>EL697051|EL697051.1 CF_WO0_29h08_SP6 Copepod Whole Organism, Normalized *Calanus finmarchicus* cDNA clone CF_WO0_29h08; 5' similar to ref|XP_967673.1| DIVDICTED: similar to CG10370-PA - Tribolium castaneum. Score = 232 bits Length = 663; score = 40.1 bits (18), expect = 0.83; identities = 21/24 (87%); strand = Plus / Plus

```
Query: 23 ttgcttgattcacttcwwyaagag 46
          ||||||||||||||||   |||||
Sbjct: 3  ttgcttgattcacttctacaagag 26
```

>EL697018|EL697018.1 CF_WO0_29e11_SP6 Copepod Whole Organism, Normalized *Calanus finmarchicus* cDNA clone CF_WO0_29e11; 5' similar to ref|XP_315109.2| ENSANGP00000011450 - Anopheles gambiae str. PEST. Score = 210 bits (535) Length = 645; score = 40.1 bits (18), expect = 0.83; identities = 21/24 (87%); strand = Plus / Plus

```
Query: 23 ttgcttgattcacttcwwyaagag 46
          ||||||||||||||||   |||||
Sbjct: 10 ttgcttgattcacttctacaagag 33
```

>EL696990|EL696990.1 CF_WO0_29c07_SP6 Copepod Whole Organism, Normalized *Calanus finmarchicus* cDNA clone CF_WO0_29c07; 5', mRNA sequence. Length = 643; score = 40.1 bits (18), expect = 0.83; identities = 21/24 (87%); strand = Plus / Plus

```
Query: 23 ttgcttgattcacttcwwyaagag 46
          ||||||||||||||||   |||||
Sbjct: 5  ttgcttgattcacttctacaagag 28
```

>EL696981|EL696981.1 CF_WO0_29b10_SP6 Copepod Whole Organism, Normalized *Calanus finmarchicus* cDNA clone CF_WO0_29b10; 5' similar to ref|XP_792543.2| DIVDICTED: similar to DHX33 protein - Strongylocentrotus purpuratus. Sco Length = 741; score = 40.1 bits (18), expect = 0.83; identities = 21/24 (87%); strand = Plus / Plus

```
Query: 23 ttgcttgattcacttcwwyaagag 46
          ||||||||||||||||   |||||
Sbjct: 10 ttgcttgattcacttctacaagag 33
```

>EL696946|EL696946.1 CF_WO0_28g08_SP6 Copepod Whole Organism, Normalized *Calanus finmarchicus* cDNA clone CF_WO0_28g08; 5' similar to ref|XP_624707.1| DIVDICTED: similar to CG6904-PA, isoform A - Apis mellifera. Score = 253 Length = 713; score = 40.1 bits (18), expect = 0.83; identities = 21/24 (87%); strand = Plus / Plus

```
Query: 23 ttgcttgattcacttcwwyaagag 46
          ||||||||||||||||   |||||
Sbjct: 3  ttgcttgattcacttctacaagag 26
```

>EL696902|EL696902.1 CF_WO0_28c11_SP6 Copepod Whole Organism, Normalized *Calanus finmarchicus* cDNA clone CF_WO0_28c11; 5', mRNA sequence. Length = 680; score = 40.1 bits (18), expect = 0.83; identities = 21/24 (87%); strand = Plus / Plus

```
Query: 23 ttgcttgattcacttcwwyaagag 46
```

          ||||||||||||||| |||||
Sbjct: 10 ttgcttgattcacttctacaagag 33

>EL696862|EL696862.1 CF_WO0_27h05_SP6 Copepod Whole Organism, Normalized *Calanus finmarchicus* cDNA clone CF_WO0_27h05; 5' similar to ref|XP_623819.1| DIVDICTED: similar to MLF1-adaptor molecule CG1098-PA [Apis. Score = 295 Length = 717; score = 40.1 bits (18), expect = 0.83; identities = 21/24 (87%); strand = Plus / Plus

Query: 23 ttgcttgattcacttcwwyaagag 46
          ||||||||||||||| |||||
Sbjct: 12 ttgcttgattcacttctacaagag 35

>EL696857|EL696857.1 CF_WO0_27g12_SP6 Copepod Whole Organism, Normalized *Calanus finmarchicus* cDNA clone CF_WO0_27g12; 5' similar to gb|AAB35644.1| replication factors MCM - Drosophila sp.. Score = 146 bits (369), expect = Length = 718; score = 40.1 bits (18), expect = 0.83; identities = 21/24 (87%); strand = Plus / Plus

Query: 23 ttgcttgattcacttcwwyaagag 46
          ||||||||||||||| |||||
Sbjct: 13 ttgcttgattcacttctacaagag 36

>EL696842|EL696842.1 CF_WO0_27f09_SP6 Copepod Whole Organism, Normalized *Calanus finmarchicus* cDNA clone CF_WO0_27f09; 5' similar to ref|XP_394933.3| DIVDICTED: similar to Arginine methyltransferase 4 CG5358-PA [Apis. Scor Length = 672; score = 40.1 bits (18), expect = 0.83; identities = 21/24 (87%); strand = Plus / Plus

Query: 23 ttgcttgattcacttcwwyaagag 46
          ||||||||||||||| |||||
Sbjct: 11 ttgcttgattcacttctacaagag 34

>EL696826|EL696826.1 CF_WO0_27e05_SP6 Copepod Whole Organism, Normalized *Calanus finmarchicus* cDNA clone CF_WO0_27e05; 5' similar to ref|NP_650833.1| CG4662-PA, isoform A - Drosophila melanogaster. Score = 160 bits (404), Length = 630; score = 40.1 bits (18), expect = 0.83; identities = 21/24 (87%); strand = Plus / Plus

Query: 23 ttgcttgattcacttcwwyaagag 46
          ||||||||||||||| |||||
Sbjct: 11 ttgcttgattcacttctacaagag 34

>EL696820|EL696820.1 CF_WO0_27d11_SP6 Copepod Whole Organism, Normalized *Calanus finmarchicus* cDNA clone CF_WO0_27d11; 5' similar to ref|NP_496736.1| Glycogen SYnthase family member (gsy-1) - Caenorhabditis elegans. Score Length = 723; score = 40.1 bits (18), expect = 0.83; identities = 21/24 (87%); strand = Plus / Plus

Query: 23 ttgcttgattcacttcwwyaagag 46
          ||||||||||||||| |||||
Sbjct: 14 ttgcttgattcacttctacaagag 37

>EL696813|EL696813.1 CF_WO0_27d04_SP6 Copepod Whole Organism, Normalized *Calanus finmarchicus* cDNA clone CF_WO0_27d04; 5’ similar to ref|XP_001123261.1| DIVDICTED: similar to CG6070-PA, partial - Apis mellifera. Score = 15 Length = 706; score = 40.1 bits (18), expect = 0.83; identities = 21/24 (87%); strand = Plus / Plus

```
Query: 23 ttgcttgattcacttcwwyaagag 46
          ||||||||||||||||   |||||
Sbjct: 11 ttgcttgattcacttctacaagag 34
```

>EL696795|EL696795.1 CF_WO0_27b10_SP6 Copepod Whole Organism, Normalized *Calanus finmarchicus* cDNA clone CF_WO0_27b10; 5’ similar to ref|XP_969775.1| DIVDICTED: similar to CG4420-PA - Tribolium castaneum. Score = 199 bits Length = 662; score = 40.1 bits (18), expect = 0.83; identities = 21/24 (87%); strand = Plus / Plus

```
Query: 23 ttgcttgattcacttcwwyaagag 46
          ||||||||||||||||   |||||
Sbjct: 6  ttgcttgattcacttctacaagag 29
```

>EL696781|EL696781.1 CF_WO0_27a08_SP6 Copepod Whole Organism, Normalized *Calanus finmarchicus* cDNA clone CF_WO0_27a08; 5’ , mRNA sequence. Length = 687; score = 40.1 bits (18), expect = 0.83; identities = 21/24 (87%); strand = Plus / Plus

```
Query: 23 ttgcttgattcacttcwwyaagag 46
          ||||||||||||||||   |||||
Sbjct: 5  ttgcttgattcacttctacaagag 28
```

>EL696775|EL696775.1 CF_WO0_27a02_SP6 Copepod Whole Organism, Normalized *Calanus finmarchicus* cDNA clone CF_WO0_27a02; 5’ similar to ref|XP_853844.1| DIVDICTED: similar to nucleoporin like 2 - Canis familiaris. Score = 67. Length = 728; score = 40.1 bits (18), expect = 0.83; identities = 21/24 (87%); strand = Plus / Plus

```
Query: 23 ttgcttgattcacttcwwyaagag 46
          ||||||||||||||||   |||||
Sbjct: 10 ttgcttgattcacttctacaagag 33
```

>EL696770|EL696770.1 CF_WO0_26h09_SP6 Copepod Whole Organism, Normalized *Calanus finmarchicus* cDNA clone CF_WO0_26h09; 5’ similar to ref|XP_623365.1| DIVDICTED: similar to UBL3 CG9038-PA, isoform A isoform 2 [Apis. Score = Length = 702; score = 40.1 bits (18), expect = 0.83; identities = 21/24 (87%); strand = Plus / Plus

```
Query: 23 ttgcttgattcacttcwwyaagag 46
          ||||||||||||||||   |||||
Sbjct: 8  ttgcttgattcacttctacaagag 31
```

>EL696768|EL696768.1 CF_WO0_26h07_SP6 Copepod Whole Organism, Normalized *Calanus finmarchicus* cDNA clone CF_WO0_26h07; 5’ similar to ref|XP_395543.3| DIVDICTED: similar to dalao CG7055-PA - Apis mellifera. Score = 142 bits Length = 684; score = 40.1 bits (18), expect = 0.83; identities = 21/24 (87%); strand = Plus / Plus

Query: 23 ttgcttgattcacttcwwyaagag 46
         |||||||||||||||| |||||
Sbjct: 14 ttgcttgattcacttctacaagag 37

>EL696754|EL696754.1 CF_WO0_26g05_SP6 Copepod Whole Organism, Normalized *Calanus finmarchicus* cDNA clone CF_WO0_26g05; 5' , mRNA sequence. Length = 685; score = 40.1 bits (18), expect = 0.83; identities = 21/24 (87%); strand = Plus / Plus

Query: 23 ttgcttgattcacttcwwyaagag 46
         |||||||||||||||| |||||
Sbjct: 11 ttgcttgattcacttctacaagag 34

>EL696688|EL696688.1 CF_WO0_26a09_SP6 Copepod Whole Organism, Normalized *Calanus finmarchicus* cDNA clone CF_WO0_26a09; 5' , mRNA sequence. Length = 676; score = 40.1 bits (18), expect = 0.83; identities = 21/24 (87%); strand = Plus / Plus

Query: 23 ttgcttgattcacttcwwyaagag 46
         |||||||||||||||| |||||
Sbjct: 10 ttgcttgattcacttctacaagag 33

>EL696673|EL696673.1 CF_WO0_25h05_SP6 Copepod Whole Organism, Normalized *Calanus finmarchicus* cDNA clone CF_WO0_25h05; 5' similar to ref|NP_001017255.1| hypothetical protein LOC550009 - Xenopus tropicalis. Score = 210 bits Length = 706; score = 40.1 bits (18), expect = 0.83; identities = 21/24 (87%); strand = Plus / Plus

Query: 23 ttgcttgattcacttcwwyaagag 46
         |||||||||||||||| |||||
Sbjct: 41 ttgcttgattcacttctacaagag 64

>EL696652|EL696652.1 CF_WO0_25f08_SP6 Copepod Whole Organism, Normalized *Calanus finmarchicus* cDNA clone CF_WO0_25f08; 5' similar to ref|NP_001015637.1| DnaJ (Hsp40) homolog, subfamily A, member 1 - Bos taurus. Score = 228 Length = 651; score = 40.1 bits (18), expect = 0.83; identities = 21/24 (87%); strand = Plus / Plus

Query: 23 ttgcttgattcacttcwwyaagag 46
         |||||||||||||||| |||||
Sbjct: 9 ttgcttgattcacttctacaagag 32

>EL696632|EL696632.1 CF_WO0_25d12_SP6 Copepod Whole Organism, Normalized *Calanus finmarchicus* cDNA clone CF_WO0_25d12; 5' similar to ref|XP_312160.2| ENSANGP00000024201 - Anopheles gambiae str. PEST. Score = 328 bits (841) Length = 694; score = 40.1 bits (18), expect = 0.83; identities = 21/24 (87%); strand = Plus / Plus

Query: 23 ttgcttgattcacttcwwyaagag 46
         |||||||||||||||| |||||
Sbjct: 5 ttgcttgattcacttctacaagag 28

>EL696602|EL696602.1 CF_WO0_25b06_SP6 Copepod Whole Organism, Normalized *Calanus finmarchicus* cDNA clone CF_WO0_25b06; 5' similar to ref|XP_971096.1| DIVDICTED: similar to CG11851-PA -

Tribolium castaneum. Score = 183 bits Length = 735; score = 40.1 bits (18), expect = 0.83; identities = 21/24 (87%); strand = Plus / Plus

```
Query: 23 ttgcttgattcacttcwwyaagag 46
          ||||||||||||||||   |||||
Sbjct: 12 ttgcttgattcacttctacaagag 35
```

>EL586627|EL586627.1 CF_W00_24d09_SP6 Copepod Whole Organism, Normalized *Calanus finmarchicus* cDNA clone CF_W00_24d09; 5' similar to CAE65756.1 Hypothetical protein CBG10845 [Caenorhabditis briggsae]. Score=49.29, Expect=0 Length = 670; score = 40.1 bits (18), expect = 0.83; identities = 21/24 (87%); strand = Plus / Plus

```
Query: 23 ttgcttgattcacttcwwyaagag 46
          ||||||||||||||||   |||||
Sbjct: 9  ttgcttgattcacttctacaagag 32
```

>EL586602|EL586602.1 CF_W00_24b07_SP6 Copepod Whole Organism, Normalized *Calanus finmarchicus* cDNA clone CF_W00_24b07; 5' , mRNA sequence. Length = 691; score = 40.1 bits (18), expect = 0.83; identities = 21/24 (87%); strand = Plus / Plus

```
Query: 23 ttgcttgattcacttcwwyaagag 46
          ||||||||||||||||   |||||
Sbjct: 10 ttgcttgattcacttctacaagag 33
```

>EL586545|EL586545.1 CF_W00_23e09_SP6 Copepod Whole Organism, Normalized *Calanus finmarchicus* cDNA clone CF_W00_23e09; 5' similar to CAJ17217.1 ribosomal protein S21e [Sphaerius sp. APV-2005]. Score=137.5, Expect=1.10E-31, Length = 370; score = 40.1 bits (18), expect = 0.83; identities = 21/24 (87%); strand = Plus / Plus

```
Query: 23 ttgcttgattcacttcwwyaagag 46
          ||||||||||||||||   |||||
Sbjct: 9  ttgcttgattcacttctacaagag 32
```

>EL586541|EL586541.1 CF_W00_23e04_SP6 Copepod Whole Organism, Normalized *Calanus finmarchicus* cDNA clone CF_W00_23e04; 5' similar to XP_623947.1 Probable 26S proteasome non-ATPase regulatory subunit 3 (26S proteasome subun Length = 744; score = 40.1 bits (18), expect = 0.83; identities = 21/24 (87%); strand = Plus / Plus

```
Query: 23 ttgcttgattcacttcwwyaagag 46
          ||||||||||||||||   |||||
Sbjct: 2  ttgcttgattcacttctacaagag 25
```

>EL586535|EL586535.1 CF_W00_23d10_SP6 Copepod Whole Organism, Normalized *Calanus finmarchicus* cDNA clone CF_W00_23d10; 5' , mRNA sequence. Length = 714; score = 40.1 bits (18), expect = 0.83; identities = 21/24 (87%); strand = Plus / Plus

```
Query: 23 ttgcttgattcacttcwwyaagag 46
          ||||||||||||||||   |||||
Sbjct: 10 ttgcttgattcacttctacaagag 33
```

>EL586525|EL586525.1 CF_WO0_23c12_SP6 Copepod Whole Organism, Normalized *Calanus finmarchicus* cDNA clone CF_WO0_23c12; 5' similar to ZP_01042739.1 Monomeric isocitrate dehydrogenase [Idiomarina baltica OS145]. Score=237.65 Length = 678; score = 40.1 bits (18), expect = 0.83; identities = 21/24 (87%); strand = Plus / Plus

```
Query: 23 ttgcttgattcacttcwwyaagag 46
          ||||||||||||||||   |||||
Sbjct: 8  ttgcttgattcacttctacaagag 31
```

>EL586470|EL586470.1 CF_WO0_22g02_SP6 Copepod Whole Organism, Normalized *Calanus finmarchicus* cDNA clone CF_WO0_22g02; 5' similar to EAT46276.1 serine hydroxymethyltransferase [Aedes aegypti]. Score=301.98, Expect=1.20E-80 Length = 682; score = 40.1 bits (18), expect = 0.83; identities = 21/24 (87%); strand = Plus / Plus

```
Query: 23 ttgcttgattcacttcwwyaagag 46
          ||||||||||||||||   |||||
Sbjct: 40 ttgcttgattcacttctacaagag 63
```

>EL586461|EL586461.1 CF_WO0_22f05_SP6 Copepod Whole Organism, Normalized *Calanus finmarchicus* cDNA clone CF_WO0_22f05; 5', mRNA sequence. Length = 744; score = 40.1 bits (18), expect = 0.83; identities = 21/24 (87%); strand = Plus / Plus

```
Query: 23 ttgcttgattcacttcwwyaagag 46
          ||||||||||||||||   |||||
Sbjct: 10 ttgcttgattcacttctacaagag 33
```

>EL586451|EL586451.1 CF_WO0_22e07_SP6 Copepod Whole Organism, Normalized *Calanus finmarchicus* cDNA clone CF_WO0_22e07; 5' similar to NP_001040037.1 asparaginyl-tRNA synthetase [Bos taurus]. Score=172.17, Expect=1.80E-41, m Length = 746; score = 40.1 bits (18), expect = 0.83; identities = 21/24 (87%); strand = Plus / Plus

```
Query: 23 ttgcttgattcacttcwwyaagag 46
          ||||||||||||||||   |||||
Sbjct: 40 ttgcttgattcacttctacaagag 63
```

>EL586449|EL586449.1 CF_WO0_22e05_SP6 Copepod Whole Organism, Normalized *Calanus finmarchicus* cDNA clone CF_WO0_22e05; 5', mRNA sequence. Length = 726; score = 40.1 bits (18), expect = 0.83; identities = 21/24 (87%); strand = Plus / Plus

```
Query: 23 ttgcttgattcacttcwwyaagag 46
          ||||||||||||||||   |||||
Sbjct: 14 ttgcttgattcacttctacaagag 37
```

>EL586394|EL586394.1 CF_WO0_21h09_SP6 Copepod Whole Organism, Normalized *Calanus finmarchicus* cDNA clone CF_WO0_21h09; 5' similar to XP_789356.2 MGC81656 protein [Strongylocentrotus purpuratus]. Score=118.63, Expect=1.60E- Length = 631; score = 40.1 bits (18), expect = 0.83; identities = 21/24 (87%); strand = Plus / Plus

```
Query: 23 ttgcttgattcacttcwwyaagag 46
          ||||||||||||||||   |||||
Sbjct: 11 ttgcttgattcacttctacaagag 34
```

>EL586390|EL586390.1 CF_W00_21h05_SP6 Copepod Whole Organism, Normalized *Calanus finmarchicus* cDNA clone CF_W00_21h05; 5’ similar to XP_794125.2 ST7 protein [Strongylocentrotus purpuratus]. Score=197.21, Expect=4.60E-49, m Length = 703; score = 40.1 bits (18), expect = 0.83; identities = 21/24 (87%); strand = Plus / Plus

```
Query: 23 ttgcttgattcacttcwwyaagag 46
          ||||||||||||||||   |||||
Sbjct: 10 ttgcttgattcacttctacaagag 33
```

>EL586367|EL586367.1 CF_W00_21f06_SP6 Copepod Whole Organism, Normalized *Calanus finmarchicus* cDNA clone CF_W00_21f06; 5’ similar to P02206 Myoglobin. Score=50.83, Expect=0.000046, mRNA sequence. Length = 654; score = 40.1 bits (18), expect = 0.83; identities = 21/24 (87%); strand = Plus / Plus

```
Query: 23 ttgcttgattcacttcwwyaagag 46
          ||||||||||||||||   |||||
Sbjct: 2  ttgcttgattcacttctacaagag 25
```

>EL586342|EL586342.1 CF_W00_21d05_SP6 Copepod Whole Organism, Normalized *Calanus finmarchicus* cDNA clone CF_W00_21d05; 5’ similar to XP_974969.1 COP9 signalosome complex subunit 4 (Signalosome subunit 4) (SGN4) (JAB1-conta Length = 628; score = 40.1 bits (18), expect = 0.83; identities = 27/34 (79%); strand = Plus / Plus

```
Query: 13 caagctwawwttgcttgattcacttcwwyaagag 46
          |||||| |  |||||||||||| |||   |||||
Sbjct: 3  caagctaatattgcttgattcatttctacaagag 36
```

>EL586340|EL586340.1 CF_W00_21d03_SP6 Copepod Whole Organism, Normalized *Calanus finmarchicus* cDNA clone CF_W00_21d03; 5’ similar to NP_003067.2 SWI/SNF-related matrix-associated actin-dependent regulator of chromatin d1 i Length = 602; score = 40.1 bits (18), expect = 0.83; identities = 21/24 (87%); strand = Plus / Plus

```
Query: 23 ttgcttgattcacttcwwyaagag 46
          ||||||||||||||||   |||||
Sbjct: 11 ttgcttgattcacttctacaagag 34
```

>EL586310|EL586310.1 CF_W00_21a07_SP6 Copepod Whole Organism, Normalized *Calanus finmarchicus* cDNA clone CF_W00_21a07; 5’ similar to XP_623743.1 Rpt1 CG1341-PA [Apis mellifera]. Score=327.79, Expect=2.20E-88, mRNA sequence Length = 693; score = 40.1 bits (18), expect = 0.83; identities = 21/24 (87%); strand = Plus / Plus

```
Query: 23 ttgcttgattcacttcwwyaagag 46
          ||||||||||||||||   |||||
Sbjct: 1  ttgcttgattcacttctacaagag 24
```

>EL586308|EL586308.1 CF_WO0_21a05_SP6 Copepod Whole Organism, Normalized *Calanus finmarchicus* cDNA clone CF_WO0_21a05; 5' , mRNA sequence. Length = 678; score = 40.1 bits (18), expect = 0.83; identities = 21/24 (87%); strand = Plus / Plus

```
Query: 23 ttgcttgattcacttcwwyaagag 46
          ||||||||||||||||   |||||
Sbjct: 15 ttgcttgattcacttctacaagag 38
```

>EL586300|EL586300.1 CF_WO0_20h09_SP6 Copepod Whole Organism, Normalized *Calanus finmarchicus* cDNA clone CF_WO0_20h09; 5' , mRNA sequence. Length = 679; score = 40.1 bits (18), expect = 0.83; identities = 21/24 (87%); strand = Plus / Plus

```
Query: 23 ttgcttgattcacttcwwyaagag 46
          ||||||||||||||||   |||||
Sbjct: 12 ttgcttgattcacttctacaagag 35
```

>EL586297|EL586297.1 CF_WO0_20h06_SP6 Copepod Whole Organism, Normalized *Calanus finmarchicus* cDNA clone CF_WO0_20h06; 5' similar to XP_001106845.1 Y55F3AM.9 [Macaca mulatta]. Score=58.15, Expect=2.50E-07, mRNA sequence. Length = 619; score = 40.1 bits (18), expect = 0.83; identities = 21/24 (87%); strand = Plus / Plus

```
Query: 23 ttgcttgattcacttcwwyaagag 46
          ||||||||||||||||   |||||
Sbjct: 15 ttgcttgattcacttctacaagag 38
```

>EL586295|EL586295.1 CF_WO0_20h04_SP6 Copepod Whole Organism, Normalized *Calanus finmarchicus* cDNA clone CF_WO0_20h04; 5' similar to NP_001013583.1 hypothetical protein LOC541440 [Danio rerio]. Score=88.58, Expect=2.30E-16 Length = 709; score = 40.1 bits (18), expect = 0.83; identities = 21/24 (87%); strand = Plus / Plus

```
Query: 23 ttgcttgattcacttcwwyaagag 46
          ||||||||||||||||   |||||
Sbjct: 9  ttgcttgattcacttctacaagag 32
```

>EL586290|EL586290.1 CF_WO0_20g11_SP6 Copepod Whole Organism, Normalized *Calanus finmarchicus* cDNA clone CF_WO0_20g11; 5' , mRNA sequence. Length = 688; score = 40.1 bits (18), expect = 0.83; identities = 21/24 (87%); strand = Plus / Plus

```
Query: 23 ttgcttgattcacttcwwyaagag 46
          ||||||||||||||||   |||||
Sbjct: 9  ttgcttgattcacttctacaagag 32
```

>EL586275|EL586275.1 CF_WO0_20f08_SP6 Copepod Whole Organism, Normalized *Calanus finmarchicus* cDNA clone CF_WO0_20f08; 5' , mRNA sequence. Length = 734; score = 40.1 bits (18), expect = 0.83; identities = 21/24 (87%); strand = Plus / Plus

```
Query: 23 ttgcttgattcacttcwwyaagag 46
          ||||||||||||||||   |||||
Sbjct: 14 ttgcttgattcacttctacaagag 37
```

>EL586265|EL586265.1 CF_WO0_20e10_SP6 Copepod Whole Organism, Normalized *Calanus finmarchicus* cDNA clone CF_WO0_20e10; 5' similar to XP_624580.1 Chromatin assembly factor 1 subunit CG4236-PA [Apis mellifera]. Score=421.01, Length = 676; score = 40.1 bits (18), expect = 0.83; identities = 21/24 (87%); strand = Plus / Plus

```
Query: 23 ttgcttgattcacttcwwyaagag 46
          |||||||||||||||   |||||
Sbjct: 10 ttgcttgattcacttctacaagag 33
```

>EL586224|EL586224.1 CF_WO0_20b04_SP6 Copepod Whole Organism, Normalized *Calanus finmarchicus* cDNA clone CF_WO0_20b04; 5', mRNA sequence. Length = 642; score = 40.1 bits (18), expect = 0.83; identities = 21/24 (87%); strand = Plus / Plus

```
Query: 23 ttgcttgattcacttcwwyaagag 46
          |||||||||||||||   |||||
Sbjct: 10 ttgcttgattcacttctacaagag 33
```

>EL586203|EL586203.1 CF_WO0_19h03_SP6 Copepod Whole Organism, Normalized *Calanus finmarchicus* cDNA clone CF_WO0_19h03; 5', mRNA sequence. Length = 702; score = 40.1 bits (18), expect = 0.83; identities = 21/24 (87%); strand = Plus / Plus

```
Query: 23 ttgcttgattcacttcwwyaagag 46
          |||||||||||||||   |||||
Sbjct: 10 ttgcttgattcacttctacaagag 33
```

>EL586195|EL586195.1 CF_WO0_19g06_SP6 Copepod Whole Organism, Normalized *Calanus finmarchicus* cDNA clone CF_WO0_19g06; 5', mRNA sequence. Length = 686; score = 40.1 bits (18), expect = 0.83; identities = 21/24 (87%); strand = Plus / Plus

```
Query: 23 ttgcttgattcacttcwwyaagag 46
          |||||||||||||||   |||||
Sbjct: 14 ttgcttgattcacttctacaagag 37
```

>EL586187|EL586187.1 CF_WO0_19f10_SP6 Copepod Whole Organism, Normalized *Calanus finmarchicus* cDNA clone CF_WO0_19f10; 5' similar to NP_989988.1 branched chain keto acid dehydrogenase E1, beta polypeptide [Gallus gallus]. Length = 710; score = 40.1 bits (18), expect = 0.83; identities = 21/24 (87%); strand = Plus / Plus

```
Query: 23 ttgcttgattcacttcwwyaagag 46
          |||||||||||||||   |||||
Sbjct: 41 ttgcttgattcacttctacaagag 64
```

>EL586116|EL586116.1 CF_WO0_18h08_SP6 Copepod Whole Organism, Normalized *Calanus finmarchicus* cDNA clone CF_WO0_18h08; 5' similar to XP_975730.1 CG4672-PA isoform 2 [Tribolium castaneum]. Score=183.34, Expect=6.90E-45, mRN Length = 701; score = 40.1 bits (18), expect = 0.83; identities = 21/24 (87%); strand = Plus / Plus

```
Query: 23 ttgcttgattcacttcwwyaagag 46
```

||||||||||||||| |||||
Sbjct: 14 ttgcttgattcacttctacaagag 37

>EL586074|EL586074.1 CF_WO0_18d12_SP6 Copepod Whole Organism, Normalized *Calanus finmarchicus* cDNA clone CF_WO0_18d12; 5' , mRNA sequence. Length = 778; score = 40.1 bits (18), expect = 0.83; identities = 21/24 (87%); strand = Plus / Plus

Query: 23 ttgcttgattcacttcwwyaagag 46
||||||||||||||| |||||
Sbjct: 9 ttgcttgattcacttctacaagag 32

>EL586004|EL586004.1 CF_WO0_17f08_SP6 Copepod Whole Organism, Normalized *Calanus finmarchicus* cDNA clone CF_WO0_17f08; 5' , mRNA sequence. Length = 625; score = 40.1 bits (18), expect = 0.83; identities = 21/24 (87%); strand = Plus / Plus

Query: 23 ttgcttgattcacttcwwyaagag 46
||||||||||||||| |||||
Sbjct: 10 ttgcttgattcacttctacaagag 33

>EL586003|EL586003.1 CF_WO0_17f07_SP6 Copepod Whole Organism, Normalized *Calanus finmarchicus* cDNA clone CF_WO0_17f07; 5' , mRNA sequence. Length = 659; score = 40.1 bits (18), expect = 0.83; identities = 21/24 (87%); strand = Plus / Plus

Query: 23 ttgcttgattcacttcwwyaagag 46
||||||||||||||| |||||
Sbjct: 8 ttgcttgattcacttctacaagag 31

>EL585994|EL585994.1 CF_WO0_17e10_SP6 Copepod Whole Organism, Normalized *Calanus finmarchicus* cDNA clone CF_WO0_17e10; 5' , mRNA sequence. Length = 672; score = 40.1 bits (18), expect = 0.83; identities = 21/24 (87%); strand = Plus / Plus

Query: 23 ttgcttgattcacttcwwyaagag 46
||||||||||||||| |||||
Sbjct: 10 ttgcttgattcacttctacaagag 33

>EL585990|EL585990.1 CF_WO0_17e06_SP6 Copepod Whole Organism, Normalized *Calanus finmarchicus* cDNA clone CF_WO0_17e06; 5' similar to NP_001006260.1 eukaryotic translation initiation factor 2, subunit 3 gamma, 52kDa [Gallus Length = 662; score = 40.1 bits (18), expect = 0.83; identities = 21/24 (87%); strand = Plus / Plus

Query: 23 ttgcttgattcacttcwwyaagag 46
||||||||||||||| |||||
Sbjct: 12 ttgcttgattcacttctacaagag 35

>EL585958|EL585958.1 CF_WO0_17b09_SP6 Copepod Whole Organism, Normalized *Calanus finmarchicus* cDNA clone CF_WO0_17b09; 5' similar to CAE60897.1 Hypothetical protein CBG04612 [Caenorhabditis briggsae]. Score=167.93, Expect= Length = 675; score = 40.1 bits (18), expect = 0.83; identities = 21/24 (87%); strand = Plus / Plus

Query: 23 ttgcttgattcacttcwwyaagag 46
         |||||||||||||||| |||||
Sbjct: 10 ttgcttgattcacttctacaagag 33

>EL585896|EL585896.1 CF_WO0_16e06_SP6 Copepod Whole Organism, Normalized *Calanus finmarchicus* cDNA clone CF_WO0_16e06; 5' similar to BAA32394.1 FTZ-F1 [Oryzias latipes]. Score=74.33, Expect=3.80E-12, mRNA sequence. Length = 648; score = 40.1 bits (18), expect = 0.83; identities = 21/24 (87%); strand = Plus / Plus

Query: 23 ttgcttgattcacttcwwyaagag 46
         |||||||||||||||| |||||
Sbjct: 2 ttgcttgattcacttctacaagag 25

>EL585866|EL585866.1 CF_WO0_16b09_SP6 Copepod Whole Organism, Normalized *Calanus finmarchicus* cDNA clone CF_WO0_16b09; 5' similar to XP_972481.1 CG11154-PA, isoform A [Tribolium castaneum]. Score=237.65, Expect=2.80E-61, m Length = 677; score = 40.1 bits (18), expect = 0.83; identities = 21/24 (87%); strand = Plus / Plus

Query: 23 ttgcttgattcacttcwwyaagag 46
         |||||||||||||||| |||||
Sbjct: 5 ttgcttgattcacttctacaagag 28

>EL585838|EL585838.1 CF_WO0_15h04_SP6 Copepod Whole Organism, Normalized *Calanus finmarchicus* cDNA clone CF_WO0_15h04; 5' similar to XP_417601.2 hypothetical protein [Gallus gallus]. Score=69.32, Expect=1.20E-10, mRNA sequ Length = 645; score = 40.1 bits (18), expect = 0.83; identities = 21/24 (87%); strand = Plus / Plus

Query: 23 ttgcttgattcacttcwwyaagag 46
         |||||||||||||||| |||||
Sbjct: 5 ttgcttgattcacttctacaagag 28

>EL585837|EL585837.1 CF_WO0_15h03_SP6 Copepod Whole Organism, Normalized *Calanus finmarchicus* cDNA clone CF_WO0_15h03; 5' similar to XP_417601.2 hypothetical protein [Gallus gallus]. Score=69.32, Expect=1.50E-10, mRNA sequ Length = 715; score = 40.1 bits (18), expect = 0.83; identities = 21/24 (87%); strand = Plus / Plus

Query: 23 ttgcttgattcacttcwwyaagag 46
         |||||||||||||||| |||||
Sbjct: 3 ttgcttgattcacttctacaagag 26

>EL585770|EL585770.1 CF_WO0_15b03_SP6 Copepod Whole Organism, Normalized *Calanus finmarchicus* cDNA clone CF_WO0_15b03; 5' similar to EAT35135.1 3-5 exonuclease [Aedes aegypti]. Score=114, Expect=3.80E-24, mRNA sequence. Length = 612; score = 40.1 bits (18), expect = 0.83; identities = 21/24 (87%); strand = Plus / Plus

Query: 23 ttgcttgattcacttcwwyaagag 46
         |||||||||||||||| |||||
Sbjct: 12 ttgcttgattcacttctacaagag 35

>EL585727|EL585727.1 CF_WO0_14f07_SP6 Copepod Whole Organism, Normalized *Calanus finmarchicus* cDNA clone CF_WO0_14f07; 5' similar to AAX26008.2 SJCHGC02654 protein [Schistosoma japonicum]. Score=50.83, Expect=0.00005-mRNA Length = 681; score = 40.1 bits (18), expect = 0.83; identities = 21/24 (87%); strand = Plus / Plus

```
Query: 23 ttgcttgattcacttcwwyaagag 46
          ||||||||||||||||   |||||
Sbjct: 5  ttgcttgattcacttctacaagag 28
```

>EL585687|EL585687.1 CF_WO0_14c03_SP6 Copepod Whole Organism, Normalized *Calanus finmarchicus* cDNA clone CF_WO0_14c03; 5' similar to NP_001026743.1 hypothetical protein LOC429380 [Gallus gallus]. Score=137.89, Expect=3.20E. Length = 688; score = 40.1 bits (18), expect = 0.83; identities = 21/24 (87%); strand = Plus / Plus

```
Query: 23 ttgcttgattcacttcwwyaagag 46
          ||||||||||||||||   |||||
Sbjct: 9  ttgcttgattcacttctacaagag 32
```

>EL585635|EL585635.1 CF_WO0_13f10_SP6 Copepod Whole Organism, Normalized *Calanus finmarchicus* cDNA clone CF_WO0_13f10; 5' , mRNA sequence. Length = 687; score = 40.1 bits (18), expect = 0.83; identities = 21/24 (87%); strand = Plus / Plus

```
Query: 23 ttgcttgattcacttcwwyaagag 46
          ||||||||||||||||   |||||
Sbjct: 11 ttgcttgattcacttctacaagag 34
```

>EL585595|EL585595.1 CF_WO0_13b12_SP6 Copepod Whole Organism, Normalized *Calanus finmarchicus* cDNA clone CF_WO0_13b12; 5' similar to AAY66861.1 unknown [Ixodes scapularis]. Score=109.77, Expect=1.00E-22, mRNA sequence. Length = 715; score = 40.1 bits (18), expect = 0.83; identities = 21/24 (87%); strand = Plus / Plus

```
Query: 23 ttgcttgattcacttcwwyaagag 46
          ||||||||||||||||   |||||
Sbjct: 8  ttgcttgattcacttctacaagag 31
```

>EL585555|EL585555.1 CF_WO0_12g04_SP6 Copepod Whole Organism, Normalized *Calanus finmarchicus* cDNA clone CF_WO0_12g04; 5' similar to XP_623743.1 Rpt1 CG1341-PA [Apis mellifera]. Score=330.1, Expect=4.10E-89, mRNA sequence. Length = 666; score = 40.1 bits (18), expect = 0.83; identities = 21/24 (87%); strand = Plus / Plus

```
Query: 23 ttgcttgattcacttcwwyaagag 46
          ||||||||||||||||   |||||
Sbjct: 10 ttgcttgattcacttcttcaagag 33
```

>EL585554|EL585554.1 CF_WO0_12g03_SP6 Copepod Whole Organism, Normalized *Calanus finmarchicus* cDNA clone CF_WO0_12g03; 5' similar to XP_395332.2 chromosome segregation 1-like [Apis mellifera]. Score=229.18, Expect=1.10E-58 Length = 718; score = 40.1 bits (18), expect = 0.83; identities = 21/24 (87%); strand = Plus / Plus

Query: 23 ttgcttgattcacttcwwyaagag 46
         |||||||||||||||| |||||
Sbjct: 10 ttgcttgattcacttctataagag 33

>EL585535|EL585535.1 CF_W00_12e06_SP6 Copepod Whole Organism, Normalized *Calanus finmarchicus* cDNA clone CF_W00_12e06; 5' similar to XP_969406.1 CG5805-PA [Tribolium castaneum]. Score=90.12, Expect=4.70E-17, mRNA sequence. Length = 559; score = 40.1 bits (18), expect = 0.83; identities = 21/24 (87%); strand = Plus / Plus

Query: 23 ttgcttgattcacttcwwyaagag 46
         |||||||||||||||| |||||
Sbjct: 11 ttgcttgattcacttctacaagag 34

>EL585502|EL585502.1 CF_W00_12b02_SP6 Copepod Whole Organism, Normalized *Calanus finmarchicus* cDNA clone CF_W00_12b02; 5' similar to NP_956571.1 hypothetical protein LOC393247 [Danio rerio]. Score=112.46, Expect=1.40E-23, Length = 684; score = 40.1 bits (18), expect = 0.83; identities = 21/24 (87%); strand = Plus / Plus

Query: 23 ttgcttgattcacttcwwyaagag 46
         |||||||||||||||| |||||
Sbjct: 9 ttgcttgattcacttctacaagag 32

>EL585496|EL585496.1 CF_W00_12a08_SP6 Copepod Whole Organism, Normalized *Calanus finmarchicus* cDNA clone CF_W00_12a08; 5' similar to XP_312436.3 ENSANGP00000023709 [Anopheles gambiae str. PEST]. Score=53.53, Expect=0.00000 Length = 442; score = 40.1 bits (18), expect = 0.83; identities = 21/24 (87%); strand = Plus / Plus

Query: 23 ttgcttgattcacttcwwyaagag 46
         |||||||||||||||| |||||
Sbjct: 8 ttgcttgattcacttctacaagag 31

>EL585486|EL585486.1 CF_W00_11h09_SP6 Copepod Whole Organism, Normalized *Calanus finmarchicus* cDNA clone CF_W00_11h09; 5' similar to YP_591988.1 protein of unknown function DUF885 [Acidobacteria bacterium Ellin345]. Score= Length = 662; score = 40.1 bits (18), expect = 0.83; identities = 21/24 (87%); strand = Plus / Plus

Query: 23 ttgcttgattcacttcwwyaagag 46
         |||||||||||||||| |||||
Sbjct: 10 ttgcttgattcacttctacaagag 33

>EL585481|EL585481.1 CF_W00_11h04_SP6 Copepod Whole Organism, Normalized *Calanus finmarchicus* cDNA clone CF_W00_11h04; 5' similar to NP_001071868.1 zinc finger protein [Ciona intestinalis]. Score=59.31, Expect=9.50E-08, mR Length = 573; score = 40.1 bits (18), expect = 0.83; identities = 21/24 (87%); strand = Plus / Plus

Query: 23 ttgcttgattcacttcwwyaagag 46
         |||||||||||||||| |||||
Sbjct: 8 ttgcttgattcacttctacaagag 31

>EL585463|EL585463.1 CF_WO0_11f09_SP6 Copepod Whole Organism, Normalized *Calanus finmarchicus* cDNA clone CF_WO0_11f09; 5' similar to XP_395842.2 BTB/POZ domain-containing protein 9 [Apis mellifera]. Score=201.83, Expect=1. Length = 607; score = 40.1 bits (18), expect = 0.83; identities = 21/24 (87%); strand = Plus / Plus

```
Query: 23 ttgcttgattcacttcwwyaagag 46
          ||||||||||||||||   |||||
Sbjct: 10 ttgcttgattcacttctacaagag 33
```

>EL585426|EL585426.1 CF_WO0_11c08_SP6 Copepod Whole Organism, Normalized *Calanus finmarchicus* cDNA clone CF_WO0_11c08; 5' , mRNA sequence. Length = 643; score = 40.1 bits (18), expect = 0.83; identities = 21/24 (87%); strand = Plus / Plus

```
Query: 23 ttgcttgattcacttcwwyaagag 46
          ||||||||||||||||   |||||
Sbjct: 14 ttgcttgattcacttctacaagag 37
```

>EL585423|EL585423.1 CF_WO0_11c05_SP6 Copepod Whole Organism, Normalized *Calanus finmarchicus* cDNA clone CF_WO0_11c05; 5' , mRNA sequence. Length = 586; score = 40.1 bits (18), expect = 0.83; identities = 21/24 (87%); strand = Plus / Plus

```
Query: 23 ttgcttgattcacttcwwyaagag 46
          ||||||||||||||||   |||||
Sbjct: 8  ttgcttgattcacttctacaagag 31
```

>EL585413|EL585413.1 CF_WO0_11b07_SP6 Copepod Whole Organism, Normalized *Calanus finmarchicus* cDNA clone CF_WO0_11b07; 5' similar to XP_969791.1 CG9253-PA [Tribolium castaneum]. Score=86.66, Expect=8.10E-16, mRNA sequence. Length = 676; score = 40.1 bits (18), expect = 0.83; identities = 21/24 (87%); strand = Plus / Plus

```
Query: 23 ttgcttgattcacttcwwyaagag 46
          ||||||||||||||||   |||||
Sbjct: 10 ttgcttgattcacttctacaagag 33
```

>EL585412|EL585412.1 CF_WO0_11b05_SP6 Copepod Whole Organism, Normalized *Calanus finmarchicus* cDNA clone CF_WO0_11b05; 5' similar to XP_974487.1 CG3194-PA [Tribolium castaneum]. Score=147.13, Expect=5.00E-34, mRNA sequence Length = 673; score = 40.1 bits (18), expect = 0.83; identities = 21/24 (87%); strand = Plus / Plus

```
Query: 23 ttgcttgattcacttcwwyaagag 46
          ||||||||||||||||   |||||
Sbjct: 5  ttgcttgattcacttctacaagag 28
```

>EH667157|EH667157.1 CF_WO0_10f06_SP6 Copepod Whole Organism, Normalized *Calanus finmarchicus* cDNA clone CF_WO0_10f06; 5' similar to ref|XP_316880.3| ENSANGP00000017843 - Anopheles gambiae str. PEST. Score = 261 bits (667) Length = 723; score = 40.1 bits (18), expect = 0.83; identities = 21/24 (87%); strand = Plus / Plus

```
Query: 23 ttgcttgattcacttcwwyaagag 46
```

          ||||||||||||||| |||||
Sbjct: 13 ttgcttgattcacttctacaagag 36

>EH667144|EH667144.1 CF_WO0_10e05_SP6 Copepod Whole Organism, Normalized *Calanus finmarchicus* cDNA clone CF_WO0_10e05; 5' similar to emb|CAI12034.1| MCM3 minichromosome maintenance deficient 3 (S. cerevisiae) [Danio. Score Length = 714; score = 40.1 bits (18), expect = 0.83; identities = 21/24 (87%); strand = Plus / Plus

Query: 23 ttgcttgattcacttcwwyaagag 46
          ||||||||||||||| |||||
Sbjct: 9 ttgcttgattcacttctacaagag 32

>EH667140|EH667140.1 CF_WO0_10d12_SP6 Copepod Whole Organism, Normalized *Calanus finmarchicus* cDNA clone CF_WO0_10d12; 5' similar to ref|XP_975206.1| DIVDICTED: similar to CG9862-PA - Tribolium castaneum. Score = 298 bits Length = 686; score = 40.1 bits (18), expect = 0.83; identities = 21/24 (87%); strand = Plus / Plus

Query: 23 ttgcttgattcacttcwwyaagag 46
          ||||||||||||||| |||||
Sbjct: 4 ttgcttgattcacttctacaagag 27

>EH667138|EH667138.1 CF_WO0_10d10_SP6 Copepod Whole Organism, Normalized *Calanus finmarchicus* cDNA clone CF_WO0_10d10; 5' , mRNA sequence. Length = 684; score = 40.1 bits (18), expect = 0.83; identities = 21/24 (87%); strand = Plus / Plus

Query: 23 ttgcttgattcacttcwwyaagag 46
          ||||||||||||||| |||||
Sbjct: 7 ttgcttgattcacttctacaagag 30

>EH667105|EH667105.1 CF_WO0_10a12_SP6 Copepod Whole Organism, Normalized *Calanus finmarchicus* cDNA clone CF_WO0_10a12; 5' similar to ref|XP_792811.2| DIVDICTED: similar to E1-like protein [Strongylocentrotus. Score = 140 b Length = 712; score = 40.1 bits (18), expect = 0.83; identities = 21/24 (87%); strand = Plus / Plus

Query: 23 ttgcttgattcacttcwwyaagag 46
          ||||||||||||||| |||||
Sbjct: 2 ttgcttgattcacttctacaagag 25

>EH667104|EH667104.1 CF_WO0_10a11_SP6 Copepod Whole Organism, Normalized *Calanus finmarchicus* cDNA clone CF_WO0_10a11; 5' similar to ref|NP_001040346.1| septin - Bombyx mori. Score = 332 bits (852), expect = 8e-90, mRNA se Length = 704; score = 40.1 bits (18), expect = 0.83; identities = 21/24 (87%); strand = Plus / Plus

Query: 23 ttgcttgattcacttcwwyaagag 46
          ||||||||||||||| |||||
Sbjct: 11 ttgcttgattcacttctacaagag 34

>EH667095|EH667095.1 CF_WO0_10a02_SP6 Copepod Whole Organism, Normalized *Calanus finmarchicus* cDNA clone CF_WO0_10a02; 5' similar to ref|XP_625041.1| DIVDICTED: similar to CG11265-PA,

isoform A - Apis mellifera. Score = 66 Length = 731; score = 40.1 bits (18), expect = 0.83; identities = 21/24 (87%); strand = Plus / Plus

```
Query: 23 ttgcttgattcacttcwwyaagag 46
          ||||||||||||||||   |||||
Sbjct: 2  ttgcttgattcacttctacaagag 25
```

>EH667089|EH667089.1 CF_W00_09h08_SP6 Copepod Whole Organism, Normalized *Calanus finmarchicus* cDNA clone CF_W00_09h08; 5' similar to ref|NP_001072534.1| hypothetical protein LOC779989 - Xenopus tropicalis. Score = 201 bits Length = 593; score = 40.1 bits (18), expect = 0.83; identities = 21/24 (87%); strand = Plus / Plus

```
Query: 23 ttgcttgattcacttcwwyaagag 46
          ||||||||||||||||   |||||
Sbjct: 10 ttgcttgattcacttctacaagag 33
```

>EH667085|EH667085.1 CF_W00_09h04_SP6 Copepod Whole Organism, Normalized *Calanus finmarchicus* cDNA clone CF_W00_09h04; 5' similar to ref|NP_188702.2| carbohydrate transporter/ sugar porter - Arabidopsis thaliana. Score = 8 Length = 591; score = 40.1 bits (18), expect = 0.83; identities = 21/24 (87%); strand = Plus / Plus

```
Query: 23 ttgcttgattcacttcwwyaagag 46
          ||||||||||||||||   |||||
Sbjct: 14 ttgcttgattcacttctacaagag 37
```

>EH667084|EH667084.1 CF_W00_09h03_SP6 Copepod Whole Organism, Normalized *Calanus finmarchicus* cDNA clone CF_W00_09h03; 5', mRNA sequence. Length = 688; score = 40.1 bits (18), expect = 0.83; identities = 21/24 (87%); strand = Plus / Plus

```
Query: 23 ttgcttgattcacttcwwyaagag 46
          ||||||||||||||||   |||||
Sbjct: 4  ttgcttgattcacttctacaagag 27
```

>EH667068|EH667068.1 CF_W00_09f11_SP6 Copepod Whole Organism, Normalized *Calanus finmarchicus* cDNA clone CF_W00_09f11; 5' similar to emb|CAG01979.1| unnamed protein product - Tetraodon nigroviridis. Score = 125 bits (314), Length = 638; score = 40.1 bits (18), expect = 0.83; identities = 21/24 (87%); strand = Plus / Plus

```
Query: 23 ttgcttgattcacttcwwyaagag 46
          ||||||||||||||||   |||||
Sbjct: 10 ttgcttgattcacttctacaagag 33
```

>EH667055|EH667055.1 CF_W00_09e06_SP6 Copepod Whole Organism, Normalized *Calanus finmarchicus* cDNA clone CF_W00_09e06; 5' similar to gb|ABF18346.1| 26S proteasome regulatory chain 4 - Aedes aegypti. Score = 368 bits (945), Length = 744; score = 40.1 bits (18), expect = 0.83; identities = 21/24 (87%); strand = Plus / Plus

```
Query: 23 ttgcttgattcacttcwwyaagag 46
          ||||||||||||||||   |||||
```

Sbjct: 11 ttgcttgattcacttctacaagag 34

>EH667023|EH667023.1 CF_WO0_09b04_SP6 Copepod Whole Organism, Normalized *Calanus finmarchicus* cDNA clone CF_WO0_09b04; 5', mRNA sequence. Length = 665; score = 40.1 bits (18), expect = 0.83; identities = 21/24 (87%); strand = Plus / Plus

```
Query: 23 ttgcttgattcacttcwwyaagag 46
          |||||||||||||||   |||||
Sbjct: 8  ttgcttgattcacttctacaagag 31
```

>EH667012|EH667012.1 CF_WO0_09a04_SP6 Copepod Whole Organism, Normalized *Calanus finmarchicus* cDNA clone CF_WO0_09a04; 5' similar to ref|NP_612069.1| CG13907-PA - Drosophila melanogaster. Score = 104 bits (259), expect = 4 Length = 699; score = 40.1 bits (18), expect = 0.83; identities = 21/24 (87%); strand = Plus / Plus

```
Query: 23 ttgcttgattcacttcwwyaagag 46
          |||||||||||||||   |||||
Sbjct: 12 ttgcttgattcacttctacaagag 35
```

>EH667003|EH667003.1 CF_WO0_08h06_SP6 Copepod Whole Organism, Normalized *Calanus finmarchicus* cDNA clone CF_WO0_08h06; 5' similar to ref|XP_416122.2| DIVDICTED: similar to Chromosome 12 open reading frame 26 [Gallus. Score Length = 653; score = 40.1 bits (18), expect = 0.83; identities = 21/24 (87%); strand = Plus / Plus

```
Query: 23 ttgcttgattcacttcwwyaagag 46
          |||||||||||||||   |||||
Sbjct: 10 ttgcttgattcacttctacaagag 33
```

>EH666985|EH666985.1 CF_WO0_08f12_SP6 Copepod Whole Organism, Normalized *Calanus finmarchicus* cDNA clone CF_WO0_08f12; 5' similar to gb|AAH84892.1| LOC495407 protein - Xenopus laevis. Score = 59.3 bits (142), expect = 1e-0 Length = 648; score = 40.1 bits (18), expect = 0.83; identities = 21/24 (87%); strand = Plus / Plus

```
Query: 23 ttgcttgattcacttcwwyaagag 46
          |||||||||||||||   |||||
Sbjct: 2  ttgcttgattcacttctacaagag 25
```

>EH666941|EH666941.1 CF_WO0_08c04_SP6 Copepod Whole Organism, Normalized *Calanus finmarchicus* cDNA clone CF_WO0_08c04; 5', mRNA sequence. Length = 721; score = 40.1 bits (18), expect = 0.83; identities = 21/24 (87%); strand = Plus / Plus

```
Query: 23 ttgcttgattcacttcwwyaagag 46
          |||||||||||||||   |||||
Sbjct: 10 ttgcttgattcacttctacaagag 33
```

>EH666929|EH666929.1 CF_WO0_08b04_SP6 Copepod Whole Organism, Normalized *Calanus finmarchicus* cDNA clone CF_WO0_08b04; 5' similar to gb|EAT41759.1| n-acetyllactosaminide beta-1,3-n-acetylglucosaminyltransferase. Score = 65 Length = 635; score = 40.1 bits (18), expect = 0.83; identities = 21/24 (87%); strand = Plus / Plus

```
Query: 23 ttgcttgattcacttcwwyaagag 46
          |||||||||||||||   |||||
Sbjct: 5  ttgcttgattcacttctacaagag 28
```

>EH666878|EH666878.1 CF_W00_07e12_SP6 Copepod Whole Organism, Normalized *Calanus finmarchicus* cDNA clone CF_W00_07e12; 5’ similar to ref|NP_956001.1| elongation factor RNA polymerase II - Danio rerio. Score = 57.8 bits (13 Length = 646; score = 40.1 bits (18), expect = 0.83; identities = 21/24 (87%); strand = Plus / Plus

```
Query: 23 ttgcttgattcacttcwwyaagag 46
          |||||||||||||||   |||||
Sbjct: 20 ttgcttgattcacttctacaagag 43
```

>EH666869|EH666869.1 CF_W00_07e03_SP6 Copepod Whole Organism, Normalized *Calanus finmarchicus* cDNA clone CF_W00_07e03; 5’ similar to gb|AAO15914.1|AF510054_1 vasa-like - Schistocerca gregaria. Score = 218 bits (554), Expec Length = 711; score = 40.1 bits (18), expect = 0.83; identities = 21/24 (87%); strand = Plus / Plus

```
Query: 23 ttgcttgattcacttcwwyaagag 46
          |||||||||||||||   |||||
Sbjct: 10 ttgcttgattcacttctacaagag 33
```

>EH666861|EH666861.1 CF_W00_07d07_SP6 Copepod Whole Organism, Normalized *Calanus finmarchicus* cDNA clone CF_W00_07d07; 5’ similar to ref|NP_989706.1| solute carrier family 2, (facilitated glucose transporter) member 8. Sco Length = 659; score = 40.1 bits (18), expect = 0.83; identities = 21/24 (87%); strand = Plus / Plus

```
Query: 23 ttgcttgattcacttcwwyaagag 46
          |||||||||||||||   |||||
Sbjct: 10 ttgcttgattcacttctacaagag 33
```

>EH666851|EH666851.1 CF_W00_07c09_SP6 Copepod Whole Organism, Normalized *Calanus finmarchicus* cDNA clone CF_W00_07c09; 5’ similar to ref|XP_967224.1| DIVDICTED: similar to Aspartate aminotransferase, mitochondrial. Score = Length = 687; score = 40.1 bits (18), expect = 0.83; identities = 21/24 (87%); strand = Plus / Plus

```
Query: 23 ttgcttgattcacttcwwyaagag 46
          |||||||||||||||   |||||
Sbjct: 13 ttgcttgattcacttctacaagag 36
```

>EH666831|EH666831.1 CF_W00_07b01_SP6 Copepod Whole Organism, Normalized *Calanus finmarchicus* cDNA clone CF_W00_07b01; 5’ , mRNA sequence. Length = 705; score = 40.1 bits (18), expect = 0.83; identities = 21/24 (87%); strand = Plus / Plus

```
Query: 23 ttgcttgattcacttcwwyaagag 46
          |||||||||||||||   |||||
Sbjct: 14 ttgcttgattcacttctacaagag 37
```

>EH666798|EH666798.1 CF_WO0_06g03_SP6 Copepod Whole Organism, Normalized *Calanus finmarchicus* cDNA clone CF_WO0_06g03; 5' , mRNA sequence. Length = 607; score = 40.1 bits (18), expect = 0.83; identities = 21/24 (87%); strand = Plus / Plus

```
Query: 23 ttgcttgattcacttcwwyaagag 46
          ||||||||||||||||   |||||
Sbjct: 2  ttgcttgattcacttctacaagag 25
```

>EH666796|EH666796.1 CF_WO0_06g01_SP6 Copepod Whole Organism, Normalized *Calanus finmarchicus* cDNA clone CF_WO0_06g01; 5' , mRNA sequence. Length = 518; score = 40.1 bits (18), expect = 0.83; identities = 21/24 (87%); strand = Plus / Plus

```
Query: 23 ttgcttgattcacttcwwyaagag 46
          ||||||||||||||||   |||||
Sbjct: 6  ttgcttgattcacttctacaagag 29
```

>EH666782|EH666782.1 CF_WO0_06e10_SP6 Copepod Whole Organism, Normalized *Calanus finmarchicus* cDNA clone CF_WO0_06e10; 5' , mRNA sequence. Length = 608; score = 40.1 bits (18), expect = 0.83; identities = 21/24 (87%); strand = Plus / Plus

```
Query: 23 ttgcttgattcacttcwwyaagag 46
          ||||||||||||||||   |||||
Sbjct: 5  ttgcttgattcacttctacaagag 28
```

>EH666742|EH666742.1 CF_WO0_06b05_SP6 Copepod Whole Organism, Normalized *Calanus finmarchicus* cDNA clone CF_WO0_06b05; 5' similar to ref|NP_001032633.1| colonic system B0+ amino acid transporter - Rattus norvegicus. Score Length = 681; score = 40.1 bits (18), expect = 0.83; identities = 21/24 (87%); strand = Plus / Plus

```
Query: 23 ttgcttgattcacttcwwyaagag 46
          ||||||||||||||||   |||||
Sbjct: 10 ttgcttgattcacttctacaagag 33
```

>EH666721|EH666721.1 CF_WO0_05h08_SP6 Copepod Whole Organism, Normalized *Calanus finmarchicus* cDNA clone CF_WO0_05h08; 5' similar to ref|XP_397387.3| DIVDICTED: similar to CG4845-PA - Apis mellifera. Score = 219 bits (559) Length = 593; score = 40.1 bits (18), expect = 0.83; identities = 21/24 (87%); strand = Plus / Plus

```
Query: 23 ttgcttgattcacttcwwyaagag 46
          ||||||||||||||||   |||||
Sbjct: 5  ttgcttgattcacttctacaagag 28
```

>EH666718|EH666718.1 CF_WO0_05h05_SP6 Copepod Whole Organism, Normalized *Calanus finmarchicus* cDNA clone CF_WO0_05h05; 5' similar to ref|XP_968648.1| DIVDICTED: similar to CG33138-PA - Tribolium castaneum. Score = 183 bits Length = 638; score = 40.1 bits (18), expect = 0.83; identities = 21/24 (87%); strand = Plus / Plus

```
Query: 23 ttgcttgattcacttcwwyaagag 46
          ||||||||||||||||   |||||
```

Sbjct: 10 ttgcttgattcacttctacaagag 33

>EH666703|EH666703.1 CF_W00_05g01_SP6 Copepod Whole Organism, Normalized *Calanus finmarchicus* cDNA clone CF_W00_05g01; 5' , mRNA sequence. Length = 548; score = 40.1 bits (18), expect = 0.83; identities = 21/24 (87%); strand = Plus / Plus

```
Query: 23 ttgcttgattcacttcwwyaagag 46
          |||||||||||||||   |||||
Sbjct: 15 ttgcttgattcacttctacaagag 38
```

>EH666696|EH666696.1 CF_W00_05f06_SP6 Copepod Whole Organism, Normalized *Calanus finmarchicus* cDNA clone CF_W00_05f06; 5' similar to gb|EAT35690.1| aldehyde dehydrogenase - Aedes aegypti. Score = 253 bits (647), expect = 5 Length = 721; score = 40.1 bits (18), expect = 0.83; identities = 21/24 (87%); strand = Plus / Plus

```
Query: 23 ttgcttgattcacttcwwyaagag 46
          |||||||||||||||   |||||
Sbjct: 10 ttgcttgattcacttctacaagag 33
```

>EH666623|EH666623.1 CF_W00_04h02_SP6 Copepod Whole Organism, Normalized *Calanus finmarchicus* cDNA clone CF_W00_04h02; 5' similar to gb|EAT34027.1| beta-arrestin 1, putative - Aedes aegypti. Score = 276 bits (706), expect Length = 641; score = 40.1 bits (18), expect = 0.83; identities = 21/24 (87%); strand = Plus / Plus

```
Query: 23 ttgcttgattcacttcwwyaagag 46
          |||||||||||||||   |||||
Sbjct: 1  ttgcttgattcacttctacaagag 24
```

>EH666608|EH666608.1 CF_W00_04f10_SP6 Copepod Whole Organism, Normalized *Calanus finmarchicus* cDNA clone CF_W00_04f10; 5' similar to gb|AAH42288.1| MGC53536 protein - Xenopus laevis. Score = 73.9 bits (180), expect = 5e-12 Length = 637; score = 40.1 bits (18), expect = 0.83; identities = 21/24 (87%); strand = Plus / Plus

```
Query: 23 ttgcttgattcacttcwwyaagag 46
          |||||||||||||||   |||||
Sbjct: 15 ttgcttgattcacttctacaagag 38
```

>EH666602|EH666602.1 CF_W00_04f03_SP6 Copepod Whole Organism, Normalized *Calanus finmarchicus* cDNA clone CF_W00_04f03; 5' similar to ref|XP_624580.1| DIVDICTED: similar to Chromatin assembly factor 1 subunit CG4236-PA. Sco Length = 673; score = 40.1 bits (18), expect = 0.83; identities = 21/24 (87%); strand = Plus / Plus

```
Query: 23 ttgcttgattcacttcwwyaagag 46
          |||||||||||||||   |||||
Sbjct: 11 ttgcttgattcacttctacaagag 34
```

>EH666581|EH666581.1 CF_W00_04d04_SP6 Copepod Whole Organism, Normalized *Calanus finmarchicus* cDNA clone CF_W00_04d04; 5' , mRNA sequence. Length = 586; score = 40.1 bits (18), expect = 0.83; identities = 21/24 (87%); strand = Plus / Plus

Query: 23 ttgcttgattcacttcwwyaagag 46
         ||||||||||||||| |||||
Sbjct: 5 ttgcttgattcacttctacaagag 28

>EH666551|EH666551.1 CF_WOO_04a08_SP6 Copepod Whole Organism, Normalized *Calanus finmarchicus* cDNA clone CF_WOO_04a08; 5' similar to ref|NP_031559.2| branched chain ketoacid dehydrogenase E1, alpha polypeptide [Mus. Score Length = 724; score = 40.1 bits (18), expect = 0.83; identities = 21/24 (87%); strand = Plus / Plus

Query: 23 ttgcttgattcacttcwwyaagag 46
         ||||||||||||||| |||||
Sbjct: 11 ttgcttgattcacttctacaagag 34

>EH666539|EH666539.1 CF_WOO_03h06_SP6 Copepod Whole Organism, Normalized *Calanus finmarchicus* cDNA clone CF_WOO_03h06; 5' similar to ref|XP_319583.3| ENSANGP00000016715 - Anopheles gambiae str. PEST. Score = 77.4 bits (189 Length = 581; score = 40.1 bits (18), expect = 0.83; identities = 21/24 (87%); strand = Plus / Plus

Query: 23 ttgcttgattcacttcwwyaagag 46
         ||||||||||||||| |||||
Sbjct: 10 ttgcttgattcacttctacaagag 33

>EH666474|EH666474.1 CF_WOO_03b12_SP6 Copepod Whole Organism, Normalized *Calanus finmarchicus* cDNA clone CF_WOO_03b12; 5' , mRNA sequence. Length = 666; score = 40.1 bits (18), expect = 0.83; identities = 21/24 (87%); strand = Plus / Plus

Query: 23 ttgcttgattcacttcwwyaagag 46
         ||||||||||||||| |||||
Sbjct: 7 ttgcttgattcacttctacaagag 30

>EH666446|EH666446.1 CF_WOO_02h05_SP6 Copepod Whole Organism, Normalized *Calanus finmarchicus* cDNA clone CF_WOO_02h05; 5' similar to ref|NP_001038620.1| hypothetical protein LOC568176 - Danio rerio. Score = 150 bits (380), Length = 699; score = 40.1 bits (18), expect = 0.83; identities = 21/24 (87%); strand = Plus / Plus

Query: 23 ttgcttgattcacttcwwyaagag 46
         ||||||||||||||| |||||
Sbjct: 10 ttgcttgattcacttctacaagag 33

>EH666417|EH666417.1 CF_WOO_02e11_SP6 Copepod Whole Organism, Normalized *Calanus finmarchicus* cDNA clone CF_WOO_02e11; 5' , mRNA sequence. Length = 737; score = 40.1 bits (18), expect = 0.83; identities = 21/24 (87%); strand = Plus / Plus

Query: 23 ttgcttgattcacttcwwyaagag 46
         ||||||||||||||| |||||
Sbjct: 10 ttgcttgattcacttctacaagag 33

>EH666414|EH666414.1 CF_WO0_02e08_SP6 Copepod Whole Organism, Normalized *Calanus finmarchicus* cDNA clone CF_WO0_02e08; 5’ similar to ref|XP_414824.2| DIVDICTED: similar to serine hydroxymethyltransferase 1 (soluble). Score Length = 657; score = 40.1 bits (18), expect = 0.83; identities = 21/24 (87%); strand = Plus / Plus

```
Query: 23 ttgcttgattcacttcwwyaagag 46
          ||||||||||||||||   |||||
Sbjct: 15 ttgcttgattcacttctacaagag 38
```

>EH666389|EH666389.1 CF_WO0_02c07_SP6 Copepod Whole Organism, Normalized *Calanus finmarchicus* cDNA clone CF_WO0_02c07; 5’ similar to gb|AAW26893.1| SJCHGC04686 protein - Schistosoma japonicum. Score = 70.9 bits (172), Expe Length = 657; score = 40.1 bits (18), expect = 0.83; identities = 21/24 (87%); strand = Plus / Plus

```
Query: 23 ttgcttgattcacttcwwyaagag 46
          ||||||||||||||||   |||||
Sbjct: 14 ttgcttgattcacttctacaagag 37
```

>EH666387|EH666387.1 CF_WO0_02c05_SP6 Copepod Whole Organism, Normalized *Calanus finmarchicus* cDNA clone CF_WO0_02c05; 5’ similar to gb|EAT48161.1| phospholipid-transporting atpase 1 (aminophospholipid flippase 1). Score = Length = 663; score = 40.1 bits (18), expect = 0.83; identities = 21/24 (87%); strand = Plus / Plus

```
Query: 23 ttgcttgattcacttcwwyaagag 46
          ||||||||||||||||   |||||
Sbjct: 9  ttgcttgattcacttctacaagag 32
```

>EH666343|EH666343.1 CF_WO0_01g08_SP6 Copepod Whole Organism, Normalized *Calanus finmarchicus* cDNA clone CF_WO0_01g08; 5’, mRNA sequence. Length = 676; score = 40.1 bits (18), expect = 0.83; identities = 21/24 (87%); strand = Plus / Plus

```
Query: 23 ttgcttgattcacttcwwyaagag 46
          ||||||||||||||||   |||||
Sbjct: 14 ttgcttgattcacttctacaagag 37
```

>EH666318|EH666318.1 CF_WO0_01e06_SP6 Copepod Whole Organism, Normalized *Calanus finmarchicus* cDNA clone CF_WO0_01e06; 5’ similar to ref|XP_315955.3| ENSANGP00000013550 - Anopheles gambiae str. PEST. Score = 106 bits (265) Length = 706; score = 40.1 bits (18), expect = 0.83; identities = 21/24 (87%); strand = Plus / Plus

```
Query: 23 ttgcttgattcacttcwwyaagag 46
          ||||||||||||||||   |||||
Sbjct: 11 ttgcttgattcacttctacaagag 34
```

>EH666313|EH666313.1 CF_WO0_01e01_SP6 Copepod Whole Organism, Normalized *Calanus finmarchicus* cDNA clone CF_WO0_01e01; 5’ similar to ref|XP_782559.1| DIVDICTED: similar to MGC84654 protein [Strongylocentrotus. Score = 236 Length = 618; score = 40.1 bits (18), expect = 0.83; identities = 21/24 (87%); strand = Plus / Plus

Query: 23 ttgcttgattcacttcwwyaagag 46
         ||||||||||||||| |||||
Sbjct: 7 ttgcttgattcacttctacaagag 30

>EH666305|EH666305.1 CF_W00_01d03_SP6 Copepod Whole Organism, Normalized *Calanus finmarchicus* cDNA clone CF_W00_01d03; 5' , mRNA sequence. Length = 614; score = 40.1 bits (18), expect = 0.83; identities = 21/24 (87%); strand = Plus / Plus

Query: 23 ttgcttgattcacttcwwyaagag 46
         ||||||||||||||| |||||
Sbjct: 8 ttgcttgattcacttctacaagag 31

>EH666304|EH666304.1 CF_W00_01d02_SP6 Copepod Whole Organism, Normalized *Calanus finmarchicus* cDNA clone CF_W00_01d02; 5' similar to gb|AAL28486.1| GM08053p - Drosophila melanogaster. Score = 55.8 bits (133), expect = 1e-0 Length = 601; score = 40.1 bits (18), expect = 0.83; identities = 21/24 (87%); strand = Plus / Plus

Query: 23 ttgcttgattcacttcwwyaagag 46
         ||||||||||||||| |||||
Sbjct: 8 ttgcttgattcacttctacaagag 31

>EH666293|EH666293.1 CF_W00_01c02_SP6 Copepod Whole Organism, Normalized *Calanus finmarchicus* cDNA clone CF_W00_01c02; 5' similar to gb|AAI27276.1| Unknown (protein for MGC:145380) - Xenopus tropicalis. Score = 228 bits (5 Length = 584; score = 40.1 bits (18), expect = 0.83; identities = 21/24 (87%); strand = Plus / Plus

Query: 23 ttgcttgattcacttcwwyaagag 46
         ||||||||||||||| |||||
Sbjct: 8 ttgcttgattcacttctacaagag 31

>EH666272|EH666272.1 CF_W00_01a04_SP6 Copepod Whole Organism, Normalized *Calanus finmarchicus* cDNA clone CF_W00_01a04; 5' , mRNA sequence. Length = 647; score = 40.1 bits (18), expect = 0.83; identities = 21/24 (87%); strand = Plus / Plus

Query: 23 ttgcttgattcacttcwwyaagag 46
         ||||||||||||||| |||||
Sbjct: 13 ttgcttgattcacttctacaagag 36

>DY656243|DY656243.1 Cm_mx1_78d05_SP6 Green Shore Crab Multiple Tissue, Normalized Carcinus maenas cDNA clone Cm_mx1_78d05 5', mRNA sequence. Length = 602; score = 40.1 bits (18), expect = 0.83; identities = 21/24 (87%); strand = Plus / Plus

Query: 23 ttgcttgattcacttcwwyaagag 46
         ||||||||||||||| |||||
Sbjct: 12 ttgcttgattcacttctacaagag 35

>DV943741|DV943741.1 Cm_mx1_05d07_SP6 Green Shore Crab Multiple Tissue, Normalized Carcinus maenas cDNA clone Cm_mx1_05d07 5' similar to Similar to emb|CAG00121.1| unnamed protein

product - Tetraodon nigroviridis. Score = Length = 678; score = 40.1 bits (18), expect = 0.83; identities = 21/24 (87%); strand = Plus / Plus

```
Query: 23 ttgcttgattcacttcwwyaagag 46
          ||||||||||||||||   |||||
Sbjct: 12 ttgcttgattcacttctacaagag 35
```

>DV642724|DV642724.1 Cm_mx0_97e08_SP6 Green Shore Crab Multiple Tissue, Normalized Carcinus maenas cDNA clone Cm_mx0_97e08 5' similar to emb|CAG00121.1| unnamed protein product - Tetraodon nigroviridis. Score = 329 bits (Length = 693; score = 40.1 bits (18), expect = 0.83; identities = 21/24 (87%); strand = Plus / Plus

```
Query: 23 ttgcttgattcacttcwwyaagag 46
          ||||||||||||||||   |||||
Sbjct: 12 ttgcttgattcacttctacaagag 35
```

>DN796273|DN796273.1 Cm_mx0_60h12_SP6 Green Shore Crab Multiple Tissue, Normalized Carcinus maenas cDNA clone Cm_mx0_60h12 5' similar to gb|EAL62546.1| hypothetical protein DDB0188508 - Dictyostelium discoideum. Score = 4 Length = 618; score = 40.1 bits (18), expect = 0.83; identities = 21/24 (87%); strand = Plus / Plus

```
Query: 23 ttgcttgattcacttcwwyaagag 46
          ||||||||||||||||   |||||
Sbjct: 3  ttgcttgattcacttctacaagag 26
```

>DN796153|DN796153.1 Cm_mx0_59d10_SP6 Green Shore Crab Multiple Tissue, Normalized Carcinus maenas cDNA clone Cm_mx0_59d10 5' similar to gb|AAD13639.1| blood-stage membrane protein Ag-1 - Plasmodium yoelii. Score = 42.7 b Length = 729; score = 40.1 bits (18), expect = 0.83; identities = 21/24 (87%); strand = Plus / Plus

```
Query: 23 ttgcttgattcacttcwwyaagag 46
          ||||||||||||||||   |||||
Sbjct: 3  ttgcttgattcacttctacaagag 26
```

>DN739048|DN739048.1 Cm_mx0_52f12_SP6 Green Shore Crab Multiple Tissue, Normalized Carcinus maenas cDNA clone Cm_mx0_52f12 5' similar to gb|EAL62546.1| hypothetical protein DDB0188508 - Dictyostelium discoideum. Score = 4 Length = 617; score = 40.1 bits (18), expect = 0.83; identities = 21/24 (87%); strand = Plus / Plus

```
Query: 23 ttgcttgattcacttcwwyaagag 46
          ||||||||||||||||   |||||
Sbjct: 3  ttgcttgattcacttctacaagag 26
```

>DN738903|DN738903.1 Cm_mx0_50h01_SP6 Green Shore Crab Multiple Tissue, Normalized Carcinus maenas cDNA clone Cm_mx0_50h01 5' similar to gb|AAD13639.1| blood-stage membrane protein Ag-1 - Plasmodium yoelii. Score = 42.7 b Length = 689; score = 40.1 bits (18), expect = 0.83; identities = 21/24 (87%); strand = Plus / Plus

```
Query: 23 ttgcttgattcacttcwwyaagag 46
```

```
         |||||||||||||||   |||||
Sbjct: 3  ttgcttgattcacttctacaagag 26
```

>LA314161|LA314161.1 TSA: Fish metagenome RNA, contig: contig17279_C2388668, isolate: YEZ. Length = 556; score = 40.1 bits (18), expect = 0.83; identities = 21/24 (87%); strand = Plus / Plus

```
Query: 23 ttgcttgattcacttcwwyaagag 46
          |||||||||||||||   |||||
Sbjct: 4  ttgcttgattcacttctacaagag 27
```

>LA313185|LA313185.1 TSA: Fish metagenome RNA, contig: contig16303_C2386388, isolate: YEZ. Length = 538; score = 40.1 bits (18), expect = 0.83; identities = 21/24 (87%); strand = Plus / Plus

```
Query: 23 ttgcttgattcacttcwwyaagag 46
          |||||||||||||||   |||||
Sbjct: 4  ttgcttgattcacttctacaagag 27
```

>JZ533428|JZ533428.1 SSH_RHO3_B05_ab1 Cal SSH_SKE_RHO 2d *Calanus helgolandicus* cDNA clone SSH_RHO3_B05_ab1 similar to 60s ribosomal protein l11, mRNA sequence. Length = 367; score = 37.9 bits (17), expect = 3.8; identities = 20/23 (86%); strand = Plus / Minus

```
Query: 24  tgcttgattcacttcwwyaagag 46
           ||||||||||||||   |||||
Sbjct: 359 tgcttgattcacttctacaagag 337
```

>JZ533427|JZ533427.1 SSH_RHO3_H11_ab1 Cal SSH_SKE_RHO 2d *Calanus helgolandicus* cDNA clone SSH_RHO3_H11_ab1 similar to cofilin actin-depolymerizing factor homolog, mRNA sequence. Length = 253; score = 37.9 bits (17), expect = 3.8; identities = 20/23 (86%); strand = Plus / Minus

```
Query: 24  tgcttgattcacttcwwyaagag 46
           ||||||||||||||   |||||
Sbjct: 245 tgcttgattcacttctacaagag 223
```

>JZ533391|JZ533391.1 SSH_RHO2_A2_T7_ab1 Cal SSH_SKE_RHO 2d *Calanus helgolandicus* cDNA clone SSH_RHO2_A2_T7_ab1 similar to 60s ribosomal protein l11, mRNA sequence. Length = 366; score = 37.9 bits (17), expect = 3.8; identities = 20/23 (86%); strand = Plus / Minus

```
Query: 24  tgcttgattcacttcwwyaagag 46
           ||||||||||||||   |||||
Sbjct: 358 tgcttgattcacttctacaagag 336
```

>JZ533362|JZ533362.1 SSH_RHO1_C2_T7_ab1 Cal SSH_SKE_RHO 2d *Calanus helgolandicus* cDNA clone SSH_RHO1_C2_T7_ab1 similar to 60s ribosomal protein l11, mRNA sequence. Length = 366; score = 37.9 bits (17), expect = 3.8; identities = 20/23 (86%); strand = Plus / Minus

```
Query: 24  tgcttgattcacttcwwyaagag 46
```

              |||||||||||||| |||||
Sbjct: 358 tgcttgattcacttctacaagag 336

>GR411182|GR411182.1 CF_W01_58e12_SP6 Copepod Whole Organism, Normalized *Calanus finmarchicus* cDNA clone CF_W01_58e12; 5' similar to gb|EEB14434.1| Fasciculation and elongation protein zeta, putative [Pediculus. Score = 17 Length = 852; score = 37.9 bits (17), expect = 3.8; identities = 20/23 (86%); strand = Plus / Plus

Query: 24 tgcttgattcacttcwwyaagag 46
           |||||||||||||| |||||
Sbjct: 19 tgcttgattcacttctacaagag 41

>GR411167|GR411167.1 CF_W01_58d07_SP6 Copepod Whole Organism, Normalized *Calanus finmarchicus* cDNA clone CF_W01_58d07; 5', mRNA sequence. Length = 874; score = 37.9 bits (17), expect = 3.8; identities = 20/23 (86%); strand = Plus / Plus

Query: 24 tgcttgattcacttcwwyaagag 46
           |||||||||||||| |||||
Sbjct: 11 tgcttgattcacttctacaagag 33

>GR411126|GR411126.1 CF_W01_57h02_SP6 Copepod Whole Organism, Normalized *Calanus finmarchicus* cDNA clone CF_W01_57h02; 5', mRNA sequence. Length = 596; score = 37.9 bits (17), expect = 3.8; identities = 20/23 (86%); strand = Plus / Plus

Query: 24 tgcttgattcacttcwwyaagag 46
           |||||||||||||| |||||
Sbjct: 9 tgcttgattcacttctacaagag 31

>GR411057|GR411057.1 CF_W01_56g12_SP6 Copepod Whole Organism, Normalized *Calanus finmarchicus* cDNA clone CF_W01_56g12; 5' similar to ref|YP_001340250.1| major facilitator transporter - Marinomonas sp. MWYL1. Score = 62.8 b Length = 588; score = 37.9 bits (17), expect = 3.8; identities = 20/23 (86%); strand = Plus / Plus

Query: 24 tgcttgattcacttcwwyaagag 46
           |||||||||||||| |||||
Sbjct: 8 tgcttgattcacttctacaagag 30

>GR411006|GR411006.1 CF_W01_55h11_SP6 Copepod Whole Organism, Normalized *Calanus finmarchicus* cDNA clone CF_W01_55h11; 5', mRNA sequence. Length = 623; score = 37.9 bits (17), expect = 3.8; identities = 20/23 (86%); strand = Plus / Plus

Query: 24 tgcttgattcacttcwwyaagag 46
           |||||||||||||| |||||
Sbjct: 9 tgcttgattcacttctacaagag 31

>GR410989|GR410989.1 CF_W01_55f12_SP6 Copepod Whole Organism, Normalized *Calanus finmarchicus* cDNA clone CF_W01_55f12; 5' similar to ref|XP_001849525.1| ribonucleaseP/MRP protein subunit POP1 - Culex quinquefasciatus. Scor Length = 649; score = 37.9 bits (17), expect = 3.8; identities = 20/23 (86%); strand = Plus / Plus

Query: 24 tgcttgattcacttcwwyaagag 46
          ||||||||||||||| |||||
Sbjct: 12 tgcttgattcacttctacaagag 34

>GR410988|GR410988.1 CF_W01_55f10_SP6 Copepod Whole Organism, Normalized *Calanus finmarchicus* cDNA clone CF_W01_55f10; 5' , mRNA sequence. Length = 616; score = 37.9 bits (17), expect = 3.8; identities = 20/23 (86%); strand = Plus / Plus

Query: 24 tgcttgattcacttcwwyaagag 46
          ||||||||||||||| |||||
Sbjct: 13 tgcttgattcacttctacaagag 35

>GR410828|GR410828.1 CF_W01_53d08_SP6 Copepod Whole Organism, Normalized *Calanus finmarchicus* cDNA clone CF_W01_53d08; 5' similar to ref|XP_002098627.1| GE23835 - Drosophila yakuba. Score = 104 bits (259), expect = 6e-21, Length = 604; score = 37.9 bits (17), expect = 3.8; identities = 20/23 (86%); strand = Plus / Plus

Query: 24 tgcttgattcacttcwwyaagag 46
          ||||||||||||||| |||||
Sbjct: 9 tgcttgattcacttctacaagag 31

>GR410804|GR410804.1 CF_W01_53b03_SP6 Copepod Whole Organism, Normalized *Calanus finmarchicus* cDNA clone CF_W01_53b03; 5' similar to ref|XP_971095.1| DIVDICTED: similar to WD repeat-containing protein 59 [Tribolium. Score Length = 793; score = 37.9 bits (17), expect = 3.8; identities = 20/23 (86%); strand = Plus / Plus

Query: 24 tgcttgattcacttcwwyaagag 46
          ||||||||||||||| |||||
Sbjct: 8 tgcttgattcacttctacaagag 30

>FP988813|FP988813.1 Pleurobrachia pileus 5-PRIME EST from clone SQ0AAA44YC10 (SQ0AAA44YC10RM1). Length = 869; score = 37.9 bits (17), expect = 3.8; identities = 20/23 (86%); strand = Plus / Plus

Query: 24 tgcttgattcacttcwwyaagag 46
          ||||||||||||||| |||||
Sbjct: 1 tgcttgattcacttctacaagag 23

>FK868376|FK868376.1 CF_W01_20G08_SP6 Copepod Whole Organism, Normalized *Calanus finmarchicus* cDNA clone CF_W01_20G08; 5' similar to ref|XP_001603850.1| DIVDICTED: similar to DEAD box ATP-dependent RNA helicase [Nasonia. S Length = 628; score = 37.9 bits (17), expect = 3.8; identities = 20/23 (86%); strand = Plus / Plus

Query: 24 tgcttgattcacttcwwyaagag 46
          ||||||||||||||| |||||
Sbjct: 14 tgcttgattcacttctacaagag 36

>FK868309|FK868309.1 CF_WO1_20A11_SP6 Copepod Whole Organism, Normalized *Calanus finmarchicus* cDNA clone CF_WO1_20A11; 5' similar to ref|XP_001605947.1| DIVDICTED: similar to conserved hypothetical protein [Nasonia. Score Length = 643; score = 37.9 bits (17), expect = 3.8; identities = 20/23 (86%); strand = Plus / Plus

```
Query: 24 tgcttgattcacttcwwyaagag 46
          ||||||||||||||   |||||
Sbjct: 13 tgcttgattcacttctacaagag 35
```

>FK868252|FK868252.1 CF_WO1_19C09_SP6 Copepod Whole Organism, Normalized *Calanus finmarchicus* cDNA clone CF_WO1_19C09; 5' similar to ref|NP_998343.1| regulator of chromosome condensation 1 - Danio rerio. Score = 106 bits (Length = 609; score = 37.9 bits (17), expect = 3.8; identities = 20/23 (86%); strand = Plus / Plus

```
Query: 24 tgcttgattcacttcwwyaagag 46
          ||||||||||||||   |||||
Sbjct: 14 tgcttgattcacttctacaagag 36
```

>FK868245|FK868245.1 CF_WO1_19C01_SP6 Copepod Whole Organism, Normalized *Calanus finmarchicus* cDNA clone CF_WO1_19C01; 5' similar to ref|XP_001375431.1| DIVDICTED: similar to methylmalonate-semialdehyde dehydrogenase. Scor Length = 661; score = 37.9 bits (17), expect = 3.8; identities = 20/23 (86%); strand = Plus / Plus

```
Query: 24 tgcttgattcacttcwwyaagag 46
          ||||||||||||||   |||||
Sbjct: 1  tgcttgattcacttctacaagag 23
```

>FK868241|FK868241.1 CF_WO1_19B06_SP6 Copepod Whole Organism, Normalized *Calanus finmarchicus* cDNA clone CF_WO1_19B06; 5' similar to ref|NP_999659.1| KRP110 - Strongylocentrotus purpuratus. Score = 137 bits (344), expect = Length = 542; score = 37.9 bits (17), expect = 3.8; identities = 20/23 (86%); strand = Plus / Plus

```
Query: 24 tgcttgattcacttcwwyaagag 46
          ||||||||||||||   |||||
Sbjct: 7  tgcttgattcacttctacaagag 29
```

>FK868177|FK868177.1 CF_WO1_18D06_SP6 Copepod Whole Organism, Normalized *Calanus finmarchicus* cDNA clone CF_WO1_18D06; 5' similar to ref|XP_973304.1| DIVDICTED: similar to pyruvate dehydrogenase kinase [Tribolium. Score = Length = 526; score = 37.9 bits (17), expect = 3.8; identities = 20/23 (86%); strand = Plus / Plus

```
Query: 24 tgcttgattcacttcwwyaagag 46
          ||||||||||||||   |||||
Sbjct: 11 tgcttgattcacttctacaagag 33
```

>FK868055|FK868055.1 CF_WO1_16C10_SP6 Copepod Whole Organism, Normalized *Calanus finmarchicus* cDNA clone CF_WO1_16C10; 5' similar to emb|CAP72045.1| tubulin alpha-2 - Fasciola hepatica. Score = 339 bits (869), expect = 1e- Length = 666; score = 37.9 bits (17), expect = 3.8; identities = 20/23 (86%); strand = Plus / Plus

Query: 24 tgcttgattcacttcwwyaagag 46
         ||||||||||||||| |||||
Sbjct: 2 tgcttgattcacttctacaagag 24

>FK868002|FK868002.1 CF_W01_15E11_SP6 Copepod Whole Organism, Normalized *Calanus finmarchicus* cDNA clone CF_W01_15E11; 5' similar to gb|EDW55803.1| GM17377 - Drosophila sechellia. Score = 108 bits (271), expect = 2e-22, mR Length = 584; score = 37.9 bits (17), expect = 3.8; identities = 20/23 (86%); strand = Plus / Plus

Query: 24 tgcttgattcacttcwwyaagag 46
         ||||||||||||||| |||||
Sbjct: 15 tgcttgattcacttctacaagag 37

>FK867966|FK867966.1 CF_W01_15B03_SP6 Copepod Whole Organism, Normalized *Calanus finmarchicus* cDNA clone CF_W01_15B03; 5' , mRNA sequence. Length = 637; score = 37.9 bits (17), expect = 3.8; identities = 20/23 (86%); strand = Plus / Plus

Query: 24 tgcttgattcacttcwwyaagag 46
         ||||||||||||||| |||||
Sbjct: 4 tgcttgattcacttctacaagag 26

>FK867941|FK867941.1 CF_W01_14G06_SP6 Copepod Whole Organism, Normalized *Calanus finmarchicus* cDNA clone CF_W01_14G06; 5' similar to ref|XP_001607195.1| DIVDICTED: similar to beta-adaptin Drosophila 1 [Nasonia. Score = 323 Length = 628; score = 37.9 bits (17), expect = 3.8; identities = 20/23 (86%); strand = Plus / Plus

Query: 24 tgcttgattcacttcwwyaagag 46
         ||||||||||||||| |||||
Sbjct: 2 tgcttgattcacttctacaagag 24

>FK867831|FK867831.1 CF_W01_13C01_SP6 Copepod Whole Organism, Normalized *Calanus finmarchicus* cDNA clone CF_W01_13C01; 5' , mRNA sequence. Length = 653; score = 37.9 bits (17), expect = 3.8; identities = 20/23 (86%); strand = Plus / Plus

Query: 24 tgcttgattcacttcwwyaagag 46
         ||||||||||||||| |||||
Sbjct: 11 tgcttgattcacttctacaagag 33

>FK867790|FK867790.1 CF_W01_12G04_SP6 Copepod Whole Organism, Normalized *Calanus finmarchicus* cDNA clone CF_W01_12G04; 5' similar to ref|XP_969407.1| DIVDICTED: similar to Abnormal X segregation, putative [Tribolium. Score Length = 716; score = 37.9 bits (17), expect = 3.8; identities = 20/23 (86%); strand = Plus / Plus

Query: 24 tgcttgattcacttcwwyaagag 46
         ||||||||||||||| |||||
Sbjct: 11 tgcttgattcacttctacaagag 33

>FK867580|FK867580.1 CF_W01_10C08_SP6 Copepod Whole Organism, Normalized *Calanus finmarchicus* cDNA clone CF_W01_10C08; 5’ , mRNA sequence. Length = 607; score = 37.9 bits (17), expect = 3.8; identities = 20/23 (86%); strand = Plus / Plus

```
Query: 24 tgcttgattcacttcwwyaagag 46
          ||||||||||||||   |||||
Sbjct: 12 tgcttgattcacttctacaagag 34
```

>FK867507|FK867507.1 CF_W01_09E02_SP6 Copepod Whole Organism, Normalized *Calanus finmarchicus* cDNA clone CF_W01_09E02; 5’ similar to ref|XP_001605758.1| DIVDICTED: similar to GM12270p - Nasonia vitripennis. Score = 265 bit Length = 626; score = 37.9 bits (17), expect = 3.8; identities = 20/23 (86%); strand = Plus / Plus

```
Query: 24 tgcttgattcacttcwwyaagag 46
          ||||||||||||||   |||||
Sbjct: 12 tgcttgattcacttctacaagag 34
```

>FK671150|FK671150.1 CF_W01_06H06_SP6 Copepod Whole Organism, Normalized *Calanus finmarchicus* cDNA clone CF_W01_06H06; 5’ similar to dbj|BAB85212.1| ATP lipid-binding protein like protein - Marsupenaeus japonicus. Score = Length = 466; score = 37.9 bits (17), expect = 3.8; identities = 20/23 (86%); strand = Plus / Plus

```
Query: 24 tgcttgattcacttcwwyaagag 46
          ||||||||||||||   |||||
Sbjct: 2  tgcttgattcacttctacaagag 24
```

>FK671083|FK671083.1 CF_W01_06B08_SP6 Copepod Whole Organism, Normalized *Calanus finmarchicus* cDNA clone CF_W01_06B08; 5’ similar to gb|EDV47396.1| GG19604 - Drosophila erecta. Score = 272 bits (695), expect = 1e-71, mRNA Length = 624; score = 37.9 bits (17), expect = 3.8; identities = 20/23 (86%); strand = Plus / Plus

```
Query: 24 tgcttgattcacttcwwyaagag 46
          ||||||||||||||   |||||
Sbjct: 10 tgcttgattcacttctacaagag 32
```

>FK671066|FK671066.1 CF_W01_06A01_SP6 Copepod Whole Organism, Normalized *Calanus finmarchicus* cDNA clone CF_W01_06A01; 5’ similar to gb|EDV48573.1| GG23047 - Drosophila erecta. Score = 149 bits (377), expect = 1e-34, mRNA Length = 706; score = 37.9 bits (17), expect = 3.8; identities = 20/23 (86%); strand = Plus / Plus

```
Query: 24 tgcttgattcacttcwwyaagag 46
          ||||||||||||||   |||||
Sbjct: 2  tgcttgattcacttctacaagag 24
```

>FK671025|FK671025.1 CF_W01_05E08_SP6 Copepod Whole Organism, Normalized *Calanus finmarchicus* cDNA clone CF_W01_05E08; 5’ , mRNA sequence. Length = 634; score = 37.9 bits (17), expect = 3.8; identities = 20/23 (86%); strand = Plus / Plus

```
Query: 24 tgcttgattcacttcwwyaagag 46
```

```
          |||||||||||||||   |||||
Sbjct: 9  tgcttgattcacttctacaagag 31
```

>FK670924|FK670924.1 CF_W01_04D05_SP6 Copepod Whole Organism, Normalized *Calanus finmarchicus* cDNA clone CF_W01_04D05; 5’ similar to ref|XP_001650629.1| serine protease - Aedes aegypti. Score = 98.6 bits (244), expect = 3e Length = 646; score = 37.9 bits (17), expect = 3.8; identities = 20/23 (86%); strand = Plus / Plus

```
Query: 24 tgcttgattcacttcwwyaagag 46
          |||||||||||||||   |||||
Sbjct: 11 tgcttgattcacttctacaagag 33
```

>FK670868|FK670868.1 CF_W01_03G04_SP6 Copepod Whole Organism, Normalized *Calanus finmarchicus* cDNA clone CF_W01_03G04; 5’, mRNA sequence. Length = 641; score = 37.9 bits (17), expect = 3.8; identities = 20/23 (86%); strand = Plus / Plus

```
Query: 24 tgcttgattcacttcwwyaagag 46
          |||||||||||||||   |||||
Sbjct: 2  tgcttgattcacttctacaagag 24
```

>FK670826|FK670826.1 CF_W01_03C01_SP6 Copepod Whole Organism, Normalized *Calanus finmarchicus* cDNA clone CF_W01_03C01; 5’ similar to ref|NP_001090564.1| hypothetical protein LOC100036802 - Xenopus laevis. Score = 79.0 bits Length = 594; score = 37.9 bits (17), expect = 3.8; identities = 20/23 (86%); strand = Plus / Plus

```
Query: 24 tgcttgattcacttcwwyaagag 46
          |||||||||||||||   |||||
Sbjct: 11 tgcttgattcacttctacaagag 33
```

>FK670795|FK670795.1 CF_W01_02G10_SP6 Copepod Whole Organism, Normalized *Calanus finmarchicus* cDNA clone CF_W01_02G10; 5’, mRNA sequence. Length = 632; score = 37.9 bits (17), expect = 3.8; identities = 20/23 (86%); strand = Plus / Plus

```
Query: 24 tgcttgattcacttcwwyaagag 46
          |||||||||||||||   |||||
Sbjct: 15 tgcttgattcacttctacaagag 37
```

>FK670711|FK670711.1 CF_W01_01H08_SP6 Copepod Whole Organism, Normalized *Calanus finmarchicus* cDNA clone CF_W01_01H08; 5’ similar to dbj|BAF83282.1| unnamed protein product - Homo sapiens. Score = 326 bits (836), expect = Length = 612; score = 37.9 bits (17), expect = 3.8; identities = 20/23 (86%); strand = Plus / Plus

```
Query: 24 tgcttgattcacttcwwyaagag 46
          |||||||||||||||   |||||
Sbjct: 2  tgcttgattcacttctacaagag 24
```

>FK670707|FK670707.1 CF_W01_01H04_SP6 Copepod Whole Organism, Normalized *Calanus finmarchicus* cDNA clone CF_W01_01H04; 5’ similar to ref|XP_794044.2| DIVDICTED: similar to Endoplasmic

reticulum-golgi intermediate. Score = Length = 560; score = 37.9 bits (17), expect = 3.8; identities = 20/23 (86%); strand = Plus / Plus

```
Query: 24 tgcttgattcacttcwwyaagag 46
          ||||||||||||||   |||||
Sbjct: 12 tgcttgattcacttctacaagag 34
```

>FK670702|FK670702.1 CF_W01_01G11_SP6 Copepod Whole Organism, Normalized *Calanus finmarchicus* cDNA clone CF_W01_01G11; 5' similar to ref|NP_112319.2| aldehyde dehydrogenase family 6, subfamily A1 - Rattus norvegicus. Score Length = 650; score = 37.9 bits (17), expect = 3.8; identities = 20/23 (86%); strand = Plus / Plus

```
Query: 24 tgcttgattcacttcwwyaagag 46
          ||||||||||||||   |||||
Sbjct: 9  tgcttgattcacttctacaagag 31
```

>FK670573|FK670573.1 CF_W01_00B09_SP6 Copepod Whole Organism, Normalized *Calanus finmarchicus* cDNA clone CF_W01_00B09; 5' similar to ref|XP_973856.1| DIVDICTED: similar to CG12948 CG12948-PA - Tribolium castaneum. Score = Length = 656; score = 37.9 bits (17), expect = 3.8; identities = 20/23 (86%); strand = Plus / Plus

```
Query: 24 tgcttgattcacttcwwyaagag 46
          ||||||||||||||   |||||
Sbjct: 1  tgcttgattcacttctacaagag 23
```

>FK670571|FK670571.1 CF_W01_00B07_SP6 Copepod Whole Organism, Normalized *Calanus finmarchicus* cDNA clone CF_W01_00B07; 5' similar to ref|XP_001363196.1| DIVDICTED: similar to chromosome 10 open reading frame 59. Score = 96 Length = 690; score = 37.9 bits (17), expect = 3.8; identities = 20/23 (86%); strand = Plus / Plus

```
Query: 24 tgcttgattcacttcwwyaagag 46
          ||||||||||||||   |||||
Sbjct: 8  tgcttgattcacttctacaagag 30
```

>FK670518|FK670518.1 CF_W00_99E09_SP6 Copepod Whole Organism, Normalized *Calanus finmarchicus* cDNA clone CF_W00_99E09; 5' , mRNA sequence. Length = 677; score = 37.9 bits (17), expect = 3.8; identities = 20/23 (86%); strand = Plus / Plus

```
Query: 24 tgcttgattcacttcwwyaagag 46
          ||||||||||||||   |||||
Sbjct: 10 tgcttgattcacttctacaagag 32
```

>FK670508|FK670508.1 CF_W00_99D03_SP6 Copepod Whole Organism, Normalized *Calanus finmarchicus* cDNA clone CF_W00_99D03; 5' , mRNA sequence. Length = 678; score = 37.9 bits (17), expect = 3.8; identities = 20/23 (86%); strand = Plus / Plus

```
Query: 24 tgcttgattcacttcwwyaagag 46
          ||||||||||||||   |||||
Sbjct: 2  tgcttgattcacttctacaagag 24
```

>FK670446|FK670446.1 CF_WO0_98E02_SP6 Copepod Whole Organism, Normalized *Calanus finmarchicus* cDNA clone CF_WO0_98E02; 5' similar to ref|XP_395842.2| DIVDICTED: similar to BTB/POZ domain-containing protein 9 [Apis. Score = Length = 704; score = 37.9 bits (17), expect = 3.8; identities = 20/23 (86%); strand = Plus / Plus

```
Query: 24 tgcttgattcacttcwwyaagag 46
          ||||||||||||||   |||||
Sbjct: 14 tgcttgattcacttctacaagag 36
```

>FK670417|FK670417.1 CF_WO0_98B09_SP6 Copepod Whole Organism, Normalized *Calanus finmarchicus* cDNA clone CF_WO0_98B09; 5' similar to ref|XP_968492.1| DIVDICTED: similar to GA20276-PA - Tribolium castaneum. Score = 204 bits Length = 694; score = 37.9 bits (17), expect = 3.8; identities = 20/23 (86%); strand = Plus / Plus

```
Query: 24 tgcttgattcacttcwwyaagag 46
          ||||||||||||||   |||||
Sbjct: 8  tgcttgattcacttctacaagag 30
```

>FK670372|FK670372.1 CF_WO0_97F08_SP6 Copepod Whole Organism, Normalized *Calanus finmarchicus* cDNA clone CF_WO0_97F08; 5' similar to ref|NP_001087613.1| MGC81119 protein - Xenopus laevis. Score = 97.4 bits (241), expect = Length = 736; score = 37.9 bits (17), expect = 3.8; identities = 20/23 (86%); strand = Plus / Plus

```
Query: 24 tgcttgattcacttcwwyaagag 46
          ||||||||||||||   |||||
Sbjct: 8  tgcttgattcacttctacaagag 30
```

>FK041619|FK041619.1 CF_WO0_96D05_SP6 Copepod Whole Organism, Normalized *Calanus finmarchicus* cDNA clone CF_WO0_96D05; 5' similar to ref|XP_001603746.1| DIVDICTED: similar to nucleolar KKE/D repeat protein; DmNOP56. Score Length = 714; score = 37.9 bits (17), expect = 3.8; identities = 20/23 (86%); strand = Plus / Plus

```
Query: 24 tgcttgattcacttcwwyaagag 46
          ||||||||||||||   |||||
Sbjct: 2  tgcttgattcacttctacaagag 24
```

>FK041576|FK041576.1 CF_WO0_95H08_SP6 Copepod Whole Organism, Normalized *Calanus finmarchicus* cDNA clone CF_WO0_95H08; 5' similar to ref|XP_623958.2| DIVDICTED: similar to Cullin-5 (CUL-5) (Vasopressin-activated. Score = 3 Length = 680; score = 37.9 bits (17), expect = 3.8; identities = 20/23 (86%); strand = Plus / Plus

```
Query: 24 tgcttgattcacttcwwyaagag 46
          ||||||||||||||   |||||
Sbjct: 15 tgcttgattcacttctacaagag 37
```

>FK041571|FK041571.1 CF_WO0_95H03_SP6 Copepod Whole Organism, Normalized *Calanus finmarchicus* cDNA clone CF_WO0_95H03; 5', mRNA sequence. Length = 679; score = 37.9 bits (17), expect = 3.8; identities = 20/23 (86%); strand = Plus / Plus

```
Query: 24 tgcttgattcacttcwwyaagag 46
          ||||||||||||||   |||||
Sbjct: 2  tgcttgattcacttctacaagag 24
```

>FK041541|FK041541.1 CF_WOO_95E05_SP6 Copepod Whole Organism, Normalized *Calanus finmarchicus* cDNA clone CF_WOO_95E05; 5' similar to ref|XP_966394.1| DIVDICTED: similar to Phosphogluconate mutase CG5165-PA, partial. Score Length = 692; score = 37.9 bits (17), expect = 3.8; identities = 20/23 (86%); strand = Plus / Plus

```
Query: 24 tgcttgattcacttcwwyaagag 46
          ||||||||||||||   |||||
Sbjct: 2  tgcttgattcacttctacaagag 24
```

>FK041368|FK041368.1 CF_WOO_93E11_SP6 Copepod Whole Organism, Normalized *Calanus finmarchicus* cDNA clone CF_WOO_93E11; 5' similar to ref|NP_001091661.1| hypothetical protein LOC100000539 - Danio rerio. Score = 95.9 bits (2 Length = 699; score = 37.9 bits (17), expect = 3.8; identities = 20/23 (86%); strand = Plus / Plus

```
Query: 24 tgcttgattcacttcwwyaagag 46
          ||||||||||||||   |||||
Sbjct: 14 tgcttgattcacttctacaagag 36
```

>FK041306|FK041306.1 CF_WOO_92H03_SP6 Copepod Whole Organism, Normalized *Calanus finmarchicus* cDNA clone CF_WOO_92H03; 5' similar to gb|EDV28963.1| hypothetical protein TRIADDRAFT_52362 - Trichoplax adhaerens. Score = 82.8 Length = 618; score = 37.9 bits (17), expect = 3.8; identities = 20/23 (86%); strand = Plus / Plus

```
Query: 24 tgcttgattcacttcwwyaagag 46
          ||||||||||||||   |||||
Sbjct: 4  tgcttgattcacttctacaagag 26
```

>FK041288|FK041288.1 CF_WOO_92F09_SP6 Copepod Whole Organism, Normalized *Calanus finmarchicus* cDNA clone CF_WOO_92F09; 5' similar to gb|ABY84139.1| tubulin, gamma complex associated protein 4 (predicted) [Callithrix. Score Length = 600; score = 37.9 bits (17), expect = 3.8; identities = 20/23 (86%); strand = Plus / Plus

```
Query: 24 tgcttgattcacttcwwyaagag 46
          ||||||||||||||   |||||
Sbjct: 11 tgcttgattcacttctacaagag 33
```

>FK041220|FK041220.1 CF_WOO_91H09_SP6 Copepod Whole Organism, Normalized *Calanus finmarchicus* cDNA clone CF_WOO_91H09; 5' similar to ref|NP_001037203.1| innexin 2 - Bombyx mori. Score = 93.2 bits (230), expect = 1e-17, mRN Length = 674; score = 37.9 bits (17), expect = 3.8; identities = 20/23 (86%); strand = Plus / Plus

```
Query: 24 tgcttgattcacttcwwyaagag 46
          ||||||||||||||   |||||
Sbjct: 2  tgcttgattcacttctacaagag 24
```

>FK041177|FK041177.1 CF_WO0_91E01_SP6 Copepod Whole Organism, Normalized *Calanus finmarchicus* cDNA clone CF_WO0_91E01; 5’ , mRNA sequence. Length = 658; score = 37.9 bits (17), expect = 3.8; identities = 20/23 (86%); strand = Plus / Plus

Query: 24 tgcttgattcacttcwwyaagag 46
          ||||||||||||||| |||||
Sbjct: 11 tgcttgattcacttctacaagag 33

>FK041150|FK041150.1 CF_WO0_91B03_SP6 Copepod Whole Organism, Normalized *Calanus finmarchicus* cDNA clone CF_WO0_91B03; 5’ similar to ref|NP_001097240.1| Pyruvate dehydrogenase kinase CG8808-PB, isoform B [Drosophila. Score Length = 716; score = 37.9 bits (17), expect = 3.8; identities = 20/23 (86%); strand = Plus / Plus

Query: 24 tgcttgattcacttcwwyaagag 46
          ||||||||||||||| |||||
Sbjct: 35 tgcttgattcacttctacaagag 57

>FK040989|FK040989.1 CF_WO0_89A08_SP6 Copepod Whole Organism, Normalized *Calanus finmarchicus* cDNA clone CF_WO0_89A08; 5’ , mRNA sequence. Length = 635; score = 37.9 bits (17), expect = 3.8; identities = 20/23 (86%); strand = Plus / Plus

Query: 24 tgcttgattcacttcwwyaagag 46
          ||||||||||||||| |||||
Sbjct: 20 tgcttgattcacttctacaagag 42

>FK040961|FK040961.1 CF_WO0_88G02_SP6 Copepod Whole Organism, Normalized *Calanus finmarchicus* cDNA clone CF_WO0_88G02; 5’ similar to ref|XP_974421.1| DIVDICTED: similar to synoviolin - Tribolium castaneum. Score = 313 bits Length = 684; score = 37.9 bits (17), expect = 3.8; identities = 20/23 (86%); strand = Plus / Plus

Query: 16 gctwawwttgcttgattcacttc 38
          ||| | ||||||||||||||||
Sbjct: 3 gctaatattgcttgattcacttc 25

>FK040956|FK040956.1 CF_WO0_88F09_SP6 Copepod Whole Organism, Normalized *Calanus finmarchicus* cDNA clone CF_WO0_88F09; 5’ , mRNA sequence. Length = 669; score = 37.9 bits (17), expect = 3.8; identities = 20/23 (86%); strand = Plus / Plus

Query: 24 tgcttgattcacttcwwyaagag 46
          ||||||||||||||| |||||
Sbjct: 9 tgcttgattcacttctacaagag 31

>FK040845|FK040845.1 CF_WO0_87D02_SP6 Copepod Whole Organism, Normalized *Calanus finmarchicus* cDNA clone CF_WO0_87D02; 5’ similar to ref|XP_001627555.1| predicted protein - Nematostella vectensis. Score = 193 bits (490), E. Length = 697; score = 37.9 bits (17), expect = 3.8; identities = 20/23 (86%); strand = Plus / Plus

Query: 24 tgcttgattcacttcwwyaagag 46

|||||||||||||| |||||
Sbjct: 9 tgcttgattcacttctacaagag 31

>FK040813|FK040813.1 CF_WO0_87A04_SP6 Copepod Whole Organism, Normalized *Calanus finmarchicus* cDNA clone CF_WO0_87A04; 5' similar to ref|XP_969681.1| DIVDICTED: similar to spliceosome associated protein [Tribolium. Score = Length = 673; score = 37.9 bits (17), expect = 3.8; identities = 20/23 (86%); strand = Plus / Plus

Query: 24 tgcttgattcacttcwwyaagag 46
|||||||||||||| |||||
Sbjct: 14 tgcttgattcacttctacaagag 36

>FG985891|FG985891.1 CF_WO0_86F04_SP6 Copepod Whole Organism, Normalized *Calanus finmarchicus* cDNA clone CF_WO0_86F04; 5' similar to ref|XP_001605831.1| DIVDICTED: similar to aspartate aminotransferase [Nasonia. Score = 26 Length = 683; score = 37.9 bits (17), expect = 3.8; identities = 20/23 (86%); strand = Plus / Plus

Query: 24 tgcttgattcacttcwwyaagag 46
|||||||||||||| |||||
Sbjct: 2 tgcttgattcacttctacaagag 24

>FG985879|FG985879.1 CF_WO0_86E04_SP6 Copepod Whole Organism, Normalized *Calanus finmarchicus* cDNA clone CF_WO0_86E04; 5' similar to ref|XP_394615.2| DIVDICTED: similar to caseinolytic peptidase X - Apis mellifera. Score = Length = 719; score = 37.9 bits (17), expect = 3.8; identities = 20/23 (86%); strand = Plus / Plus

Query: 24 tgcttgattcacttcwwyaagag 46
|||||||||||||| |||||
Sbjct: 11 tgcttgattcacttctacaagag 33

>FG985867|FG985867.1 CF_WO0_86D04_SP6 Copepod Whole Organism, Normalized *Calanus finmarchicus* cDNA clone CF_WO0_86D04; 5' similar to ref|ZP_02169478.1| acyl esterase-like protein - Bacillus selenitireducens MLS10. Score = Length = 706; score = 37.9 bits (17), expect = 3.8; identities = 20/23 (86%); strand = Plus / Plus

Query: 24 tgcttgattcacttcwwyaagag 46
|||||||||||||| |||||
Sbjct: 8 tgcttgattcacttctacaagag 30

>FG985847|FG985847.1 CF_WO0_86B08_SP6 Copepod Whole Organism, Normalized *Calanus finmarchicus* cDNA clone CF_WO0_86B08; 5' similar to ref|XP_001896281.1| enolase - Brugia malayi. Score = 327 bits (838), expect = 4e-88, mRNA Length = 697; score = 37.9 bits (17), expect = 3.8; identities = 20/23 (86%); strand = Plus / Plus

Query: 24 tgcttgattcacttcwwyaagag 46
|||||||||||||| |||||
Sbjct: 9 tgcttgattcacttctacaagag 31

>FG985744|FG985744.1 CF_WO0_84G07_SP6 Copepod Whole Organism, Normalized *Calanus finmarchicus* cDNA clone CF_WO0_84G07; 5' similar to gb|ABD83915.1| disulfide isomerase-like - Ictalurus punctatus. Score = 79.7 bits (195), E. Length = 689; score = 37.9 bits (17), expect = 3.8; identities = 20/23 (86%); strand = Plus / Plus

```
Query: 24 tgcttgattcacttcwwyaagag 46
          ||||||||||||||   |||||
Sbjct: 2  tgcttgattcacttctacaagag 24
```

>FG985711|FG985711.1 CF_WO0_84D04_SP6 Copepod Whole Organism, Normalized *Calanus finmarchicus* cDNA clone CF_WO0_84D04; 5' , mRNA sequence. Length = 687; score = 37.9 bits (17), expect = 3.8; identities = 20/23 (86%); strand = Plus / Plus

```
Query: 24 tgcttgattcacttcwwyaagag 46
          ||||||||||||||   |||||
Sbjct: 14 tgcttgattcacttctacaagag 36
```

>FG985684|FG985684.1 CF_WO0_84A11_SP6 Copepod Whole Organism, Normalized *Calanus finmarchicus* cDNA clone CF_WO0_84A11; 5' similar to ref|XP_970433.1| DIVDICTED: similar to Protein SDA1 homolog (Mystery protein 45A). Score Length = 646; score = 37.9 bits (17), expect = 3.8; identities = 20/23 (86%); strand = Plus / Plus

```
Query: 24 tgcttgattcacttcwwyaagag 46
          ||||||||||||||   |||||
Sbjct: 2  tgcttgattcacttctacaagag 24
```

>FG985662|FG985662.1 CF_WO0_83G08_SP6 Copepod Whole Organism, Normalized *Calanus finmarchicus* cDNA clone CF_WO0_83G08; 5' similar to gb|EDV43183.1| GF16654 - Drosophila ananassae. Score = 57.4 bits (137), expect = 7e-07, m Length = 628; score = 37.9 bits (17), expect = 3.8; identities = 20/23 (86%); strand = Plus / Plus

```
Query: 24 tgcttgattcacttcwwyaagag 46
          ||||||||||||||   |||||
Sbjct: 2  tgcttgattcacttctacaagag 24
```

>FG985644|FG985644.1 CF_WO0_83E03_SP6 Copepod Whole Organism, Normalized *Calanus finmarchicus* cDNA clone CF_WO0_83E03; 5' similar to ref|XP_315051.4| AGAP004953-PA - Anopheles gambiae str. PEST. Score = 247 bits (631), Exp Length = 714; score = 37.9 bits (17), expect = 3.8; identities = 20/23 (86%); strand = Plus / Plus

```
Query: 24 tgcttgattcacttcwwyaagag 46
          ||||||||||||||   |||||
Sbjct: 2  tgcttgattcacttctacaagag 24
```

>FG985589|FG985589.1 CF_WO0_82G04_SP6 Copepod Whole Organism, Normalized *Calanus finmarchicus* cDNA clone CF_WO0_82G04; 5' similar to ref|NP_787974.3| dumpy CG33196-PB - Drosophila melanogaster. Score = 109 bits (272), Expe Length = 695; score = 37.9 bits (17), expect = 3.8; identities = 20/23 (86%); strand = Plus / Plus

```
Query: 24 tgcttgattcacttcwwyaagag 46
          ||||||||||||||   |||||
Sbjct: 2  tgcttgattcacttctacaagag 24
```

>FG985555|FG985555.1 CF_WO0_82D03_SP6 Copepod Whole Organism, Normalized *Calanus finmarchicus* cDNA clone CF_WO0_82D03; 5' similar to gb|AB021408.1| pyruvate kinase - Litopenaeus vannamei. Score = 191 bits (484), expect = 5 Length = 705; score = 37.9 bits (17), expect = 3.8; identities = 20/23 (86%); strand = Plus / Plus

```
Query: 24 tgcttgattcacttcwwyaagag 46
          ||||||||||||||   |||||
Sbjct: 43 tgcttgattcacttctacaagag 65
```

>FG985531|FG985531.1 CF_WO0_82B01_SP6 Copepod Whole Organism, Normalized *Calanus finmarchicus* cDNA clone CF_WO0_82B01; 5' , mRNA sequence. Length = 674; score = 37.9 bits (17), expect = 3.8; identities = 20/23 (86%); strand = Plus / Plus

```
Query: 24 tgcttgattcacttcwwyaagag 46
          ||||||||||||||   |||||
Sbjct: 11 tgcttgattcacttctacaagag 33
```

>FG985461|FG985461.1 CF_WO0_81C06_SP6 Copepod Whole Organism, Normalized *Calanus finmarchicus* cDNA clone CF_WO0_81C06; 5' similar to ref|XP_972366.1| DIVDICTED: similar to N-acetyl lactosaminide beta-1,3-N-acetyl. Score = Length = 678; score = 37.9 bits (17), expect = 3.8; identities = 20/23 (86%); strand = Plus / Plus

```
Query: 24 tgcttgattcacttcwwyaagag 46
          ||||||||||||||   |||||
Sbjct: 8  tgcttgattcacttctacaagag 30
```

>FG985448|FG985448.1 CF_WO0_81B02_SP6 Copepod Whole Organism, Normalized *Calanus finmarchicus* cDNA clone CF_WO0_81B02; 5' , mRNA sequence. Length = 673; score = 37.9 bits (17), expect = 3.8; identities = 20/23 (86%); strand = Plus / Plus

```
Query: 24 tgcttgattcacttcwwyaagag 46
          ||||||||||||||   |||||
Sbjct: 2  tgcttgattcacttctacaagag 24
```

>FG985420|FG985420.1 CF_WO0_80G06_SP6 Copepod Whole Organism, Normalized *Calanus finmarchicus* cDNA clone CF_WO0_80G06; 5' similar to ref|XP_001605917.1| DIVDICTED: similar to ENSANGP00000003788 - Nasonia vitripennis. Score Length = 607; score = 37.9 bits (17), expect = 3.8; identities = 20/23 (86%); strand = Plus / Plus

```
Query: 24 tgcttgattcacttcwwyaagag 46
          ||||||||||||||   |||||
Sbjct: 39 tgcttgattcacttctacaagag 61
```

>FG985407|FG985407.1 CF_WO0_80F05_SP6 Copepod Whole Organism, Normalized *Calanus finmarchicus* cDNA clone CF_WO0_80F05; 5' similar to ref|XP_001014168.1| hypothetical protein

TTHERM_00222400 [Tetrahymena thermophila. Score Length = 645; score = 37.9 bits (17), expect = 3.8; identities = 20/23 (86%); strand = Plus / Plus

```
Query: 16 gctwawwttgcttgattcacttc 38
          ||| |  ||||||||||||||||
Sbjct: 3  gctaatattgcttgattcacttc 25
```

>FG633115|FG633115.1 CF_WOO_77f07_SP6 Copepod Whole Organism, Normalized *Calanus finmarchicus* cDNA clone CF_WOO_77f07; 5' , mRNA sequence. Length = 591; score = 37.9 bits (17), expect = 3.8; identities = 20/23 (86%); strand = Plus / Plus

```
Query: 24 tgcttgattcacttcwwyaagag 46
          |||||||||||||||   |||||
Sbjct: 14 tgcttgattcacttctacaagag 36
```

>FG633046|FG633046.1 CF_WOO_76g11_SP6 Copepod Whole Organism, Normalized *Calanus finmarchicus* cDNA clone CF_WOO_76g11; 5' similar to ref|NP_001002162.1| ELL associated factor 2 - Danio rerio. Score = 111 bits (278), expect Length = 539; score = 37.9 bits (17), expect = 3.8; identities = 20/23 (86%); strand = Plus / Plus

```
Query: 24 tgcttgattcacttcwwyaagag 46
          |||||||||||||||   |||||
Sbjct: 8  tgcttgattcacttctacaagag 30
```

>FG633035|FG633035.1 CF_WOO_76f11_SP6 Copepod Whole Organism, Normalized *Calanus finmarchicus* cDNA clone CF_WOO_76f11; 5' , mRNA sequence. Length = 592; score = 37.9 bits (17), expect = 3.8; identities = 20/23 (86%); strand = Plus / Plus

```
Query: 24 tgcttgattcacttcwwyaagag 46
          |||||||||||||||   |||||
Sbjct: 10 tgcttgattcacttctacaagag 32
```

>FG633010|FG633010.1 CF_WOO_76d09_SP6 Copepod Whole Organism, Normalized *Calanus finmarchicus* cDNA clone CF_WOO_76d09; 5' similar to ref|XP_394637.2| DIVDICTED: similar to Nat1 CG12202-PA isoform 1 - Apis mellifera. Score Length = 599; score = 37.9 bits (17), expect = 3.8; identities = 20/23 (86%); strand = Plus / Plus

```
Query: 24 tgcttgattcacttcwwyaagag 46
          |||||||||||||||   |||||
Sbjct: 15 tgcttgattcacttctacaagag 37
```

>FG633009|FG633009.1 CF_WOO_76d08_SP6 Copepod Whole Organism, Normalized *Calanus finmarchicus* cDNA clone CF_WOO_76d08; 5' similar to ref|XP_394637.2| DIVDICTED: similar to Nat1 CG12202-PA isoform 1 - Apis mellifera. Score Length = 579; score = 37.9 bits (17), expect = 3.8; identities = 20/23 (86%); strand = Plus / Plus

```
Query: 24 tgcttgattcacttcwwyaagag 46
          |||||||||||||||   |||||
Sbjct: 10 tgcttgattcacttctacaagag 32
```

>FG632993|FG632993.1 CF_WO0_76c02_SP6 Copepod Whole Organism, Normalized *Calanus finmarchicus* cDNA clone CF_WO0_76c02; 5' similar to ref|XP_969608.1| DIVDICTED: similar to Integrator complex subunit 7 (Int7). Score = 261 b Length = 693; score = 37.9 bits (17), expect = 3.8; identities = 20/23 (86%); strand = Plus / Plus

```
Query: 24 tgcttgattcacttcwwyaagag 46
          ||||||||||||||   |||||
Sbjct: 4  tgcttgattcacttctacaagag 26
```

>FG632948|FG632948.1 CF_WO0_75g03_SP6 Copepod Whole Organism, Normalized *Calanus finmarchicus* cDNA clone CF_WO0_75g03; 5' similar to gb|EDL09766.1| casein kinase 1, alpha 1, isoform CRA_d - Mus musculus. Score = 192 bits (Length = 697; score = 37.9 bits (17), expect = 3.8; identities = 20/23 (86%); strand = Plus / Plus

```
Query: 24 tgcttgattcacttcwwyaagag 46
          ||||||||||||||   |||||
Sbjct: 1  tgcttgattcacttctacaagag 23
```

>FG632930|FG632930.1 CF_WO0_75e09_SP6 Copepod Whole Organism, Normalized *Calanus finmarchicus* cDNA clone CF_WO0_75e09; 5', mRNA sequence. Length = 696; score = 37.9 bits (17), expect = 3.8; identities = 20/23 (86%); strand = Plus / Plus

```
Query: 24 tgcttgattcacttcwwyaagag 46
          ||||||||||||||   |||||
Sbjct: 14 tgcttgattcacttctacaagag 36
```

>FG632928|FG632928.1 CF_WO0_75e07_SP6 Copepod Whole Organism, Normalized *Calanus finmarchicus* cDNA clone CF_WO0_75e07; 5', mRNA sequence. Length = 660; score = 37.9 bits (17), expect = 3.8; identities = 20/23 (86%); strand = Plus / Plus

```
Query: 24 tgcttgattcacttcwwyaagag 46
          ||||||||||||||   |||||
Sbjct: 9  tgcttgattcacttctacaagag 31
```

>FG632918|FG632918.1 CF_WO0_75d07_SP6 Copepod Whole Organism, Normalized *Calanus finmarchicus* cDNA clone CF_WO0_75d07; 5' similar to emb|CAG08303.1| unnamed protein product - Tetraodon nigroviridis. Score = 237 bits (604), Length = 667; score = 37.9 bits (17), expect = 3.8; identities = 20/23 (86%); strand = Plus / Plus

```
Query: 24 tgcttgattcacttcwwyaagag 46
          ||||||||||||||   |||||
Sbjct: 14 tgcttgattcacttctacaagag 36
```

>FG632891|FG632891.1 CF_WO0_75b02_SP6 Copepod Whole Organism, Normalized *Calanus finmarchicus* cDNA clone CF_WO0_75b02; 5' similar to ref|XP_968859.1| DIVDICTED: similar to coiled-coil domain-containing protein 76. Score = Length = 685; score = 37.9 bits (17), expect = 3.8; identities = 20/23 (86%); strand = Plus / Plus

```
Query: 24 tgcttgattcacttcwwyaagag 46
          ||||||||||||||   |||||
Sbjct: 10 tgcttgattcacttctacaagag 32
```

>FG632815|FG632815.1 CF_W00_74c05_SP6 Copepod Whole Organism, Normalized *Calanus finmarchicus* cDNA clone CF_W00_74c05; 5' similar to gb|EDL24762.1| RFT1 homolog (S. cerevisiae) - Mus musculus. Score = 118 bits (295), Expec Length = 647; score = 37.9 bits (17), expect = 3.8; identities = 20/23 (86%); strand = Plus / Plus

```
Query: 24 tgcttgattcacttcwwyaagag 46
          ||||||||||||||   |||||
Sbjct: 12 tgcttgattcacttctacaagag 34
```

>FG632751|FG632751.1 CF_W00_73e12_SP6 Copepod Whole Organism, Normalized *Calanus finmarchicus* cDNA clone CF_W00_73e12; 5' similar to ref|XP_417682.1| DIVDICTED: similar to mitochondrial solute carrier protein [Gallus. Scor Length = 655; score = 37.9 bits (17), expect = 3.8; identities = 20/23 (86%); strand = Plus / Plus

```
Query: 24 tgcttgattcacttcwwyaagag 46
          ||||||||||||||   |||||
Sbjct: 9  tgcttgattcacttctacaagag 31
```

>FG632735|FG632735.1 CF_W00_73d08_SP6 Copepod Whole Organism, Normalized *Calanus finmarchicus* cDNA clone CF_W00_73d08; 5' similar to ref|XP_968541.1| DIVDICTED: similar to predicted protein - Tribolium castaneum. Score = 2 Length = 681; score = 37.9 bits (17), expect = 3.8; identities = 20/23 (86%); strand = Plus / Plus

```
Query: 24 tgcttgattcacttcwwyaagag 46
          ||||||||||||||   |||||
Sbjct: 2  tgcttgattcacttctacaagag 24
```

>FG632604|FG632604.1 CF_W00_71h11_SP6 Copepod Whole Organism, Normalized *Calanus finmarchicus* cDNA clone CF_W00_71h11; 5' similar to ref|XP_308965.3| AGAP006780-PA - Anopheles gambiae str. PEST. Score = 278 bits (710), Exp Length = 693; score = 37.9 bits (17), expect = 3.8; identities = 20/23 (86%); strand = Plus / Plus

```
Query: 24 tgcttgattcacttcwwyaagag 46
          ||||||||||||||   |||||
Sbjct: 13 tgcttgattcacttctacaagag 35
```

>FG632572|FG632572.1 CF_W00_71f02_SP6 Copepod Whole Organism, Normalized *Calanus finmarchicus* cDNA clone CF_W00_71f02; 5', mRNA sequence. Length = 718; score = 37.9 bits (17), expect = 3.8; identities = 20/23 (86%); strand = Plus / Plus

```
Query: 24 tgcttgattcacttcwwyaagag 46
          ||||||||||||||   |||||
Sbjct: 3  tgcttgattcacttctacaagag 25
```

>FG632569|FG632569.1 CF_WO0_71e11_SP6 Copepod Whole Organism, Normalized *Calanus finmarchicus* cDNA clone CF_WO0_71e11; 5' similar to ref|XP_624673.2| DIVDICTED: similar to P-element somatic inhibitor CG8912-PB,. Score = 10 Length = 690; score = 37.9 bits (17), expect = 3.8; identities = 20/23 (86%); strand = Plus / Plus

```
Query: 24 tgcttgattcacttcwwyaagag 46
          ||||||||||||||   |||||
Sbjct: 13 tgcttgattcacttctacaagag 35
```

>FG632568|FG632568.1 CF_WO0_71e10_SP6 Copepod Whole Organism, Normalized *Calanus finmarchicus* cDNA clone CF_WO0_71e10; 5' , mRNA sequence. Length = 664; score = 37.9 bits (17), expect = 3.8; identities = 20/23 (86%); strand = Plus / Plus

```
Query: 24 tgcttgattcacttcwwyaagag 46
          ||||||||||||||   |||||
Sbjct: 9  tgcttgattcacttctacaagag 31
```

>FG632551|FG632551.1 CF_WO0_71d05_SP6 Copepod Whole Organism, Normalized *Calanus finmarchicus* cDNA clone CF_WO0_71d05; 5' similar to ref|XP_001846964.1| tubulin gamma-1 chain - Culex pipiens quinquefasciatus. Score = 348 b Length = 711; score = 37.9 bits (17), expect = 3.8; identities = 20/23 (86%); strand = Plus / Plus

```
Query: 24 tgcttgattcacttcwwyaagag 46
          ||||||||||||||   |||||
Sbjct: 15 tgcttgattcacttctacaagag 37
```

>FG632512|FG632512.1 CF_WO0_71a01_SP6 Copepod Whole Organism, Normalized *Calanus finmarchicus* cDNA clone CF_WO0_71a01; 5' similar to emb|CAG07918.1| unnamed protein product - Tetraodon nigroviridis. Score = 272 bits (695), Length = 640; score = 37.9 bits (17), expect = 3.8; identities = 20/23 (86%); strand = Plus / Plus

```
Query: 24 tgcttgattcacttcwwyaagag 46
          ||||||||||||||   |||||
Sbjct: 2  tgcttgattcacttctacaagag 24
```

>FG343041|FG343041.1 CF_WO0_70e07_SP6 Copepod Whole Organism, Normalized *Calanus finmarchicus* cDNA clone CF_WO0_70e07; 5' similar to emb|CAI12034.1| MCM3 minichromosome maintenance deficient 3 (S. cerevisiae) [Danio. Score Length = 676; score = 37.9 bits (17), expect = 3.8; identities = 20/23 (86%); strand = Plus / Plus

```
Query: 24 tgcttgattcacttcwwyaagag 46
          ||||||||||||||   |||||
Sbjct: 13 tgcttgattcacttctacaagag 35
```

>FG343031|FG343031.1 CF_WO0_70d09_SP6 Copepod Whole Organism, Normalized *Calanus finmarchicus* cDNA clone CF_WO0_70d09; 5' similar to ref|XP_971368.1| DIVDICTED: similar to CG4260-PA, isoform A - Tribolium castaneum. Score Length = 672; score = 37.9 bits (17), expect = 3.8; identities = 20/23 (86%); strand = Plus / Plus

```
Query: 24 tgcttgattcacttcwwyaagag 46
          |||||||||||||||   |||||
Sbjct: 10 tgcttgattcacttctacaagag 32
```

>FG343025|FG343025.1 CF_WO0_70d03_SP6 Copepod Whole Organism, Normalized *Calanus finmarchicus* cDNA clone CF_WO0_70d03; 5’ similar to ref|XP_001605870.1| DIVDICTED: similar to CG5522-PD - Nasonia vitripennis. Score = 60.8 b Length = 650; score = 37.9 bits (17), expect = 3.8; identities = 20/23 (86%); strand = Plus / Plus

```
Query: 24 tgcttgattcacttcwwyaagag 46
          |||||||||||||||   |||||
Sbjct: 2  tgcttgattcacttctacaagag 24
```

>FG343021|FG343021.1 CF_WO0_70c11_SP6 Copepod Whole Organism, Normalized *Calanus finmarchicus* cDNA clone CF_WO0_70c11; 5’ , mRNA sequence. Length = 643; score = 37.9 bits (17), expect = 3.8; identities = 20/23 (86%); strand = Plus / Plus

```
Query: 24 tgcttgattcacttcwwyaagag 46
          |||||||||||||||   |||||
Sbjct: 2  tgcttgattcacttctacaagag 24
```

>FG342998|FG342998.1 CF_WO0_70a11_SP6 Copepod Whole Organism, Normalized *Calanus finmarchicus* cDNA clone CF_WO0_70a11; 5’ similar to ref|XP_001601807.1| DIVDICTED: similar to ENSANGP00000003967 - Nasonia vitripennis. Score Length = 665; score = 37.9 bits (17), expect = 3.8; identities = 20/23 (86%); strand = Plus / Plus

```
Query: 24 tgcttgattcacttcwwyaagag 46
          |||||||||||||||   |||||
Sbjct: 2  tgcttgattcacttctacaagag 24
```

>FG342956|FG342956.1 CF_WO0_69f05_SP6 Copepod Whole Organism, Normalized *Calanus finmarchicus* cDNA clone CF_WO0_69f05; 5’ similar to ref|XP_318380.4| AGAP003928-PA - Anopheles gambiae str. PEST. Score = 205 bits (522), Exp Length = 697; score = 37.9 bits (17), expect = 3.8; identities = 20/23 (86%); strand = Plus / Plus

```
Query: 24 tgcttgattcacttcwwyaagag 46
          |||||||||||||||   |||||
Sbjct: 10 tgcttgattcacttctacaagag 32
```

>FG342955|FG342955.1 CF_WO0_69f04_SP6 Copepod Whole Organism, Normalized *Calanus finmarchicus* cDNA clone CF_WO0_69f04; 5’ similar to ref|XP_318380.4| AGAP003928-PA - Anopheles gambiae str. PEST. Score = 207 bits (526), Exp Length = 586; score = 37.9 bits (17), expect = 3.8; identities = 20/23 (86%); strand = Plus / Plus

```
Query: 24 tgcttgattcacttcwwyaagag 46
          |||||||||||||||   |||||
Sbjct: 10 tgcttgattcacttctacaagag 32
```

>FG342876|FG342876.1 CF_W00_68g04_SP6 Copepod Whole Organism, Normalized *Calanus finmarchicus* cDNA clone CF_W00_68g04; 5' similar to ref|XP_624707.1| DIVDICTED: similar to CG6904-PA, isoform A - Apis mellifera. Score = 251 Length = 724; score = 37.9 bits (17), expect = 3.8; identities = 20/23 (86%); strand = Plus / Plus

```
Query: 24 tgcttgattcacttcwwyaagag 46
          |||||||||||||||   |||||
Sbjct: 14 tgcttgattcacttctacaagag 36
```

>FG342856|FG342856.1 CF_W00_68e08_SP6 Copepod Whole Organism, Normalized *Calanus finmarchicus* cDNA clone CF_W00_68e08; 5' similar to gb|AAH65324.1| Cct4 protein - Danio rerio. Score = 242 bits (618), expect = 1e-62, mRNA s Length = 655; score = 37.9 bits (17), expect = 3.8; identities = 20/23 (86%); strand = Plus / Plus

```
Query: 16 gctwawwttgcttgattcacttc 38
          ||| |  ||||||||||||||||
Sbjct: 1  gctaatattgcttgattcacttc 23
```

>FG342847|FG342847.1 CF_W00_68d10_SP6 Copepod Whole Organism, Normalized *Calanus finmarchicus* cDNA clone CF_W00_68d10; 5' similar to ref|XP_971368.1| DIVDICTED: similar to CG4260-PA, isoform A - Tribolium castaneum. Score Length = 705; score = 37.9 bits (17), expect = 3.8; identities = 20/23 (86%); strand = Plus / Plus

```
Query: 24 tgcttgattcacttcwwyaagag 46
          |||||||||||||||   |||||
Sbjct: 10 tgcttgattcacttctacaagag 32
```

>FG342820|FG342820.1 CF_W00_68b06_SP6 Copepod Whole Organism, Normalized *Calanus finmarchicus* cDNA clone CF_W00_68b06; 5', mRNA sequence. Length = 665; score = 37.9 bits (17), expect = 3.8; identities = 20/23 (86%); strand = Plus / Plus

```
Query: 16 gctwawwttgcttgattcacttc 38
          ||| |  ||||||||||||||||
Sbjct: 14 gctaatattgcttgattcacttc 36
```

>FG342738|FG342738.1 CF_W00_67c03_SP6 Copepod Whole Organism, Normalized *Calanus finmarchicus* cDNA clone CF_W00_67c03; 5' similar to ref|XP_001472301.1| DIVDICTED: similar to novel KRAB box containing protein [Mus. Score = Length = 574; score = 37.9 bits (17), expect = 3.8; identities = 20/23 (86%); strand = Plus / Plus

```
Query: 24 tgcttgattcacttcwwyaagag 46
          |||||||||||||||   |||||
Sbjct: 11 tgcttgattcacttctacaagag 33
```

>FG342730|FG342730.1 CF_W00_67b07_SP6 Copepod Whole Organism, Normalized *Calanus finmarchicus* cDNA clone CF_W00_67b07; 5' similar to ref|XP_001655207.1| transcription factor IIIB 90 kDa subunit (TFIIIB90) - Aedes aegypti. Length = 602; score = 37.9 bits (17), expect = 3.8; identities = 20/23 (86%); strand = Plus / Plus

Query: 24 tgcttgattcacttcwwyaagag 46
         |||||||||||||| |||||
Sbjct: 12 tgcttgattcacttctacaagag 34

>FG342729|FG342729.1 CF_WO0_67b06_SP6 Copepod Whole Organism, Normalized *Calanus finmarchicus* cDNA clone CF_WO0_67b06; 5' similar to ref|XP_001607399.1| DIVDICTED: similar to transcription factor IIIB 90 kDa subunit. Score Length = 691; score = 37.9 bits (17), expect = 3.8; identities = 20/23 (86%); strand = Plus / Plus

Query: 24 tgcttgattcacttcwwyaagag 46
         |||||||||||||| |||||
Sbjct: 12 tgcttgattcacttctacaagag 34

>FG342642|FG342642.1 CF_WO0_66b08_SP6 Copepod Whole Organism, Normalized *Calanus finmarchicus* cDNA clone CF_WO0_66b08; 5' similar to ref|XP_001606514.1| DIVDICTED: similar to ENSANGP00000019521 - Nasonia vitripennis. Score Length = 697; score = 37.9 bits (17), expect = 3.8; identities = 20/23 (86%); strand = Plus / Plus

Query: 24 tgcttgattcacttcwwyaagag 46
         |||||||||||||| |||||
Sbjct: 10 tgcttgattcacttctacaagag 32

>FG342568|FG342568.1 CF_WO0_65d04_SP6 Copepod Whole Organism, Normalized *Calanus finmarchicus* cDNA clone CF_WO0_65d04; 5' similar to ref|XP_001603984.1| DIVDICTED: similar to innexin-1 - Nasonia vitripennis. Score = 124 bi Length = 665; score = 37.9 bits (17), expect = 3.8; identities = 20/23 (86%); strand = Plus / Plus

Query: 24 tgcttgattcacttcwwyaagag 46
         |||||||||||||| |||||
Sbjct: 10 tgcttgattcacttctacaagag 32

>FG342564|FG342564.1 CF_WO0_65c12_SP6 Copepod Whole Organism, Normalized *Calanus finmarchicus* cDNA clone CF_WO0_65c12; 5', mRNA sequence. Length = 545; score = 37.9 bits (17), expect = 3.8; identities = 20/23 (86%); strand = Plus / Plus

Query: 24 tgcttgattcacttcwwyaagag 46
         |||||||||||||| |||||
Sbjct: 15 tgcttgattcacttctacaagag 37

>FG342563|FG342563.1 CF_WO0_65c11_SP6 Copepod Whole Organism, Normalized *Calanus finmarchicus* cDNA clone CF_WO0_65c11; 5', mRNA sequence. Length = 642; score = 37.9 bits (17), expect = 3.8; identities = 20/23 (86%); strand = Plus / Plus

Query: 24 tgcttgattcacttcwwyaagag 46
         |||||||||||||| |||||
Sbjct: 15 tgcttgattcacttctacaagag 37

>FG342524|FG342524.1 CF_WO0_64h03_SP6 Copepod Whole Organism, Normalized *Calanus finmarchicus* cDNA clone CF_WO0_64h03; 5' similar to ref|XP_001602632.1| DIVDICTED: similar to LOC496040

protein - Nasonia vitripennis. Score Length = 600; score = 37.9 bits (17), expect = 3.8; identities = 20/23 (86%); strand = Plus / Plus

```
Query: 24 tgcttgattcacttcwwyaagag 46
          ||||||||||||||   |||||
Sbjct: 6  tgcttgattcacttctacaagag 28
```

>FG342467|FG342467.1 CF_WO0_64b06_SP6 Copepod Whole Organism, Normalized *Calanus finmarchicus* cDNA clone CF_WO0_64b06; 5' similar to ref|XP_969227.1| DIVDICTED: similar to CG7638-PA - Tribolium castaneum. Score = 129 bits Length = 656; score = 37.9 bits (17), expect = 3.8; identities = 20/23 (86%); strand = Plus / Plus

```
Query: 16 gctwawwttgcttgattcacttc 38
          ||| |  |||||||||||||||
Sbjct: 3  gctaatattgcttgattcacttc 25
```

>FG342361|FG342361.1 CF_WO0_62h12_SP6 Copepod Whole Organism, Normalized *Calanus finmarchicus* cDNA clone CF_WO0_62h12; 5' , mRNA sequence. Length = 598; score = 37.9 bits (17), expect = 3.8; identities = 20/23 (86%); strand = Plus / Plus

```
Query: 24 tgcttgattcacttcwwyaagag 46
          ||||||||||||||   |||||
Sbjct: 1  tgcttgattcacttctacaagag 23
```

>FG342243|FG342243.1 CF_WO0_61f11_SP6 Copepod Whole Organism, Normalized *Calanus finmarchicus* cDNA clone CF_WO0_61f11; 5' similar to ref|XP_321691.4| AGAP001440-PA - Anopheles gambiae str. PEST. Score = 151 bits (382), Exp Length = 697; score = 37.9 bits (17), expect = 3.8; identities = 20/23 (86%); strand = Plus / Plus

```
Query: 24 tgcttgattcacttcwwyaagag 46
          ||||||||||||||   |||||
Sbjct: 10 tgcttgattcacttctacaagag 32
```

>FG342238|FG342238.1 CF_WO0_61f06_SP6 Copepod Whole Organism, Normalized *Calanus finmarchicus* cDNA clone CF_WO0_61f06; 5' similar to sp|Q9D2N9|VP33A_MOUSE Vacuolar protein sorting-associated protein 33A. Score = 193 bits (Length = 674; score = 37.9 bits (17), expect = 3.8; identities = 20/23 (86%); strand = Plus / Plus

```
Query: 24 tgcttgattcacttcwwyaagag 46
          ||||||||||||||   |||||
Sbjct: 15 tgcttgattcacttctacaagag 37
```

>FG342230|FG342230.1 CF_WO0_61e10_SP6 Copepod Whole Organism, Normalized *Calanus finmarchicus* cDNA clone CF_WO0_61e10; 5' similar to ref|NP_990307.1| vitamin D3 hydroxylase associated protein - Gallus gallus. Score = 70.9 Length = 598; score = 37.9 bits (17), expect = 3.8; identities = 20/23 (86%); strand = Plus / Plus

```
Query: 24 tgcttgattcacttcwwyaagag 46
          ||||||||||||||   |||||
```

Sbjct: 2 tgcttgattcacttctacaagag 24

>FG342222|FG342222.1 CF_WO0_61e02_SP6 Copepod Whole Organism, Normalized *Calanus finmarchicus* cDNA clone CF_WO0_61e02; 5' , mRNA sequence. Length = 673; score = 37.9 bits (17), expect = 3.8; identities = 20/23 (86%); strand = Plus / Plus

```
Query: 24 tgcttgattcacttcwwyaagag 46
          ||||||||||||||   |||||
Sbjct: 10 tgcttgattcacttctacaagag 32
```

>FG342194|FG342194.1 CF_WO0_61b10_SP6 Copepod Whole Organism, Normalized *Calanus finmarchicus* cDNA clone CF_WO0_61b10; 5' similar to ref|NP_649340.1| CG11306 CG11306-PA - Drosophila melanogaster. Score = 222 bits (565), Ex Length = 652; score = 37.9 bits (17), expect = 3.8; identities = 20/23 (86%); strand = Plus / Plus

```
Query: 24 tgcttgattcacttcwwyaagag 46
          ||||||||||||||   |||||
Sbjct: 10 tgcttgattcacttctacaagag 32
```

>FG342193|FG342193.1 CF_WO0_61b09_SP6 Copepod Whole Organism, Normalized *Calanus finmarchicus* cDNA clone CF_WO0_61b09; 5' similar to ref|XP_393347.2| DIVDICTED: similar to CG3527-PA - Apis mellifera. Score = 229 bits (585) Length = 722; score = 37.9 bits (17), expect = 3.8; identities = 20/23 (86%); strand = Plus / Plus

```
Query: 24 tgcttgattcacttcwwyaagag 46
          ||||||||||||||   |||||
Sbjct: 11 tgcttgattcacttctacaagag 33
```

>ES237849|ES237849.1 CF_WO0_60f10_SP6 Copepod Whole Organism, Normalized *Calanus finmarchicus* cDNA clone CF_WO0_60f10; 5' similar to pdb|1O1R|B Chain B, Structure Of Fpt Bound To Ggpp. Score = 193 bits (490), expect = 5e-4 Length = 593; score = 37.9 bits (17), expect = 3.8; identities = 20/23 (86%); strand = Plus / Plus

```
Query: 24 tgcttgattcacttcwwyaagag 46
          ||||||||||||||   |||||
Sbjct: 13 tgcttgattcacttctacaagag 35
```

>ES237847|ES237847.1 CF_WO0_60f08_SP6 Copepod Whole Organism, Normalized *Calanus finmarchicus* cDNA clone CF_WO0_60f08; 5' similar to gb|EAT46272.1| protein disulfide isomerase - Aedes aegypti. Score = 185 bits (469), Expec Length = 631; score = 37.9 bits (17), expect = 3.8; identities = 20/23 (86%); strand = Plus / Plus

```
Query: 24 tgcttgattcacttcwwyaagag 46
          ||||||||||||||   |||||
Sbjct: 11 tgcttgattcacttctataagag 33
```

>ES237778|ES237778.1 CF_WO0_59h08_SP6 Copepod Whole Organism, Normalized *Calanus finmarchicus* cDNA clone CF_WO0_59h08; 5' similar to ref|XP_976099.1| DIVDICTED: similar to CG6016-PB,

isoform B isoform 3 [Tribolium. Score = Length = 685; score = 37.9 bits (17), expect = 3.8; identities = 20/23 (86%); strand = Plus / Plus

```
Query: 24 tgcttgattcacttcwwyaagag 46
          ||||||||||||||   |||||
Sbjct: 11 tgcttgattcacttctacaagag 33
```

>ES237581|ES237581.1 CF_WO0_57g10_SP6 Copepod Whole Organism, Normalized *Calanus finmarchicus* cDNA clone CF_WO0_57g10; 5' similar to emb|CAG10633.1| unnamed protein product - Tetraodon nigroviridis. Score = 129 bits (325), Length = 634; score = 37.9 bits (17), expect = 3.8; identities = 20/23 (86%); strand = Plus / Plus

```
Query: 24 tgcttgattcacttcwwyaagag 46
          ||||||||||||||   |||||
Sbjct: 2  tgcttgattcacttctacaagag 24
```

>ES237575|ES237575.1 CF_WO0_57g04_SP6 Copepod Whole Organism, Normalized *Calanus finmarchicus* cDNA clone CF_WO0_57g04; 5' , mRNA sequence. Length = 720; score = 37.9 bits (17), expect = 3.8; identities = 20/23 (86%); strand = Plus / Plus

```
Query: 24 tgcttgattcacttcwwyaagag 46
          ||||||||||||||   |||||
Sbjct: 10 tgcttgattcacttctacaagag 32
```

>ES237553|ES237553.1 CF_WO0_57e06_SP6 Copepod Whole Organism, Normalized *Calanus finmarchicus* cDNA clone CF_WO0_57e06; 5' , mRNA sequence. Length = 718; score = 37.9 bits (17), expect = 3.8; identities = 20/23 (86%); strand = Plus / Plus

```
Query: 24 tgcttgattcacttcwwyaagag 46
          ||||||||||||||   |||||
Sbjct: 11 tgcttgattcacttctacaagag 33
```

>ES237531|ES237531.1 CF_WO0_57c08_SP6 Copepod Whole Organism, Normalized *Calanus finmarchicus* cDNA clone CF_WO0_57c08; 5' , mRNA sequence. Length = 707; score = 37.9 bits (17), expect = 3.8; identities = 20/23 (86%); strand = Plus / Plus

```
Query: 24 tgcttgattcacttcwwyaagag 46
          ||||||||||||||   |||||
Sbjct: 2  tgcttgattcacttctacaagag 24
```

>ES237530|ES237530.1 CF_WO0_57c07_SP6 Copepod Whole Organism, Normalized *Calanus finmarchicus* cDNA clone CF_WO0_57c07; 5' , mRNA sequence. Length = 674; score = 37.9 bits (17), expect = 3.8; identities = 20/23 (86%); strand = Plus / Plus

```
Query: 24 tgcttgattcacttcwwyaagag 46
          ||||||||||||||   |||||
Sbjct: 9  tgcttgattcacttctacaagag 31
```

>ES237529|ES237529.1 CF_WO0_57c06_SP6 Copepod Whole Organism, Normalized *Calanus finmarchicus* cDNA clone CF_WO0_57c06; 5' similar to ref|XP_001376745.1| DIVDICTED: similar to RCC1-like protein - Monodelphis domestica. Scor Length = 689; score = 37.9 bits (17), expect = 3.8; identities = 20/23 (86%); strand = Plus / Plus

```
Query: 24 tgcttgattcacttcwwyaagag 46
          ||||||||||||||   |||||
Sbjct: 2  tgcttgattcacttctacaagag 24
```

>ES237497|ES237497.1 CF_WO0_56h08_SP6 Copepod Whole Organism, Normalized *Calanus finmarchicus* cDNA clone CF_WO0_56h08; 5', mRNA sequence. Length = 719; score = 37.9 bits (17), expect = 3.8; identities = 20/23 (86%); strand = Plus / Plus

```
Query: 24 tgcttgattcacttcwwyaagag 46
          ||||||||||||||   |||||
Sbjct: 2  tgcttgattcacttctacaagag 24
```

>ES237459|ES237459.1 CF_WO0_56e05_SP6 Copepod Whole Organism, Normalized *Calanus finmarchicus* cDNA clone CF_WO0_56e05; 5' similar to ref|XP_972481.1| DIVDICTED: similar to CG11154-PA, isoform A - Tribolium castaneum. Score Length = 681; score = 37.9 bits (17), expect = 3.8; identities = 20/23 (86%); strand = Plus / Plus

```
Query: 24 tgcttgattcacttcwwyaagag 46
          ||||||||||||||   |||||
Sbjct: 11 tgcttgattcacttctacaagag 33
```

>ES237398|ES237398.1 CF_WO0_55h04_SP6 Copepod Whole Organism, Normalized *Calanus finmarchicus* cDNA clone CF_WO0_55h04; 5' similar to gb|AAH75179.1| Polr3c-prov protein - Xenopus laevis. Score = 97.1 bits (240), expect = 6e Length = 651; score = 37.9 bits (17), expect = 3.8; identities = 20/23 (86%); strand = Plus / Plus

```
Query: 24 tgcttgattcacttcwwyaagag 46
          ||||||||||||||   |||||
Sbjct: 2  tgcttgattcacttctacaagag 24
```

>ES237371|ES237371.1 CF_WO0_55f01_SP6 Copepod Whole Organism, Normalized *Calanus finmarchicus* cDNA clone CF_WO0_55f01; 5', mRNA sequence. Length = 592; score = 37.9 bits (17), expect = 3.8; identities = 20/23 (86%); strand = Plus / Plus

```
Query: 24 tgcttgattcacttcwwyaagag 46
          ||||||||||||||   |||||
Sbjct: 6  tgcttgattcacttctacaagag 28
```

>ES237363|ES237363.1 CF_WO0_55e05_SP6 Copepod Whole Organism, Normalized *Calanus finmarchicus* cDNA clone CF_WO0_55e05; 5' similar to ref|XP_394952.3| DIVDICTED: similar to GTPase-activating protein 69C CG4237-PA [Apis. Sco Length = 687; score = 37.9 bits (17), expect = 3.8; identities = 20/23 (86%); strand = Plus / Plus

```
Query: 24 tgcttgattcacttcwwyaagag 46
```

```
         |||||||||||||||   |||||
Sbjct: 42 tgcttgattcacttctacaagag 64
```

>ES237359|ES237359.1 CF_WO0_55e01_SP6 Copepod Whole Organism, Normalized *Calanus finmarchicus* cDNA clone CF_WO0_55e01; 5' similar to ref|NP_001008015.1| rnf13 protein - Xenopus tropicalis. Score = 66.2 bits (160), expect = Length = 688; score = 37.9 bits (17), expect = 3.8; identities = 20/23 (86%); strand = Plus / Plus

```
Query: 24 tgcttgattcacttcwwyaagag 46
          |||||||||||||||   |||||
Sbjct: 1  tgcttgattcacttctacaagag 23
```

>ES237315|ES237315.1 CF_WO0_55a05_SP6 Copepod Whole Organism, Normalized *Calanus finmarchicus* cDNA clone CF_WO0_55a05; 5' similar to ref|NP_001073456.1| hypothetical protein LOC561208 - Danio rerio. Score = 178 bits (452), Length = 713; score = 37.9 bits (17), expect = 3.8; identities = 20/23 (86%); strand = Plus / Plus

```
Query: 24 tgcttgattcacttcwwyaagag 46
          |||||||||||||||   |||||
Sbjct: 15 tgcttgattcacttctacaagag 37
```

>EL966262|EL966262.1 CF_WO0_54h04_SP6 Copepod Whole Organism, Normalized *Calanus finmarchicus* cDNA clone CF_WO0_54h04; 5', mRNA sequence. Length = 726; score = 37.9 bits (17), expect = 3.8; identities = 20/23 (86%); strand = Plus / Plus

```
Query: 24 tgcttgattcacttcwwyaagag 46
          |||||||||||||||   |||||
Sbjct: 9  tgcttgattcacttctacaagag 31
```

>EL966069|EL966069.1 CF_WO0_52g08_SP6 Copepod Whole Organism, Normalized *Calanus finmarchicus* cDNA clone CF_WO0_52g08; 5' similar to ref|XP_968588.1| DIVDICTED: similar to G protein pathway suppressor 1 isoform 2. Score = Length = 729; score = 37.9 bits (17), expect = 3.8; identities = 20/23 (86%); strand = Plus / Plus

```
Query: 24 tgcttgattcacttcwwyaagag 46
          |||||||||||||||   |||||
Sbjct: 10 tgcttgattcacttctacaagag 32
```

>EL966035|EL966035.1 CF_WO0_52d07_SP6 Copepod Whole Organism, Normalized *Calanus finmarchicus* cDNA clone CF_WO0_52d07; 5' similar to ref|XP_975557.1| DIVDICTED: similar to CG9060-PA - Tribolium castaneum. Score = 244 bits Length = 644; score = 37.9 bits (17), expect = 3.8; identities = 20/23 (86%); strand = Plus / Plus

```
Query: 24 tgcttgattcacttcwwyaagag 46
          |||||||||||||||   |||||
Sbjct: 2  tgcttgattcacttctacaagag 24
```

>EL966015|EL966015.1 CF_WO0_52b10_SP6 Copepod Whole Organism, Normalized *Calanus finmarchicus* cDNA clone CF_WO0_52b10; 5' , mRNA sequence. Length = 705; score = 37.9 bits (17), expect = 3.8; identities = 20/23 (86%); strand = Plus / Plus

```
Query: 24 tgcttgattcacttcwwyaagag 46
          |||||||||||||||   |||||
Sbjct: 2  tgcttgattcacttctacaagag 24
```

>EL965990|EL965990.1 CF_WO0_51h09_SP6 Copepod Whole Organism, Normalized *Calanus finmarchicus* cDNA clone CF_WO0_51h09; 5' , mRNA sequence. Length = 506; score = 37.9 bits (17), expect = 3.8; identities = 20/23 (86%); strand = Plus / Plus

```
Query: 24 tgcttgattcacttcwwyaagag 46
          |||||||||||||||   |||||
Sbjct: 14 tgcttgattcacttctacaagag 36
```

>EL965975|EL965975.1 CF_WO0_51g06_SP6 Copepod Whole Organism, Normalized *Calanus finmarchicus* cDNA clone CF_WO0_51g06; 5' similar to ref|XP_623061.1| DIVDICTED: similar to CG4747-PA, partial - Apis mellifera. Score = 107 b Length = 701; score = 37.9 bits (17), expect = 3.8; identities = 20/23 (86%); strand = Plus / Plus

```
Query: 24 tgcttgattcacttcwwyaagag 46
          |||||||||||||||   |||||
Sbjct: 2  tgcttgattcacttctacaagag 24
```

>EL965820|EL965820.1 CF_WO0_50b04_SP6 Copepod Whole Organism, Normalized *Calanus finmarchicus* cDNA clone CF_WO0_50b04; 5' similar to gb|EAT35255.1| aspartate aminotransferase - Aedes aegypti. Score = 265 bits (677), expect Length = 687; score = 37.9 bits (17), expect = 3.8; identities = 20/23 (86%); strand = Plus / Plus

```
Query: 24 tgcttgattcacttcwwyaagag 46
          |||||||||||||||   |||||
Sbjct: 2  tgcttgattcacttctacaagag 24
```

>EL965767|EL965767.1 CF_WO0_49e10_SP6 Copepod Whole Organism, Normalized *Calanus finmarchicus* cDNA clone CF_WO0_49e10; 5' similar to ref|XP_966693.1| DIVDICTED: similar to CG17332-PA, isoform A isoform 1 [Tribolium. Score Length = 710; score = 37.9 bits (17), expect = 3.8; identities = 20/23 (86%); strand = Plus / Plus

```
Query: 24 tgcttgattcacttcwwyaagag 46
          |||||||||||||||   |||||
Sbjct: 2  tgcttgattcacttctacaagag 24
```

>EL965727|EL965727.1 CF_WO0_49b05_SP6 Copepod Whole Organism, Normalized *Calanus finmarchicus* cDNA clone CF_WO0_49b05; 5' , mRNA sequence. Length = 661; score = 37.9 bits (17), expect = 3.8; identities = 20/23 (86%); strand = Plus / Plus

```
Query: 24 tgcttgattcacttcwwyaagag 46
          |||||||||||||||   |||||
```

Sbjct: 11 tgcttgattcacttctacaagag 33

>EL965720|EL965720.1 CF_WO0_49a09_SP6 Copepod Whole Organism, Normalized *Calanus finmarchicus* cDNA clone CF_WO0_49a09; 5' similar to ref|XP_623216.2| DIVDICTED: similar to Connector of kinase to AP-1 CG7392-PA,. Score = 16 Length = 713; score = 37.9 bits (17), expect = 3.8; identities = 20/23 (86%); strand = Plus / Plus

```
Query: 24 tgcttgattcacttcwwyaagag 46
          ||||||||||||||   |||||
Sbjct: 9  tgcttgattcacttctacaagag 31
```

>EL965685|EL965685.1 CF_WO0_48f10_SP6 Copepod Whole Organism, Normalized *Calanus finmarchicus* cDNA clone CF_WO0_48f10; 5' similar to ref|XP_967858.1| DIVDICTED: similar to CG3774-PA, isoform A - Tribolium castaneum. Score Length = 771; score = 37.9 bits (17), expect = 3.8; identities = 20/23 (86%); strand = Plus / Plus

```
Query: 24 tgcttgattcacttcwwyaagag 46
          ||||||||||||||   |||||
Sbjct: 14 tgcttgattcacttctacaagag 36
```

>EL965673|EL965673.1 CF_WO0_48e09_SP6 Copepod Whole Organism, Normalized *Calanus finmarchicus* cDNA clone CF_WO0_48e09; 5', mRNA sequence. Length = 727; score = 37.9 bits (17), expect = 3.8; identities = 20/23 (86%); strand = Plus / Plus

```
Query: 24 tgcttgattcacttcwwyaagag 46
          ||||||||||||||   |||||
Sbjct: 7  tgcttgattcacttctacaagag 29
```

>EL965655|EL965655.1 CF_WO0_48d03_SP6 Copepod Whole Organism, Normalized *Calanus finmarchicus* cDNA clone CF_WO0_48d03; 5', mRNA sequence. Length = 731; score = 37.9 bits (17), expect = 3.8; identities = 20/23 (86%); strand = Plus / Plus

```
Query: 24 tgcttgattcacttcwwyaagag 46
          ||||||||||||||   |||||
Sbjct: 10 tgcttgattcacttctacaagag 32
```

>EL965613|EL965613.1 CF_WO0_47h08_SP6 Copepod Whole Organism, Normalized *Calanus finmarchicus* cDNA clone CF_WO0_47h08; 5' similar to ref|XP_001377063.1| DIVDICTED: similar to Histidyl-tRNA synthetase-like [Monodelphis. Sco Length = 676; score = 37.9 bits (17), expect = 3.8; identities = 20/23 (86%); strand = Plus / Plus

```
Query: 24 tgcttgattcacttcwwyaagag 46
          ||||||||||||||   |||||
Sbjct: 8  tgcttgattcacttctacaagag 30
```

>EL965572|EL965572.1 CF_WO0_47e01_SP6 Copepod Whole Organism, Normalized *Calanus finmarchicus* cDNA clone CF_WO0_47e01; 5' similar to ref|XP_692500.2| DIVDICTED: hypothetical protein - Danio rerio. Score = 131 bits (330), E. Length = 671; score = 37.9 bits (17), expect = 3.8; identities = 20/23 (86%); strand = Plus / Plus

```
Query: 24 tgcttgattcacttcwwyaagag 46
          ||||||||||||||   |||||
Sbjct: 14 tgcttgattcacttctacaagag 36
```

>EL965467|EL965467.1 CF_WO0_46d03_SP6 Copepod Whole Organism, Normalized *Calanus finmarchicus* cDNA clone CF_WO0_46d03; 5' similar to ref|NP_001026523.1| pyruvate dehydrogenase kinase, isoenzyme 1 - Gallus gallus. Score = 5 Length = 683; score = 37.9 bits (17), expect = 3.8; identities = 20/23 (86%); strand = Plus / Plus

```
Query: 24 tgcttgattcacttcwwyaagag 46
          ||||||||||||||   |||||
Sbjct: 18 tgcttgattcacttctacaagag 40
```

>EL965388|EL965388.1 CF_WO0_45e05_SP6 Copepod Whole Organism, Normalized *Calanus finmarchicus* cDNA clone CF_WO0_45e05; 5' similar to ref|XP_972002.1| DIVDICTED: similar to CG6006-PA - Tribolium castaneum. Score = 93.6 bits Length = 746; score = 37.9 bits (17), expect = 3.8; identities = 20/23 (86%); strand = Plus / Plus

```
Query: 24 tgcttgattcacttcwwyaagag 46
          ||||||||||||||   |||||
Sbjct: 2  tgcttgattcacttctacaagag 24
```

>EL965356|EL965356.1 CF_WO0_45b09_SP6 Copepod Whole Organism, Normalized *Calanus finmarchicus* cDNA clone CF_WO0_45b09; 5' similar to ref|XP_788859.1| DIVDICTED: similar to nuclear pore-targeting complex component of. Score Length = 711; score = 37.9 bits (17), expect = 3.8; identities = 20/23 (86%); strand = Plus / Plus

```
Query: 24 tgcttgattcacttcwwyaagag 46
          ||||||||||||||   |||||
Sbjct: 14 tgcttgattcacttctacaagag 36
```

>EL774175|EL774175.1 CF_WO0_44c06_SP6 Copepod Whole Organism, Normalized *Calanus finmarchicus* cDNA clone CF_WO0_44c06; 5' similar to ref|XP_972366.1| DIVDICTED: similar to CG9171-PA, isoform A - Tribolium castaneum. Score Length = 699; score = 37.9 bits (17), expect = 3.8; identities = 20/23 (86%); strand = Plus / Plus

```
Query: 24 tgcttgattcacttcwwyaagag 46
          ||||||||||||||   |||||
Sbjct: 2  tgcttgattcacttctacaagag 24
```

>EL774158|EL774158.1 CF_WO0_44b01_SP6 Copepod Whole Organism, Normalized *Calanus finmarchicus* cDNA clone CF_WO0_44b01; 5' , mRNA sequence. Length = 688; score = 37.9 bits (17), expect = 3.8; identities = 20/23 (86%); strand = Plus / Plus

```
Query: 24 tgcttgattcacttcwwyaagag 46
          ||||||||||||||   |||||
Sbjct: 10 tgcttgattcacttctacaagag 32
```

>EL774107|EL774107.1 CF_WO0_43e10_SP6 Copepod Whole Organism, Normalized *Calanus finmarchicus* cDNA clone CF_WO0_43e10; 5' similar to ref|XP_395624.3| DIVDICTED: similar to Broad-complex core-protein isoform 6 [Apis. Score Length = 676; score = 37.9 bits (17), expect = 3.8; identities = 20/23 (86%); strand = Plus / Plus

```
Query: 24 tgcttgattcacttcwwyaagag 46
          ||||||||||||||   |||||
Sbjct: 9  tgcttgattcacttctacaagag 31
```

>EL774099|EL774099.1 CF_WO0_43e02_SP6 Copepod Whole Organism, Normalized *Calanus finmarchicus* cDNA clone CF_WO0_43e02; 5' , mRNA sequence. Length = 717; score = 37.9 bits (17), expect = 3.8; identities = 20/23 (86%); strand = Plus / Plus

```
Query: 24 tgcttgattcacttcwwyaagag 46
          ||||||||||||||   |||||
Sbjct: 16 tgcttgattcacttctacaagag 38
```

>EL774053|EL774053.1 CF_WO0_43a03_SP6 Copepod Whole Organism, Normalized *Calanus finmarchicus* cDNA clone CF_WO0_43a03; 5' , mRNA sequence. Length = 683; score = 37.9 bits (17), expect = 3.8; identities = 20/23 (86%); strand = Plus / Plus

```
Query: 24 tgcttgattcacttcwwyaagag 46
          ||||||||||||||   |||||
Sbjct: 11 tgcttgattcacttctacaagag 33
```

>EL773987|EL773987.1 CF_WO0_42c08_SP6 Copepod Whole Organism, Normalized *Calanus finmarchicus* cDNA clone CF_WO0_42c08; 5' , mRNA sequence. Length = 652; score = 37.9 bits (17), expect = 3.8; identities = 20/23 (86%); strand = Plus / Plus

```
Query: 24 tgcttgattcacttcwwyaagag 46
          ||||||||||||||   |||||
Sbjct: 2  tgcttgattcacttctacaagag 24
```

>EL773979|EL773979.1 CF_WO0_42b12_SP6 Copepod Whole Organism, Normalized *Calanus finmarchicus* cDNA clone CF_WO0_42b12; 5' similar to gb|AAX45785.1| glucose-6-phosphate dehydrogenase isoform B - Ips typographus. Score = 189 Length = 703; score = 37.9 bits (17), expect = 3.8; identities = 20/23 (86%); strand = Plus / Plus

```
Query: 24 tgcttgattcacttcwwyaagag 46
          ||||||||||||||   |||||
Sbjct: 16 tgcttgattcacttctacaagag 38
```

>EL773921|EL773921.1 CF_WO0_41f01_SP6 Copepod Whole Organism, Normalized *Calanus finmarchicus* cDNA clone CF_WO0_41f01; 5' similar to ref|XP_966465.1| DIVDICTED: similar to CG13391-PA, isoform A - Tribolium castaneum. Score Length = 678; score = 37.9 bits (17), expect = 3.8; identities = 20/23 (86%); strand = Plus / Plus

```
Query: 23 ttgcttgattcacttcwwyaaga 45
          |||||||||||||||   ||||
```

Sbjct: 10 ttgcttgattcacttctacaaga 32

>EL773897|EL773897.1 CF_WO0_41c11_SP6 Copepod Whole Organism, Normalized *Calanus finmarchicus* cDNA clone CF_WO0_41c11; 5’ similar to ref|XP_794940.2| DIVDICTED: similar to smooth muscle myosin heavy chain 11 isoform. Score Length = 664; score = 37.9 bits (17), expect = 3.8; identities = 20/23 (86%); strand = Plus / Plus

```
Query: 24 tgcttgattcacttcwwyaagag 46
          |||||||||||||||   |||||
Sbjct: 15 tgcttgattcacttctacaagag 37
```

>EL773667|EL773667.1 CF_WO0_38h03_SP6 Copepod Whole Organism, Normalized *Calanus finmarchicus* cDNA clone CF_WO0_38h03; 5’ similar to ref|XP_966892.1| DIVDICTED: similar to CG9009-PA - Tribolium castaneum. Score = 118 bits Length = 647; score = 37.9 bits (17), expect = 3.8; identities = 20/23 (86%); strand = Plus / Plus

```
Query: 24 tgcttgattcacttcwwyaagag 46
          |||||||||||||||   |||||
Sbjct: 10 tgcttgattcacttctacaagag 32
```

>EL773657|EL773657.1 CF_WO0_38g05_SP6 Copepod Whole Organism, Normalized *Calanus finmarchicus* cDNA clone CF_WO0_38g05; 5’ similar to sp|P31401|VATB_MANSE Vacuolar ATP synthase subunit B (V-ATPase B subunit) (Vacuolar. Scor Length = 685; score = 37.9 bits (17), expect = 3.8; identities = 20/23 (86%); strand = Plus / Plus

```
Query: 24 tgcttgattcacttcwwyaagag 46
          |||||||||||||||   |||||
Sbjct: 2  tgcttgattcacttctacaagag 24
```

>EL773656|EL773656.1 CF_WO0_38g04_SP6 Copepod Whole Organism, Normalized *Calanus finmarchicus* cDNA clone CF_WO0_38g04; 5’ , mRNA sequence. Length = 749; score = 37.9 bits (17), expect = 3.8; identities = 20/23 (86%); strand = Plus / Plus

```
Query: 24 tgcttgattcacttcwwyaagag 46
          |||||||||||||||   |||||
Sbjct: 13 tgcttgattcacttctacaagag 35
```

>EL773655|EL773655.1 CF_WO0_38g03_SP6 Copepod Whole Organism, Normalized *Calanus finmarchicus* cDNA clone CF_WO0_38g03; 5’ , mRNA sequence. Length = 686; score = 37.9 bits (17), expect = 3.8; identities = 20/23 (86%); strand = Plus / Plus

```
Query: 24 tgcttgattcacttcwwyaagag 46
          |||||||||||||||   |||||
Sbjct: 7  tgcttgattcacttctacaagag 29
```

>EL773651|EL773651.1 CF_WO0_38f11_SP6 Copepod Whole Organism, Normalized *Calanus finmarchicus* cDNA clone CF_WO0_38f11; 5’ similar to ref|XP_966693.1| DIVDICTED: similar to CG17332-PA, isoform A isoform 1 [Tribolium. Score Length = 747; score = 37.9 bits (17), expect = 3.8; identities = 20/23 (86%); strand = Plus / Plus

```
Query: 24 tgcttgattcacttcwwyaagag 46
          ||||||||||||||   |||||
Sbjct: 2  tgcttgattcacttctacaagag 24
```

>EL773600|EL773600.1 CF_WO0_38b08_SP6 Copepod Whole Organism, Normalized *Calanus finmarchicus* cDNA clone CF_WO0_38b08; 5' similar to ref|NP_610242.1| coro CG9446-PA, isoform A - Drosophila melanogaster. Score = 310 bits (7 Length = 703; score = 37.9 bits (17), expect = 3.8; identities = 20/23 (86%); strand = Plus / Plus

```
Query: 24 tgcttgattcacttcwwyaagag 46
          ||||||||||||||   |||||
Sbjct: 2  tgcttgattcacttctacaagag 24
```

>EL773538|EL773538.1 CF_WO0_37e05_SP6 Copepod Whole Organism, Normalized *Calanus finmarchicus* cDNA clone CF_WO0_37e05; 5' similar to emb|CAE60022.1| Hypothetical protein CBG03527 - Caenorhabditis briggsae. Score = 115 bits Length = 723; score = 37.9 bits (17), expect = 3.8; identities = 20/23 (86%); strand = Plus / Plus

```
Query: 24 tgcttgattcacttcwwyaagag 46
          ||||||||||||||   |||||
Sbjct: 1  tgcttgattcacttctacaagag 23
```

>EL773322|EL773322.1 CF_WO0_35c02_SP6 Copepod Whole Organism, Normalized *Calanus finmarchicus* cDNA clone CF_WO0_35c02; 5' similar to ref|XP_975072.1| DIVDICTED: similar to CG8594-PA - Tribolium castaneum. Score = 181 bits Length = 601; score = 37.9 bits (17), expect = 3.8; identities = 20/23 (86%); strand = Plus / Plus

```
Query: 24 tgcttgattcacttcwwyaagag 46
          ||||||||||||||   |||||
Sbjct: 9  tgcttgattcacttctacaagag 31
```

>EL773302|EL773302.1 CF_WO0_35a06_SP6 Copepod Whole Organism, Normalized *Calanus finmarchicus* cDNA clone CF_WO0_35a06; 5' similar to gb|EAT37056.1| conserved hypothetical protein - Aedes aegypti. Score = 62.4 bits (150), E. Length = 640; score = 37.9 bits (17), expect = 3.8; identities = 20/23 (86%); strand = Plus / Plus

```
Query: 24 tgcttgattcacttcwwyaagag 46
          ||||||||||||||   |||||
Sbjct: 9  tgcttgattcacttctacaagag 31
```

>EL697518|EL697518.1 CF_WO0_34g12_SP6 Copepod Whole Organism, Normalized *Calanus finmarchicus* cDNA clone CF_WO0_34g12; 5' similar to ref|NP_998293.1| eukaryotic translation initiation factor 3, subunit 6 interacting. Score Length = 660; score = 37.9 bits (17), expect = 3.8; identities = 20/23 (86%); strand = Plus / Plus

```
Query: 24 tgcttgattcacttcwwyaagag 46
          ||||||||||||||   |||||
Sbjct: 1  tgcttgattcacttctacaagag 23
```

>EL697503|EL697503.1 CF_WO0_34f08_SP6 Copepod Whole Organism, Normalized *Calanus finmarchicus* cDNA clone CF_WO0_34f08; 5' similar to ref|XP_971407.1| DIVDICTED: similar to CG8402-PA, isoform A - Tribolium castaneum. Score Length = 715; score = 37.9 bits (17), expect = 3.8; identities = 20/23 (86%); strand = Plus / Plus

```
Query: 24 tgcttgattcacttcwwyaagag 46
          ||||||||||||||   |||||
Sbjct: 10 tgcttgattcacttctacaagag 32
```

>EL697498|EL697498.1 CF_WO0_34f03_SP6 Copepod Whole Organism, Normalized *Calanus finmarchicus* cDNA clone CF_WO0_34f03; 5' , mRNA sequence. Length = 726; score = 37.9 bits (17), expect = 3.8; identities = 20/23 (86%); strand = Plus / Plus

```
Query: 24 tgcttgattcacttcwwyaagag 46
          ||||||||||||||   |||||
Sbjct: 2  tgcttgattcacttctacaagag 24
```

>EL697422|EL697422.1 CF_WO0_33g10_SP6 Copepod Whole Organism, Normalized *Calanus finmarchicus* cDNA clone CF_WO0_33g10; 5' , mRNA sequence. Length = 664; score = 37.9 bits (17), expect = 3.8; identities = 20/23 (86%); strand = Plus / Plus

```
Query: 24 tgcttgattcacttcwwyaagag 46
          ||||||||||||||   |||||
Sbjct: 2  tgcttgattcacttctacaagag 24
```

>EL697418|EL697418.1 CF_WO0_33g06_SP6 Copepod Whole Organism, Normalized *Calanus finmarchicus* cDNA clone CF_WO0_33g06; 5' similar to gb|AAY81965.1| importin beta 1 - Aplysia californica. Score = 241 bits (616), expect = 2e Length = 696; score = 37.9 bits (17), expect = 3.8; identities = 20/23 (86%); strand = Plus / Plus

```
Query: 24 tgcttgattcacttcwwyaagag 46
          ||||||||||||||   |||||
Sbjct: 10 tgcttgattcacttctacaagag 32
```

>EL697390|EL697390.1 CF_WO0_33e02_SP6 Copepod Whole Organism, Normalized *Calanus finmarchicus* cDNA clone CF_WO0_33e02; 5' similar to gb|EAT42014.1| ubiquitin-conjugating enzyme morgue - Aedes aegypti. Score = 118 bits (295 Length = 749; score = 37.9 bits (17), expect = 3.8; identities = 20/23 (86%); strand = Plus / Plus

```
Query: 24 tgcttgattcacttcwwyaagag 46
          ||||||||||||||   |||||
Sbjct: 14 tgcttgattcacttctacaagag 36
```

>EL697380|EL697380.1 CF_WO0_33d04_SP6 Copepod Whole Organism, Normalized *Calanus finmarchicus* cDNA clone CF_WO0_33d04; 5' similar to ref|XP_422434.2| DIVDICTED: hypothetical protein - Gallus gallus. Score = 219 bits (557), Length = 675; score = 37.9 bits (17), expect = 3.8; identities = 20/23 (86%); strand = Plus / Plus

Query: 24 tgcttgattcacttcwwyaagag 46
          |||||||||||||| |||||
Sbjct: 2 tgcttgattcacttctacaagag 24

>EL697373|EL697373.1 CF_W00_33c09_SP6 Copepod Whole Organism, Normalized *Calanus finmarchicus* cDNA clone CF_W00_33c09; 5' similar to ref|XP_226262.4| DIVDICTED: similar to CG31120-PA, isoform A - Rattus norvegicus. Score = Length = 598; score = 37.9 bits (17), expect = 3.8; identities = 20/23 (86%); strand = Plus / Plus

Query: 24 tgcttgattcacttcwwyaagag 46
          |||||||||||||| |||||
Sbjct: 2 tgcttgattcacttctacaagag 24

>EL697275|EL697275.1 CF_W00_32c07_SP6 Copepod Whole Organism, Normalized *Calanus finmarchicus* cDNA clone CF_W00_32c07; 5' , mRNA sequence. Length = 418; score = 37.9 bits (17), expect = 3.8; identities = 20/23 (86%); strand = Plus / Plus

Query: 24 tgcttgattcacttcwwyaagag 46
          |||||||||||||| |||||
Sbjct: 11 tgcttgattcacttctacaagag 33

>EL697238|EL697238.1 CF_W00_31h04_SP6 Copepod Whole Organism, Normalized *Calanus finmarchicus* cDNA clone CF_W00_31h04; 5' similar to dbj|BAF02549.1| amino acid transporter - Crassostrea gigas. Score = 200 bits (508), Expec Length = 676; score = 37.9 bits (17), expect = 3.8; identities = 20/23 (86%); strand = Plus / Plus

Query: 24 tgcttgattcacttcwwyaagag 46
          |||||||||||||| |||||
Sbjct: 2 tgcttgattcacttctacaagag 24

>EL697217|EL697217.1 CF_W00_31f06_SP6 Copepod Whole Organism, Normalized *Calanus finmarchicus* cDNA clone CF_W00_31f06; 5' similar to ref|XP_001193936.1| DIVDICTED: similar to WD repeat domain 51B [Strongylocentrotus. Score Length = 671; score = 37.9 bits (17), expect = 3.8; identities = 20/23 (86%); strand = Plus / Plus

Query: 24 tgcttgattcacttcwwyaagag 46
          |||||||||||||| |||||
Sbjct: 11 tgcttgattcacttctacaagag 33

>EL697208|EL697208.1 CF_W00_31e09_SP6 Copepod Whole Organism, Normalized *Calanus finmarchicus* cDNA clone CF_W00_31e09; 5' , mRNA sequence. Length = 659; score = 37.9 bits (17), expect = 3.8; identities = 20/23 (86%); strand = Plus / Plus

Query: 24 tgcttgattcacttcwwyaagag 46
          |||||||||||||| |||||
Sbjct: 2 tgcttgattcacttctacaagag 24

>EL697178|EL697178.1 CF_W00_31c03_SP6 Copepod Whole Organism, Normalized *Calanus finmarchicus* cDNA clone CF_W00_31c03; 5' similar to ref|XP_001193936.1| DIVDICTED: similar to WD repeat

domain 51B [Strongylocentrotus. Score Length = 659; score = 37.9 bits (17), expect = 3.8; identities = 20/23 (86%); strand = Plus / Plus

```
Query: 24 tgcttgattcacttcwwyaagag 46
          ||||||||||||||   |||||
Sbjct: 11 tgcttgattcacttctacaagag 33
```

>EL697123|EL697123.1 CF_WO0_30f08_SP6 Copepod Whole Organism, Normalized *Calanus finmarchicus* cDNA clone CF_WO0_30f08; 5' similar to sp|Q8SPZ3|P53_DELLE Cellular tumor antigen p53 (Tumor suppressor p53). Score = 58.5 bits Length = 712; score = 37.9 bits (17), expect = 3.8; identities = 20/23 (86%); strand = Plus / Plus

```
Query: 24 tgcttgattcacttcwwyaagag 46
          ||||||||||||||   |||||
Sbjct: 2  tgcttgattcacttctacaagag 24
```

>EL697114|EL697114.1 CF_WO0_30e11_SP6 Copepod Whole Organism, Normalized *Calanus finmarchicus* cDNA clone CF_WO0_30e11; 5' , mRNA sequence. Length = 692; score = 37.9 bits (17), expect = 3.8; identities = 20/23 (86%); strand = Plus / Plus

```
Query: 24 tgcttgattcacttcwwyaagag 46
          ||||||||||||||   |||||
Sbjct: 8  tgcttgattcacttctacaagag 30
```

>EL697094|EL697094.1 CF_WO0_30d03_SP6 Copepod Whole Organism, Normalized *Calanus finmarchicus* cDNA clone CF_WO0_30d03; 5' similar to ref|NP_524429.1| tetracycline resistance CG5760-PA - Drosophila melanogaster. Score = 161 Length = 682; score = 37.9 bits (17), expect = 3.8; identities = 20/23 (86%); strand = Plus / Plus

```
Query: 24 tgcttgattcacttcwwyaagag 46
          ||||||||||||||   |||||
Sbjct: 15 tgcttgattcacttctacaagag 37
```

>EL697047|EL697047.1 CF_WO0_29h04_SP6 Copepod Whole Organism, Normalized *Calanus finmarchicus* cDNA clone CF_WO0_29h04; 5' similar to ref|XP_001122626.1| DIVDICTED: similar to roundabout CG13521-PA, isoform A, partial. Scor Length = 710; score = 37.9 bits (17), expect = 3.8; identities = 20/23 (86%); strand = Plus / Plus

```
Query: 24 tgcttgattcacttcwwyaagag 46
          ||||||||||||||   |||||
Sbjct: 2  tgcttgattcacttctacaagag 24
```

>EL696942|EL696942.1 CF_WO0_28g03_SP6 Copepod Whole Organism, Normalized *Calanus finmarchicus* cDNA clone CF_WO0_28g03; 5' , mRNA sequence. Length = 671; score = 37.9 bits (17), expect = 3.8; identities = 20/23 (86%); strand = Plus / Plus

```
Query: 24 tgcttgattcacttcwwyaagag 46
          ||||||||||||||   |||||
Sbjct: 12 tgcttgattcacttctacaagag 34
```

>EL696906|EL696906.1 CF_WO0_28d03_SP6 Copepod Whole Organism, Normalized *Calanus finmarchicus* cDNA clone CF_WO0_28d03; 5’ similar to ref|XP_969316.1| DIVDICTED: similar to CG9181-PA, isoform A - Tribolium castaneum. Score Length = 615; score = 37.9 bits (17), expect = 3.8; identities = 20/23 (86%); strand = Plus / Plus

```
Query: 24 tgcttgattcacttcwwyaagag 46
          |||||||||||||||   |||||
Sbjct: 9  tgcttgattcacttctacaagag 31
```

>EL696901|EL696901.1 CF_WO0_28c10_SP6 Copepod Whole Organism, Normalized *Calanus finmarchicus* cDNA clone CF_WO0_28c10; 5’, mRNA sequence. Length = 668; score = 37.9 bits (17), expect = 3.8; identities = 20/23 (86%); strand = Plus / Plus

```
Query: 24 tgcttgattcacttcwwyaagag 46
          |||||||||||||||   |||||
Sbjct: 12 tgcttgattcacttctacaagag 34
```

>EL696898|EL696898.1 CF_WO0_28c06_SP6 Copepod Whole Organism, Normalized *Calanus finmarchicus* cDNA clone CF_WO0_28c06; 5’ similar to gb|EAT39488.1| conserved hypothetical protein - Aedes aegypti. Score = 171 bits (432), Ex Length = 735; score = 37.9 bits (17), expect = 3.8; identities = 20/23 (86%); strand = Plus / Plus

```
Query: 24 tgcttgattcacttcwwyaagag 46
          |||||||||||||||   |||||
Sbjct: 1  tgcttgattcacttctacaagag 23
```

>EL696864|EL696864.1 CF_WO0_27h07_SP6 Copepod Whole Organism, Normalized *Calanus finmarchicus* cDNA clone CF_WO0_27h07; 5’ similar to gb|EAT48688.1| heat shock protein - Aedes aegypti. Score = 166 bits (420), expect = 8e-40 Length = 628; score = 37.9 bits (17), expect = 3.8; identities = 20/23 (86%); strand = Plus / Plus

```
Query: 24 tgcttgattcacttcwwyaagag 46
          |||||||||||||||   |||||
Sbjct: 2  tgcttgattcacttctacaagag 24
```

>EL696788|EL696788.1 CF_WO0_27b03_SP6 Copepod Whole Organism, Normalized *Calanus finmarchicus* cDNA clone CF_WO0_27b03; 5’ similar to ref|XP_313758.3| ENSANGP00000017072 - Anopheles gambiae str. PEST. Score = 194 bits (493) Length = 682; score = 37.9 bits (17), expect = 3.8; identities = 20/23 (86%); strand = Plus / Plus

```
Query: 24 tgcttgattcacttcwwyaagag 46
          |||||||||||||||   |||||
Sbjct: 2  tgcttgattcacttctacaagag 24
```

>EL696725|EL696725.1 CF_WO0_26d11_SP6 Copepod Whole Organism, Normalized *Calanus finmarchicus* cDNA clone CF_WO0_26d11; 5’ similar to ref|XP_624580.1| DIVDICTED: similar to Chromatin assembly factor 1 subunit CG4236-PA. Sco Length = 668; score = 37.9 bits (17), expect = 3.8; identities = 20/23 (86%); strand = Plus / Plus

Query: 24 tgcttgattcacttcwwyaagag 46
||||||||||||||| |||||
Sbjct: 4 tgcttgattcacttctacaagag 26

>EL696719|EL696719.1 CF_W00_26d05_SP6 Copepod Whole Organism, Normalized *Calanus finmarchicus* cDNA clone CF_W00_26d05; 5' , mRNA sequence. Length = 765; score = 37.9 bits (17), expect = 3.8; identities = 20/23 (86%); strand = Plus / Plus

Query: 24 tgcttgattcacttcwwyaagag 46
||||||||||||||| |||||
Sbjct: 9 tgcttgattcacttctacaagag 31

>EL696670|EL696670.1 CF_W00_25h02_SP6 Copepod Whole Organism, Normalized *Calanus finmarchicus* cDNA clone CF_W00_25h02; 5' similar to ref|XP_393723.2| DIVDICTED: similar to Rab-protein 10 CG17060-PA - Apis mellifera. Score Length = 705; score = 37.9 bits (17), expect = 3.8; identities = 20/23 (86%); strand = Plus / Plus

Query: 24 tgcttgattcacttcwwyaagag 46
||||||||||||||| |||||
Sbjct: 13 tgcttgattcacttctacaagag 35

>EL696662|EL696662.1 CF_W00_25g06_SP6 Copepod Whole Organism, Normalized *Calanus finmarchicus* cDNA clone CF_W00_25g06; 5' similar to gb|EAT37426.1| zinc finger protein, putative - Aedes aegypti. Score = 179 bits (453), Exp Length = 750; score = 37.9 bits (17), expect = 3.8; identities = 20/23 (86%); strand = Plus / Plus

Query: 24 tgcttgattcacttcwwyaagag 46
||||||||||||||| |||||
Sbjct: 11 tgcttgattcacttctacaagag 33

>EL586660|EL586660.1 CF_W00_24g11_SP6 Copepod Whole Organism, Normalized *Calanus finmarchicus* cDNA clone CF_W00_24g11; 5' similar to XP_967453.1 pyruvate dehydrogenase phosphatase regulatory subunit [Tribolium castaneum]. Length = 657; score = 37.9 bits (17), expect = 3.8; identities = 20/23 (86%); strand = Plus / Plus

Query: 24 tgcttgattcacttcwwyaagag 46
||||||||||||||| |||||
Sbjct: 2 tgcttgattcacttctacaagag 24

>EL586655|EL586655.1 CF_W00_24g06_SP6 Copepod Whole Organism, Normalized *Calanus finmarchicus* cDNA clone CF_W00_24g06; 5' , mRNA sequence. Length = 668; score = 37.9 bits (17), expect = 3.8; identities = 20/23 (86%); strand = Plus / Plus

Query: 24 tgcttgattcacttcwwyaagag 46
||||||||||||||| |||||
Sbjct: 2 tgcttgattcacttctacaagag 24

>EL586653|EL586653.1 CF_WO0_24g04_SP6 Copepod Whole Organism, Normalized *Calanus finmarchicus* cDNA clone CF_WO0_24g04; 5' similar to NP_001006823.1 solute carrier family 30 (zinc transporter), member 3 [Xenopus tropicalis] Length = 713; score = 37.9 bits (17), expect = 3.8; identities = 20/23 (86%); strand = Plus / Plus

```
Query: 24 tgcttgattcacttcwwyaagag 46
          |||||||||||||||   |||||
Sbjct: 2  tgcttgattcacttctacaagag 24
```

>EL586643|EL586643.1 CF_WO0_24f04_SP6 Copepod Whole Organism, Normalized *Calanus finmarchicus* cDNA clone CF_WO0_24f04; 5' similar to XP_967950.1 Vacuolar ATP synthase catalytic subunit A, osteoclast isoform (V-ATPase A sub Length = 697; score = 37.9 bits (17), expect = 3.8; identities = 20/23 (86%); strand = Plus / Plus

```
Query: 24 tgcttgattcacttcwwyaagag 46
          |||||||||||||||   |||||
Sbjct: 11 tgcttgattcacttctacaagag 33
```

>EL586638|EL586638.1 CF_WO0_24e11_SP6 Copepod Whole Organism, Normalized *Calanus finmarchicus* cDNA clone CF_WO0_24e11; 5' similar to Q6PA48 Protein TBRG4 (Transforming growth factor beta regulator 4). Score=78.57, Expect=2 Length = 711; score = 37.9 bits (17), expect = 3.8; identities = 20/23 (86%); strand = Plus / Plus

```
Query: 24 tgcttgattcacttcwwyaagag 46
          |||||||||||||||   |||||
Sbjct: 11 tgcttgattcacttctacaagag 33
```

>EL586605|EL586605.1 CF_WO0_24b10_SP6 Copepod Whole Organism, Normalized *Calanus finmarchicus* cDNA clone CF_WO0_24b10; 5' similar to XP_783924.2 hypothetical protein [Strongylocentrotus purpuratus]. Score=65.08, Expect=6.6 Length = 415; score = 37.9 bits (17), expect = 3.8; identities = 20/23 (86%); strand = Plus / Plus

```
Query: 24 tgcttgattcacttcwwyaagag 46
          |||||||||||||||   |||||
Sbjct: 13 tgcttgattcacttctacaagag 35
```

>EL586452|EL586452.1 CF_WO0_22e08_SP6 Copepod Whole Organism, Normalized *Calanus finmarchicus* cDNA clone CF_WO0_22e08; 5' similar to XP_624146.2 crooked neck CG3193-PA [Apis mellifera]. Score=143.67, Expect=1.50E-33, mRNA Length = 376; score = 37.9 bits (17), expect = 3.8; identities = 20/23 (86%); strand = Plus / Plus

```
Query: 24 tgcttgattcacttcwwyaagag 46
          |||||||||||||||   |||||
Sbjct: 2  tgcttgattcacttctacaagag 24
```

>EL586404|EL586404.1 CF_WO0_22a07_SP6 Copepod Whole Organism, Normalized *Calanus finmarchicus* cDNA clone CF_WO0_22a07; 5', mRNA sequence. Length = 724; score = 37.9 bits (17), expect = 3.8; identities = 20/23 (86%); strand = Plus / Plus

Query: 24 tgcttgattcacttcwwyaagag 46
          |||||||||||||| |||||
Sbjct: 10 tgcttgattcacttctacaagag 32

>EL586402|EL586402.1 CF_W00_22a05_SP6 Copepod Whole Organism, Normalized *Calanus finmarchicus* cDNA clone CF_W00_22a05; 5', mRNA sequence. Length = 653; score = 37.9 bits (17), expect = 3.8; identities = 20/23 (86%); strand = Plus / Plus

Query: 24 tgcttgattcacttcwwyaagag 46
          |||||||||||||| |||||
Sbjct: 2 tgcttgattcacttctacaagag 24

>EL586370|EL586370.1 CF_W00_21f09_SP6 Copepod Whole Organism, Normalized *Calanus finmarchicus* cDNA clone CF_W00_21f09; 5' similar to NP_524099.2 DNA-polymerase-delta CG5949-PA [Drosophila melanogaster]. Score=188.35, Expec Length = 687; score = 37.9 bits (17), expect = 3.8; identities = 20/23 (86%); strand = Plus / Plus

Query: 24 tgcttgattcacttcwwyaagag 46
          |||||||||||||| |||||
Sbjct: 10 tgcttgattcacttctacaagag 32

>EL586283|EL586283.1 CF_W00_20g04_SP6 Copepod Whole Organism, Normalized *Calanus finmarchicus* cDNA clone CF_W00_20g04; 5' similar to XP_418921.1 thiopurine methyltransferase [Gallus gallus]. Score=117.86, Expect=3.20E-25, Length = 666; score = 37.9 bits (17), expect = 3.8; identities = 20/23 (86%); strand = Plus / Plus

Query: 24 tgcttgattcacttcwwyaagag 46
          |||||||||||||| |||||
Sbjct: 2 tgcttgattcacttctacaagag 24

>EL586269|EL586269.1 CF_W00_20f02_SP6 Copepod Whole Organism, Normalized *Calanus finmarchicus* cDNA clone CF_W00_20f02; 5' similar to NP_998293.1 eukaryotic translation initiation factor 3, subunit 6 interacting protein [Da Length = 681; score = 37.9 bits (17), expect = 3.8; identities = 20/23 (86%); strand = Plus / Plus

Query: 24 tgcttgattcacttcwwyaagag 46
          |||||||||||||| |||||
Sbjct: 2 tgcttgattcacttctacaagag 24

>EL586254|EL586254.1 CF_W00_20d10_SP6 Copepod Whole Organism, Normalized *Calanus finmarchicus* cDNA clone CF_W00_20d10; 5', mRNA sequence. Length = 709; score = 37.9 bits (17), expect = 3.8; identities = 20/23 (86%); strand = Plus / Plus

Query: 24 tgcttgattcacttcwwyaagag 46
          |||||||||||||| |||||
Sbjct: 2 tgcttgattcacttctacaagag 24

>EL586229|EL586229.1 CF_W00_20b09_SP6 Copepod Whole Organism, Normalized *Calanus finmarchicus* cDNA clone CF_W00_20b09; 5' similar to AAH76828.1 Ercc4-prov protein [Xenopus laevis].

Score=273.86, Expect=3.90E-72, mRNA seque Length = 706; score = 37.9 bits (17), expect = 3.8; identities = 20/23 (86%); strand = Plus / Plus

```
Query: 24 tgcttgattcacttcwwyaagag 46
          |||||||||||||||   |||||
Sbjct: 15 tgcttgattcacttctacaagag 37
```

>EL586225|EL586225.1 CF_W00_20b05_SP6 Copepod Whole Organism, Normalized *Calanus finmarchicus* cDNA clone CF_W00_20b05; 5' similar to XP_792687.2 LOC495188 protein [Strongylocentrotus purpuratus]. Score=87.81, Expect=3.80E- Length = 687; score = 37.9 bits (17), expect = 3.8; identities = 20/23 (86%); strand = Plus / Plus

```
Query: 24 tgcttgattcacttcwwyaagag 46
          |||||||||||||||   |||||
Sbjct: 2  tgcttgattcacttctacaagag 24
```

>EL586208|EL586208.1 CF_W00_19h10_SP6 Copepod Whole Organism, Normalized *Calanus finmarchicus* cDNA clone CF_W00_19h10; 5' , mRNA sequence. Length = 687; score = 37.9 bits (17), expect = 3.8; identities = 20/23 (86%); strand = Plus / Plus

```
Query: 24 tgcttgattcacttcwwyaagag 46
          |||||||||||||||   |||||
Sbjct: 13 tgcttgattcacttctacaagag 35
```

>EL586019|EL586019.1 CF_W00_17g11_SP6 Copepod Whole Organism, Normalized *Calanus finmarchicus* cDNA clone CF_W00_17g11; 5' similar to AAM96688.1 TFIIB-related Factor [Bombyx mori]. Score=223.4, Expect=4.60E-57, mRNA sequenc Length = 618; score = 37.9 bits (17), expect = 3.8; identities = 20/23 (86%); strand = Plus / Plus

```
Query: 24 tgcttgattcacttcwwyaagag 46
          |||||||||||||||   |||||
Sbjct: 11 tgcttgattcacttctacaagag 33
```

>EL586018|EL586018.1 CF_W00_17g10_SP6 Copepod Whole Organism, Normalized *Calanus finmarchicus* cDNA clone CF_W00_17g10; 5' similar to NP_001017006.1 hypothetical protein LOC549760 [Xenopus tropicalis]. Score=103.6, Expect=7 Length = 714; score = 37.9 bits (17), expect = 3.8; identities = 20/23 (86%); strand = Plus / Plus

```
Query: 24 tgcttgattcacttcwwyaagag 46
          |||||||||||||||   |||||
Sbjct: 10 tgcttgattcacttctacaagag 32
```

>EL585998|EL585998.1 CF_W00_17f02_SP6 Copepod Whole Organism, Normalized *Calanus finmarchicus* cDNA clone CF_W00_17f02; 5' similar to XP_393080.3 spastin CG5977-PA, isoform A [Apis mellifera]. Score=48.14, Expect=0.000295, Length = 651; score = 37.9 bits (17), expect = 3.8; identities = 20/23 (86%); strand = Plus / Plus

```
Query: 24 tgcttgattcacttcwwyaagag 46
          |||||||||||||||   |||||
```

Sbjct: 2 tgcttgattcacttctacaagag 24

>EL585979|EL585979.1 CF_WO0_17d06_SP6 Copepod Whole Organism, Normalized *Calanus finmarchicus* cDNA clone CF_WO0_17d06; 5' similar to XP_966498.1 Vesicle-associated membrane protein-associated protein B/C (VAMP-associated p Length = 650; score = 37.9 bits (17), expect = 3.8; identities = 20/23 (86%); strand = Plus / Plus

```
Query: 24 tgcttgattcacttcwwyaagag 46
          |||||||||||||||   |||||
Sbjct: 2  tgcttgattcacttctacaagag 24
```

>EL585963|EL585963.1 CF_WO0_17c02_SP6 Copepod Whole Organism, Normalized *Calanus finmarchicus* cDNA clone CF_WO0_17c02; 5', mRNA sequence. Length = 731; score = 37.9 bits (17), expect = 3.8; identities = 20/23 (86%); strand = Plus / Plus

```
Query: 24 tgcttgattcacttcwwyaagag 46
          |||||||||||||||   |||||
Sbjct: 15 tgcttgattcacttctacaagag 37
```

>EL585934|EL585934.1 CF_WO0_16h08_SP6 Copepod Whole Organism, Normalized *Calanus finmarchicus* cDNA clone CF_WO0_16h08; 5' similar to NP_055556.2 hypothetical protein LOC9776 [Homo sapiens]. Score=147.52, Expect=3.80E-34, m Length = 673; score = 37.9 bits (17), expect = 3.8; identities = 20/23 (86%); strand = Plus / Plus

```
Query: 24 tgcttgattcacttcwwyaagag 46
          |||||||||||||||   |||||
Sbjct: 10 tgcttgattcacttctacaagag 32
```

>EL585920|EL585920.1 CF_WO0_16g06_SP6 Copepod Whole Organism, Normalized *Calanus finmarchicus* cDNA clone CF_WO0_16g06; 5' similar to NP_610562.1 CG12214-PA, isoform A [Drosophila melanogaster]. Score=58.54, Expect=1.50E-07 Length = 565; score = 37.9 bits (17), expect = 3.8; identities = 20/23 (86%); strand = Plus / Plus

```
Query: 16 gctwawwttgcttgattcacttc 38
          ||| |  ||||||||||||||||
Sbjct: 1  gctaatattgcttgattcacttc 23
```

>EL585886|EL585886.1 CF_WO0_16d05_SP6 Copepod Whole Organism, Normalized *Calanus finmarchicus* cDNA clone CF_WO0_16d05; 5', mRNA sequence. Length = 615; score = 37.9 bits (17), expect = 3.8; identities = 20/23 (86%); strand = Plus / Plus

```
Query: 24 tgcttgattcacttcwwyaagag 46
          |||||||||||||||   |||||
Sbjct: 2  tgcttgattcacttctacaagag 24
```

>EL585850|EL585850.1 CF_WO0_16a04_SP6 Copepod Whole Organism, Normalized *Calanus finmarchicus* cDNA clone CF_WO0_16a04; 5', mRNA sequence. Length = 651; score = 37.9 bits (17), expect = 3.8; identities = 20/23 (86%); strand = Plus / Plus

Query: 24 tgcttgattcacttcwwyaagag 46
          ||||||||||||||| |||||
Sbjct: 2 tgcttgattcacttctacaagag 24

>EL585815|EL585815.1 CF_WO0_15f03_SP6 Copepod Whole Organism, Normalized *Calanus finmarchicus* cDNA clone CF_WO0_15f03; 5' , mRNA sequence. Length = 679; score = 37.9 bits (17), expect = 3.8; identities = 20/23 (86%); strand = Plus / Plus

Query: 24 tgcttgattcacttcwwyaagag 46
          ||||||||||||||| |||||
Sbjct: 9 tgcttgattcacttctacaagag 31

>EL585806|EL585806.1 CF_WO0_15e06_SP6 Copepod Whole Organism, Normalized *Calanus finmarchicus* cDNA clone CF_WO0_15e06; 5' similar to XP_966417.1 protein phosphatase 1, catalytic subunit, beta isoform 1 [Tribolium castaneum Length = 617; score = 37.9 bits (17), expect = 3.8; identities = 20/23 (86%); strand = Plus / Plus

Query: 24 tgcttgattcacttcwwyaagag 46
          ||||||||||||||| |||||
Sbjct: 7 tgcttgattcacttctacaagag 29

>EL585805|EL585805.1 CF_WO0_15e05_SP6 Copepod Whole Organism, Normalized *Calanus finmarchicus* cDNA clone CF_WO0_15e05; 5' similar to XP_966417.1 protein phosphatase 1, catalytic subunit, beta isoform 1 [Tribolium castaneum Length = 668; score = 37.9 bits (17), expect = 3.8; identities = 20/23 (86%); strand = Plus / Plus

Query: 24 tgcttgattcacttcwwyaagag 46
          ||||||||||||||| |||||
Sbjct: 9 tgcttgattcacttctacaagag 31

>EL585790|EL585790.1 CF_WO0_15d02_SP6 Copepod Whole Organism, Normalized *Calanus finmarchicus* cDNA clone CF_WO0_15d02; 5' , mRNA sequence. Length = 666; score = 37.9 bits (17), expect = 3.8; identities = 20/23 (86%); strand = Plus / Plus

Query: 24 tgcttgattcacttcwwyaagag 46
          ||||||||||||||| |||||
Sbjct: 14 tgcttgattcacttctacaagag 36

>EL585783|EL585783.1 CF_WO0_15c05_SP6 Copepod Whole Organism, Normalized *Calanus finmarchicus* cDNA clone CF_WO0_15c05; 5' similar to XP_418739.2 KIAA0379 protein [Gallus gallus]. Score=267.31, Expect=3.80E-70, mRNA sequenc Length = 723; score = 37.9 bits (17), expect = 3.8; identities = 20/23 (86%); strand = Plus / Plus

Query: 24 tgcttgattcacttcwwyaagag 46
          ||||||||||||||| |||||
Sbjct: 10 tgcttgattcacttctacaagag 32

>EL585709|EL585709.1 CF_WO0_14e01_SP6 Copepod Whole Organism, Normalized *Calanus finmarchicus* cDNA clone CF_WO0_14e01; 5' similar to AAH73694.1 MGC83596 protein [Xenopus laevis].

Score=254.99, Expect=1.80E-66, mRNA sequenc Length = 685; score = 37.9 bits (17), expect = 3.8; identities = 20/23 (86%); strand = Plus / Plus

```
Query: 24 tgcttgattcacttcwwyaagag 46
          ||||||||||||||   |||||
Sbjct: 6  tgcttgattcacttctacaagag 28
```

>EL585706|EL585706.1 CF_WO0_14d10_SP6 Copepod Whole Organism, Normalized *Calanus finmarchicus* cDNA clone CF_WO0_14d10; 5' , mRNA sequence. Length = 705; score = 37.9 bits (17), expect = 3.8; identities = 20/23 (86%); strand = Plus / Plus

```
Query: 24 tgcttgattcacttcwwyaagag 46
          ||||||||||||||   |||||
Sbjct: 14 tgcttgattcacttctacaagag 36
```

>EL585692|EL585692.1 CF_WO0_14c08_SP6 Copepod Whole Organism, Normalized *Calanus finmarchicus* cDNA clone CF_WO0_14c08; 5' similar to EAL26082.1 GA20924-PA [Drosophila pseudoobscura]. Score=233.8, Expect=4.10E-60, mRNA sequ Length = 674; score = 37.9 bits (17), expect = 3.8; identities = 20/23 (86%); strand = Plus / Plus

```
Query: 24 tgcttgattcacttcwwyaagag 46
          ||||||||||||||   |||||
Sbjct: 11 tgcttgattcacttctacaagag 33
```

>EL585668|EL585668.1 CF_WO0_14a07_SP6 Copepod Whole Organism, Normalized *Calanus finmarchicus* cDNA clone CF_WO0_14a07; 5' , mRNA sequence. Length = 692; score = 37.9 bits (17), expect = 3.8; identities = 20/23 (86%); strand = Plus / Plus

```
Query: 24 tgcttgattcacttcwwyaagag 46
          ||||||||||||||   |||||
Sbjct: 10 tgcttgattcacttctacaagag 32
```

>EL585666|EL585666.1 CF_WO0_14a05_SP6 Copepod Whole Organism, Normalized *Calanus finmarchicus* cDNA clone CF_WO0_14a05; 5' similar to XP_664793.1 hypothetical protein AN7189.2 [Aspergillus nidulans FGSC A4]. Score=48.91, Ex Length = 702; score = 37.9 bits (17), expect = 3.8; identities = 20/23 (86%); strand = Plus / Plus

```
Query: 24 tgcttgattcacttcwwyaagag 46
          ||||||||||||||   |||||
Sbjct: 15 tgcttgattcacttctacaagag 37
```

>EL585609|EL585609.1 CF_WO0_13d04_SP6 Copepod Whole Organism, Normalized *Calanus finmarchicus* cDNA clone CF_WO0_13d04; 5' similar to XP_970646.1 CG7033-PA, isoform A [Tribolium castaneum]. Score=305.06, Expect=1.60E-81, mR Length = 703; score = 37.9 bits (17), expect = 3.8; identities = 20/23 (86%); strand = Plus / Plus

```
Query: 24 tgcttgattcacttcwwyaagag 46
          ||||||||||||||   |||||
Sbjct: 2  tgcttgattcacttctacaagag 24
```

>EL585584|EL585584.1 CF_WO0_13a11_SP6 Copepod Whole Organism, Normalized *Calanus finmarchicus* cDNA clone CF_WO0_13a11; 5' similar to XP_793318.2 DNA mismatch repair protein homolog [Strongylocentrotus purpuratus]. Score=26 Length = 637; score = 37.9 bits (17), expect = 3.8; identities = 20/23 (86%); strand = Plus / Plus

```
Query: 24 tgcttgattcacttcwwyaagag 46
          |||||||||||||||   |||||
Sbjct: 11 tgcttgattcacttctacaagag 33
```

>EL585542|EL585542.1 CF_WO0_12f01_SP6 Copepod Whole Organism, Normalized *Calanus finmarchicus* cDNA clone CF_WO0_12f01; 5' similar to NP_036312.2 F-box only protein 8 [Homo sapiens]. Score=145.98, Expect=9.00E-34, mRNA sequ Length = 611; score = 37.9 bits (17), expect = 3.8; identities = 20/23 (86%); strand = Plus / Plus

```
Query: 24 tgcttgattcacttcwwyaagag 46
          |||||||||||||||   |||||
Sbjct: 18 tgcttgattcacttctacaagag 40
```

>EL585529|EL585529.1 CF_WO0_12d12_SP6 Copepod Whole Organism, Normalized *Calanus finmarchicus* cDNA clone CF_WO0_12d12; 5' similar to NP_001002657.1 hypothetical protein LOC436930 [Danio rerio]. Score=111.31, Expect=2.80E-2 Length = 650; score = 37.9 bits (17), expect = 3.8; identities = 20/23 (86%); strand = Plus / Plus

```
Query: 23 ttgcttgattcacttcwwyaaga 45
          ||||||||||||||||   ||||
Sbjct: 8  ttgcttgattcacttctacaaga 30
```

>EL585512|EL585512.1 CF_WO0_12c01_SP6 Copepod Whole Organism, Normalized *Calanus finmarchicus* cDNA clone CF_WO0_12c01; 5' similar to Q9GR88 Eukaryotic peptide chain release factor subunit 1 (eRF1) (Eukaryotic release facto Length = 691; score = 37.9 bits (17), expect = 3.8; identities = 20/23 (86%); strand = Plus / Plus

```
Query: 24 tgcttgattcacttcwwyaagag 46
          |||||||||||||||   |||||
Sbjct: 9  tgcttgattcacttctacaagag 31
```

>EL585492|EL585492.1 CF_WO0_12a03_SP6 Copepod Whole Organism, Normalized *Calanus finmarchicus* cDNA clone CF_WO0_12a03; 5' similar to XP_547328.2 Acyl-CoA dehydrogenase, medium-chain specific, mitochondrial precursor (MCAD) Length = 624; score = 37.9 bits (17), expect = 3.8; identities = 20/23 (86%); strand = Plus / Plus

```
Query: 24 tgcttgattcacttcwwyaagag 46
          |||||||||||||||   |||||
Sbjct: 14 tgcttgattcacttctacaagag 36
```

>EL585479|EL585479.1 CF_WO0_11h02_SP6 Copepod Whole Organism, Normalized *Calanus finmarchicus* cDNA clone CF_WO0_11h02; 5' similar to XP_967453.1 pyruvate dehydrogenase phosphatase

regulatory subunit [Tribolium castaneum]. Length = 634; score = 37.9 bits (17), expect = 3.8; identities = 20/23 (86%); strand = Plus / Plus

```
Query: 24 tgcttgattcacttcwwyaagag 46
          ||||||||||||||   |||||
Sbjct: 12 tgcttgattcacttctacaagag 34
```

>EL585458|EL585458.1 CF_W00_11f04_SP6 Copepod Whole Organism, Normalized *Calanus finmarchicus* cDNA clone CF_W00_11f04; 5' similar to XP_314928.2 ENSANGP00000012460 [Anopheles gambiae str. PEST]. Score=287.73, Expect=2.30E- Length = 659; score = 37.9 bits (17), expect = 3.8; identities = 20/23 (86%); strand = Plus / Plus

```
Query: 24 tgcttgattcacttcwwyaagag 46
          ||||||||||||||   |||||
Sbjct: 2  tgcttgattcacttctacaagag 24
```

>EH667186|EH667186.1 CF_W00_10h11_SP6 Copepod Whole Organism, Normalized *Calanus finmarchicus* cDNA clone CF_W00_10h11; 5' similar to ref|XP_393085.2| DIVDICTED: similar to F-actin capping protein subunit beta [Apis. Score Length = 697; score = 37.9 bits (17), expect = 3.8; identities = 20/23 (86%); strand = Plus / Plus

```
Query: 24 tgcttgattcacttcwwyaagag 46
          ||||||||||||||   |||||
Sbjct: 2  tgcttgattcacttctacaagag 24
```

>EH667174|EH667174.1 CF_W00_10g11_SP6 Copepod Whole Organism, Normalized *Calanus finmarchicus* cDNA clone CF_W00_10g11; 5' similar to ref|XP_001120287.1| DIVDICTED: similar to CG8412-PA - Apis mellifera. Score = 201 bits (5 Length = 700; score = 37.9 bits (17), expect = 3.8; identities = 20/23 (86%); strand = Plus / Plus

```
Query: 24 tgcttgattcacttcwwyaagag 46
          ||||||||||||||   |||||
Sbjct: 11 tgcttgattcacttctacaagag 33
```

>EH667148|EH667148.1 CF_W00_10e09_SP6 Copepod Whole Organism, Normalized *Calanus finmarchicus* cDNA clone CF_W00_10e09; 5' similar to ref|XP_342651.3| DIVDICTED: similar to inosine monophosphate dehydrogenase 1 isoform. Sco Length = 637; score = 37.9 bits (17), expect = 3.8; identities = 20/23 (86%); strand = Plus / Plus

```
Query: 24 tgcttgattcacttcwwyaagag 46
          ||||||||||||||   |||||
Sbjct: 2  tgcttgattcacttctacaagag 24
```

>EH667121|EH667121.1 CF_W00_10c04_SP6 Copepod Whole Organism, Normalized *Calanus finmarchicus* cDNA clone CF_W00_10c04; 5' similar to ref|NP_001011178.1| F-box and WD-40 domain protein 2 - Xenopus tropicalis. Score = 80.1 b Length = 619; score = 37.9 bits (17), expect = 3.8; identities = 20/23 (86%); strand = Plus / Plus

```
Query: 24 tgcttgattcacttcwwyaagag 46
```

|||||||||||||| |||||
Sbjct: 8 tgcttgattcacttctacaagag 30

>EH667112|EH667112.1 CF_WO0_10b07_SP6 Copepod Whole Organism, Normalized *Calanus finmarchicus* cDNA clone CF_WO0_10b07; 5' , mRNA sequence. Length = 671; score = 37.9 bits (17), expect = 3.8; identities = 20/23 (86%); strand = Plus / Plus

Query: 24 tgcttgattcacttcwwyaagag 46
|||||||||||||| |||||
Sbjct: 11 tgcttgattcacttctacaagag 33

>EH667100|EH667100.1 CF_WO0_10a07_SP6 Copepod Whole Organism, Normalized *Calanus finmarchicus* cDNA clone CF_WO0_10a07; 5' similar to ref|XP_795871.1| DIVDICTED: similar to De-etiolated homolog 1 (Arabidopsis). Score = 220 Length = 706; score = 37.9 bits (17), expect = 3.8; identities = 20/23 (86%); strand = Plus / Plus

Query: 24 tgcttgattcacttcwwyaagag 46
|||||||||||||| |||||
Sbjct: 2 tgcttgattcacttctacaagag 24

>EH667094|EH667094.1 CF_WO0_10a01_SP6 Copepod Whole Organism, Normalized *Calanus finmarchicus* cDNA clone CF_WO0_10a01; 5' , mRNA sequence. Length = 732; score = 37.9 bits (17), expect = 3.8; identities = 20/23 (86%); strand = Plus / Plus

Query: 24 tgcttgattcacttcwwyaagag 46
|||||||||||||| |||||
Sbjct: 16 tgcttgattcacttctacaagag 38

>EH667069|EH667069.1 CF_WO0_09f12_SP6 Copepod Whole Organism, Normalized *Calanus finmarchicus* cDNA clone CF_WO0_09f12; 5' similar to ref|XP_001097946.1| DIVDICTED: similar to vacuolar protein sorting 33B (yeast homolog)). Length = 606; score = 37.9 bits (17), expect = 3.8; identities = 20/23 (86%); strand = Plus / Plus

Query: 24 tgcttgattcacttcwwyaagag 46
|||||||||||||| |||||
Sbjct: 12 tgcttgattcacttctacaagag 34

>EH667059|EH667059.1 CF_WO0_09e12_SP6 Copepod Whole Organism, Normalized *Calanus finmarchicus* cDNA clone CF_WO0_09e12; 5' similar to ref|XP_700365.1| DIVDICTED: similar to OMA1 homolog, zinc metallopeptidase, partial. Scor Length = 687; score = 37.9 bits (17), expect = 3.8; identities = 20/23 (86%); strand = Plus / Plus

Query: 24 tgcttgattcacttcwwyaagag 46
|||||||||||||| |||||
Sbjct: 10 tgcttgattcacttctacaagag 32

>EH667046|EH667046.1 CF_WO0_09d07_SP6 Copepod Whole Organism, Normalized *Calanus finmarchicus* cDNA clone CF_WO0_09d07; 5' similar to emb|CAG09753.1| unnamed protein product - Tetraodon

nigroviridis. Score = 142 bits (358), Length = 683; score = 37.9 bits (17), expect = 3.8; identities = 20/23 (86%); strand = Plus / Plus

```
Query: 24 tgcttgattcacttcwwyaagag 46
          |||||||||||||||   |||||
Sbjct: 2  tgcttgattcacttctacaagag 24
```

>EH667029|EH667029.1 CF_W00_09b11_SP6 Copepod Whole Organism, Normalized *Calanus finmarchicus* cDNA clone CF_W00_09b11; 5' , mRNA sequence. Length = 696; score = 37.9 bits (17), expect = 3.8; identities = 20/23 (86%); strand = Plus / Plus

```
Query: 24 tgcttgattcacttcwwyaagag 46
          |||||||||||||||   |||||
Sbjct: 12 tgcttgattcacttctacaagag 34
```

>EH666952|EH666952.1 CF_W00_08d03_SP6 Copepod Whole Organism, Normalized *Calanus finmarchicus* cDNA clone CF_W00_08d03; 5' , mRNA sequence. Length = 722; score = 37.9 bits (17), expect = 3.8; identities = 20/23 (86%); strand = Plus / Plus

```
Query: 24 tgcttgattcacttcwwyaagag 46
          |||||||||||||||   |||||
Sbjct: 13 tgcttgattcacttctacaagag 35
```

>EH666938|EH666938.1 CF_W00_08c01_SP6 Copepod Whole Organism, Normalized *Calanus finmarchicus* cDNA clone CF_W00_08c01; 5' similar to ref|NP_001072464.1| cysteine endopeptidase AUT-like (10128) - Xenopus tropicalis. Score = Length = 672; score = 37.9 bits (17), expect = 3.8; identities = 20/23 (86%); strand = Plus / Plus

```
Query: 24 tgcttgattcacttcwwyaagag 46
          |||||||||||||||   |||||
Sbjct: 2  tgcttgattcacttctacaagag 24
```

>EH666922|EH666922.1 CF_W00_08a09_SP6 Copepod Whole Organism, Normalized *Calanus finmarchicus* cDNA clone CF_W00_08a09; 5' , mRNA sequence. Length = 792; score = 37.9 bits (17), expect = 3.8; identities = 20/23 (86%); strand = Plus / Plus

```
Query: 24 tgcttgattcacttcwwyaagag 46
          |||||||||||||||   |||||
Sbjct: 13 tgcttgattcacttctacaagag 35
```

>EH666909|EH666909.1 CF_W00_07h08_SP6 Copepod Whole Organism, Normalized *Calanus finmarchicus* cDNA clone CF_W00_07h08; 5' similar to gb|AAH95104.1| Zgc:109981 protein - Danio rerio. Score = 126 bits (316), expect = 9e-28, Length = 641; score = 37.9 bits (17), expect = 3.8; identities = 20/23 (86%); strand = Plus / Plus

```
Query: 24 tgcttgattcacttcwwyaagag 46
          |||||||||||||||   |||||
Sbjct: 9  tgcttgattcacttctacaagag 31
```

>EH666793|EH666793.1 CF_WO0_06f10_SP6 Copepod Whole Organism, Normalized *Calanus finmarchicus* cDNA clone CF_WO0_06f10; 5' similar to emb|CAG10633.1| unnamed protein product - Tetraodon nigroviridis. Score = 130 bits (326), Length = 634; score = 37.9 bits (17), expect = 3.8; identities = 20/23 (86%); strand = Plus / Plus

```
Query: 24 tgcttgattcacttcwwyaagag 46
          ||||||||||||||   |||||
Sbjct: 2  tgcttgattcacttctacaagag 24
```

>EH666770|EH666770.1 CF_WO0_06d09_SP6 Copepod Whole Organism, Normalized *Calanus finmarchicus* cDNA clone CF_WO0_06d09; 5' similar to ref|XP_624707.1| DIVDICTED: similar to CG6904-PA, isoform A - Apis mellifera. Score = 237 Length = 662; score = 37.9 bits (17), expect = 3.8; identities = 20/23 (86%); strand = Plus / Plus

```
Query: 24 tgcttgattcacttcwwyaagag 46
          ||||||||||||||   |||||
Sbjct: 2  tgcttgattcacttctacaagag 24
```

>EH666768|EH666768.1 CF_WO0_06d07_SP6 Copepod Whole Organism, Normalized *Calanus finmarchicus* cDNA clone CF_WO0_06d07; 5' , mRNA sequence. Length = 723; score = 37.9 bits (17), expect = 3.8; identities = 20/23 (86%); strand = Plus / Plus

```
Query: 24 tgcttgattcacttcwwyaagag 46
          ||||||||||||||   |||||
Sbjct: 15 tgcttgattcacttctacaagag 37
```

>EH666764|EH666764.1 CF_WO0_06d03_SP6 Copepod Whole Organism, Normalized *Calanus finmarchicus* cDNA clone CF_WO0_06d03; 5' , mRNA sequence. Length = 671; score = 37.9 bits (17), expect = 3.8; identities = 20/23 (86%); strand = Plus / Plus

```
Query: 24 tgcttgattcacttcwwyaagag 46
          ||||||||||||||   |||||
Sbjct: 14 tgcttgattcacttctacaagag 36
```

>EH666758|EH666758.1 CF_WO0_06c09_SP6 Copepod Whole Organism, Normalized *Calanus finmarchicus* cDNA clone CF_WO0_06c09; 5' similar to ref|XP_624527.2| DIVDICTED: similar to CG40196-PA.3 - Apis mellifera. Score = 112 bits (2 Length = 736; score = 37.9 bits (17), expect = 3.8; identities = 20/23 (86%); strand = Plus / Plus

```
Query: 24 tgcttgattcacttcwwyaagag 46
          ||||||||||||||   |||||
Sbjct: 2  tgcttgattcacttctacaagag 24
```

>EH666734|EH666734.1 CF_WO0_06a09_SP6 Copepod Whole Organism, Normalized *Calanus finmarchicus* cDNA clone CF_WO0_06a09; 5' similar to ref|NP_001018451.1| hypothetical protein LOC553642 - Danio rerio. Score = 155 bits (391), Length = 594; score = 37.9 bits (17), expect = 3.8; identities = 20/23 (86%); strand = Plus / Plus

```
Query: 24 tgcttgattcacttcwwyaagag 46
```

|||||||||||||| |||||
Sbjct: 6 tgcttgattcacttctacaagag 28

>EH666701|EH666701.1 CF_WO0_05f11_SP6 Copepod Whole Organism, Normalized *Calanus finmarchicus* cDNA clone CF_WO0_05f11; 5' similar to ref|ZP_01596943.1| major facilitator superfamily MFS_1 - Marinomonas sp. MWYL1. Score = 7 Length = 644; score = 37.9 bits (17), expect = 3.8; identities = 20/23 (86%); strand = Plus / Plus

Query: 24 tgcttgattcacttcwwyaagag 46
|||||||||||||| |||||
Sbjct: 10 tgcttgattcacttctacaagag 32

>EH666682|EH666682.1 CF_WO0_05e04_SP6 Copepod Whole Organism, Normalized *Calanus finmarchicus* cDNA clone CF_WO0_05e04; 5' similar to ref|XP_392877.1| DIVDICTED: similar to Unc-76 CG3981-PB, isoform B isoform 1 [Apis. Score Length = 746; score = 37.9 bits (17), expect = 3.8; identities = 20/23 (86%); strand = Plus / Plus

Query: 24 tgcttgattcacttcwwyaagag 46
|||||||||||||| |||||
Sbjct: 36 tgcttgattcacttctacaagag 58

>EH666680|EH666680.1 CF_WO0_05e02_SP6 Copepod Whole Organism, Normalized *Calanus finmarchicus* cDNA clone CF_WO0_05e02; 5' similar to ref|XP_001200801.1| DIVDICTED: hypothetical protein - Strongylocentrotus purpuratus. Scor Length = 727; score = 37.9 bits (17), expect = 3.8; identities = 20/23 (86%); strand = Plus / Plus

Query: 24 tgcttgattcacttcwwyaagag 46
|||||||||||||| |||||
Sbjct: 11 tgcttgattcacttctacaagag 33

>EH666654|EH666654.1 CF_WO0_05b11_SP6 Copepod Whole Organism, Normalized *Calanus finmarchicus* cDNA clone CF_WO0_05b11; 5' similar to ref|XP_973047.1| DIVDICTED: similar to CG7057-PA, isoform A - Tribolium castaneum. Score Length = 645; score = 37.9 bits (17), expect = 3.8; identities = 20/23 (86%); strand = Plus / Plus

Query: 24 tgcttgattcacttcwwyaagag 46
|||||||||||||| |||||
Sbjct: 15 tgcttgattcacttctacaagag 37

>EH666644|EH666644.1 CF_WO0_05b01_SP6 Copepod Whole Organism, Normalized *Calanus finmarchicus* cDNA clone CF_WO0_05b01; 5' similar to ref|XP_973304.1| DIVDICTED: similar to CG8808-PA - Tribolium castaneum. Score = 212 bits Length = 681; score = 37.9 bits (17), expect = 3.8; identities = 20/23 (86%); strand = Plus / Plus

Query: 24 tgcttgattcacttcwwyaagag 46
|||||||||||||| |||||
Sbjct: 11 tgcttgattcacttctacaagag 33

>EH666630|EH666630.1 CF_W00_04h11_SP6 Copepod Whole Organism, Normalized *Calanus finmarchicus* cDNA clone CF_W00_04h11; 5' , mRNA sequence. Length = 678; score = 37.9 bits (17), expect = 3.8; identities = 20/23 (86%); strand = Plus / Plus

```
Query: 24 tgcttgattcacttcwwyaagag 46
          ||||||||||||||   |||||
Sbjct: 13 tgcttgattcacttctacaagag 35
```

>EH666609|EH666609.1 CF_W00_04f11_SP6 Copepod Whole Organism, Normalized *Calanus finmarchicus* cDNA clone CF_W00_04f11; 5' similar to ref|XP_966417.1| DIVDICTED: similar to protein phosphatase 1, catalytic subunit,. Score = Length = 683; score = 37.9 bits (17), expect = 3.8; identities = 20/23 (86%); strand = Plus / Plus

```
Query: 24 tgcttgattcacttcwwyaagag 46
          ||||||||||||||   |||||
Sbjct: 9  tgcttgattcacttctacaagag 31
```

>EH666584|EH666584.1 CF_W00_04d07_SP6 Copepod Whole Organism, Normalized *Calanus finmarchicus* cDNA clone CF_W00_04d07; 5' similar to ref|XP_395294.3| DIVDICTED: similar to CG9924-PC, isoform C, partial [Apis. Score = 93.2 Length = 637; score = 37.9 bits (17), expect = 3.8; identities = 20/23 (86%); strand = Plus / Plus

```
Query: 24 tgcttgattcacttcwwyaagag 46
          ||||||||||||||   |||||
Sbjct: 14 tgcttgattcacttctacaagag 36
```

>EH666578|EH666578.1 CF_W00_04d01_SP6 Copepod Whole Organism, Normalized *Calanus finmarchicus* cDNA clone CF_W00_04d01; 5' similar to ref|XP_001234118.1| DIVDICTED: hypothetical protein - Gallus gallus. Score = 71.2 bits (1 Length = 724; score = 37.9 bits (17), expect = 3.8; identities = 20/23 (86%); strand = Plus / Plus

```
Query: 24 tgcttgattcacttcwwyaagag 46
          ||||||||||||||   |||||
Sbjct: 1  tgcttgattcacttctacaagag 23
```

>EH666549|EH666549.1 CF_W00_04a06_SP6 Copepod Whole Organism, Normalized *Calanus finmarchicus* cDNA clone CF_W00_04a06; 5' , mRNA sequence. Length = 716; score = 37.9 bits (17), expect = 3.8; identities = 20/23 (86%); strand = Plus / Plus

```
Query: 24 tgcttgattcacttcwwyaagag 46
          ||||||||||||||   |||||
Sbjct: 11 tgcttgattcacttctacaagag 33
```

>EH666461|EH666461.1 CF_W00_03a10_SP6 Copepod Whole Organism, Normalized *Calanus finmarchicus* cDNA clone CF_W00_03a10; 5' , mRNA sequence. Length = 692; score = 37.9 bits (17), expect = 3.8; identities = 20/23 (86%); strand = Plus / Plus

```
Query: 24 tgcttgattcacttcwwyaagag 46
          ||||||||||||||   |||||
```

```
Sbjct: 21 tgcttgattcacttctacaagag 43
```

>EH666455|EH666455.1 CF_WO0_03a04_SP6 Copepod Whole Organism, Normalized *Calanus finmarchicus* cDNA clone CF_WO0_03a04; 5’ similar to gb|EAT36614.1| actin - Aedes aegypti. Score = 273 bits (699), expect = 3e-72, mRNA sequen Length = 621; score = 37.9 bits (17), expect = 3.8; identities = 20/23 (86%); strand = Plus / Plus

```
Query: 16 gctwawwttgcttgattcacttc 38
          ||| |  ||||||||||||||||
Sbjct: 1  gctaatattgcttgattcacttc 23
```

>EH666411|EH666411.1 CF_WO0_02e05_SP6 Copepod Whole Organism, Normalized *Calanus finmarchicus* cDNA clone CF_WO0_02e05; 5’ similar to gb|AAX62435.1| ribosomal protein L4 - Lysiphlebus testaceipes. Score = 347 bits (889), Ex Length = 702; score = 37.9 bits (17), expect = 3.8; identities = 20/23 (86%); strand = Plus / Plus

```
Query: 24 tgcttgattcacttcwwyaagag 46
          |||||||||||||||   |||||
Sbjct: 14 tgcttgattcacttctacaagag 36
```

>EH666344|EH666344.1 CF_WO0_01g09_SP6 Copepod Whole Organism, Normalized *Calanus finmarchicus* cDNA clone CF_WO0_01g09; 5’ similar to ref|XP_624697.1| DIVDICTED: similar to 26S proteasome non-ATPase regulatory subunit. Scor Length = 553; score = 37.9 bits (17), expect = 3.8; identities = 20/23 (86%); strand = Plus / Plus

```
Query: 24 tgcttgattcacttcwwyaagag 46
          |||||||||||||||   |||||
Sbjct: 14 tgcttgattcacttctacaagag 36
```

>DY657059|DY657059.1 Cm_mx1_88a03_SP6 Green Shore Crab Multiple Tissue, Normalized Carcinus maenas cDNA clone Cm_mx1_88a03 5’ similar to Similar to emb|CAI64731.1| aurora/Ipl1p-related kinase - Marthasterias *glacialis*. Sc Length = 678; score = 37.9 bits (17), expect = 3.8; identities = 20/23 (86%); strand = Plus / Plus

```
Query: 24 tgcttgattcacttcwwyaagag 46
          |||||||||||||||   |||||
Sbjct: 2  tgcttgattcacttctacaagag 24
```

Table S3a2. nblast of CopepodSL GTCTAAWACGACCAAGCTWAWWTTGCTTGATTCACTTCWWYAAGAG against DDBJ/EMBL/GenBank nr/nt, EST and TSA databases.

Note 1: Database: nt+tsa+est; 106,461,619 sequences; 119,623,300,248 total letters. Matrix: blastn matrix: 1 -3; Gap Penalties: Existence: 5, Extension: 2.

Note 2: tblastx of the 64 non-copepod sequences against the databases hit only copepod transcripts except 11 sequences without hit to any known sequences. Details: 2 sequences to be Fish intestine microbial community metagenome (LA314161 and LA313185); 16 of Sea ice eukaryotic metatranscriptome [JZ760438, JZ758879, JZ760438, JZ757307, JZ757041, JZ756818, JZ756648, JZ756141, JZ755703, JZ755442, JZ760213, JZ755909, JZ755697, JZ752877, JZ758795, JZ752592); 30 amphioxus Branchiostoma floridae cDNAs (BW777469, BW782010, FE575842, FE564053, FE564052, BW849092, BW813862, BW809910, BW804361, BW802526, BW792514, BW785609, BW779325, BW778946, BW772893, BW772235, FE596692, FE591355, FE573549, FE568647, FE565967, FE563520, FE562105, FE546172, FE596722, FE596360, BW838699, BW770801, BW773653; the last 5 (underline) no hit]; 1 Dogfish Shark cDNA (EB740555); 3 Pleurobrachia pileus cDNAs (FQ001105, FP988813, FQ001353); 12 Green Shore Crab Carcinus maenas cDNAs [DY656934, DY656747, DY657059, DY656243, DV943741, DV642724, DW250273, DW250218, DN796273, DN796153, DN739048, DN738903; the last 6 (underline) no hit].

>gi|648686185|dbj|LA314161.1| TSA: Fish metagenome RNA, contig: contig17279_C2388668, isolate: YEZ, transcribed RNA sequence. Transcriptome Shotgun Assembly of microbial community in Ctenopharyngodon idella intestine

ATATTGCTTGATTCACTTCTACAAGAGAGAGAACCATGGGTCGGGTCCGTACCAAGACGGTCAAAAAGGCCGCCAGGGTCATCATTGAGAAGT
ACTACACCAAGCTCACCCTGGACTTTCATACCAATAAGAGGATCATTGAGGAGGTGGCTCTCATTCCCTCCAAACCTCTCCGTAACAAGATTG
GTGGCTTCATCACCCATTTGATGAAACGTCTACAGCGCTCCACAGTACGCGGCATCTCCATCAAGCTGCAGGAGGAGGAGAGAGAGAGGAGGG
ATAACTATGTCCCCGATGTCTCTGCCCTTGAACAGGACGTCATTGAGGTGGACAGTGAGACCAAGGACATGCTCAAGATGATGGACTTCAACT
CAATTCCTGTTCAGCTCACAAGTGGAAACAGGAAAGGGGGCCAGGGATTCCAGGCCAGGAGCTAGAACGGCTGTATCTTCATCTTGACCAACT
TGCCTAAGAGCAGAACTGCCTCTGCCACCAACACAGTTCCAGCCCAGTGTTTGTGTGCGAGTATTAGCACTGCTAGTAGAACTCTCTGAAA

EST_lsaA_evv_1234076 lsaAevv mixed_tissue Lepeophtheirus salmonis (Atlantic Norway) cDNA Lepeophtheirus salmonis cDNA clone lsaA_evv_501_122 5', mRNA sequence.

Sequence ID: gb|GW646414.1|Length: 562Number of Matches: 7

252 bits(544) 1e-66 108/128(84%) 115/128(89%) 0/128(0%) +3/+1

```
Query  36   MGRVRTKTVKKAARVIIEKYYTKLTLDFHTNKRIIEEVALIPSKPLRNKIGGFITHLMKR  215
            MGRVRTKT+KKAARVIIEKYYTKL LDFHTN RIIEEVA+IPSKPLRNKI GFITHLMKR
Sbjct  37   MGRVRTKTIKKAARVIIEKYYTKLNLDFHTNNRIIEEVAMIPSKPLRNKIAGFITHLMKR  216

Query  216  LQRSTVRGISIKLQEEERERRDNYVPDVSALEQDVIEVDSETKDMLKMMDFNSIPVQLTS  395
```

```
           LQRSTVRGISIKLQEEERERRDNYVP+VSAL QD+I++DSETKDMLK MDFN+IP    S
Sbjct  217  LQRSTVRGISIKLQEEERERRDNYVPEVSALVQDIIDIDSETKDMLKAMDFNNIPGVQLS  396

Query  396  GNRKGGQG  419
              R GG G
Sbjct  397  TTRTGGGG  420
```

>gi|648698115|dbj|LA313185.1| TSA: Fish metagenome RNA, contig: contig16303_C2386388, isolate: YEZ, transcribed RNA sequence. Transcriptome Shotgun Assembly of microbial community in Ctenopharyngodon idella intestine

```
ATATTGCTTGATTCACTTCTACAAGAGAAGTTAAAAAAACCGCTGGAAAAATGGGTCGCATGCACGCACCAGGTAAGGGTATTTCCCAGTCTG
CCCTGCCCTACAGGCGTTCCGTACCCCAGTGGATGAAGATGACCAGCGATGAGGTCAAGGACCAGATCTTCAAACTGGCCAAGAAGGGGCTCA
CCCCCTCCCAGATCGGTGTCATCCTCAGAGACTCCCACGGTGTTGCTCAGGTCCGTTTTATCACCGGCAACAAGATTCTTCGTATCTTGAAGT
CCAAGGCCCTGGCACCCGACCTACCCGAGGACATGTACTTCCTCATCAAGAAGGCCGTGTCCATCCGCAAGCACCTGGAAAGGAACAGGAAGG
ACAAGGACGCTAAGTTCAGGCTGATTCTGGTCGAGTCAAGGATTCACCGCTTGGGCAGGTACTACAAGATCAAGAGCGTTCTGCCCCCCAACT
GGAAGTACGAGTCTAGCACAGCCTCGACCCTCGTGGCCTAACTGTTCAAGTGCGGAATTCTGAAATAAACTCG
```

EST_ccle_evs_1034274 ccleevs mixed_tissue Caligus clemensi cDNA Caligus clemensi cDNA clone ccle_evs_513_066 5', mRNA sequence.
Sequence ID: gb|GO405439.1|Length: 617Number of Matches: 8

319 bits(691) 7e-87 132/153(86%) 145/153(94%) 0/153(0%) +3/+3

```
Query  48   KMGRMHAPGKGISQSALPYRRSVPQWMKMTSDEVKDQIFKLAKKGLTPSQIGVILRDSHG  227
            KMGRMHAPGKGISQSALPYRRSVP W+K+T  EVKDQI+KLAKKGLTPSQIGVILRDSHG
Sbjct  87   KMGRMHAPGKGISQSALPYRRSVPAWLKLTPVEVKDQIYKLAKKGLTPSQIGVILRDSHG  266

Query  228  VAQVRFITGNKILRILKSKALAPDLPEDMYFLIKKAVSIRKHLERNRKDKDAKFRLILVE  407
             AQVRF+TGNKILRILKS+ LAPDLPED+Y+LIKKAVSIRKH+ERNRKD+DAKFRLIL+E
Sbjct  267  AAQVRFVTGNKILRILKSEGLAPDLPEDIYYLIKKAVSIRKHMERNRKDRDAKFRLILIE  446

Query  408  SRIHRLGRYYKIKSVLPPNWKYESSTASTLVA*  506
            SRI+RL RYYKIK VLPP WKYES+TASTLV+*
Sbjct  447  SRIYRLARYYKIKGVLPPTWKYESATASTLVS*  545
```

>gi|698735374|gb|JZ760438.1|JZ760438 awis5P0020M09_F.ab1 Sea ice eukaryotic metatranscriptome Weddell Sea 2 uncultured organism cDNA, mRNA sequence

```
TCTAAAACGACCAAGCTAATATTGCTTGATTCACTTCTACAAGAGAAATCTTCCCAACTCTAAAACAAAC
CATGCAGAACGAAGCTGGAGAATATGTCGACCTCTACATCCCAAGGAAGTGCTCCGCCTCCAACAGGATC
CTCGCCGCCAAGGACCACGCCTCCATCCAGATCAACCTCGCAGAGGTTGACGAGGAGACAGGTAGAATGA
CCGGTGGATACAAGACCTACGCTATCTGTGGAGCCATTAGGAGAATGGGAGAGTCTGATGACTGTATCAA
CAGACTAGCCAAGAAGGATGGTGTCATTGCCAAGAACTTCTAAACATATGATATCTCTTGTATGTTGTAC
TTGATGTACTTTGTATCTTGTACTTCTTGTACTTGATGTACTTTGTATCTTGTACTTGATGTACTTTGAC
AATGTATACTTGCACAATTTATGTACTTGATGTACATTTGTTTTTATTTTGTACCTGTACTTTGTTCTTT
GTAAATATTCAACTGAATAAATAGGTATGATAT
```

CF_WOO_23e09_SP6 Copepod Whole Organism, Normalized Calanus finmarchicus cDNA clone
CF_WOO_23e09 5' similar to CAJ17217.1 ribosomal protein S21e [Sphaerius sp. APV-2005].
Score=137.5, Expect=1.10E-31, mRNA sequence.
Sequence ID: gb|EL586545.1|Length: 370Number of Matches: 14

185 bits(399) 8e-50 76/92(83%) 83/92(90%) 0/92(0%) +3/+1

```
Query  72   MQNEAGEYVDLYIPRKCSASNRILAAKDHASIQINLAEVDEETGRMTGGYKTYAICGAIR  251
            MQNEAGEYVD+YIPRKCSASNRIL+AKDHASIQINLAEVDE TGRMTG +KTYAICGAIR
Sbjct  52   MQNEAGEYVDMYIPRKCSASNRILSAKDHASIQINLAEVDESTGRMTGAFKTYAICGAIR  231

Query  252  RMGESDDCINRLAKKDGVIAKNF*TYDISCML  347
            RMGESDDCINRLAKKDG++ K F*     C++
Sbjct  232  RMGESDDCINRLAKKDGILDKKF*NVF*ICLI  327
```

>gi|698733806|gb|JZ758879.1|JZ758879 awis5P0013P12_F.ab1 Sea ice eukaryotic metatranscriptome Weddell Sea 2 uncultured organism cDNA, mRNA sequence

TCTAAAACGACCAAGCTAATATTGCTTGATTCACTTCTACAAGAGAGAAGTCACCATGTCTGCTGCAGAG
GCCGAAGCTCTGAATGCCGCTAATCTGGAAGCCCAGAAGAAGAGGAGGACCTTCCGTAAGTTTACATACC
GTGGAGTGGACCTGGACCAGCTCCTGGACCTCTCTCACGAGCAGCTCATGGAGCTCTTCCCCTGCAGAAT
CAGGAGGAAGTTCGCCAGGGGAATCAAGAGGAAGCCCATGGCTCTCATCAAGAAGCTCCGGAAGAAGAGG
AAGGAGTGCCCTGCCAACGAGAAGCCCGATGTCGTCAAGACTCACCTGAGGAACATGGTTATCGTACCCG
AGATGACCGGATCCATTGTTGGAGTGTACAACGGAAAGACCTTCAACCAGGTCGAGGTGAAGCCTGAGAT
GATTGGACACTATCTTGGAGAGTTCTCCATCTCTTACAAGCCCGTAAAGCACGGAAGGCCCGGTATTGGT
GCCACCCACTCCTCCAGATTCATCCCCCTCAAGTAGACCAACATCATCATGCTCCGCTTCTACACAATCT
TCTTTGTTAGCTATCAGAGCTCTATTCCATCTTGATGCTCTGTATTTATAAATATCATTTGCTTCATCTC
TTGATAGTCCGGTTTATGTGAACTATNAATTGGAATTAAAAACTAAATGTTTC

PU505-2004-09-30.ab1 Lepeophtheirus salmonis PU Lepeophtheirus salmonis cDNA, mRNA sequence.
Sequence ID: gb|HO702112.1|Length: 634Number of Matches: 6
277 bits(599) 4e-74 112/131(85%) 123/131(93%) 0/131(0%) +2/-3

```
Query  137  YRGVDLDQLLDLSHEQLMELFPCRIRRKFARGIKRKPMALIKKLRKKRKECPANEKPDVV  316
            YRGVDLDQLLD S+++LMELFPCRIRRK+ RG+KRK + L+ KLRKK+KEC   EKPDVV
Sbjct  494  YRGVDLDQLLDQSNKELMELFPCRIRRKYVRGLKRKQLTLMMKLRKKKKECLPMEKPDVV  315

Query  317  KTHLRNMVIVPEMTGSIVGVYNGKTFNQVEVKPEMIGHYLGEFSISYKPVKHGRPGIGAT  496
            KTHLRNMV+VPEMTGSIVGVYNGKTFNQVE+KPEMIGHYLGEFSI+YKPVKHGRPGIGAT
Sbjct  314  KTHLRNMVVVPEMTGSIVGVYNGKTFNQVEIKPEMIGHYLGEFSITYKPVKHGRPGIGAT  135
```

Query 497 HSSRFIPLK*T 529
           HSSRFIPLK*T
Sbjct 134 HSSRFIPLK*T 102

>gi|698735374|gb|JZ760438.1|JZ760438 awis5P0020M09_F.ab1 Sea ice eukaryotic metatranscriptome Weddell Sea 2 uncultured organism cDNA, mRNA sequence
TCTAAAACGACCAAGCTAATATTGCTTGATTCACTTCTACAAGAGAAATCTTCCCAACTCTAAAACAAAC
CATGCAGAACGAAGCTGGAGAATATGTCGACCTCTACATCCCAAGGAAGTGCTCCGCCTCCAACAGGATC
CTCGCCGCCAAGGACCACGCCTCCATCCAGATCAACCTCGCAGAGGTTGACGAGGAGACAGGTAGAATGA
CCGGTGGATACAAGACCTACGCTATCTGTGGAGCCATTAGGAGAATGGGAGAGTCTGATGACTGTATCAA
CAGACTAGCCAAGAAGGATGGTGTCATTGCCAAGAACTTCTAAACATATGATATCTCTTGTATGTTGTAC
TTGATGTACTTTGTATCTTGTACTTCTTGTACTTGATGTACTTTGTATCTTGTACTTGATGTACTTTGAC
AATGTATACTTGCACAATTTATGTACTTGATGTACATTTGTTTTTATTTTGTACCTGTACTTTGTTCTTT
GTAAATATTCAACTGAATAAATAGGTATGATAT
CF_WO0_23e09_SP6 Copepod Whole Organism, Normalized Calanus finmarchicus cDNA clone CF_WO0_23e09 5' similar to CAJ17217.1 ribosomal protein S21e [Sphaerius sp. APV-2005]. Score=137.5, Expect=1.10E-31, mRNA sequence.
Sequence ID: gb|EL586545.1|Length: 370Number of Matches: 14
185 bits(399) 8e-50 76/92(83%) 83/92(90%) 0/92(0%) +3/+1

Query 72   MQNEAGEYVDLYIPRKCSASNRILAAKDHASIQINLAEVDEETGRMTGGYKTYAICGAIR 251
            MQNEAGEYVD+YIPRKCSASNRIL+AKDHASIQINLAEVDE TGRMTG +KTYAICGAIR
Sbjct 52   MQNEAGEYVDMYIPRKCSASNRILSAKDHASIQINLAEVDESTGRMTGAFKTYAICGAIR 231

Query 252 RMGESDDCINRLAKKDGVIAKNF*TYDISCML 347
            RMGESDDCINRLAKKDG++ K F*   C++
Sbjct 232 RMGESDDCINRLAKKDGILDKKF*NVF*ICLI 327
>gi|698731109|gb|JZ757307.1|JZ757307 awis5P0007A02_F.ab1 Sea ice eukaryotic metatranscriptome Weddell Sea 2 uncultured organism cDNA, mRNA sequence
TCTAAAACGACCAAGCTAATATTGCTTGATTCACTTCTACAAGAGAAGAGAAGATGACTAACCCAACTGG
ATACCGTCGTGGAACTAGAGACATGTTCTCTAGGGCTTACAAGAAGAGGGGAGTTGAGCATCTGTCCACC
TACCTCAAGACCTACAAGATGGGAGACATTGTTGATGTCAAGGGATGTGGTGCCTTCCAGAAAGGTATGC
CCCATAAGGCCTACCACGGTAAGACTGGACGTGTTTTCAACGTGTCCAAGCGTGCCGTCGGTGTTGTCGT
CAACAAGAGGGTCAAGGGAAAGATCATCGGCAAGAGGATCTCCGTCCGTATCGAGCACGTTAAGCACTCC
CAGTGCAGGAAGGATTTCCTCGACAGAGTCCACTCCAACGAGGTGAAGAAGAAGGAGGCCAAGATCTCTG
GGAAGGATGTCATCTGCAAGAGGTTCCCCCTGGCTCCCAGACCTGGACACGTTGTCTCCACCAAGGACAA
CGCTCCCCAAGTCATTGGCCCAGTACCTTTCGAGTTCATTGCTTAAATTTGTCTTCTCTTTATTTTGAAA
TACCCATAAAAAATTTTGCTGCATCAACATCTTTCTATTGCAG
EST_lbra_evt_1114675 lbraevt mixed_tissue Lernaeocera branchialis cDNA Lernaeocera branchialis cDNA clone lbra_evt_020_115 3', mRNA sequence.
Sequence ID: gb|GO425613.1|Length: 591Number of Matches: 10

153 bits(329) 2e-55 60/68(88%) 64/68(94%) 0/68(0%) +3/-3

```
Query  54   MTNPTGYRRGTRDMFSRAYKKRGVEHLSTYLKTYKMGDIVDVKGCGAFQKGMPHKAYHGK  233
            MTNP GYRRGTRDMFSRAYKK+GVEHLSTYLK Y+ GDIVD+KG GAFQKGMPHK+YHGK
Sbjct  532  MTNPKGYRRGTRDMFSRAYKKKGVEHLSTYLKCYRNGDIVDIKGNGAFQKGMPHKSYHGK  353

Query  234  TGRVFNVS  257
            TGRVFNVS
Sbjct  352  TGRVFNVS  329
```

>gi|698730843|gb|JZ757041.1|JZ757041 awis5P0006D06_F.ab1 Sea ice eukaryotic metatranscriptome Weddell Sea 2 uncultured organism cDNA, mRNA sequence

TCTAAAACGACCAAGCTAATATTGCTTGATTCACTTCTACAAGAGAAGACAGTATGCAGTTGATTGTTAG
GTCCCAGAACACCCATGTCCTTGAGGTCTCCCCAGAGGCAAATGTTGAGACCCTGAGGGCTCAGCTGGCC
CAGCTGGAGGGCGTCTCTGTTGACATGCTGAACCTGTCCTGTGCTGGCTCTATGCCTCAGGATGGTATGA
GCATCTCTCTGTTGGCCAACCTACCCGTTGATGTCAACATCCCACTCATGGGAGGAAAGGTTCACGGGTC
CCTGGCTCGTGCTGGTAAGGTGCGTGGACAGACCCCTAAAGTTGATGCCGGGGAGAAGAAGAAGAAGAAG
ACCGGAAGATGCAAGAGGAGGATTCAGTACAACAGGAGATTCGTGAATGTCGTCGAGTCTTTTGGTAAGA
AGAAGGGCCCCAACTCCAACCAGGCTGATGCTGCCAAGTAGAGGGTTCCCACTCCCTCCCACGACCAGTC
TCAAACTGCAAAGTAGAGGGGTCCAAACCCCCCACTAGTAGGCTAGAGGCGCCAAATAGAGGGATGAAGA
ACATCTGATTTCATCACTTTATAATTAATTTGTTAAAATGTGATAATTGAATAAATATTGCAACAATT

LPU201-2007-07-30.ab1 Lepeophtheirus salmonis LPU Lepeophtheirus salmonis cDNA, mRNA sequence.

Sequence ID: gb|HO696401.1|Length: 801Number of Matches: 4

140 bits(300) 7e-33 63/94(67%) 78/94(82%) 0/94(0%) +3/+2

```
Query  168  LNLSCAGSMPQDGMSISLLANLPVDVNIPLMGGKVHGSLARAGKVRGQTPKVDAGEkkkk  347
            +++  AG+  +   S+  L+ L +DVN+ L+GGKVHGSLARAGKVRGQTPKV+A EKKKK
Sbjct  218  ISIFAAGNPVEKETSLEALSGLTLDVNVKLLGGKVHGSLARAGKVRGQTPKVEAQEKKKK  397

Query  348  kTGRCKRRIQYNRRFVNVVESFGKKKGPNSNQAD  449
            KTGR KRRIQYNRRFVNVV+SFG+++GPNSN  D
Sbjct  398  KTGRAKRRIQYNRRFVNVVQSFGRRRGPNSNS*D  499
```

>gi|698730620|gb|JZ756818.1|JZ756818 awis5P0005I09_F.ab1 Sea ice eukaryotic metatranscriptome Weddell Sea 2 uncultured organism cDNA, mRNA sequence

TCTAAAACGACCAAGCTAATATTGCTTGATTCACTTCTACAAGAGATATCAGAATGGCCGATAAATTGAA
TGATCTTGCTGGAAAATTAGGAAAGGCCCCCAAGGGTCTGGGTGCAGGTGCCAAGCTGCTGGGTGTTGCC
CTGGCCGGTGCCTATGGTCTCCAGCAGAGCATGTACACAGTGGAGGGAGGACACAGGGCCATCATGTTCA
ACAGAATCGGCGGAGTTCAGGACTATGCACTCACTGAGGGTCTCCACTTCAGGGTCCCCTGGTTCCAGTA
TCCCATCATCTATGATATCCGTGCCAAGCCCAGGAAGATCACCTCCCCCACCGGAAGCAAGGATTTGCAG
ATGGTTAATATTTCCCTACGAGTCCTGTCTCGTCCTGACTCTGGTCACCTCTCCATTCTTCATAAGGAAC

TAGGGCAAGACTTTGATGAGAAGGTGCTCCCATCTATTTGCAATGAAGTACTGAAAGCAGTTGTTGCTAA
ATTTAATGCCTCT
EST_lsaA_evu_1224027 lsaAevu mixed_tissue Lepeophtheirus salmonis (Atlantic Canada) cDNA
Lepeophtheirus salmonis cDNA clone lsaA_evu_509_057 3', mRNA sequence.
Sequence ID: gb|GW642629.1|Length: 870Number of Matches: 9
237 bits(513) 2e-62 95/114(83%) 107/114(93%) 0/114(0%) +3/-2

```
Query  162  QQSMYTVEGGHRAIMFNRIGGVQDYALTEGLHFRVPWFQYPIIYDIRAKPRKITSPTGSK  341
            QQ+MYTVEGGHRAIMF+RIGG+QD  +TEGLHFR+PWFQYPIIYDIR++PRKITSPTGSK
Sbjct  866  QQAMYTVEGGHRAIMFSRIGGIQDTIMTEGLHFRIPWFQYPIIYDIRSRPRKITSPTGSK  687

Query  342  DLQMVNISLRVLSRPDSGHLSILHKELGQDFDEKVLPSICNEVLKAVVAKFNAS  503
            DLQMVNISLRVLSRP+S  +  +H+ELG+DFDEKVLPSICNEVLK VVAKFNAS
Sbjct  686  DLQMVNISLRVLSRPESMSIPTIHRELGRDFDEKVLPSICNEVLKGVVAKFNAS  525
```

>gi|698729184|gb|JZ756648.1|JZ756648 awis5P0004P22_F.ab1 Sea ice eukaryotic metatranscriptome
Weddell Sea 2 uncultured organism cDNA, mRNA sequence
TCTAAAACGACCAAGCTAATATTGCTTGATTCACTTCTACAAGAGCATTCATTTCAGAGAAAAATGGGGA
ATGAGTCTGATGATGAGGAAGAACCTATAACCTTATCTTCAGACACATTTGCGGCCCTTTCCCAATTTTA
CAAAGAGCAAGATGAGAGGGAGAAAGTATGGGAGGAGGGAGGGGAGAGTGTGGACGTTTCTGTCCAGGTC
AAGGAAGATTGGCAGCTCTCCCAGTTCTGGTACAGCCAGCAGACGGCGGAAACACTCGCCAAAATGGTGA
TGAGCACTGTCGGGGAGGGGGGAAGTATCGCCTGTGTAAGCGCCCCCACCTTATACCGTGCTGTGAAATT
ACTGGACAAAGACTTTGCCGCCTCTATATTTGAATACGATAAACGGTTTGCAAGCTATGGTTCAGACTTT
ATATTCTACGATTACAAGGCTCCTCTGTCGATTCCGAAGGAATGTAGGGAGAAGTTTGATCTTGTGTTTG
CAGACCCTCCTTTCCTCTCAGATGAGTGCCTCACCAAGACAGCTGTCACAATGAAGTACATGTGCAAGGA
TCCGAAGAAGCTGGTTCTCTGCTCTGGAGCTGTCATGGAGGAAATGGCTGGCCGGCTCCTTGACTTGAAA
ATATGTAAATACAAACCTCAGCACGAAAATAATCTTGCAAATGACTTTTGC
EST_crog_evm_803481 crog_evm Caligus rogercresseyi cDNA clone crog_evm_507_249_rev 5', mRNA
sequence.
Sequence ID: gb|FK895431.1|Length: 736Number of Matches: 8
99.9 bits(212) 2e-46 38/51(75%) 44/51(86%) 0/51(0%) +1/-2

```
Query  409  GSDFIFYDYKAPLSIPKECREKFDLVFADPPFLSDECLTKTAVTMKYMCKD  561
            GS F+FYDYK+PL IP+E R+ F LV ADPPFLSDECLTKTAVT+KY+ KD
Sbjct  387  GSKFVFYDYKSPLDIPRELRDDFSLVVADPPFLSDECLTKTAVTVKYLSKD  235
```

>gi|698728677|gb|JZ756141.1|JZ756141 awis5P0003G13_F.ab1 Sea ice eukaryotic metatranscriptome
Weddell Sea 2 uncultured organism cDNA, mRNA sequence
TCTAAAACGACCAAGCTAATATTGCTTGATTCACTTCTACAAGAGCATTCATTTCAGAGAAAAATGGGGA
ATGAGTCTGATGATGAGGAAGAACCTATAACCTTATCTTCAGACACATTTGCGGCCCTTTCCCAATTTTA
CAAAGAGCAAGATGAGAGGGAGAAAGTATGGGAGGAGGGAGGGGAGAGTGTGGACGTTTCTGTCCAGGTC

AAGGAAGATTGGCAGCTCTCCCAGTTCTGGTACAGCCAGCAGACGGCGGAAACACTCGCCAAAATGGTGA
TGAGCACTGTCGGGGAGGGGGGAAGTATCGCCTGTGTAAGCGCCCCCACCTTATACCGTGCTGTGAAATT
ACTGGACAAAGACTTTGCCGCCTCTATATTTGAATACGATAAACGGTTTGCAAGCTATGGTTCAGACTTT
ATATTCTACGATTACAAGGCTCCTCTGTCGATTCCGAAGGAATGTAGGGAGAAGTTTGATCTTGTGTTTG
CAGACCCTCCTTTCCTCTCAGATGAGTGCCTCACCAAGACAGCTGTCACAATGAAGTACATGTGCAAGGA
TCCGAAGAAGCTGGTTCTCTGCTCTGGAGCTGTCATGGAGGAAATGGCTGGCCGGCTCCTTGACTTNGAA
ATAT
EST_crog_evm_803481 crog_evm Caligus rogercresseyi cDNA clone crog_evm_507_249_rev 5', mRNA sequence.
Sequence ID: gb|FK895431.1|Length: 736Number of Matches: 7
99.7 bits(212) 9e-39 38/51(75%) 44/51(86%) 0/51(0%) +1/-2

```
Query  409  GSDFIFYDYKAPLSIPKECREKFDLVFADPPFLSDECLTKTAVTMKYMCKD  561
            GS F+FYDYK+PL IP+E R+ F LV ADPPFLSDECLTKTAVT+KY+ KD
Sbjct  387  GSKFVFYDYKSPLDIPRELRDDFSLVVADPPFLSDECLTKTAVTVKYLSKD  235
```

>gi|698728239|gb|JZ755703.1|JZ755703 awis5P0002A19_F.ab1 Sea ice eukaryotic metatranscriptome Weddell Sea 2 uncultured organism cDNA, mRNA sequence
TCTAAAACGACCAAGCTAATATTGCTTGATTCACTTCTACAAGAGACCGAAGAATGCCGATCTTCGTGAA
GACCCTGACGGGGAAGACCATCACCCTGGAGGTGGAGGCCAGCGACTCCATCGAGAATGTCAAGGCCAAG
ATCCAGGATAAGGAGGGAATCCCCCCAGATCAGCAGAGGTTGATCTTTGCTGGTAAGCAGCTGGAGGATG
GGCGGACTTTGTCCGACTACAACATCCAGAAGGAGTCTACCCTTCATTTGGTTCTGCGTCTTCGTGGTGG
TGTCATCGAGCCCTCCCTTCGTCTCCTGGCACAGAAGTACAACTGTGACAAGATGATTTGCAGGAAGTGC
TATGCCAGGCTCCATCCCAGGGCAACAAACTGCAGAAAGAAGGCTTGTGGTCATACCTCCCAGCTGAGAC
CCACGAAGAAGCTGAAGTAAACTGCAACATCAACAGCAGCAGCAACAGCAGCAACAACAACAACATCAGC
AACAACAACAACAATATTTTGTAGATCATGTCAAGACTCACATCGTACTTTACCGTACAATATCTGTTGA
TACAACTGTACACTTATATCTTCAAATATTTTGTGATGTCCAATCTGATCTACTTAAAATAAAACATTAT
AACATG
(>gi|283509595|gb|FN251249.1|FN251249 FN251249 dmp037 Tigriopus californicus cDNA clone dmp037P0006C16 5', mRNA sequence
GGCCTCACTTTGGATCCGTGAACTGGCTTCCAATAGAGCGGCCCAAGATGCAGATCTTCGTGAAGACCCT
CACGGGCAAAACCATCACCTTGGAGGTGGAGCCCTCGGATTCGATCGAAAATGTCAAGGCTAAAATCCAA
GACAAGGAAGGCATTCCCCCAGATCAGCAACGTCTGATCTTTGCTGGCAAGCAATTGGAGGATGGGCGAA
CCTTGTCCGACTACAACATCCAAAAGGAGTCCACCCTTCATCTGGTGCTTCGTCTTCGCGGTGGTGTGAT
TGAGCCTTCTCTCCGGATTCTGGCCCAAAAGTACAACGCCGACAAGATGATTTGCCGCAAGTGCTACACT
CGTCTCCATCCCAGAGCCAACAATTGCCGCAAGAAGTCCTGCGGACACACCTCCAACATCCGACCCAAGA
AGAAGCTGAAGTAAACGGAAATCTGGGTCTTGGCTTGGGGTGTATGTGTCGCTTAAGGGTATCCTTAATG
TAAGCCTGGTCAGGCTTGGGAGCTGAAATACATCTGGAATGG)
FN251249 dmp037 Tigriopus californicus cDNA clone dmp037P0006C16 5', mRNA sequence.
Sequence ID: emb|FN251249.1|Length: 532Number of Matches: 9
286 bits(618) 1e-76 119/130(92%) 123/130(94%) 0/130(0%) +3/+3

```
Query  51   RMPIFVKTLTGKTITLEVEASDSIENVKAKIQDKEGIPPDQQRLIFAGKQLEDGRTLSDY  230
            +M IFVKTLTGKTITLEVE SDSIENVKAKIQDKEGIPPDQQRLIFAGKQLEDGRTLSDY
Sbjct  45   KMQIFVKTLTGKTITLEVEPSDSIENVKAKIQDKEGIPPDQQRLIFAGKQLEDGRTLSDY  224

Query  231  NIQKESTLHLVLRLRGGVIEPSLRLLAQKYNCDKMICRKCYARLHPRATNCRKKACGHTS  410
            NIQKESTLHLVLRLRGGVIEPSLR+LAQKYN DKMICRKCY RLHPRA NCRKK+CGHTS
Sbjct  225  NIQKESTLHLVLRLRGGVIEPSLRILAQKYNADKMICRKCYTRLHPRANNCRKKSCGHTS  404

Query  411  QLRPTKKLK*  440
             +RP KKLK*
Sbjct  405  NIRPKKKLK*  434
```

>gi|698727961|gb|JZ755442.1|JZ755442 awis5P0001E02_F.ab1 Sea ice eukaryotic metatranscriptome Weddell Sea 2 uncultured organism cDNA, mRNA sequence

```
TCTAAAACGACCAAGCTAATATTGCTTGATTCACTTCTACAAGAGAAGTCAGAATGGCCAAGCGTACCAA
GAAGGTTGGAATCACTGGTAAGTATGGAACCAGATACGGAGCCTCCCTCCGTAAGACCGTCAAGAAGATG
GAGATATCTCAGCACAGCAAGTACGTCTGCACCTTCTGTGGCAAGGAGAACATGAAGAGGAAGGCTGTCG
GTATCTGGCAGTGTGGTACCAAGAACTGTCGGATCCGCGTGGCAGGAGGTGCCTGGAACTATACCACAAC
AGCCGCTGCATCAGTGAGGTCTGCCGTGAGAAGGTTGAGAGAATTAAAGGAGATGTAACTCCTCTCCTCT
CCTACCTCCTGCTGGACCTTCCCTCCTGCTGGACCCTCCTCCCTTCGTACTGATATCTTTATTAACATAT
TGGACCGTTCTGAATGTGGAATATATTCCAACATGAAATCCTTCTCTCGTAATTTGCGATGTACCATTGT
GTGATCTTAATATAATCTATGTGATTTATAAGAACAAAAATTTCACAAATTAAGTCTAACCAAGGGTTTA
CAAGCAAACAAGAGAAATAATCAACTTATTTCTATTTCAG
```

EST_lsaA_evv_1238322 lsaAevv mixed_tissue Lepeophtheirus salmonis (Atlantic Norway) cDNA Lepeophtheirus salmonis cDNA clone lsaA_evv_521_144 3’, mRNA sequence.

Sequence ID: gb|GW661359.1|Length: 390Number of Matches: 7

171 bits(369) 2e-42 65/74(88%) 71/74(95%) 0/74(0%) +3/-3

```
Query  51   RMAKRTKKVGITGKYGTRYGASLRKTVKKMEISQHSKYVCTFCGKENMKRKAVGIWQCGT  230
            +MAKRTKKVG+ GKYGTRYGASLRKTVKKMEI+QHSKYVCTFCGKENMKR+AVGIW+CGT
Sbjct  379  KMAKRTKKVGVVGKYGTRYGASLRKTVKKMEITQHSKYVCTFCGKENMKREAVGIWKCGT  200

Query  231  KNCRIRVAGGAWNY  272
            KNCR+ VAGGAW Y
Sbjct  199  KNCRVIVAGGAWTY  158
```

>gi|698735149|gb|JZ760213.1|JZ760213 awis5P0020B21_F.ab1 Sea ice eukaryotic metatranscriptome Weddell Sea 2 uncultured organism cDNA, mRNA sequence

```
TCTAAAACGACCAAGCTTTTATTGCTTGATTCACTTCTACAAGAGATAATCTTGAGAAGTTTTCACGATG
GTGAACGGCCGCGTCCCAATGAAGTACTCCAAGGTGTACACCACACCAAGGAGGCCTTTTGAGAAATCCA
GCCTTGACCAAGAGCTCAAACTCATCGGTCAATACGGTCTAAGGAATAAGAGGGAGGTATGGAGAGTTAA
GTACTCTTTGGCCAAGATCAGGGCCGCTGCCAGAGAATTACTGACTCTCGAAGAAAAAGACGCCAAGAGG
```

CTTTTCGAAGGTAATGCACTTCTGAGGCGTCTTGTTCGTACTGGTGTCCTTGGCGATGACCGTATGAAGT
TGGATTACGTTCTTGGCCTCAAGATCGAAGACTTCCTTGAGAGGAGGCTCCAGACCCAAGTCTTCAAGCT
CGGGTTAGCCAAGTCTATCCACCACGCCAGGATTTTGATCAAGCAAAAGCACATCCGAGTCCGGAAGCAG
GTTGTGAACATTCCTAGCTTCATTGTGAGGTTGGACAGCCAGAAACACATTGACTTCAGCCTCAAGTCAC
CCTTCGGAGGTGGCAGGCCCGGCCGTGTCAAGAGGAAGAAACGCCAGAAGGGCGATGACGCTGGAGCTAA
NGA
FN248535 dmp037 Tigriopus californicus cDNA clone dmp037P0027K17 5', mRNA sequence.
Sequence ID: emb|FN248535.1|Length: 659Number of Matches: 13
322 bits(697) 1e-87 145/167(87%) 158/167(94%) 0/167(0%) +2/+1

```
Query  65   TMVNGRVPMKYSKVYTTPRRPFEKSSLDQELKLIGQYGLRNKREVWRVKYSLAKIRAAAR  244
            TMVNGR+P  YSKVY TPRRPFEK+ LDQELKLIG YGLRNKREVWRVK++LAKIR AAR
Sbjct  22   TMVNGRLPTNYSKVYATPRRPFEKARLDQELKLIGDYGLRNKREVWRVKFALAKIRKAAR  201

Query  245  ELLTLEEKDAKRLFEGNAllrrlvrtgvlgDDRMKLDYVLGLKIEDFLERRLQTQVFKLG  424
            ELLTLEE+DAKRLFEGNALLRRLVR GVL + RMKLD+VLGLK+EDFLERRLQTQVFKLG
Sbjct  202  ELLTLEEEDAKRLFEGNALLRRLVRIGVLDESRMKLDFVLGLKVEDFLERRLQTQVFKLG  381

Query  425  LAKSIHHARILIKQKHIRVRKQVVNIPSFIVRLDSQKHIDFSLKSPF  565
            LAKSIHHAR+LI+Q+HIRVRKQVVN+PSF+VRLDSQKHIDFSLKSPF
Sbjct  382  LAKSIHHARVLIRQRHIRVRKQVVNVPSFVVRLDSQKHIDFSLKSPF  522
```
>gi|698728445|gb|JZ755909.1|JZ755909 awis5P0002K18_F.ab1 Sea ice eukaryotic metatranscriptome Weddell Sea 2 uncultured organism cDNA, mRNA sequence
TCTAAAACGACCAAGCTTTTATTGCTTGATTCACTTCTACAAGAGACTTCAGGATGGCCTTGCCTGCTCT
TGTTTACTGGGAAGACCCCATCCAGACTGGAGCTGTATTCGGCACAGTGTTGGTGGCCTTGGTCTCCCTC
TCCTCCTGCTCCCTCCCATCTGTCCTCTCCTATTTCTGTCTCTCTGCTCTTTCTCTCGTCCTTTTGGTCA
AGGCTTACAGCTTCGTCATGATCAAGGCTGGCAAGGCAGAGGCTGGTTCCGACCCTCTGGCTAAGGTGGC
CAACTTGCCCCTGGAGGTCCCTGCTGACAAAATCTCCTCGTTTGCTCCCTACATCTCCAGCTCTCTGAAT
GCCGCTCTCTCTGAACTTCGTCGTCTCTTTCTTGTTGAGAGCATGGTTGACACAATAAAGTTCGGTCTCT
CCCTCTGGATGCTCACCTACATCGGTTCCTGGTTCAATGCCCTCACCCTCCTCATCCTCATCTGGGTTGG
CTTCTTCACCCTGCCCAAGGTCTACATCCAAAACCAAGCCCAGTGTGATGAGGTCTTCAACAAGGTCATG
GCCCAGGTGGATGAAGTGAAAGCTAAAGTTGTAGCAATCATCCCCATGAAAGGAGCAGTGAAGGAAGAGT
AAAAAGTGGCTGCTT
EST_crog_evm_832585 crog_evm Caligus rogercresseyi cDNA clone crog_evm_514_169_rev 5', mRNA sequence.
Sequence ID: gb|FK900246.1|Length: 779Number of Matches: 2
166 bits(356) 1e-40 64/116(55%) 89/116(76%) 0/116(0%) +3/-3

```
Query  258  SDPLAKVANLPLEVPADKISSFAPYISSSLNAALSELRRLFLVESMVDTIKFGLSLWMLT  437
            S+PL K + + L V +DK+ S A  ++   NAA+SELRRLFLVE M D++KFGL LW+LT
Sbjct  747  SNPLLKYSGVDLTVSSDKVISIAKNMTDKFNAAVSELRRLFLVECMADSVKFGLGLWVLT  568
```

Query 438 YIGSWFNALTLLILIWVGFFTLPKVYIQNQAQCDEVFNKVMAQVDEVKAKVVAIIP 605
Y+GSWFN +TL+IL WVG F++PK+Y+ N+A D + N+V+A+V+EVK+KV IP
Sbjct 567 YVGSWFNMMTLIILAWVGLFSIPKIYVNNKAAIDPILNQVLAKVNEVKSKVETAIP 400

>gi|698728233|gb|JZ755697.1|JZ755697 awis5P0002A11_F.ab1 Sea ice eukaryotic metatranscriptome Weddell Sea 2 uncultured organism cDNA, mRNA sequence
TCTAAAACGACCAAGCTTTTATTGCTTGATTCACTTCTACAAGAGAGTTGCTAACAGAGCATCAAAATGG
CTGACGGTTGTAAACTGTTCGTTTATGGCGTCGATCAAAACCTCTCAAATGAAGAAATTCAGTCCGAGTT
CGGTAAATTTGGCAACGTTACCGATGTCTACAACACCGGAAAAGGCTCTGCCTTCGTAACTTTCGAGAGC
AAGGAAGAGGCTGATACAGCTACACGGGAAATGGATGGATCTACCATCAATGGCCAGCAGATCAAGGTCA
ACGAAGCCCGTCCTCGTGATGGAGGTGGTGGTGGACGTGGTGGTGGCGGACGTGGAGGTGGCCGTGGAGG
CGGTGGTGGCGGATATGGAGGTGGTGGCGGCAGTTACGGAGGTGGAGGCTATGGCGGCGATGGTGGTGGC
GGCGGATATGGAGGTCGTGGTGGTGGAGGAGGCGGATACGGTGGTCGTGGAGGCGGAGGCTATGATGGCG
GCCGTGGAAGATGTGGAGGCGGCGGATATGGCG
CF_WO0_02f05_SP6 Copepod Whole Organism, Normalized Calanus finmarchicus cDNA clone CF_WO0_02f05 5’ similar to gb|AAQ94565.1| RNA binding motif protein - Danio rerio. Score = 61.6 bits (148), Expect = 2e-08, mRNA sequence.
Sequence ID: gb|EH666423.1|Length: 548Number of Matches: 4
121 bits(259) 3e-27 47/76(62%) 64/76(84%) 0/76(0%) +1/+1

Query 73 DGCKLFVYGVDQNLSNEEIQSEFGKFGNVTDVYNTGKGSAFVTFESKEEADTATREMDGS 252
+G KLFVYGV++ SN E+Q+EF KFG VTD YNTGKG AFVTF++K++A+ AT +++G+
Sbjct 142 EGGKLFVYGVNEGTSNSELQTEFEKFGTVTDTYNTGKGYAFVTFDNKDDANEATEQLNGA 321

Query 253 TINGQQIKVNEARPRD 300
TI GQQ+KVN ARP++
Sbjct 322 TILGQQVKVNVARPKN 369

>gi|698727198|gb|JZ752877.1|JZ752877 awig5P0012L04_F.ab1 Sea ice eukaryotic metatranscriptome Weddell Sea 1 uncultured organism cDNA, mRNA sequence
TCTAAAACGACCAAGCTTTTATTGCTTGATTCACTTCTACAAGAGAGGTGACTAAAAAGTCGCCATGGGG
AAGATTATGAAGAGCGGGAAGGTTGTCCTGCTGCTCGCTGGCAGATATGCTGGTCGCAAGGCAGTCATTG
TGAAGCCTTCGGACGAGGGTACAACTGATAAGCCATACAGTCATGCTCTTGTTGCTGGCATCGATAGGTA
CCCCCGTAAGGTCACCAAGAAGATGTCCAAGAAGAAGATCCAGAAAAGGTCCAAGGTCAAGCCTTTCCTC
AAGGTGATTAATTATAACCACGTTATGCCTACTCGTTATTCTGTGGACATCAGCTTCGACAAGACTAACA
TCAACAAGGAGCTTCTGAAGGATCCCATGAAGAAGAAAAAGGCCAGGAACATGGTGAGAACAAAGTTCGA
GGAGCGTTACAAGAGCGGAAAGAACAGGTGGTTCTTCCAGAAGCTGAAGTTCTAAGCTGGGGATTTGATG
CTGTTAGCAAGCACCTTTTCTATCTCCTGTTTCGAACCCCTTGAGCCCTATGTTGTCTACACAGCTTTGA
CTAANGACTCCATATTTCAG
FN250765 dmp037 Tigriopus californicus cDNA clone dmp037P0027L24 5’, mRNA sequence.

Sequence ID: emb|FN250765.1|Length: 457Number of Matches: 8
121 bits(258) 4e-52 58/69(84%) 66/69(95%) 0/69(0%) +2/+2

```
Query  272  PFLKVINYNHVMPTRYSVDISFDKTNINkellkdpmkkkkARNMVRTKFEERYKSGKNRW  451
            PFLKV+NYNH+MPTRYSV++ FDK N+NKE LKDPMKKKKARNMVRT+FEERYKSGKNRW
Sbjct  206  PFLKVVNYNHLMPTRYSVELGFDKANLNKETLKDPMKKKKARNMVRTQFEERYKSGKNRW  385

Query  452  FFQKLKF*A  478
            FFQKL+F*+
Sbjct  386  FFQKLRF*S  412
```

>gi|698733722|gb|JZ758795.1|JZ758795 awis5P0013L03_F.ab1 Sea ice eukaryotic metatranscriptome Weddell Sea 2 uncultured organism cDNA, mRNA sequence
TCTAAAACGACCAAGCTTTTATTGCTTGACTCACTTCTACAAGAGAATCACAAGCAAAAAGAGTTCAGGA
TGACTGCCACTATCGACAGCTTCTTCAACACCTTCAACACTAAGTTCGAGGCTCCAGTGCGTCACCACCT
GAAGAATGTTTACGCCTCCTTGGCTATGTCTGTGCTCGCAGCTGCTGCTGGAGCCTATACTCACCTCTTC
ACCTCTCTTCTGCAGGGAGGTGGAATCCTTTTCTCCCTCGCCGGAGTTGGACTGGCTCTGGGTCTCTTCA
TGACCCCAGACAACGGCAAGAACCGAGGTCAGCGCCTCTCCATGCTTCTTGGATTCGCCTTTCTTACTGG
CCTCGGCCTGGGACCCCTTCTTGGCATGGCAATGCAAATGAACCCCACCATTGTTCCAACTGCCCTCGCC
ACAACCTCTGCTATCTTTGCCTGCTTCTCCGCTGCCGCTCTCTTTGCCCCAGATGGAAAATACCTCTACC
TCGGTGGTAGCCTCCTCTCTGGGCTCACTACAATCACTATCCTTGGCCTCCTCAACATCTTCTTCAAGTC
CCAGCTTCTCTTCCAGGCTTACGTCTACCTCAGCCTGGCCTTATTCTGTGGCTTTGTCGTGTTTGACACC
CAAGTGATCATCAGGAAGGCTAGGAATGGCGACAGGGACTTCATCGCCCACTCCCCTCGACTCTTCATCG
ACTTCGTCC
LNC1809-2008-01-14.ab1 Lepeophtheirus salmonis LNC Lepeophtheirus salmonis cDNA, mRNA sequence.
Sequence ID: gb|HO677847.1|Length: 839Number of Matches: 7
76.7 bits(161) 8e-34 32/53(60%) 39/53(73%) 0/53(0%) +1/+2

```
Query  550  FFKSQLLFQAYVYLSLALFCGFVVFDTQVIIRKARNGDRDFIAHSPRLFIDFV  708
            F  S L+F+  ++  LA+FCGFVVFDTQ II+K   GD+DFI HS  LFIDFV
Sbjct  545  FAHSYLIFKIQLWAGLAVFCGFVVFDTQNIIQKCEAGDKDFIRHSVDLFIDFV  703
```

>gi|66380571|gb|BW778077.1|BW778077 BW778077 Amphioxus Branchiostoma floridae unpublished cDNA library, gastrula whole animal Branchiostoma floridae cDNA clone bfga048h24 5', mRNA sequence
CAAGCTAATATTGCTTGATTCACTTCTACAAGAGAGGTAGAAAAGAAAGGCTCCATCATGCTGATGCCCA
AGAAGAACAGAGTTGCCATCTACGAACACTTGTTCAAGGAGGGAGTTCTCACCGCTGAGAAGAACTTCAA
TGCTCCCAAGCATACTGAGCTGACTGAGATCCCCAACCTCCAGGTCATCAAGGCTCTGACTTCTTTGAAG
TCTCGTGGTTATGTGAAGGAACAGTTTGCCTGGAGACATTACTACTGGAGCCTGACCAACGAGGGTATCC
AGTACCTGCGTGACTTCCTCCACCTGCCCCCAGAGATCGTGNCCGCC

EST_lbra_evt_1105878 lbraevt mixed_tissue Lernaeocera branchialis cDNA Lernaeocera branchialis cDNA clone lbra_evt_010_246 3', mRNA sequence.
Sequence ID: gb|GO418367.1|Length: 683Number of Matches: 9

115 bits(246) 1e-38 44/52(85%) 48/52(92%) 0/52(0%) +1/-3

```
Query  166  EIPNLQVIKALTSLKSRGYVKEQFAWRHYYWSLTNEGIQYLRDFLHLPPEIV  321
            ++PNL V+KAL SLKSRGYVKEQFAWRHYYW LTNEGIQYLRD+LHLP EIV
Sbjct  552  DVPNLHVMKALNSLKSRGYVKEQFAWRHYYWYLTNEGIQYLRDYLHLPSEIV  397
```

Range 2: 553 to 657GenBankGraphics Next Match Previous Match First Match

62.5 bits(130) 1e-38 25/35(71%) 29/35(82%) 0/35(0%) +1/-3

```
Query  58   MLMPKKNRVAIYEHLFKEGVLTAEKNFNAPKHTEL  162
            MLM KKN V IYEHLF EGV+ A+K+ +APKHTEL
Sbjct  657  MLMSKKNLVMIYEHLFNEGVMVAKKDVHAPKHTEL  553
```

>gi|66379963|gb|BW777469.1|BW777469 BW777469 Amphioxus Branchiostoma floridae unpublished cDNA library, gastrula whole animal Branchiostoma floridae cDNA clone bfga045p12 5', mRNA sequence

CAAGCTAATATTGCTTGATTCACTTCTACAAGAGAAGCGTGTCGTATTCTTTTAAAACTTAACCATGGGC
AAGGAAAAGACTCATATCAACATTGTTGTCATTGGACACGTCGACTCCGGCAAGTCCACCACCACTGGTC
ACTTGATCTACCAGTGCGGAGGTATTGACAAGAGAACCATCGAGAAGTTTGAGAAGGAGTCCTCGGAATC
TGGCAAGGGATCCTTCAAGTACGCCTGGGTCTTGGACAAGCTGAAGGCTGAGCGTGAGAGAGGTATCACC
ATTGATATTGCTCTGTGGAAGTTTGAGACTCCCAAGTACTATGTGACCATCATTGATGCTCCTGGACATC
GTGACTTCATCAAGAACATGATCACTGGTACATCCCAGGCTGACTGCGGAGTCTTGATCATTGCCTCTGG
AACCGGTGAGTTTGAGGCTGGCATCTCCAGGAACGGTCAGA

EST_lsal_pm_787651 lsalpm whole Lepeophtheirus salmonis (Pacific) cDNA Lepeophtheirus salmonis cDNA clone lsal_pm_3_253_rev 5', mRNA sequence.
Sequence ID: gb|EY509796.1|Length: 931Number of Matches: 11
185 bits(398) 5e-69 Identities 75/77(97%), positive 77/77(100%), gap 0/77(0%) +2/+3

```
Query  230  YAWVLDKLKAERERGITIDIALWKFETPKYYVTIIDAPGHRDFIKNMITGTSQADCGVLI  409
            YAWVLDKLKAERERGITIDIALWKFETPK+YVTIIDAPGHRDFIKNMITGTSQADCGVLI
Sbjct  234  YAWVLDKLKAERERGITIDIALWKFETPKFYVTIIDAPGHRDFIKNMITGTSQADCGVLI  413

Query  410  IASGTGEFEAGISRNGQ  460
            IASGTGEFEAGIS+NGQ
Sbjct  414  IASGTGEFEAGISKNGQ  464
```

>gi|66384505|gb|BW782010.1|BW782010 BW782010 Amphioxus Branchiostoma floridae unpublished cDNA library, gastrula whole animal Branchiostoma floridae cDNA clone bfga040j16 5', mRNA sequence

AAGCTAATATTGCTTGATTCACTTCTACAAGAGAAGTCCACAACATGGCTGTAGGCAAGAACAAGGGCCT
CAAGATTGGAGGCAAGAAGGGTGTCAAGAAGAAGGTCGTCGATCCTTTCACCAGGAAGGACTGGTATGAC
ATCAAGGCCCCCAACACCTTCAAAGTGAGACAGGTTGGCAAGACCTTGGTCAACAGGACTGCTGGTACCA
AGATTGCTTCTGAGGGACTCAAGGGCCGCGTCTTCGAGGTCTCCCTTGCTGATCTGCAGAAGGAGGAAAA
TGCTGAGAGATCCTTCAGAAAGTTCAAGCTGATCGTTGAGGATGTGCAGGGCAAGAGCTGCTTGACCACC
TTCCACGGCATGAACCTGACCACCGACAAGCTGAGGTCCATGGTCAAGAAGTGGCAAACCCTGATTGAGG
GCAACGTTGATGTCAAGACCACTGACGGCTACATGCTCCGTCTCTTCTGCATCGGCTTCACCAAGAAGAA
CGACCTGTCCACCAAGAAGACTTGCTACGCCCAGTCTGCCCAGGTCAAGAACATCCGCAAGAAGATGACT
GACATCATCACCANAGAGGTCTCCAAGACCGACTTGAAGGGAGTTGTCAACAANCTTATCCCTGATTCTA
TTGCAACAGACATTGCTACAGCTTGTCAAGGAATCTATCCTCTTCACGATGTGCACATCANGA

EST_lsal_evj_942247 lsalevj mixed_tissue_mixed_stage Lepeophtheirus salmonis (Pacific) cDNA Lepeophtheirus salmonis cDNA clone lsal_evj_545_199_fwd 5', mRNA sequence.

Sequence ID: gb|FK926144.1|Length: 911Number of Matches: 12

Range 1: 90 to 485GenBankGraphics Next Match Previous Match

257 bits(555) 2e-93 102/132(77%) 117/132(88%) 0/132(0%) +3/+3

```
Query  111  DPFTRKDWYDIKAPNTFKVRQVGKTLVNRTAGTKIASEGLKGRVFEVSLADLQKEENAER  290
            DPFTRKDWYD+KAP  FKVR+VGKTLVNRT GT+IAS+GLKGRV+EVSLADLQ E +AER
Sbjct  90   DPFTRKDWYDVKAPTIFKVREVGKTLVNRTQGTRIASDGLKGRVYEVSLADLQSETDAER  269

Query  291  SFRKFKLIVEDVQGKSCLTTFHGMNLTTDKLRSMVKKWQTLIEGNVDVKTTDGYMLRLFC  470
            SF+KFKLI EDVQG++CLT FHGMNLTTDKLRS+VKKWQTLIE + DVKTTDGY++R FC
Sbjct  270  SFKKFKLICEDVQGRNCLTNFHGMNLTTDKLRSVVKKWQTLIEAHADVKTTDGYLIRAFC  449

Query  471  IGFTKKNDLSTK  506
            IGFT +   + K
Sbjct  450  IGFTSRQSPNQK  485
```

Range 2: 480 to 665GenBankGraphics Next Match Previous Match First Match

Alignment statistics for match #2

102 bits(217) 2e-93 40/62(65%) 49/62(79%) 0/62(0%) +3/+3

```
Query  504  KKTCYAQSAQVKNIRKKMTDIITXEVSKTDLKGVVNXLIPDSIATDIATACQGIYPLHDV  683
            +K CYAQS Q++ IRKKM DI+T E+S  DLK VV  LIPDS+A D+  AC+GIYPLHDV
Sbjct  480  QKACYAQSQQIRQIRKKMVDIMTREISSCDLKEVVKKLIPDSMAVDVQRACRGIYPLHDV  659
```

Query 684 HI 689
+I
Sbjct 660 YI 665
>gi|698725579|gb|JZ752592.1|JZ752592 awig5P0011N19_F.ab1 Sea ice eukaryotic metatranscriptome Weddell Sea 1 uncultured organism cDNA, mRNA sequence
TCTAAAACGACCAAGCTTTTATTGCCTGATTCACTTCTACAAGAGATAATCTTGAGAAGTTTTCACGATG
GTGAACGGCCGCGTCCCAATGAAGTACTCCAAGGTGTACACCACACCAAGGAGGCCTTTTGAGAAATCCA
GCCTTGACCAGGAGCTCGAACTCATCGGTCAATACGGTCTAAGGAACAAGAGGGAGGTATGGAGAGTTAA
GTACTCTTTGGCCAAGATCAGGGCTGCTGCCAGAGAATTGCTCACTCTTGAGGAAAAGGATGCTAAGAGG
CTTTTCGAAGGTAATGCACTTCTGAAGCGTCTTGTTCGCACTGGTGTCCTTGGCGATGACCGTATGAAGT
TGGATTACGTTCTTGGTCTCAAGATCGAAGACTTCCTTGAGAGGAGGCTCCAGACCCAAGTCTTCAAGCT
CGGGTTAGCCAAGTCTATCCATCACGCCAGGATTTTGATCAAGCAAAAGCACATCCGAGTCCGGAAGTAG
GTTGTGAACATCCCTAGCTTCATTGTGAGGTTGGACAGCCAGAAACATATTGACTTCAGCCTTAAGTCAC
CCTTCGGAGGTGGTAGGCCCGGCCGTGTCAAGAGGAAGAACGCCAAGAAGGGCGATGACGCTGGAGCTAA
GGAAGACGAGGATGAAGAGTAGAATGGACCCTTCTATGACTTTGTCTGTAATCCTGACCTTACTTGTGCT
ACATTTAGCCA
FN248535 dmp037 Tigriopus californicus cDNA clone dmp037P0027K17 5', mRNA sequence.
Sequence ID: emb|FN248535.1|Length: 659Number of Matches: 11
365 bits(791) 2e-100 152/177(86%) 167/177(94%) 0/177(0%) +2/+1

Query 65 TMVNGRVPMKYSKVYTTPRRPFEKSSLDQELELIGQYGLRNKREVWRVKYSLAKIRAAAR 244
TMVNGR+P YSKVY TPRRPFEK+ LDQEL+LIG YGLRNKREVWRVK++LAKIR AAR
Sbjct 22 TMVNGRLPTNYSKVYATPRRPFEKARLDQELKLIGDYGLRNKREVWRVKFALAKIRKAAR 201

Query 245 ELLTLEEKDAKRLFEGNALLKRLVRTGVLGDDRMKLDYVLGLKIEDFLERRLQTQVFKLG 424
ELLTLEE+DAKRLFEGNALL+RLVR GVL + RMKLD+VLGLK+EDFLERRLQTQVFKLG
Sbjct 202 ELLTLEEEDAKRLFEGNALLRRLVRIGVLDESRMKLDFVLGLKVEDFLERRLQTQVFKLG 381

Query 425 LAKSIHHARILIKQKHIRVRK*VVNIPSFIVRLDSQKHIDFSLKSPFGGGRPGRVKR 595
LAKSIHHAR+LI+Q+HIRVRK VVN+PSF+VRLDSQKHIDFSLKSPFGGGRPGRVKR
Sbjct 382 LAKSIHHARVLIRQRHIRVRKQVVNVPSFVVRLDSQKHIDFSLKSPFGGGRPGRVKR 552

>gi|169560353|gb|FE575842.1|FE575842 CAXF9244.fwd Amphioxus Branchiostoma floridae unpublished cDNA library CAXF, gastrula whole animal Branchiostoma floridae cDNA clone CAXF9244 5', mRNA sequence
AAGCTAATATTGCTTGATTCACTTCTACAAGAGAAAGATGTCTCTCGCAGCGGCCCGTCCGTTGATCAGT
GTCTACGATGACAAGAACAAGAGCACCGGTGTGACCGTGTGTCTCCCAGCCGTGTTCCGCGCCCCTGTCA
GGCCCGACATTGTCTCCACCATCCACAATGAGGTGGCCAAGAACAAGCGCCAGCCCTACTGCGTCAACCA
GTATGCTGGACACCAGACCAGTGCTGAGTCCTGGGGAACTGGACGTGCTGTGGCCAGGATCCCAAGGGTC
AGGGGAGGTGGTACCCACAGATCTGGCCAGGGAGCTTTCGGTAACATGTGCCCGCGGTGGAAGGATGTTTG
CCCCCACCAAGACCTGGAGAAGGTGGCACAGGAAGGTCAATGTTGCCCAGAAGAGGTACGCCATGTGCTC

CGCCATTGCCGCCTCCGGTGTTCCCGCTCTTGTCATGGCTAAGGGACACGTGATCAATGGCATCAATGAG
GTGCCTCTCGTTGTGTCAGACAAGGTTCAAGGCTTCCAGAAGACCAAGGAGGTTGTTGAGTTCTTGAGAC
GCAACAAGGCCTGGGCTGATGTTGCCAAGGTGTACGCTACCAGGAGAATGAGGGCTGGCAAGGGTAAGCT
GAGGAACAGGAGGCATGTGCAGAAGAGGGGACCTCTGGTTGTCTACGACCAGGACCAGGGACTGACCAAG
GCCTTCAGGAACATCCCCGGCGTGGACACCATCCAGGTGGACAACCTCAACCTGCTCAAGCTGGCTCCCG
GTGGTCATGTTGGCAGATTCTGCATCTGGACTGAGGGTGCC
EST_lsal_evj_936983 lsalevj mixed_tissue_mixed_stage Lepeophtheirus salmonis (Pacific) cDNA
Lepeophtheirus salmonis cDNA clone lsal_evj_553_311_fwd 5', mRNA sequence.
Sequence ID: gb|FK932307.1|Length: 916Number of Matches: 14
Range 1: 73 to 846GenBankGraphics Next Match Previous Match
490 bits(1064) 4e-138 196/258(76%) 226/258(87%) 0/258(0%) +2/+1

```
Query  38   MSLAAARPLISVYDDKNKSTGVTVCLPAVFRAPVRPDIVSTIHNEVAKNKRQPYCVNQYA  217
            MSLA+ARPL++V  +K    G TV LPAVF+AP+RPD+VS IH+EVAKN+RQPY VNQ A
Sbjct  73   MSLASARPLVTVRSEKGLPAGSTVTLPAVFKAPIRPDVVSFIHHEVAKNRRQPYAVNQDA  252

Query  218  GHQTSAESWGTGRAVARIPRVRGGGTHRSGQGAFGNMCRGGRMFAPTKTWRRWHRKVNVA  397
            GHQTSAESWGTGRAVARIPRVRGGGTHRSGQGAFGNMCRGG MFAPTKT+RRWHRKVNVA
Sbjct  253  GHQTSAESWGTGRAVARIPRVRGGGTHRSGQGAFGNMCRGGHMFAPTKTFRRWHRKVNVA  432

Query  398  QKRYAMCSAIAASGVPALVMAKGHVINGINEVPLVVSDKVQGFQKTKEVVEFLRRNKAWA  577
            QKRYAMCSAIA+SGVPALVMAKGH I  ++EVPLVV DKV+ + KTK+ +  L R KAW
Sbjct  433  QKRYAMCSAIASSGVPALVMAKGHQIQDVHEVPLVVQDKVESYTKTKDAMILLHRLKAWK  612

Query  578  DVAKVYATRRMRAGKGKLRNRRHVQKRGPLVVYDQDQGLTKAFRNIPGVDTIQVDNLNLL  757
            D+ +VY ++RMR GKGK+RNRR +QK GPL+VY++D+G+ +AFRNIPG DTIQV+NLNLL
Sbjct  613  DIDQVYKSKRMRPGKGKMRNRRRIQKLGPLIVYNKDEGVRRAFRNIPGCDTIQVENLNLL  792

Query  758  KLAPGGHVGRFCIWTEGA  811
            KLAPGGHVGRFCIWTE A
Sbjct  793  KLAPGGHVGRFCIWTESA  846
```

>gi|169549926|gb|FE564053.1|FE564053 CAXF12510.fwd Amphioxus Branchiostoma floridae unpublished cDNA library CAXF, gastrula whole animal Branchiostoma floridae cDNA clone CAXF12510 5', mRNA sequence
AAGCTAATATTGCTTGATTCACTTCTACAAGAGACAGGACAACACTAAGGATGTTCGCCCAGATTGCCAG
ACGTGCTGCTCTCTCCACCAGGAACTTCAGCACCTCCGTAGCAAGGAGAGGAGGAGATGGACTCCCTCCT
CCAGGAGACAATCTCCCATTCTCCATCAACAACAGATACAAGTTGACTGTCTACTTCATCCTCTTCTTCG
GCAGTGGACTGTCCATTCCCTTCATTGCCATGAGACATCAGCTCCTCAAGAAATAGACCTCTTACTAATC
TGGACACTATCTTTAGTCTTGCATAGAATCTTGTAGTGTGATATGGATCTATGCTCTATCCTTTGTGTAA
TTATAAAATGGTAAATATGATGAATATTTCAGAAAAAAAAAAAAAAAAAAAAAA

FN251659 dmp037 Tigriopus californicus cDNA clone dmp037P0022M20 5', mRNA sequence.
Sequence ID: emb|FN251659.1|Length: 430Number of Matches: 2
58.3 bits(121) 2e-08 36/63(57%) 42/63(66%) 0/63(0%) +3/+1

```
Query  72   RAALSTRNFSTSVARRggdglpppgdnlpFSINNRYKLTVYFILFFGSGLSIPFIAMRHQ  251
            R   + R F+TS  RRG DG  PPGDNLPFSI NR+ LT   I+FFGS   +PF  +RHQ
Sbjct  91   RQVSNLRRFTTSAIRRGLDGPQPPGDNLPFSIENRFALTAKMIVFFGSAFGLPFFLVRHQ  270

Query  252  LLK  260
            L K
Sbjct  271  LTK  279
```

>gi|169549925|gb|FE564052.1|FE564052 CAXF12510.rev Amphioxus Branchiostoma floridae unpublished cDNA library CAXF, gastrula whole animal Branchiostoma floridae cDNA clone CAXF12510 3', mRNA sequence

```
CGGAAATATTCATCATATTTACCATTTTATAATTACCCAAAGGATAGAGCATAGATCCATATCACACTAC
AAGATTCTATGCAAGACTAAAGATAGTGTCCAGATTAGTAAGAGGTCTATTTCTTGAGGAGCTGATGTCT
CATGGCAATGAAGGGAATGGACAGTCCACTGCCGAAGAAGAGGATGAAGTAGACAGTCAACTTGTATCTG
TTGTTGATGGAGAATGGGAGATTGTCTCCTGGAGGAGGGAGTCCATCTCCTCCTCTCCTTGCAACGGAGG
TGCTGAAGTTCCTGGTGGAGAGAGCAGCACGTCTGGCAATCTGGGCGAACATCCTTAGGGTTGTCCTGTC
TCTTGTAGAAGTGAATCAAGCAATATTAGCTT
```

EST_crog_evm_834404 crog_evm Caligus rogercresseyi cDNA clone crog_evm_511_068_rev 5', mRNA sequence.
Sequence ID: gb|FK897933.1|Length: 313Number of Matches: 2

Related Information

Range 1: 99 to 200GenBankGraphics Next Match Previous Match
52.8 bits(109) 8e-07 21/34(62%) 25/34(73%) 0/34(0%) -3/-3

```
Query  224  FSINNRYKLTVYFILFFGSGLSIPFIAMRHQLLK  123
            F I NRY LT  FILFFGS  S+PF+A++ QL K
Sbjct  200  FKIENRYALTAKFILFFGSAFSLPFVAVKLQLCK  99
```

Range 2: 99 to 284GenBankGraphics Next Match Previous Match First Match
37.3 bits(75) 0.038 22/62(35%) 33/62(53%) 0/62(0%) +3/+3

```
Query  123  LEELMSHGNEGNGQSTAEEEDEVDSQLVSVVDGEWEIVSWRRESISSSPCNGGAEVPGGE  302
            L EL   GNEG  +   +EEE+E+  + +SV++ + +IV+    S   S   GG      E
```

Sbjct 99 LAELELDGNEGE*EGGSEEENELGREGISVLNLKGKIVAGGNASSFPSESRGGELTGNVE 278

Query 303 SS 308
SS
Sbjct 279 SS 284

>gi|66457308|gb|BW849092.1|BW849092 BW849092 Amphioxus Branchiostoma floridae unpublished cDNA library, neurula whole animal Branchiostoma floridae cDNA clone bfne035p19 5', mRNA sequence

GCTAATATTGCTTGATTCACTTCTACAAGAGATAAAAAAATGCGCGTGGAAAAGTGCTACTTCTGTGGCT
CCAGCTGTTATCCTGGCCATGGATTGACATTTGTCAGAAATGACTGCAAGATTTTCAAATTTTGCAGAAG
TAAATGCCACAGGTCCTTCAAGCTGAAGAGAAATCCCAGGAAGACCAAGCACACCAAGGCCTACAGAAAA
GCTGCAGGCAAAGAATTGGCGGTTGATCCCACATTCGAATTCGAGAAGAAACGCAATATTCCCGTCAAGT
ACGACAGAGAACTCTGGGCCAAGACCATCGAGGCAATGAAGAAGATTGAGGTGATCAAACAGAAACGCCA
ACAACACTTCTACGCACAGAGAAGAAAGCTGGCCGATAA

FN251067 dmp037 Tigriopus californicus cDNA clone dmp037P0001022 5', mRNA sequence.
Sequence ID: emb|FN251067.1|Length: 726Number of Matches: 7
210 bits(452) 4e-54 81/114(71%) 94/114(82%) 0/114(0%) +1/+3

Query 37 KMRVEKCYFCGSSCYPGHGLTFVRNDCKIFKFCRSKCHRSFKLKRNPRKTKHTKAYRKAA 216
KMR++KC+FCGS YPGHG FVRNDCKIFKFCRSKCH++FK K+NPRK K TKA+RK+A
Sbjct 60 KMRIDKCFFCGSPMYPGHGTHFVRNDCKIFKFCRSKCHKNFKKKKNPRKAKWTKAFRKSA 239

Query 217 GKELAVDPTFEFEKKRNIPVKYDRELWAKTIEAMKKIEVIKQKRQQHFYAQRRK 378
GKELAVDP+FEFEK+RN PVKYDR LW +TI A+K+IE IK KRQ R K
Sbjct 240 GKELAVDPSFEFEKRRNEPVKYDRALWTETITAVKRIEEIKNKRQAKHILDRLK 401

>gi|66422078|gb|BW813862.1|BW813862 BW813862 Amphioxus Branchiostoma floridae unpublished cDNA library, larva whole animal Branchiostoma floridae cDNA clone bflv057d24 5', mRNA sequence

TAATATTGCTTGATTCACTTCTACAAGAGATGAAAATGGCCGTTGCTTCCAGAGCCCTTCGCCAGCTAAT
CTCCTCCAGGTCATTTGTGACCTCTAGTTCCGCTGGCCAGGTTGCTCCAGGCGTCAAGACTTCAGGCAGT
GCACCCACACCCACCAGCGACGGAGGTCTTAACTTCACTATTACAGCAGAACAGCAAGAGTATTTAGACT
TGGCAGAGAATTTCACCAAAAATGAGATTATCCCTGTAGCTGCTCACTATGATGCTACTGGGGAGTTCCC
TTGGGCAGTCATAAAGAAGGCTCATGAGACAGGTCTTATGAATTTACACATCCCTGAGAAGTATGGAGGT
ATGGGCCTGGGAACTCTAGATGGTTGTCTCATCACTGA

CF_WO0_23h12_SP6 Copepod Whole Organism, Normalized Calanus finmarchicus cDNA clone CF_WO0_23h12 5' similar to XP_970506.1 CG12262-PA [Tribolium castaneum]. Score=220.32, Expect=4.70E-56, mRNA sequence.
Sequence ID: gb|EL586583.1|Length: 673Number of Matches: 10
188 bits(408) 1e-49 75/109(69%) 86/109(78%) 0/109(0%) +3/+1

Query 60   RQLISSRSFVTSSSAGQVAPGVKTSGSAPTPTSDGGLNFTITAEQQEYLDLAENFTKNEI 239
            R   + RS +SS+ Q APG KT+   P+   GGLNFT+T EQQEYLDLAE FTKNEI
Sbjct 94   RTCFTVRSISSSSTNNQTAPGAKTTSGEPSQDLPGGLNFTVTPEQQEYLDLAEQFTKNEI 273

Query 240 IPVAAHYDATGEFPWAVIKKAHETGLMNLHIPEKYGGMGLGTLDGCLIT 386
            IP AAHYD TGE+PW ++ KAHETGLMNLHIPE+YGGMGLGTLDGC+IT
Sbjct 274 IPNAAHYDLTGEYPWPILNKAHETGLMNLHIPEEYGGMGLGTLDGCMIT 420

>gi|66418126|gb|BW809910.1|BW809910 BW809910 Amphioxus Branchiostoma floridae unpublished cDNA library, larva whole animal Branchiostoma floridae cDNA clone bflv045p11 5', mRNA sequence

CTAATATTGCTTGATTCACTTCTACAAGAGACATCAGAATGTTGAGTAGCAAATTGACCGCGGCACTGGC
CCCCCACCTCGGCAGGCGGTGCTTCAGCTACGCCGGCGGCAGAGTCCCGCCGACCACTCAGAACGTGATC
GGCGGCGAATGGCAGGAGTCCAAGACAGACAGATGGATTGATGTGCATAACCCCGCCACAAATGAGGTCG
TCACAAGGGTCCCCCAATCAACCAACGACGAGAGAACGCCGCCGTGGCGTCCGCCAAGGAAGCCTACAAG
ACCTGGAGCAAAACTACTCCTCTCACCAGACAACAGATTATGCTCAAGTATCAGCAACTGATC

CF_WO1_19C01_SP6 Copepod Whole Organism, Normalized Calanus finmarchicus cDNA clone CF_WO1_19C01 5' similar to ref|XP_001375431.1| PREDICTED: similar to methylmalonate-semialdehyde dehydrogenase. Score = 266 bits (680), Expect = 9e-70, mRNA sequence.

Sequence ID: gb|FK868245.1|Length: 661Number of Matches: 13

115 bits(245) 7e-39 46/68(68%) 52/68(76%) 0/68(0%) +3/+2

Query 39   MLSSKLTAALAPHLGRRCFSYAGGRVPPTTQNVIGGEWQESKTDRWIDVHNPATNEVVTR 218
            MLS   ++ + G R SYAGGR+PPTTQNVI G+W ES TDRWIDVHNPATN+VVTR
Sbjct 29   MLSKVRCCGISANSGVRSLSYAGGRIPPTTQNVINGQWVESSTDRWIDVHNPATNQVVTR 208

Query 219 VPQSTNDE 242
            VPQST E
Sbjct 209 VPQSTQAE 232

>gi|66412577|gb|BW804361.1|BW804361 BW804361 Amphioxus Branchiostoma floridae unpublished cDNA library, larva whole animal Branchiostoma floridae cDNA clone bflv030d22 5', mRNA sequence

TAATATTGCTTGATTCACTTCTACAAGAGATAAAAAATGCGCGTGGAAAAGTGCTACTTTTGTGGCTCCA
GCTGTTATCCTGGCCATGGATTGACATTTGTCAGAAATGACTGCAAGATTTTCAAATTTTGCAGAAGTAA
ATGTCACAGGTCCTTCAAGCTGAAGAGAAATCCCAGGAAGACTAAGCACACCAAGGCCTACAGAAAAGCT
GCAGGCAAAGAATTGGCGGTGGATCCCACATTCGAATTCGAGAAGAAACGCAACATTCCCGTCAAGTACG
ACAGAGAACTCTGGGCCAAGACCATCGAGGCAATGAAGAAGATTGAAGTGATCAAACAGAAACGCCNACA
ACACTTCTACGCGCAG

FN251067 dmp037 Tigriopus californicus cDNA clone dmp037P0001022 5', mRNA sequence.

Sequence ID: emb|FN251067.1|Length: 726Number of Matches: 7

206 bits(444) 5e-53 78/104(75%) 91/104(87%) 0/104(0%) +1/+3

```
Query  34   KMRVEKCYFCGSSCYPGHGLTFVRNDCKIFKFCRSKCHRSFKLKRNPRKTKHTKAYRKAA  213
            KMR++KC+FCGS  YPGHG  FVRNDCKIFKFCRSKCH++FK K+NPRK K TKA+RK+A
Sbjct  60   KMRIDKCFFCGSPMYPGHGTHFVRNDCKIFKFCRSKCHKNFKKKKNPRKAKWTKAFRKSA  239

Query  214  GKELAVDPTFEFEKKRNIPVKYDRELWAKTIEAMKKIEVIKQKR  345
            GKELAVDP+FEFEK+RN PVKYDR LW +TI A+K+IE IK KR
Sbjct  240  GKELAVDPSFEFEKRRNEPVKYDRALWTETITAVKRIEEIKNKR  371
```

>gi|66410742|gb|BW802526.1|BW802526 BW802526 Amphioxus Branchiostoma floridae unpublished cDNA library, larva whole animal Branchiostoma floridae cDNA clone bflv024l18 5', mRNA sequence

```
CTAATATTGCTTGATTCACTTCTACAAGAGAGGTCAAAATGGTTGAGGGAAAAGACAAGACCAAGAACCC
CATGAGGGATCTCAGGATCAGGAAGTTGTGCGTCAACATCGCCGTCGGANAGTCTGGTGACAGACTGACC
CGTGCCGGTNAGGTGTTGGAATCTTTGGCTGGACAGACTCCTGTCTTCTCCAAGGCTAGATATACCATCA
GATCTTTCGGTATCAGACGTAACGAGAAGATCGCCGTCCACTGCACTGTCAGAGGCCCCAAGGCTGAGGA
GATCCTGGAGCGTGGCCTCAAGGTCAAGGAGTACGAGTTGAGAAGGGAGAACTTTTCCGACATGGGCAAC
TTTGGTTTCGGTATTGCTGAACATATCGATCTGGGTCTCAAGTACGATCCCTCCATCGGTATCTATGGTA
TGCACTTCTATGTTGTCCTTGGACGCCCTGGCATGAATGTCCGTCACAAGAAGAGGAAGCCCGGCAAGGT
TGGTGCTC
```

FN249076 dmp037 Tigriopus californicus cDNA clone dmp037P0001H17 5', mRNA sequence.

Sequence ID: emb|FN249076.1|Length: 632Number of Matches: 8

303 bits(656) 4e-82 125/156(80%) 136/156(87%) 0/156(0%) +3/+2

```
Query  27   REVKMVEGKDKTKNPMRDLRIRKLCVNIAVGXSGDRLTRAGXVLESLAGQTPVFSKARYT  206
            R+   V  + K KNPMR+ RIRKLC+NI VG SGDRLTRA  VLE L GQTPVFSKARYT
Sbjct  77   RKPAAVVNEVKAKNPMRETRIRKLCLNICVGESGDRLTRAAKVLEQLTGQTPVFSKARYT  256

Query  207  IRSFGIRRNEKIAVHCTVRGPKAEEILERGLKVKEYELRRENFSDMGNFGFGIAEHIDLG  386
            +RSFGIRRNEKIAVHCTVRGPKAEEILERGLKV+EYELRRENFS  GNFGFGI EHIDLG
Sbjct  257  VRSFGIRRNEKIAVHCTVRGPKAEEILERGLKVREYELRRENFSQNGNFGFGIQEHIDLG  436

Query  387  LKYDPSIGIYGMHFYVVLGRPGMNVRHKKRKPGKVG  494
            +KYDPSIGIYGM FYVV+GR GMNVRH++RK G+VG
Sbjct  437  IKYDPSIGIYGMDFYVVMGRAGMNVRHRRRKTGRVG  544
```

>gi|66400730|gb|BW792514.1|BW792514 BW792514 Amphioxus Branchiostoma floridae unpublished cDNA library, gastrula whole animal Branchiostoma floridae cDNA clone bfga054j09 3', mRNA sequence

```
AAGAGTCTTACTACTGAGACTGGTGACCTGTTGAAATGAAAATATTTACTTGGTGTAGTCGGGGCGGTTG
TTGGACAAGGTGGAGTAGTCCACTCCCCTGGGGTTGAACCACTTGAACTGACGGTTGGTGTAGTAATTGG
CCACCTCAGCCGTGTCCTTGTTCTTCCTCCAGTTGACATCACAAGTCTTGGAGGCAAGTCTTCCGACATA
TGCCCCGACAAAGATGATACCGACGGTCATGATGGCAAACAGGGGCTGGAGAGCGGTGTGGTGCTTGATG
```

CTGGCCACAGTCAGGCCCTGGGGCATCTTGTTGTTCAGCAGGCGACCTCCGGCTCCGAAGAGGTAAGGAT
GGTCGGCAGCTCGGACGATACGGGTCAGGAACATGTTGGAATAATATCTCTTGTAGAAGTGAATCAAGCA
ATATTA
FN251846 dmp037 Tigriopus californicus cDNA clone dmp037P0024F16 5', mRNA sequence.
Sequence ID: emb|FN251846.1|Length: 519Number of Matches: 3
Range 1: 27 to 191GenBankGraphics Next Match Previous Match
68.4 bits(143) 8e-19 28/55(51%) 38/55(69%) 0/55(0%) -1/+3
Query 318 LNNKMPQGLTVASIKHHTALQPLFAIMTVGIIFVGAYVGRLASKTCDVNWRKNKD 154
            +N+K+PQGLT S+KHH AL PL IM G+ FV YV RLA+ + +V+W K +
Sbjct 27   MNSKLPQGLTKESLKHHYALWPLMGIMGAGMTFVAWYVIRLATTSPEVSWSKKPE 191

Range 2: 189 to 287GenBankGraphics Next Match Previous Match First Match
43.7 bits(89) 8e-19 14/33(42%) 23/33(69%) 0/33(0%) -1/+3
Query 147 EVANYYTNRQFKWFNPRGVDYSTLSNNRPDYTK 49
            E+ N Y N+++K+ NP G+DY +++ PDY K
Sbjct 189 EIYNQYHNKRYKFLNPYGLDYEKMNDEIPDYKK 287

>gi|66388104|gb|BW785609.1|BW785609 BW785609 Amphioxus Branchiostoma floridae unpublished cDNA library, gastrula whole animal Branchiostoma floridae cDNA clone bfga021o05 3', mRNA sequence
ATGGGACGCAATACAAGAAATACAGTTTATTCGGCTTCCTCAGCCTCTTCAGCCTTCTCCTTCTTTCCCT
TCTTGCCTCCCTTCTGCTTCTTCTCGACAACAACTCCACCCTTTGGCTCCTCCTCAGCGACCTCGTTGGT
CATTCTGGTGTAGATGAGCTGGCTTCTGTGAGCGGACACGAGCTTGATCAAACCCTTGCCAGCCATCTCA
CTCAGAGCACGCTTGGCAAGAGATCCTCTAACCTTTAGCCTTTCAGAGACAATGCTGGGTGTGATAAGTT
TGTATGTGGGAACCTCCTTGTACAGTTTGTCATAGGTGTTCTTATCGAACAGAACCAAGTTGTTCAACTT
GTCCCTGGACTTTCCCTTGGACCACTTCTTCTTCTTGGCCTTGCCGCCGCCTCCTCCCTCCTTCTTCTTG
GGGTTCTTGGCGGGGGTCTTAGAATCCTTCTTAGGATCCTTTTTGGCTCCCATGTCTCTTGTAGAAGTGA
ATCAAGCAATATTAG
EST_crog_evp_898960 crog_evp Caligus rogercresseyi cDNA clone crog_evp_509_304_rev 5', mRNA sequence.
Sequence ID: gb|FK874945.1|Length: 547Number of Matches: 7
133 bits(285) 6e-31 56/73(77%) 64/73(87%) 0/73(0%) -3/-1
Query 356 RDKLNNLVLFDKNTYDKLYKEVPTYKLITPSIVSERLKVRGSLAKRALSEMAGKGLIKLV 177
            RDKLNNLVLFDK TY+KL KEVP YKLITP++VSERLK+RGSLA+ AL E+ KG+IKLV
Sbjct 388 RDKLNNLVLFDKATYEKLNKEVPNYKLITPAVVSERLKIRGSLARAALQELLSKGMIKLV 209

Query 176 SAHRSQLIYTRMT 138
            S HRSQ+IYTR T
Sbjct 208 SKHRSQVIYTRNT 170

>gi|66381819|gb|BW779325.1|BW779325 BW779325 Amphioxus Branchiostoma floridae unpublished cDNA library, gastrula whole animal Branchiostoma floridae cDNA clone bfga054j09 5', mRNA sequence

TAATATTGCTTGATTCACTTCTACAAGAGATATTATTCCAACATGTTCCTGACCCGTATCGTCCGAGCTG
CCGACCATCCTTACCTCTTCGGAGCCGGAGGTCGCCTGCTGAACAACAAGATGCCCCAGGGCCTGACTGT
GGCCAGCATCAAGCACCACACCGCTCTCCAGCCCCTGTTTGCCATCATGACCGTCGGTATCATCTTTGTC
GGGGCATATGTCGGAAGACTTGCCTCCAAGACTTGTGATGTCAACTGGAGGAAGAACAAGGACACGGCTG
AGGTGGCCAATTACTACACCAACCGTCAGTTCAAGTGGTTCAACCCCAGGGGAGTGGACTACTCCACCTT
GTCCAACAACCGCCCCGACTACACCAAGTAAATATTTTCATTTCAACAGGTCACCAGTCTCAGTAGTAAG
ACTCTTTAGATTGTAAATACCATTGAATAAAACATTTATGATATT

FN251846 dmp037 Tigriopus californicus cDNA clone dmp037P0024F16 5', mRNA sequence.

Sequence ID: emb|FN251846.1|Length: 519Number of Matches: 3

Range 1: 27 to 191GenBankGraphics Next Match Previous Match

68.4 bits(143) 9e-19 28/55(51%) 38/55(69%) 0/55(0%) +1/+3

```
Query  109  LNNKMPQGLTVASIKHHTALQPLFAIMTVGIIFVGAYVGRLASKTCDVNWRKNKD  273
            +N+K+PQGLT  S+KHH AL PL  IM  G+ FV  YV RLA+ + +V+W K  +
Sbjct  27   MNSKLPQGLTKESLKHHYALWPLMGIMGAGMTFVAWYVIRLATTSPEVSWSKKPE  191
```

Range 2: 189 to 287GenBankGraphics Next Match Previous Match First Match

43.7 bits(89) 9e-19 14/33(42%) 23/33(69%) 0/33(0%) +1/+3

```
Query  280  EVANYYTNRQFKWFNPRGVDYSTLSNNRPDYTK  378
            E+ N Y N+++K+ NP G+DY  +++  PDY K
Sbjct  189  EIYNQYHNKRYKFLNPYGLDYEKMNDEIPDYKK  287
```

>gi|66381440|gb|BW778946.1|BW778946 BW778946 Amphioxus Branchiostoma floridae unpublished cDNA library, gastrula whole animal Branchiostoma floridae cDNA clone bfga053b23 5', mRNA sequence

TAATATTGCTTGATTCACTTCTACAAGAGATTCATGTATTAATTGGGCCACTATGAAGGTGAACAATAGA
GACTCTCAGAGTGAAAATCATGTGAGCAGCCCATTCAGTGATTTCAAGCCAGCAGACTTTGTGAAAAAAC
ACAAAAAATCCTTTAGCTTGGGATTGACAGTGATAGGAGTATTAGCCATAGTTAGCACAGTTCTCAGTGT
AAATTATTCTGGTAGTGCATCAAAATTATCTCGTCAGCCAAAATTTGAGGCCCAACTTTGGCCTGAAACA
GAAGAAAATTATGAAGATATCCCAGCAAATAGAGGGAAGAGGAACCCTCAGTTCTCAAGAAATAATTTCA
AAACACCTTTCAATTTGCAAGAAATCTTGGATAACAACTTCAGTTCGGAAACATGGAACGGTACATGGGT
CAGCGACACCCAGTATGTCTACAGGGATGTTGACGAGAGTCTGATCATGGTGTCTGCCAATGGAGGATCA
G

CF_WO0_43a03_SP6 Copepod Whole Organism, Normalized Calanus finmarchicus cDNA clone CF_WO0_43a03 5', mRNA sequence.

Sequence ID: gb|EL774053.1|Length: 683Number of Matches: 11

Range 1: 439 to 639GenBankGraphics Next Match Previous Match

```
Query  284  ENYEDIPANRGKRNPQFSRNNFKTPFNLQEILDNNFSSETWNGTWVSDTQYVYRDVDESL  463
            E+ E++P+NRGKRNPQFS  NFKTPFNLQEILDNNFSSETWNG+WVSD+QYVYRDVDESL
```

Sbjct 439 EDSEEVPSNRGKRNPQFSGGNFKTPFNLQEILDNNFSSETWNGSWVSDSQYVYRDVDESL 618

Query 464 IMVSANG 484
+MV A G
Sbjct 619 VMVDARG 639

Range 2: 268 to 387GenBankGraphics Next Match Previous Match First Match
56.7 bits(118) 3e-49 24/40(60%) 30/40(75%) 0/40(0%) +2/+1
Query 110 DFKPADFVKKHKKSFSLGLTVIGVLAIVSTVLSVNYSGSA 229
+F P D +K +KKS SLGL VIG A++STVLSVN SG +
Sbjct 268 NFSPLDLLKTNKKSVSLGLVVIGAFALLSTVLSVNLSGES 387

Range 3: 412 to 450GenBankGraphics Next Match Previous Match First Match
26.2 bits(51) 3e-49 9/13(69%) 9/13(69%) 0/13(0%) +2/+1
Query 248 PKFEAQLWPETEE 286
PKFEAQ W E E
Sbjct 412 PKFEAQFWAEDSE 450

>gi|66375109|gb|BW772893.1|BW772893 BW772893 Amphioxus Branchiostoma floridae unpublished cDNA library, gastrula whole animal Branchiostoma floridae cDNA clone bfga004j21 5', mRNA sequence
TAATATTGCTTGATTCACTTCTACAAGAGAAGATGGTCGTTACCAGGAGCTGAGAGCTGTGAGGCGCCAG
CGAAGGTGTCCCTCGCGGTAGCTCACACACTCCTGCTCTCCCTCACTAGCCATTTGACAACAAGAGACAA
CATATTAAAGAAACCTTACAACATTCTCCGCTGAGGAAACCTTATTTTATCAAGATTCAAGTCAACACTT
GCCAATCAATTTTTAGTTTTTCTCATTCATTTCAACATTTTCAAAATCAAGAGCAAGATGAAACTGAAGA
ACATCGATCTTAACGCAATGATGGAGGCGGAGCAGAAGAAAAGAAAGATCTGGAAACTGGTTCTGACGGG
AGGCCCCTGTGGCGGCAAGACGACAGGCCAAGCCAGAATTTCGACTTTCTTTGAAAATCTTGGATGGAAA
GTGTACAGGGTGCCGGAGACTGCCACCGTTCTACTCGGAGGCGGTGTCAACTTTGCAGATCTGCCGGAGT
GGGCGGCAATCAAATTCCAGGAAAACCTGTTGAAGACAATGATTCAAATTGAGCAGTCATTCTTTGACCT
TGCGGAGGCCAGCGAAAGGAACTGTTTGGTGATCTGCGACC
EST_lsal_evj_1203051 lsalevj whole Lepeophtheirus salmonis (Pacific) cDNA Lepeophtheirus salmonis cDNA clone lsal_evj_019_092 5', mRNA sequence.
Sequence ID: gb|GW625572.1|Length: 877Number of Matches: 5
201 bits(434) 2e-51 81/127(64%) 104/127(81%) 0/127(0%) +1/+3
Query 220 FLVFLIHFNIFKIKSKMKLKNIDLNAMMEAEQKKRKIWKLVLTGGPCGGKTTGQARISTF 399
+ + LI+F K K+ L I +AM +++ ++ +WKLVLTGGPCGGKTTGQAR+STF
Sbjct 30 YFICLIYF*TSNYKMKLDLNTIADSAMETSKKSQKIVWKLVLTGGPCGGKTTGQARLSTF 209

Query 400 FENLGWKVYRVPETATVLLGGGVNFADLPEWAAIKFQENLLKTMIQIEQSFFDLAEASER 579
FE++GWKV+RVPETATVLL GG++F++L E A+KFQENLLKTMIQIE SFFDLA+ ++R
Sbjct 210 FEDIGWKVFRVPETATVLLSGGIDFSELDEEGAMKFQENLLKTMIQIENSFFDLAKETKR 389

Query 580 NCLVICD 600
NCLVICD
Sbjct 390 NCLVICD 410

>gi|66374451|gb|BW772235.1|BW772235 BW772235 Amphioxus Branchiostoma floridae unpublished cDNA library, gastrula whole animal Branchiostoma floridae cDNA clone bfga021o05 5', mRNA sequence
CTAATATTGCTTGATTCACTTCTACAAGAGACATGGGAGCCAAAAAGGATCCTAAGAAGGATTCTAAGAC
CCCCGCCAAGAACCCCAAGAAGAAGGAGGGAGGAGGCGGCGGCAAGGCCAAGAAGAAGAAGTGGTCCAAG
GGAAAGTCCAGGGACAAGTTGAACAACTTGGTTCTGTTCGATAAGAACACCTATGACAAACTGTACAAGG
AGGTTCCCACATACAAACTTATCACACCCAGCATTGTCTCTGAAAGGCTAAAGGTTAGAGGATCTCTTGC
CAAGCGTGCTCTGAGTGAGATGGCTGGCAAGGGTTTGATCAAGCTCGTGTCCGCTCACAGAAGCCAGCTC
ATCTACACCAGAATGACCAACGAGGTCGCTGAGGAGGAGCCAAAGGGTGGAGTTGTTGTCGAGAANAANC
AGAAGGGAGGCAAGAAGGGAAAGAANGANAANGCTGAANAGGCTGAGGAAGCCGAATAAACTGTATTTCT
TGTATTGCGTCCCATTGTAACTGAACTCCNACCNAGTTACNAGTGTTTCTAACCTTGGTTTANA
EST_crog_evp_898960 crog_evp Caligus rogercresseyi cDNA clone crog_evp_509_304_rev 5', mRNA sequence.
Sequence ID: gb|FK874945.1|Length: 547Number of Matches: 7
133 bits(285) 7e-31 56/73(77%) 64/73(87%) 0/73(0%) +3/-1
Query 150 RDKLNNLVLFDKNTYDKLYKEVPTYKLITPSIVSERLKVRGSLAKRALSEMAGKGLIKLV 329
RDKLNNLVLFDK TY+KL KEVP YKLITP++VSERLK+RGSLA+ AL E+ KG+IKLV
Sbjct 388 RDKLNNLVLFDKATYEKLNKEVPNYKLITPAVVSERLKIRGSLARAALQELLSKGMIKLV 209

Query 330 SAHRSQLIYTRMT 368
S HRSQ+IYTR T
Sbjct 208 SKHRSQVIYTRNT 170

>gi|169578713|gb|FE596692.1|FE596692 CAXG9737.fwd Amphioxus Branchiostoma floridae unpublished cDNA library CAXG, larva whole animal Branchiostoma floridae cDNA clone CAXG9737 5', mRNA sequence
AATATTGCTTGATTCACTTCTACAAGAGATTATCTCTCCTCTAACTGTGTCCTACTAAAATGGCTGCCTT
CCCCGAGATCAAGTCCACCCACTCCCTGGTGGCCAAGCACGTGACGGAGGAGAAGTGGAACAAGCTGGGG
GAGCATAAGACCGCCACCTCTGGCTTCACCCTTGGACAGGCTATTGCCTGTGCTGTCGAGTTCGACGACC
AGCACTGTGGTATCTATGCCGGAGATGCTGACTCATACAAGGACTTTGCTGAGGTCTTTGATCCCATCAT
CCAGGAGTACCATGGTATCTCAGCTGACTCTACCCACACCAGTGATATGGATGCTGGTAAGATTGTTGGA
AACATTGACGAAGATGTCCCAGTTAAATCTACCAGAATCCGTGTTGGTCGCAGCATTGAGGGATTCGGTC
TGTCCCCCGGTATCACTCAGGAGCAGAGACTTGAGGTTGAGAAGCTGATGAAGACTGCTTTCTCTAAGCT
GGAAGGTGACCTCTCTGGCGAGTACTATCCCCTCCTTGGCATGGAGGAGGAGGTCAGACAGAAGCTGGTC
GATGACCATTTCCTGTTCATGTCCGGAGATAAGAATCTGATCGCCGCTGGTATGGAGCGTGACTGGCCCG
AGGGTCGTGGCATCTTCCACAACGCCGAGAAGACCTTCTTGGTGTGGGTGAACGAGGAGGACCAGCTGAG

AATCATCTCTATGCAGAGCGGCGGTGACGTCAAGGGTGTCTTTGAGAGGCTCGCCCGCGGCATNCAGGCT
GTTCANGATTCCGTGAAGGGAGAATCTGGCAAAGACTTTATGCTTGATGAGAAGTATGGTTCCC

C5d140 C. finmarchicus C5 subtractive library, deep Calanus finmarchicus cDNA similar to arginine kinase, mRNA sequence.

Sequence ID: gb|ES387423.1|Length: 1106Number of Matches: 9

477 bits(1035) 4e-134 191/259(74%) 221/259(85%) 0/259(0%) +3/+1

```
Query  54   TKMAAFPEIKSTHSLVAKHVTEEKWNKLGEHKTATSGFTLGQAIACAVEFDDQHCGIYAG  233
            T    FP IKS HSLVAKHVT+E+W+K+ + KT T GFT+ +A ACA+EFD+QHCGIYAG
Sbjct  37   TMSGEFPVIKSAHSLVAKHVTKERWDKVKDIKTKTCGFTIAKACACAIEFDNQHCGIYAG  216

Query  234  DADSYKDFAEVFDPIIQEYHGISADSTHTSDMDAGKIVGNIDEDVPVKSTRIRVGRSIEG  413
            D D YKDFAE+FD +IQ+YHGISA + HTSDMDA K+ GNID+ VPV STRIRVGRSI+G
Sbjct  217  DWDCYKDFAEIFDAVIQDYHGISATAKHTSDMDATKVTGNIDKAVPVHSTRIRVGRSIDG  396

Query  414  FGLSPGITQEQRLEVEKLMKTAFSKLEGDLSGEYYPLLGMEEEVRQKLVDDHFLFMSGDK  593
            +GLSPGIT++QR++VEKLM TA SK+ GDL+G YYPL GM+E VRQ+LVDDHFLF+SGD+
Sbjct  397  YGLSPGITKQQRIDVEKLMSTALSKMTGDLAGSYYPLTGMDETVRQQLVDDHFLFVSGDR  576

Query  594  NLIAAGMERDWPEGRGIFHNAEKTFLVWVNEEDQLRIISMQSGGDVKGVFERLARGXQAV  773
            NL  AGMERDWPEGRGIFHNA KTFL+WVNEEDQ RIISMQ GGDVKGVFERLARG +AV
Sbjct  577  NLTVAGMERDWPEGRGIFHNAAKTFLLWVNEEDQTRIISMQQGGDVKGVFERLARGIKAV  756

Query  774  XDSVKGESGKDFMLDEKYG  830
             DSVK E GKDF L E+YG
Sbjct  757  GDSVKAECGKDFALSEQYG  813
```

>gi|169576431|gb|FE591355.1|FE591355 CAXG5584.fwd Amphioxus Branchiostoma floridae unpublished cDNA library CAXG, larva whole animal Branchiostoma floridae cDNA clone CAXG5584 5', mRNA sequence

CTAATATTGCTTGATTCACTTCTACAAGAGAAGTAAAAATGACGGCAACGCCTGGTGCAACCATTAGGCC
CCGCAAGAAGCTTCCTGCAGTCCCTGAGACCATCCTCAAGAAGCGCAAGCGCAATGATGAGGAAAGGGCA
TCCAAACGTAAGGAACAGAAAGAGCTGAAAACTAAAAGTTTGGAGAAGCGCAAAGAATATTTCAGACGTG
CTGAAAGCTACGTACAGGAGTATTTGGCTGCACAGGCGGACGAAAGGAGGTTGAAGATGGAAGCCAAGAA
GTCTGGTAACTTTTACGTTCCCTCTGAGCCTAAGCTCGCCTTTGTTATCAGAATTCGTGGTATCAACCAG
ATTGCTCCCAAGCCCCGCAAGATCTTGCAGCTGTTCCGTCTTCGCCAGATCAACAATGGTGTCTTCATCA
AGCTGAACAAGGCCACTATCAACATGTTGAGAATCTGCGAGCCCTACGTTACATGGGGAACCCCTAATCT
TGACGTTATCAGAAAGCTGATCTACAAGAGAGGATTCATCAAGGTTAATGGAGACCGTAAGCCCATTGAT
GACAACGAGGTTATTGAGGAGATCCTTAAGAAGAAGAAGTGCGATATTATTTGCGTTGAGGATTTGATCC
ACGAGATCTACACCGTTGGTGACAACTTCAAGTACGCCTCCAACCTGCTGTGGCCCCTTTCAGCTGAACAA
CCCCAAGGGTGGATGGAGAAAGA

FN4561-2003-03-05.ab1 Lepeophtheirus salmonis ME Lepeophtheirus salmonis cDNA, mRNA sequence.
Sequence ID: gb|HO670396.1|Length: 846Number of Matches: 12
Range 1: 45 to 605GenBankGraphics Next Match Previous Match

280 bits(605) 1e-95 119/187(64%) 142/187(75%) 0/187(0%) +3/+3
```
Query  33   VKMTATPGATIRPRKKLPAVPETILKKRKRNDEERASKRKEQKELKTKSLEKRKEYFRRA  212
            +   A     I P KKLPAVPET+LK+RKR  E RA++ K     K     KR + F+RA
Sbjct  45   IPRAAGDNEAIMPDKKLPAVPETVLKRRKRLAENRATRAKAVIASKKTRHAKRIDVFKRA  224

Query  213  ESYVQEYLAAQADERRLKMEAKKSGNFYVPSEPKLAFVIRIRGINQIAPKPRKILQLFRL  392
            E YV+EY   +ADE RL+ +AKK G +YVPSEPKLAFV+RIRGIN++APK RK+LQLFRL
Sbjct  225  ERYVKEYRNKEADETRLQRDAKKEGGYYVPSEPKLAFVMRIRGINKVAPKVRKVLQLFRL  404

Query  393  RQINNGVFIKLNKATINMLRICEPYVTWGTPNLDVIRKLIYKRGFIKVNGDRKPIDDNEV  572
            RQINNGVFIKLNKATINMLRICEPY+TWG+PNL  IR+LIYKRGF+K+ G R PI  N +
Sbjct  405  RQINNGVFIKLNKATINMLRICEPYITWGSPNLKSIRELIYKRGFVKIQGKRIPITSNTL  584

Query  573  IEEILKK  593
            IE +L K
Sbjct  585  IESVLGK  605
```

Range 2: 603 to 728GenBankGraphics Next Match Previous Match First Match
90.0 bits(190) 1e-95 34/42(81%) 36/42(85%) 0/42(0%) +3/+3
```
Query  597  KCDIICVEDLIHEIYTVGDNFKYASNLLWPFQLNNPKGGWRK  722
            K +IICVEDLIHEI TVG NFKYASN LWPF+LN P GGWRK
Sbjct  603  KYNIICVEDLIHEIVTVGPNFKYASNFLWPFKLNTPTGGWRK  728
```

>gi|169557514|gb|FE573549.1|FE573549 CAXF7035.fwd Amphioxus Branchiostoma floridae unpublished cDNA library CAXF, gastrula whole animal Branchiostoma floridae cDNA clone CAXF7035 5’, mRNA sequence
AATATTGCTTGATTCACTTCTACAAGAGATGAAGGTTGTTGGCTTGGTGTCCGGAGGCAAGGACAGTTGC
TACAACCTGGTGCAATGCGTTGCCGCTGGTCACGATGTCACGTGTCTGGCGCACCTTGTGCCACGTGAAG
CGACGTGTCACGAGGCGGACAGTCACATGTACCAGACCATCGGCTGGGACAGTGTCAACATGATTGCCCA
GGCCATGCAGCTCCCTCTCTACGTGGAGACTGTCCAGGGCGAGGGTGTCAATGTTGGCCGAGACTATATA
CCCCAAGAGGGTGACGAGGTGGAGGACCTGTACAGGTTGTTGTCACGGGTGAAGGCAGAGACAGGAGTGG
AAGGTGTGGCAGTGGGGGCAATATTGTCAGACTACCAGAGAGTCAGGGTGGAGAGTGTGTGTCTCCGGCT
GGGTCTCACTCCCCTGGCCTACCTGTGGCAGAGAGACCAGTCCCAGCTGCTTGGTGATATGATAGAGGCT
GGGATGGAGAGTATATTGGTCAAGGTGGCATGCTTGGGGCTGGAGAAGAAACACCTTGGACAGAGCTTGG
CTCAGATGAGGCCGGAGCTGGAGAGGTTGGCAGACAAGTTCGGTGTCAATGTGTGCGGAGAGGGAGGGGA
GTATGAGACATTCACCCTGGACTGTCCTCTCTTCAGACACAAGCTGGCCATCACAACCTCCAAGGTCTTG
ACACATTCAGACGACGCCTTCGCCTCAGTCCATCTTCTGAACATCCAATGTAAGCTTCAGAGTANAGGTG

AACAGTTCCAGAGGATGTCACA

EST_lsal_evj_1201737 lsalevj whole Lepeophtheirus salmonis (Pacific) cDNA Lepeophtheirus salmonis cDNA clone lsal_evj_018_231 5’, mRNA sequence.

Sequence ID: gb|GW625095.1|Length: 852Number of Matches: 6

303 bits(656) 7e-82 118/232(51%) 171/232(73%) 0/232(0%) +2/+3

```
Query  26   EMKVVGLVSGGKDSCYNLVQCVAAGHDVTCLAHLVPREATCHEADSHMYQTIGWDSVNMI  205
            +MKVV LVSGGKDSCYN+VQ +  GH++  L +L P      E DS+MYQT+G  ++++
Sbjct  138  KMKVVALVSGGKDSCYNIVQAIKDGHEIVALGNLYPENKEVEELDSYMYQTVGHGAIDLY  317

Query  206  AQAMQLPLYVETVQGEGVNVGRDYIPQEGDEVEDLYRLLSRVKAETGVEGVAVGAILSDY  385
            A+A +LPLY E + G  + +   Y   E DEVEDL+RLL+++K +   + VA GAI S+Y
Sbjct  318  AEAFELPLYREPITGSPLCLDSVYQKNEKDEVEDLFRLLTKIKKDIPFDAVASGAIFSNY  497

Query  386  QRVRVESVCLRLGLTPLAYLWQRDQSQLLGDMIEAGMESILVKVACLGLEKKHLGQSLAQ  565
            QRVR+E +C R+GL  L YLW+R+Q +LL +MI   +E+I+VKVA LGL++ HLG+++A+
Sbjct  498  QRVRIEDICSRMGLKSLTYLWERNQRELLQEMISCPIEAIVVKVATLGLDESHLGKTIAE  677

Query  566  MRPELERLADKFGVNVCGEGGEYETFTLDCPLFRHKLAITTSKVLTHSDDAF  721
            ++P L ++ADKFG+NVCGEGGEYETFTL+CPLF+ KL +  ++V+ +  D +
Sbjct  678  LQPHLLKMADKFGINVCGEGGEYETFTLNCPLFKKKLVLKETEVVPNGADVY  833
```

>gi|169556382|gb|FE568647.1|FE568647 CAXF17113.fwd Amphioxus Branchiostoma floridae unpublished cDNA library CAXF, gastrula whole animal Branchiostoma floridae cDNA clone CAXF17113 5’, mRNA sequence

AATATTGCTTGATTCACTTCTACAAGAGATAAAAAGGAGTGACAAAGTGTGGAGTGTGAAAGGAGAGTGT
AGAGGTGAGTGACTGGAATCCCTAGAGCCGCAGCATGGCGTTGTCGGCCAAATACTTGTTTGCTTGGTGC
TTTGTTGCTCTTTGCTTGGTCGGAACCACCTTCGCCAACGAGGAAGATGATGATGAGGAGGTTGGAGATG
TTGCCTCTGAAGGTATCAATTGCGAAGATGGTCTCCTTATCCCCATCTGGCCCGGATTTGATGAAATGAG
CATGGGAGACAGATTCGGACGCGGCCTGCTCTATATTGCCCTCATGATTTACCTGTTCATTGGCGTAGCC
ATCGCCTCTGACAAGTTCATGGAGTCTATTGAGATGATCACCGCCCAGGAGAAGGAGGTTGCCATCAAGG
ACCCCAAGACCGGCAAGTCCCAGGTGGTGGTGGTCAAGGTCTGGAACGAGACTGTGGCCAACTTGACCCT
GATGGCTCTGGGATCCTCCGCCCCCGAGATCATGTTGTCCGTCATTGAGATCTGGGCCAAGAACTTCAAA
GCTGGTGACCTGGGACCCGGCACAATTGTCGGCTCCGCTGCCTTCAACTTGTTCATGATTATCGGTCTCT
GCATGTACGTTATTCCCGACGATGAGGTGAGGAAGATCAAGCATCTGCGCGTGTTTTGCATCACCGCCAC
TTGGTCTGTGTTCGCCTATGTCTGGCTTTACGTCATCTTGGGAGGCATTTCCTATGGAGTGGT

CF_W01_58b12_SP6 Copepod Whole Organism, Normalized Calanus finmarchicus cDNA clone CF_W01_58b12 5’ similar to ref|XP_001663976.1| sodium/calcium exchanger - Aedes aegypti. Score = 130 bits (326), Expect = 2e-28, mRNA sequence.

Sequence ID: gb|GR411149.1|Length: 875Number of Matches: 4

Range 1: 202 to 489GenBankGraphics Next Match Previous Match

138 bits(296) 1e-61 50/96(52%) 76/96(79%) 0/96(0%) +3/+1

```
Query  222  GINCEDGLLIPIWPGFDEMSMGDRFGRGLLYIALMIYLFIGVAIASDKFMESIEMITAQE  401
            GINCEDG +IP+WPG D++S+GDR GRGLLY  +++YL +G++I  +K MES+E +T+
Sbjct  202  GINCEDGFIIPLWPGTDDLSIGDRVGRGLLYTLVLLYLMLGISIYLNKMMESMETMTSLM  381

Query  402  KEVAIKDPKTGKSQVVVVKVWNETVANLTLMALGSS  509
            K  ++ D +TGK+QV++VK+WN+TVAN+ ++   SS
Sbjct  382  KRTSVPDLETGKTQVIIVKIWNQTVANMFMVLGSSS  489
```

Range 2: 469 to 735GenBankGraphics Next Match Previous Match First Match

118 bits(252) 1e-61 45/89(51%) 64/89(71%) 0/89(0%) +3/+1

```
Query  492  MALGSSAPEIMLSVIEIWAKNFKAGDLGPGTIVGSAAFNLFMIIGLCMYVIPDDEVRKIK  671
            M LGSS+P I L +IEI+AK F AGDLGP T++GS+AFNL +++G+ +  +P  +VRK+K
Sbjct  469  MVLGSSSPLIFLCIIEIFAKGFNAGDLGPSTVIGSSAFNLLIVVGVIISSVPRSQVRKVK  648

Query  672  HLRVFCITATWSVFAYVWLYVILGGISYG  758
            +L  F + A WS   Y WL+ +L  +SYG
Sbjct  649  NLLTFVLIAVWSFILYPWLFSMLSLVSYG  735
```

>gi|169547940|gb|FE565967.1|FE565967 CAXF14625.fwd Amphioxus Branchiostoma floridae unpublished cDNA library CAXF, gastrula whole animal Branchiostoma floridae cDNA clone CAXF14625 5', mRNA sequence

```
CTAATATTGCTTGATTCACTTCTACAAGAGAAGATAAAATGCCGTTGGCTAAAGATTTGCTTCATCCCCT
CGCTCACGAAGAGAAGAGGAAGCACAAGCTGAAGAGGCTTGTCCAGCATCCCAACAGTTACTTCATGGAT
GTCAAGTGCCCCGGCTGTTACAGAATCACCACAGTGTTCAGTCACGCCCAGACTGTTGTTGTGTGTGCCG
GATGCTCCACCGTCTTGTGTCAGCCAACCGGAGGCAGGGCTAGGTTGACCGAAGGCTGCAGTTTCAGAAG
GAAGCAGCACTAAACAACTGTTGGGCGATGTGGAGTTAAAATATTTCAAGTTTTGGGAATCATATATTTC
CTTGACCACGCATTGTACATCCATTGTGTTTTCCGAGAATAAATATTATGTGATAAAAAAAAAAAAAAAA
AAAAAAAAAAAAAA
```

EST_crog_evp_908374 crog_evp Caligus rogercresseyi cDNA clone crog_evp_524_118_rev 5', mRNA sequence.

Sequence ID: gb|FK885479.1|Length: 544Number of Matches: 6

194 bits(419) 2e-49 76/86(88%) 80/86(93%) 0/86(0%) +3/-3

```
Query  39   MPLAKDLLHPLAHEEKRKHKLKRLVQHPNSYFMDVKCPGCYRITTVFSHAQTVVVCAGCS  218
            MPLAKDLLHP + EEKR HK KRLVQ PNSYFMDVKCPGCY+ITTVFSHAQTVV+C GCS
Sbjct  509  MPLAKDLLHPTSEEEKRSHKKKRLVQSPNSYFMDVKCPGCYKITTVFSHAQTVVLCVGCS  330

Query  219  TVLCQPTGGRARLTEGCSFRRKQH*T  296
            TVLCQPTGG+ARLTEGCSFRRKQH*T
Sbjct  329  TVLCQPTGGKARLTEGCSFRRKQH*T  252
```

>gi|169549172|gb|FE563520.1|FE563520 CAXF12045.fwd Amphioxus Branchiostoma floridae unpublished cDNA library CAXF, gastrula whole animal Branchiostoma floridae cDNA clone CAXF12045 5', mRNA sequence

```
AATATTGCTTGATTCACTTCTACAAGAGAGCATCTTTGGGCTGACTGAGATGAGTGGAGGTAGTCAGGAC
AGCAATATGACAGCTACCGGTCTGAGTCTGATAGTCCGGGGCAGCCAGGGACTGTGTCTCCAGGCCTCTC
CAGACTTCACCATCCACCAGGGCTTTGCTCCAGACCTGAGCAAGAATGTCAAGGTGTTTGGCTGGAACAG
GACCGGAAGCAGTGTGGCTTGGTCCAACATGACAGGAGTGACAATTGCTGGTCACAACTCGAAGACTGGC
AAGTGGGACACTCTTCATCAGATCAATCAATCAAAGGTTGTGTGTCTGTCCTGGTCTCCGTGCGGCAGTT
TGCTTGCAACTTGGGAGCAGTATGCTGTGATCCAGGGCCAGCAGCCTCAACCCAACCTCCACCTGTGGGA
CGTCAGCACTGGGCAGCTGGTCAAGAGCTTCTTCCAGAAGAAGATGAGTGGCTGGTGTCCTCAGTGGAGC
GAGGACGAAAAGATATGCTCAAGGATGGTGAACAATGAGGTCCAGTTCTACGAGGATAACAGTTTTCAGG
CCATCGCCCATAAGATCCACATGGCCAAGGTGGCCAACTACAGCATGGCTCAGGGCGGCTCCAAGGGCAT
TCACGTTGTCACCTACGTCCCAGGAGCCAAGGGAGCCCCCAGCTTCACCAAGATGTATCAGTATCCGAAC
TTTGCTGACAC
```

CF_WOO_26h06_SP6 Copepod Whole Organism, Normalized Calanus finmarchicus cDNA clone CF_WOO_26h06 5' similar to ref|XP_534306.2| PREDICTED: similar to CDA02 protein isoform 1 - Canis familiaris. Score = 199 bits (505), Expect = 1e-49, mRNA sequence.

Sequence ID: gb|EL696767.1|Length: 686Number of Matches: 21

Range 1: 233 to 607GenBankGraphics Next Match Previous Match

205 bits(444) 8e-89 80/125(64%) 95/125(76%) 0/125(0%) +2/+2

```
Query  251   TIAGHNSKTGKWDTLHQINQSKVVCLSWSPCGSLLATWEQYAVIQGQQPQPNLHLWDVST  430
             T+     K GK+   H + Q+KV CL+WSP  ++LATWEQY    GQ PQPNLHLW+  T
Sbjct  233   TVQIAREKDGKFAVEHTLPQTKVYCLAWSPDSTMLATWEQYTTTAGQAPQPNLHLWNTDT  412

Query  431   GQLVKSFFQKKMSGWCPQWSEDEKICSRMVNNEVQFYEDNSFQAIAHKIHMAKVANYSMA  610
             G LVKSFFQKKM  WCP WS D KICSRMVNNEVQFYE+++F AIAHKIH+AKV+++SMA
Sbjct  413   GALVKSFFQKKMKNWCPMWSLDTKICSRMVNNEVQFYENSNFDAIAHKIHLAKVSDFSMA  592

Query  611   QGGSK  625
              G SK
Sbjct  593   PGYSK  607
```

Range 2: 68 to 247GenBankGraphics Next Match Previous Match First Match

92.6 bits(196) 8e-89 37/60(62%) 44/60(73%) 0/60(0%) +2/+2

```
Query  80    TATGLSLIVRGSQGLCLQASPDFTIHQGFAPDLSKNVKVFGWNRTGSSVAWSNMTGVTIA  259
             + T  SL+VRGS GL LQA PDF +HQGF PDL+K+VKV  W+R G  +AWSNM  V IA
Sbjct  68    SVTSPSLVVRGSVGLSLQAPPDFAVHQGFVPDLAKSVKVMAWDREGKGLAWSNMGTVQIA  247
```

Range 3: 596 to 682GenBankGraphics Next Match Previous Match First Match

58.7 bits(122) 8e-89 21/29(72%) 25/29(86%) 0/29(0%) +2/+2

```
Query  617   GSKGIHVVTYVPGAKGAPSFTKMYQYPNF  703
```

G   HVVTYVPG+KGAPSFT++YQYP+F
Sbjct 596 GYSKFHVVTYVPGSKGAPSFTRLYQYPSF 682

>gi|169551873|gb|FE562105.1|FE562105 CAXF10707.fwd Amphioxus Branchiostoma floridae unpublished cDNA library CAXF, gastrula whole animal Branchiostoma floridae cDNA clone CAXF10707 5', mRNA sequence
CTAATATTGCTTGATTCACTTCTACAAGAGATCATGATCAAATAGAAAACAGACAGACAGTCAGGATGCT
CCGACTGCCACCAGGTATCTCTCTTCTCCATTTCTCTGGCATCTGTCCGCCCTCAGTGGACAGGAGGGTC
CTCACCTACAGGCTCTCCATCCTCCTGCTCACCTTCTGCGCCTACACCAGCTACCACCTCTCCAGGAAAC
CCATCTCCATCGTCAAGAACAGCAAAGCATTCCTTGACTGTGGAGACAACCATAATGAGAACTCCACCTG
CAAGTCCTGGATTAGTGAGATTGATGGAAAGACTGAGCAGGAAGCCAAGACCTACCTTGGTCTCCTGGAC
ACAGCCTACCTCTTCTCCTACGCCTTCTTCATGTTCTGGTCAGGTGCCCTGGCTGAGAGGATGGACCTCC
GGTACTTCCTGTCCCTGGGCATGCTCTTCTCCGGAATCTTCACCTTCCTGTTTGGTCTAGGCCATGATGC
TGGAATCCACACCATTGTCTACTACATTGCAATTCAGGTGATACTGGGACTGTTCCAGTCCTCCGGCTGG
CCGGGTGTGGTGACGGTGATGGCCAACTGGTTTGGTAAGGGCAGGCGAGGCCTCATCATGGGTCTGTGGA
ACAGTCACACTAGCCTGGGCAACATTTTTGGCTCTCTGATAGCAGGGGCCTTCGTGACCAGCAACTGGGG
GCTGTCCTTCACCGTGCCGGGCATGCTGATTGCCGGGATAGGATTCATATTGTTCATGTTCATGGTGC
CF_WO0_51a03_SP6 Copepod Whole Organism, Normalized Calanus finmarchicus cDNA clone CF_WO0_51a03 5' similar to ref|NP_726048.1| CG10069-PB, isoform B - Drosophila melanogaster. Score = 209 bits (533), Expect = 8e-53, mRNA sequence.
Sequence ID: gb|EL965902.1|Length: 722Number of Matches: 8
373 bits(808) 8e-103 149/202(74%) 175/202(86%) 0/202(0%) +3/+1
Query 69   LRLPPGISLLHFSGICPPSVDRRVLTYRLSILLLTFCAYTSYHLSRKPISIVKNSKAFLD 248
+ + PG+ L+ +G CP V RR L YRLSILLLTF AYTSYHLSRKPISIVKNS AFLD
Sbjct 115 MAVAPGVKLISAAGACPSPVWRRELIYRLSILLLTFLAYTSYHLSRKPISIVKNSAAFLD 294

Query 249 CGDNHNENSTCKSWISEIDGKTEQEAKTYLGLLDTAYLFSYAFFMFWSGALAERMDLRYF 428
CG HN+++ CKSWI++IDGKTE EAKT+LGLLDT+YLFSYAFFMF SG +AERMDLRYF
Sbjct 295 CGKVHNDSTRCKSWITQIDGKTEAEAKTFLGLLDTSYLFSYAFFMFGSGFVAERMDLRYF 474

Query 429 LSLGMLFSGIFTFLFGLGHDAGIHTIVYYIAIQVILGLFQSSGWPGVVTVMANWFGKGRR 608
LSLGM+ SG+FTFLFG   +AGIH++ Y +AIQ++L +FQ++GWPGVV+VMANWFGKG+R
Sbjct 475 LSLGMIISGVFTFLFGFAKNAGIHSLWYLLAIQIVLXMFQATGWPGVVSVMANWFGKGKR 654

Query 609 GLIMGLWNSHTSLGNIFGSLIA 674
GLIMGLWNSHTSLGNI GSLIA
Sbjct 655 GLIMGLWNSHTSLGNILGSLIA 720

>gi|169533867|gb|FE546172.1|FE546172 CAXC14451.fwd Amphioxus Branchiostoma floridae unpublished cDNA library CAXC, neurula whole animal Branchiostoma floridae cDNA clone CAXC14451 5', mRNA sequence

CTAATATTGCTTGATTCACTTCTACAAGAGATTTCCATCTCTTGCTATTGACGTTTTTATCCCTTAAAAC
TAATCCTTACAACACAAGATGAGTAGGGAGGGTGGAACCAAAGCCGTATGGAAGGGCAAGTACTTCGAGA
AGATTGCCGCCCTTTTGGATGAGTACCCCAATTGCTTCATCGTGGGCGCCGACAACGTCGGTTCCAAGCA
GATGCAGGAAATCCGTATTGCTCTGCGCGGAAAAGCTATTGTGTTGATGGGAAAGAACACCATGATGAGA
AAGGCCATCAGAGCTCATATGGAGAAAAATCCTGATCTTGAAAAGCTGCTCCCATGCATCAAGATGAACG
TTGGTTTCGTCTTCACCAAGGAGGACTTGTGCGAGATCCGTGACCTGCTTCTGGCCAACAAGGTCAAAGC
TCCTGCCAAGGCTGGTGCTATTGCCCCTCTGGACGTTACTGTCCCTGCCCAGAACACTGGTATGGGTCCC
GAGAAGACCTCTTTCTTCCAGGCTCTGTCTATCCCAACCAAGATTACCAAGGGTACTATTGAAATTATTC
AGGATGTGCACTTGATTAAGACTGGTGACAAGGTCGGTATGTCCGAGTCTACCCTCTTGAACATGTTGAA
GATTTCTCCCTTCACCTATGGTCTGGTTATCCAGAAGGTCTACGATGCTGGATCCGTCTTCGACCCCGAG
ATCCTCGACATCACCGACGATGTCCTCAAGGCCACCTTCATGGCTGGTGTTGCCAACGTTGCTTCAGTTT
GCCTGGTCATTGGCTACCCAACTGTTGCCTCCGTGGCCCACTCTGT
LF1631-2007-04-23.ab1 Lepeophtheirus salmonis LF Lepeophtheirus salmonis cDNA, mRNA sequence.
Sequence ID: gb|HO675246.1|Length: 829Number of Matches: 12
408 bits(885) 2e-113 168/219(77%) 193/219(88%) 0/219(0%) +2/+1

```
Query  110   KAVWKGKYFEKIAALLDEYPNCFIVGADNVGSKQMQEIRIALRGKAIVLMGKNTMMRKAI  289
             KA WK  YF K+  L DEYP CF+VGADNVGSKQMQ+IR +LRGK +VLMGKNTMMRKAI
Sbjct  172   KATWKSNYFLKLVQLFDEYPKCFLVGADNVGSKQMQQIRASLRGKGVVLMGKNTMMRKAI  351

Query  290   RAHMEKNPDLEKLLPCIKMNVGFVFTKEDLCEIRDLLLANKVKAPAKAGAIAPLDVTVPA  469
             R H++KNP+LEKLLP I+ NVGFVFT EDL  +RD L+ANKVKAPAKAGA+APLDV VPA
Sbjct  352   RGHIDKNPNLEKLLPHIRGNVGFVFTSEDLVAVRDSLIANKVKAPAKAGALAPLDVRVPA  531

Query  470   QNTGMGPEKTSFFQALSIPTKITKGTIEIIQDVHLIKTGDKVGMSESTLLNMLKISPFTY  649
             QNTG+GPEKTSFFQAL+IPTKITKGTIEI+ DVHLIK GD+VGMS++TLLNMLKISPF+Y
Sbjct  532   QNTGLGPEKTSFFQALNIPTKITKGTIEIVHDVHLIKAGDRVGMSDATLLNMLKISPFSY  711

Query  650   GLVIQKVYDAGSVFDPEILDITDDVLKATFMAGVANVAS  766
             GLV+Q+VYD+G++F P ILDITDD +KA FM GVA VAS
Sbjct  712   GLVVQQVYDSGTIFHPSILDITDDDVKARFMEGVAVVAS  828
```

>gi|169578769|gb|FE596722.1|FE596722 CAXG9756.fwd Amphioxus Branchiostoma floridae unpublished cDNA library CAXG, larva whole animal Branchiostoma floridae cDNA clone CAXG9756 5', mRNA sequence---No hit
AGCTAATATTGCTTGATTCACTTCTACAAGAGAATTAACAAAATGCCAGCTGATCTGAAGAAGGCAGACA
CCCCAAAACCGGCGGCTGTAAGGAAGCCCAGCCTAAAGGAGATACCCAGCTATTTGAAAGAAGTGGATGT
AGCCACCAAACGCAGACAAGAACTTCTTAAATTGAAGGAGAGGCTCAGCAGCAGCAAGCTGATCCTGAGC
GCTGATCCTGCGTCCAAGCCATGGCTCCTAGCCAGACAAATATCTGCTGCCAAGCTGGAGTCTGTCAAGA
AGGTAGAGCCCCGTATAACTCCTGAACGCCTGTACTCATCCTCTTCTGAATAAAGACTCACGAGCAAGGG
ACGCGAAGCCTTAAACAGACAAAATCTACATATTTCTTACCTGCATATGTATCTATCCAAAAGTAAAACT
TATAGCCATGTTTTAAAGCAAAATCAGTTTAAACAAAAAAATGTTTTGAAATTGGCAGCAAAACGAAGTT

TCAATTCTAAAGCATCTTTGAATACTTATCCACAATAGGTGTACTTACTTTTATATTCTATTTATGATTT
ATAAATGGATACAATATACAATTTAAAATCAAAAGTTTTTTCAATATCAAAATAGTTGCCCAAAATGCAA
TATCATATTTCAATCTCAGTTGTATAAAAATGTATTTAATATTTACTAACTTATCAATGTAAATATTTTA
ACAAAATTAATATAAAGTTAACAAAATCTTATTTAAATAAAACAATAAAATTATAATTTTGAAAAATACG
ACTTTTTTATATGTATTTAAAAAAGATTTTGTTGAGTATTATATAAATAACATGATCATAACATTTTATG
CATTTTTTCATATTATTATCACC

>gi|169582222|gb|FE596360.1|FE596360 CAXG9496.fwd Amphioxus Branchiostoma floridae unpublished cDNA library CAXG, larva whole animal Branchiostoma floridae cDNA clone CAXG9496 5', mRNA sequence---No hit
AGCTAATATTGCTTGATTCACTTCTACAAGAGTTTTAGAGCTTTAAGTGCCAACATGTCTTTGTCCTCCC
TGATATCAGCAGCACAGACTGCTTTGGAGATGTCAGAAGCAGCTGCTCAAGCTGCTGAGGAGGCAATGAC
AGCCTCAAAACTGGCAACAGTTCATGCCAAAGCTGCTCTGGAGGCTGCCAAACTTGCTGAGGACGTTGAT
AGGAGACTTAAAGCCACAACAGCACCAGAGCCTCCAGATAGAGAACAAAGCATTCCTCAGACCCAGGGTG
CCAAGTTTGTGTCCTCAGTTGAAGAGGATATGTTTCAAGAGGAATCAAAAAGGTCCAGAACCAAGCAACA
TAACCCACCATCTCAAAGTAGCAGTTACAGTGCTGACGTCTCCGAGGGTCATGGTGACAATGACAATCCA
GGATTAAACAACAACAACAATATCAAGTCTCAAACCTTTGAAGGAGCAGCAGCCGCCAGCAAGTCTGGCT
ATTGTGATAACTGTGGGAGTACTAGTGAATGTCTTCATGGCAAGTTTCTCTTCTATGTGACAGAGAAGAA
CAAAGACATGAGGTTGAAGACATGTGGAAGTGTATGGCCATCAGAGCTGAGAGGAAAGCTTTGTTGGGTT
GTCAACGGTGGGGATGCCATTGTGTGGATTAGGTGGAAAGGTCCAGGTTCTGGTGGTGGCAAGATCGAAA
GCTACAAGTTGAAGACAAGAGGAGAATGTGCTTTCCAGTTTTGGTGTCATGATGAATCCGACTC

>gi|66446915|gb|BW838699.1|BW838699 BW838699 Amphioxus Branchiostoma floridae unpublished cDNA library, neurula whole animal Branchiostoma floridae cDNA clone bfne001b15 5', mRNA sequence---No hit
GCTAATATTGCTTGATTCACTTCTACAAGAGAATCAACAAGTCTTGGTCAAAATTAAGTCCAAAAACTTG
TTAAAAGTAAATGAAAATGGTAACAGCTACAGCCGTTGACATGGACAAAGCTGAGCGCAAGAAAAAGCGC
AAACTCCAGAAACTGGAGGAGGAGGGAAAGGAGAAGGTAGTTGAGAATCAGGACAACGAGGAAAAAGTTC
CCAAGAAGAAG

>gi|66372971|gb|BW770801.1|BW770801 BW770801 Amphioxus Branchiostoma floridae unpublished cDNA library, gastrula whole animal Branchiostoma floridae cDNA clone bfga016d06 5', mRNA sequence---No hit
GCTAATATTGCTTGATTCACTTCTACAAGAGAAGAGGAAGGGTTGGAGTTCTCAATGCTGTGAGAATAGA
GAGTGATCTGCATTGTATTATTGACAAACCTGGGACAGGAGAGAGAGGGTTGCCTAGGATACCAACATTG
ACAGTGTACAGTGGCACACCTGTCAGTAACATTGTCAGTAACCCACACACCGGACACAGTGAAATCAGTT
GCGTTGCAACAGTGAAATAAATAGTTGAGTCAGTAATAGAGTTGAGCGGCAGAAAAATATTTTACTGAGT
GACGGAGAAAATATTTGAGCATGTCGGGTTGGCGGGGCGGTGACAATCGATGTGCCACGGTGTTACACGG
AGGTGGCATTGGCAACTACGATGGCTACAAGAGCCAATATGGTTACGGTAACCAATACGGAGACAGATAT
GGAGGTCCAGGGGGTCCCTACCCCTACCAGTCCCCAAGCTGGGGCTATGTCATCCTCTACCTGACCATCA
TCATCATTGGCCTCGTCGGCAACTTCGTCTTCTGCTACATCATCAAGAAATGCGACCATCTCCACCGTAC

ACATCACTTCTTCTTCCTATCAATATCCGTCATGGACATGTTGGCCTGCGTTCTCGTCATTCCATTCGCG
ATTGACTCCTCGTCCAAGTTTGCCCGATGGTATCTGCCCGACTTTCTCTG

>gi|66375869|gb|BW773653.1|BW773653 BW773653 Amphioxus Branchiostoma floridae unpublished cDNA library, gastrula whole animal Branchiostoma floridae cDNA clone bfga007k20 5', mRNA sequence---No hit
TAATATTGCTTGATTCACTTCTACAAGAGTATAAATTCCGCAAGAAAAAGTAAATATTATAGCTTGTGAA
AGGCAAGTCAAGCAGAGACCATGATTACCTCAAGAAGCAAGTCTGAAGACTCCAGAGTCAGCAAATCATT
GGCATCGGGCTCAGTGCCTTCAAAGTGCTTTGAGGAAAGTGCAACCAAACATGAAGAGAATACTACATTA
TACCTTGCAGTCAAGTTTGACAAGTTGATAGTTTCACCAAATACCTCTTTCAAGGGCTCAGGTTCTAAAA
GTACACCGTTGGTCAAAAGTGAGGAGAAAGTATTGAAAATCAAGAAAGACAACAAGAAGAGGCCTTTGAT
AAGGAAGAGCAAACTTGGAGAATCTTTTGCAAAGTTTAGAGACAGTGTATCTTCTCGTTGTACAGTCAAA
GAAAAAAATCCTTCAGAGAAAAGTGTCTTTGGCAGAAAATATTCATTCAGTCTAGAAAACTTTAACAGTC
TTTCACTGCAAGACAACGCAGAGGCAGGCAGCAGCTCTTCATCAGAAAAATACTCAACAGAAAGCTTTAA
AACAATTAATGGTTCTGTTGGAAAAAGGAAGCGAGAGCGTTGTCACAGCTGTGCCGAGCGGCTAGAGACC
ATTGACCAGGCGACCAGTGAGAGCACCAGTGAGGCTGGCAGCATCGTCAGCTGTGGCCAGCAG

>gi|95132184|gb|EB740555.1|EB740555 Sa_mx1_34h03_SP6 Dogfish Shark Multiple Tissues, Normalized Squalus acanthias cDNA clone Sa_mx1_34h03 5' similar to ref|XP_395978.2| PREDICTED: similar to out at first, partial - Apis mellifera. Score = 97.8 bits (242), Expect = 3e-19, mRNA sequence
ATAGCTAATATTGCTTGATTCACTTCTACAAGAGAATTAGAAAATGTTTGTCTGTGTTTACTTTCTTCTC
CGTGTATTGACTGGAAGCTCTGGAGAACTAGTGATCAATGTCAGCAAGGGAACAGAACAGGCGTCCGTGT
ACCAGCAGCACATCAGCGGCAACCTCACCACCAACTCCGTCAAGATACAGTTCATCTCCCCCCAGGGGGA
CTCCGTTAATCAGGTGACTGACTTAAAGAACCGGGTTGTGATAACTGTGTACACAGTGCCAGGGGAGCAG
GACCTTGGTCAGGATGGATATCAGGTTTTTTGCTTTGTGACCGCGTACACCGGCGACCTGATCCCCACGG
AGGCGGTCACCAAGCTCAGGCAGAAGCACTCCGCCACGGTCAGGGTGGCAGAGGAACACAGGGGCACTGT
CGTTCAGGAGAACCCTGTAAGTTTGTTTGTGGTGAAGGCTGGAGTCCTCTCCCCACATATTCCAACTCTC
TGCAGGGAGGCGAAGGGTTCAACCTACAGCTCGGTCGCTGAGCTCGCCAGGATCCTCAAGAACCCCACCA
GACATCCTTCAATCATCAACAAACATATCAAGCAATACCAGGACGCACAAAGGTGTGGCGAGCAACCAGT
GAGCTCCCGACCAACTGCATCTGCTCCCTGCCCTCCTGCATC
EST_lsaA_evv_1227895 lsaAevv mixed_tissue Lepeophtheirus salmonis (Atlantic Norway) cDNA Lepeophtheirus salmonis cDNA clone lsaA_evv_501_085 3', mRNA sequence.
Sequence ID: gb|GW646341.1|Length: 896Number of Matches: 3
115 bits(245) 3e-25 48/105(46%) 71/105(67%) 0/105(0%) +2/-1

```
Query  131  ASVYQQHISGNLTTNSVKIQFISPQGDSVNQVTDLKNRVVITVYTVPGEQDLGQDGYQVF  310
            ++ Y+Q I GN ++ +V IQ+    G  ++Q+TD +    I    +PGE++L Q  YQ
Sbjct  824  SAYYRQDILGNSSSETVLIQYSLADGSLISQLTDFRMSSQIVRILIPGEEELDQPMYQGL  645

Query  311  CFVTAYTGDLIPTEAVTKLRQKHSATVRVAEEHRGTVVQENPVSL  445
            CFV++Y GDLIP EAV KLRQKH AT+R+AE+  G +VQ++ +SL
```

Sbjct 644 CFVSSYGGDLIPPEAVMKLRQKHPATLRIAEQDNGEIVQDSSLSL 510

>gi|295251958|gb|FQ001105.1|FQ001105 FQ001105 Pleurobrachia pileus library Pleurobrachia pileus cDNA clone SQ0AAA96YL18 5', mRNA sequence (Pleurobrachia pileus is a common genus of Ctenophora)

GCTAATATTGCTTGATTCACTTCTACAAGAGGTCCAAAGAGTCATAACATGTCCGGAGAATTCCCAGTTA
TCAAGTCTGCCCACTCCCTGGTCGCCAAGCACGTGACCAAGGAAAGATGGGACAAAGTCAAGGACATTGA
GACCAAGACCTGTGGATTCACCATTGCCAAGGCTTGTGCCTGTGCTATTGAGTTCGACAACCAGCACTGT
GGTATCTATGCTGGTGATTGGGATTGCTACAAAGATTTCTCTGAGATCTTTGATGCCATCATCCAGGACT
ACCATGGAATCAGCGCTGATGCCAAGCACACCTCTGACATGGATGCTACCAAGATCACCGGAAACATTGA
CCCCGCTGTCCCAGTTCACTCTACCCGTATCCGTGTTGGACGTAGCATTGATGGATTCGGTCTTTCCCCC
GGAATCACCAAGCAGCAGCGTATTGATGTTGAGAAGCTGATGAGCAGCGCTCTCTCCAAGCTGACTGAAG
ATCTTGCTGGTAGCTACTTTCCTCTTACTGGCATGGATGAGGCTGTCCGCCAACAGCTTGTTGATGACCA
CTTCCTCTTTGTCAGCGGAGACCGTAACCTTACTGTTGCTGGTATGGAGCGTGACTGGCCCGAGGGCCGT
GGAATCTTCCACAATGCTGCCAAGACCTTCCTTCTCTGGGTCAATGAGGAAGATCAGATGAGAATCATCT
CCATGGAAAAGGGAGGTGATGTCAAGGGTGTCTTTTCTCGCCTTGCCCGTGGAATCAAGGCTGTTGGAGA
CTCTGTCAAGGCTGAATGTGGCAAGGACTTTGCTTTGTCTGAGCAATACGGATACA

C5d140 C. finmarchicus C5 subtractive library, deep Calanus finmarchicus cDNA similar to arginine kinase, mRNA sequence.

Sequence ID: gb|ES387423.1|Length: 1106Number of Matches: 10

605 bits(1315) 1e-172 245/264(93%) 255/264(96%) 0/264(0%) +1/+1

```
Query  34   PKSHNMSGEFPVIKSAHSLVAKHVTKERWDKVKDIETKTCGFTIAKACACAIEFDNQHCG  213
            PK   MSGEFPVIKSAHSLVAKHVTKERWDKVKDI+TKTCGFTIAKACACAIEFDNQHCG
Sbjct  25   PKKRTMSGEFPVIKSAHSLVAKHVTKERWDKVKDIKTKTCGFTIAKACACAIEFDNQHCG  204

Query  214  IYAGDWDCYKDFSEIFDAIIQDYHGISADAKHTSDMDATKITGNIDPAVPVHSTRIRVGR  393
            IYAGDWDCYKDF+EIFDA+IQDYHGISA AKHTSDMDATK+TGNID AVPVHSTRIRVGR
Sbjct  205  IYAGDWDCYKDFAEIFDAVIQDYHGISATAKHTSDMDATKVTGNIDKAVPVHSTRIRVGR  384

Query  394  SIDGFGLSPGITKQQRIDVEKLMSSALSKLTEDLAGSYFPLTGMDEAVRQQLVDDHFLFV  573
            SIDG+GLSPGITKQQRIDVEKLMS+ALSK+T DLAGSY+PLTGMDE VRQQLVDDHFLFV
Sbjct  385  SIDGYGLSPGITKQQRIDVEKLMSTALSKMTGDLAGSYYPLTGMDETVRQQLVDDHFLFV  564

Query  574  SGDRNLTVAGMERDWPEGRGIFHNAAKTFLLWVNEEDQMRIISMEKGGDVKGVFSRLARG  753
            SGDRNLTVAGMERDWPEGRGIFHNAAKTFLLWVNEEDQ RIISM++GGDVKGVF RLARG
Sbjct  565  SGDRNLTVAGMERDWPEGRGIFHNAAKTFLLWVNEEDQTRIISMQQGGDVKGVFERLARG  744

Query  754  IKAVGDSVKAECGKDFALSEQYGY  825
            IKAVGDSVKAECGKDFALSEQYGY
Sbjct  745  IKAVGDSVKAECGKDFALSEQYGY  816
```

>gi|294433601|gb|FP988813.1|FP988813 FP988813 Pleurobrachia pileus library Pleurobrachia pileus cDNA clone SQ0AAA44YC10 5', mRNA sequence

TGCTTGATTCACTTCTACAAGAGATAGAAGAGACGCCAGCTTAAAAATGGGTAAGGAGAAGACTCATATT
AACATTGTCGTAATTGGTCACGTCGACTCTGGCAAATCTACCACCACCGGTCACTTGATCTACCAATGTG
GCGGTATCGACAAGAGAACCATCGAGAAGTTCGAGAAGGAAGCCCAGGAGATGGGCAAGGGCTCCTTCAA
GTATGCCTGGGTGTTGGACAAACTGAAGGCTGAGCGTGAGCGTGGTATCACCATTGACATTGCTCTCTGG
AAGTTTGAGACTCCCAAGTACTACGTGACCATCATCGACGCCCCCGGACACAGAGATTTCATCAAGAACA
TGATTACTGGAACCTCTCAGGCTGATTGTGGTGTACTTATCATCGCCTCTGGTGTTGGTGAGTTTGAGGC
CGGTATCTCCAAGAACGGACAGACTCGTGAACACGCTCTGCTTGCCTACACTCTGGGTGTCAAACAGCTC
ATCGTCGGAGTAAACAAGATGGACTCTACCGAGCCTCCCTACAGCAAGGACAGATTTGAGGAAATCCAAA
AGGAGGTGTCTGGCTTCATCAAGAAGGTTGGATATGACCCTAAGGCTGTTGCTTTCGTCCCCATTTCTGG
ATGGCACGGAGACAACATGCTGTCACCCTCTACCAACATGCCCTGGTTTGGAGGATGGCAGGTAGAGAGG
AAGGGAGAGAATGTTGCCAAGGGTAAGACTCTCCTTGAGGCTCTTGACGCCATCATTCCCCCACAGAGAC
CTACAGACAAGCCCCTCCGTCTTCCCCTGCAGGATGTCTACAAAATTGGAGGTATTGGAACAGTACCCGT
GGGCAGAAGTGAGACTGGTGTGTTGAAGC

EST_lsal_af_786024 lsalaf whole Lepeophtheirus salmonis (Pacific) cDNA Lepeophtheirus salmonis cDNA clone lsal_af_1_312_rev 5', mRNA sequence.

Sequence ID: gb|EY506720.1|Length: 927Number of Matches: 17

Range 1: 83 to 739GenBankGraphics Next Match Previous Match

489 bits(1063) 4e-164 199/219(91%) 207/219(94%) 0/219(0%) +2/+2

```
Query  47   MGKEKTHINIVVIGHVDSGKSTTTGHLIYQCGGIDKRTIEKFEKEAQEMGKGSFKYAWVL  226
            MGKEK HINIVVIGHVDSGKSTTTGHLIYQCGGIDKRTIEKFEKEAQEMGKGSFKYAWVL
Sbjct  83   MGKEKVHINIVVIGHVDSGKSTTTGHLIYQCGGIDKRTIEKFEKEAQEMGKGSFKYAWVL  262

Query  227  DKLKAERERGITIDIALWKFETPKYYVTIIDAPGHRDFIKNMITGTSQADCGVLIIASGV  406
            DKLKAERERGITIDIALWKFETPK+YVTIIDAPGHRDFIKNMITGTSQADCGVLIIASG
Sbjct  263  DKLKAERERGITIDIALWKFETPKFYVTIIDAPGHRDFIKNMITGTSQADCGVLIIASGT  442

Query  407  GEFEAGISKNGQTREHALLAYTLGVKQLIVGVNKMDSTEPPYSKDRFEEIQKEVSGFIKK  586
            GEFEAGISKNGQTREHALLAYTLGVKQLIVGVNKMDSTEPPYS+ RFEEI+KEVSG+IKK
Sbjct  443  GEFEAGISKNGQTREHALLAYTLGVKQLIVGVNKMDSTEPPYSQARFEEIKKEVSGYIKK  622

Query  587  VGYDPKAVAFVPISGWHGDNMLSPSTNMPWFGGWQVERK  703
            VGY+P  V FVPISGWHGDNML  S NM W+ GW++ RK
Sbjct  623  VGYNPATVPFVPISGWHGDNMLQTSPNMSWYKGWEITRK  739
```

Range 2: 740 to 901GenBankGraphics Next Match Previous Match First Match

108 bits(231) 4e-164 44/54(81%) 48/54(88%) 0/54(0%) +2/+2

```
Query  707  ENVAKGKTLLEALDAIIPPQRPTDKPLRLPLQDVYKIGGIGTVPVGRSETGVLK  868
            E  A G TLL+ALD+I+PP RPTDKPLRLPLQDVYKIGGIGTVPVGR ETG+LK
Sbjct  740  EGKASGTTLLDALDSIVPPARPTDKPLRLPLQDVYKIGGIGTVPVGRVETGILK  901
```

>gi|295257867|gb|FQ001353.1|FQ001353 FQ001353 Pleurobrachia pileus library Pleurobrachia pileus cDNA clone SQ0AAA96YA03 5', mRNA sequence
CTAATATTGCTTGATTCACTTCTACAAGAGGTCATGATGAAGCTTTTGCTTTTTTCTTCCTTGTTGGTTG
GATTGTCTTTGGCCGAGATTACCAAGGACGAAGGAGTGCTTGTCCTCACCGAGGCTAATTTCCAGGAGGC
TATTGATGCTCATGAGTTCTTGCTTGTTGAGTTTTATGCCCCTTGGTGTGGACACTGCAAGGCTCTTGCC
CCAGAGTATGTGAAGGCTGCCCAAACTTTGGCTGAGAAAGATTCTCCCATCAAGCTTGGAAAAGTTGATG
CCACGGAGGAGTCCAAGCTTGCAGAGAAGTTTGATGTCCGAGGCTACCCAACTCTTAAGTTCTACAAGAA
TGGCAAAGACCTGGAGTACAATGGTGGCAGAACTGCCGACACTATCGTGACCTGGCTCGAGAAGAAAACT
GGTCCAGCTGCTAAAACTCTGGAAAGTGGTGACGATGCCAAAGCCTTCATTGCTGACAATGATATTGCTG
TCATCGGGTTCTTTAAAGATCTCGAGTCTGCTGATGCCAAGGCTTTCCTTGATGCAGCTGGTTCCATGGA
TGACTATCCCTTTGCTATTTCTTCTGAAGCAGCTGTTCTGGAAGAATACAAAGTGGAGGCATCTGGTATT
GTGCTCTTCAAGAACTTTGATGAGGGACGCAATGATCTTGAGGGAGAAGTGACTGAGGAAGCCATCGTGA
AGTTTGTGTCTGGAAATGCTCTTCCCCTTGTGGTTGAGTTTAACCAGGAAACTGCTCAGAAAATATTTTC
TGGAGAAAATCAGTCTCACTTGCT
CF_WOO_18h11_SP6 Copepod Whole Organism, Normalized Calanus finmarchicus cDNA clone CF_WOO_18h11 5' similar to ABF18249.1 ER protein disulfide isomerase [Aedes aegypti]. Score=263.46, Expect=4.50E-69, mRNA sequence.
Sequence ID: gb|EL586118.1|Length: 654Number of Matches: 11
439 bits(962) 1e-122 203/217(94%) 211/217(97%) 0/217(0%) +1/+2

```
Query  1    LILLDSLLQEVMMKlllfssllvglslAEITKDEGVLVLTEANFQEAIDAHEFLLVEFYA  180
            L LLDSL  EVMMKLLLFSSLLVG+SLAEITKDEGVLVLTEANFQEAIDAHEFLLVEFYA
Sbjct  2    LYLLDSLP*EVMMKLLLFSSLLVGISLAEITKDEGVLVLTEANFQEAIDAHEFLLVEFYA  181

Query  181  PWCGHCKALAPEYVKAAQTLAEKDSPIKLGKVDATEESKLAEKFDVRGYPTLKFYKNGKD  360
            PWCGHCKALAPEYVKAAQTLAEK+SPIKLGKVDATEESKL EKFDVRGYPTLKF+KNGKD
Sbjct  182  PWCGHCKALAPEYVKAAQTLAEKESPIKLGKVDATEESKLGEKFDVRGYPTLKFFKNGKD  361

Query  361  LEYNGGRTADTIVTWLEKKTGPAAKTLESGDDAKAFIADNDIAVIGFFKDLESADAKAFL  540
            LEYNGGRTADTIVTWLEKKTGPAAKTLES D+AKAFIADNDIAVIGFFKDLESADAKAFL
Sbjct  362  LEYNGGRTADTIVTWLEKKTGPAAKTLESVDEAKAFIADNDIAVIGFFKDLESADAKAFL  541

Query  541  DAAGSMDDYPFAISSEAAVLEEYKVEASGIVLFKNFD  651
             AAGSMDDYPFA+++EAAVLEEYKVE+SGIVLFKNFD
Sbjct  542  GAAGSMDDYPFALATEAAVLEEYKVESSGIVLFKNFD  652
```

>gi|89501138|gb|DY656934.1|DY656934 Cm_mx1_86f06_SP6 Green Shore Crab Multiple Tissue, Normalized Carcinus maenas cDNA clone Cm_mx1_86f06 5' similar to Similar to ref|NP_524469.2| Proteasome 26S subunit subunit 4 ATPase CG5289-PA [Drosophila. Score = 362 bits (930), Expect = 6e-99, mRNA sequence. Note: essentially identical to Calanus finmarchicus cDNA EH667055
ATAGCTAATATTGCTTGATTCACTTCTACAAGAGAGTTTCGATGGGAAACCAACAATCTGGGTCTGGAGG

TGGAGCAGGGAAGGATAAGGGAGAGCAGAAGGAGAAGAAGAAATACGAGCCTCCTGTGCCAACCAGGGTT
GGCAAGAAGCGCAAGCGCGTTAAGGGACCCGATGCTGCTAACAAGCTGCCCGCTGTCACCCCCCACACCA
AGTGCAGGCTGAGGTTGCTTAAGCAGGAGAGGATCAAGGACTATCTTCTCATGGAGGAGGAGTTCATCAG
GAACCAGGAGAGACTGAAGCCACAGGAGGAGAAGAACGAAGAGGAGCGCAGCAAGGTCGATGACCTGAGG
GGAACACCAATGAGTGTTGGTAACTTGGAGGAGATCATAGATGACAACCACGCTATCGTCTCCACCAGCG
TGGGCAGTGAGCACTACGTGTCCATCCTCAGCTTTGTGGACAAGGATCAGTTGGAGCCCGGCTGCAGCGT
CCTTCTCAACCACAAGGTGCATGCTGTAGTTGGTGTTCTATCTGATGACACTGACCCTATGGTGACTGTG
ATGAAGCTGGAGAAAGCTCCTACTGAGAGTTATGCTGATATTGGTGGTCTGGACACCCAGATCCAAGAGA
TCAAGGAGAGCGTTGAGCTGCCCCTGACCCACCCTGAGTATTACGAGGAGATGGGTATCAAGCCACCCAA
GGGTGTCATCCTGTACGGTCCTCCTGGCACTGGCA
(>gi|124246340|gb|EH667055.1|EH667055 CF_WO0_09e06_SP6 Copepod Whole Organism, Normalized Calanus finmarchicus cDNA clone CF_WO0_09e06 5' similar to gb|ABF18346.1| 26S proteasome regulatory chain 4 - Aedes aegypti. Score = 368 bits (945), Expect = e-100, mRNA sequence
ATGCTTTAAATTGCTTGATTCACTTCTACAAGAGAGTTTCGATGGGAAACCAACAATCTGGGTCTGGAGG
TGGAGCAGGGAAGGATAAGGGAGAGCAGAAGGAGAAGAAGAAATACGAGCCTCCTGTGCCAACCAGGGTT
GGCAAGAAGCGCAAGCGCGTTAAGGGACCCGATGCTGCTAACAAGCTGCCCGCTGTCACCCCCCACACCA
AGTGCAGGCTGAGGCTGCTTAAGCAGGAGAGGATCAAGGACTATCTCCTCATGGAGGAGGAGTTCATCAG
GAACCAGGAGAGACTGAAGCCACAGGAGGAGAAGAACGAAGAGGAGCGCAGCAAGGTCGATGACCTGAGG
GGAACACCAATGAGTGTTGGTAACTTGGAGGAGATCATAGATGACAACCACGCCATCGTCTCCACCAGCG
TGGGCAGTGAGCACTACGTGTCCATCCTCAGCTTTGTGGACAAGGACCAGTTGGAGCCCGGCTGCAGCGT
TCTTCTCAACCACAAGGTGCATGCTGTTGTTGGTGTTCTATCTGATGACACTGACCCTATGGTGACTGTG
ATGAAGCTGGAGAAAGCTCCTACTGAGAGTTACGCTGATATTGGTGGTCTGGACACCCAGATCCAAGAGA
TCAAGGAGAGCGTGGAGCTGCCCCTGACCCACCCTGAGTATTACGAGGAGATGGGTATCAAGCCACCCAA
GGGTGTCATCCTGTACGGTCCTCCTGGCACTGGCAAGACCCTCC)
179 bits(385) 9e-142 91/92(99%) 92/92(100%) 0/92(0%) -2/-2
Range 2: 9 to 284GenBankGraphics Next Match Previous Match First Match

```
Query  284  VPDELLLHEKIVLDPLLLKQPQPALGVGGDSGQLVSSIGSLNALALLANPGWHRRlvfll  105
            VPDELLLHE+IVLDPLLLKQPQPALGVGGDSGQLVSSIGSLNALALLANPGWHRRLVFLL
Sbjct  284  VPDELLLHEEIVLDPLLLKQPQPALGVGGDSGQLVSSIGSLNALALLANPGWHRRLVFLL  105

Query  104  llllsliLPCSTSRPRLLVSHRNSLVEVNQAI  9
            LLLLSLILPCSTSRPRLLVSHRNSLVEVNQAI
Sbjct  104  LLLLSLILPCSTSRPRLLVSHRNSLVEVNQAI  9
```

Range 3: 171 to 734GenBankGraphics Next Match Previous Match First Match
447 bits(973) 1e-136 188/188(100%) 188/188(100%) 0/188(0%) +3/+3

```
Query  171  DAANKLPAVTPHTKCRLRLLKQERIKDYLLMEEEFIRNQERLKPQEEKNEEERSKVDDLR  350
            DAANKLPAVTPHTKCRLRLLKQERIKDYLLMEEEFIRNQERLKPQEEKNEEERSKVDDLR
Sbjct  171  DAANKLPAVTPHTKCRLRLLKQERIKDYLLMEEEFIRNQERLKPQEEKNEEERSKVDDLR  350
```

Query 351 GTPMSVGNLEEIIDDNHAIVSTSVGSEHYVSILSFVDKDQLEPGCSVLLNHKVHAVVGVL 530
             GTPMSVGNLEEIIDDNHAIVSTSVGSEHYVSILSFVDKDQLEPGCSVLLNHKVHAVVGVL
Sbjct 351 GTPMSVGNLEEIIDDNHAIVSTSVGSEHYVSILSFVDKDQLEPGCSVLLNHKVHAVVGVL 530

Query 531 SDDTDPMVTVMKLEKAPTESYADIGGLDTQIQEIKESVELPLTHPEYYEEMGIKPPKGVI 710
             SDDTDPMVTVMKLEKAPTESYADIGGLDTQIQEIKESVELPLTHPEYYEEMGIKPPKGVI
Sbjct 531 SDDTDPMVTVMKLEKAPTESYADIGGLDTQIQEIKESVELPLTHPEYYEEMGIKPPKGVI 710

Query 711 LYGPPGTG 734
             LYGPPGTG
Sbjct 711 LYGPPGTG 734

Range 4: 11 to 76GenBankGraphics Next Match Previous Match First Match
60.2 bits(125) 1e-136 22/22(100%) 22/22(100%) 0/22(0%) +2/+2
Query 11 LLDSLLQESFDGKPTIWVWRWS 76
           LLDSLLQESFDGKPTIWVWRWS
Sbjct 11 LLDSLLQESFDGKPTIWVWRWS 76

>gi|89500951|gb|DY656747.1|DY656747 Cm_mx1_84f04_SP6 Green Shore Crab Multiple Tissue, Normalized Carcinus maenas cDNA clone Cm_mx1_84f04 5' similar to Similar to ref|NP_524469.2| Proteasome 26S subunit subunit 4 ATPase CG5289-PA [Drosophila. Score = 334 bits (856), Expect = 2e-90, mRNA sequence. Note: essentially identical to DY656934, and identical to Calanus finmarchicus cDNA EH667055)
ATAGCTAATATTGCTTGATTCACTTCTACAAGAGAGTTTCGATGGGAAACCAACAATCTGGGTCTGGAGG
TGGAGCAGGGAAGGATAAGGGAGAGCAGAAGGAGAAGAAGAAATACGAGCCTCCTGTGCCAACCAGGGTT
GGCAAGAAGCGCAAGCGCGTTAAGGGACCCGATGCTGCTAACAAGCTGCCCGCTGTCACCCCCCACACCA
AGTGCAGGCTGAGGTTGCTTAAGCAGGAGAGGATCAAGGACTATCTTCTCATGGAGGAGGAGTTCATCAG
GAACCAGGAGAGACTGAAGCCACAGGAGGAGAAGAACGAAGAGGAGCGCAGCAAGGTCGATGACCTGAGG
GGAACACCAATGAGTGTTGGTAACTTGGAGGAGATCATAGATGACAACCACGCTATCGTCTCCACCAGCG
TGGGCAGTGAGCACTACGTGTCCATCCTCAGCTTTGTGGACAAGGATCAGTTGGAGCCCGGCTGCAGCGT
CCTTCTCAACCACAAGGTGCATGCTGTAGTTGGTGTTCTATCTGATGACACTGACCCTATGGTGACTGTG
ATGAAGCTGGAGAAAGCTCCTACTGAGAGTTATGCTGATATTGGTGGTCTGGACACCCAGATCCAAGAGA
TCAAGGAGAGCGTTGAGCTGCCCCTGACCCACCCTGAGTATTACGAGGAGATGGGTATCAAGCCA

Range 2: 9 to 284GenBankGraphics Next Match Previous Match First Match
179 bits(385) 6e-130 91/92(99%) 92/92(100%) 0/92(0%) -1/-2
Query 284 VPDELLLHEKIVLDPLLLKQPQPALGVGGDSGGQLVSSIGSLNALALLANPGWHRRlvfll 105
             VPDELLLHE+IVLDPLLLKQPQPALGVGGDSGGQLVSSIGSLNALALLANPGWHRRLVFLL
Sbjct 284 VPDELLLHEEIVLDPLLLKQPQPALGVGGDSGGQLVSSIGSLNALALLANPGWHRRLVFLL 105

Query 104 llllsliLPCSTSRPRLLVSHRNSLVEVNQAI 9

LLLLSLILPCSTSRPRLLVSHRNSLVEVNQAI
Sbjct 104 LLLLSLILPCSTSRPRLLVSHRNSLVEVNQAI 9

Range 3: 171 to 695GenBankGraphics Next Match Previous Match First Match
413 bits(899) 1e-126 175/175(100%) 175/175(100%) 0/175(0%) +3/+3
Query 171 DAANKLPAVTPHTKCRLRLLKQERIKDYLLMEEEFIRNQERLKPQEEKNEEERSKVDDLR 350
DAANKLPAVTPHTKCRLRLLKQERIKDYLLMEEEFIRNQERLKPQEEKNEEERSKVDDLR
Sbjct 171 DAANKLPAVTPHTKCRLRLLKQERIKDYLLMEEEFIRNQERLKPQEEKNEEERSKVDDLR 350

Query 351 GTPMSVGNLEEIIDDNHAIVSTSVGSEHYVSILSFVDKDQLEPGCSVLLNHKVHAVVGVL 530
GTPMSVGNLEEIIDDNHAIVSTSVGSEHYVSILSFVDKDQLEPGCSVLLNHKVHAVVGVL
Sbjct 351 GTPMSVGNLEEIIDDNHAIVSTSVGSEHYVSILSFVDKDQLEPGCSVLLNHKVHAVVGVL 530

Query 531 SDDTDPMVTVMKLEKAPTESYADIGGLDTQIQEIKESVELPLTHPEYYEEMGIKP 695
SDDTDPMVTVMKLEKAPTESYADIGGLDTQIQEIKESVELPLTHPEYYEEMGIKP
Sbjct 531 SDDTDPMVTVMKLEKAPTESYADIGGLDTQIQEIKESVELPLTHPEYYEEMGIKP 695

Range 4: 11 to 76GenBankGraphics Next Match Previous Match First Match
60.2 bits(125) 1e-126 22/22(100%) 22/22(100%) 0/22(0%) +2/+2
Query 11 LLDSLLQESFDGKPTIWVWRWS 76
LLDSLLQESFDGKPTIWVWRWS
Sbjct 11 LLDSLLQESFDGKPTIWVWRWS 76

>gi|89501263|gb|DY657059.1|DY657059 Cm_mx1_88a03_SP6 Green Shore Crab Multiple Tissue, Normalized Carcinus maenas cDNA clone Cm_mx1_88a03 5' similar to Similar to emb|CAI64731.1| aurora/Ipl1p-related kinase - Marthasterias glacialis. Score = 164 bits (415), Expect = 3e-39, mRNA sequence
ATGCTTGATTCACTTCTACAAGAGAATTTAGGAAAAGCTTTGCAATGACCAGTCGTCCCCCCCTCCAGTC
CACTGCTAACCTGCCCCTCAAACAGCCTATTGCACCACCAAAGAGACCAGAAGCCATCCCTGGAGCTGTC
AAACCAAGTTCAGGATCAAAACCAGTTCCAGTTTCCAACCCTGGGTCTGTCAAGCCTGGCTCTGGTTCTA
AACCGGTTCCACTGACCAAGCCATCAGGACTTGCCAAGCCTGGTTCTGGGTCCAAGCCAACAGTGATGGC
CAAACCTGGACCAGTTTCTAAACAAGTCCAAAGTTCCAAACCAACAACTTCAACGTCTTCCAACCCTGAG
TCTGCCCCCAACCCACCCCCGGGTGGTTCCCTTAACGGGCTGCCTAAAATGGGCCCAAATCACAGATGGA
AATTAGAAGATTTCGACATTGGTCGACCCCTGGGGAAGGGTAAGTTTGGCAACGTGTACTTAGCCAGGGA
GAAGGCCAGCAAGTACATCGTGGCCCTGAAGGTTCTCTTCAAGTCTCAGTTACAGAAGGCCCAAGTGGAG
CACCAGCTTAGACGTGAAATAGAGATACAGTCCCATCTTAGACACAACCACATTTTGAGATTGTTTGGAT
ACTTTTATGATGAGAGCAGAGTCTACCTCATCTTGGAGTTTGCTCCTA
EST_lbra_evt_1109920 lbraevt mixed_tissue Lernaeocera branchialis cDNA Lernaeocera branchialis cDNA clone lbra_evt_014_160 5', mRNA sequence.
Sequence ID: gb|GO421219.1|Length: 896Number of Matches: 4
188 bits(405) 3e-47 77/88(88%) 83/88(94%) 0/88(0%) +3/+2

```
Query  414  RWKLEDFDIGRPLGKGKFGNVYLAREKASKYIVALKVLFKSQLQKAQVEHQLRREIEIQS  593
            RWKL DFDIGRPLGKGKFGNVYLAREK SK+IVALKV+FKSQLQKA VEHQLRREIEIQS
Sbjct  284  RWKLNDFDIGRPLGKGKFGNVYLAREKKSKFIVALKVMFKSQLQKANVEHQLRREIEIQS  463

Query  594  HLRHNHILRLFGYFYDESRVYLILEFAP  677
            HLRH +ILRL+ YFYDE+RVYLILE+AP
Sbjct  464  HLRHPNILRLYAYFYDEARVYLILEYAP  547
```

>gi|89500447|gb|DY656243.1|DY656243 Cm_mx1_78d05_SP6 Green Shore Crab Multiple Tissue, Normalized Carcinus maenas cDNA clone Cm_mx1_78d05 5', mRNA sequence

```
CCAGCTAATATTTGCTTGATTCACTTCTACAAGAGTTCCTATCCTACAGACCAGAACTGTCCACGATGTA
TACAGAGTACTTGAACATCATCGTGTATGACTATCTCTCACAAGTGGATCCTAAACTGGCCAAAAAGTTC
AAGAAGGAAGCAAACGTGACAGAAGAGCTTCCTCCAGGCTCTCCAGCTATTGGTGATGTGGTCAAATTCT
TCAACGAAACATCCCCCAAGAAACTGAAGAGAGAACTTGAACCTGCCGAGGAAGAAGCTGTTGAGCCCAA
GAAGGATAAAAAGGCCAAGAAAGCTGAAAAGAATGCGGCAAGAGCAGTCGCTGTGAAGGAATCAGCTGAC
GATGAAGTTCCTGCTCAGAAAAGTAGGAAGGCGAAGAAAGCCAAGAAGGACTCTGCTTCTGCTGCTGCAG
TTGAAGAAGTTGCAGTCGTCGAGGAAGAGGAAGCTGGATCTGCAAAGAAAAGCAAGAAGTCTAAAAAAGC
CAAAAAGGATTAGAACCAGTTGCCTATTCCAGCCAGCAGATGAAGCTTTGTTCTCTTTAATCAAATAAGC
TATGCATTGTTATCTATTTGAAATAATATGTGTATTTGGCTA
```

EST_crog_evp_900029 crog_evp Caligus rogercresseyi cDNA clone crog_evp_525_221_fwd 5', mRNA sequence.

Sequence ID: gb|FK886399.1|Length: 862Number of Matches: 2

Range 1: 180 to 278GenBankGraphics Next Match Previous Match

37.7 bits(76) 0.013 15/33(45%) 22/33(66%) 0/33(0%) +3/+3

```
Query  114  VDPKLAKKFKKEANVTEELPPGSPAIGDVVKFF  212
            V  K+A+KFKK   +++ LP GSP I ++V  F
Sbjct  180  VSDKMAEKFKKTTKLSQTLPKGSPTITELVDDF  278
```

Range 2: 135 to 170GenBankGraphics Next Match Previous Match First Match

21.7 bits(41) 0.013 8/12(67%) 9/12(75%) 0/12(0%) +3/+3

```
Query  72   TEYLNIIVYDYL  107
            T  LN +VYDYL
Sbjct  135  TTKLNALVYDYL  170
```

>gi|83279733|gb|DV943741.1|DV943741 Cm_mx1_05d07_SP6 Green Shore Crab Multiple Tissue, Normalized Carcinus maenas cDNA clone Cm_mx1_05d07 5' similar to Similar to emb|CAG00121.1| unnamed protein product - Tetraodon nigroviridis. Score = 325 bits (833), Expect = 8e-88, mRNA sequence. Note: essentially identical to XP_001607854 Calanus finmarchicus cDNA clone CF_W01_53c08

```
CAAGCTATTACTTGCTTGATTCACTTCTACAAGAGATCCAAACTTCTGTCTCACAATGTCCAGAGGAAGC
AGCGCAGGGTTTGACCGTCATATCACCATCTTCTCCCCTGAGGGCAGGCTTTACCAGGTCGAGTACGCCT
```

TCAAGGCCATCAACCAGGGGGCCACCACCTCTGTGGCTGTCAAGGGAGTCGACACAGCAGCATGCGCCAC
CCTCAAGAAGGTCCCTGACAAGCTCCTTGACGCCAAGACTGTGACCAACCTTTACCAGCTGACGGACACC
ATCGGGTGCGTGATGACCGGCATGGTGGCGGACAGCAGGTCCCAGGTGCAGAGGGCCAGGTATGAGGCCG
CCAACTGGAAGTACAAGTACGGATACGAGATCCCCGTCGATATGCTCTGCAGAAGGCTCAGTGACATTTC
CCAGGTTTACACCCAAAATGCTGAGATGCGCCCACTGGGCTGCTCGATGATTCTGATCGCCTTTGATGAC
GAGATTGGAGCTTGTGTTTACAAAACTGATCCAGCTGGCTACTACTCGGCCTTCAAGGCTTGCTCAGTGG
GGTCCAAGCAGACTGAGGCTAATTCATTCCTGGAGAAGAAGTTCAAGAAGAAGGAAGATTACNACCACGA
CGAGACAATCCAGATTGCCATCAACTGCTTGTCTACCATCCTCTCCGC

CF_WO1_53c08_SP6 Copepod Whole Organism, Normalized Calanus finmarchicus cDNA clone CF_WO1_53c08 5’ similar to ref|XP_001607854.1| PREDICTED: similar to proteasome subunit alpha type [Nasonia. Score = 217 bits (553), Expect = 7e-55, mRNA sequence.

Sequence ID: gb|GR410818.1|Length: 739Number of Matches: 24

Range 1: 3 to 395GenBankGraphics Next Match Previous Match

309 bits(669) 5e-134 130/131(99%) 130/131(99%) 0/131(0%) +2/+3

```
Query  41   NFCLTMSRGSSAGFDRHITIFSPEGRLYQVEYAFKAINQGATTSVAVKGVDTAACATLKK  220
            NFCLTMSRGSSAGFDRHITIFSPEGRLYQVEYAFKAINQGATTSVAVKGVDTAACATLKK
Sbjct  3    NFCLTMSRGSSAGFDRHITIFSPEGRLYQVEYAFKAINQGATTSVAVKGVDTAACATLKK  182

Query  221  VPDKLLDAKTVTNLYQLTDTIGCVMTGMVADSRSQVQRARYEAANWKYKYGYEIPVDMLC  400
            VPDKLLDAKTVTNLYQLTDTIGCVMTGMVADSRSQVQRARYEAANWKYKYGYEIPVDMLC
Sbjct  183  VPDKLLDAKTVTNLYQLTDTIGCVMTGMVADSRSQVQRARYEAANWKYKYGYEIPVDMLC  362

Query  401  RRLSDISQVYT  433
            RRLSDISQV T
Sbjct  363  RRLSDISQVRT  395
```

Range 2: 433 to 588GenBankGraphics Next Match Previous Match First Match

129 bits(276) 5e-134 52/52(100%) 52/52(100%) 0/52(0%) +2/+1

```
Query  422  QVYTQNAEMRPLGCSMILIAFDDEIGACVYKTDPAGYYSAFKACSVGSKQTE  577
            QVYTQNAEMRPLGCSMILIAFDDEIGACVYKTDPAGYYSAFKACSVGSKQTE
Sbjct  433  QVYTQNAEMRPLGCSMILIAFDDEIGACVYKTDPAGYYSAFKACSVGSKQTE  588
```

>gi|80465641|gb|DV642724.1|DV642724 Cm_mx0_97e08_SP6 Green Shore Crab Multiple Tissue, Normalized Carcinus maenas cDNA clone Cm_mx0_97e08 5’ similar to emb|CAG00121.1| unnamed protein product - Tetraodon nigroviridis. Score = 329 bits (844), Expect = 4e-89, mRNA sequence. Note: essentially identical to DV943741, and to XP_001607854 Calanus finmarchicus cDNA clone CF_WO1_53c08

CAAGCTATTACTTGCTTGATTCACTTCTACAAGAGATCCAAACTTCTGTCTCACAATGTCCAGAGGAAGC
AGCGCAGGGTTTGACCGTCATATCACCATCTTCTCCCCTGAGGGCAGGCTTTACCAGGTCGAGTACGCCT
TCAAGGCCATCAACCAGGGGGCCACCACCTCTGTGGCTGTCAAGGGAGTCGACACAGCAGCATGCGCCAC
CCTCAAGAAGGTCCCTGACAAGCTCCTTGACGCCAAGACTGTGACCAACCTTTACCAGCTGACGGACACC

ATCGGGTGCGTGATGACCGGCATGGTGGCGGACAGCAGGTCCCAGGTGCAGAGGGCCAGGTATGAGGCCG
CCAACTGGAAGTACAAGTACGGATACGAGATCCCCGTCGATATGCTCTGCAGAAGGCTCAGTGACATTTC
CCAGGTTTACACCCAAAATGCTGAGATGCGCCCACTGGGCTGCTCGATGATTCTGATCGCCTTTGATGAC
GAGATTGGAGCTTGTGTTTACAAAACTGATCCAGCTGGCTACTACTCGGCCTTCAAGGCTTGCTCAGTGG
GGTCCAAGCAGACTGAGGCTAATTCATTCCTGGAGAAGAAGTTCAAGAAGAAGGAAGATTACNACCACGA
CGAGACAATCCAGATTGCCATCAACTGCTTGTCTACCATCCTCTCCGCAGACTTCAGCCATCT
CF_WO1_53c08_SP6 Copepod Whole Organism, Normalized Calanus finmarchicus cDNA clone
CF_WO1_53c08 5’ similar to ref|XP_001607854.1| PREDICTED: similar to proteasome subunit alpha type [Nasonia. Score = 217 bits (553), Expect = 7e-55, mRNA sequence.
Sequence ID: gb|GR410818.1|Length: 739Number of Matches: 24
Range 1: 3 to 395GenBankGraphics Next Match Previous Match
309 bits(669) 5e-134 130/131(99%) 130/131(99%) 0/131(0%) +2/+3

```
Query  41   NFCLTMSRGSSAGFDRHITIFSPEGRLYQVEYAFKAINQGATTSVAVKGVDTAACATLKK  220
            NFCLTMSRGSSAGFDRHITIFSPEGRLYQVEYAFKAINQGATTSVAVKGVDTAACATLKK
Sbjct  3    NFCLTMSRGSSAGFDRHITIFSPEGRLYQVEYAFKAINQGATTSVAVKGVDTAACATLKK  182

Query  221  VPDKLLDAKTVTNLYQLTDTIGCVMTGMVADSRSQVQRARYEAANWKYKYGYEIPVDMLC  400
            VPDKLLDAKTVTNLYQLTDTIGCVMTGMVADSRSQVQRARYEAANWKYKYGYEIPVDMLC
Sbjct  183  VPDKLLDAKTVTNLYQLTDTIGCVMTGMVADSRSQVQRARYEAANWKYKYGYEIPVDMLC  362

Query  401  RRLSDISQVYT  433
            RRLSDISQV T
Sbjct  363  RRLSDISQVRT  395
```

Range 2: 433 to 588GenBankGraphics Next Match Previous Match First Match
129 bits(276) 5e-134 52/52(100%) 52/52(100%) 0/52(0%) +2/+1

```
Query  422  QVYTQNAEMRPLGCSMILIAFDDEIGACVYKTDPAGYYSAFKACSVGSKQTE  577
            QVYTQNAEMRPLGCSMILIAFDDEIGACVYKTDPAGYYSAFKACSVGSKQTE
Sbjct  433  QVYTQNAEMRPLGCSMILIAFDDEIGACVYKTDPAGYYSAFKACSVGSKQTE  588
```

>gi|84411659|gb|DW250273.1|DW250273 Cm_mx1_32b03_SP6 Green Shore Crab Multiple Tissue, Normalized Carcinus maenas cDNA clone Cm_mx1_32b03 5’, mRNA sequence. Note: essentially identical to DW250218---No hit
AGCTAATATTGCTTGATTCACTTCTACAAGAGATAATATTATCCCACCATGTCTCTCTCCTCCCACTATG
CCCAGACCCTGCCCATCACACAGAGGGTCAAGTTTAACAGAGACTTTTCTAAATCTTACAGAGATACAGA
CAGTTCTAACAACAAGCAGGACTGGGAGAACGTGAAGTTCAACAACCTGAAGTACAGTCTACCACAACAA
ACAACCACGGGCAGTGCGCGCAGGGCAGAGATGTTTTTAAAACATGGAATATTGAAATGAGAATAAATGA
TATTTAACATCTG

>gi|84411398|gb|DW250218.1|DW250218 Cm_mx1_31e08_SP6 Green Shore Crab Multiple Tissue, Normalized Carcinus maenas cDNA clone Cm_mx1_31e08 5’, mRNA sequence---No hit

ATTCCCGGGAGCTAATATTGCTTGATTCACTTCTACAAGAGATAATATTATCCCACCATGTCTCTCTCCT
CCCACTATGCCCAGACCCTGCCCATCACACAGAGGGTCAAGTTTAACAGAGACTTTTCTAAATCTTACAG
AGATACAGACAGTTCTAACAACAAGCAGGACTGGGAGAACGTGAAGTTCAACAACCTGAAGTACAGTCTA
CCACAACAAACAACCACGGGCAGTGCGCGCAGGGCAGAGATGTTTTTAAAACATGGAATATTGAAATGAG
AATAAATGATATTTAACATCTG

>gi|62428079|gb|DN796273.1|DN796273 Cm_mx0_60h12_SP6 Green Shore Crab Multiple Tissue, Normalized Carcinus maenas cDNA clone Cm_mx0_60h12 5’ similar to gb|EAL62546.1| hypothetical protein DDB0188508 - Dictyostelium discoideum. Score = 41.2 bits (95), Expect = 0.019, mRNA sequence---No hit
ATTTGCTTGATTCACTTCTACAAGAGATCAATCTCAAAATGGCTTCAAATTCTTCTCTAACCTTACCGAC
TTTTGATGACCCTCCTGCTTCCCCACTTCAAAAGATTATCTCCAGACTGCCCTCAGGCCTGAGTATCAAG
AGAGTCTCCCTTGCCAGTCCTGCCAGCTCCCCCAGTCCCTCACACAAGAGGCTGAGAGCAGACAGCAGTA
TCCTCTCCTCCCCGGACATGAGACGAGAGGTCTTGGTCAAGAAAGTCAAGCAGAAGCTGTCCTGTGGTAG
TACTATCAGTTTGGTAGGTACCAAGATGGTAAAAGATGCTACATTGAAACTAGATGACAGTGTCGAAATT
ATTACCGATACAAATGAAAATGAATCCCTTAGCCTTGAAGAACGTGATGTTAAAACAAGCCAACATGTTG
GAGATGAAGCGGAAGTCAATCAAAACAAGAACAAACCAAATGATAATGTCAAATCAACAACAGAAGAACT
GAAGCCAACTGAAGCCAAGCAGGTTCAACAACCGAGAACAAGCAAGTCAAAGAAAATTGAAATAAAATGG
ACTGAGGTAAAAAGAAATAAAAGGAAAAATAGGTTCTATATTTTTGGGTCTACAGTTG

>gi|62427959|gb|DN796153.1|DN796153 Cm_mx0_59d10_SP6 Green Shore Crab Multiple Tissue, Normalized Carcinus maenas cDNA clone Cm_mx0_59d10 5’ similar to gb|AAD13639.1| blood-stage membrane protein Ag-1 - Plasmodium yoelii. Score = 42.7 bits (99), Expect = 0.009, mRNA sequence---No hit. Essentially identical to DN796273.
ATTTGCTTGATTCACTTCTACAAGAGATCAATCTCAAAATGGCTTCAAATTCTTCTCTAACCTTACCGAC
TTTTGATGACCCTCCTGCTTCCCCACTTCAAAAGATTATCTCCAGACTGCCCTCAGGCCTGAGTATCAAG
AGAGTCTCCCTTGCCAGTCCTGCCAGCTCCCCCAGTCCCTCACACAAGAGGCTGAGAGCAGACAGCAGTA
TCCTCTCCTCCCCGGACATGAGACGAGAGGTCTTGGTCAAGAAAGTCAAGCAGAAGCTGTCCTGTGGTAG
TACTATCAGTTTGGTAGGTACCAAGATGGTAAAAGATGCTACATTGAAACTAGATGACAGTGTCGAAATT
ATTACCGATACAAATGAAAATGAATCCCTTAGCCTTGAAGAACGTGATGTTAAAACAAGCCAACATGTTG
GAGATGAAGCGGAAGTCAATCAAAACAAGAACAAACCAAATGATAATGTCAAATCAACAACAGAAGAACT
GAAGCCAACTGAAGCCAAGCAGGTTCAACAACCGAGAACAAGCAAGTCAAAGAAAATTGAAATAAAATGG
ACTGAGGTAAAAAGAAATAAAAGGAAAAATAGGTTCTATATTTTTGGGTCTACAGTTGAAGGGGAAAAAG
ACGAGGTGACAGTAGAGAAAGCTGAAAATAATCAGAAAGTTCCACGTGTCATTAATGGTCGCAGGCCCTA
CATTGTGGTTCCAGGCGTTGAATATGACC

>gi|62116058|gb|DN739048.1|DN739048 Cm_mx0_52f12_SP6 Green Shore Crab Multiple Tissue, Normalized Carcinus maenas cDNA clone Cm_mx0_52f12 5’ similar to gb|EAL62546.1| hypothetical protein DDB0188508 - Dictyostelium discoideum. Score = 41.2 bits (95), Expect = 0.019, mRNA sequence---No hit. Essentially identical to DN796273 and DN796153
ATTTGCTTGATTCACTTCTACAAGAGATCAATCTCAAAATGGCTTCAAATTCTTCTCTAACCTTACCGAC

TTTTGATGACCCTCCTGCTTCCCCACTTCAAAAGATTATCTCCAGACTGCCCTCAGGCCTGAGTATCAAG
AGAGTCTCCCTTGCCAGTCCTGCCAGCTCCCCCAGTCCCTCACACAAGAGGCTGAGAGCAGACAGCAGTA
TCCTCTCCTCCCCGGACATGAGACGAGAGGTCTTGGTCAAGAAAGTCAAGCAGAAGCTGTCCTGTGGTAG
TACTATCAGTTTGGTAGGTACCAAGATGGTAAAAGATGCTACATTGAAACTAGATGACAGTGTCGAAATT
ATTACCGATACAAATGAAAATGAATCCCTTAGCCTTGAAGAACGTGATGTTAAAACAAGCCAACATGTTG
GAGATGAAGCGGAAGTCAATCAAAACAAGAACAAACCAAATGATAATGTCAAATCAACAACAGAAGAACT
GAAGCCAACTGAAGCCAAGCAGGTTCAACAACCGAGAACAAGCAAGTCAAAGAAAATTGAAATAAAATGG
ACTGAGGTAAAAAGAAATAAAAGGAAAAATAGGTTCTATATTTTTGGGTCTACAGTT

>gi|62115913|gb|DN738903.1|DN738903 Cm_mx0_50h01_SP6 Green Shore Crab Multiple Tissue, Normalized Carcinus maenas cDNA clone Cm_mx0_50h01 5' similar to gb|AAD13639.1| blood-stage membrane protein Ag-1 - Plasmodium yoelii. Score = 42.7 bits (99), Expect = 0.008, mRNA sequence---No hit. Essentially identical to DN796273, DN796153 and DN739048
ATTTGCTTGATTCACTTCTACAAGAGATCAATCTCAAAATGGCTTCAAATTCTTCTCTAACCTTACCGAC
TTTTGATGACCCTCCTGCTTCCCCACTTCAAAAGATTATCTCCAGACTGCCCTCAGGCCTGAGTATCAAG
AGAGTCTCCCTTGCCAGTCCTGCCAGCTCCCCCAGTCCCTCACACAAGAGGCTGAGAGCAGACAGCAGTA
TCCTCTCCTCCCCGGACATGAGACGAGAGGTCTTGGTCAAGAAAGTCAAGCAGAAGCTGTCCTGTGGTAG
TACTATCAGTTTGGTAGGTACCAAGATGGTAAAAGATGCTACATTGAAACTAGATGACAGTGTCGAAATT
ATTACCGATACAAATGAAAATGAATCCCTTAGCCTTGAAGAACGTGATGTTAAAACAAGCCAACATGTTG
GAGATGAAGCGGAAGTCAATCAAAACAAGAACAAACCAAATGATAATGTCAAATCAACAACAGAAGAACT
GAAGCCAACTGAAGCCAAGCAGGTTCAACAACCGAGAACAAGCAAGTCAAAGAAAATTGAAATAAAATGG
ACTGAGGTAAAAAGAAATAAAAGGAAAAATAGGTTCTATATTTTTGGGTCTACAGTTGAAGGGGAAAAAG
ACGAGGTGACAGTAGAGAAAGCTGAAAATAATCAGAAGGTTCCACGTGTCATTAATGGT

Table S3b. nblast of CopepodSL-like1a CCAAGTAAATAATACGTGTCTCTGACAAAAATCAAG and CopepodSL-like1b CCAAGTAAATAATACGTGTCTCTGACTAATAATCAAG against DDBJ/EMBL/GenBank nr/nt, EST and TSA databases.
Note 1: Database: nt+tsa+est; 106,461,619 sequences; 119,623,300,248 total letters. Matrix: blastn matrix: 1 -3; Gap Penalties: Existence: 5, Extension: 2.
Note 2: Total hits 228 (CopepodSL-like1a 157 hits and CopepodSL-like1b 71 hits, respectively), including 169 hits to *Lepeophtheirus salmonis* sequences, 38 to *Caligus rogercresseyi*, 9 to *Caligus clemensi*, and 13 hit sequences of other organisms (for CopepodSL-like1a: 1 parasite Trichobilharzia regent; for CopepodSL-like1b: 7 Sea urchin *Strongylocentrotus purpuratus*; 5 Termite *Coptotermes formosanus*, 1 pine *Pinus taeda*).
Note 3: The yellow-highlighted sequences (150) were common hit of both CopepodSL-like1a and 1b, blue-highlighted ones (8) are CopepodSL-like1a specific, and green-highlighted ones (70) are CopepodSL-like1b specific.

>FK900567|FK900567.1 EST_crog_evm_832754 crog_evm *Caligus rogercresseyi* cDNA clone crog_evm_514_338_rev 5', mRNA sequence. Length = 552. Score = 71.9 bits (36), Expect = 2e-10; Identities = 36/36 (100%); Strand = Plus / Minus

```
Query: 1   ccaagtaaataatacgtgtctctgacaaaaatcaag 36
           ||||||||||||||||||||||||||||||||||||
Sbjct: 541 ccaagtaaataatacgtgtctctgacaaaaatcaag 506
```

>FK900566|FK900566.1 EST_crog_evm_829298 crog_evm *Caligus rogercresseyi* cDNA clone crog_evm_514_338_fwd 5', mRNA sequence. Length = 552. Score = 71.9 bits (36), Expect = 2e-10; Identities = 36/36 (100%); Strand = Plus / Plus

```
Query: 1  ccaagtaaataatacgtgtctctgacaaaaatcaag 36
          ||||||||||||||||||||||||||||||||||||
Sbjct: 12 ccaagtaaataatacgtgtctctgacaaaaatcaag 47
```

>FK897007|FK897007.1 EST_crog_evm_809806 crog_evm *Caligus rogercresseyi* cDNA clone crog_evm_509_334_fwd 5', mRNA sequence. Length = 851. Score = 71.9 bits (36), Expect = 2e-10; Identities = 36/36 (100%); Strand = Plus / Plus

```
Query: 1  ccaagtaaataatacgtgtctctgacaaaaatcaag 36
          ||||||||||||||||||||||||||||||||||||
Sbjct: 12 ccaagtaaataatacgtgtctctgacaaaaatcaag 47
```

>FK891778|FK891778.1 EST_crog_evm_813239 crog_evm *Caligus rogercresseyi* cDNA clone crog_evm_502_215_fwd 5', mRNA sequence. Length = 900. Score = 71.9 bits (36), Expect = 2e-10; Identities = 36/36 (100%); Strand = Plus / Plus

```
Query: 1  ccaagtaaataatacgtgtctctgacaaaaatcaag 36
          ||||||||||||||||||||||||||||||||||||
Sbjct: 11 ccaagtaaataatacgtgtctctgacaaaaatcaag 46
```

>FK891741|FK891741.1 EST_crog_evm_813220 crog_evm *Caligus rogercresseyi* cDNA clone crog_evm_502_196_fwd 5', mRNA sequence. Length = 933. Score = 71.9 bits (36), Expect = 2e-10; Identities = 36/36 (100%); Strand = Plus / Plus

```
Query: 1  ccaagtaaataatacgtgtctctgacaaaaatcaag 36
          ||||||||||||||||||||||||||||||||||||
Sbjct: 13 ccaagtaaataatacgtgtctctgacaaaaatcaag 48
```

>FK891058|FK891058.1 EST_crog_evm_819000 crog_evm *Caligus rogercresseyi* cDNA clone crog_evm_501_216_fwd 5', mRNA sequence. Length = 872. Score = 71.9 bits (36), Expect = 2e-10; Identities = 36/36 (100%); Strand = Plus / Plus

```
Query: 1  ccaagtaaataatacgtgtctctgacaaaaatcaag 36
          ||||||||||||||||||||||||||||||||||||
Sbjct: 11 ccaagtaaataatacgtgtctctgacaaaaatcaag 46
```

>FK890984|FK890984.1 EST_crog_evm_818957 crog_evm *Caligus rogercresseyi* cDNA clone crog_evm_501_173_fwd 5', mRNA sequence. Length = 881. Score = 71.9 bits (36), Expect = 2e-10; Identities = 36/36 (100%); Strand = Plus / Plus

```
Query: 1  ccaagtaaataatacgtgtctctgacaaaaatcaag 36
          ||||||||||||||||||||||||||||||||||||
Sbjct: 13 ccaagtaaataatacgtgtctctgacaaaaatcaag 48
```

>FK889015|FK889015.1 EST_crog_evm_856528 crog_evm *Caligus rogercresseyi* cDNA clone crog_evm_007_112_fwd 5', mRNA sequence. Length = 746. Score = 71.9 bits (36), Expect = 2e-10; Identities = 36/36 (100%); Strand = Plus / Plus

```
Query: 1  ccaagtaaataatacgtgtctctgacaaaaatcaag 36
          ||||||||||||||||||||||||||||||||||||
Sbjct: 19 ccaagtaaataatacgtgtctctgacaaaaatcaag 54
```

>FK888297|FK888297.1 EST_crog_evm_854627 crog_evm *Caligus rogercresseyi* cDNA clone crog_evm_005_131_fwd 5', mRNA sequence. Length = 816. Score = 71.9 bits (36), Expect = 2e-10; Identities = 36/36 (100%); Strand = Plus / Plus

```
Query: 1  ccaagtaaataatacgtgtctctgacaaaaatcaag 36
          ||||||||||||||||||||||||||||||||||||
Sbjct: 11 ccaagtaaataatacgtgtctctgacaaaaatcaag 46
```

```
>FK887609|FK887609.1 EST_crog_evm_852660 crog_evm Caligus rogercresseyi cDNA clone
crog_evm_003_084_fwd 5’, mRNA sequence. Length = 666. Score = 71.9 bits (36), Expect = 2e-10;
Identities = 36/36 (100%); Strand = Plus / Plus

Query: 1  ccaagtaaataatacgtgtctctgacaaaaatcaag 36
          ||||||||||||||||||||||||||||||||||||
Sbjct: 7  ccaagtaaataatacgtgtctctgacaaaaatcaag 42


>FK884728|FK884728.1 EST_crog_evp_905672 crog_evp Caligus rogercresseyi cDNA clone
crog_evp_523_104_fwd 5’, mRNA sequence. Length = 907. Score = 71.9 bits (36), Expect = 2e-10;
Identities = 36/36 (100%); Strand = Plus / Plus

Query: 1  ccaagtaaataatacgtgtctctgacaaaaatcaag 36
          ||||||||||||||||||||||||||||||||||||
Sbjct: 12 ccaagtaaataatacgtgtctctgacaaaaatcaag 47


>FK882913|FK882913.1 EST_crog_evp_906240 crog_evp Caligus rogercresseyi cDNA clone
crog_evp_520_288_fwd 5’, mRNA sequence. Length = 904. Score = 71.9 bits (36), Expect = 2e-10;
Identities = 36/36 (100%); Strand = Plus / Plus

Query: 1  ccaagtaaataatacgtgtctctgacaaaaatcaag 36
          ||||||||||||||||||||||||||||||||||||
Sbjct: 20 ccaagtaaataatacgtgtctctgacaaaaatcaag 55


>FK880642|FK880642.1 EST_crog_evp_902742 crog_evp Caligus rogercresseyi cDNA clone
crog_evp_517_246_rev 5’, mRNA Sequence. Length = 597. Score = 71.9 bits (36), Expect = 2e-10;
Identities = 36/36 (100%); Strand = Plus / Minus

Query: 1   ccaagtaaataatacgtgtctctgacaaaaatcaag 36
            ||||||||||||||||||||||||||||||||||||
Sbjct: 586 ccaagtaaataatacgtgtctctgacaaaaatcaag 551


>FK880641|FK880641.1 EST_crog_evp_911190 crog_evp Caligus rogercresseyi cDNA clone
crog_evp_517_246_fwd 5’, mRNA sequence. Length = 598. Score = 71.9 bits (36), Expect = 2e-10;
Identities = 36/36 (100%); Strand = Plus / Plus

Query: 1  ccaagtaaataatacgtgtctctgacaaaaatcaag 36
          ||||||||||||||||||||||||||||||||||||
Sbjct: 13 ccaagtaaataatacgtgtctctgacaaaaatcaag 48
```

>FK874942|FK874942.1 EST_crog_evp_898958 crog_evp *Caligus rogercresseyi* cDNA clone crog_evp_509_302_rev 5', mRNA Sequence. Length = 638. Score = 71.9 bits (36), Expect = 2e-10; Identities = 36/36 (100%); Strand = Plus / Minus

```
Query: 1   ccaagtaaataatacgtgtctctgacaaaaatcaag 36
           ||||||||||||||||||||||||||||||||||||
Sbjct: 620 ccaagtaaataatacgtgtctctgacaaaaatcaag 585
```

>FK874941|FK874941.1 EST_crog_evp_899726 crog_evp *Caligus rogercresseyi* cDNA clone crog_evp_509_302_fwd 5', mRNA sequence. Length = 638. Score = 71.9 bits (36), Expect = 2e-10; Identities = 36/36 (100%); Strand = Plus / Plus

```
Query: 1  ccaagtaaataatacgtgtctctgacaaaaatcaag 36
          ||||||||||||||||||||||||||||||||||||
Sbjct: 19 ccaagtaaataatacgtgtctctgacaaaaatcaag 54
```

>FK874895|FK874895.1 EST_crog_evp_899701 crog_evp *Caligus rogercresseyi* cDNA clone crog_evp_509_277_fwd 5', mRNA sequence. Length = 888. Score = 71.9 bits (36), Expect = 2e-10; Identities = 36/36 (100%); Strand = Plus / Plus

```
Query: 1  ccaagtaaataatacgtgtctctgacaaaaatcaag 36
          ||||||||||||||||||||||||||||||||||||
Sbjct: 20 ccaagtaaataatacgtgtctctgacaaaaatcaag 55
```

>FK872654|FK872654.1 EST_crog_evp_904646 crog_evp *Caligus rogercresseyi* cDNA clone crog_evp_506_230_rev 5', mRNA Sequence. Length = 767. Score = 71.9 bits (36), Expect = 2e-10; Identities = 36/36 (100%); Strand = Plus / Minus

```
Query: 1   ccaagtaaataatacgtgtctctgacaaaaatcaag 36
           ||||||||||||||||||||||||||||||||||||
Sbjct: 765 ccaagtaaataatacgtgtctctgacaaaaatcaag 730
```

>FK872653|FK872653.1 EST_crog_evp_897734 crog_evp *Caligus rogercresseyi* cDNA clone crog_evp_506_230_fwd 5', mRNA sequence. Length = 776. Score = 71.9 bits (36), Expect = 2e-10; Identities = 36/36 (100%); Strand = Plus / Plus

```
Query: 1  ccaagtaaataatacgtgtctctgacaaaaatcaag 36
          ||||||||||||||||||||||||||||||||||||
Sbjct: 12 ccaagtaaataatacgtgtctctgacaaaaatcaag 47
```

>FK870975|FK870975.1 EST_crog_evp_909514 crog_evp *Caligus rogercresseyi* cDNA clone crog_evp_504_106_fwd 5', mRNA sequence. Length = 954. Score = 71.9 bits (36), Expect = 2e-10; Identities = 36/36 (100%); Strand = Plus / Plus

```
Query: 1  ccaagtaaataatacgtgtctctgacaaaaatcaag 36
          ||||||||||||||||||||||||||||||||||||
Sbjct: 19 ccaagtaaataatacgtgtctctgacaaaaatcaag 54
```

>BT077311|BT077311.1 *Caligus rogercresseyi* clone crog-evm-502-215 Exosome complex exonuclease RRP43 putative mRNA, complete cds. Length = 900. Score = 71.9 bits (36), Expect = 2e-10; Identities = 36/36 (100%); Strand = Plus / Plus

```
Query: 1  ccaagtaaataatacgtgtctctgacaaaaatcaag 36
          ||||||||||||||||||||||||||||||||||||
Sbjct: 11 ccaagtaaataatacgtgtctctgacaaaaatcaag 46
```

>BT077080|BT077080.1 *Caligus rogercresseyi* clone crog-evp-509-277 Probable O-sialoglycoprotein endopeptidase putative mRNA, complete cds. Length = 1131. Score = 71.9 bits (36), Expect = 2e-10; Identities = 36/36 (100%); Strand = Plus / Plus

```
Query: 1  ccaagtaaataatacgtgtctctgacaaaaatcaag 36
          ||||||||||||||||||||||||||||||||||||
Sbjct: 20 ccaagtaaataatacgtgtctctgacaaaaatcaag 55
```

>BT076119|BT076119.1 *Caligus rogercresseyi* clone crog-evp-504-106 Small ubiquitin-related modifier 3 precursor putative mRNA, complete cds. Length = 956. Score = 71.9 bits (36), Expect = 2e-10; Identities = 36/36 (100%); Strand = Plus / Plus

```
Query: 1  ccaagtaaataatacgtgtctctgacaaaaatcaag 36
          ||||||||||||||||||||||||||||||||||||
Sbjct: 19 ccaagtaaataatacgtgtctctgacaaaaatcaag 54
```

>BT075921|BT075921.1 *Caligus rogercresseyi* clone crog-evm-502-196 Dehydrogenase/reductase SDR family member 1 putative mRNA, complete cds. Length = 1195. Score = 71.9 bits (36), Expect = 2e-10; Identities = 36/36 (100%); Strand = Plus / Plus

```
Query: 1  ccaagtaaataatacgtgtctctgacaaaaatcaag 36
          ||||||||||||||||||||||||||||||||||||
Sbjct: 13 ccaagtaaataatacgtgtctctgacaaaaatcaag 48
```

>FK898459|FK898459.1 EST_crog_evm_816441 crog_evm *Caligus rogercresseyi* cDNA clone crog_evm_511_345_fwd 5', mRNA sequence. Length = 904. Score = 63.9 bits (32), Expect = 5e-08; Identities = 32/32 (100%); Strand = Plus / Plus

```
Query: 5  gtaaataatacgtgtctctgacaaaaatcaag 36
          ||||||||||||||||||||||||||||||||
Sbjct: 5  gtaaataatacgtgtctctgacaaaaatcaag 36
```

>FK893205|FK893205.1 EST_crog_evm_807668 crog_evm *Caligus rogercresseyi* cDNA clone crog_evm_504_212_fwd 5', mRNA sequence. Length = 896. Score = 63.9 bits (32), Expect = 5e-08; Identities = 32/32 (100%); Strand = Plus / Plus

```
Query: 5  gtaaataatacgtgtctctgacaaaaatcaag 36
          ||||||||||||||||||||||||||||||||
Sbjct: 5  gtaaataatacgtgtctctgacaaaaatcaag 36
```

>FK885407|FK885407.1 EST_crog_evp_916014 crog_evp *Caligus rogercresseyi* cDNA clone crog_evp_524_078_fwd 5', mRNA sequence. Length = 906. Score = 63.9 bits (32), Expect = 5e-08; Identities = 35/36 (97%); Strand = Plus / Plus

```
Query: 1  ccaagtaaataatacgtgtctctgacaaaaatcaag 36
          |||||||||||||||||||||||||||| |||||||
Sbjct: 19 ccaagtaaataatacgtgtctctgacaataatcaag 54
```

>BT076438|BT076438.1 *Caligus rogercresseyi* clone crog-evm-511-345 rRNA-processing protein FCF1 homolog putative mRNA, complete cds. Length = 966. Score = 63.9 bits (32), Expect = 5e-08; Identities = 32/32 (100%); Strand = Plus / Plus

```
Query: 5  gtaaataatacgtgtctctgacaaaaatcaag 36
          ||||||||||||||||||||||||||||||||
Sbjct: 5  gtaaataatacgtgtctctgacaaaaatcaag 36
```

>BT076141|BT076141.1 *Caligus rogercresseyi* clone crog-evm-504-212 Geranylgeranyl pyrophosphate synthetase putative mRNA, complete cds. Length = 1065. Score = 63.9 bits (32), Expect = 5e-08; Identities = 32/32 (100%); Strand = Plus / Plus

```
Query: 5  gtaaataatacgtgtctctgacaaaaatcaag 36
          ||||||||||||||||||||||||||||||||
Sbjct: 5  gtaaataatacgtgtctctgacaaaaatcaag 36
```

>FK896085|FK896085.1 EST_crog_evm_822935 crog_evm *Caligus rogercresseyi* cDNA clone crog_evm_508_215_fwd 5', mRNA sequence. Length = 862. Score = 60.0 bits (30), Expect = 8e-07; Identities = 30/30 (100%); Strand = Plus / Plus

```
Query: 1  ccaagtaaataatacgtgtctctgacaaaa 30
          ||||||||||||||||||||||||||||||
Sbjct: 7  ccaagtaaataatacgtgtctctgacaaaa 36
```

>BT076974|BT076974.1 *Caligus rogercresseyi* clone crog-evm-508-215 Selenocysteine lyase putative mRNA, complete cds. Length = 862. Score = 60.0 bits (30), Expect = 8e-07; Identities = 30/30 (100%); Strand = Plus / Plus

```
Query: 1  ccaagtaaataatacgtgtctctgacaaaa 30
          ||||||||||||||||||||||||||||||
Sbjct: 7  ccaagtaaataatacgtgtctctgacaaaa 36
```

>GO403137|GO403137.1 EST_ccle_evs_1033843 ccleevs mixed_tissue *Caligus clemensi* cDNA *Caligus clemensi* cDNA clone ccle_evs_510_019 5', mRNA sequence. Length = 736. Score = 58.0 bits (29), Expect = 3e-06; Identities = 29/29 (100%); Strand = Plus / Plus

```
Query: 1  ccaagtaaataatacgtgtctctgacaaa 29
          |||||||||||||||||||||||||||||
Sbjct: 10 ccaagtaaataatacgtgtctctgacaaa 38
```

>GO400088|GO400088.1 EST_ccle_evs_1045706 ccleevs mixed_tissue *Caligus clemensi* cDNA *Caligus clemensi* cDNA clone ccle_evs_505_362 3', mRNA sequence. Length = 802. Score = 58.0 bits (29), Expect = 3e-06; Identities = 29/29 (100%); Strand = Plus / Minus

```
Query: 1   ccaagtaaataatacgtgtctctgacaaa 29
           |||||||||||||||||||||||||||||
Sbjct: 789 ccaagtaaataatacgtgtctctgacaaa 761
```

>GO400087|GO400087.1 EST_ccle_evs_1038794 ccleevs mixed_tissue *Caligus clemensi* cDNA *Caligus clemensi* cDNA clone ccle_evs_505_362 5', mRNA sequence. Length = 808. Score = 58.0 bits (29), Expect = 3e-06; Identities = 29/29 (100%); Strand = Plus / Plus

```
Query: 1  ccaagtaaataatacgtgtctctgacaaa 29
          |||||||||||||||||||||||||||||
Sbjct: 20 ccaagtaaataatacgtgtctctgacaaa 48
```

>BT080960|BT080960.1 *Caligus clemensi* clone ccle-evs-505-362 N-acetyltransferase NAT13 putative mRNA, complete cds. Length = 808. Score = 58.0 bits (29), Expect = 3e-06; Identities = 29/29 (100%); Strand = Plus / Plus

```
Query: 1  ccaagtaaataatacgtgtctctgacaaa 29
          |||||||||||||||||||||||||||||
Sbjct: 20 ccaagtaaataatacgtgtctctgacaaa 48
```

>GO404472|GO404472.1 EST_ccle_evs_1041452 ccleevs mixed_tissue *Caligus clemensi* cDNA *Caligus clemensi* cDNA clone ccle_evs_511_332 5', mRNA sequence. Length = 885. Score = 56.0 bits (28), Expect = 1e-05; Identities = 28/28 (100%); Strand = Plus / Plus

```
Query: 1  ccaagtaaataatacgtgtctctgacaa 28
          ||||||||||||||||||||||||||||
Sbjct: 16 ccaagtaaataatacgtgtctctgacaa 43
```

>GW663516|GW663516.1 EST_lsaA_evv_1239427 lsaAevv mixed_tissue *Lepeophtheirus salmonis* (Atlantic Norway) cDNA *Lepeophtheirus salmonis* cDNA clone lsaA_evv_524_097 3', mRNA sequence. Length = 699. Score = 52.0 bits (26), Expect = 2e-04; Identities = 26/26 (100%); Strand = Plus / Minus

```
Query: 1   ccaagtaaataatacgtgtctctgac 26
           ||||||||||||||||||||||||||
Sbjct: 690 ccaagtaaataatacgtgtctctgac 665
```

>GW663515|GW663515.1 EST_lsaA_evv_1230979 lsaAevv mixed_tissue *Lepeophtheirus salmonis* (Atlantic Norway) cDNA *Lepeophtheirus salmonis* cDNA clone lsaA_evv_524_097 5', mRNA sequence. Length = 699. Score = 52.0 bits (26), Expect = 2e-04; Identities = 26/26 (100%); Strand = Plus / Plus

```
Query: 1  ccaagtaaataatacgtgtctctgac 26
          ||||||||||||||||||||||||||
Sbjct: 10 ccaagtaaataatacgtgtctctgac 35
```

>GW654699|GW654699.1 EST_lsaA_evv_1237204 lsaAevv mixed_tissue *Lepeophtheirus salmonis* (Atlantic Norway) cDNA *Lepeophtheirus salmonis* cDNA clone lsaA_evv_512_178 5', mRNA sequence. Length = 815. Score = 52.0 bits (26), Expect = 2e-04; Identities = 26/26 (100%); Strand = Plus / Plus

```
Query: 1  ccaagtaaataatacgtgtctctgac 26
          ||||||||||||||||||||||||||
Sbjct: 12 ccaagtaaataatacgtgtctctgac 37
```

>GW632461|GW632461.1 EST_lsal_evj_1206504 lsalevj whole *Lepeophtheirus salmonis* (Pacific) cDNA *Lepeophtheirus salmonis* cDNA clone lsal_evj_028_198 5', mRNA sequence. Length = 842. Score = 52.0 bits (26), Expect = 2e-04; Identities = 26/26 (100%); Strand = Plus / Plus

```
Query: 1  ccaagtaaataatacgtgtctctgac 26
          ||||||||||||||||||||||||||
Sbjct: 8  ccaagtaaataatacgtgtctctgac 33
```

>EX484407|EX484407.1 EST_lsal_evj_795148 lsalevj mixed_tissue_mixed_stage *Lepeophtheirus salmonis* (Pacific) cDNA *Lepeophtheirus salmonis* cDNA clone lsal_evj_513_172_rev 3', mRNA sequence. Length = 745. Score = 52.0 bits (26), Expect = 2e-04; Identities = 26/26 (100%); Strand = Plus / Minus

```
Query: 1   ccaagtaaataatacgtgtctctgac 26
           ||||||||||||||||||||||||||
Sbjct: 734 ccaagtaaataatacgtgtctctgac 709
```

>EX484406|EX484406.1 EST_lsal_evj_794764 lsalevj mixed_tissue_mixed_stage *Lepeophtheirus salmonis* (Pacific) cDNA *Lepeophtheirus salmonis* cDNA clone lsal_evj_513_172_fwd 5', mRNA sequence. Length = 745. Score = 52.0 bits (26), Expect = 2e-04; Identities = 26/26 (100%); Strand = Plus / Plus

```
Query: 1  ccaagtaaataatacgtgtctctgac 26
          ||||||||||||||||||||||||||
Sbjct: 12 ccaagtaaataatacgtgtctctgac 37
```

>JP341769|JP341769.1 TSA: *Lepeophtheirus salmonis* lsal-atl-6219376 mRNA sequence. Length = 515. Score = 52.0 bits (26), Expect = 2e-04; Identities = 26/26 (100%); Strand = Plus / Plus

```
Query: 1  ccaagtaaataatacgtgtctctgac 26
          ||||||||||||||||||||||||||
Sbjct: 3  ccaagtaaataatacgtgtctctgac 28
```

>JP326866|JP326866.1 TSA: *Lepeophtheirus salmonis* lsal-pac-6256820 mRNA sequence. Length = 829. Score = 52.0 bits (26), Expect = 2e-04; Identities = 26/26 (100%); Strand = Plus / Plus

```
Query: 1  ccaagtaaataatacgtgtctctgac 26
          ||||||||||||||||||||||||||
Sbjct: 3  ccaagtaaataatacgtgtctctgac 28
```

>JP325104|JP325104.1 TSA: *Lepeophtheirus salmonis* lsal-pac-6255058 mRNA sequence. Length = 677. Score = 52.0 bits (26), Expect = 2e-04; Identities = 26/26 (100%); Strand = Plus / Plus

```
Query: 1  ccaagtaaataatacgtgtctctgac 26
          ||||||||||||||||||||||||||
Sbjct: 3  ccaagtaaataatacgtgtctctgac 28
```

>JP324653|JP324653.1 TSA: *Lepeophtheirus salmonis* lsal-pac-6254607 mRNA sequence. Length = 650. Score = 52.0 bits (26), Expect = 2e-04; Identities = 26/26 (100%); Strand = Plus / Minus

```
Query: 1   ccaagtaaataatacgtgtctctgac 26
           ||||||||||||||||||||||||||
Sbjct: 648 ccaagtaaataatacgtgtctctgac 623
```

>JP317678|JP317678.1 TSA: *Lepeophtheirus salmonis* lsal-pac-6247632 mRNA sequence. Length = 449. Score = 52.0 bits (26), Expect = 2e-04; Identities = 26/26 (100%); Strand = Plus / Minus

```
Query: 1   ccaagtaaataatacgtgtctctgac 26
           ||||||||||||||||||||||||||
```

Sbjct: 447 ccaagtaaataatacgtgtctctgac 422

>JP316075|JP316075.1 TSA: *Lepeophtheirus salmonis* lsal-pac-6246028 mRNA sequence. Length = 426. Score = 52.0 bits (26), Expect = 2e-04; Identities = 26/26 (100%); Strand = Plus / Plus

```
Query: 1  ccaagtaaataatacgtgtctctgac 26
          ||||||||||||||||||||||||||
Sbjct: 3  ccaagtaaataatacgtgtctctgac 28
```

>JP309928|JP309928.1 TSA: *Lepeophtheirus salmonis* lsal-pac-6239873 mRNA sequence. Length = 1718. Score = 52.0 bits (26), Expect = 2e-04; Identities = 26/26 (100%); Strand = Plus / Plus

```
Query: 1  ccaagtaaataatacgtgtctctgac 26
          ||||||||||||||||||||||||||
Sbjct: 1  ccaagtaaataatacgtgtctctgac 26
```

>JP309589|JP309589.1 TSA: *Lepeophtheirus salmonis* lsal-pac-6239534 mRNA sequence. Length = 1136. Score = 52.0 bits (26), Expect = 2e-04; Identities = 26/26 (100%); Strand = Plus / Plus

```
Query: 1  ccaagtaaataatacgtgtctctgac 26
          ||||||||||||||||||||||||||
Sbjct: 3  ccaagtaaataatacgtgtctctgac 28
```

>JP309129|JP309129.1 TSA: *Lepeophtheirus salmonis* lsal-pac-6239074 mRNA sequence. Length = 906. Score = 52.0 bits (26), Expect = 2e-04; Identities = 26/26 (100%); Strand = Plus / Plus

```
Query: 1  ccaagtaaataatacgtgtctctgac 26
          ||||||||||||||||||||||||||
Sbjct: 8  ccaagtaaataatacgtgtctctgac 33
```

>JP306950|JP306950.1 TSA: *Lepeophtheirus salmonis* lsal-pac-6236892 mRNA sequence. Length = 1115. Score = 52.0 bits (26), Expect = 2e-04; Identities = 26/26 (100%); Strand = Plus / Plus

```
Query: 1  ccaagtaaataatacgtgtctctgac 26
          ||||||||||||||||||||||||||
Sbjct: 3  ccaagtaaataatacgtgtctctgac 28
```

>JP305792|JP305792.1 TSA: *Lepeophtheirus salmonis* lsal-pac-6235733 mRNA sequence. Length = 745. Score = 52.0 bits (26), Expect = 2e-04; Identities = 26/26 (100%); Strand = Plus / Plus

```
Query: 1  ccaagtaaataatacgtgtctctgac 26
          ||||||||||||||||||||||||||
Sbjct: 12 ccaagtaaataatacgtgtctctgac 37
```

>JP305248|JP305248.1 TSA: *Lepeophtheirus salmonis* lsal-pac-6235189 mRNA sequence. Length = 551. Score = 52.0 bits (26), Expect = 2e-04; Identities = 26/26 (100%); Strand = Plus / Plus

```
Query: 1  ccaagtaaataatacgtgtctctgac 26
          ||||||||||||||||||||||||||
Sbjct: 2  ccaagtaaataatacgtgtctctgac 27
```

>BT121551|BT121551.1 *Lepeophtheirus salmonis* Atlantic form clone lsaA-evv-524-097 rRNA-processing protein FCF1 homolog putative mRNA, complete cds. Length = 699. Score = 52.0 bits (26), Expect = 2e-04; Identities = 26/26 (100%); Strand = Plus / Plus

```
Query: 1  ccaagtaaataatacgtgtctctgac 26
          ||||||||||||||||||||||||||
Sbjct: 10 ccaagtaaataatacgtgtctctgac 35
```

>BT121323|BT121323.1 *Lepeophtheirus salmonis* Atlantic form clone lsaA-evv-512-178 Alkylated DNA repair protein alkB homolog 6 putative mRNA, complete cds. Length = 1031. Score = 52.0 bits (26), Expect = 2e-04; Identities = 26/26 (100%); Strand = Plus / Plus

```
Query: 1  ccaagtaaataatacgtgtctctgac 26
          ||||||||||||||||||||||||||
Sbjct: 12 ccaagtaaataatacgtgtctctgac 37
```

>BT120775|BT120775.1 *Lepeophtheirus salmonis* clone lsal-evj-028-198 Probable U3 small nucleolar RNA-associated protein 11 putative mRNA, complete cds. Length = 881. Score = 52.0 bits (26), Expect = 2e-04; Identities = 26/26 (100%); Strand = Plus / Plus

```
Query: 1  ccaagtaaataatacgtgtctctgac 26
          ||||||||||||||||||||||||||
Sbjct: 8  ccaagtaaataatacgtgtctctgac 33
```

>BT077439|BT077439.1 *Lepeophtheirus salmonis* Pacific form clone lsal-evj-513-172 39S ribosomal protein L41, mitochondrial precursor putative mRNA, complete cds. Length = 745. Score = 52.0 bits (26), Expect = 2e-04; Identities = 26/26 (100%); Strand = Plus / Plus

```
Query: 1  ccaagtaaataatacgtgtctctgac 26
          ||||||||||||||||||||||||||
Sbjct: 12 ccaagtaaataatacgtgtctctgac 37
```

>JP312499|JP312499.1 TSA: *Lepeophtheirus salmonis* lsal-pac-6242449 mRNA sequence. Length = 1597. Score = 50.1 bits (25), Expect = 8e-04; Identities = 25/25 (100%); Strand = Plus / Minus

```
Query: 2    caagtaaataatacgtgtctctgac 26
            |||||||||||||||||||||||||
Sbjct: 1597 caagtaaataatacgtgtctctgac 1573
```

>JP310143|JP310143.1 TSA: *Lepeophtheirus salmonis* lsal-pac-6240088 mRNA sequence. Length = 924. Score = 50.1 bits (25), Expect = 8e-04; Identities = 25/25 (100%); Strand = Plus / Plus

```
Query: 2  caagtaaataatacgtgtctctgac 26
          |||||||||||||||||||||||||
Sbjct: 1  caagtaaataatacgtgtctctgac 25
```

>HO699365|HO699365.1 NLG2733-2007-09-17.ab1 *Lepeophtheirus salmonis* NLG *Lepeophtheirus salmonis* cDNA, mRNA sequence. Length = 809. Score = 46.1 bits (23), Expect = 0.012; Identities = 23/23 (100%); Strand = Plus / Plus

```
Query: 4  agtaaataatacgtgtctctgac 26
          |||||||||||||||||||||||
Sbjct: 1  agtaaataatacgtgtctctgac 23
```

>GW648607|GW648607.1 EST_lsaA_evv_1239819 lsaAevv mixed_tissue *Lepeophtheirus salmonis* (Atlantic Norway) cDNA *Lepeophtheirus salmonis* cDNA clone lsaA_evv_504_105 3', mRNA sequence. Length = 411. Score = 44.1 bits (22), Expect = 0.048; Identities = 22/22 (100%); Strand = Plus / Minus

```
Query: 5   gtaaataatacgtgtctctgac 26
           ||||||||||||||||||||||
Sbjct: 407 gtaaataatacgtgtctctgac 386
```

>GW648606|GW648606.1 EST_lsaA_evv_1225995 lsaAevv mixed_tissue *Lepeophtheirus salmonis* (Atlantic Norway) cDNA *Lepeophtheirus salmonis* cDNA clone lsaA_evv_504_105 5', mRNA sequence. Length = 411. Score = 44.1 bits (22), Expect = 0.048; Identities = 22/22 (100%); Strand = Plus / Plus

```
Query: 5  gtaaataatacgtgtctctgac 26
          ||||||||||||||||||||||
Sbjct: 5  gtaaataatacgtgtctctgac 26
```

>GW623776|GW623776.1 EST_lsal_evj_1200638 lsalevj whole *Lepeophtheirus salmonis* (Pacific) cDNA *Lepeophtheirus salmonis* cDNA clone lsal_evj_016_284 5', mRNA sequence. Length = 473. Score = 44.1 bits (22), Expect = 0.048; Identities = 25/26 (96%); Strand = Plus / Plus

```
Query: 1  ccaagtaaataatacgtgtctctgac 26
          ||||||| ||||||||||||||||||
```

Sbjct: 10 ccaagtagataatacgtgtctctgac 35

>FK904154|FK904154.1 EST_lsal_evj_875439 lsalevj mixed_tissue_mixed_stage *Lepeophtheirus salmonis* (Pacific) cDNA *Lepeophtheirus salmonis* cDNA clone lsal_evj_011_207_fwd 5', mRNA sequence. Length = 815. Score = 44.1 bits (22), Expect = 0.048; Identities = 25/26 (96%); Strand = Plus / Plus

```
Query: 1  ccaagtaaataatacgtgtctctgac 26
          |||||| |||||||||||||||||||
Sbjct: 14 ccaagttaataatacgtgtctctgac 39
```

>JP306585|JP306585.1 TSA: *Lepeophtheirus salmonis* lsal-pac-6236527 mRNA sequence. Length = 815. Score = 44.1 bits (22), Expect = 0.048; Identities = 25/26 (96%); Strand = Plus / Plus

```
Query: 1  ccaagtaaataatacgtgtctctgac 26
          |||||| |||||||||||||||||||
Sbjct: 14 ccaagttaataatacgtgtctctgac 39
```

>BT120941|BT120941.1 *Lepeophtheirus salmonis* clone lsal-evj-016-284 N6-adenine-specific DNA methyltransferase 2 putative mRNA, complete cds. Length = 902. Score = 44.1 bits (22), Expect = 0.048; Identities = 25/26 (96%); Strand = Plus / Plus

```
Query: 1  ccaagtaaataatacgtgtctctgac 26
          ||||||| ||||||||||||||||||
Sbjct: 10 ccaagtagataatacgtgtctctgac 35
```

>HO674114|HO674114.1 LF132-2007-01-09.ab1 *Lepeophtheirus salmonis* LF *Lepeophtheirus salmonis* cDNA, mRNA sequence. Length = 748. Score = 40.1 bits (20), Expect = 0.75; Identities = 20/20 (100%); Strand = Plus / Plus

```
Query: 7  aaataatacgtgtctctgac 26
          ||||||||||||||||||||
Sbjct: 65 aaataatacgtgtctctgac 84
```

>HO670227|HO670227.1 FN4312-2003-02-19.ab1 *Lepeophtheirus salmonis* ME *Lepeophtheirus salmonis* cDNA, mRNA sequence. Length = 734. Score = 40.1 bits (20), Expect = 0.75; Identities = 20/20 (100%); Strand = Plus / Plus

```
Query: 7  aaataatacgtgtctctgac 26
          ||||||||||||||||||||
Sbjct: 52 aaataatacgtgtctctgac 71
```

>HO669877|HO669877.1 FN4062-2003-02-18.ab1 *Lepeophtheirus salmonis* ME *Lepeophtheirus salmonis* cDNA, mRNA sequence. Length = 763. Score = 40.1 bits (20), Expect = 0.75; Identities = 20/20 (100%); Strand = Plus / Plus

```
Query: 7  aaataatacgtgtctctgac 26
          ||||||||||||||||||||
Sbjct: 65 aaataatacgtgtctctgac 84
```

>HO669284|HO669284.1 FN3546-2002-03-25.ab1 *Lepeophtheirus salmonis* ME *Lepeophtheirus salmonis* cDNA, mRNA sequence. Length = 798. Score = 40.1 bits (20), Expect = 0.75; Identities = 20/20 (100%); Strand = Plus / Plus

```
Query: 7  aaataatacgtgtctctgac 26
          ||||||||||||||||||||
Sbjct: 62 aaataatacgtgtctctgac 81
```

>HO669064|HO669064.1 FN3088-2002-02-18.ab1 *Lepeophtheirus salmonis* ME *Lepeophtheirus salmonis* cDNA, mRNA sequence. Length = 721. Score = 40.1 bits (20), Expect = 0.75; Identities = 20/20 (100%); Strand = Plus / Plus

```
Query: 7  aaataatacgtgtctctgac 26
          ||||||||||||||||||||
Sbjct: 47 aaataatacgtgtctctgac 66
```

>HO697196|HO697196.1 LPU1746-2007-09-05.ab1 *Lepeophtheirus salmonis* LPU *Lepeophtheirus salmonis* cDNA, mRNA sequence. Length = 956. Score = 38.2 bits (19), Expect = 3.0; Identities = 19/19 (100%); Strand = Plus / Plus

```
Query: 8   aataatacgtgtctctgac 26
           |||||||||||||||||||
Sbjct: 87  aataatacgtgtctctgac 105
```

>HO697082|HO697082.1 LPU1523-2007-09-04.ab1 *Lepeophtheirus salmonis* LPU *Lepeophtheirus salmonis* cDNA, mRNA sequence. Length = 817. Score = 38.2 bits (19), Expect = 3.0; Identities = 19/19 (100%); Strand = Plus / Plus

```
Query: 8   aataatacgtgtctctgac 26
           |||||||||||||||||||
Sbjct: 86  aataatacgtgtctctgac 104
```

>HO670896|HO670896.1 FN5196-2005-01-17.ab1 *Lepeophtheirus salmonis* ME *Lepeophtheirus salmonis* cDNA, mRNA sequence. Length = 705. Score = 38.2 bits (19), Expect = 3.0; Identities = 19/19 (100%); Strand = Plus / Plus

```
Query: 8  aataatacgtgtctctgac 26
          |||||||||||||||||||
Sbjct: 22 aataatacgtgtctctgac 40
```

>HO669489|HO669489.1 HAV-FN-FN2856-2001-12-19.ab1 *Lepeophtheirus salmonis* ME *Lepeophtheirus salmonis* cDNA, mRNA sequence. Length = 684. Score = 38.2 bits (19), Expect = 3.0; Identities = 19/19 (100%); Strand = Plus / Plus

```
Query: 8  aataatacgtgtctctgac 26
          |||||||||||||||||||
Sbjct: 48 aataatacgtgtctctgac 66
```

>HO668711|HO668711.1 HAV-FN-FN2625-2001-12-13.ab1 *Lepeophtheirus salmonis* ME *Lepeophtheirus salmonis* cDNA, mRNA sequence. Length = 626. Score = 38.2 bits (19), Expect = 3.0; Identities = 19/19 (100%); Strand = Plus / Plus

```
Query: 8  aataatacgtgtctctgac 26
          |||||||||||||||||||
Sbjct: 20 aataatacgtgtctctgac 38
```

>HO668468|HO668468.1 HAV-FN-FN2389-2001-12-10.ab1 *Lepeophtheirus salmonis* ME *Lepeophtheirus salmonis* cDNA, mRNA sequence. Length = 738. Score = 38.2 bits (19), Expect = 3.0; Identities = 19/19 (100%); Strand = Plus / Plus

```
Query: 8  aataatacgtgtctctgac 26
          |||||||||||||||||||
Sbjct: 45 aataatacgtgtctctgac 63
```

>GW643157|GW643157.1 EST_lsaA_evu_1219699 lsaAevu mixed_tissue *Lepeophtheirus salmonis* (Atlantic Canada) cDNA *Lepeophtheirus salmonis* cDNA clone lsaA_evu_509_337 5', mRNA sequence. Length = 898. Score = 38.2 bits (19), Expect = 3.0; Identities = 19/19 (100%); Strand = Plus / Plus

```
Query: 8  aataatacgtgtctctgac 26
          |||||||||||||||||||
Sbjct: 17 aataatacgtgtctctgac 35
```

>GW627016|GW627016.1 EST_lsal_evj_1211364 lsalevj whole *Lepeophtheirus salmonis* (Pacific) cDNA *Lepeophtheirus salmonis* cDNA clone lsal_evj_021_066 5', mRNA sequence. Length = 838. Score = 38.2 bits (19), Expect = 3.0; Identities = 19/19 (100%); Strand = Plus / Plus

```
Query: 8  aataatacgtgtctctgac 26
          |||||||||||||||||||
Sbjct: 19 aataatacgtgtctctgac 37
```

>GW626427|GW626427.1 EST_lsal_evj_1208369 lsalevj whole *Lepeophtheirus salmonis* (Pacific) cDNA *Lepeophtheirus salmonis* cDNA clone lsal_evj_020_143 5', mRNA sequence. Length = 874. Score = 38.2 bits (19), Expect = 3.0; Identities = 19/19 (100%); Strand = Plus / Plus

```
Query: 8  aataatacgtgtctctgac 26
          |||||||||||||||||||
Sbjct: 17 aataatacgtgtctctgac 35
```

>EY509580|EY509580.1 EST_lsal_pf_801979 lsalpf whole *Lepeophtheirus salmonis* (Pacific) cDNA *Lepeophtheirus salmonis* cDNA clone lsal_pf_6_379_rev 5', mRNA sequence. Length = 763. Score = 38.2 bits (19), Expect = 3.0; Identities = 19/19 (100%); Strand = Plus / Plus

```
Query: 8  aataatacgtgtctctgac 26
          |||||||||||||||||||
Sbjct: 14 aataatacgtgtctctgac 32
```

>EY508162|EY508162.1 EST_lsal_ch4_771033 lsalch4 *whole Lepeophtheirus salmonis* (Pacific) cDNA *Lepeophtheirus salmonis* cDNA clone lsal_ch4_1_153_fwd 3', mRNA sequence. Length = 868. Score = 38.2 bits (19), Expect = 3.0; Identities = 19/19 (100%); Strand = Plus / Minus

```
Query: 8   aataatacgtgtctctgac 26
           |||||||||||||||||||
Sbjct: 867 aataatacgtgtctctgac 849
```

>JP329312|JP329312.1 TSA: *Lepeophtheirus salmonis* lsal-pac-6259268 mRNA sequence. Length = 1912. Score = 38.2 bits (19), Expect = 3.0; Identities = 19/19 (100%); Strand = Plus / Plus

```
Query: 8  aataatacgtgtctctgac 26
          |||||||||||||||||||
Sbjct: 9  aataatacgtgtctctgac 27
```

>JP329213|JP329213.1 TSA: *Lepeophtheirus salmonis* lsal-pac-6259169 mRNA sequence. Length = 1722. Score = 38.2 bits (19), Expect = 3.0; Identities = 19/19 (100%); Strand = Plus / Minus

```
Query: 8    aataatacgtgtctctgac 26
            |||||||||||||||||||
Sbjct: 1702 aataatacgtgtctctgac 1684
```

>JP329151|JP329151.1 TSA: *Lepeophtheirus salmonis* lsal-pac-6259107 mRNA sequence. Length = 1655. Score = 38.2 bits (19), Expect = 3.0; Identities = 19/19 (100%); Strand = Plus / Plus

```
Query: 8  aataatacgtgtctctgac 26
```

```
          |||||||||||||||||||
Sbjct: 10 aataatacgtgtctctgac 28
```

>JP329001|JP329001.1 TSA: *Lepeophtheirus salmonis* lsal-pac-6258957 mRNA sequence. Length = 1494. Score = 38.2 bits (19), Expect = 3.0; Identities = 19/19 (100%); Strand = Plus / Plus

```
Query: 8  aataatacgtgtctctgac 26
          |||||||||||||||||||
Sbjct: 8  aataatacgtgtctctgac 26
```

>JP328915|JP328915.1 TSA: *Lepeophtheirus salmonis* lsal-pac-6258871 mRNA sequence. Length = 1437. Score = 38.2 bits (19), Expect = 3.0; Identities = 19/19 (100%); Strand = Plus / Plus

```
Query: 8  aataatacgtgtctctgac 26
          |||||||||||||||||||
Sbjct: 8  aataatacgtgtctctgac 26
```

>JP328699|JP328699.1 TSA: *Lepeophtheirus salmonis* lsal-pac-6258654 mRNA sequence. Length = 1285. Score = 38.2 bits (19), Expect = 3.0; Identities = 19/19 (100%); Strand = Plus / Plus

```
Query: 8  aataatacgtgtctctgac 26
          |||||||||||||||||||
Sbjct: 9  aataatacgtgtctctgac 27
```

>JP328516|JP328516.1 TSA: *Lepeophtheirus salmonis* lsal-pac-6258471 mRNA sequence. Length = 1203. Score = 38.2 bits (19), Expect = 3.0; Identities = 19/19 (100%); Strand = Plus / Minus

```
Query: 8    aataatacgtgtctctgac 26
            |||||||||||||||||||
Sbjct: 1196 aataatacgtgtctctgac 1178
```

>JP328267|JP328267.1 TSA: *Lepeophtheirus salmonis* lsal-pac-6258221 mRNA sequence. Length = 1101. Score = 38.2 bits (19), Expect = 3.0; Identities = 19/19 (100%); Strand = Plus / Plus

```
Query: 8  aataatacgtgtctctgac 26
          |||||||||||||||||||
Sbjct: 7  aataatacgtgtctctgac 25
```

>JP328237|JP328237.1 TSA: *Lepeophtheirus salmonis* lsal-pac-6258191 mRNA sequence. Length = 1095. Score = 38.2 bits (19), Expect = 3.0; Identities = 19/19 (100%); Strand = Plus / Plus

```
Query: 8  aataatacgtgtctctgac 26
          |||||||||||||||||||
```

```
Sbjct: 7  aataatacgtgtctctgac 25
```

>JP327990|JP327990.1 TSA: *Lepeophtheirus salmonis* lsal-pac-6257944 mRNA sequence. Length = 1028. Score = 38.2 bits (19), Expect = 3.0; Identities = 19/19 (100%); Strand = Plus / Minus

```
Query: 8    aataatacgtgtctctgac 26
            |||||||||||||||||||
Sbjct: 1021 aataatacgtgtctctgac 1003
```

>JP327515|JP327515.1 TSA: *Lepeophtheirus salmonis* lsal-pac-6257469 mRNA sequence. Length = 929. Score = 38.2 bits (19), Expect = 3.0; Identities = 19/19 (100%); Strand = Plus / Minus

```
Query: 8   aataatacgtgtctctgac 26
           |||||||||||||||||||
Sbjct: 922 aataatacgtgtctctgac 904
```

>JP327467|JP327467.1 TSA: *Lepeophtheirus salmonis* lsal-pac-6257421 mRNA sequence. Length = 921. Score = 38.2 bits (19), Expect = 3.0; Identities = 19/19 (100%); Strand = Plus / Minus

```
Query: 8   aataatacgtgtctctgac 26
           |||||||||||||||||||
Sbjct: 914 aataatacgtgtctctgac 896
```

>JP327353|JP327353.1 TSA: *Lepeophtheirus salmonis* lsal-pac-6257307 mRNA sequence. Length = 901. Score = 38.2 bits (19), Expect = 3.0; Identities = 19/19 (100%); Strand = Plus / Plus

```
Query: 8  aataatacgtgtctctgac 26
           |||||||||||||||||||
Sbjct: 5  aataatacgtgtctctgac 23
```

>JP327043|JP327043.1 TSA: *Lepeophtheirus salmonis* lsal-pac-6256997 mRNA sequence. Length = 853. Score = 38.2 bits (19), Expect = 3.0; Identities = 19/19 (100%); Strand = Plus / Minus

```
Query: 8   aataatacgtgtctctgac 26
           |||||||||||||||||||
Sbjct: 845 aataatacgtgtctctgac 827
```

>JP326842|JP326842.1 TSA: *Lepeophtheirus salmonis* lsal-pac-6256796 mRNA sequence. Length = 826. Score = 38.2 bits (19), Expect = 3.0; Identities = 19/19 (100%); Strand = Plus / Minus

```
Query: 8   aataatacgtgtctctgac 26
           |||||||||||||||||||
Sbjct: 818 aataatacgtgtctctgac 800
```

>JP326619|JP326619.1 TSA: *Lepeophtheirus salmonis* lsal-pac-6256573 mRNA sequence. Length = 800. Score = 38.2 bits (19), Expect = 3.0; Identities = 19/19 (100%); Strand = Plus / Minus

```
Query: 8   aataatacgtgtctctgac 26
           |||||||||||||||||||
Sbjct: 791 aataatacgtgtctctgac 773
```

>JP326238|JP326238.1 TSA: *Lepeophtheirus salmonis* lsal-pac-6256192 mRNA sequence. Length = 762. Score = 38.2 bits (19), Expect = 3.0; Identities = 19/19 (100%); Strand = Plus / Minus

```
Query: 8   aataatacgtgtctctgac 26
           |||||||||||||||||||
Sbjct: 756 aataatacgtgtctctgac 738
```

>JP326203|JP326203.1 TSA: *Lepeophtheirus salmonis* lsal-pac-6256157 mRNA sequence. Length = 759. Score = 38.2 bits (19), Expect = 3.0; Identities = 19/19 (100%); Strand = Plus / Plus

```
Query: 8  aataatacgtgtctctgac 26
          |||||||||||||||||||
Sbjct: 8  aataatacgtgtctctgac 26
```

>JP326098|JP326098.1 TSA: *Lepeophtheirus salmonis* lsal-pac-6256052 mRNA sequence. Length = 750. Score = 38.2 bits (19), Expect = 3.0; Identities = 19/19 (100%); Strand = Plus / Plus

```
Query: 8  aataatacgtgtctctgac 26
          |||||||||||||||||||
Sbjct: 10 aataatacgtgtctctgac 28
```

>JP326078|JP326078.1 TSA: *Lepeophtheirus salmonis* lsal-pac-6256032 mRNA sequence. Length = 748. Score = 38.2 bits (19), Expect = 3.0; Identities = 19/19 (100%); Strand = Plus / Plus

```
Query: 8  aataatacgtgtctctgac 26
          |||||||||||||||||||
Sbjct: 9  aataatacgtgtctctgac 27
```

>JP325687|JP325687.1 TSA: *Lepeophtheirus salmonis* lsal-pac-6255641 mRNA sequence. Length = 717. Score = 38.2 bits (19), Expect = 3.0; Identities = 19/19 (100%); Strand = Plus / Plus

```
Query: 8  aataatacgtgtctctgac 26
          |||||||||||||||||||
Sbjct: 19 aataatacgtgtctctgac 37
```

>JP325630|JP325630.1 TSA: *Lepeophtheirus salmonis* lsal-pac-6255584 mRNA sequence. Length = 712. Score = 38.2 bits (19), Expect = 3.0; Identities = 19/19 (100%); Strand = Plus / Minus

```
Query: 8   aataatacgtgtctctgac 26
           |||||||||||||||||||
Sbjct: 704 aataatacgtgtctctgac 686
```

>JP324975|JP324975.1 TSA: *Lepeophtheirus salmonis* lsal-pac-6254929 mRNA sequence. Length = 670. Score = 38.2 bits (19), Expect = 3.0; Identities = 19/19 (100%); Strand = Plus / Minus

```
Query: 8   aataatacgtgtctctgac 26
           |||||||||||||||||||
Sbjct: 664 aataatacgtgtctctgac 646
```

>JP324802|JP324802.1 TSA: *Lepeophtheirus salmonis* lsal-pac-6254756 mRNA sequence. Length = 659. Score = 38.2 bits (19), Expect = 3.0; Identities = 19/19 (100%); Strand = Plus / Plus

```
Query: 8  aataatacgtgtctctgac 26
          |||||||||||||||||||
Sbjct: 7  aataatacgtgtctctgac 25
```

>JP324175|JP324175.1 TSA: *Lepeophtheirus salmonis* lsal-pac-6254129 mRNA sequence. Length = 625. Score = 38.2 bits (19), Expect = 3.0; Identities = 19/19 (100%); Strand = Plus / Minus

```
Query: 8   aataatacgtgtctctgac 26
           |||||||||||||||||||
Sbjct: 624 aataatacgtgtctctgac 606
```

>JP323974|JP323974.1 TSA: *Lepeophtheirus salmonis* lsal-pac-6253928 mRNA sequence. Length = 615. Score = 38.2 bits (19), Expect = 3.0; Identities = 19/19 (100%); Strand = Plus / Minus

```
Query: 8   aataatacgtgtctctgac 26
           |||||||||||||||||||
Sbjct: 606 aataatacgtgtctctgac 588
```

>JP323500|JP323500.1 TSA: *Lepeophtheirus salmonis* lsal-pac-6253454 mRNA sequence. Length = 595. Score = 38.2 bits (19), Expect = 3.0; Identities = 19/19 (100%); Strand = Plus / Plus

```
Query: 8  aataatacgtgtctctgac 26
          |||||||||||||||||||
Sbjct: 7  aataatacgtgtctctgac 25
```

>JP323339|JP323339.1 TSA: *Lepeophtheirus salmonis* lsal-pac-6253293 mRNA sequence. Length = 588. Score = 38.2 bits (19), Expect = 3.0; Identities = 19/19 (100%); Strand = Plus / Plus

```
Query: 8  aataatacgtgtctctgac 26
          |||||||||||||||||||
Sbjct: 8  aataatacgtgtctctgac 26
```

>JP323046|JP323046.1 TSA: *Lepeophtheirus salmonis* lsal-pac-6253000 mRNA sequence. Length = 576. Score = 38.2 bits (19), Expect = 3.0; Identities = 19/19 (100%); Strand = Plus / Plus

```
Query: 8  aataatacgtgtctctgac 26
          |||||||||||||||||||
Sbjct: 7  aataatacgtgtctctgac 25
```

>JP322641|JP322641.1 TSA: *Lepeophtheirus salmonis* lsal-pac-6252595 mRNA sequence. Length = 562. Score = 38.2 bits (19), Expect = 3.0; Identities = 19/19 (100%); Strand = Plus / Minus

```
Query: 8   aataatacgtgtctctgac 26
           |||||||||||||||||||
Sbjct: 557 aataatacgtgtctctgac 539
```

>JP322583|JP322583.1 TSA: *Lepeophtheirus salmonis* lsal-pac-6252537 mRNA sequence. Length = 560. Score = 38.2 bits (19), Expect = 3.0; Identities = 19/19 (100%); Strand = Plus / Minus

```
Query: 8   aataatacgtgtctctgac 26
           |||||||||||||||||||
Sbjct: 553 aataatacgtgtctctgac 535
```

>JP322320|JP322320.1 TSA: *Lepeophtheirus salmonis* lsal-pac-6252274 mRNA sequence. Length = 552. Score = 38.2 bits (19), Expect = 3.0; Identities = 19/19 (100%); Strand = Plus / Plus

```
Query: 8  aataatacgtgtctctgac 26
          |||||||||||||||||||
Sbjct: 8  aataatacgtgtctctgac 26
```

>JP321802|JP321802.1 TSA: *Lepeophtheirus salmonis* lsal-pac-6251756 mRNA sequence. Length = 536. Score = 38.2 bits (19), Expect = 3.0; Identities = 19/19 (100%); Strand = Plus / Minus

```
Query: 8   aataatacgtgtctctgac 26
           |||||||||||||||||||
Sbjct: 528 aataatacgtgtctctgac 510
```

>JP320771|JP320771.1 TSA: *Lepeophtheirus salmonis* lsal-pac-6250725 mRNA sequence. Length = 509. Score = 38.2 bits (19), Expect = 3.0; Identities = 19/19 (100%); Strand = Plus / Minus

```
Query: 8   aataatacgtgtctctgac 26
           |||||||||||||||||||
Sbjct: 503 aataatacgtgtctctgac 485
```

>JP318531|JP318531.1 TSA: *Lepeophtheirus salmonis* lsal-pac-6248485 mRNA sequence. Length = 464. Score = 38.2 bits (19), Expect = 3.0; Identities = 19/19 (100%); Strand = Plus / Plus

```
Query: 8  aataatacgtgtctctgac 26
          |||||||||||||||||||
Sbjct: 7  aataatacgtgtctctgac 25
```

>JP318385|JP318385.1 TSA: *Lepeophtheirus salmonis* lsal-pac-6248339 mRNA sequence. Length = 462. Score = 38.2 bits (19), Expect = 3.0; Identities = 19/19 (100%); Strand = Plus / Minus

```
Query: 8   aataatacgtgtctctgac 26
           |||||||||||||||||||
Sbjct: 456 aataatacgtgtctctgac 438
```

>JP318150|JP318150.1 TSA: *Lepeophtheirus salmonis* lsal-pac-6248104 mRNA sequence. Length = 457. Score = 38.2 bits (19), Expect = 3.0; Identities = 19/19 (100%); Strand = Plus / Minus

```
Query: 8   aataatacgtgtctctgac 26
           |||||||||||||||||||
Sbjct: 449 aataatacgtgtctctgac 431
```

>JP315348|JP315348.1 TSA: *Lepeophtheirus salmonis* lsal-pac-6245301 mRNA sequence. Length = 417. Score = 38.2 bits (19), Expect = 3.0; Identities = 19/19 (100%); Strand = Plus / Minus

```
Query: 8   aataatacgtgtctctgac 26
           |||||||||||||||||||
Sbjct: 411 aataatacgtgtctctgac 393
```

>JP314800|JP314800.1 TSA: *Lepeophtheirus salmonis* lsal-pac-6244753 mRNA sequence. Length = 411. Score = 38.2 bits (19), Expect = 3.0; Identities = 19/19 (100%); Strand = Plus / Minus

```
Query: 8   aataatacgtgtctctgac 26
           |||||||||||||||||||
Sbjct: 402 aataatacgtgtctctgac 384
```

>JP314529|JP314529.1 TSA： *Lepeophtheirus salmonis* lsal-pac-6244482 mRNA sequence. Length = 408. Score = 38.2 bits (19), Expect = 3.0; Identities = 19/19 (100%); Strand = Plus / Plus

```
Query： 8  aataatacgtgtctctgac 26
           |||||||||||||||||||
Sbjct： 10 aataatacgtgtctctgac 28
```

>JP314184|JP314184.1 TSA： *Lepeophtheirus salmonis* lsal-pac-6244137 mRNA sequence. Length = 404. Score = 38.2 bits (19), Expect = 3.0; Identities = 19/19 (100%); Strand = Plus / Plus

```
Query： 8  aataatacgtgtctctgac 26
           |||||||||||||||||||
Sbjct： 8  aataatacgtgtctctgac 26
```

>JP314114|JP314114.1 TSA： *Lepeophtheirus salmonis* lsal-pac-6244067 mRNA sequence. Length = 403. Score = 38.2 bits (19), Expect = 3.0; Identities = 19/19 (100%); Strand = Plus / Plus

```
Query： 8  aataatacgtgtctctgac 26
           |||||||||||||||||||
Sbjct： 8  aataatacgtgtctctgac 26
```

>JP313386|JP313386.1 TSA： *Lepeophtheirus salmonis* lsal-pac-6243339 mRNA sequence. Length = 2913. Score = 38.2 bits (19), Expect = 3.0; Identities = 19/19 (100%); Strand = Plus / Plus

```
Query： 8  aataatacgtgtctctgac 26
           |||||||||||||||||||
Sbjct： 7  aataatacgtgtctctgac 25
```

>JP313254|JP313254.1 TSA： *Lepeophtheirus salmonis* lsal-pac-6243207 mRNA sequence. Length = 3276. Score = 38.2 bits (19), Expect = 3.0; Identities = 19/19 (100%); Strand = Plus / Minus

```
Query： 8    aataatacgtgtctctgac 26
             |||||||||||||||||||
Sbjct： 3257 aataatacgtgtctctgac 3239
```

>JP312437|JP312437.1 TSA： *Lepeophtheirus salmonis* lsal-pac-6242387 mRNA sequence. Length = 2583. Score = 38.2 bits (19), Expect = 3.0; Identities = 19/19 (100%); Strand = Plus / Minus

```
Query： 8    aataatacgtgtctctgac 26
             |||||||||||||||||||
Sbjct： 2578 aataatacgtgtctctgac 2560
```

>JP312370|JP312370.1 TSA: *Lepeophtheirus salmonis* lsal-pac-6242320 mRNA sequence. Length = 2579. Score = 38.2 bits (19), Expect = 3.0; Identities = 19/19 (100%); Strand = Plus / Minus

```
Query: 8    aataatacgtgtctctgac 26
            |||||||||||||||||||
Sbjct: 2572 aataatacgtgtctctgac 2554
```

>JP312325|JP312325.1 TSA: *Lepeophtheirus salmonis* lsal-pac-6242275 mRNA sequence. Length = 2959. Score = 38.2 bits (19), Expect = 3.0; Identities = 19/19 (100%); Strand = Plus / Minus

```
Query: 8    aataatacgtgtctctgac 26
            |||||||||||||||||||
Sbjct: 2950 aataatacgtgtctctgac 2932
```

>JP312245|JP312245.1 TSA: *Lepeophtheirus salmonis* lsal-pac-6242195 mRNA sequence. Length = 1460. Score = 38.2 bits (19), Expect = 3.0; Identities = 19/19 (100%); Strand = Plus / Minus

```
Query: 8    aataatacgtgtctctgac 26
            |||||||||||||||||||
Sbjct: 1453 aataatacgtgtctctgac 1435
```

>JP311726|JP311726.1 TSA: *Lepeophtheirus salmonis* lsal-pac-6241675 mRNA sequence. Length = 2097. Score = 38.2 bits (19), Expect = 3.0; Identities = 19/19 (100%); Strand = Plus / Plus

```
Query: 8  aataatacgtgtctctgac 26
          |||||||||||||||||||
Sbjct: 7  aataatacgtgtctctgac 25
```

>JP311630|JP311630.1 TSA: *Lepeophtheirus salmonis* lsal-pac-6241579 mRNA sequence. Length = 2056. Score = 38.2 bits (19), Expect = 3.0; Identities = 19/19 (100%); Strand = Plus / Minus

```
Query: 8    aataatacgtgtctctgac 26
            |||||||||||||||||||
Sbjct: 2037 aataatacgtgtctctgac 2019
```

>JP310373|JP310373.1 TSA: *Lepeophtheirus salmonis* lsal-pac-6240319 mRNA sequence. Length = 1916. Score = 38.2 bits (19), Expect = 3.0; Identities = 19/19 (100%); Strand = Plus / Minus

```
Query: 8    aataatacgtgtctctgac 26
            |||||||||||||||||||
Sbjct: 1895 aataatacgtgtctctgac 1877
```

>JP310288|JP310288.1 TSA: *Lepeophtheirus salmonis* lsal-pac-6240234 mRNA sequence. Length = 2091. Score = 38.2 bits (19), Expect = 3.0; Identities = 19/19 (100%); Strand = Plus / Minus

```
Query: 8    aataatacgtgtctctgac 26
            |||||||||||||||||||
Sbjct: 2088 aataatacgtgtctctgac 2070
```

>JP309468|JP309468.1 TSA: *Lepeophtheirus salmonis* lsal-pac-6239413 mRNA sequence. Length = 1052. Score = 38.2 bits (19), Expect = 3.0; Identities = 19/19 (100%); Strand = Plus / Plus

```
Query: 8  aataatacgtgtctctgac 26
          |||||||||||||||||||
Sbjct: 7  aataatacgtgtctctgac 25
```

>JP309166|JP309166.1 TSA: *Lepeophtheirus salmonis* lsal-pac-6239111 mRNA sequence. Length = 1012. Score = 38.2 bits (19), Expect = 3.0; Identities = 19/19 (100%); Strand = Plus / Plus

```
Query: 8  aataatacgtgtctctgac 26
          |||||||||||||||||||
Sbjct: 10 aataatacgtgtctctgac 28
```

>JP307790|JP307790.1 TSA: *Lepeophtheirus salmonis* lsal-pac-6237732 mRNA sequence. Length = 2187. Score = 38.2 bits (19), Expect = 3.0; Identities = 19/19 (100%); Strand = Plus / Minus

```
Query: 8    aataatacgtgtctctgac 26
            |||||||||||||||||||
Sbjct: 2180 aataatacgtgtctctgac 2162
```

>JP307643|JP307643.1 TSA: *Lepeophtheirus salmonis* lsal-pac-6237585 mRNA sequence. Length = 1512. Score = 38.2 bits (19), Expect = 3.0; Identities = 19/19 (100%); Strand = Plus / Minus

```
Query: 8    aataatacgtgtctctgac 26
            |||||||||||||||||||
Sbjct: 1493 aataatacgtgtctctgac 1475
```

>JP307629|JP307629.1 TSA: *Lepeophtheirus salmonis* lsal-pac-6237571 mRNA sequence. Length = 1469. Score = 38.2 bits (19), Expect = 3.0; Identities = 19/19 (100%); Strand = Plus / Plus

```
Query: 8  aataatacgtgtctctgac 26
          |||||||||||||||||||
Sbjct: 18 aataatacgtgtctctgac 36
```

>JP307527|JP307527.1 TSA: *Lepeophtheirus salmonis* lsal-pac-6237469 mRNA sequence. Length = 1284. Score = 38.2 bits (19), Expect = 3.0; Identities = 19/19 (100%); Strand = Plus / Minus

```
Query: 8    aataatacgtgtctctgac 26
            |||||||||||||||||||
Sbjct: 1271 aataatacgtgtctctgac 1253
```

>JP307470|JP307470.1 TSA: *Lepeophtheirus salmonis* lsal-pac-6237412 mRNA sequence. Length = 1830. Score = 38.2 bits (19), Expect = 3.0; Identities = 19/19 (100%); Strand = Plus / Plus

```
Query: 8  aataatacgtgtctctgac 26
          |||||||||||||||||||
Sbjct: 8  aataatacgtgtctctgac 26
```

>JP307336|JP307336.1 TSA: *Lepeophtheirus salmonis* lsal-pac-6237278 mRNA sequence. Length = 1037. Score = 38.2 bits (19), Expect = 3.0; Identities = 19/19 (100%); Strand = Plus / Plus

```
Query: 8  aataatacgtgtctctgac 26
          |||||||||||||||||||
Sbjct: 19 aataatacgtgtctctgac 37
```

>JP307066|JP307066.1 TSA: *Lepeophtheirus salmonis* lsal-pac-6237008 mRNA sequence. Length = 1012. Score = 38.2 bits (19), Expect = 3.0; Identities = 19/19 (100%); Strand = Plus / Plus

```
Query: 8  aataatacgtgtctctgac 26
          |||||||||||||||||||
Sbjct: 9  aataatacgtgtctctgac 27
```

>JP306747|JP306747.1 TSA: *Lepeophtheirus salmonis* lsal-pac-6236689 mRNA sequence. Length = 1273. Score = 38.2 bits (19), Expect = 3.0; Identities = 19/19 (100%); Strand = Plus / Plus

```
Query: 8  aataatacgtgtctctgac 26
          |||||||||||||||||||
Sbjct: 8  aataatacgtgtctctgac 26
```

>JP306485|JP306485.1 TSA: *Lepeophtheirus salmonis* lsal-pac-6236427 mRNA sequence. Length = 1473. Score = 38.2 bits (19), Expect = 3.0; Identities = 19/19 (100%); Strand = Plus / Plus

```
Query: 8  aataatacgtgtctctgac 26
          |||||||||||||||||||
Sbjct: 10 aataatacgtgtctctgac 28
```

>JP305957|JP305957.1 TSA: *Lepeophtheirus salmonis* lsal-pac-6235898 mRNA sequence. Length = 465. Score = 38.2 bits (19), Expect = 3.0; Identities = 19/19 (100%); Strand = Plus / Minus

```
Query: 8   aataatacgtgtctctgac 26
           |||||||||||||||||||
Sbjct: 457 aataatacgtgtctctgac 439
```

>JP299361|JP299361.1 TSA: *Lepeophtheirus salmonis* lsal-pac-6229292 mRNA sequence. Length = 1299. Score = 38.2 bits (19), Expect = 3.0; Identities = 19/19 (100%); Strand = Plus / Plus

```
Query: 8  aataatacgtgtctctgac 26
          |||||||||||||||||||
Sbjct: 19 aataatacgtgtctctgac 37
```

>BT121189|BT121189.1 *Lepeophtheirus salmonis* Atlantic form clone lsaA-evu-509-337 N6-adenine-specific DNA methyltransferase 2 putative mRNA, complete cds. Length = 898. Score = 38.2 bits (19), Expect = 3.0; Identities = 19/19 (100%); Strand = Plus / Plus

```
Query: 8  aataatacgtgtctctgac 26
          |||||||||||||||||||
Sbjct: 17 aataatacgtgtctctgac 35
```

>BT120800|BT120800.1 *Lepeophtheirus salmonis* clone lsal-evj-021-066 Actin-related protein 10 putative mRNA, complete cds. Length = 1307. Score = 38.2 bits (19), Expect = 3.0; Identities = 19/19 (100%); Strand = Plus / Plus

```
Query: 8  aataatacgtgtctctgac 26
          |||||||||||||||||||
Sbjct: 19 aataatacgtgtctctgac 37
```

>FK893984|FK893984.1 EST_crog_evm_804246 crog_evm *Caligus rogercresseyi* cDNA clone crog_evm_505_246_rev 5', mRNA Sequence. Length = 643. Score = 48.1 bits (24), Expect = 0.003; Identities = 24/24 (100%); Strand = Plus / Minus

```
Query: 13  tacgtgtctctgacaaaaatcaag 36
           ||||||||||||||||||||||||
Sbjct: 638 tacgtgtctctgacaaaaatcaag 615
```

>FK893983|FK893983.1 EST_crog_evm_816726 crog_evm *Caligus rogercresseyi* cDNA clone crog_evm_505_246_fwd 5', mRNA sequence. Length = 643. Score = 48.1 bits (24), Expect = 0.003; Identities = 24/24 (100%); Strand = Plus / Plus

```
Query: 13 tacgtgtctctgacaaaaatcaag 36
          ||||||||||||||||||||||||
```

Sbjct: 6 tacgtgtctctgacaaaaatcaag 29

>FK878825|FK878825.1 EST_crog_evp_913303 crog_evp *Caligus rogercresseyi* cDNA clone crog_evp_515_055_fwd 5', mRNA sequence. Length = 535. Score = 48.1 bits (24), Expect = 0.003; Identities = 24/24 (100%); Strand = Plus / Plus

Query: 13 tacgtgtctctgacaaaaatcaag 36
         ||||||||||||||||||||||||
Sbjct: 5 tacgtgtctctgacaaaaatcaag 28

>FK877065|FK877065.1 EST_crog_evp_901228 crog_evp *Caligus rogercresseyi* cDNA clone crog_evp_512_268_rev 5', mRNA Sequence. Length = 643. Score = 48.1 bits (24), Expect = 0.003; Identities = 24/24 (100%); Strand = Plus / Minus

Query: 13 tacgtgtctctgacaaaaatcaag 36
          ||||||||||||||||||||||||
Sbjct: 639 tacgtgtctctgacaaaaatcaag 616

>BT077244|BT077244.1 *Caligus rogercresseyi* clone crog-evp-515-055 C9orf85 putative mRNA, complete cds. Length = 535. Score = 48.1 bits (24), Expect = 0.003; Identities = 24/24 (100%); Strand = Plus / Plus

Query: 13 tacgtgtctctgacaaaaatcaag 36
         ||||||||||||||||||||||||
Sbjct: 5 tacgtgtctctgacaaaaatcaag 28

>FK893997|FK893997.1 EST_crog_evm_816733 crog_evm *Caligus rogercresseyi* cDNA clone crog_evm_505_253_fwd 5', mRNA sequence. Length = 924. Score = 38.2 bits (19), Expect = 3.0; Identities = 19/19 (100%); Strand = Plus / Plus

Query: 18 gtctctgacaaaaatcaag 36
         |||||||||||||||||||
Sbjct: 5 gtctctgacaaaaatcaag 23

>BT077054|BT077054.1 *Caligus rogercresseyi* clone crog-evm-505-253 Probable U3 small nucleolar RNA-associated protein 11 putative mRNA, complete cds. Length = 922. Score = 38.2 bits (19), Expect = 3.0; Identities = 19/19 (100%); Strand = Plus / Plus

Query: 18 gtctctgacaaaaatcaag 36
         |||||||||||||||||||
Sbjct: 5 gtctctgacaaaaatcaag 23

>HO668861|HO668861.1 542_FN1785_E11_054.ab1 Lepeophtheirus salmonis ME Lepeophtheirus salmonis cDNA, mRNA sequence. Length = 734. Score = 58.0 bits (29), Expect = 3e-06. Identities = 29/29 (100%). Strand = Plus / Plus

```
Query: 9  ataatacgtgtctctgactaataatcaag 37
           |||||||||||||||||||||||||||||
Sbjct: 56 ataatacgtgtctctgactaataatcaag 84
```

>HO667886|HO667886.1 HAV-FN-FN2279-2001-11-30.ab1 Lepeophtheirus salmonis ME Lepeophtheirus salmonis cDNA, mRNA sequence. Length = 651. Score = 58.0 bits (29), Expect = 3e-06. Identities = 32/33 (96%). Strand = Plus / Plus

```
Query: 5  gtaaataatacgtgtctctgactaataatcaag 37
           |||||||||| ||||||||||||||||||||||
Sbjct: 40 gtaaataataggtgtctctgactaataatcaag 72
```

>JP329187|JP329187.1 TSA: Lepeophtheirus salmonis lsal-pac-6259143 mRNA sequence. Length = 1691. Score = 56.0 bits (28), Expect = 1e-05. Identities = 28/28 (100%). Strand = Plus / Minus

```
Query: 10   taatacgtgtctctgactaataatcaag 37
             ||||||||||||||||||||||||||||
Sbjct: 1691 taatacgtgtctctgactaataatcaag 1664
```

>HO696733|HO696733.1 LPU952-2007-08-29.ab1 Lepeophtheirus salmonis LPU Lepeophtheirus salmonis cDNA, mRNA sequence. Length = 826. Score = 54.0 bits (27), Expect = 5e-05. Identities = 27/27 (100%). Strand = Plus / Plus

```
Query: 11  aatacgtgtctctgactaataatcaag 37
            |||||||||||||||||||||||||||
Sbjct: 82  aatacgtgtctctgactaataatcaag 108
```

>HO675736|HO675736.1 LF1850-2007-05-08.ab1 Lepeophtheirus salmonis LF Lepeophtheirus salmonis cDNA, mRNA sequence. Length = 814. Score = 54.0 bits (27), Expect = 5e-05. Identities = 27/27 (100%). Strand = Plus / Plus

```
Query: 11  aatacgtgtctctgactaataatcaag 37
            |||||||||||||||||||||||||||
Sbjct: 86  aatacgtgtctctgactaataatcaag 112
```

>HO675725|HO675725.1 LF1839-2007-05-08.ab1 Lepeophtheirus salmonis LF Lepeophtheirus salmonis cDNA, mRNA sequence. Length = 850. Score = 54.0 bits (27), Expect = 5e-05. Identities = 27/27 (100%). Strand = Plus / Plus

```
Query: 11  aatacgtgtctctgactaataatcaag 37
            |||||||||||||||||||||||||||
Sbjct: 84  aatacgtgtctctgactaataatcaag 110
```

>JP345740|JP345740.1 TSA: Lepeophtheirus salmonis lsal-atl-6223347mRNA sequence. Length = 710. Score = 52.0 bits (26), Expect = 2e-04. Identities = 26/26 (100%). Strand = Plus / Minus
Query: 12 atacgtgtctctgactaataatcaag 37
            ||||||||||||||||||||||||||
Sbjct: 710 atacgtgtctctgactaataatcaag 685

>HO669414|HO669414.1 HAV-FN-FN1881-2001-11-28.ab1 Lepeophtheirus salmonis ME Lepeophtheirus salmonis cDNA, mRNA sequence. Length = 601. Score = 50.1 bits (25), Expect = 8e-04. Identities = 28/29 (96%). Strand = Plus / Plus
Query: 9 ataatacgtgtctctgactaataatcaag 37
          |||||||||||||||||| ||||||||||
Sbjct: 47 ataatacgtgtctctgacgaataatcaag 75

>EY509570|EY509570.1 EST_lsal_pf_801967 lsalpf whole; Lepeophtheirus salmonis (Pacific) cDNA Lepeophtheirus salmonis cDNA clone lsal_pf_6_367_rev 5', mRNA sequence. Length = 819. Score = 50.1 bits (25), Expect = 8e-04. Identities = 28/29 (96%). Strand = Plus / Plus
Query: 9 ataatacgtgtctctgactaataatcaag 37
          |||||||||||||||||| ||||||||||
Sbjct: 9 ataatacgtgtctctgacgaataatcaag 37

>EY509347|EY509347.1 EST_lsal_pf_801797 lsalpf whole; Lepeophtheirus salmonis (Pacific) cDNA Lepeophtheirus salmonis cDNA clone lsal_pf_6_137_rev 5', mRNA sequence. Length = 819. Score = 50.1 bits (25), Expect = 8e-04. Identities = 28/29 (96%). Strand = Plus / Plus
Query: 9 ataatacgtgtctctgactaataatcaag 37
          |||||||||||||||||| ||||||||||
Sbjct: 9 ataatacgtgtctctgacgaataatcaag 37

>JP345202|JP345202.1 TSA: Lepeophtheirus salmonis lsal-atl-6222809mRNA sequence. Length = 665. Score = 50.1 bits (25), Expect = 8e-04. Identities = 25/25 (100%). Strand = Plus / Minus
Query: 13 tacgtgtctctgactaataatcaag 37
            |||||||||||||||||||||||||
Sbjct: 665 tacgtgtctctgactaataatcaag 641

>JP344038|JP344038.1 TSA: Lepeophtheirus salmonis lsal-atl-6221645mRNA sequence. Length = 597. Score = 50.1 bits (25), Expect = 8e-04. Identities = 25/25 (100%). Strand = Plus / Minus
Query: 13 tacgtgtctctgactaataatcaag 37
            |||||||||||||||||||||||||
Sbjct: 597 tacgtgtctctgactaataatcaag 573

>JP341708|JP341708.1 TSA: Lepeophtheirus salmonis lsal-atl-6219315 mRNA sequence. Length = 513. Score = 50.1 bits (25), Expect = 8e-04. Identities = 25/25 (100%). Strand = Plus / Plus
Query: 13 tacgtgtctctgactaataatcaag 37
           |||||||||||||||||||||||||

Sbjct: 1 tacgtgtctctgactaataatcaag 25

>JP304090|JP304090.1 TSA: Lepeophtheirus salmonis lsal-pac-6234029 mRNA sequence. Length = 1043. Score = 50.1 bits (25), Expect = 8e-04. Identities = 28/29 (96%). Strand = Plus / Plus

```
Query: 9  ataatacgtgtctctgactaataatcaag 37
          |||||||||||||||||| ||||||||||
Sbjct: 9  ataatacgtgtctctgacgaataatcaag 37
```

>HO673382|HO673382.1 L1T_1269-2007-01-15.ab1 Lepeophtheirus salmonis L1T Lepeophtheirus salmonis cDNA, mRNA sequence. Length = 836. Score = 48.1 bits (24), Expect = 0.003. Identities = 24/24 (100%). Strand = Plus / Plus

```
Query: 14  acgtgtctctgactaataatcaag 37
           ||||||||||||||||||||||||
Sbjct: 84  acgtgtctctgactaataatcaag 107
```

>HO669542|HO669542.1 FN3713-2003-01-16.ab1 Lepeophtheirus salmonis ME Lepeophtheirus salmonis cDNA, mRNA sequence. Length = 291. Score = 48.1 bits (24), Expect = 0.003. Identities = 27/28 (96%). Strand = Plus / Plus

```
Query: 10 taatacgtgtctctgactaataatcaag 37
          ||||||||||||||||| ||||||||||
Sbjct: 60 taatacgtgtctctgacgaataatcaag 87
```

>HO668574|HO668574.1 HAV-FN-FN2493-2001-12-10.ab1 Lepeophtheirus salmonis ME Lepeophtheirus salmonis cDNA, mRNA sequence. Length = 561. Score = 48.1 bits (24), Expect = 0.003. Identities = 27/28 (96%). Strand = Plus / Plus

```
Query: 10 taatacgtgtctctgactaataatcaag 37
          ||||||||||||||||| ||||||||||
Sbjct: 41 taatacgtgtctctgacgaataatcaag 68
```

>HO667678|HO667678.1 HAV-FN-FN1963-2001-11-30.ab1 Lepeophtheirus salmonis ME Lepeophtheirus salmonis cDNA, mRNA sequence. Length = 773. Score = 48.1 bits (24), Expect = 0.003. Identities = 27/28 (96%). Strand = Plus / Plus

```
Query: 10 taatacgtgtctctgactaataatcaag 37
          ||||||||||||||||| ||||||||||
Sbjct: 46 taatacgtgtctctgacgaataatcaag 73
```

>HO667631|HO667631.1 HAV-FN-FN1915-2001-11-30.ab1 Lepeophtheirus salmonis ME Lepeophtheirus salmonis cDNA, mRNA sequence. Length = 726. Score = 48.1 bits (24), Expect = 0.003. Identities = 27/28 (96%). Strand = Plus / Plus

```
Query: 10 taatacgtgtctctgactaataatcaag 37
          ||||||||||||||||| ||||||||||
Sbjct: 53 taatacgtgtctctgacgaataatcaag 80
```

>JP336860|JP336860.1 TSA: Lepeophtheirus salmonis lsal-atl-6214467mRNA sequence. Length = 419. Score = 48.1 bits (24), Expect = 0.003. Identities = 24/24 (100%). Strand = Plus / Minus

```
Query: 14  acgtgtctctgactaataatcaag 37
           ||||||||||||||||||||||||
Sbjct: 419 acgtgtctctgactaataatcaag 396
```

>HO697674|HO697674.1 LPU1375-2007-09-12.ab1 Lepeophtheirus salmonis LPU Lepeophtheirus salmonis cDNA, mRNA sequence. Length = 885. Score = 46.1 bits (23), Expect = 0.013. Identities = 23/23 (100%). Strand = Plus / Plus

```
Query: 15 cgtgtctctgactaataatcaag 37
          |||||||||||||||||||||||
Sbjct: 68 cgtgtctctgactaataatcaag 90
```

>HO697527|HO697527.1 LPU2012-2007-09-06.ab1 Lepeophtheirus salmonis LPU Lepeophtheirus salmonis cDNA, mRNA sequence. Length = 784. Score = 46.1 bits (23), Expect = 0.013. Identities = 26/27 (96%). Strand = Plus / Plus

```
Query: 11  aatacgtgtctctgactaataatcaag 37
            |||||||||||||||| ||||||||||
Sbjct: 78  aatacgtgtctctgacgaataatcaag 104
```

>HO672198|HO672198.1 HA681-2005-02-24.ab1 Lepeophtheirus salmonis HA; Lepeophtheirus salmonis cDNA, mRNA sequence. Length = 315. Score = 46.1 bits (23), Expect = 0.013. Identities = 26/27 (96%). Strand = Plus / Plus

```
Query: 11  aatacgtgtctctgactaataatcaag 37
            |||||||||||||||| ||||||||||
Sbjct: 94  aatacgtgtctctgacgaataatcaag 120
```

>EY509004|EY509004.1 EST_lsal_pf_787786 lsalpf whole; Lepeophtheirus salmonis (Pacific) cDNA Lepeophtheirus salmonis cDNA clone lsal_pf_5_106_rev 5', mRNA sequence. Length = 877. Score = 46.1 bits (23), Expect = 0.013. Identities = 26/27 (96%). Strand = Plus / Plus

```
Query: 11 aatacgtgtctctgactaataatcaag 37
          |||||||||||||||| ||||||||||
Sbjct: 1  aatacgtgtctctgacgaataatcaag 27
```

>JP342580|JP342580.1 TSA: Lepeophtheirus salmonis lsal-atl-6220187 mRNA sequence. Length = 540. Score = 46.1 bits (23), Expect = 0.013. Identities = 26/27 (96%). Strand = Plus / Plus

```
Query: 11 aatacgtgtctctgactaataatcaag 37
          |||||||||||||||| ||||||||||
Sbjct: 1  aatacgtgtctctgacgaataatcaag 27
```

>HO675589|HO675589.1 LF1593-2007-05-08.ab1 Lepeophtheirus salmonis LF Lepeophtheirus salmonis cDNA, mRNA sequence. Length = 715. Score = 44.1 bits (22), Expect = 0.051. Identities = 22/22 (100%). Strand = Plus / Plus

```
Query: 16 gtgtctctgactaataatcaag 37
          ||||||||||||||||||||||
Sbjct: 72 gtgtctctgactaataatcaag 93
```

>HO670379|HO670379.1 FN4544-2003-03-05.ab1 Lepeophtheirus salmonis ME Lepeophtheirus salmonis cDNA, mRNA sequence. Length = 66. Score = 44.1 bits (22), Expect = 0.051. Identities = 22/22 (100%). Strand = Plus / Plus

```
Query: 15 cgtgtctctgactaataatcaa 36
          ||||||||||||||||||||||
Sbjct: 45 cgtgtctctgactaataatcaa 66
```

>GW636740|GW636740.1 EST_lsaA_evu_1221358 lsaAevu mixed_tissue; Lepeophtheirus salmonis (Atlantic Canada) cDNA; Lepeophtheirus salmonis cDNA clone lsaA_evu_501_076 5’, mRNA sequence. Length = 952. Score = 44.1 bits (22), Expect = 0.051. Identities = 22/22 (100%). Strand = Plus / Plus

```
Query: 16 gtgtctctgactaataatcaag 37
          ||||||||||||||||||||||
Sbjct: 5  gtgtctctgactaataatcaag 26
```

>EY509548|EY509548.1 EST_lsal_pf_801950 lsalpf whole; Lepeophtheirus salmonis (Pacific) cDNA Lepeophtheirus salmonis cDNA clone lsal_pf_6_340_rev 5’, mRNA sequence. Length = 723. Score = 44.1 bits (22), Expect = 0.051. Identities = 22/22 (100%). Strand = Plus / Plus

```
Query: 16 gtgtctctgactaataatcaag 37
          ||||||||||||||||||||||
Sbjct: 9  gtgtctctgactaataatcaag 30
```

>EY509313|EY509313.1 EST_lsal_pf_801626 lsalpf whole; Lepeophtheirus salmonis (Pacific) cDNA Lepeophtheirus salmonis cDNA clone lsal_pf_6_99_rev 5’, mRNA sequence. Length = 799. Score = 44.1 bits (22), Expect = 0.051. Identities = 25/26 (96%). Strand = Plus / Plus

```
Query: 12 atacgtgtctctgactaataatcaag 37
          ||||||||||||||| ||||||||||
Sbjct: 9  atacgtgtctctgacgaataatcaag 34
```

>EY509093|EY509093.1 EST_lsal_pf_787883 lsalpf whole; Lepeophtheirus salmonis (Pacific) cDNA Lepeophtheirus salmonis cDNA clone lsal_pf_5_203_rev 5’, mRNA sequence. Length = 871. Score = 44.1 bits (22), Expect = 0.051. Identities = 22/22 (100%). Strand = Plus / Plus

```
Query: 16 gtgtctctgactaataatcaag 37
          ||||||||||||||||||||||
Sbjct: 9  gtgtctctgactaataatcaag 30
```

>JP344396|JP344396.1 TSA: Lepeophtheirus salmonis lsal-atl-6222003mRNA sequence. Length = 615. Score = 44.1 bits (22), Expect = 0.051. Identities = 25/26 (96%). Strand = Plus / Minus
Query: 12 atacgtgtctctgactaataatcaag 37
           ||||||||||||||| ||||||||||
Sbjct: 615 atacgtgtctctgacgaataatcaag 590

>JP331262|JP331262.1 TSA: Lepeophtheirus salmonis lsal-pac-6261218mRNA sequence. Length = 552. Score = 44.1 bits (22), Expect = 0.051. Identities = 31/34 (91%). Strand = Plus / Plus
Query: 4   agtaaataatacgtgtctctgactaataatcaag 37
           |||||||||||||| |||| ||| ||||||||||
Sbjct: 418 agtaaataatacgtatctcggacaaataatcaag 451

>JP311665|JP311665.1 TSA: Lepeophtheirus salmonis lsal-pac-6241614 mRNA sequence. Length = 1959. Score = 44.1 bits (22), Expect = 0.051. Identities = 22/22 (100%). Strand = Plus / Plus
Query: 16 gtgtctctgactaataatcaag 37
          ||||||||||||||||||||||
Sbjct: 9 gtgtctctgactaataatcaag 30

>JP347648|JP347648.1 TSA: Lepeophtheirus salmonis lsal-atl-6225255 mRNA sequence. Length = 1197. Score = 42.1 bits (21), Expect = 0.20. Identities = 24/25 (96%). Strand = Plus / Plus
Query: 13 tacgtgtctctgactaataatcaag 37
          |||||||||||||| ||||||||||
Sbjct: 1 tacgtgtctctgacgaataatcaag 25

>JP343439|JP343439.1 TSA: Lepeophtheirus salmonis lsal-atl-6221046mRNA sequence. Length = 571. Score = 42.1 bits (21), Expect = 0.20. Identities = 24/25 (96%). Strand = Plus / Minus
Query: 13 tacgtgtctctgactaataatcaag 37
           |||||||||||||| ||||||||||
Sbjct: 571 tacgtgtctctgacgaataatcaag 547

>HO671245|HO671245.1 FN5709-2005-02-14.ab1 Lepeophtheirus salmonis ME Lepeophtheirus salmonis cDNA, mRNA sequence. Length = 565. Score = 40.1 bits (20), Expect = 0.80. Identities = 20/20 (100%). Strand = Plus / Plus
Query: 18 gtctctgactaataatcaag 37
          ||||||||||||||||||||
Sbjct: 63 gtctctgactaataatcaag 82

>HO668748|HO668748.1 HAV-FN-FN2662-2001-12-13.ab1 Lepeophtheirus salmonis ME Lepeophtheirus salmonis cDNA, mRNA sequence. Length = 606. Score = 40.1 bits (20), Expect = 0.80. Identities = 20/20 (100%). Strand = Plus / Plus
Query: 18 gtctctgactaataatcaag 37
          ||||||||||||||||||||
Sbjct: 35 gtctctgactaataatcaag 54

>HO667942|HO667942.1 FN2936-2002-02-06.ab1 Lepeophtheirus salmonis ME Lepeophtheirus salmonis cDNA, mRNA sequence. Length = 616. Score = 40.1 bits (20), Expect = 0.80. Identities = 23/24 (95%). Strand = Plus / Plus
Query： 14 acgtgtctctgactaataatcaag 37
          |||||| |||||||||||||||||
Sbjct： 69 acgtgtgtctgactaataatcaag 92

>HO696138|HO696138.1 zslaa0_019217.z1.scf Lepeophtheirus salmonis LNO Lepeophtheirus salmonis cDNA, mRNA sequence. Length = 429. Score = 38.2 bits (19), Expect = 3.1. Identities = 25/27 (92%). Strand = Plus / Plus
Query： 11 aatacgtgtctctgactaataatcaag 37
           ||||||||||||||| ||| |||||||
Sbjct： 345 aatacgtgtctctgaataacaatcaag 371

>HO689895|HO689895.1 zslaa0_010561.z1.scf Lepeophtheirus salmonis LNO Lepeophtheirus salmonis cDNA, mRNA sequence. Length = 549. Score = 38.2 bits (19), Expect = 3.1. Identities = 25/27 (92%). Strand = Plus / Plus
Query： 11 aatacgtgtctctgactaataatcaag 37
           ||||||||||||||| ||| |||||||
Sbjct： 202 aatacgtgtctctgaataacaatcaag 228

>HO667319|HO667319.1 472_FN-1095_C03_024.ab1 Lepeophtheirus salmonis FB Lepeophtheirus salmonis cDNA, mRNA sequence. Length = 734. Score = 38.2 bits (19), Expect = 3.1. Identities = 22/23 (95%). Strand = Plus / Plus
Query： 15 cgtgtctctgactaataatcaag 37
          |||||||||||| ||||||||||
Sbjct： 56 cgtgtctctgacgaataatcaag 78

>GW641968|GW641968.1 EST_lsaA_evu_1217915 lsaAevu mixed_tissue; Lepeophtheirus salmonis (Atlantic Canada) cDNA; Lepeophtheirus salmonis cDNA clone lsaA_evu_508_089 5', mRNA sequence. Length = 906. Score = 38.2 bits (19), Expect = 3.1. Identities = 22/23 (95%). Strand = Plus / Plus
Query： 15 cgtgtctctgactaataatcaag 37
          |||||||||||| ||||||||||
Sbjct： 5 cgtgtctctgacgaataatcaag 27

>GW638481|GW638481.1 EST_lsaA_evu_1219186 lsaAevu mixed_tissue; Lepeophtheirus salmonis (Atlantic Canada) cDNA; Lepeophtheirus salmonis cDNA clone lsaA_evu_503_208 3', mRNA sequence. Length = 784. Score = 38.2 bits (19), Expect = 3.1. Identities = 25/27 (92%). Strand = Plus / Minus
Query： 11 aatacgtgtctctgactaataatcaag 37
           ||||||||||||||| ||| |||||||

Sbjct: 712 aatacgtgtctctgaataacaatcaag 686

>GW638480|GW638480.1 EST_lsaA_evu_1221106 lsaAevu mixed_tissue; Lepeophtheirus salmonis (Atlantic Canada) cDNA; Lepeophtheirus salmonis cDNA clone lsaA_evu_503_208 5', mRNA sequence. Length = 882. Score = 38.2 bits (19), Expect = 3.1. Identities = 25/27 (92%). Strand = Plus / Plus

```
Query: 11  aatacgtgtctctgactaataatcaag 37
           ||||||||||||||| ||| |||||||
Sbjct: 170 aatacgtgtctctgaataacaatcaag 196
```

>GW622952|GW622952.1 EST_lsal_evj_1199054 lsalevj whole; Lepeophtheirus salmonis (Pacific) cDNA Lepeophtheirus Salmonis cDNA clone lsal_evj_015_236 3', mRNA sequence. Length = 785. Score = 38.2 bits (19), Expect = 3.1. Identities = 25/27 (92%). Strand = Plus / Minus

```
Query: 11  aatacgtgtctctgactaataatcaag 37
           ||||||||||||||| ||| |||||||
Sbjct: 712 aatacgtgtctctgaataacaatcaag 686
```

>GW622951|GW622951.1 EST_lsal_evj_1199438 lsalevj whole; Lepeophtheirus salmonis (Pacific) cDNA Lepeophtheirus Salmonis cDNA clone lsal_evj_015_236 5', mRNA sequence. Length = 842. Score = 38.2 bits (19), Expect = 3.1. Identities = 25/27 (92%). Strand = Plus / Plus

```
Query: 11  aatacgtgtctctgactaataatcaag 37
           ||||||||||||||| ||| |||||||
Sbjct: 124 aatacgtgtctctgaataacaatcaag 150
```

>FK901747|FK901747.1 EST_lsal_evj_853332 lsalevj mixed_tissue_mixed_stage Lepeophtheirus salmonis (Pacific) cDNA Lepeophtheirus salmonis cDNA clone lsal_evj_003_372_fwd 5', mRNA sequence. Length = 350. Score = 38.2 bits (19), Expect = 3.1. Identities = 25/27 (92%). Strand = Plus / Plus

```
Query: 11  aatacgtgtctctgactaataatcaag 37
           ||||||||||||||| ||| |||||||
Sbjct: 123 aatacgtgtctctgaataacaatcaag 149
```

>EY509802|EY509802.1 EST_lsal_pm_787654 lsalpm whole; Lepeophtheirus salmonis (Pacific) cDNA Lepeophtheirus salmonis cDNA clone lsal_pm_3_259_rev 5', mRNA sequence. Length = 885. Score = 38.2 bits (19), Expect = 3.1. Identities = 22/23 (95%). Strand = Plus / Plus

```
Query: 15 cgtgtctctgactaataatcaag 37
          |||||||||||| ||||||||||
Sbjct: 1  cgtgtctctgacgaataatcaag 23
```

>EY509153|EY509153.1 EST_lsal_pf_787950 lsalpf whole; Lepeophtheirus salmonis (Pacific) cDNA Lepeophtheirus salmonis cDNA clone lsal_pf_5_270_rev 5', mRNA sequence. Length = 644. Score = 38.2 bits (19), Expect = 3.1. Identities = 22/23 (95%). Strand = Plus / Plus

```
Query: 15 cgtgtctctgactaataatcaag 37
```

```
          |||||||||||| ||||||||||
Sbjct: 12 cgtgtctctgacgaataatcaag 34
```

>JP348960|JP348960.1 TSA: Lepeophtheirus salmonis lsal-atl-6226567 mRNA sequence. Length = 1383. Score = 38.2 bits (19), Expect = 3.1. Identities = 22/23 (95%). Strand = Plus / Plus

```
Query: 15 cgtgtctctgactaataatcaag 37
          |||||||||||| ||||||||||
Sbjct: 5  cgtgtctctgacgaataatcaag 27
```

>JP348327|JP348327.1 TSA: Lepeophtheirus salmonis lsal-atl-6225934mRNA sequence. Length = 882. Score = 38.2 bits (19), Expect = 3.1. Identities = 25/27 (92%). Strand = Plus / Plus

```
Query: 11  aatacgtgtctctgactaataatcaag 37
           |||||||||||||| ||| |||||||
Sbjct: 170 aatacgtgtctctgaataacaatcaag 196
```

>JP334729|JP334729.1 TSA: Lepeophtheirus salmonis lsal-atl-6212336mRNA sequence. Length = 977. Score = 38.2 bits (19), Expect = 3.1. Identities = 22/23 (95%). Strand = Plus / Minus

```
Query: 15  cgtgtctctgactaataatcaag 37
           |||||||||||| ||||||||||
Sbjct: 977 cgtgtctctgacgaataatcaag 955
```

>JP312656|JP312656.1 TSA: Lepeophtheirus salmonis lsal-pac-6242606mRNA sequence. Length = 844. Score = 38.2 bits (19), Expect = 3.1. Identities = 25/27 (92%). Strand = Plus / Plus

```
Query: 11  aatacgtgtctctgactaataatcaag 37
           |||||||||||||| ||| |||||||
Sbjct: 124 aatacgtgtctctgaataacaatcaag 150
```

>JP307145|JP307145.1 TSA: Lepeophtheirus salmonis lsal-pac-6237087 mRNA sequence. Length = 1092. Score = 38.2 bits (19), Expect = 3.1. Identities = 22/23 (95%). Strand = Plus / Minus

```
Query: 15   cgtgtctctgactaataatcaag 37
            |||||||||||| ||||||||||
Sbjct: 1092 cgtgtctctgacgaataatcaag 1070
```

>FK919252|FK919252.1 EST_lsal_evj_959063 lsalevj mixed_tissue_mixed_stage Lepeophtheirus salmonis (Pacific) cDNA Lepeophtheirus salmonis cDNA clone lsal_evj_536_119_rev 5', mRNA sequence. Length = 552. Score = 44.1 bits (22), Expect = 0.051. Identities = 31/34 (91%). Strand = Plus / Minus

```
Query: 4   agtaaataatacgtgtctctgactaataatcaag 37
           |||||||||||||| |||| ||| ||||||||||
Sbjct: 135 agtaaataatacgtatctcggacaaataatcaag 102
```

>FK919251|FK919251.1 EST_lsal_evj_946007 lsalevj mixed_tissue_mixed_stage Lepeophtheirus salmonis (Pacific) cDNA Lepeophtheirus salmonis cDNA clone lsal_evj_536_119_fwd 5', mRNA sequence. Length = 552. Score = 44.1 bits (22), Expect = 0.051. Identities = 31/34 (91%). Strand = Plus / Plus

```
Query: 4   agtaaataatacgtgtctctgactaataatcaag 37
           |||||||||||||| |||| ||| ||||||||||
Sbjct: 418 agtaaataatacgtatctcggacaaataatcaag 451
```

>EC437307|EC437307.1 132531662 Sea Urchin Coelomocyte cDNA library Strongylocentrotus purpuratus cDNA clone CSP-1D24 5', mRNA sequence. Length = 769. Score = 38.2 bits (19), Expect = 3.1. Identities = 19/19 (100%). Strand = Plus / Plus

```
Query: 19  tctctgactaataatcaag 37
           |||||||||||||||||||
Sbjct: 511 tctctgactaataatcaag 529
```

>DN568651|DN568651.1 93828849 Sea Urchin primary mesenchyme cell cDNA library Strongylocentrotus purpuratus cDNA clone PMCSPR2-153I12 3', mRNA sequence. Length = 952. Score = 38.2 bits (19), Expect = 3.1. Identities = 19/19 (100%). Strand = Plus / Plus

```
Query: 19  tctctgactaataatcaag 37
           |||||||||||||||||||
Sbjct: 787 tctctgactaataatcaag 805
```

>CD331071|CD331071.1 StrPu537.003894 Sea urchin embryo 20hr blastula Stage cDNA library MPMGp537 Strongylocentrotus purpuratus cDNA clone CALTp537C212;MPI_537_2C21 5', mRNA Sequence. Length = 528. Score = 38.2 bits (19), Expect = 3.1. Identities = 19/19 (100%). Strand = Plus / Minus

```
Query: 19  tctctgactaataatcaag 37
           |||||||||||||||||||
Sbjct: 382 tctctgactaataatcaag 364
```

>CD317783|CD317783.1 StrPu621.002145 Sea urchin unfertilised egg cDNA library MPMGp621 Strongylocentrotus purpuratus cDNA clone MPMGp621E0367;MPI_621_67E3 5', mRNA sequence. Length = 370. Score = 38.2 bits (19), Expect = 3.1. Identities = 19/19 (100%). Strand = Plus / Minus

```
Query: 19  tctctgactaataatcaag 37
           |||||||||||||||||||
Sbjct: 274 tctctgactaataatcaag 256
```

>CD294151|CD294151.1 StrPu537.009183 Sea urchin embryo 20hr blastula Stage cDNA library MPMGp537 Strongylocentrotus purpuratus cDNA clone CALTp537C212;MPI_537_2C21 3', mRNA Sequence. Length = 606. Score = 38.2 bits (19), Expect = 3.1. Identities = 19/19 (100%). Strand = Plus / Plus

```
Query: 19  tctctgactaataatcaag 37
           |||||||||||||||||||
```

Sbjct: 303 tctctgactaataatcaag 321

>CD293564|CD293564.1 StrPu536.007451 Sea urchin embryo 40hr gastrula stage cDNA library MPMGp536 Strongylocentrotus purpuratus cDNA clone CALTp536C148;MPI_536_8C14 5', mRNA sequence. Length = 528. Score = 38.2 bits (19), Expect = 3.1. Identities = 19/19 (100%). Strand = Plus / Minus

```
Query: 19 tctctgactaataatcaag 37
          |||||||||||||||||||
Sbjct: 37 tctctgactaataatcaag 19
```

>JT111880|JT111880.1 TSA: Strongylocentrotus purpuratus WHL22.525353.0 mRNA sequence. Length = 4733. Score = 38.2 bits (19), Expect = 3.1. Identities = 19/19 (100%). Strand = Plus / Minus

```
Query: 19   tctctgactaataatcaag 37
            |||||||||||||||||||
Sbjct: 4202 tctctgactaataatcaag 4184
```

>JK444397|JK444397.1 CTFJA08TU Termite Coptotermes formosanus metatranscriptomic library Coptotermes formosanus cDNA clone CTFJ 3', mRNA sequence. Length = 319. Score = 40.1 bits (20), Expect = 0.80. Identities = 20/20 (100%). Strand = Plus / Plus

```
Query: 17  tgtctctgactaataatcaa 36
           ||||||||||||||||||||
Sbjct: 250 tgtctctgactaataatcaa 269
```

>JK444396|JK444396.1 CTFJA08SA Termite Coptotermes formosanus metatranscriptomic library Coptotermes formosanus cDNA clone CTFJ 5', mRNA sequence. Length = 765. Score = 40.1 bits (20), Expect = 0.80. Identities = 20/20 (100%). Strand = Plus / Minus

```
Query: 17  tgtctctgactaataatcaa 36
           ||||||||||||||||||||
Sbjct: 573 tgtctctgactaataatcaa 554
```

>JK400477|JK400477.1 CTEX644TU Termite Coptotermes formosanus metatranscriptomic library Coptotermes formosanus cDNA clone CTEX 3', mRNA sequence. Length = 413. Score = 40.1 bits (20), Expect = 0.80. Identities = 20/20 (100%). Strand = Plus / Plus

```
Query: 17  tgtctctgactaataatcaa 36
           ||||||||||||||||||||
Sbjct: 120 tgtctctgactaataatcaa 139
```

>JK391306|JK391306.1 CTEV041SA Termite Coptotermes formosanus metatranscriptomic library Coptotermes formosanus cDNA clone CTEV 5', mRNA sequence. Length = 587. Score = 40.1 bits (20), Expect = 0.80. Identities = 20/20 (100%). Strand = Plus / Minus

```
Query: 17  tgtctctgactaataatcaa 36
           ||||||||||||||||||||
```

Sbjct: 573 tgtctctgactaataatcaa 554

>JO991834|JO991834.1 TSA: termite metagenome Contig3713 mRNA Sequence. Length = 903. Score = 40.1 bits (20), Expect = 0.80. Identities = 20/20 (100%). Strand = Plus / Plus

Query: 17 tgtctctgactaataatcaa 36
            ||||||||||||||||||||
Sbjct: 331 tgtctctgactaataatcaa 350

>LL015746|LL015746.1 Trichobilharzia regenti genome assembly T_regenti_v1_0_4, scaffold TRE_scaffold0015739. Length = 10202. Score = 38.2 bits (19), Expect = 3.1. Identities = 19/19 (100%). Strand = Plus / Plus

Query: 1   ccaagtaaataatacgtgt 19
             |||||||||||||||||||
Sbjct: 2161 ccaagtaaataatacgtgt 2179

>AI812962|AI812962.1 2A6 Pine Lambda Zap Xylem library Pinus taeda cDNA, mRNA sequence. Length = 365. Score = 38.2 bits (19), Expect = 3.0; Identities = 19/19 (100%); Strand = Plus / Minus

Query: 17 tgtctctgacaaaaatcaa 35
           |||||||||||||||||||
Sbjct: 57 tgtctctgacaaaaatcaa 39

Table S3c. nblast of CopepodSL-like2a CCAAGCTACACTGCTTGAGTATAACACTTTAAAAG, CopepodSL-like2b CCAAGCTATACTGCTTGTCTAAACACTTTAAAAG and CopepodSL-like2c ATGCTATACTGCTTGTTTAACACTTTAAAAG against DDBJ/EMBL/GenBank nr/nt, EST and TSA databases.

Note 1: Database: nt+tsa+est; 106,461,619 sequences; 119,623,300,248 total letters. Matrix: blastn matrix: 1 -3; Gap Penalties: Existence: 5, Extension: 2.

Note 2: For CopepodSL-like2a, total hits are **45**, including **28** hits to *Calanus finmarchicus* cDNAs; 1 hit to TSA of Fish intestine metagenome RNA; 8 hits for Douglas-fir Pseudotsuga menziesii *Caligus rogercresseyi*, 2 hits to Zebrafish, 1 hit to parasitic nematode *Heligmosomoides polygyrus* sequences, 1 hit to tapeworm *Spirometra erinaceieuropaei*; 1 hit to human, 1 hit to Chimpanzee; 1 hit to earthworm *Perionyx excavates*; 1 hit for society finch *Lonchura striata domestica*.

Note 3: For CopepodSL-like2b, total hits are **28**, including **16** hits to *Calanus finmarchicus* cDNAs; 4 hits to mouse *Mus musculus*, 1 hit to *Absidia idahoensis*; 1 hit to Pinworm *Syphacia muris*; 1 hit to *Cylicostephanus goldi*; 1 hit to pig *Sus scrofa*; 1 hit to *Methanocaldococcus* sp.; 1 hit to *Brassica rapa* subsp. *Pekinensis*; 1 hit to *Trichobilharzia regent*; 1 hit to Zebrafish.

Note 4: For CopepodSL-like2c, total hits are **15**, including **3** hits to *Calanus finmarchicus* cDNAs; 1 hit to *Lymnaea stagnalis; Trichobilharzia regent; Ceraphronidae sp.; Trichoplax adhaerens; Niger Seed Guizotia Abyssinica; Fundulus grandis; Cylindrospermum stagnale; Spirometra erinaceieuropaei; Angiostrongylus cantonensis; Hymenolepis diminuta; Zebrafish; Cyprinus carpio.*

I. CopepodSL-like2a nblast

```
Query= CopepodSL-like2a
>EL586112|EL586112.1 CF_WO0_18h04_SP6 Copepod Whole Organism,
          Normalized Calanus finmarchicus cDNA clone
CF_WO0_18h04
          5', mRNA sequence.
          Length = 684

 Score = 69.9 bits (35), Expect = 2e-09
 Identities = 35/35 (100%)
 Strand = Plus / Plus
```

```
Query: 1  ccaagctacactgcttgagtataacactttaaaag 35
          |||||||||||||||||||||||||||||||||||
Sbjct: 3  ccaagctacactgcttgagtataacactttaaaag 37


>FK671185|FK671185.1 CF_WO1_07C07_SP6 Copepod Whole Organism,
          Normalized Calanus finmarchicus cDNA clone
CF_WO1_07C07
          5', mRNA sequence.
          Length = 650

 Score = 67.9 bits (34), Expect = 6e-09
 Identities = 34/34 (100%)
 Strand = Plus / Plus


Query: 2  caagctacactgcttgagtataacactttaaaag 35
          ||||||||||||||||||||||||||||||||||
Sbjct: 1  caagctacactgcttgagtataacactttaaaag 34


>EH666341|EH666341.1 CF_WO0_01g06_SP6 Copepod Whole Organism,
          Normalized Calanus finmarchicus cDNA clone
CF_WO0_01g06
          5', mRNA sequence.
          Length = 671

 Score = 67.9 bits (34), Expect = 6e-09
 Identities = 34/34 (100%)
 Strand = Plus / Plus


Query: 2  caagctacactgcttgagtataacactttaaaag 35
          ||||||||||||||||||||||||||||||||||
Sbjct: 3  caagctacactgcttgagtataacactttaaaag 36


>EL586060|EL586060.1 CF_WO0_18c07_SP6 Copepod Whole Organism,
          Normalized Calanus finmarchicus cDNA clone
CF_WO0_18c07
          5' similar to XP_969453.1 CG9556-PB, isoform B
          [Tribolium castaneum]. Score=333.95, Expect=3.50E-90,
          mR
          Length = 741

 Score = 65.9 bits (33), Expect = 2e-08
```

```
 Identities = 33/33 (100%)
 Strand = Plus / Plus


Query: 1  ccaagctacactgcttgagtataacactttaaa 33
          |||||||||||||||||||||||||||||||||
Sbjct: 3  ccaagctacactgcttgagtataacactttaaa 35


>FG633006|FG633006.1 CF_WO0_76d05_SP6 Copepod Whole Organism,
          Normalized Calanus finmarchicus cDNA clone
CF_WO0_76d05
          5' similar to ref|XP_320478.4| AGAP012048-PA -
          Anopheles gambiae str. PEST. Score = 246 bits (628),
          Exp
          Length = 622

 Score = 63.9 bits (32), Expect = 9e-08
 Identities = 32/32 (100%)
 Strand = Plus / Plus


Query: 4  agctacactgcttgagtataacactttaaaag 35
          ||||||||||||||||||||||||||||||||
Sbjct: 3  agctacactgcttgagtataacactttaaaag 34


>EL697036|EL697036.1 CF_WO0_29g05_SP6 Copepod Whole Organism,
          Normalized Calanus finmarchicus cDNA clone
CF_WO0_29g05
          5' similar to gb|AAH45492.1| Cyclin B1 - Danio rerio.
          Score = 108 bits (269), Expect = 3e-22, mRNA sequ
          Length = 701

 Score = 63.9 bits (32), Expect = 9e-08
 Identities = 32/32 (100%)
 Strand = Plus / Plus


Query: 4  agctacactgcttgagtataacactttaaaag 35
          ||||||||||||||||||||||||||||||||
Sbjct: 3  agctacactgcttgagtataacactttaaaag 34


>EL586469|EL586469.1 CF_WO0_22g01_SP6 Copepod Whole Organism,
          Normalized Calanus finmarchicus cDNA clone
CF_WO0_22g01
```

```
          5' similar to NP_001071856.1 zinc finger protein
[Ciona
          intestinalis]. Score=56.61, Expect=9.50E-07, mR
          Length = 696

 Score = 63.9 bits (32), Expect = 9e-08
 Identities = 32/32 (100%)
 Strand = Plus / Plus


Query: 4  agctacactgcttgagtataacactttaaaag 35
           ||||||||||||||||||||||||||||||||
Sbjct: 1  agctacactgcttgagtataacactttaaaag 32


>EL586391|EL586391.1 CF_WO0_21h06_SP6 Copepod Whole Organism,
          Normalized Calanus finmarchicus cDNA clone
CF_WO0_21h06
          5' similar to AAH71012.1 MGC81400 protein [Xenopus
          laevis]. Score=58.92, Expect=1.80E-07, mRNA sequence
          Length = 670

 Score = 63.9 bits (32), Expect = 9e-08
 Identities = 32/32 (100%)
 Strand = Plus / Plus


Query: 4  agctacactgcttgagtataacactttaaaag 35
           ||||||||||||||||||||||||||||||||
Sbjct: 3  agctacactgcttgagtataacactttaaaag 34


>EH667067|EH667067.1 CF_WO0_09f10_SP6 Copepod Whole Organism,
          Normalized Calanus finmarchicus cDNA clone
CF_WO0_09f10
          5' similar to ref|XP_624643.2| DIVDICTED: similar to
          ariadne 2 CG5709-PA - Apis mellifera. Score = 95.5
          Length = 688

 Score = 63.9 bits (32), Expect = 9e-08
 Identities = 32/32 (100%)
 Strand = Plus / Plus


Query: 4  agctacactgcttgagtataacactttaaaag 35
           ||||||||||||||||||||||||||||||||
Sbjct: 3  agctacactgcttgagtataacactttaaaag 34
```

```
>GR411197|GR411197.1 CF_WO1_58g05_SP6 Copepod Whole Organism,
          Normalized Calanus finmarchicus cDNA clone
CF_WO1_58g05
          5' similar to ref|XP_002246743.1| hypothetical protein
          BRAFLDRAFT_132189 - Branchiostoma floridae. Scor
          Length = 867

 Score = 61.9 bits (31), Expect = 4e-07
 Identities = 34/35 (97%)
 Strand = Plus / Plus


Query: 1  ccaagctacactgcttgagtataacactttaaaag 35
          |||||||||| ||||||||||||||||||||||||
Sbjct: 1  ccaagctacattgcttgagtataacactttaaaag 35


>FK670642|FK670642.1 CF_WO1_00H07_SP6 Copepod Whole Organism,
          Normalized Calanus finmarchicus cDNA clone
CF_WO1_00H07
          5', mRNA sequence.
          Length = 723

 Score = 61.9 bits (31), Expect = 4e-07
 Identities = 34/35 (97%)
 Strand = Plus / Plus


Query: 1  ccaagctacactgcttgagtataacactttaaaag 35
          |||||||||||||||||| ||||||||||||||||
Sbjct: 14 ccaagctacactgcttgattataacactttaaaag 48


>EL696836|EL696836.1 CF_WO0_27f03_SP6 Copepod Whole Organism,
          Normalized Calanus finmarchicus cDNA clone
CF_WO0_27f03
          5' similar to ref|XP_783505.2| DIVDICTED: hypothetical
          protein - Strongylocentrotus purpuratus. Score =
          Length = 701

 Score = 61.9 bits (31), Expect = 4e-07
 Identities = 31/31 (100%)
 Strand = Plus / Plus
```

```
Query: 5  gctacactgcttgagtataacactttaaaag 35
          |||||||||||||||||||||||||||||||
Sbjct: 1  gctacactgcttgagtataacactttaaaag 31


>EL585465|EL585465.1 CF_WO0_11f11_SP6 Copepod Whole Organism,
          Normalized Calanus finmarchicus cDNA clone
CF_WO0_11f11
          5' similar to XP_970295.1 budding uninhibited by
          benzimidazoles 3 homolog [Tribolium castaneum]. Score=
          Length = 665

 Score = 61.9 bits (31), Expect = 4e-07
 Identities = 34/35 (97%)
 Strand = Plus / Plus


Query: 1  ccaagctacactgcttgagtataacactttaaaag 35
          |||||||||| ||||||||||||||||||||||||
Sbjct: 3  ccaagctacattgcttgagtataacactttaaaag 37


>FK670459|FK670459.1 CF_WO0_98F04_SP6 Copepod Whole Organism,
          Normalized Calanus finmarchicus cDNA clone
CF_WO0_98F04
          5' similar to ref|XP_001605798.1| DIVDICTED: similar
to
          ENSANGP00000012220 - Nasonia vitripennis. Score
          Length = 644

 Score = 58.0 bits (29), Expect = 6e-06
 Identities = 29/29 (100%)
 Strand = Plus / Plus


Query: 7  tacactgcttgagtataacactttaaaag 35
          |||||||||||||||||||||||||||||
Sbjct: 1  tacactgcttgagtataacactttaaaag 29


>FK670621|FK670621.1 CF_WO1_00F09_SP6 Copepod Whole Organism,
          Normalized Calanus finmarchicus cDNA clone
CF_WO1_00F09
          5' similar to emb|CAP19071.1| polymerase (DNA
          directed), delta 2, regulatory subunit [Mus. Score =
          132
          Length = 594
```

```
 Score = 54.0 bits (27), Expect = 9e-05
 Identities = 27/27 (100%)
 Strand = Plus / Plus


Query: 9  cactgcttgagtataacactttaaaag 35
          |||||||||||||||||||||||||||
Sbjct: 1  cactgcttgagtataacactttaaaag 27


>FK670617|FK670617.1 CF_WO1_00F05_SP6 Copepod Whole Organism,
          Normalized Calanus finmarchicus cDNA clone
CF_WO1_00F05
          5' similar to ref|XP_783899.1| DIVDICTED: similar to
          Ribophorin II - Strongylocentrotus purpuratus. Sco
          Length = 641

 Score = 54.0 bits (27), Expect = 9e-05
 Identities = 27/27 (100%)
 Strand = Plus / Plus


Query: 9  cactgcttgagtataacactttaaaag 35
          |||||||||||||||||||||||||||
Sbjct: 1  cactgcttgagtataacactttaaaag 27


>FK040838|FK040838.1 CF_WO0_87C07_SP6 Copepod Whole Organism,
          Normalized Calanus finmarchicus cDNA clone
CF_WO0_87C07
          5' similar to ref|XP_968588.1| DIVDICTED: similar to G
          protein pathway suppressor 1 [Tribolium. Score =
          Length = 630

 Score = 54.0 bits (27), Expect = 9e-05
 Identities = 30/31 (96%)
 Strand = Plus / Plus


Query: 5  gctacactgcttgagtataacactttaaaag 35
          |||||| ||||||||||||||||||||||||
Sbjct: 3  gctacattgcttgagtataacactttaaaag 33


>EL965391|EL965391.1 CF_WO0_45e08_SP6 Copepod Whole Organism,
```

```
          Normalized Calanus finmarchicus cDNA clone
CF_WO0_45e08
          5' similar to ref|XP_975299.1| DIVDICTED: similar to
          CG8258-PA - Tribolium castaneum. Score = 314 bits
          Length = 730

 Score = 54.0 bits (27), Expect = 9e-05
 Identities = 27/27 (100%)
 Strand = Plus / Plus


Query: 9  cactgcttgagtataacactttaaaag 35
           |||||||||||||||||||||||||||
Sbjct: 3  cactgcttgagtataacactttaaaag 29


>EH666730|EH666730.1 CF_WO0_06a05_SP6 Copepod Whole Organism,
          Normalized Calanus finmarchicus cDNA clone
CF_WO0_06a05
          5' similar to ref|XP_392801.3| DIVDICTED: similar to
          CCR4-NOT transcription complex, subunit 10. Score
          Length = 699

 Score = 52.0 bits (26), Expect = 4e-04
 Identities = 32/34 (94%)
 Strand = Plus / Plus


Query: 2  caagctacactgcttgagtataacactttaaaag 35
           |||||||||||| | ||||||||||||||||||
Sbjct: 1  caagctacactgttcgagtataacactttaaaag 34


>FK868247|FK868247.1 CF_WO1_19C03_SP6 Copepod Whole Organism,
          Normalized Calanus finmarchicus cDNA clone
CF_WO1_19C03
          5' similar to ref|XP_001602897.1| DIVDICTED: similar
to
          DEAD box ATP-dependent RNA helicase [Nasonia. S
          Length = 635

 Score = 50.1 bits (25), Expect = 0.001
 Identities = 31/33 (93%)
 Strand = Plus / Plus


Query: 3  aagctacactgcttgagtataacactttaaaag 35
```

```
          |||||||| ||||||||||||||||||||||
Sbjct: 3  aagctacatcgcttgagtataacactttaaaag 35


>EL696588|EL696588.1 CF_WO0_25a04_SP6 Copepod Whole Organism,
          Normalized Calanus finmarchicus cDNA clone
CF_WO0_25a04
          5' similar to gb|EAZ16186.1| hypothetical protein
          OsJ_030395 [Oryza sativa (japonica. Score = 63.9 bits
          Length = 637

 Score = 50.1 bits (25), Expect = 0.001
 Identities = 25/25 (100%)
 Strand = Plus / Plus


Query: 11 ctgcttgagtataacactttaaaag 35
          |||||||||||||||||||||||||
Sbjct: 1  ctgcttgagtataacactttaaaag 25


>FK868361|FK868361.1 CF_WO1_20F05_SP6 Copepod Whole Organism,
          Normalized Calanus finmarchicus cDNA clone
CF_WO1_20F05
          5' similar to dbj|BAF83848.1| unnamed protein product
-
          Homo sapiens. Score = 140 bits (354), Expect =
          Length = 635

 Score = 48.1 bits (24), Expect = 0.006
 Identities = 24/24 (100%)
 Strand = Plus / Plus


Query: 12 tgcttgagtataacactttaaaag 35
          ||||||||||||||||||||||||
Sbjct: 6  tgcttgagtataacactttaaaag 29


>FK671142|FK671142.1 CF_WO1_06G09_SP6 Copepod Whole Organism,
          Normalized Calanus finmarchicus cDNA clone
CF_WO1_06G09
          5' similar to emb|CAI12034.1| MCM3 minichromosome
          maintenance deficient 3 (S. cerevisiae) [Danio. Score
          Length = 697

 Score = 48.1 bits (24), Expect = 0.006
```

```
 Identities = 24/24 (100%)
 Strand = Plus / Plus


Query: 12 tgcttgagtataacactttaaaag 35
          ||||||||||||||||||||||||
Sbjct: 6  tgcttgagtataacactttaaaag 29


>ES237811|ES237811.1 CF_WO0_60c07_SP6 Copepod Whole Organism,
          Normalized Calanus finmarchicus cDNA clone
CF_WO0_60c07
          5' similar to gb|AAK52419.1|AF265346_1 protein kinase
          Chk2 - Danio rerio. Score = 68.9 bits (167), Expe
          Length = 726

 Score = 48.1 bits (24), Expect = 0.006
 Identities = 24/24 (100%)
 Strand = Plus / Plus


Query: 12 tgcttgagtataacactttaaaag 35
          ||||||||||||||||||||||||
Sbjct: 2  tgcttgagtataacactttaaaag 25


>FG985505|FG985505.1 CF_WO0_81G07_SP6 Copepod Whole Organism,
          Normalized Calanus finmarchicus cDNA clone
CF_WO0_81G07
          5' similar to ref|XP_974616.1| DIVDICTED: similar to
          Anaphase Promoting Complex, putative. Score = 156
          Length = 657

 Score = 44.1 bits (22), Expect = 0.087
 Identities = 22/22 (100%)
 Strand = Plus / Plus


Query: 14 cttgagtataacactttaaaag 35
          ||||||||||||||||||||||
Sbjct: 3  cttgagtataacactttaaaag 24


>EL966001|EL966001.1 CF_WO0_52a08_SP6 Copepod Whole Organism,
          Normalized Calanus finmarchicus cDNA clone
CF_WO0_52a08
          5' similar to ref|XP_974549.1| DIVDICTED: similar to
```

```
          transducin (beta)-like 3 (predicted). Score = 136
          Length = 722

 Score = 44.1 bits (22), Expect = 0.087
 Identities = 22/22 (100%)
 Strand = Plus / Plus


Query: 14 cttgagtataacactttaaaag 35
          ||||||||||||||||||||||
Sbjct: 1  cttgagtataacactttaaaag 22


>EH666402|EH666402.1 CF_WO0_02d08_SP6 Copepod Whole Organism,
          Normalized Calanus finmarchicus cDNA clone
CF_WO0_02d08
          5' similar to ref|XP_974549.1| DIVDICTED: similar to
          transducin (beta)-like 3 (predicted). Score = 135
          Length = 742

 Score = 44.1 bits (22), Expect = 0.087
 Identities = 22/22 (100%)
 Strand = Plus / Plus


Query: 14 cttgagtataacactttaaaag 35
          ||||||||||||||||||||||
Sbjct: 38 cttgagtataacactttaaaag 59


>FK867692|FK867692.1 CF_WO1_11F02_SP6 Copepod Whole Organism,
          Normalized Calanus finmarchicus cDNA clone
CF_WO1_11F02
          5', mRNA sequence.
          Length = 570

 Score = 40.1 bits (20), Expect = 1.4
 Identities = 20/20 (100%)
 Strand = Plus / Plus


Query: 16 tgagtataacactttaaaag 35
          ||||||||||||||||||||
Sbjct: 12 tgagtataacactttaaaag 31

>LA164406|LA164406.1 TSA: Fish metagenome RNA, contig:
           contig12763_scaffold12162, isolate: DFGC6.
```

```
          Length = 1584

 Score = 42.1 bits (21), Expect = 0.34
 Identities = 21/21 (100%)
 Strand = Plus / Minus


Query: 3   aagctacactgcttgagtata 23
           |||||||||||||||||||||
Sbjct: 830 aagctacactgcttgagtata 810


>LN003417|LN003417.1 Spirometra erinaceieuropaei genome assembly
            S_erinaceieuropaei, scaffold SPER_scaffold0003390.
          Length = 17406

 Score = 42.1 bits (21), Expect = 0.34
 Identities = 21/21 (100%)
 Strand = Plus / Plus


Query: 15   ttgagtataacactttaaaag 35
            |||||||||||||||||||||
Sbjct: 2638 ttgagtataacactttaaaag 2658




>CT027724|CT027724.7 Zebrafish DNA sequence from clone CH211-
128D6 in
             linkage group 19.
          Length = 146035

 Score = 40.1 bits (20), Expect = 1.4
 Identities = 20/20 (100%)
 Strand = Plus / Minus


Query: 9     cactgcttgagtataacact 28
             ||||||||||||||||||||
Sbjct: 70930 cactgcttgagtataacact 70911


>BX005455|BX005455.8 Zebrafish DNA sequence from clone CH211-
77K19 in
             linkage group 19 Contains the gene for a novel
protein
```

```
             similar to vertebrate calcitonin receptor (CALCR)
and a
             novel gene.
          Length = 164741

 Score = 40.1 bits (20), Expect = 1.4
 Identities = 20/20 (100%)
 Strand = Plus / Minus


Query: 9     cactgcttgagtataacact 28
             ||||||||||||||||||||
Sbjct: 73670 cactgcttgagtataacact 73651


>AL589763|AL589763.6 Human DNA sequence from clone RP11-290P14
on
             chromosome 1.
          Length = 166772

 Score = 40.1 bits (20), Expect = 1.4
 Identities = 20/20 (100%)
 Strand = Plus / Minus


Query: 8     acactgcttgagtataacac 27
             ||||||||||||||||||||
Sbjct: 62535 acactgcttgagtataacac 62516


>BP999014|BP999014.1 Perionyx excavatus mRNA, clone: PER11200.
          Length = 480

 Score = 38.2 bits (19), Expect = 5.4
 Identities = 19/19 (100%)
 Strand = Plus / Plus


Query: 11  ctgcttgagtataacactt 29
           |||||||||||||||||||
Sbjct: 238 ctgcttgagtataacactt 256


>ES428765|ES428765.1 pm_OSU_shoot_CD_2003-04_052A02 Douglas-fir
          cold deacclimating cDNA library 2003-2004 (CD_2003-04)
          Pseudotsuga menziesii var. menziesii cDNA clone
          pm_OSU_WP_CD_052_A02, mRNA sequence.
```

```
          Length = 242

 Score = 38.2 bits (19), Expect = 5.4
 Identities = 22/23 (95%)
 Strand = Plus / Plus


Query: 8  acactgcttgagtataacacttt 30
          |||||| ||||||||||||||||
Sbjct: 69 acactgtttgagtataacacttt 91


>ES427798|ES427798.1 pm_OSU_shoot_CD_2003-04_037A08 Douglas-fir
cold
           deacclimating cDNA library 2003-2004 (CD_2003-04)
           Pseudotsuga menziesii var. menziesii cDNA clone
           pm_OSU_WP_CD_037_A08, mRNA sequence.
          Length = 610

 Score = 38.2 bits (19), Expect = 5.4
 Identities = 22/23 (95%)
 Strand = Plus / Plus


Query: 8   acactgcttgagtataacacttt 30
           |||||| ||||||||||||||||
Sbjct: 90  acactgtttgagtataacacttt 112


>ES426284|ES426284.1 pm_OSU_shoot_CD_2003-04_014E01 Douglas-fir
cold
           deacclimating cDNA library 2003-2004 (CD_2003-04)
           Pseudotsuga menziesii var. menziesii cDNA clone
           pm_OSU_WP_CD_014_E01, mRNA sequence.
          Length = 655

 Score = 38.2 bits (19), Expect = 5.4
 Identities = 22/23 (95%)
 Strand = Plus / Plus


Query: 8   acactgcttgagtataacacttt 30
           |||||| ||||||||||||||||
Sbjct: 110 acactgtttgagtataacacttt 132
```

```
>ES425852|ES425852.1 pm_OSU_shoot_CD_2003-04_008A09 Douglas-fir
cold
           deacclimating cDNA library 2003-2004 (CD_2003-04)
           Pseudotsuga menziesii var. menziesii cDNA clone
           pm_OSU_WP_CD_008_A09, mRNA sequence.
          Length = 628

 Score = 38.2 bits (19), Expect = 5.4
 Identities = 22/23 (95%)
 Strand = Plus / Plus


Query: 8   acactgcttgagtataacacttt 30
           |||||| ||||||||||||||||
Sbjct: 106 acactgtttgagtataacacttt 128


>ES424912|ES424912.1 pm_OSU_shoot_MH_2003-04_051A06 Douglas-fir
          maximum cold hardiness cDNA library 2003-2004
          (MH_2003-04) Pseudotsuga menziesii cDNA clone
          pm_OSU_WP_MD_051_A06, mRNA sequence.
          Length = 397

 Score = 38.2 bits (19), Expect = 5.4
 Identities = 22/23 (95%)
 Strand = Plus / Plus


Query: 8  acactgcttgagtataacacttt 30
          |||||| ||||||||||||||||
Sbjct: 35 acactgtttgagtataacacttt 57


>ES421807|ES421807.1 pm_OSU_shoot_MH_2003-04_002F09 Douglas-fir
           maximum cold hardiness cDNA library 2003-2004
           (MH_2003-04) Pseudotsuga menziesii cDNA clone
           pm_OSU_WP_MD_002_F09, mRNA sequence.
          Length = 592

 Score = 38.2 bits (19), Expect = 5.4
 Identities = 22/23 (95%)
 Strand = Plus / Plus


Query: 8   acactgcttgagtataacacttt 30
           |||||| ||||||||||||||||
Sbjct: 306 acactgtttgagtataacacttt 328
```

```
>ES420868|ES420868.1 pm_OSU_shoot_CA_2003-04_040H03 Douglas-fir
cold
           acclimating cDNA library 2003-2004 (CA_2003-04)
           Pseudotsuga menziesii var. menziesii cDNA clone
           pm_OSU_WP2_CDB_040_H03, mRNA sequence.
          Length = 248

 Score = 38.2 bits (19), Expect = 5.4
 Identities = 22/23 (95%)
 Strand = Plus / Plus


Query: 8   acactgcttgagtataacacttt 30
           |||||| ||||||||||||||||
Sbjct: 86  acactgtttgagtataacacttt 108


>CN640140|CN640140.1 263D09_554799 Douglas-fir cDNA library
          PmIFG_73-6 Pseudotsuga menziesii var. menziesii cDNA
          clone Df263-D09 5, mRNA sequence.
          Length = 591

 Score = 38.2 bits (19), Expect = 5.4
 Identities = 22/23 (95%)
 Strand = Plus / Plus


Query: 8  acactgcttgagtataacacttt 30
          |||||| ||||||||||||||||
Sbjct: 59 acactgtttgagtataacacttt 81


>DC285272|DC285272.1 Lonchura striata domestica mRNA, clone:
           SL26A10, 5'-end sequence, expressed in telencephalon,
           adult male silent cDNA library.
          Length = 751

 Score = 38.2 bits (19), Expect = 5.4
 Identities = 19/19 (100%)
 Strand = Plus / Minus


Query: 17  gagtataacactttaaaag 35
           |||||||||||||||||||
Sbjct: 339 gagtataacactttaaaag 321
```

```
>LL200711|LL200711.1 Heligmosomoides polygyrus genome assembly
            H_bakeri_Edinburgh, scaffold HPBE_scaffold0011439.
          Length = 12582

 Score = 38.2 bits (19), Expect = 5.4
 Identities = 19/19 (100%)
 Strand = Plus / Plus


Query: 6    ctacactgcttgagtataa 24
            |||||||||||||||||||
Sbjct: 6461 ctacactgcttgagtataa 6479


>BS000010|BS000010.1 Pan troglodytes chromosome 22 clone:PTB-
095F11, map
             22, complete sequences.
          Length = 177250

 Score = 38.2 bits (19), Expect = 5.4
 Identities = 19/19 (100%)
 Strand = Plus / Minus


Query: 12    tgcttgagtataacacttt 30
             |||||||||||||||||||
Sbjct: 71467 tgcttgagtataacacttt 71449
```

II. CopepodSL-like2b nblast

```
>FK868231|FK868231.1 CF_WO1_19A05_SP6 Copepod Whole Organism,
          Normalized Calanus finmarchicus cDNA clone
CF_WO1_19A05
          5' similar to gb|EDV35124.1| GF22364 - Drosophila
          ananassae. Score = 263 bits (673), Expect = 4e-69, mR
          Length = 590

 Score = 67.9 bits (34), Expect = 6e-09
 Identities = 34/34 (100%)
 Strand = Plus / Plus


Query: 1  ccaagctatactgcttgtctaaacactttaaaag 34
          ||||||||||||||||||||||||||||||||||
Sbjct: 1  ccaagctatactgcttgtctaaacactttaaaag 34
```

```
>FK868063|FK868063.1 CF_WO1_16D06_SP6 Copepod Whole Organism,
          Normalized Calanus finmarchicus cDNA clone
CF_WO1_16D06
          5', mRNA sequence.
          Length = 637

 Score = 67.9 bits (34), Expect = 6e-09
 Identities = 34/34 (100%)
 Strand = Plus / Plus


Query: 1  ccaagctatactgcttgtctaaacactttaaaag 34
          ||||||||||||||||||||||||||||||||||
Sbjct: 3  ccaagctatactgcttgtctaaacactttaaaag 36


>ES237451|ES237451.1 CF_WO0_56d09_SP6 Copepod Whole Organism,
          Normalized Calanus finmarchicus cDNA clone
CF_WO0_56d09
          5' similar to gb|EAU84110.1| hypothetical protein
          CC1G_06972 - Coprinopsis cinerea okayama7#130. Score
          Length = 736

 Score = 67.9 bits (34), Expect = 6e-09
 Identities = 34/34 (100%)
 Strand = Plus / Plus


Query: 1  ccaagctatactgcttgtctaaacactttaaaag 34
          ||||||||||||||||||||||||||||||||||
Sbjct: 7  ccaagctatactgcttgtctaaacactttaaaag 40


>EL965865|EL965865.1 CF_WO0_50f01_SP6 Copepod Whole Organism,
          Normalized Calanus finmarchicus cDNA clone
CF_WO0_50f01
          5' similar to ref|XP_308774.3| ENSANGP00000015131 -
          Anopheles gambiae str. PEST. Score = 300 bits (769)
          Length = 688

 Score = 67.9 bits (34), Expect = 6e-09
 Identities = 34/34 (100%)
 Strand = Plus / Plus
```

```
Query: 1  ccaagctatactgcttgtctaaacactttaaaag 34
          ||||||||||||||||||||||||||||||||||
Sbjct: 2  ccaagctatactgcttgtctaaacactttaaaag 35
```

```
>EL965448|EL965448.1 CF_WO0_46b07_SP6 Copepod Whole Organism,
          Normalized Calanus finmarchicus cDNA clone
CF_WO0_46b07
          5' similar to ref|XP_001121318.1| DIVDICTED: similar
to
          Mediator complex subunit 20 CG18780-PA [Apis. S
          Length = 695

 Score = 67.9 bits (34), Expect = 6e-09
 Identities = 34/34 (100%)
 Strand = Plus / Plus


Query: 1  ccaagctatactgcttgtctaaacactttaaaag 34
          ||||||||||||||||||||||||||||||||||
Sbjct: 3  ccaagctatactgcttgtctaaacactttaaaag 36


>EL586164|EL586164.1 CF_WO0_19d11_SP6 Copepod Whole Organism,
          Normalized Calanus finmarchicus cDNA clone
CF_WO0_19d11
          5', mRNA sequence.
          Length = 696

 Score = 65.9 bits (33), Expect = 2e-08
 Identities = 33/33 (100%)
 Strand = Plus / Plus


Query: 2  caagctatactgcttgtctaaacactttaaaag 34
          |||||||||||||||||||||||||||||||||
Sbjct: 1  caagctatactgcttgtctaaacactttaaaag 33


>EH666992|EH666992.1 CF_WO0_08g07_SP6 Copepod Whole Organism,
          Normalized Calanus finmarchicus cDNA clone
CF_WO0_08g07
          5' similar to gb|EAW69721.1| WD repeat domain 26,
          isoform CRA_b - Homo sapiens. Score = 79.3 bits (194)
          Length = 635

 Score = 63.9 bits (32), Expect = 9e-08
```

```
 Identities = 32/32 (100%)
 Strand = Plus / Plus


Query: 3  aagctatactgcttgtctaaacactttaaaag 34
          ||||||||||||||||||||||||||||||||
Sbjct: 1  aagctatactgcttgtctaaacactttaaaag 32


>EL965937|EL965937.1 CF_WO0_51d03_SP6 Copepod Whole Organism,
          Normalized Calanus finmarchicus cDNA clone
CF_WO0_51d03
          5' similar to ref|XP_539984.2| DIVDICTED: similar to
          Werner syndrome protein - Canis familiaris. Score
          Length = 684

 Score = 60.0 bits (30), Expect = 1e-06
 Identities = 33/34 (97%)
 Strand = Plus / Plus


Query: 1  ccaagctatactgcttgtctaaacactttaaaag 34
          |||||||||||||||||| |||||||||||||||
Sbjct: 3  ccaagctatactgcttgtttaaacactttaaaag 36


>EL773299|EL773299.1 CF_WO0_35a03_SP6 Copepod Whole Organism,
          Normalized Calanus finmarchicus cDNA clone
CF_WO0_35a03
          5' similar to ref|XP_966746.1| DIVDICTED: similar to
          proteasome 26S non-ATPase subunit 12. Score = 215
          Length = 602

 Score = 52.0 bits (26), Expect = 3e-04
 Identities = 32/34 (94%)
 Strand = Plus / Plus


Query: 1  ccaagctatactgcttgtctaaacactttaaaag 34
          |||||||| ||||||||||||| |||||||||||
Sbjct: 2  ccaagctacactgcttgtctaaccactttaaaag 35


>EH667020|EH667020.1 CF_WO0_09b01_SP6 Copepod Whole Organism,
          Normalized Calanus finmarchicus cDNA clone
CF_WO0_09b01
          5', mRNA sequence.
```

```
          Length = 707

 Score = 52.0 bits (26), Expect = 3e-04
 Identities = 32/34 (94%)
 Strand = Plus / Plus


Query: 1  ccaagctatactgcttgtctaaacactttaaaag 34
          |||||||| ||||||||||||| |||||||||||
Sbjct: 1  ccaagctacactgcttgtctaaccactttaaaag 34


>FK670861|FK670861.1 CF_WO1_03F07_SP6 Copepod Whole Organism,
          Normalized Calanus finmarchicus cDNA clone
CF_WO1_03F07
          5' similar to ref|XP_001814448.1| DIVDICTED: similar
to
          MGC81609 protein - Tribolium castaneum. Score =
          Length = 612

 Score = 48.1 bits (24), Expect = 0.005
 Identities = 24/24 (100%)
 Strand = Plus / Plus


Query: 11 ctgcttgtctaaacactttaaaag 34
          ||||||||||||||||||||||||
Sbjct: 3  ctgcttgtctaaacactttaaaag 26


>FK868068|FK868068.1 CF_WO1_16D11_SP6 Copepod Whole Organism,
          Normalized Calanus finmarchicus cDNA clone
CF_WO1_16D11
          5' similar to ref|XP_001864663.1| alanine
          aminotransferase - Culex pipiens quinquefasciatus.
          Score = 27
          Length = 656

 Score = 46.1 bits (23), Expect = 0.020
 Identities = 23/23 (100%)
 Strand = Plus / Plus


Query: 12 tgcttgtctaaacactttaaaag 34
          |||||||||||||||||||||||
Sbjct: 2  tgcttgtctaaacactttaaaag 24
```

```
>FK040994|FK040994.1 CF_WO0_89B04_SP6 Copepod Whole Organism,
          Normalized Calanus finmarchicus cDNA clone
CF_WO0_89B04
          5' similar to ref|XP_624100.2| DIVDICTED: similar to
          CG18028-PA.3 - Apis mellifera. Score = 176 bits (4
          Length = 655

 Score = 44.1 bits (22), Expect = 0.081
 Identities = 22/22 (100%)
 Strand = Plus / Plus


Query: 13 gcttgtctaaacactttaaaag 34
          ||||||||||||||||||||||
Sbjct: 2  gcttgtctaaacactttaaaag 23


>EL697212|EL697212.1 CF_WO0_31f01_SP6 Copepod Whole Organism,
          Normalized Calanus finmarchicus cDNA clone
CF_WO0_31f01
          5' similar to ref|XP_974845.1| DIVDICTED: similar to
          kaptin (actin binding protein) [Tribolium. Score =
          Length = 636

 Score = 44.1 bits (22), Expect = 0.081
 Identities = 22/22 (100%)
 Strand = Plus / Plus


Query: 13 gcttgtctaaacactttaaaag 34
          ||||||||||||||||||||||
Sbjct: 1  gcttgtctaaacactttaaaag 22


>EH666382|EH666382.1 CF_WO0_02b12_SP6 Copepod Whole Organism,
          Normalized Calanus finmarchicus cDNA clone
CF_WO0_02b12
          5' similar to gb|AAA74020.1| ribonuleotide reductase
          small subunit. Score = 258 bits (659), Expect = 2e
          Length = 724

 Score = 44.1 bits (22), Expect = 0.081
 Identities = 29/30 (96%), Gaps = 1/30 (3%)
 Strand = Plus / Plus
```

```
Query: 5  gctatactgcttgtctaaacactttaaaag 34
          |||||||||||||||| |||||||||||||
Sbjct: 1  gctatactgcttgtct-aacactttaaaag 29


>EL965670|EL965670.1 CF_WO0_48e06_SP6 Copepod Whole Organism,
          Normalized Calanus finmarchicus cDNA clone
CF_WO0_48e06
          5', mRNA sequence.
          Length = 735

 Score = 42.1 bits (21), Expect = 0.32
 Identities = 21/21 (100%)
 Strand = Plus / Plus


Query: 14 cttgtctaaacactttaaaag 34
          |||||||||||||||||||||
Sbjct: 3  cttgtctaaacactttaaaag 23


>LK023313|LK023313.1 Absidia idahoensis var. thermophila genome
assembly
               Lramosa_hybrid_454_Illumina, scaffold SCAF1;.
          Length = 3088551

 Score = 40.1 bits (20), Expect = 1.3
 Identities = 23/24 (95%)
 Strand = Plus / Minus


Query: 2       caagctatactgcttgtctaaaca 25
               ||||||||||| ||||||||||||
Sbjct: 1999566 caagctatactacttgtctaaaca 1999543


>LK996261|LK996261.1 Syphacia muris genome assembly
S_muris_Valencia
           , scaffold SMUV_scaffold0000217.
          Length = 91601

 Score = 40.1 bits (20), Expect = 1.3
 Identities = 20/20 (100%)
 Strand = Plus / Minus


Query: 11   ctgcttgtctaaacacttta 30
```

```
            ||||||||||||||||||||
Sbjct: 6904 ctgcttgtctaaacacttta 6885


>LL361766|LL361766.1 Cylicostephanus goldi genome assembly
            C_goldi_Cheshire, scaffold CGOC_scaffold0000498.
          Length = 4976

 Score = 40.1 bits (20), Expect = 1.3
 Identities = 23/24 (95%)
 Strand = Plus / Plus


Query: 10   actgcttgtctaaacactttaaaa 33
            |||||||||| |||||||||||||
Sbjct: 2701 actgcttgtcaaaacactttaaaa 2724


>AL671865|AL671865.11 Mouse DNA sequence from clone RP23-312B13
on
             chromosome X.
          Length = 226471

 Score = 40.1 bits (20), Expect = 1.3
 Identities = 20/20 (100%)
 Strand = Plus / Plus


Query: 14    cttgtctaaacactttaaaa 33
             ||||||||||||||||||||
Sbjct: 47062 cttgtctaaacactttaaaa 47081


>CJ022279|CJ022279.1 Sus scrofa mRNA, clone:SPL01F070103, 5'
end,
           expressed in spleen.
          Length = 831

 Score = 38.2 bits (19), Expect = 5.0
 Identities = 19/19 (100%)
 Strand = Plus / Minus


Query: 7   tatactgcttgtctaaaca 25
           |||||||||||||||||||
Sbjct: 509 tatactgcttgtctaaaca 491
```

```
>CP001901|CP001901.1 Methanocaldococcus sp. FS406-22, complete
genome.
          Length = 1760939

 Score = 38.2 bits (19), Expect = 5.0
 Identities = 19/19 (100%)
 Strand = Plus / Plus


Query: 15     ttgtctaaacactttaaaa 33
              |||||||||||||||||||
Sbjct: 491190 ttgtctaaacactttaaaa 491208


>AC189400|AC189400.2 Brassica rapa subsp. pekinensis clone
KBrB056L15,
              complete sequence.
          Length = 117834

 Score = 38.2 bits (19), Expect = 5.0
 Identities = 22/23 (95%)
 Strand = Plus / Plus


Query: 9      tactgcttgtctaaacactttaa 31
              |||| ||||||||||||||||||
Sbjct: 109572 tacttcttgtctaaacactttaa 109594


>LL062426|LL062426.1 Trichobilharzia regenti genome assembly
            T_regenti_v1_0_4, scaffold TRE_scaffold0060888.
          Length = 3034

 Score = 38.2 bits (19), Expect = 5.0
 Identities = 19/19 (100%)
 Strand = Plus / Plus


Query: 15   ttgtctaaacactttaaaa 33
            |||||||||||||||||||
Sbjct: 2459 ttgtctaaacactttaaaa 2477


>BX649487|BX649487.7 Zebrafish DNA sequence from clone DKEY-
148C4 in
             linkage group 15.
```

```
          Length = 201933

 Score = 38.2 bits (19), Expect = 5.0
 Identities = 22/23 (95%)
 Strand = Plus / Plus


Query: 12    tgcttgtctaaacactttaaaag 34
             ||||| |||||||||||||||||
Sbjct: 13284 tgcttatctaaacactttaaaag 13306


>AC158678|AC158678.6 Mus musculus 6 BAC RP23-142I6 (Roswell Park
Cancer
              Institute (C57BL/6J Female) Mouse BAC Library)
complete
              sequence.
          Length = 217586

 Score = 38.2 bits (19), Expect = 5.0
 Identities = 19/19 (100%)
 Strand = Plus / Plus


Query: 8      atactgcttgtctaaacac 26
              |||||||||||||||||||
Sbjct: 126347 atactgcttgtctaaacac 126365


>AC153630|AC153630.1 Mus musculus 6 BAC RP23-289O16 (Roswell
Park Cancer
              Institute (C57BL/6J Female) Mouse BAC Library)
complete
              sequence.
          Length = 217496

 Score = 38.2 bits (19), Expect = 5.0
 Identities = 19/19 (100%)
 Strand = Plus / Minus


Query: 8      atactgcttgtctaaacac 26
              |||||||||||||||||||
Sbjct: 176882 atactgcttgtctaaacac 176864
```

```
>AC138359|AC138359.8 Mus musculus chromosome 1, clone RP24-
383H12,
             complete sequence.
          Length = 217891

 Score = 38.2 bits (19), Expect = 5.0
 Identities = 19/19 (100%)
 Strand = Plus / Plus


Query: 15    ttgtctaaacactttaaaa 33
             |||||||||||||||||||
Sbjct: 66645 ttgtctaaacactttaaaa 66663
```

III. CopepodSL-like2c nblast

```
>EL585416|EL585416.1 CF_WO0_11b10_SP6 Copepod Whole Organism,
          Normalized Calanus finmarchicus cDNA clone
CF_WO0_11b10
          5' similar to NP_524429.1 tetracycline resistance
          CG5760-PA [Drosophila melanogaster]. Score=149.06, Ex
          Length = 715

 Score = 61.9 bits (31), Expect = 3e-07
 Identities = 31/31 (100%)
 Strand = Plus / Plus


Query: 1  atgctatactgcttgtttaacactttaaaag 31
          |||||||||||||||||||||||||||||||
Sbjct: 1  atgctatactgcttgtttaacactttaaaag 31


>EH666382|EH666382.1 CF_WO0_02b12_SP6 Copepod Whole Organism,
          Normalized Calanus finmarchicus cDNA clone
CF_WO0_02b12
          5' similar to gb|AAA74020.1| ribonuleotide reductase
          small subunit. Score = 258 bits (659), Expect = 2e
          Length = 724

 Score = 50.1 bits (25), Expect = 0.001
 Identities = 28/29 (96%)
 Strand = Plus / Plus


Query: 3  gctatactgcttgtttaacactttaaaag 31
          |||||||||||||| ||||||||||||||
Sbjct: 1  gctatactgcttgtctaacactttaaaag 29
```

```
>EL965937|EL965937.1 CF_WO0_51d03_SP6 Copepod Whole Organism,
          Normalized Calanus finmarchicus cDNA clone
CF_WO0_51d03
          5' similar to ref|XP_539984.2| DIVDICTED: similar to
          Werner syndrome protein - Canis familiaris. Score
          Length = 684

 Score = 44.1 bits (22), Expect = 0.065
 Identities = 29/30 (96%), Gaps = 1/30 (3%)
 Strand = Plus / Plus


Query: 3  gctatactgcttgttt-aacactttaaaag 31
          |||||||||||||||| |||||||||||||
Sbjct: 7  gctatactgcttgtttaaacactttaaaag 36


>ES574196|ES574196.1 FPS0114.C7_M18 LSG10-01 Lymnaea stagnalis
cDNA
           clone FPS0114_M18 3', mRNA sequence.
          Length = 889

 Score = 40.1 bits (20), Expect = 1.0
 Identities = 23/24 (95%)
 Strand = Plus / Minus


Query: 7   tactgcttgtttaacactttaaaa 30
           |||| |||||||||||||||||||
Sbjct: 686 tacttcttgtttaacactttaaaa 663


>LL034897|LL034897.1 Trichobilharzia regenti genome assembly
            T_regenti_v1_0_4 ,scaffold TRE_scaffold0034717.
          Length = 5624

 Score = 40.1 bits (20), Expect = 1.0
 Identities = 20/20 (100%)
 Strand = Plus / Plus


Query: 12   cttgtttaacactttaaaag 31
            ||||||||||||||||||||
Sbjct: 2886 cttgtttaacactttaaaag 2905
```

```
>HO080193|HO080193.1 ms_bjs_1a98_1_34_cera122_e03.nt
Ceraphronidae
          sp. whole body female library Ceraphronidae sp.
          BJS-2010 cDNA 5', mRNA sequence.
          Length = 555

 Score = 38.2 bits (19), Expect = 4.0
 Identities = 19/19 (100%)
 Strand = Plus / Minus


Query: 12 cttgtttaacactttaaaa 30
          |||||||||||||||||||
Sbjct: 51 cttgtttaacactttaaaa 33


>GR951537|GR951537.1 Trc_1267860.b Trichoplax adhaerens EST
Library
           Trichoplax adhaerens cDNA, mRNA sequence.
          Length = 987

 Score = 38.2 bits (19), Expect = 4.0
 Identities = 19/19 (100%)
 Strand = Plus / Plus


Query: 9   ctgcttgtttaacacttta 27
           |||||||||||||||||||
Sbjct: 165 ctgcttgtttaacacttta 183


>GE559053|GE559053.1 CCHT17129.b1_A12.ab1 CCHT Niger Seed
Guizotia
           abyssinica cDNA clone CCHT17129, mRNA sequence.
          Length = 806

 Score = 38.2 bits (19), Expect = 4.0
 Identities = 19/19 (100%)
 Strand = Plus / Minus


Query: 11  gcttgtttaacactttaaa 29
           |||||||||||||||||||
Sbjct: 681 gcttgtttaacactttaaa 663
```

```
>JW566977|JW566977.1 TSA: Fundulus grandis
          Locus_28820_Transcript_1/1 mRNA sequence.
          Length = 695

 Score = 38.2 bits (19), Expect = 4.0
 Identities = 22/23 (95%)
 Strand = Plus / Minus


Query: 9  ctgcttgtttaacactttaaaag 31
           |||| ||||||||||||||||||
Sbjct: 98 ctgcatgtttaacactttaaaag 76


>CP003642|CP003642.1 Cylindrospermum stagnale PCC 7417, complete
genome.
          Length = 7003560

 Score = 38.2 bits (19), Expect = 4.0
 Identities = 19/19 (100%)
 Strand = Plus / Minus


Query: 12      cttgtttaacactttaaaa 30
                |||||||||||||||||||
Sbjct: 3943307 cttgtttaacactttaaaa 3943289


>LK945835|LK945835.1 Angiostrongylus cantonensis genome assembly
              A_cantonensis_China ,scaffold
ACAC_scaffold0000254.
          Length = 109488

 Score = 38.2 bits (19), Expect = 4.0
 Identities = 19/19 (100%)
 Strand = Plus / Plus


Query: 12     cttgtttaacactttaaaa 30
              |||||||||||||||||||
Sbjct: 105877 cttgtttaacactttaaaa 105895


>LN205535|LN205535.1 Spirometra erinaceieuropaei genome assembly
            S_erinaceieuropaei ,scaffold SPER_scaffold0173721.
          Length = 1717
```

```
 Score = 38.2 bits (19), Expect = 4.0
 Identities = 19/19 (100%)
 Strand = Plus / Minus


Query: 13   ttgtttaacactttaaaag 31
            |||||||||||||||||||
Sbjct: 1021 ttgtttaacactttaaaag 1003


>LM383521|LM383521.1 Hymenolepis diminuta genome assembly
              H_diminuta_Denmark ,scaffold HDID_scaffold0000072.
          Length = 151207

 Score = 38.2 bits (19), Expect = 4.0
 Identities = 19/19 (100%)
 Strand = Plus / Plus


Query: 8      actgcttgtttaacacttt 26
              |||||||||||||||||||
Sbjct: 100716 actgcttgtttaacacttt 100734


>BX511225|BX511225.7 Zebrafish DNA sequence from clone DKEY-
28J24 in
             linkage group 21.
          Length = 256675

 Score = 38.2 bits (19), Expect = 4.0
 Identities = 19/19 (100%)
 Strand = Plus / Plus


Query: 8     actgcttgtttaacacttt 26
             |||||||||||||||||||
Sbjct: 61205 actgcttgtttaacacttt 61223


>LN590685|LN590685.1 Cyprinus carpio genome assembly common carp
genome
              ,scaffold LG16.
          Length = 20606094

 Score = 38.2 bits (19), Expect = 4.0
 Identities = 22/23 (95%)
 Strand = Plus / Plus
```

```
Query: 9       ctgcttgtttaacactttaaaag 31
               ||||||||||| ||||||||||||
Sbjct: 7623748 ctgcttgttttacactttaaaag 7623770
```

Table S4a. Codon usage of the full-length cDNAs in *Acartia pacifica*

#Coding GC 51.69%
#1st letter GC 51.77%
#2nd letter GC 39.76%
#3rd letter GC 63.56%

#Codon	AA	Fraction	Frequency	Number
GCA	A	0.120	8.810	827
GCC	A	0.439	32.269	3029
GCG	A	0.124	9.151	859
GCT	A	0.317	23.310	2188
TGC	C	0.575	10.920	1025
TGT	C	0.425	8.075	758
GAC	D	0.561	28.391	2665
GAT	D	0.439	22.255	2089
GAA	E	0.284	17.354	1629
GAG	E	0.716	43.785	4110
TTC	F	0.593	21.509	2019
TTT	F	0.407	14.787	1388
GGA	G	0.252	16.513	1550
GGC	G	0.369	24.194	2271
GGG	G	0.094	6.136	576
GGT	G	0.285	18.665	1752
CAC	H	0.669	14.414	1353
CAT	H	0.331	7.138	670
ATA	I	0.113	5.827	547
ATC	I	0.522	26.910	2526
ATT	I	0.365	18.846	1769
AAA	K	0.230	21.232	1993
AAG	K	0.770	71.154	6679
CTA	L	0.070	5.732	538
CTC	L	0.183	14.957	1404
CTG	L	0.401	32.748	3074
CTT	L	0.137	11.229	1054
TTA	L	0.051	4.176	392
TTG	L	0.158	12.923	1213
ATG	M	1.000	27.326	2565
AAC	N	0.696	29.467	2766
AAT	N	0.304	12.848	1206
CCA	P	0.191	8.310	780
CCC	P	0.384	16.747	1572
CCG	P	0.187	8.139	764
CCT	P	0.239	10.398	976
CAA	Q	0.215	7.404	695
CAG	Q	0.785	27.017	2536
AGA	R	0.212	13.828	1298
AGG	R	0.265	17.269	1621
CGA	R	0.063	4.091	384
CGC	R	0.188	12.230	1148
CGG	R	0.086	5.582	524
CGT	R	0.186	12.113	1137

AGC	S	0.218	14.052	1319
AGT	S	0.120	7.777	730
TCA	S	0.119	7.660	719
TCC	S	0.251	16.214	1522
TCG	S	0.098	6.349	596
TCT	S	0.194	12.518	1175
ACA	T	0.203	10.930	1026
ACC	T	0.437	23.544	2210
ACG	T	0.111	5.966	560
ACT	T	0.249	13.434	1261
GTA	V	0.080	5.508	517
GTC	V	0.267	18.302	1718
GTG	V	0.370	25.408	2385
GTT	V	0.283	19.410	1822
TGG	W	1.000	10.803	1014
TAC	Y	0.689	20.156	1892
TAT	Y	0.311	9.087	853
TAA	*	0.539	3.611	339
TAG	*	0.229	1.534	144
TGA	*	0.232	1.555	146

S4b. Codon usage of the full-length cDNAs in *Pseudodiaptomus poplesia.*

#Coding GC 52.66%

#1st letter GC 51.96%

#2nd letter GC 40.49%

#3rd letter GC 65.53%

#Codon	AA	Fraction	Frequency	Number
GCA	A	0.138	10.995	710
GCC	A	0.493	39.366	2542
GCG	A	0.070	5.575	360
GCT	A	0.300	23.988	1549
TGC	C	0.621	10.128	654
TGT	C	0.379	6.179	399
GAC	D	0.615	29.563	1909
GAT	D	0.385	18.475	1193
GAA	E	0.281	15.873	1025
GAG	E	0.719	40.589	2621
TTC	F	0.664	23.601	1524
TTT	F	0.336	11.94	771
GGA	G	0.331	24.793	1601
GGC	G	0.335	25.087	1620
GGG	G	0.068	5.064	327
GGT	G	0.266	19.962	1289
CAC	H	0.678	13.953	901

CAT	H	0.322	6.628	428
ATA	I	0.059	3.004	194
ATC	I	0.603	30.709	1983
ATT	I	0.338	17.19	1110
AAA	K	0.185	18.444	1191
AAG	K	0.815	81.24	5246
CTA	L	0.045	3.484	225
CTC	L	0.268	20.597	1330
CTG	L	0.326	25.072	1619
CTT	L	0.162	12.435	803
TTA	L	0.032	2.431	157
TTG	L	0.166	12.776	825
ATG	M	1.000	28.061	1812
AAC	N	0.758	30.151	1947
AAT	N	0.242	9.648	623
CCA	P	0.194	8.781	567
CCC	P	0.443	20.024	1293
CCG	P	0.114	5.141	332
CCT	P	0.249	11.243	726
CAA	Q	0.224	7.557	488
CAG	Q	0.776	26.172	1690
AGA	R	0.222	15.269	986
AGG	R	0.348	23.942	1546
CGA	R	0.054	3.701	239
CGC	R	0.155	10.623	686
CGG	R	0.044	3.02	195
CGT	R	0.177	12.188	787
AGC	S	0.200	11.32	731
AGT	S	0.083	4.692	303
TCA	S	0.129	7.294	471
TCC	S	0.331	18.785	1213
TCG	S	0.06	3.391	219
TCT	S	0.198	11.243	726
ACA	T	0.153	7.867	508
ACC	T	0.554	28.556	1844
ACG	T	0.066	3.407	220
ACT	T	0.227	11.723	757
GTA	V	0.064	4.46	288
GTC	V	0.351	24.468	1580
GTG	V	0.301	20.937	1352
GTT	V	0.284	19.76	1276
TGG	W	1.000	10.484	677
TAC	Y	0.728	21.882	1413

TAT	Y	0.272	8.192	529
TAA	*	0.604	4.15	268
TAG	*	0.239	1.642	106
TGA	*	0.158	1.084	70
